# Metabolites and Immune Cells Mediated the Causal Relationship Between the Gut Microbiota and Osteosarcoma: A Mendelian Randomization Study

**DOI:** 10.1002/hsr2.71430

**Published:** 2025-10-30

**Authors:** Guanjun Chen, Zhenyu Song, Imtiaz Abdullah, Xinyu Wang, Chunjiang Zhu, Jincheng Huang

**Affiliations:** ^1^ Affiliated Hospital of Guilin Medical University Guilin Guangxi China; ^2^ The Second Affiliated Hospital of Guilin Medical University Guilin Guangxi China; ^3^ Department of Orthopedics Guilin Medical University Guilin Guangxi China; ^4^ Department of Orthopedics Henan Provincial People's Hospital, Zhengzhou University People's Hospital Zhengzhou Henan China

**Keywords:** gut microbiota, immune cells, Mendelian randomization, osteosarcoma, plasma metabolites

## Abstract

**Background and Aims:**

Osteosarcoma (OS) is characterized by a primary malignant bone tumor with high local invasion and metastasis potential. The role of gut microbiota (GM) in the development of OS remains poorly understood. This study aims to investigate the relationship between GM, plasma metabolites, immune cells, and OS utilizing Mendelian randomization (MR).

**Methods:**

Summary data from large‐scale Genome‐Wide Association Studies (GWAS) involving GM, plasma metabolites, immune cells, and OS cases were applied. A two‐sample MR approach was used to evaluate the causal effects among these factors. The analysis was performed by using the inverse variance weighting (IVM) method, MR‐Egger, and other sensitivity analyses to ensure the reliability of results. Furthermore, mediation analysis was employed to elucidate the mediating roles of metabolites and immune cells in the relationship between GM and OS.

**Results:**

MR identified six gut microbial traits that are causally linked to OS, including three metabolic pathways and three taxa. Pathways involved in amino acid metabolism were associated with increased OS risk. In contrast, the Bacteroidaceae family appeared protective, whereas the genus *Flavonifractor* was linked to a higher risk. MR also identified 12 plasma metabolites associated with OS; seven were protective (including 3‐carboxy‐4‐methyl‐5‐propyl‐2‐furanpropanoate [CMPF]) and two increased risk. The l‐isoleucine biosynthesis pathway (PWY‐3001) was associated with lower CMPF levels, whereas *Flavonifractor* was associated with higher CMPF. Mediation analysis showed that CMPF partially mediated the effect of PWY‐3001 on OS (~35%) but not that of *Flavonifractor*. Nine immune cell phenotypes were linked to OS (five positively, four inversely). *Flavonifractor plautii* (*F. plautii*) was associated with an increased proportion of CX3CR1+ monocytes, which in turn increased OS risk; these monocytes mediated ~5% of *F. plautii*'s effect on OS.

**Conclusions:**

This study provides evidence supporting the involvement of GM, plasma metabolites, and immune cells play critical roles in the development of OS.

## 1 Introduction

Osteosarcoma (OS) is a primary malignant bone tumor originating from mesenchymal tissue. It is the most prevalent form of primary bone malignancy and is characterized by a high tendency for local invasion and metastasis [1]. Despite the significant improvements in the prognosis of OS patients achieved through the combination of surgery and chemotherapy, the prognosis for metastatic or recurrent OS remains unsatisfactory, with the survival rate for metastatic OS patients being < 5 years [2]. These clinical challenges highlight the urgent need to elucidate novel etiological factors and molecular mechanisms driving OS progression. Previous studies have explored potential etiological factors—including radiation exposure, mutations in tumor suppressor genes, and exposure to alkylating agents—and further elucidated their mechanistic implications. For example, germline alterations in tumor suppressor genes, such as RB1 mutations in hereditary retinoblastoma and TP53 mutations in Li–Fraumeni syndrome, have been shown to significantly increase OS incidence, indicating a genetic predisposition to tumor initiation [3].

In recent years, the gut microbiota (GM) has emerged as a key regulator in cancer biology, exerting profound effects on host metabolism and immune surveillance [4]. These two mechanisms are now recognized as key mediators linking the GM to tumor initiation and progression. Accumulating evidence indicates that microbial metabolites, such as short‐chain fatty acids, can modulate immune surveillance and inflammatory pathways, while GM‐driven immune cell regulation may influence tumor‐associated macrophage polarization and T‐cell activation, thereby reshaping the osteosarcoma microenvironment [5, 6, 7]. However, whether the GM directly drives the onset of OS, and its potential metabolic and immune regulatory mechanisms, remain unclear. Furthermore, existing studies predominantly focus on the correlation analysis of the GM, lacking systematic causal inference, with very few investigations delving into the complex interactions between the microbiota, metabolites, and immune cells [8, 9, 10]. Additionally, how to comprehensively explore the interactions between these factors remains a significant challenge in current research.

Mendelian randomization (MR), which leverages genetic variants as instrumental variables (IVs), provides a robust tool for causal inference by minimizing confounding and reverse causation. This approach is particularly suited to disentangling the complex GM–metabolite–immune cell network in OS, where conventional observational studies are prone to bias [11, 12]. Therefore, the present study aims to assess the causal relationships between the GM, metabolites, immune cells, and OS, and to elucidate whether metabolites and immune cells mediate the effects of the GM on OS development. By integrating large‐scale genomic data within a two‐sample MR framework, we provide novel insights into the multilayered mechanisms through which the GM may influence OS pathogenesis.

## 2 Materials and Methods

### 2.1 Data Sources and Research Design

This study utilizes summary data from large‐scale Genome‐Wide Association Studies (GWAS) and adopts a two‐sample MR framework to systematically evaluate the causal relationship between the gut microbiome and OS, with a particular focus on the mediating roles of metabolites and immune cells. Detailed information regarding all databases involved in this study can be found in Table 1. (As this analysis is based entirely on publicly available summary‐level data, ethical approval is not required. All original GWAS studies included in our analysis obtained informed consent and ethical approval from their respective institutions.)

**Table 1:** Database information.

Phenotypes	Sample sizes	Data source	Phenotypic code	Ancestry
Exposure				
Gut microbiota	7738	DMP [13]	GCST90027446 to GCST90027857	European
Mediator				
Plasma metabolites	8299	Chen et al. [14]	GCST90199621 to GCST90201020	Canada
Immune cells	3757	Orru et al. [15]	ebi‐a‐GCST90001391 to ebi‐a‐GCST90002121	European
Outcome				
Osteosarcoma	345,187	FinnGen	finngen_R11_C3_OSTEOSARCOMA_EXALLC	European

#### 2.1.1 Gut Microbiota Data

The GM (and pathway) data utilized in this study originate from the Dutch Microbiome Project (DMP) and its associated metagenomic sequencing data (GWAS ID: GCST90027446–GCST90027857). The data set encompasses 7738 healthy individuals, providing 207 microbiota taxonomic units (ranging from phylum to species level). The microbiota abundance was log‐transformed and subsequently standardized. A lenient significance threshold (*p* < 1 × 10^−5^) was applied for the selection of IVs to balance statistical power with potential pleiotropic risks. Linkage disequilibrium (LD) clustering was performed using PLINK v1.9 (window size = 10,000 kb, *r* ^2^ < 0.001), and single‐nucleotide polymorphisms (SNPs) within the MHC region on chromosome 6 (chr6: 28,477,897–33,448,354) were excluded to avoid autoimmune‐related confounding.

#### 2.1.2 Osteosarcoma Data

The case‐control data for OS were extracted from the 11th release (R11) of the FinnGen consortium database, which includes 69 OS patients diagnosed by histopathological examination and 345,118 healthy controls (ICD‐10 code: C40). All cases were validated through the Finnish National Health Registry, ensuring the accuracy of the phenotypic data.

#### 2.1.3 Metabolite and Immune Cell Data

##### 2.1.3.1 Plasma Metabolites

This data set includes summary GWAS data for 1400 metabolites (ID: GCST90199621–GCST90201020), covering metabolic pathways related to lipids, amino acids, carbohydrates, and other compounds.

##### 2.1.3.2 Immune Cell Phenotypes

Data from the European Bioinformatics Institute (EBI) GWAS (ID: ebi‐a‐GCST90001391 to ebi‐a‐GCST90002121) were used, covering 731 immune‐related traits. These include absolute cell counts (e.g., CD4+ T cells), relative proportions (e.g., CD8+/CD4+ ratio), and surface antigen expression levels (e.g., HLA‐DR+ monocytes).

### 2.2 Genetic Instrument Selection

**Figure 1:**
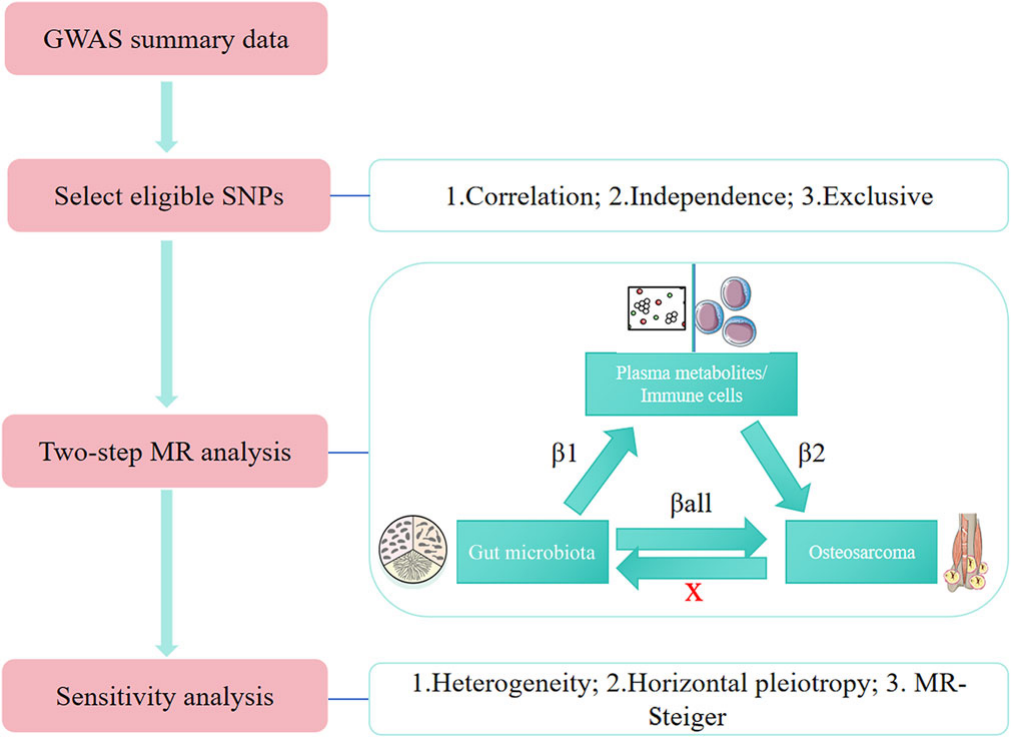
Overview of the MR study design. This study investigates the potential causal relationships between 207 GM (and pathways), 1400 metabolites, 731 immune cells, and OS using a two‐sample MR approach.

We applied rigorous standards in selecting IVs. All IVs were required to meet the three core assumptions of MR: (1) strong association with the exposure (relevance), (2) no association with confounding factors (independence), and (3) affecting the outcome solely through the exposure (exclusivity). The specific quality control steps are illustrated in Figure 1.

In forward MR, the GM, metabolites, and immune cells all employ a threshold of *p* < 1 × 10^−5^ for selection, ensuring a more comprehensive set of IVs. In reverse MR, when OS is considered as the exposure, the threshold for IV selection is set to *p* < 5 × 10^−5^. Allele frequency alignment is performed using the 1000 Genomes European reference panel, and SNPs with unclear linkage are excluded. For the GM, immune cells, and reverse MR with OS as the exposure, the window size is set to 10,000 kb with an *r* ^2^ < 0.001. Due to the large volume of metabolite data, stricter LD control is necessary; thus, we set the window size for metabolites as 1000 kb with an *r* ^2^ < 0.001. Furthermore, weak IVs are excluded by applying the *F*‐statistic (*F* = *β* ^2^/se^2^) > 10. Detailed information regarding these SNPs is provided in Supporting Information S3: Table 1.

### 2.3 Causal Effect Estimation and Sensitivity Analysis

#### 2.3.1 Primary Analytical Method

We employed the inverse variance weighting (IVW) method as the default for the random effects model to estimate causal effects through weighted regression slopes. This approach assumes that all IVs are free from pleiotropy.

Supplementary Methods: MR‐Egger Regression: This method allows for the inclusion of an intercept term to quantify directional pleiotropy, contingent upon the InSIDE assumption (independence between IVs and pleiotropic effects); Weighted Median Method: This provides robust estimates under the assumption that more than 50% of the IVs are valid; Mode‐based Estimation (Simple/Weighted Mode): This technique reduces heterogeneity by clustering SNP effect sizes.

#### 2.3.2 Effect Size Standardization

In this study, the binary outcome (OS) is expressed using odds ratios (OR) and 95% confidence intervals (CI). For continuous exposures (e.g., microbiome abundance), standardized *β* coefficients are utilized, representing log(OR) per standard deviation change.

#### 2.3.3 Sensitivity Analysis

##### 2.3.3.1 Heterogeneity Test

Cochran's *Q* test (*p* < 0.05 indicates heterogeneity, random effects model applied); Horizontal Pleiotropy Test: MR‐Egger intercept test (*p* > 0.05 indicates no pleiotropy); MR‐PRESSO Global Test and Outlier Correction: Applied to detect and correct for outliers; MR‐Steiger Analysis: Ensures the direction of the overall causal effect is consistent.

### 2.4 Multilevel Mediation Effect Validation

To elucidate the potential causal pathways from “gut microbiota–metabolites/immune cells–OS,” we employed a two‐step MR approach (Figure 1).

#### 2.4.1 Step 1

MR is used to evaluate the causal effect of the GM on mediators (metabolites or immune cells) (*β*_1_), with significant associations (*p* < 0.05) being selected.

#### 2.4.2 Step 2

The mediators identified in Step 1 are treated as exposures to assess their causal effects on OS (*β*_2_).

#### 2.4.3 Mediation Effect Size

The product term *β*_1_ × *β*_2_ is calculated, and standard errors and 95% CIs are estimated using the Delta method or nonparametric bootstrap (1000 repetitions).

#### 2.4.4 Mediation Proportion

Defined as (*β*_1_ × *β*_2_)/Total Effect (*β*_all_), where *β*_all_ represents the direct MR estimate of the causal effect of the GM on OS.

### 2.5 Statistical Analysis

This study adhered to the methodological framework for MR established by Davies et al. [16], with reporting compliant to the STROBE‐MR guidelines [17]. All analyses were conducted using R statistical software (version 4.3.2; R Foundation for Statistical Computing) with the following key packages: TwoSampleMR (v0.5.7) for MR analyses; MRPRESSO (v1.0) for pleiotropy correction; Mediation (v4.5.0) for mediation effect estimation; forestplot (v3.1.1) for data visualization. All *p* values were directly obtained from R software output, without any numerical rounding.

Prespecified primary analyses employed bidirectional MR to infer causality, using IVW regression with random‐effects modeling as the principal method. Sensitivity analyses implemented MR‐Egger regression (testing directional pleiotropy), weighted median estimation, and mode‐based estimation. We applied a two‐sided significance threshold (*α* = 0.05), with IVs validated via minimum *F*‐statistic > 10 (excluding weak instruments) and LD clumping (*r* ^2^ < 0.001; window size = 10 Mb). The statistical testing framework is shown in Table 2. The definition of key terms is shown in Table 3. Statistical reporting follows SAMPL guidelines [18].

**Table 2:** Statistical testing framework.

Test type	Method	Threshold	Sidedness
Primary causal effect	IVW regression	*p* < 0.05	Two‐sided
Heterogeneity	Cochran's *Q* test	*p* < 0.05^a^	—
Directional pleiotropy	MR‐Egger intercept test	*p* < 0.05	Two‐sided

^a^ Indicates use of random‐effects IVW model when significant.

**Table 3:** Definition of key terms.

Term	Abbr./Symbol	Definition
Primary MR estimator	IVW	Inverse‐variance weighted method for causal effect estimation
Standardized effect size	*β*	Standardized regression coefficient for continuous exposures
Odds ratio	OR	Effect estimate for binary outcomes
Confidence interval	95% CI	Interval containing true parameter with 95% probability
Instrument strength statistic	*F*‐stat	Quantifies instrumental variable strength (*F* < 10 indicates weak instruments, *F* = *β*_exposure_^2^/SE_exposure_^2^)
Linkage disequilibrium	LD	Nonrandom association between genetic variants (clumping threshold: *r* ^2^ < 0.001)
Directional pleiotropy test	MR‐Egger	Regression‐based method detecting/correcting directional pleiotropy

Exploratory analyses included: mediation pathway testing via two‐step MR (gut microbiota → mediator → osteosarcoma) with effect decomposition calculated as *β*_1_ × *β*_2_ using direct product methods.

## 3 Results

### 3.1 Causal Relationship Between Gut Microbiota (and Pathways) and Osteosarcoma

**Figure 2:**
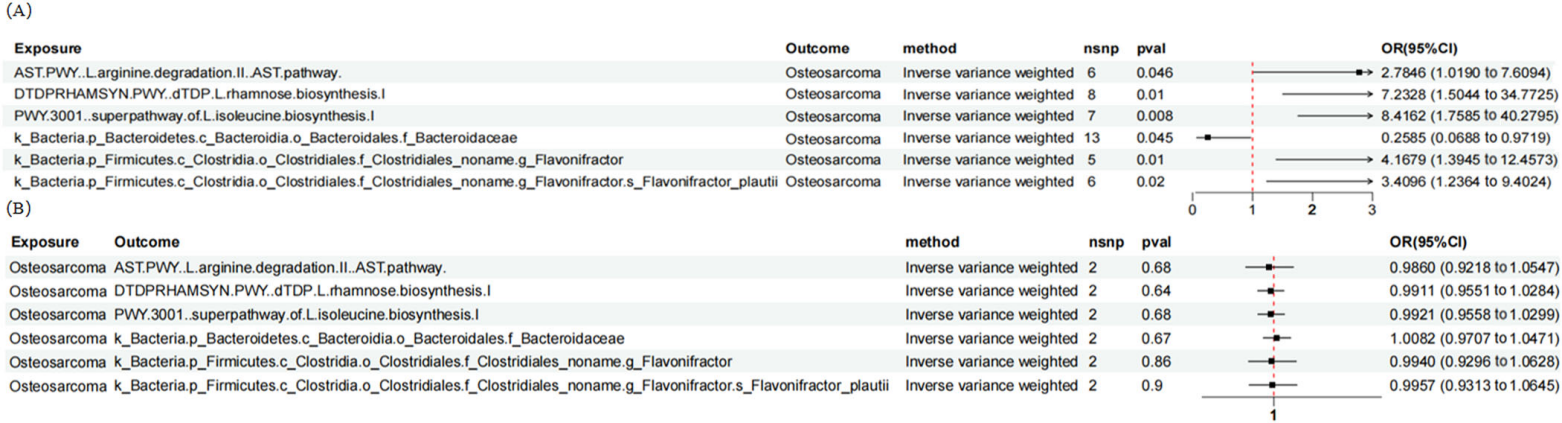
Forest plot of the bidirectional causal relationship between GM and OS. (A) The impact of GM on OS risk; (B) the reverse causal relationship of OS on GM.

We conducted MR analysis to identify six distinct GM (and pathways) that exhibit a causal relationship with the risk of OS (IVW method, Figure 2A). Among these, three microbial‐associated metabolic pathways were identified: the l‐arginine degradation II pathway‐related microbiota (AST.PWY) increased the risk of OS (OR = 2.78, 95% CI: 1.02–7.61, *p* = 0.046); the dTDP‐l‐rhamnose biosynthesis pathway‐related microbiota (DTDPRHAMSYN.PWY) increased the OS risk (OR = 7.23, 95% CI: 1.50–34.77, *p* = 0.01); and the microbiota related to l‐isoleucine biosynthesis, PWY‐3001 (with *Escherichia coli* [*E. coli*] as the core species), increased the OS risk (OR = 8.42, 95% CI: 1.76–40.28, *p* = 0.008).

Additionally, three bacterial taxa were found to have a causal effect on OS risk: the Bacteroidaceae family was identified as a protective factor (OR = 0.26, 95% CI: 0.07–0.97, *p* = 0.045), whereas the genus *Flavonifractor*, responsible for flavonoid degradation, was found to be a risk factor (OR = 4.17, 95% CI: 1.39–12.46, *p* = 0.01). Furthermore, *Flavonifractor plautii* (*F. plautii*), a species within this genus, also emerged as a significant risk factor, with its abundance positively correlated with OS risk (OR = 3.41, 95% CI: 1.24–9.4, *p* = 0.02). The full MR analysis results are available in Supporting Information S3: Table 2, and the forest plot of the final causal estimates is shown in Supporting Information S1: Figure 1. The scatter plot in Supporting Information S2: Figure 2 illustrates the causal effects of SNPs on the exposure and outcome.

Sensitivity analyses revealed no significant heterogeneity (Cochran's *Q p* > 0.05, Supporting Information S3: Table 3) or pleiotropy (MR‐Egger intercept *p* > 0.05, Supporting Information S3: Table 4). A reverse MR analysis ruled out reverse causality (IVW method, *p* > 0.05, Figure 2B). The associations between GM abundance and OS risk remained robust after MR‐PRESSO correction (*p* > 0.05, Supporting Information S3: Table 6). After excluding pleiotropy, heterogeneity, and reverse causality, these six GM (and pathways) were found to demonstrate a unidirectional causal relationship with OS.

### 3.2 Causal Relationship Between Metabolites‐Mediated Gut Microbiota and Osteosarcoma

#### 3.2.1 Genetic Association of Metabolites With Osteosarcoma

**Figure 3:**
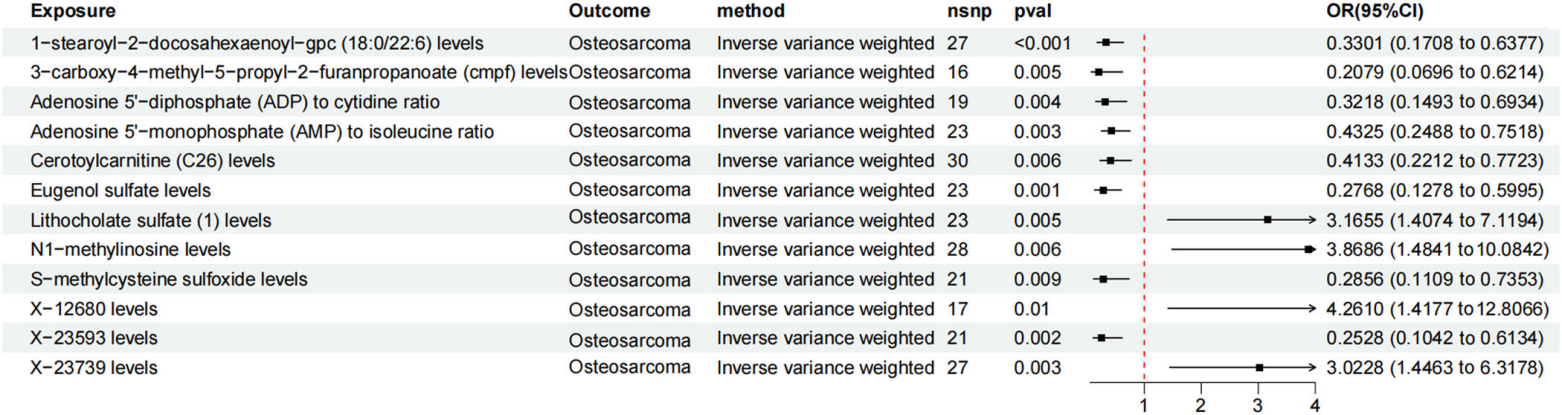
Forest plot illustrating the causal relationship between metabolites and OS.

Our study reveals a compelling genetic link between metabolites and OS risk, identified within a comprehensive set of 1400 plasma metabolites. A total of 12 metabolites were found to be significantly associated with the risk of OS. These include: one lipid, one organic acid, three nucleotides, one amino acid derivative, one fatty acid metabolite, one bile acid, one plant‐derived metabolite, and three unidentified compounds (using the IVW method, *p* < 0.01; Figure 3). Among these, two metabolites were associated with an increased risk of OS: Lithocholate sulfate (1) levels (OR = 3.17, 95% CI: 1.41–7.12, *p* = 0.005) and N1‐methylinosine levels (OR = 3.87, 95% CI: 1.48–10.08, *p* = 0.006). In contrast, seven metabolites were found to act as protective factors against OS: 1‐stearoyl‐2‐docosahexaenoyl‐gpc (18:0/22:6) levels (OR = 0.33, 95% CI: 0.17–0.64, *p* < 0.001), 3‐carboxy‐4‐methyl‐5‐propyl‐2‐furanpropanoate (cmpf) levels (OR = 0.21, 95% CI: 0.07–0.62, *p* = 0.005), Adenosine 5′‐diphosphate (ADP) to cytidine ratio (OR = 0.32, 95% CI: 0.15–0.69, *p* = 0.004), Adenosine 5′‐monophosphate (AMP) to isoleucine ratio (OR = 0.43, 95% CI: 0.25–0.75, *p* = 0.003), Cerotoylcarnitine (C26) levels (OR = 0.41, 95% CI: 0.22–0.77, *p* = 0.006), Eugenol sulfate levels (OR = 0.28, 95% CI: 0.13–0.60, *p* = 0.001), S‐methylcysteine sulfoxide levels (OR = 0.29, 95% CI: 0.11–0.74, *p* = 0.009). Importantly, no significant heterogeneity (Supporting Information S3: Table 3) or pleiotropy (Supporting Information S3: Table 4) was detected in these results.

#### 3.2.2 Regulatory Relationship Between Osteosarcoma‐Associated Microbiota and Metabolites

**Figure 4:**
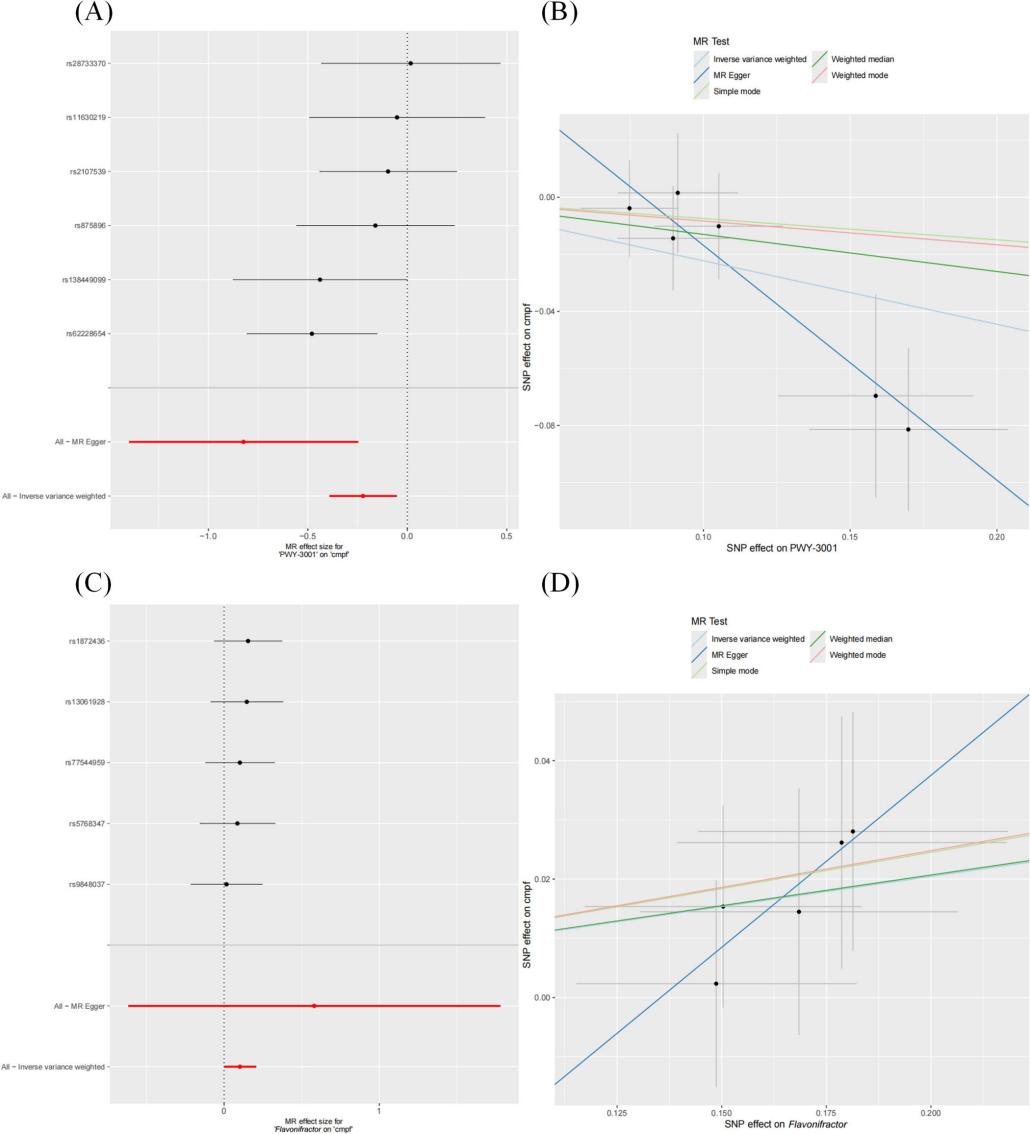
Forest and scatter plots illustrating the relationship between OS‐associated GM and metabolites. (A and B) The forest and scatter plots showing the relationship between the microbiota metabolic pathway PWY‐3001 involved in l‐isoleucine biosynthesis and the metabolite CMPF, respectively. (C and D) The forest and scatter plots showing the relationship between the *Flavonifractor* genus and CMPF.

In this study, we examined the regulatory effects of 6 OS‐associated GM (and associated pathways) on 12 metabolites. Ultimately, we identified causal relationships between two GM (and pathways) and one metabolite, as depicted in Figure 4. The microbiota metabolic pathway involved in l‐isoleucine biosynthesis, PWY‐3001 (with *E. coli* as the core species), exhibited a negative correlation with the CMPF level (OR = 0.8, 95% CI: 0.68–0.95, *p* = 0.01), while the *Flavonifractor* genus, associated with flavonoid degradation, was positively correlated with CMPF levels (OR = 1.11, 95% CI: 1.0003–1.23, *p* = 0.049). Notably, no significant heterogeneity (Supporting Information S3: Table 3) or pleiotropy (Supporting Information S3: Table 4) was detected in the above results.

#### 3.2.3 The Mediating Role of Metabolites in the Gut Microbiome–Osteosarcoma Association

**Figure 5:**
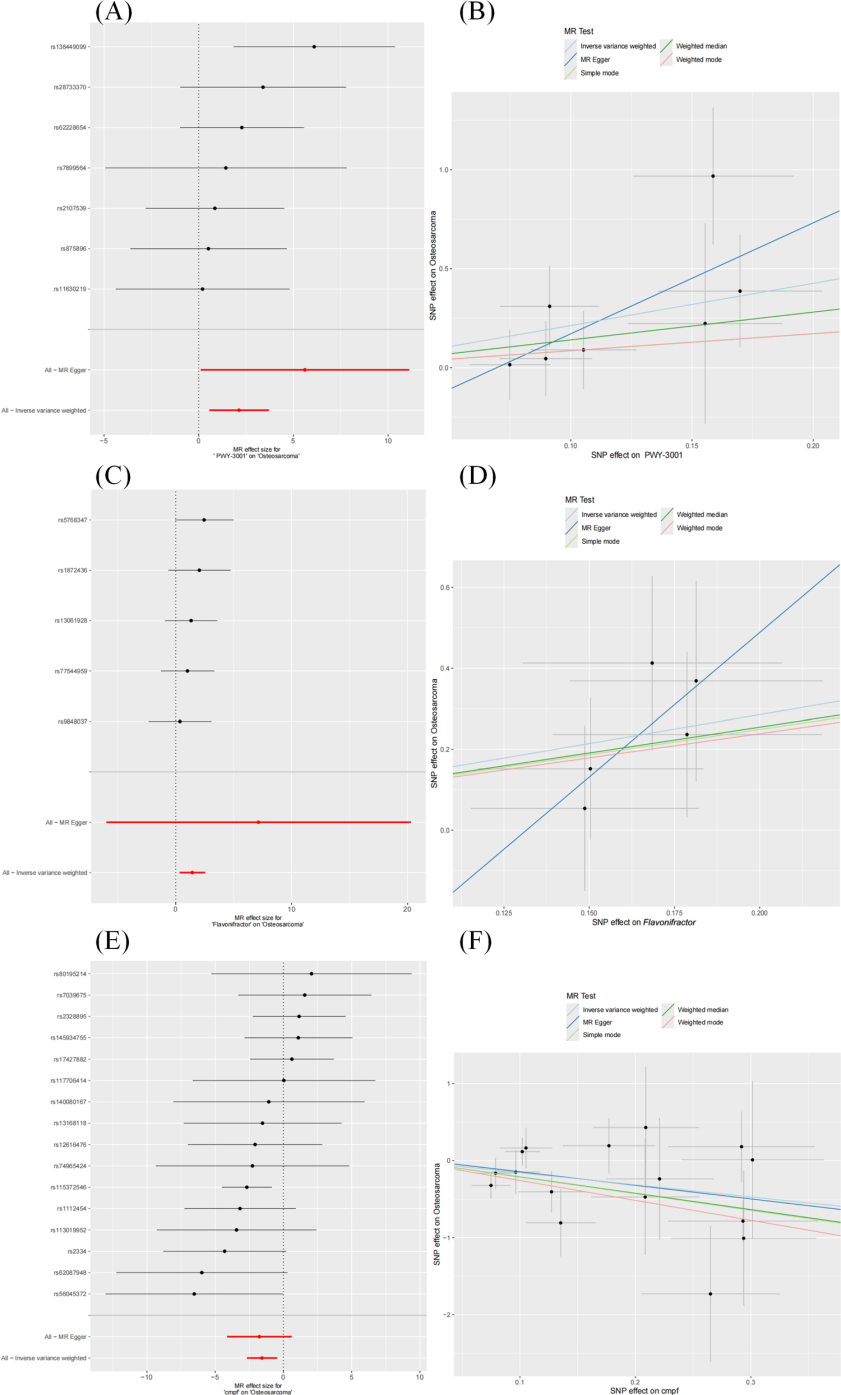
Forest plots and scatter plots showing significant causal effects on OS in MR analysis. (A and B) The impact of PWY‐3001 on OS risk. (C and D) The effect of *Flavonifractor* on OS risk. (E and F) The impact of cmpf metabolite levels on OS risk.

The findings indicate a positive correlation between the microbiome metabolic pathway PWY‐3001, involved in l‐isoleucine biosynthesis (with *E. coli* as the core species), flavonoid‐degrading bacteria (*Flavonifractor*), and OS (Figure 5A–D). On the other hand, the metabolite cmpf demonstrates a negative correlation with OS risk (Figure 5E,F). These GM and metabolites appear to be linked through a potential causal relationship (Figure 4). Results from heterogeneity, pleiotropy tests, and the MR‐Steiger causality direction test are presented in Table 4.

**Table 4:** Results of heterogeneity, pleiotropy tests, and MR‐Steiger causal direction test for some MR positive results.

Exposures	Outcomes	*Q* from IVW	*Q* from MR‐Egger	*P*val_Q from IVW	*P*val_Q from MR‐Egger	MR‐Steiger	*P*val of MR‐Egger interception
PWY‐3001	Osteosarcoma	5.47	3.8	0.48	0.58	True	0.25
*Flavonifractor*	Osteosarcoma	1.58	0.85	0.81	0.84	True	0.45
*Flavonifractor plautii*	Osteosarcoma	2.5	1.54	0.78	0.82	True	0.38
cmpf	Osteosarcoma	17.13	17.09	0.31	0.25	True	0.86
CX3CR1 on CD14− CD16−	Osteosarcoma	16.58	16.36	0.55	0.5	True	0.65
PWY‐3001	cmpf	2.58	1.04	0.35	0.9	True	0.1
*Flavonifractor*	cmpf	0.93	0.31	0.92	0.96	True	0.49
*Flavonifractor plautii*	CX3CR1 on CD14− CD16−	4.52	4.49	0.48	0.34	True	0.87

**Figure 6:**
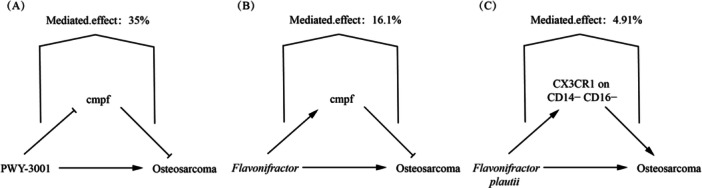
Visualization of mediation analysis results. (A) The indirect pathway through which PWY‐3001 influences OS risk by modulating cmpf metabolite levels. (B) The indirect pathway through which *Flavonifractor* influences OS risk by modulating cmpf metabolite levels. (C) The indirect pathway through which *Flavonifractor plautii* influences OS risk by modulating the levels of immune cell CX3CR1 on CD14− CD16− cells.

When evaluating the metabolites as mediators, we observed that while *Flavonifractor*, a risk factor for OS, elevates cmpf levels, cmpf itself is negatively correlated with OS risk (Figure 10B). Thus, it is unlikely that cmpf mediates the increased OS risk induced by the elevated abundance of *Flavonifractor*. The microbiome pathway PWY‐3001, involved in l‐isoleucine biosynthesis (with *E. coli* as the core species), shows a risk effect on OS mediated by cmpf (Figure 10A). Specifically, PWY‐3001, a risk factor for OS, may increase OS risk by downregulating cmpf levels. The mediating effects are shown in Figure 6A,B and Supporting Information S3: Table 5. MR analysis demonstrates genetically predicted associations of PWY‐3001 (35% effect mediation) and *Flavonifractor* (16.1% effect mediation) with osteosarcoma risk through CMPF, suggesting potential involvement of the microbiome in tumorigenesis via metabolic reprogramming.

### 3.3 Causal Relationship Between Immune Cells, Gut Microbiota, and Osteosarcoma

#### 3.3.1 Genetic Association Between Immune Cells and Osteosarcoma

**Figure 7:**
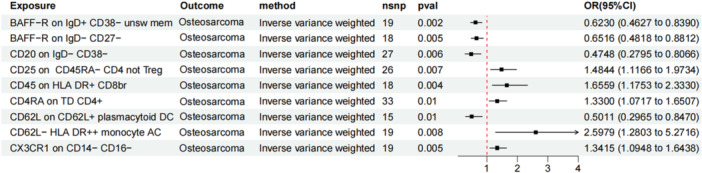
Forest plot of the causal relationship between immune cells and OS.

Our findings reveal that among 731 immune cell phenotypes, 9 are significantly associated with OS risk (IVW method, Figure 7). Of these, five exhibit a positive correlation with OS: CD25 on CD45RA− CD4 not Treg (OR = 1.48, 95% CI: 1.12–1.97, *p* = 0.007); CD45 on HLA DR+ CD8br (OR = 1.66, 95% CI: 1.18–2.33, *p* = 0.004); CD4RA on TD CD4+ (OR = 1.33, 95% CI: 1.07–1.65, *p* = 0.01); CD62L− HLA DR++ monocyte AC (OR = 2.6, 95% CI: 1.28–5.27, *p* = 0.008); CX3CR1 on CD14− CD16− (OR = 1.34, 95% CI: 1.09–1.64, *p* = 0.005). Conversely, four immune cell phenotypes are negatively associated with OS: BAFF‐R on IgD+ CD38− unsw mem (OR = 0.62, 95% CI: 0.46–0.84, *p* = 0.002); BAFF‐R on IgD− CD27− (OR = 0.65, 95% CI: 0.48–0.88, *p* = 0.005); CD20 on IgD− CD38− (OR = 0.47, 95% CI: 0.28–0.81, *p* = 0.006); CD62L on CD62L+ plasmacytoid DC (OR = 0.5, 95% CI: 0.3–0.85, *p* = 0.01). None of the results revealed significant heterogeneity (Supporting Information S3: Table 3) or pleiotropy (Supporting Information S3: Table 4).

#### 3.3.2 Regulatory Relationship Between Osteosarcoma‐Associated Microbiota and Immune Cells

**Figure 8:**
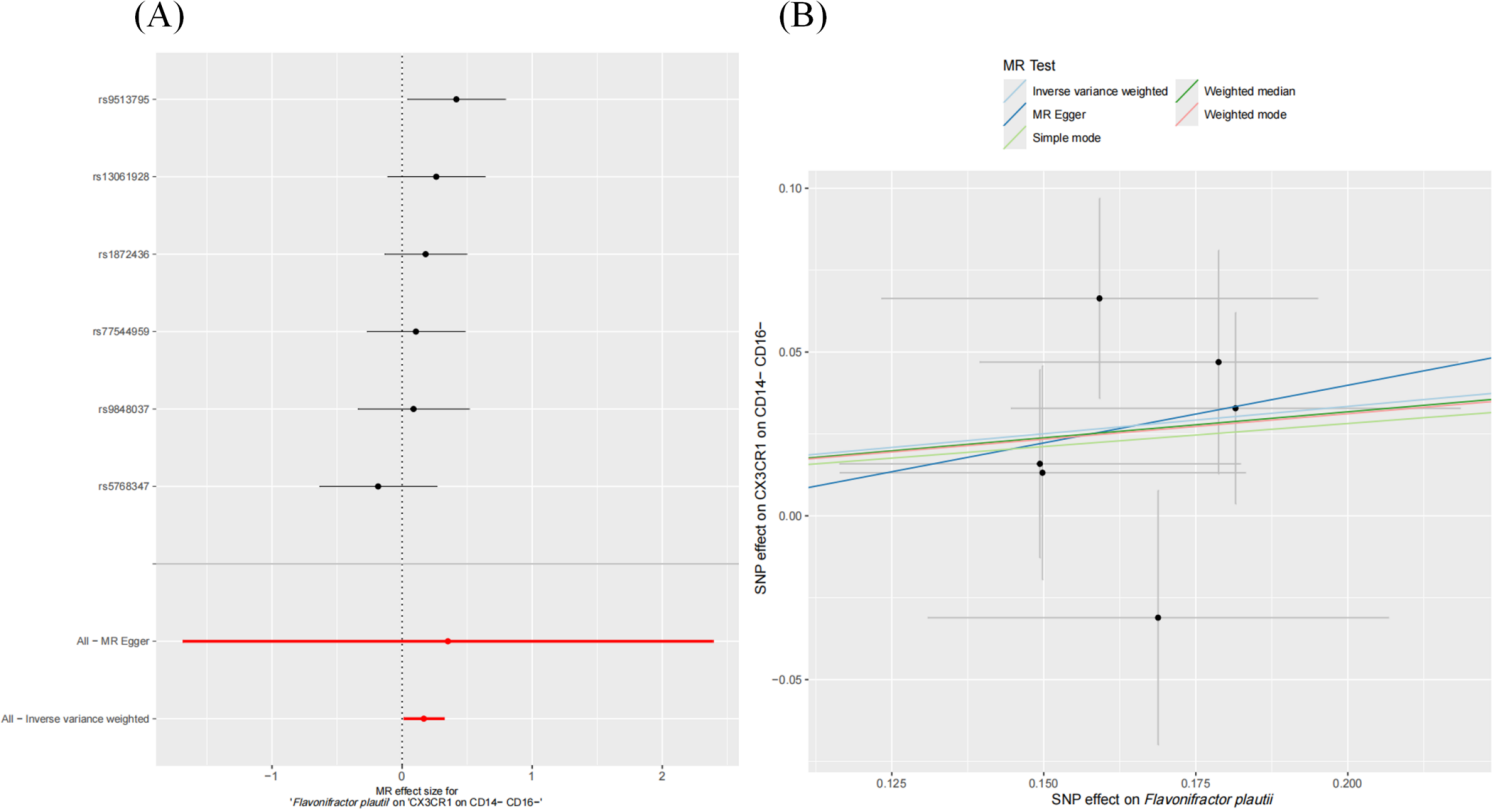
Forest plot and scatter plot illustrating the relationship between OS‐associated GM and immune cells. (A and B) The forest plot and scatter plot showing the relationship between *Flavonifractor plautii* and CX3CR1 expression on the CD14− CD16− immune cell subset.

We conducted an in‐depth investigation into the impact of six OS‐associated GM (and associated pathways) on nine distinct immune cell populations. Our findings revealed a causal relationship between one specific microbiota and one immune cell subset, as illustrated in Figure 8. Notably, *F. plautii*, a flavonoid‐degrading bacterium, was found to be positively correlated with the expression level of CX3CR1 on the CD14− CD16− subset of monocytes (OR = 1.18, 95% CI: 1.01–1.38, *p* = 0.03). No significant heterogeneity (Supporting Information S3: Table 3) or pleiotropy (Supporting Information S3: Table 4) was observed.

#### 3.3.3 The Mediating Role of Immune Cells in the Gut Microbiome–Osteosarcoma Association

**Figure 9:**
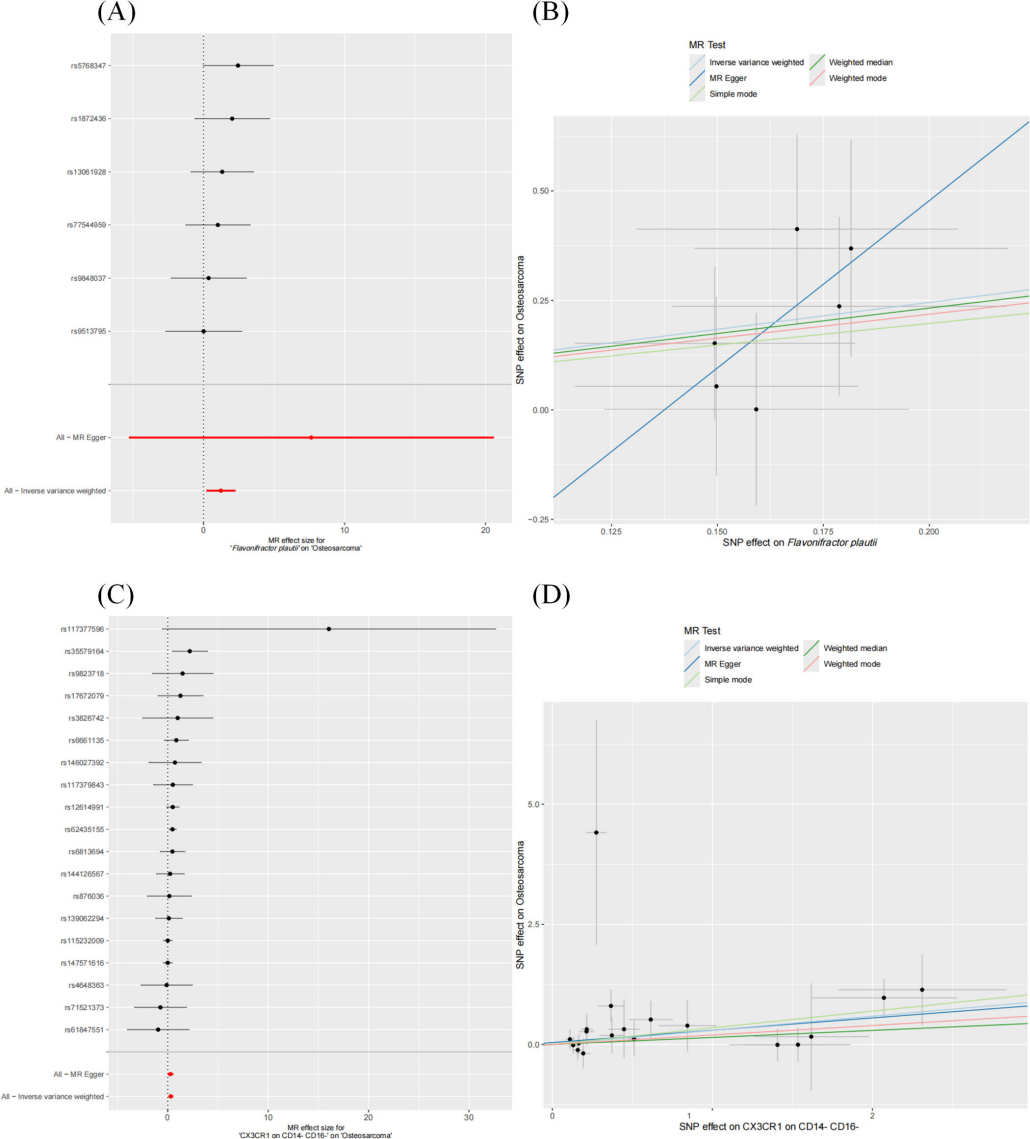
Forest and scatter plots from MR analysis showing significant causal effects on OS. (A and B) The influence of *Flavonifractor plautii* on OS risk; (C and D) The influence of immune cells expressing CX3CR1 on CD14− CD16− on OS risk.

The findings indicate a positive correlation between *F. plautii*, a flavonoid‐degrading bacterium, and OS (Figure 9A,B). Additionally, the level of immune cells expressing CX3CR1 on CD14− CD16− is positively associated with OS risk (Figure 9C,D). A potential causal relationship exists between the gut microbiome and immune cells (Figure 8). The results of heterogeneity, pleiotropy tests, and the MR‐Steiger causal direction test are presented in Table 4.

**Figure 10:**
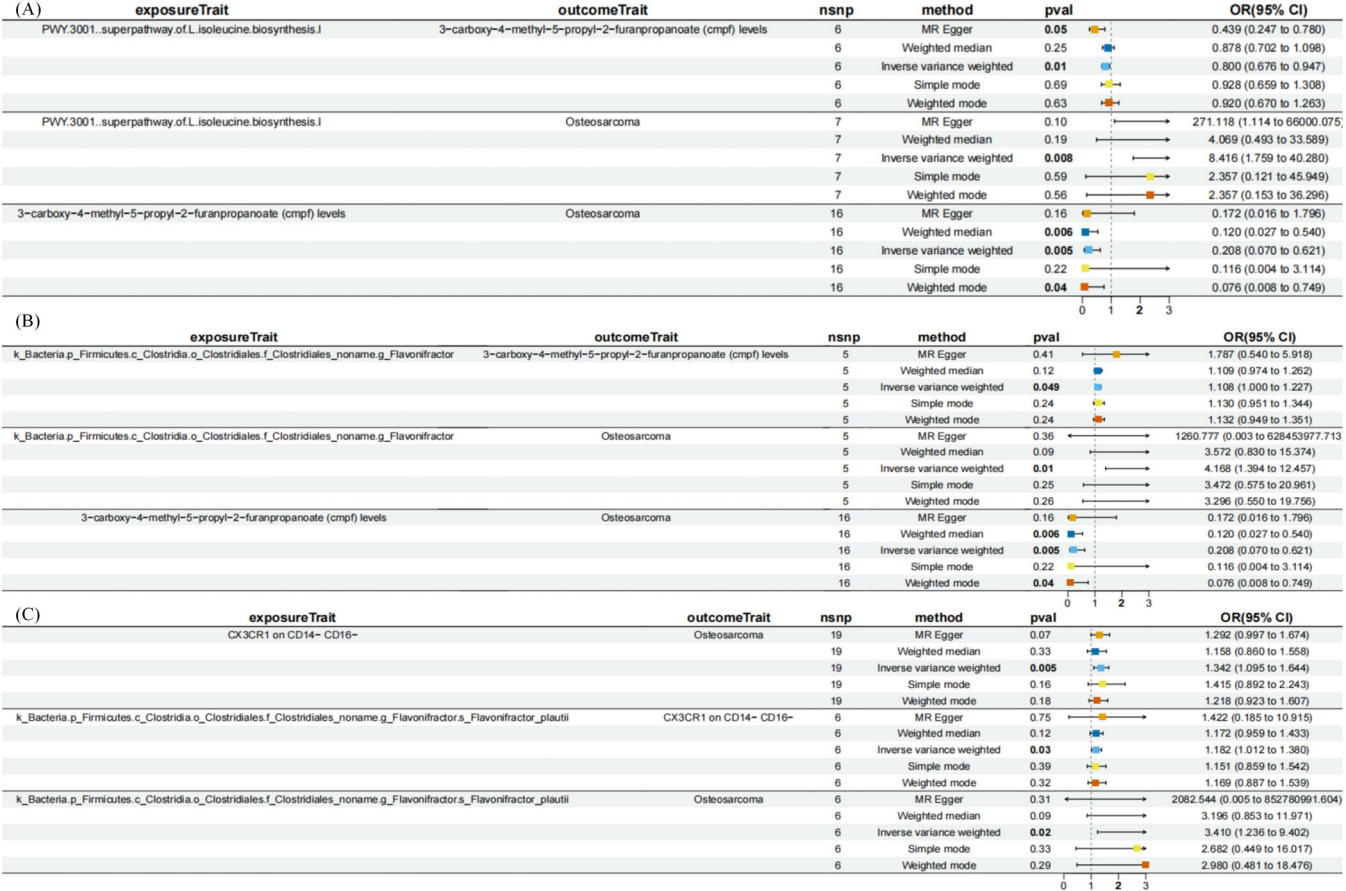
Forest plots summarizing MR results. (A) The causal effect of PWY‐3001 on OS and the metabolite CMPF on OS; (B) The causal effects of *Flavonifractor* on OS and the metabolite CMPF; (C) The causal effects of *Flavonifractor plautii* on OS and immune cells expressing CX3CR1 on CD14− CD16−, and the effects of these immune cells on OS risk.

CX3CR1+ monocytes mediate part of the relationship between *F. plautii* and OS (Figure 10C). Specifically, with every standard deviation increase in microbiome abundance, the proportion of CX3CR1+ cells increases by 0.18 standard deviations (*β* = 0.18, *p* = 0.01). This increase in CX3CR1+ cell proportion leads to a 34% higher risk of OS (OR = 1.34, *p* = 0.003), with a mediation effect accounting for 4.91% of the total effect (Figure 6C and Supporting Information S3: Table 5). This suggests that the microbiome may promote tumor progression through immune phenotype remodeling.

### 3.4 Sensitivity Analysis Results

In the sensitivity analysis, we conducted heterogeneity tests, horizontal pleiotropy analysis, and overall MR‐Steiger tests, with the results shown in Table 4 and Supporting Information S3: Tables 3 and 4. The heterogeneity tests revealed no significant heterogeneity among the instruments (SNPs) for any of the GM, metabolite, and immune cell features examined. We computed the intercept term in MR‐Egger regression and found no evidence of horizontal pleiotropy. Finally, to ensure the overall direction of causality in the MR analyses, we applied the MR‐Steiger model to validate the direction of causal estimates, confirming that the causal direction was correct in each MR analysis conducted.

## 4 Discussion

This study systematically investigates the potential causal relationships between GM, metabolites, and immune cells in the pathogenesis of OS using a two‐sample MR approach. This methodology enhances the reliability of causal inference and bolsters the robustness of the analysis. It also mitigates the risk of bias and facilitates the detection of multiple causal associations. Our findings reveal that 6 bacterial traits, 12 plasma metabolites, and 9 immune cell phenotypes are potentially causally linked to OS. Furthermore, mediation analysis supports the role of plasma metabolites and immune cells in mediating the impact of GM on the pathogenesis of OS. For the first time, this study uncovers a detailed mechanism by which GM influences the onset of OS via dual pathways involving metabolites and immune cells, providing new perspectives for the prevention and treatment of this malignancy.

The GM regulates a wide array of host functions, thereby influencing host health, phenotype, and disease. In recent years, dysbiosis has been closely associated with various complex diseases such as obesity, diabetes, cardiovascular diseases, gastrointestinal disorders, mental illnesses, and cancer [19, 20, 21, 22]. In this study, we first identified six GM (and associated pathways) that exhibited significant causal relationships with OS risk. Specifically, the l‐arginine degradation II pathway, dTDP‐l‐rhamnose biosynthesis pathway, and microbiota metabolic pathways involved in l‐isoleucine biosynthesis (with *E. coli* as the core species) were found to increase the incidence of OS. In contrast, the Bacteroidaceae family emerged as a protective factor against OS. Moreover, the abundance of flavonoid‐degrading genera, such as *Flavonifractor* and *F. plautii*, was positively correlated with OS risk. Genetic association analysis of metabolites with OS risk revealed 12 metabolites related to the disease, 4 of which were risk factors, while 8 were protective factors. These findings align with recent multi‐omics studies in OS patients, which demonstrated decreased gut microbial diversity and distinct enrichment of carbohydrate‐related metabolic pathways, supporting the critical role of the GM–metabolite axis in OS pathophysiology [23]. Notably, our identification of amino acid metabolic pathways as mediators is consistent with emerging evidence that GM‐derived metabolites, including 2‐ketoglutaric acid, can modulate systemic metabolic and immune signaling relevant to tumor progression [24].

Next, through mediation effect analysis, we further elucidated the mediating role of metabolites in the association between the GM and OS. We found that the l‐isoleucine biosynthesis microbiota metabolic pathway PWY‐3001 (with *E. coli* as the core species) mediated the relationship by 35% through *cmpf*, while *Flavonifractor* mediated it by 16.1% through CMPF, suggesting that the microbiota may indirectly drive tumorigenesis via metabolic reprogramming. Recent studies by Li and colleagues involved constructing an OS mouse model by subcutaneously inoculating 4‐week‐old female BALB/c nude mice with Saos‐2 OS cells, followed by 16S rRNA gene sequencing and untargeted metabolomics analysis. Their findings indicated that amino acid metabolism was dysregulated in OS, with genera related to bone metabolism, such as *Alloprevotella*, *Rikenellaceae_RC9_gut_group*, and *Muribaculum*, being correlated with amino acid metabolism, particularly histidine metabolism [25]. Additionally, Coelho and colleagues compared fecal samples from dogs with previously published microbiota gene catalogs based on similar sequencing methods, discovering that the gene phylum distribution in dogs more closely resembled that of humans compared to mice or pigs [26]. When clustering genes by species‐specific libraries, the dog gut gene catalog overlapped with the human gut microbiome by 23%, while the mouse gene catalog overlapped by only 4.9%. As in previous human studies, most components appeared to be influenced by environmental exposure, diet, and disease status. Notably, certain bacterial species have been found to exert similar cross‐species effects [10]. Hence, emphasizing the differences in GM across species is crucial for in vivo studies.

Furthermore, recent research has revealed significant differences in the oral microbiota between OS patients and healthy controls. Principal component analysis indicated that the oral microbial community differed markedly between these two groups. Fourteen genera, including *Rothia*, *Halomonas*, *Rhodococcus*, and *Granulicatella*, were significantly reduced, while *Alloprevotella*, *Prevotella*, *Selenomonas*, and *Campylobacter* were enriched in OS patients. Ultimately, the optimal four OTUs were identified to construct a microbial classifier using a random forest model via fivefold cross‐validation, achieving an area under the curve of 99.44% in the training group (30 OS patients vs. 60 healthy controls) and 87.33% in the test group (15 OS patients vs. 30 healthy controls), respectively. This suggests the potential efficacy of targeting oral microbiota biomarkers as a noninvasive diagnostic tool for OS [27]. These findings imply that the GM may regulate the progression of OS by influencing the synthesis and metabolism of metabolites.

Metabolites derived from the GM represent a crucial nexus linking the gut microbiome and the progression of cancer, primarily through the remodeling of the tumor microenvironment and the regulation of key signaling pathways in cancer cells and various immune cell types [28]. Therefore, investigating the regulatory relationship between the microbiota associated with OS and immune cells, as well as exploring the mediating role of immune cells in the GM–OS association, is pivotal for the future development of microbiome‐related immunotherapies for OS. In this study, we identified nine immune cell phenotypes associated with OS risk (four negatively correlated and five positively correlated). Further analysis revealed that *F. plautii* was positively correlated with the expression levels of the monocyte subpopulation CD14− CD16− surface marker CX3CR1. Additionally, the mediating effect of immune cells in the GM–OS relationship was confirmed. Specifically, an increase in the proportion of CX3CR1+ cells was associated with a 34% higher OS risk (OR = 1.34, *p* = 0.003), with a mediation effect accounting for 4.91%, suggesting that the microbiota may promote tumor progression through immune phenotype remodeling. This discovery provides suggestive evidence that GM dysbiosis may influence peripheral immune cell phenotypes, contributing to OS pathogenesis. However, clinical translation requires quantifying its absolute risk contribution—specifically, determining whether GM‐driven immune alterations account for a substantive proportion of population‐level OS risk (e.g., via population attributable fraction analysis).

Furthermore, Tian and colleagues evaluated changes in the GM during OS growth and postchemotherapy with cisplatin (CDDP) or doxorubicin (DOX) in an OS mouse model, indicating correlations between dysbiosis of the GM and OS growth as well as chemotherapy treatment with CDDP or DOX, potentially leading to novel adjuvant therapies [8]. Recent studies have shown a causal relationship between the GM and non‐small cell lung cancer (NSCLC). Notably, the mediation MR illustrated the causal role of *Peptococcus* on NSCLC (Total Effect IVW: OR = 0.790, 95% CI [0.657, 0.950], *p* = 0.012), with a significant mediation by CD45 on HLA DR CD4 in the TBNK panel (−0.034, 95% CI [−0.070, −0.005]; *p* = 0.037), accounting for 14.4% of the total effect [29]. Li and colleagues investigated the relationship between GM and obesity, clarifying the mediating role of peripheral cells and inflammatory cytokines [30]. Recent results also showed that *Megamonas funiformis* was associated with an increased risk of breast cancer, with 11.20% of this effect mediated by CD38 on IgD+ CD24−. Likewise, HLA DR on CD33br HLA DR+ CD14− mediated the causal relationship between *Prevotellamassilia* and breast cancer, accounting for a mediation ratio of 7.89% [31]. These studies provide new perspectives for understanding the complex interactions between the GM and host health and offer potential targets for the development of future disease prevention and treatment strategies.

The results of this study have significant clinical and public health implications. First, by identifying GM associated with OS risk, we can offer new targets for early screening and prevention of OS. Second, by intervening in the composition and function of the GM, we can explore new strategies for OS treatment. For instance, through dietary adjustments, the use of probiotics, or antibiotics, we may alter the composition of the GM, thereby influencing metabolites and immune cell states, ultimately reducing the risk of OS. However, this study also has some limitations. First, it relies on summary data from large‐scale GWAS and lacks detailed individual‐level information, which may affect the accuracy and reliability of the results. Second, the study primarily focuses on the causal relationships between GM, metabolites, and immune cells but does not comprehensively consider other potential environmental and genetic factors influencing OS pathogenesis. Therefore, further experimental studies are needed to verify and extend our findings. Finally, some wide 95% CIs in this study indicate potential result uncertainty. For instance, the DTDPRHAMSYN.PWY showed an elevated risk estimate (OR = 7.23, 95% CI: 1.50–34.77), but its broad CI reflects the limited precision of current genetic instruments. Larger‐sample GWAS are required in future investigations to refine IV strength.

Although MR design reduces confounding bias, we recommend future studies to: validate the microbiota–metabolite–osteosarcoma pathway in independent cohorts; analyze the spatial distribution of CX3CR1+ monocytes in the tumor microenvironment through single‐cell sequencing to verify their mediating role. The microbiota (e.g., *F. plautii*) and metabolites (e.g., CMPF) identified in this study can serve as potential intervention targets. For instance, developing probiotic preparations or dietary supplements to regulate CMPF levels may provide new strategies for osteosarcoma prevention.

In conclusion, this study systematically explored the causal mechanisms by which the GM influences OS pathogenesis through metabolic and immune pathways using MR, providing new perspectives and potential targets for the prevention and treatment of OS. Future research should further validate these findings and explore their clinical applicability.

## 5 Conclusion

This study is the first to uncover the causal mechanisms by which the GM influences OS through metabolic and immune pathways using MR. The PWY.3001‐associated microbiota increases OS risk by inhibiting protective metabolites like cmpf (mediating effect of 35%), while *F. plautii* promotes tumor progression via CX3CR1 activation on CD14− CD16− protumor monocytes (mediating effect of 4.91%). These findings provide potential targets for precision prevention and treatment of OS by modulating the gut microbiome.

## Author Contributions

All authors made substantial contributions to the manuscript, including conception and design, preparing the manuscript, and approving the final version. Supervision and writing (review and editing): Guanjun Chen and Jincheng Huang; conceptualization and writing (original draft): Guanjun Chen and Zhenyu Song; revision: Xinyu Wang and Chunjiang Zhu; funding acquisition: Zhenyu Song and Guanjun Chen; investigation, visualization, and validation: Jincheng Huang and Zhenyu Song; formal analysis and methodology: Imtiaz Abdullah and Xinyu Wang; resources and writing (review and editing): Imtiaz Abdullah and Guanjun Chen. All authors have read and approved the final version of the manuscript.

## Ethics Statement

The authors have nothing to report.

## Conflicts of Interest

The authors declare no conflicts of interest.

## Transparency Statement

The lead authors Chunjiang Zhu and Jincheng Huang affirm that this manuscript is an honest, accurate, and transparent account of the study being reported; that no important aspects of the study have been omitted; and that any discrepancies from the study as planned (and, if relevant, registered) have been explained.

## Supporting information


**Supporting Figure 1:** Forest plots of Mendelian randomization causal estimates.

**Supporting Figure 2:** Scatter plots of SNP effect sizes for Mendelian randomization analyses visualizing genetic instrument associations between exposures and outcomes.

**Supporting Table 1:** SNP information included in MR studies. **Supporting Table 2:** MR results. **Supporting Table 3:** Cochrane *Q*‐test results. **Supporting Table 4:** Results of pleiotropic tests. **Supporting Table 5:** Mediation analysis results. **Supporting Table 6:** Results of the MR‐PRESSO test.

## Data Availability

The data that support the findings of this study are available from the corresponding author upon reasonable request. Guanjun Chen had full access to all of the data in this study and took complete responsibility for the integrity of the data and the accuracy of the data analysis.

## References

[1] C. Chen , L. Xie , T. Ren , Y. Huang , J. Xu , and W. Guo , “Immunotherapy for Osteosarcoma: Fundamental Mechanism, Rationale, and Recent Breakthroughs,” Cancer Letters 500 (2021): 1–10. 33359211 10.1016/j.canlet.2020.12.024

[2] B. Zhang , Y. Zhang , R. Li , J. Li , X. Lu , and Y. Zhang , “The Efficacy and Safety Comparison of First‐Line Chemotherapeutic Agents (High‐Dose Methotrexate, Doxorubicin, Cisplatin, and Ifosfamide) for Osteosarcoma: A Network Meta‐Analysis,” Journal of Orthopaedic Surgery and Research 15, no. 1 (2020): 51. 32054494 10.1186/s13018-020-1576-0 PMC7020590

[3] J. Ritter and S. S. Bielack , “Osteosarcoma,” Annals of Oncology 21, no. Suppl 7 (2010): vii320–vii325. 20943636 10.1093/annonc/mdq276

[4] V. Tremaroli and F. Bäckhed , “Functional Interactions Between the Gut Microbiota and Host Metabolism,” Nature 489, no. 7415 (2012): 242–249. 22972297 10.1038/nature11552

[5] A. Ben Ya'Acov , Y. Lichtenstein , L. Zolotarov , et al., “The Gut Microbiome as a Target for Regulatory T Cell‐Based Immunotherapy: Induction of Regulatory Lymphocytes by Oral Administration of Anti‐LPS Enriched Colostrum Alleviates Immune Mediated Colitis,” BMC Gastroenterology 15 (2015): 154. 26518263 10.1186/s12876-015-0388-x PMC4628342

[6] H. C. Wastyk , G. K. Fragiadakis , D. Perelman , et al., “Gut‐Microbiota‐Targeted Diets Modulate Human Immune Status,” Cell 184, no. 16 (2021): 4137–4153.e14. 34256014 10.1016/j.cell.2021.06.019 PMC9020749

[7] N. Shi , N. Li , X. Duan , et al., “Interaction Between the Gut Microbiome and Mucosal Immune System,” Military Medical Research 4 (2017): 14. 28465831 10.1186/s40779-017-0122-9 PMC5408367

[8] Z. Tian , X. Qiao , Z. Wang , et al., “Cisplatin and Doxorubicin Chemotherapy Alters Gut Microbiota in a Murine Osteosarcoma Model,” Aging 16, no. 2 (2024): 1336–1351. 38231481 10.18632/aging.205428 PMC10866425

[9] D. Le , M. M. Chambers , K. Mercado , and C. J. Gutowski , “Characterization of the Gut Microbiome in an Osteosarcoma Mouse Model,” Journal of Orthopaedic Research 41, no. 12 (2023): 2730–2739. 37246455 10.1002/jor.25635

[10] K. T. Kleber , K. R. Iranpur , L. M. Perry , et al., “Using the Canine Microbiome to Bridge Translation of Cancer Immunotherapy From Pre‐Clinical Murine Models to Human Clinical Trials,” Frontiers in Immunology 13 (2022): 983344. 36032113 10.3389/fimmu.2022.983344 PMC9412231

[11] G. Davey Smith and G. Hemani , “Mendelian Randomization: Genetic Anchors for Causal Inference in Epidemiological Studies,” Human Molecular Genetics 23, no. R1 (2014): R89–R98. 25064373 10.1093/hmg/ddu328 PMC4170722

[12] S. Burgess , R. A. Scott , N. J. Timpson , G. Davey Smith , and S. G. Thompson , “Using Published Data in Mendelian Randomization: A Blueprint for Efficient Identification of Causal Risk Factors,” European Journal of Epidemiology 30, no. 7 (2015): 543–552. 25773750 10.1007/s10654-015-0011-z PMC4516908

[13] E. A. Lopera‐Maya , A. Kurilshikov , A. Van Der Graaf , et al., “Effect of Host Genetics on the Gut Microbiome in 7,738 Participants of the Dutch Microbiome Project,” Nature Genetics 54, no. 2 (2022): 143–151. 35115690 10.1038/s41588-021-00992-y

[14] Y. Chen , T. Lu , U. Pettersson‐Kymmer , et al., “Genomic Atlas of the Plasma Metabolome Prioritizes Metabolites Implicated in Human Diseases,” Nature Genetics 55, no. 1 (2023): 44–53. 36635386 10.1038/s41588-022-01270-1 PMC7614162

[15] V. Orrù , M. Steri , C. Sidore , et al., “Complex Genetic Signatures in Immune Cells Underlie Autoimmunity and Inform Therapy,” Nature Genetics 52, no. 10 (2020): 1036–1045. 32929287 10.1038/s41588-020-0684-4 PMC8517961

[16] N. M. Davies , M. V. Holmes , and G. Davey Smith , “Reading Mendelian Randomisation Studies: A Guide, Glossary, and Checklist for Clinicians,” BMJ 362 (2018): k601. 30002074 10.1136/bmj.k601 PMC6041728

[17] V. W. Skrivankova , R. C. Richmond , B. A. R. Woolf , et al., “Strengthening the Reporting of Observational Studies in Epidemiology Using Mendelian Randomization: The STROBE‐MR Statement,” Journal of the American Medical Association 326, no. 16 (2021): 1614–1621. 34698778 10.1001/jama.2021.18236

[18] T. A. Lang and D. G. Altman , “Basic Statistical Reporting for Articles Published in Biomedical Journals: The ‘Statistical Analyses and Methods in the Published Literature’ or the SAMPL Guidelines,” International Journal of Nursing Studies 52, no. 1 (2015): 5–9. 25441757 10.1016/j.ijnurstu.2014.09.006

[19] J. Cai , L. Sun , and F. J. Gonzalez , “Gut Microbiota‐Derived Bile Acids in Intestinal Immunity, Inflammation, and Tumorigenesis,” Cell Host & Microbe 30, no. 3 (2022): 289–300. 35271802 10.1016/j.chom.2022.02.004 PMC8923532

[20] Q. Xu , J. J. Ni , B. X. Han , et al., “Causal Relationship Between Gut Microbiota and Autoimmune Diseases: A Two‐Sample Mendelian Randomization Study,” Frontiers in Immunology 12 (2021): 746998. 35140703 10.3389/fimmu.2021.746998 PMC8819003

[21] T. Liu , Y. Cao , N. Liang , X. Ma , J. Fang , and X. Zhang , “Investigating the Causal Association Between Gut Microbiota and Type 2 Diabetes: A Meta‐Analysis and Mendelian Randomization,” Frontiers in Public Health 12 (2024): 1342313. 38962766 10.3389/fpubh.2024.1342313 PMC11220316

[22] C. Chen , D. Zhang , D. Wu , F. Chen , Z. Li , and Y. Hu , “Gut Microbiome, and Immune Cells Mediated Effect on Depression: A Two‐Step, Two‐Sample Mendelian Randomization Analysis,” Experimental Gerontology 195 (2024): 112530. 39059516 10.1016/j.exger.2024.112530

[23] C. Li , Y. Chen , W. Yao , et al., “Gut Microbiome and Serum Metabolome Alterations in Osteosarcoma Patients,” Frontiers in Microbiology 16 (2025): 1616603. 40746321 10.3389/fmicb.2025.1616603 PMC12310664

[24] X. Sun , W. Li , G. Chen , G. Hu , and J. Jia , “ *Faecalibacterium duncaniae* Mitigates Intestinal Barrier Damage in Mice Induced by High‐Altitude Exposure by Increasing Levels of 2‐Ketoglutaric Acid,” Nutrients 17, no. 8 (2025): 1380. 40284246 10.3390/nu17081380 PMC12030221

[25] Y. Li , X. Qiao , Y. Feng , et al., “Characterization of the Gut Microbiota and Fecal Metabolome in the Osteosarcoma Mouse Model,” Aging 16, no. 13 (2024): 10841–10859. 38967635 10.18632/aging.205951 PMC11272122

[26] S. Ciaravolo , L. M. Martínez‐López , R. J. N. Allcock , A. P. Woodward , and C. Mansfield , “Longitudinal Survey of Fecal Microbiota in Healthy Dogs Administered a Commercial Probiotic,” Frontiers in Veterinary Science 8 (2021): 664318. 34235200 10.3389/fvets.2021.664318 PMC8255976

[27] Y. Chen , C. Li , X. Wang , C. L. Zhang , Z. G. Ren , and Z. Q. Wang , “Oral Microbiota Distinguishes Patients With Osteosarcoma From Healthy Controls,” Frontiers in Cellular and Infection Microbiology 14 (2024): 1383878. 39055977 10.3389/fcimb.2024.1383878 PMC11269967

[28] Q. Yang , B. Wang , Q. Zheng , et al., “A Review of Gut Microbiota‐Derived Metabolites in Tumor Progression and Cancer Therapy,” Advanced Science 10, no. 15 (2023): e2207366. 36951547 10.1002/advs.202207366 PMC10214247

[29] J. Chen , X. Yu , X. Wu , K. Chai , and S. Wang , “Causal Relationships Between Gut Microbiota, Immune Cell, and Non‐Small Cell Lung Cancer: A Two‐Step, Two‐Sample Mendelian Randomization Study,” Journal of Cancer 15, no. 7 (2024): 1890–1897. 38434967 10.7150/jca.92699 PMC10905411

[30] Y. Li , X. Wang , Z. Zhang , L. Shi , L. Cheng , and X. Zhang , “Effect of the Gut Microbiome, Plasma Metabolome, Peripheral Cells, and Inflammatory Cytokines on Obesity: A Bidirectional Two‐Sample Mendelian Randomization Study and Mediation Analysis,” Frontiers in Immunology 15 (2024): 1348347. 38558794 10.3389/fimmu.2024.1348347 PMC10981273

[31] R. Lv , D. Wang , T. Wang , et al., “Causality Between Gut Microbiota, Immune Cells, and Breast Cancer: Mendelian Randomization Analysis,” Medicine 103, no. 49 (2024): e40815. 39654239 10.1097/MD.0000000000040815 PMC11630993

