# Supplementary material for: Metabolites and Immune Cells Mediated the Causal Relationship Between the Gut Microbiota and Osteosarcoma: A Mendelian Randomization Study
Source: Health Sci Rep. 2025 Oct 30;8(11):e71430. doi: 10.1002/hsr2.71430 (PMC12575158; doi:10.1002/hsr2.71430)
Supplement: Supplementary file 1 — Supporting Figure 1: Forest plots of Mendelian randomization causal estimates. [file HSR2-8-e71430-s001.pdf]

Figure Title

Supplementary Figure 1: Forest Plots of Mendelian Randomization Causal Estimates

Figure Legend

1. Overview

Forest plots summarizing causal effect estimates from Mendelian Randomization (MR) analyses. Each subplot represents a unique exposure-outcome pair, displaying effect sizes of instrumental variables (SNPs) and summary estimates.

2. Key Components

(1) Point estimates & error bars:

Dots indicate SNP-specific effect sizes (e.g., rs1325380, rs7209568).

Horizontal lines show 95% confidence intervals.

(2) Summary methods:

Inverse variance weighted (IVW): Primary fixed/random-effects estimator.

MR Egger: Sensitivity analysis correcting for pleiotropy.

(3) Axes:

X-axis: Effect size (β). Negative values = protective effect; positive values = risk effect.

Y-axis: Individual SNPs or summary estimates.

3. Exposure-Outcome Pairs

Labels follow the format:

"MR effect size for '[EXPOSURE]' on '[OUTCOME]'"

Where:

(1) Exposures:

GCST...: GWAS Catalog study IDs (e.g., GCST90027452).

Microbial taxa (e.g., k__Bacteria...g__Flavonifractor).

Metabolic pathways (e.g., PWY-3001 = L-isoleucine biosynthesis).

Metabolites (e.g., cmpf, e12NEt).

(2) Outcomes: Primarily Osteosarcoma, with additional targets (e.g., AST.PWY..L.arginine.degradation).

4. Critical Abbreviations

SNP: Single nucleotide polymorphism

IVW: Inverse variance weighted

GCST: GWAS Catalog Study ID

PWY: Metabolic pathway identifier

k/p/c/o/f/g/s: Microbial taxonomy ranks (kingdom/phylum/class/order/family/genus/species)

5. Interpretation Guidance

Effect direction: Left of zero (↓ risk), right of zero (↑ risk).

Method comparison: Discordance between IVW and MR Egger suggests pleiotropy.

Scale variability: X-axis ranges differ across subplots (e.g., −20 to 20 vs. −0.10 to 0.10) to optimize visualization of effect sizes.

Note: All analyses leverage genetic variants as instrumental variables to infer causal relationships, with summary statistics derived from genome-wide association studies (GWAS).

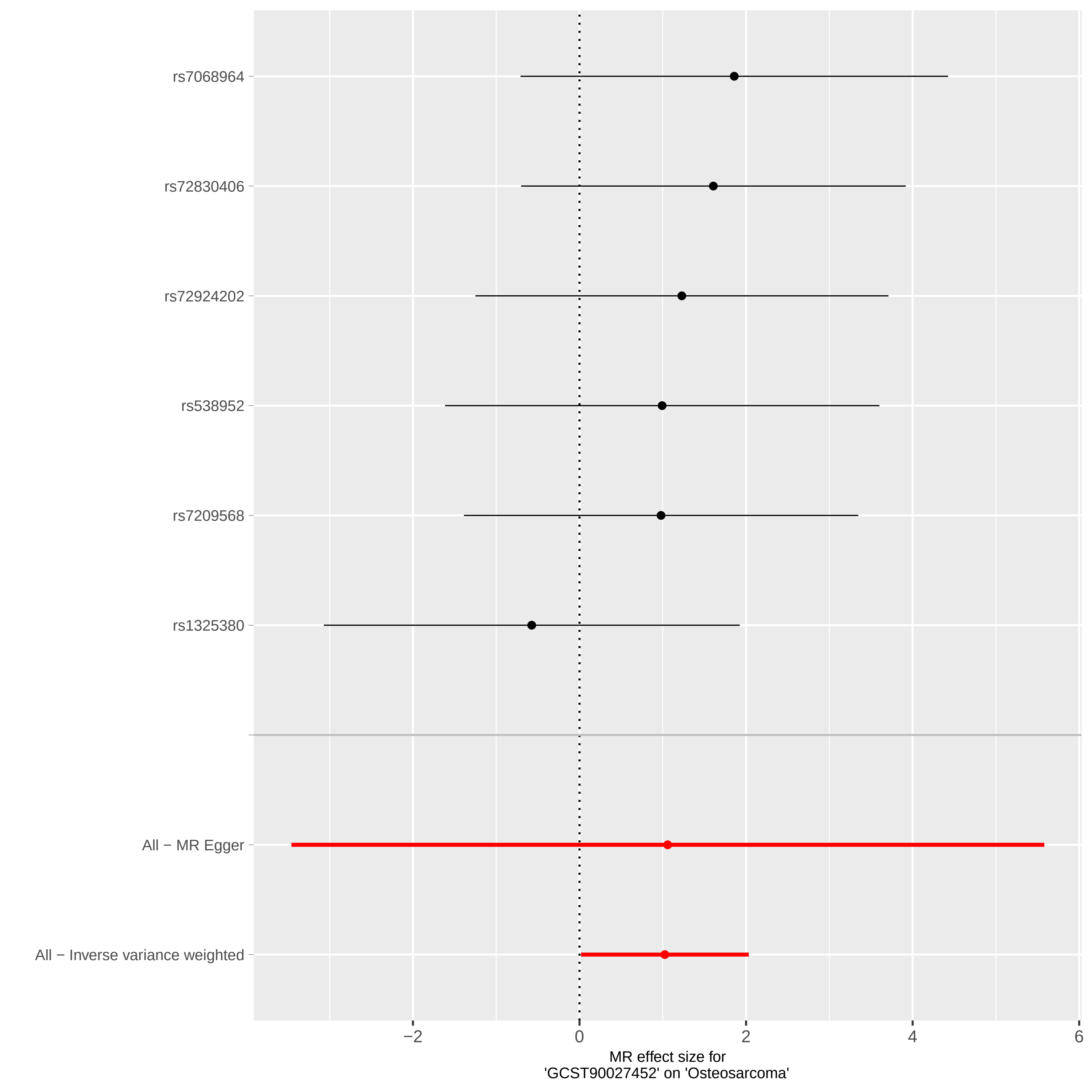

rs7068964
rs72830406
rs72924202
rs538952
rs7209568
rs1325380
All − MR Egger
All − Inverse variance weighted
−2
0
2
4
6
MR effect size for
'GCST90027452' on 'Osteosarcoma'

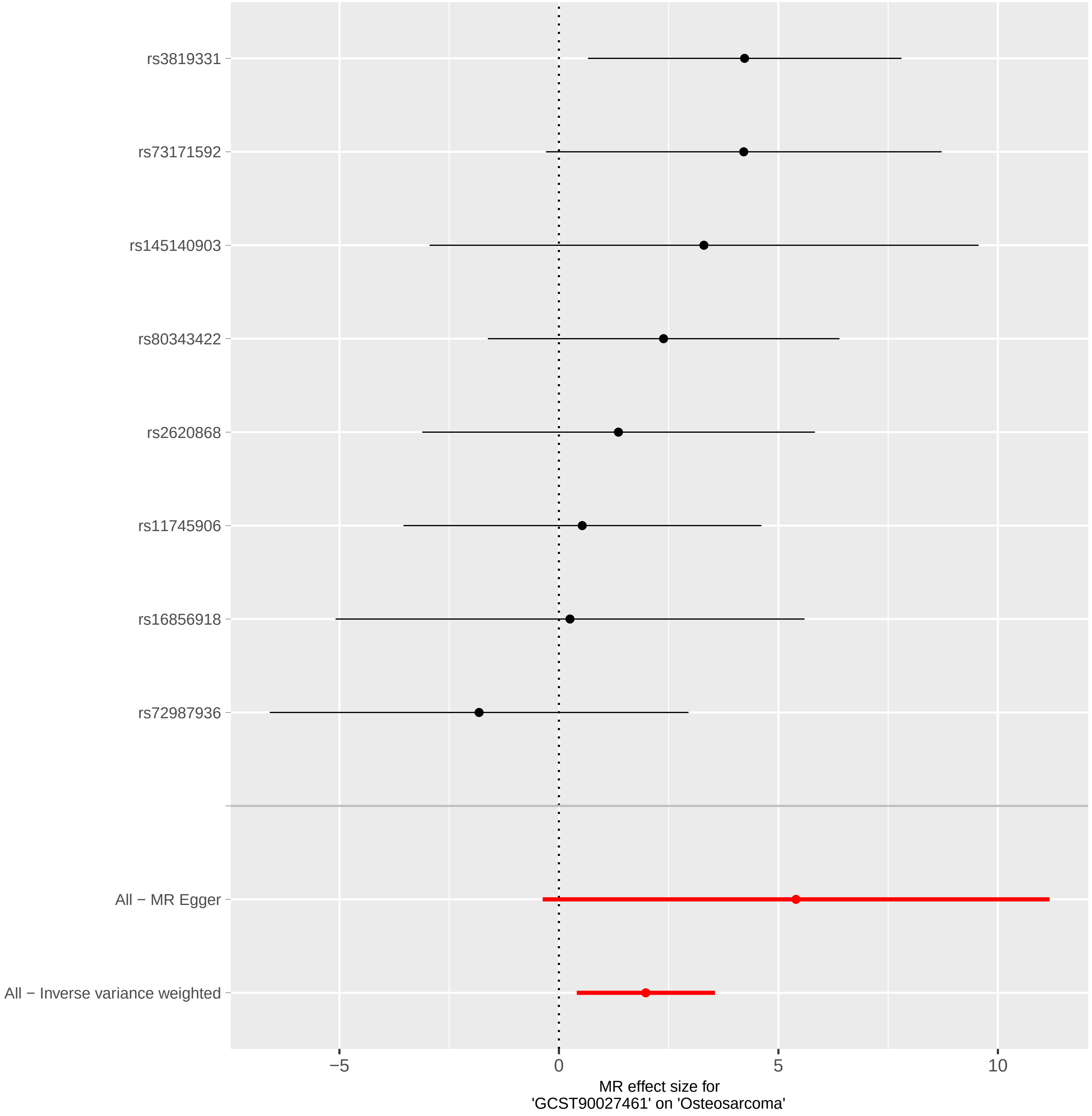
rs3819331
rs73171592
rs145140903
rs80343422
rs2620868
rs11745906
rs16856918
rs72987936
All − MR Egger
All − Inverse variance weighted
−5
0
5
10
MR effect size for
'GCST90027461' on 'Osteosarcoma'

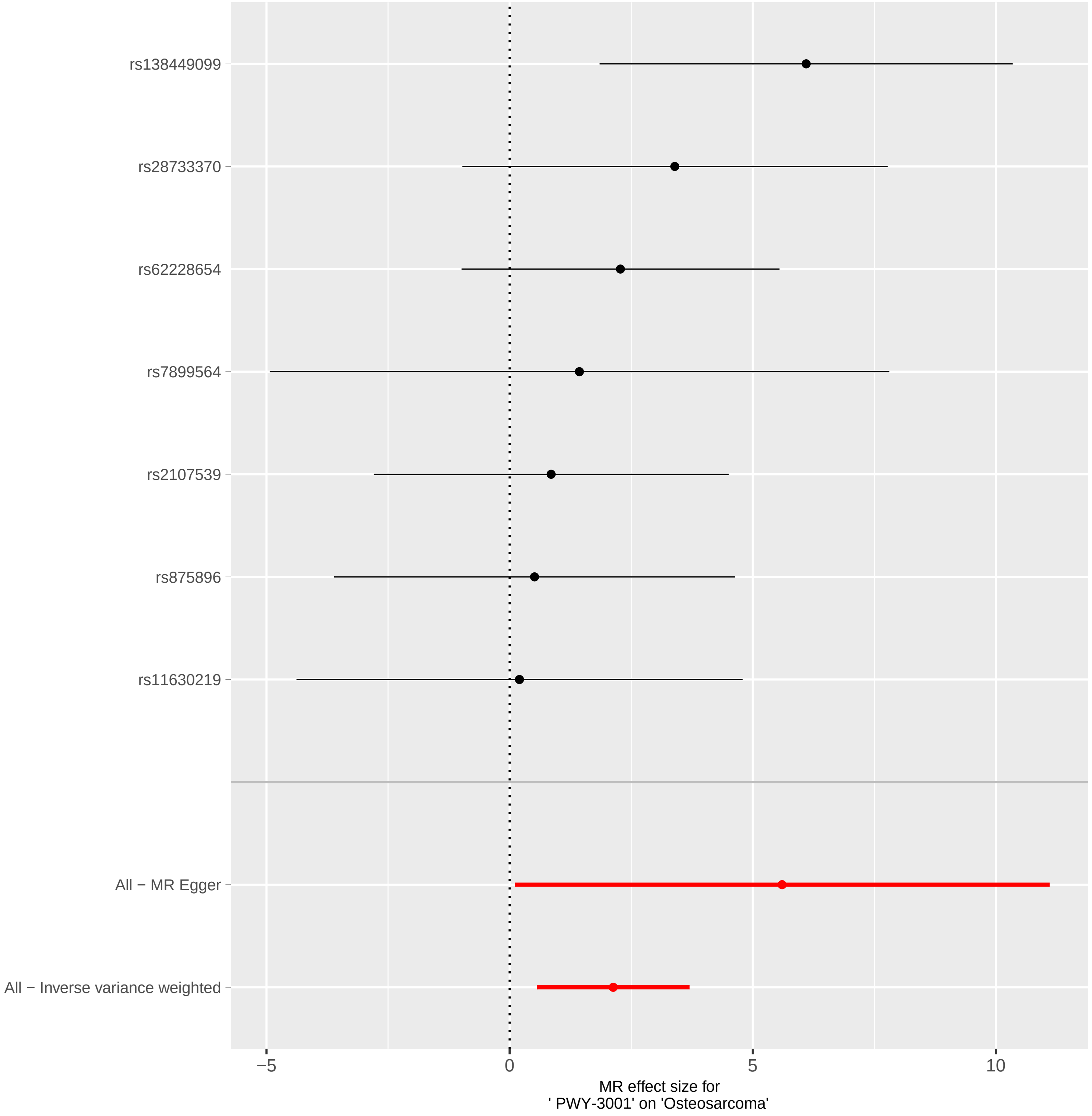

rs138449099
rs28733370
rs62228654
rs7899564
rs2107539
rs875896
rs11630219
All − MR Egger
All − Inverse variance weighted
−5
0
5
10
MR effect size for
' PWY-3001' on 'Osteosarcoma'

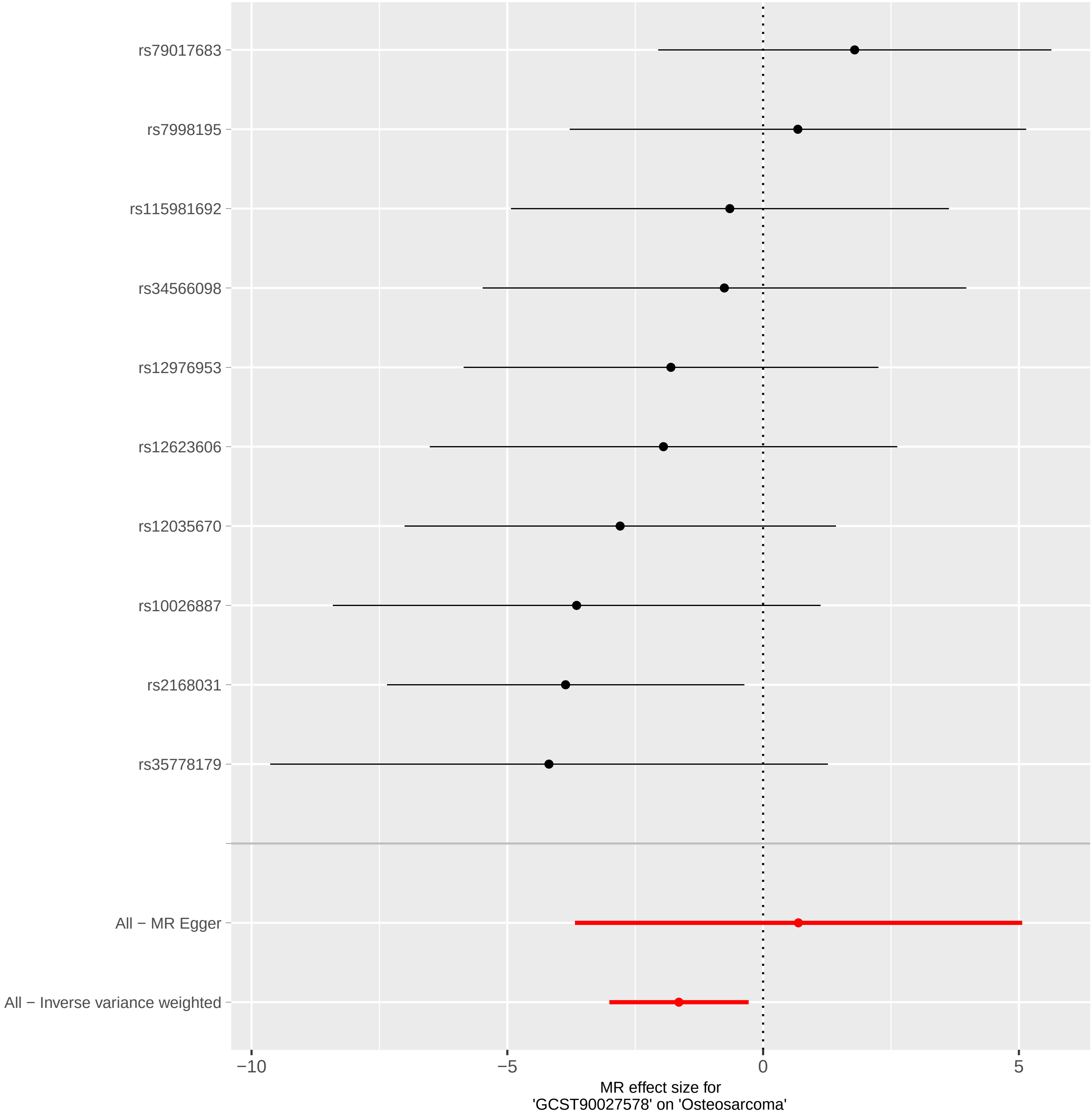

rs79017683
rs7998195
rs115981692
rs34566098
rs12976953
rs12623606
rs12035670
rs10026887
rs2168031
rs35778179
All − MR Egger
All − Inverse variance weighted
−10
−5
0
5
MR effect size for
'GCST90027578' on 'Osteosarcoma'

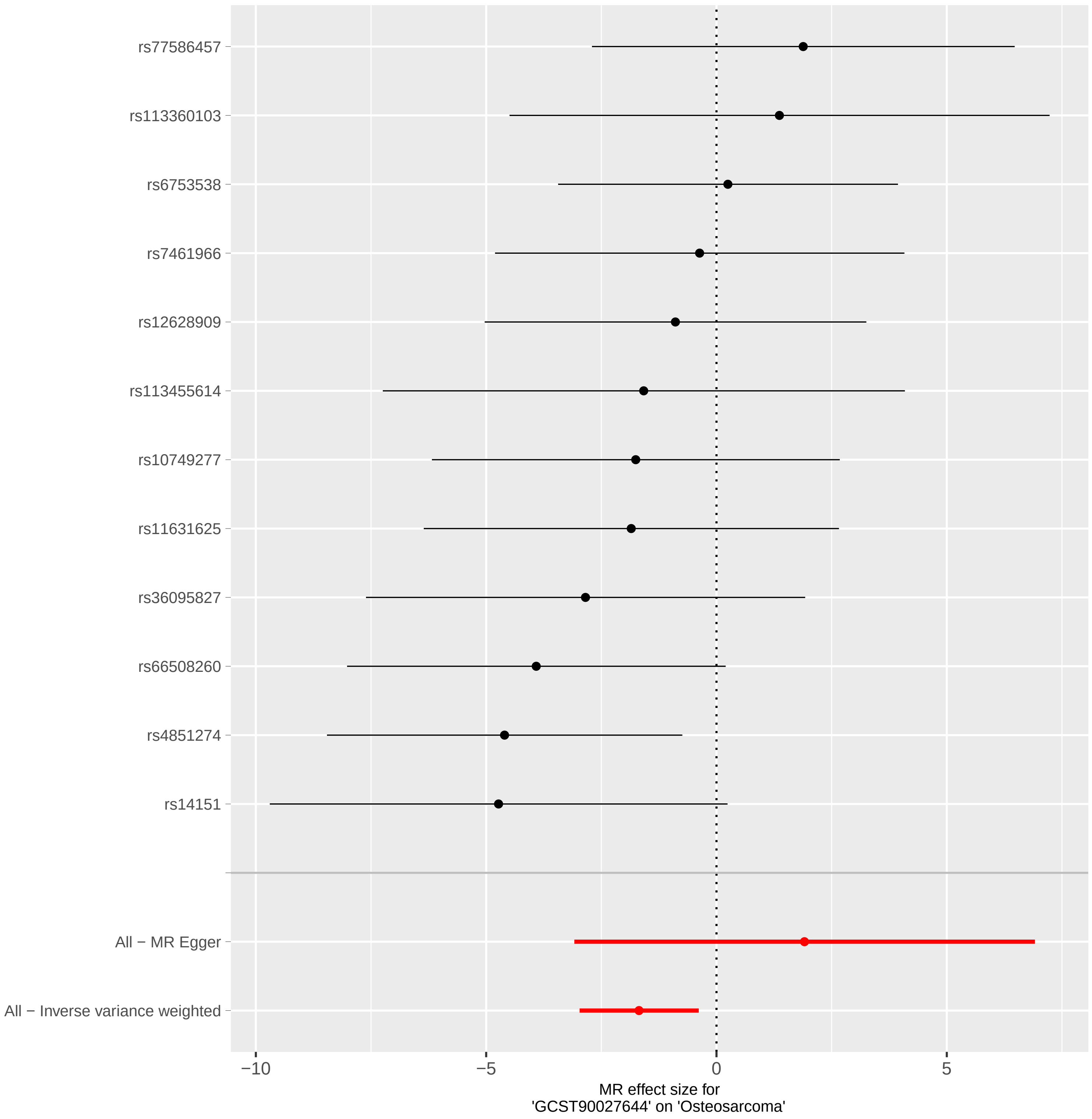

rs77586457
rs113360103
rs6753538
rs7461966
rs12628909
rs113455614
rs10749277
rs11631625
rs36095827
rs66508260
rs4851274
rs14151
All − MR Egger
All − Inverse variance weighted
−10
−5
0
5
MR effect size for
'GCST90027644' on 'Osteosarcoma'

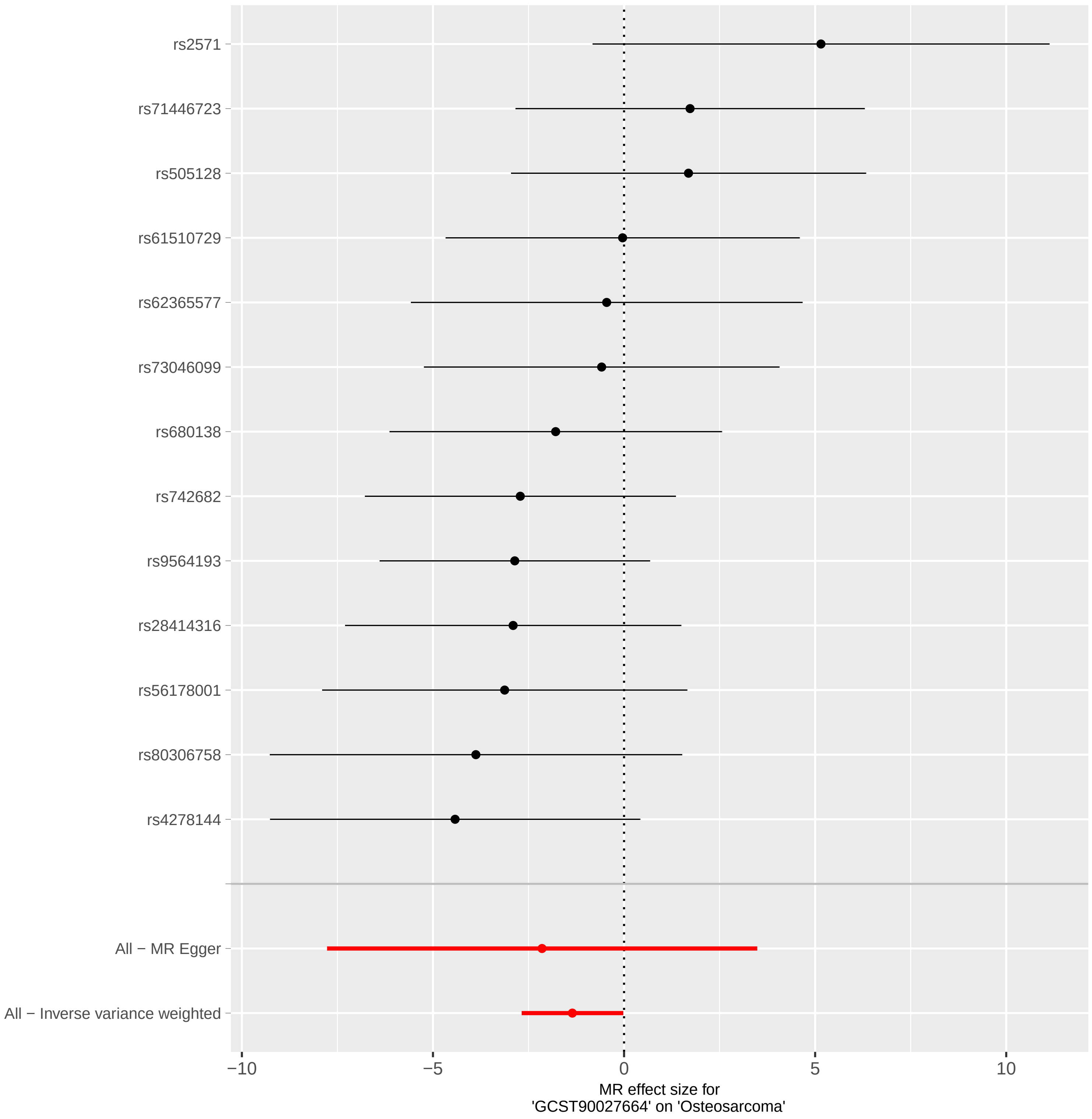
rs2571
rs71446723
rs505128
rs61510729
rs62365577
rs73046099
rs680138
rs742682
rs9564193
rs28414316
rs56178001
rs80306758
rs4278144
All − MR Egger
All − Inverse variance weighted
−10
−5
0
5
10
MR effect size for
'GCST90027664' on 'Osteosarcoma'

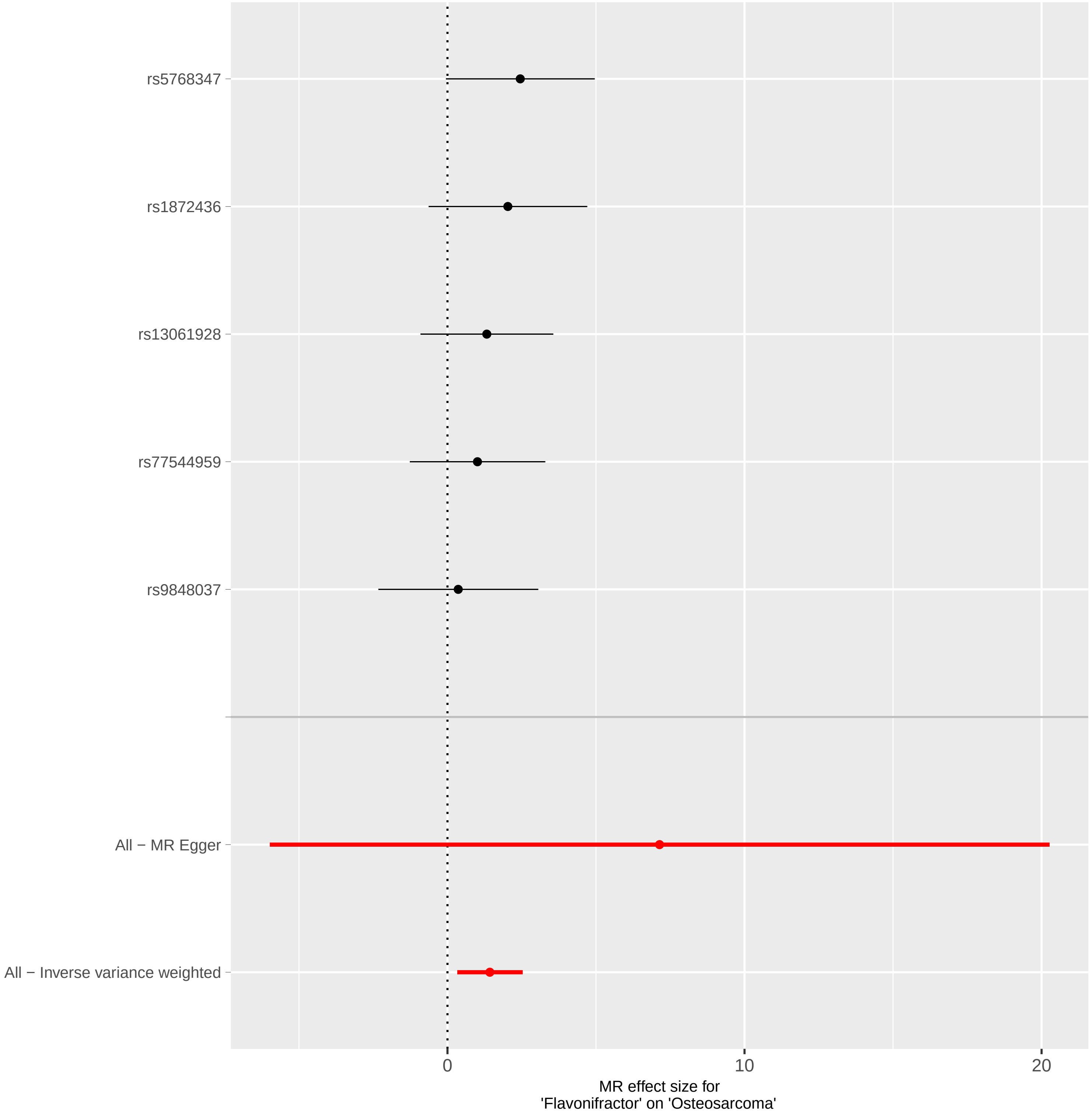

rs5768347
rs1872436
rs13061928
rs77544959
rs9848037
All − MR Egger
All − Inverse variance weighted
0
10
20
MR effect size for
'Flavonifractor' on 'Osteosarcoma'

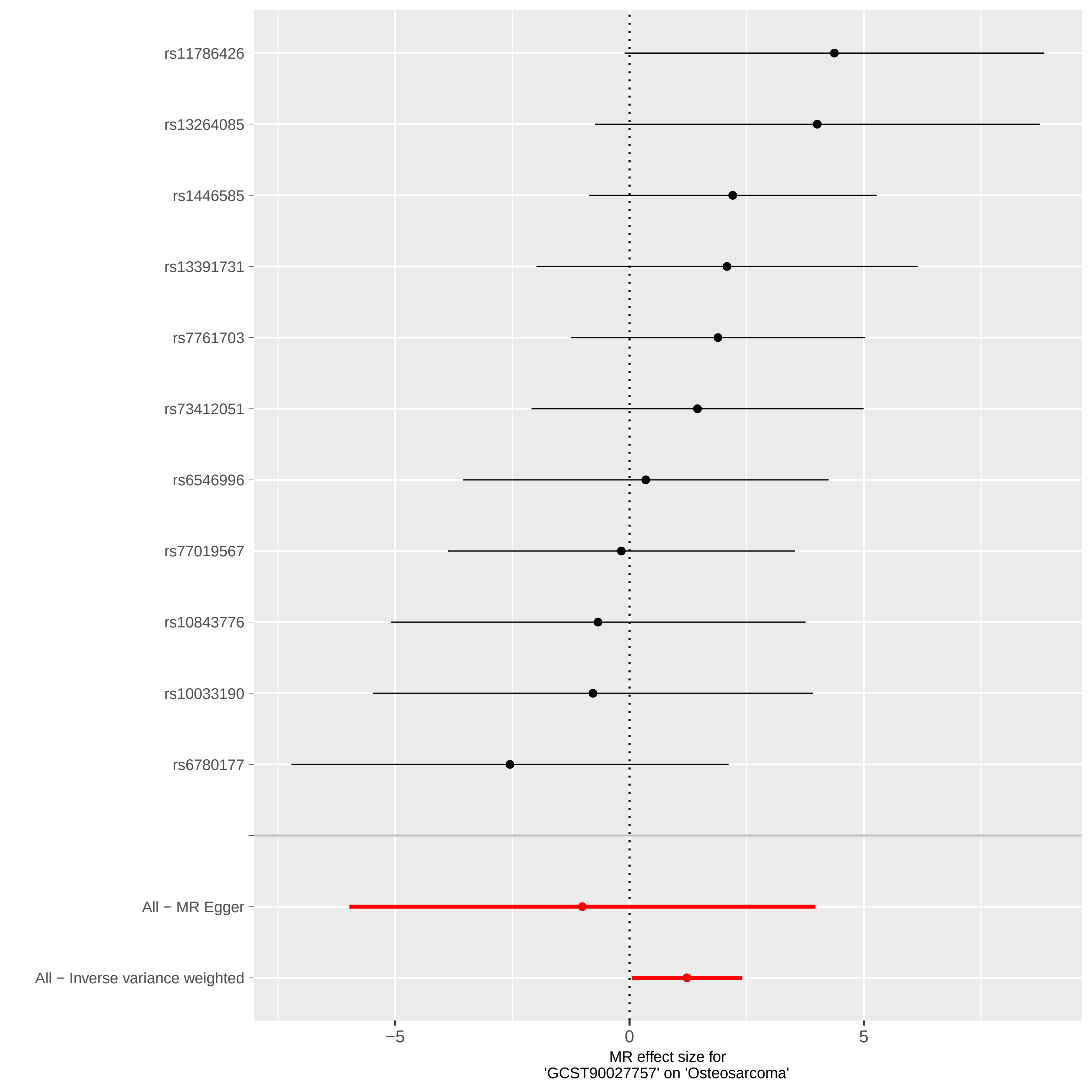
rs11786426
rs13264085
rs1446585
rs13391731
rs7761703
rs73412051
rs6546996
rs77019567
rs10843776
rs10033190
rs6780177
All − MR Egger
All − Inverse variance weighted
−5
0
5
MR effect size for
'GCST90027757' on 'Osteosarcoma'

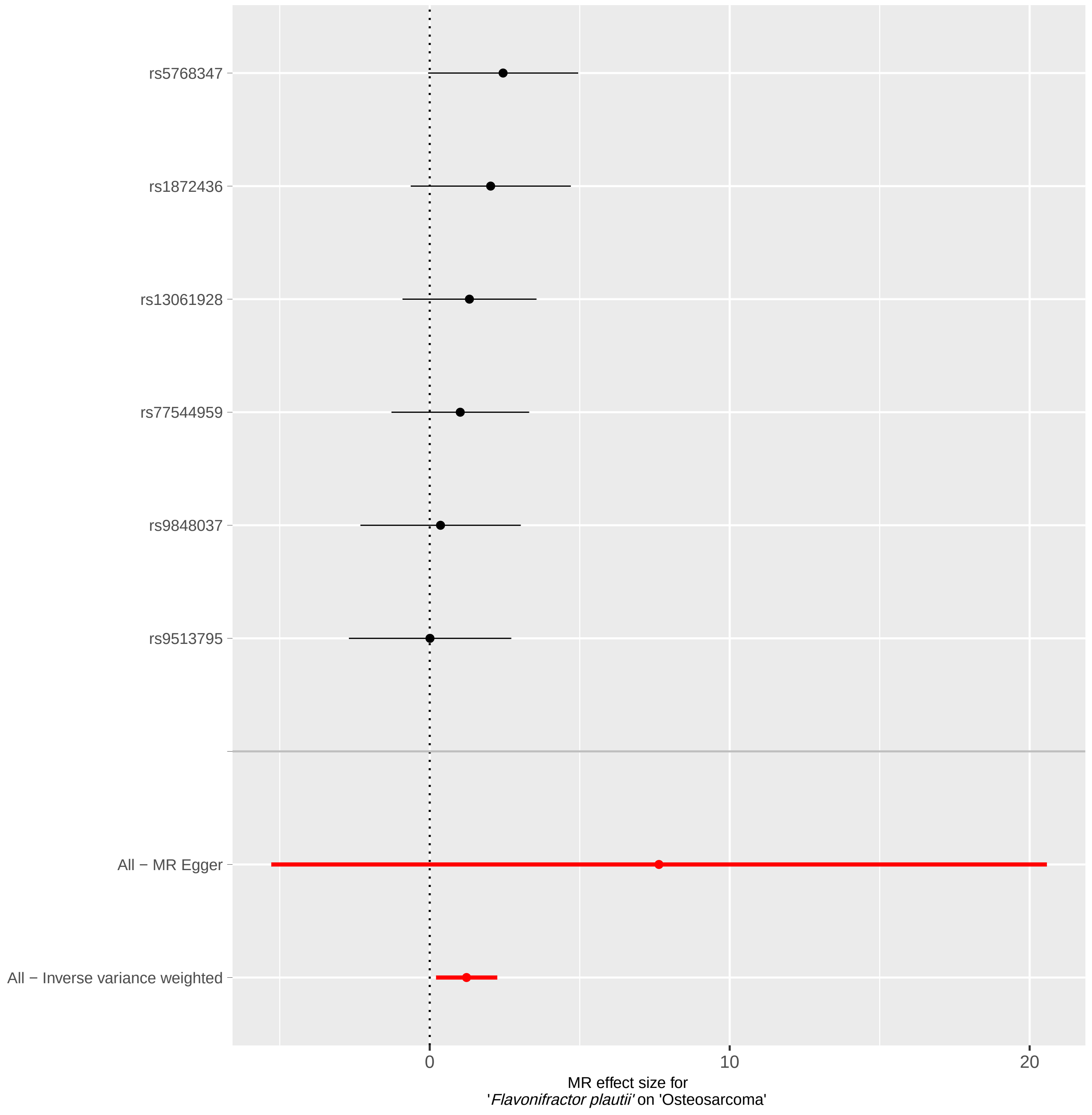

rs5768347
rs1872436
rs13061928
rs77544959
rs9848037
rs9513795
All − MR Egger
All − Inverse variance weighted
0
10
20
MR effect size for
'*Flavonifractor plautii*' on 'Osteosarcoma'

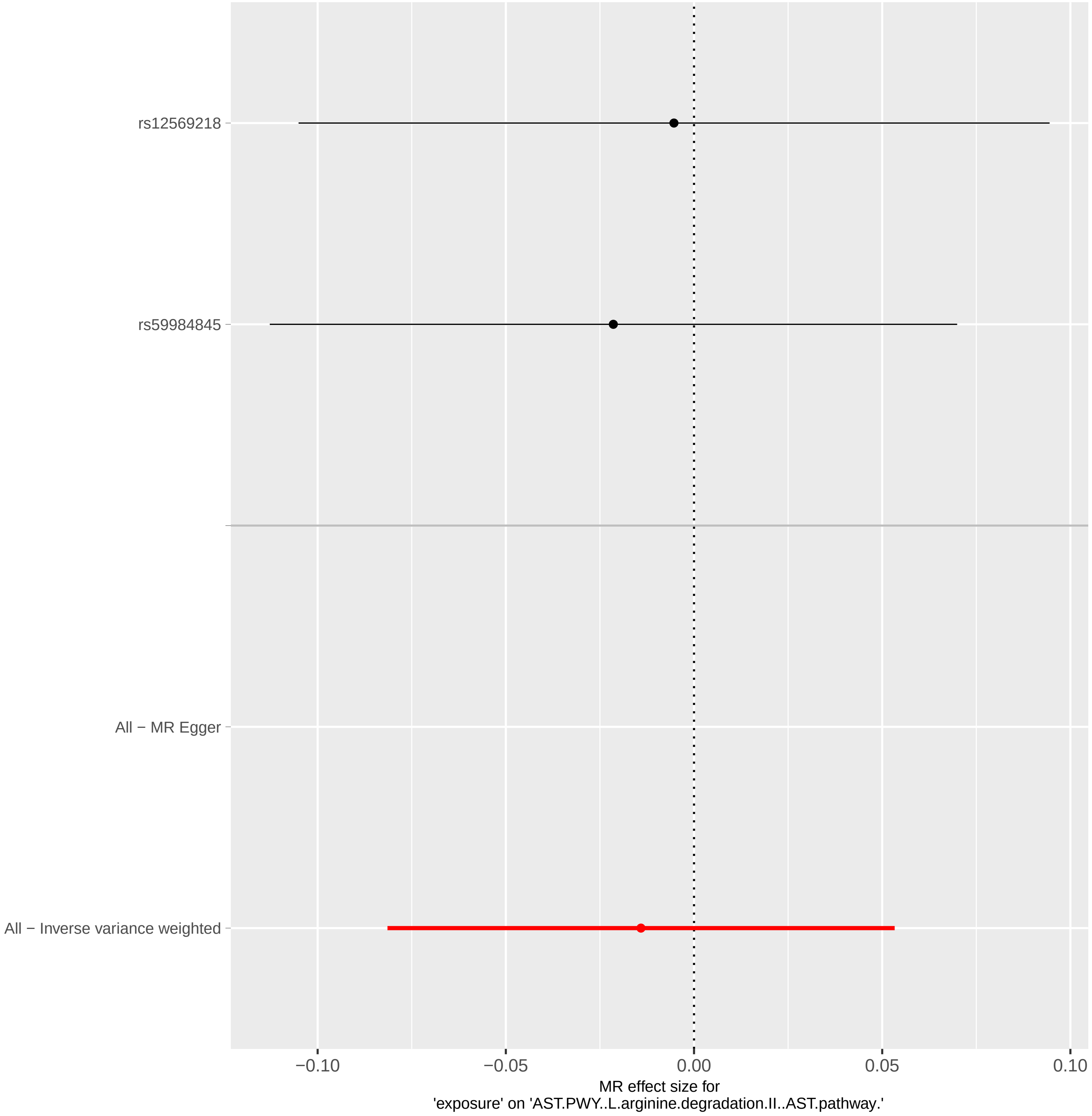

rs12569218
rs59984845
All − MR Egger
All − Inverse variance weighted
−0.10
−0.05
0.00
0.05
0.10
MR effect size for
'exposure' on 'AST.PWY..L.arginine.degradation.II..AST.pathway.'

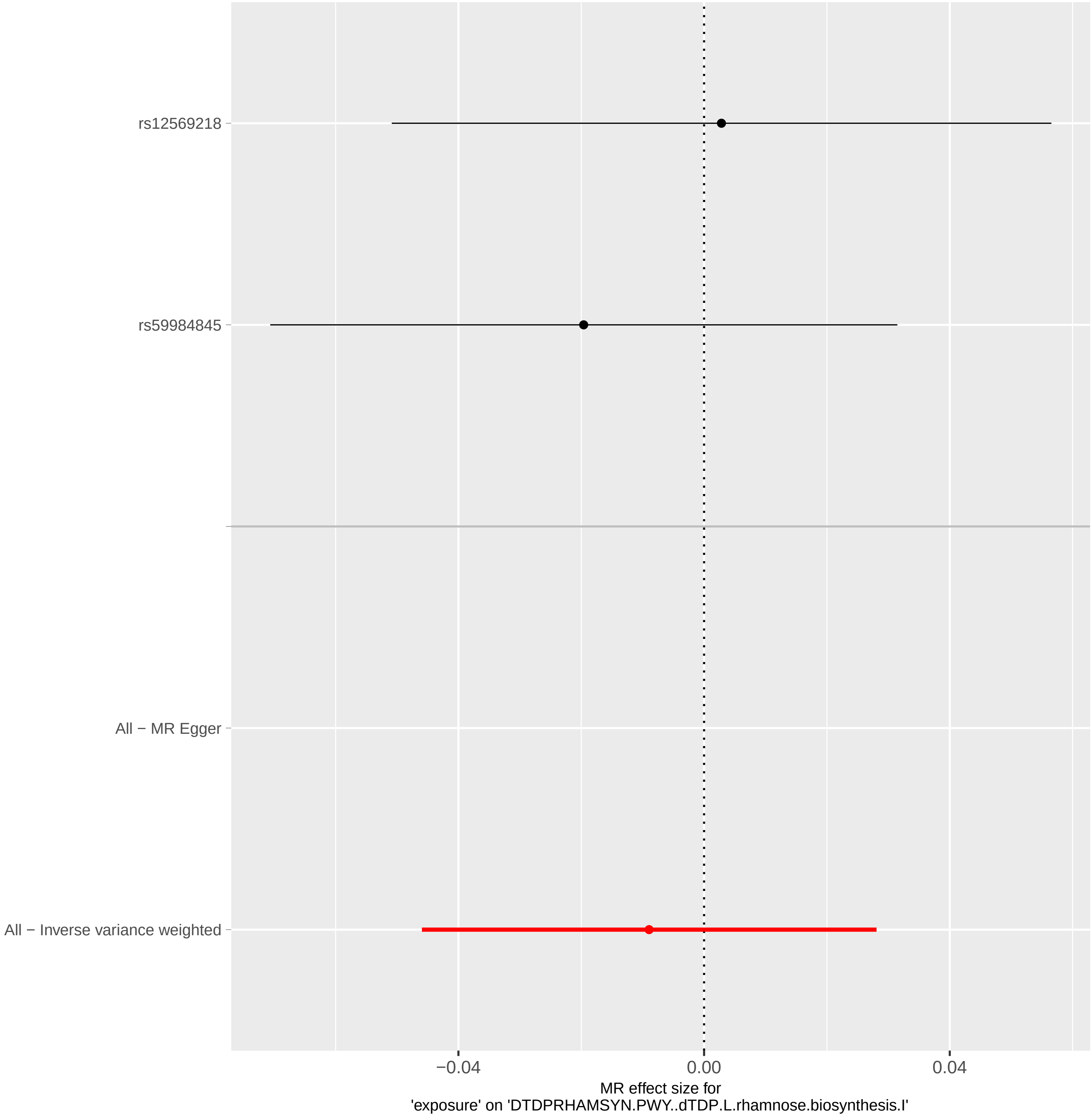
rs12569218
rs59984845
All − MR Egger
All − Inverse variance weighted
−0.04
0.00
0.04
MR effect size for
'exposure' on 'DTDPRHAMSYN.PWY..dTDP.L.rhamnose.biosynthesis.I'

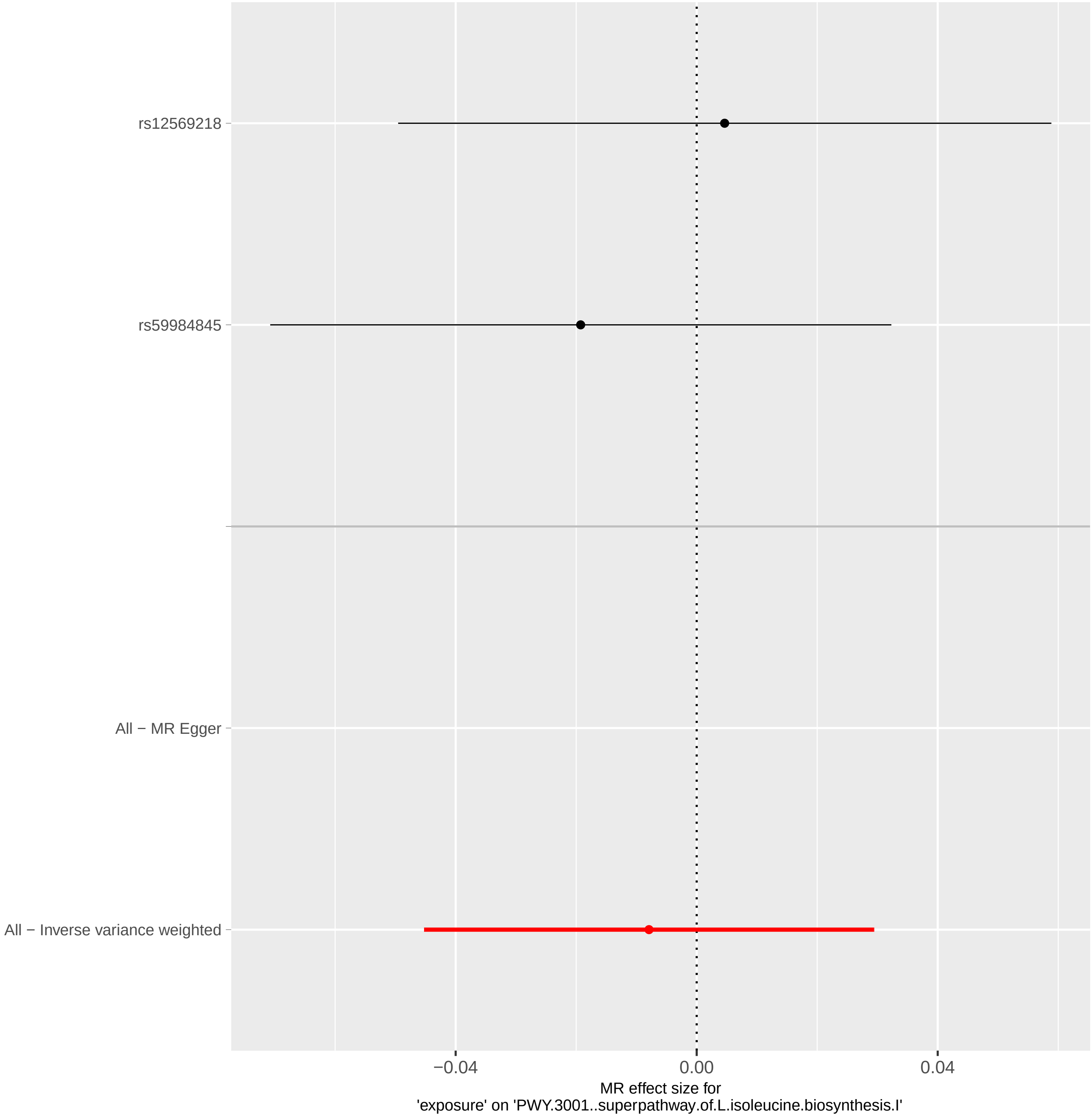
rs12569218
rs59984845
All − MR Egger
All − Inverse variance weighted
−0.04
0.00
0.04
MR effect size for
'exposure' on 'PWY.3001..superpathway.of.L.isoleucine.biosynthesis.I'

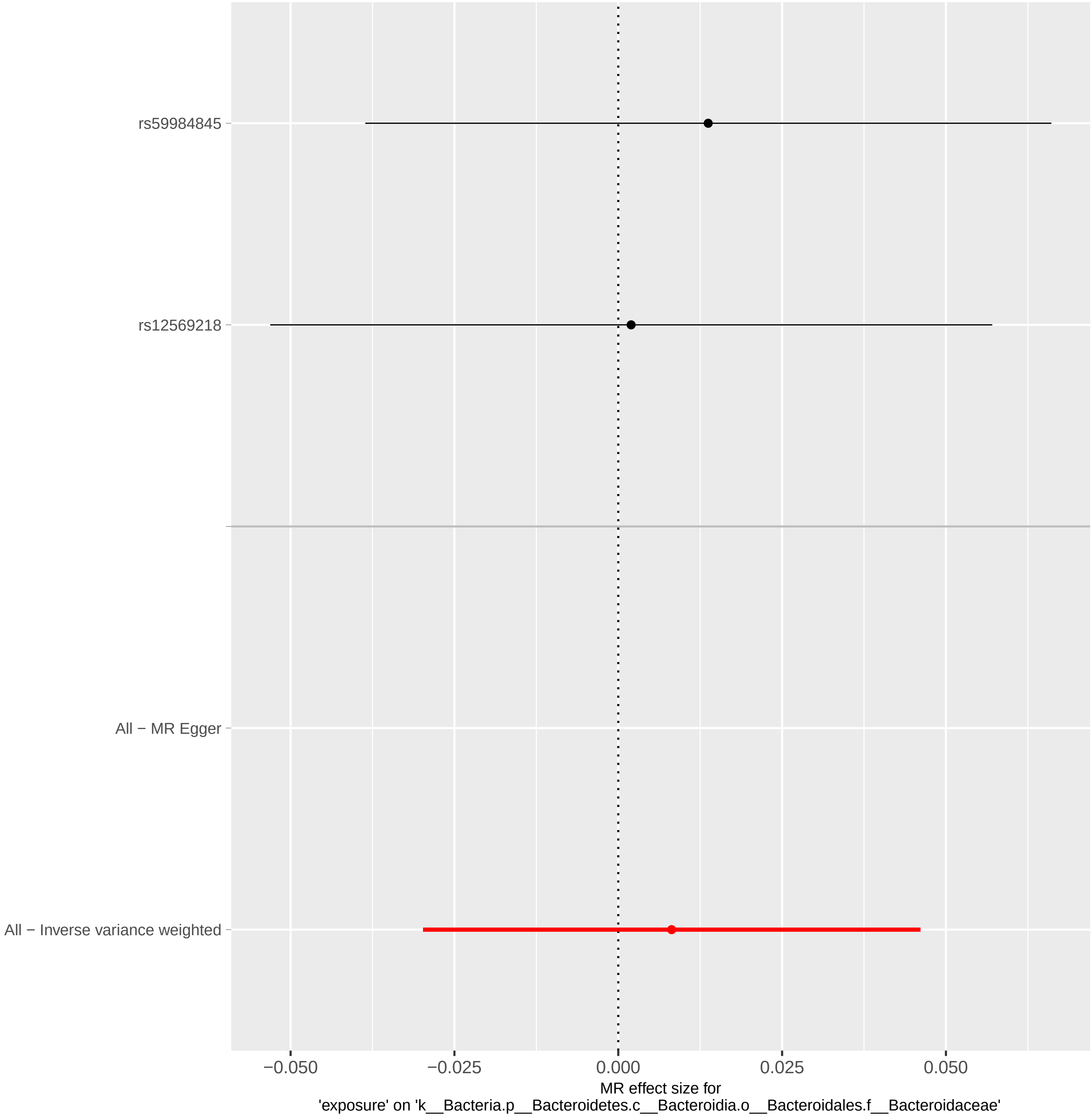

rs59984845
rs12569218
All − MR Egger
All − Inverse variance weighted
−0.050
−0.025
0.000
0.025
0.050
MR effect size for
'exposure' on 'k__Bacteria.p__Bacteroidetes.c__Bacteroidia.o__Bacteroidales.f__Bacteroidaceae'

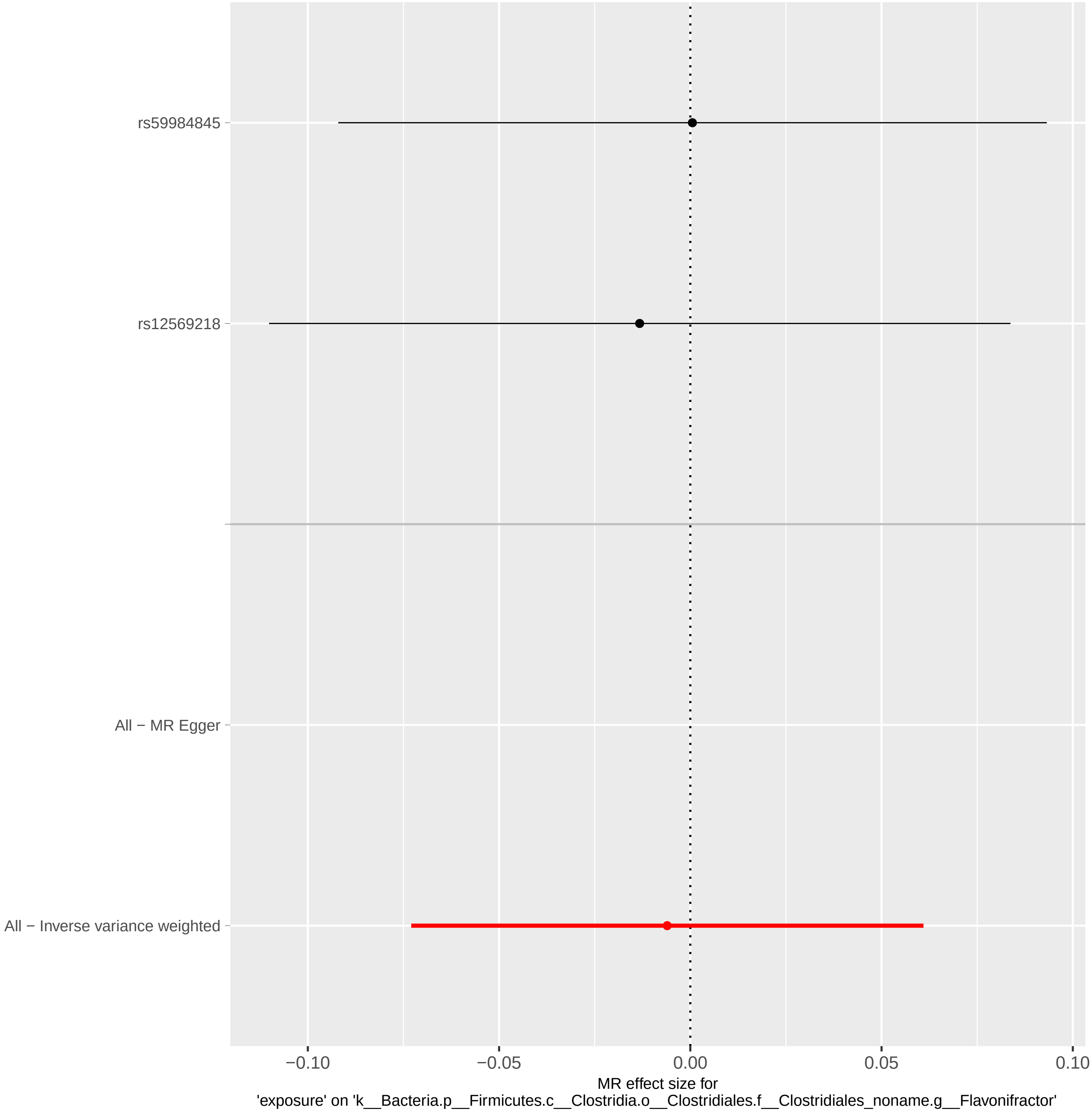
rs59984845
rs12569218
All − MR Egger
All − Inverse variance weighted
−0.10
−0.05
0.00
0.05
0.10
MR effect size for
'exposure' on 'k__Bacteria.p__Firmicutes.c__Clostridia.o__Clostridiales.f__Clostridiales_noname.g__Flavonifractor'

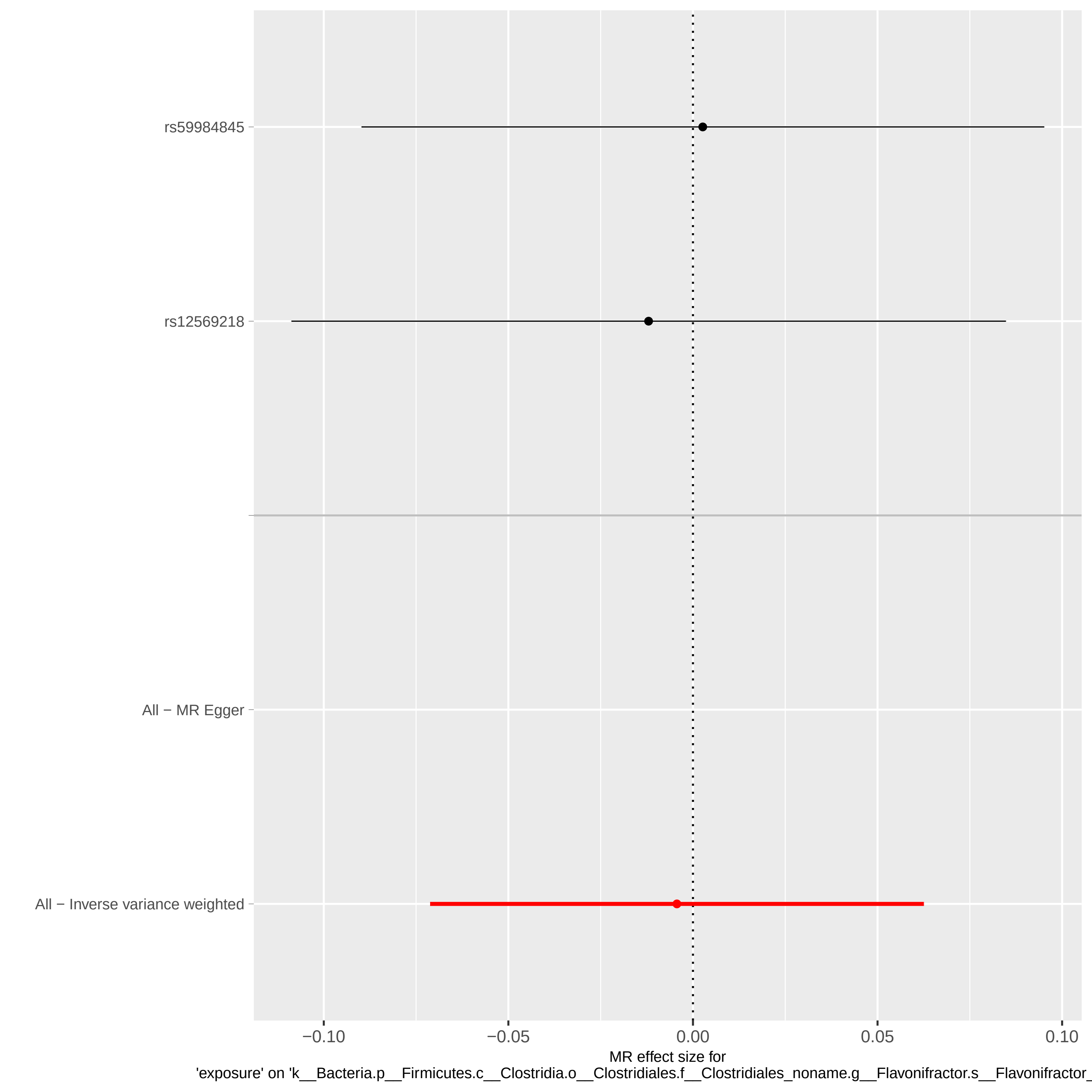

rs59984845
rs12569218
All − MR Egger
All − Inverse variance weighted
−0.10
−0.05
0.00
0.05
0.10
MR effect size for
'exposure' on 'k__Bacteria.p__Firmicutes.c__Clostridia.o__Clostridiales.f__Clostridiales_noname.g__Flavonifractor.s__Flavonifractor_

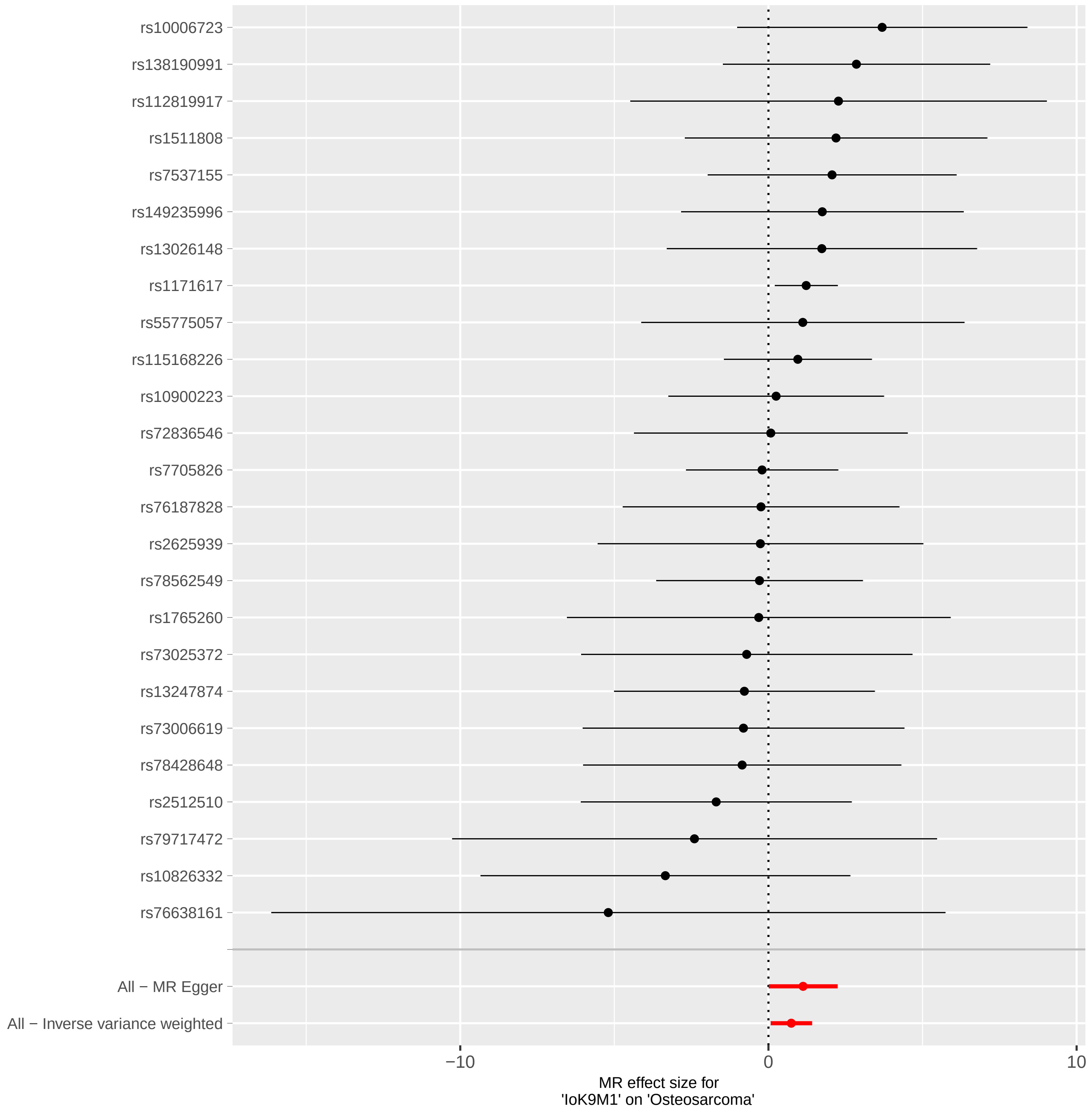
rs10006723
rs138190991
rs112819917
rs1511808
rs7537155
rs149235996
rs13026148
rs1171617
rs55775057
rs115168226
rs10900223
rs72836546
rs7705826
rs76187828
rs2625939
rs78562549
rs1765260
rs73025372
rs13247874
rs73006619
rs78428648
rs2512510
rs79717472
rs10826332
rs76638161
All − MR Egger
All − Inverse variance weighted
−10
0
10
MR effect size for
'IoK9M1' on 'Osteosarcoma'

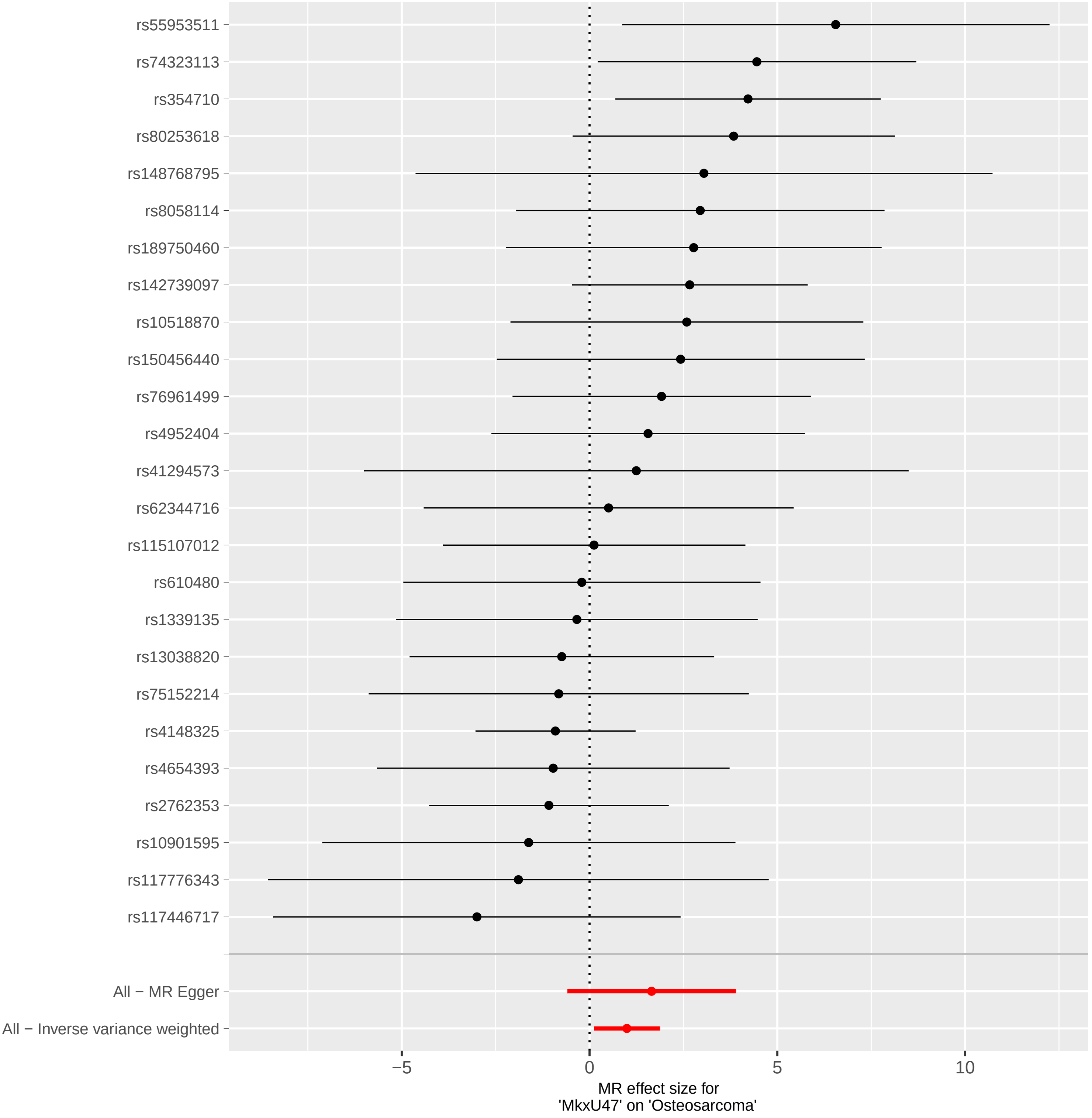
rs55953511
rs74323113
rs354710
rs80253618
rs148768795
rs8058114
rs189750460
rs142739097
rs10518870
rs150456440
rs76961499
rs4952404
rs41294573
rs62344716
rs115107012
rs610480
rs1339135
rs13038820
rs75152214
rs4148325
rs4654393
rs2762353
rs10901595
rs117776343
rs117446717
All − MR Egger
All − Inverse variance weighted
−5
0
5
10
MR effect size for
'MkxU47' on 'Osteosarcoma'

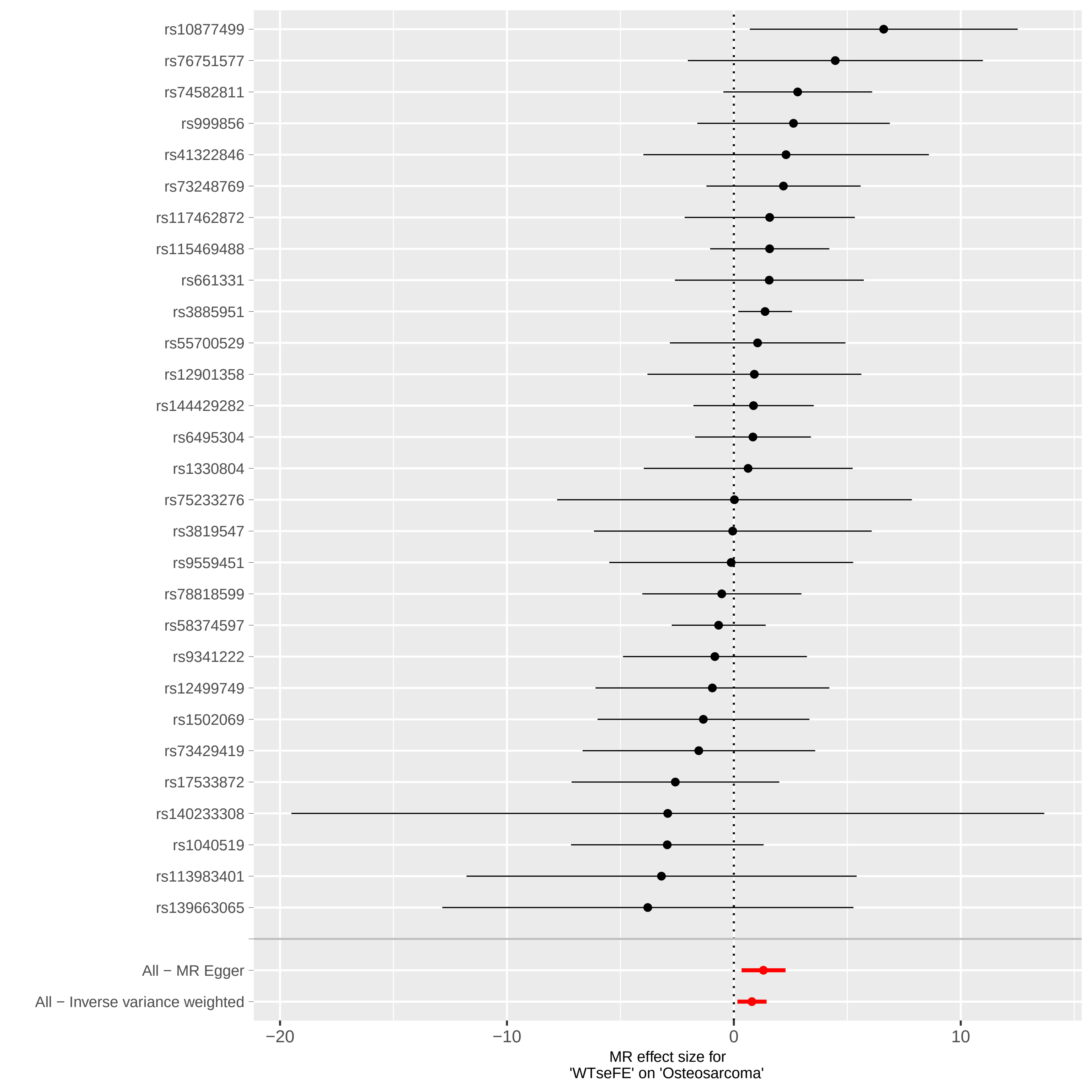
rs10877499
rs76751577
rs74582811
rs999856
rs41322846
rs73248769
rs117462872
rs115469488
rs661331
rs3885951
rs55700529
rs12901358
rs144429282
rs6495304
rs1330804
rs75233276
rs3819547
rs9559451
rs78818599
rs58374597
rs9341222
rs12499749
rs1502069
rs73429419
rs17533872
rs140233308
rs1040519
rs113983401
rs139663065
All − MR Egger
All − Inverse variance weighted
−20
−10
0
10
MR effect size for
'WTseFE' on 'Osteosarcoma'

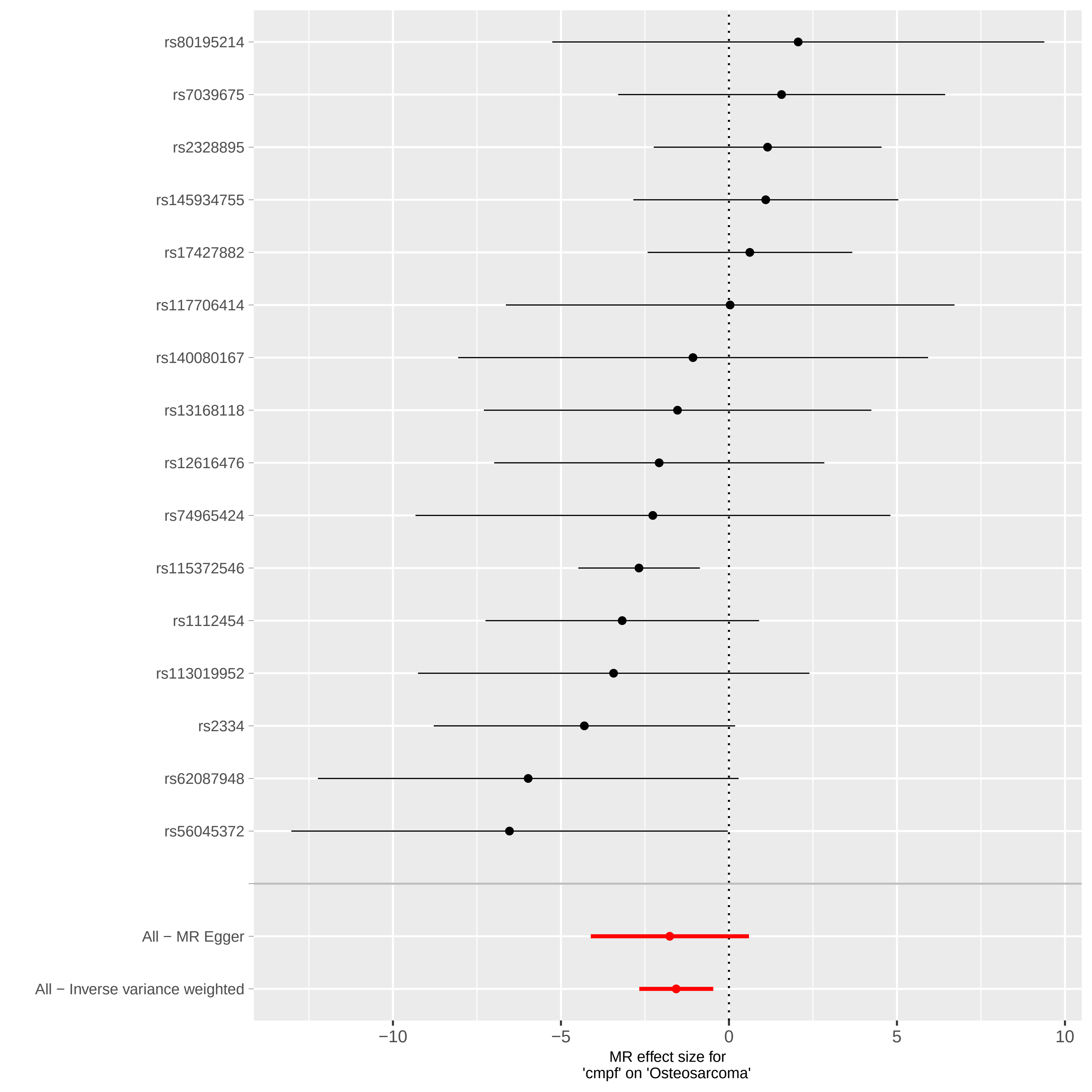
rs80195214
rs7039675
rs2328895
rs145934755
rs17427882
rs117706414
rs140080167
rs13168118
rs12616476
rs74965424
rs115372546
rs1112454
rs113019952
rs2334
rs62087948
rs56045372
All − MR Egger
All − Inverse variance weighted
−10
−5
0
5
10
MR effect size for
'cmpf' on 'Osteosarcoma'

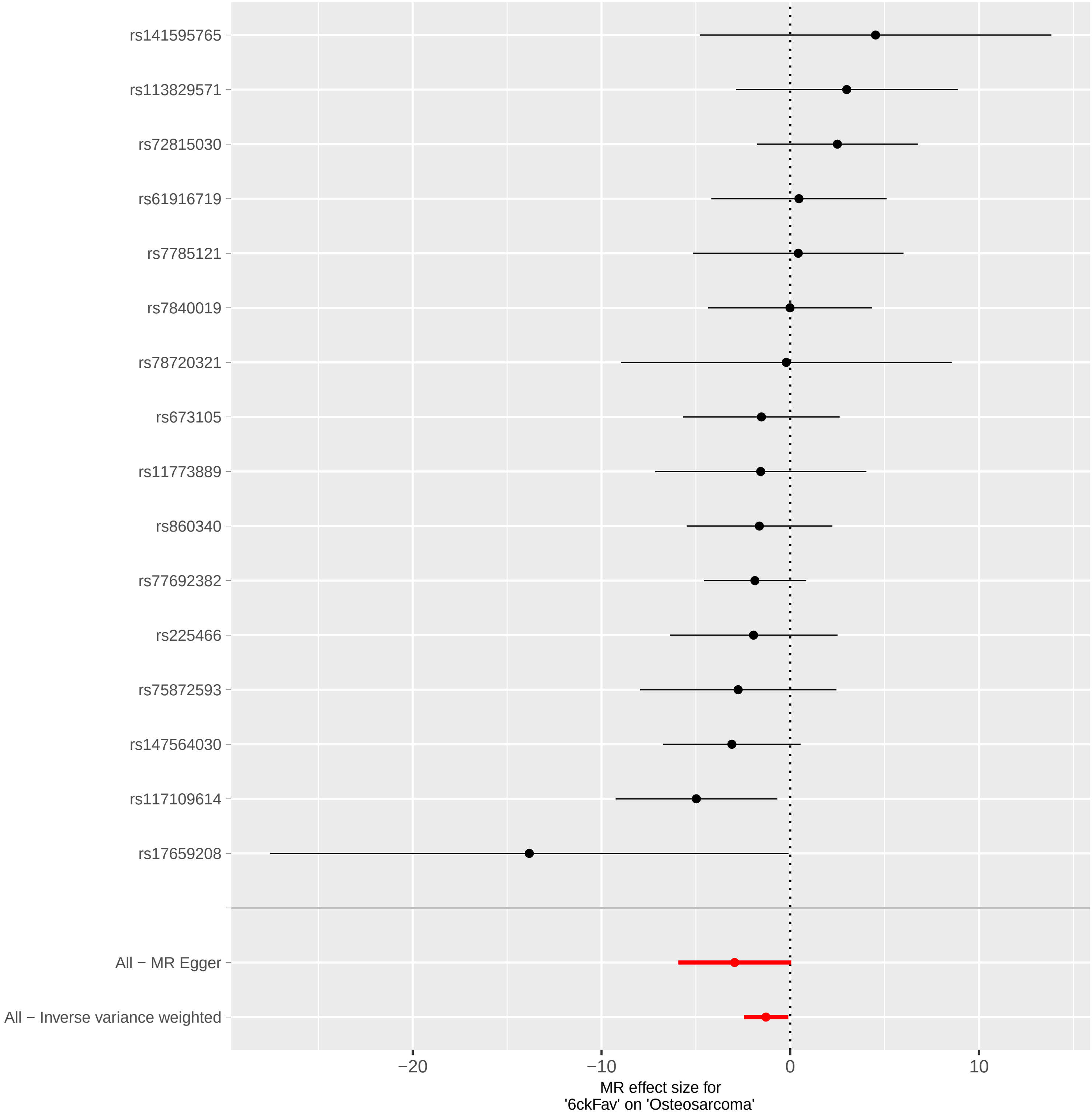
rs141595765
rs113829571
rs72815030
rs61916719
rs7785121
rs7840019
rs78720321
rs673105
rs11773889
rs860340
rs77692382
rs225466
rs75872593
rs147564030
rs117109614
rs17659208
All − MR Egger
All − Inverse variance weighted
−20
−10
0
10
MR effect size for
'6ckFav' on 'Osteosarcoma'

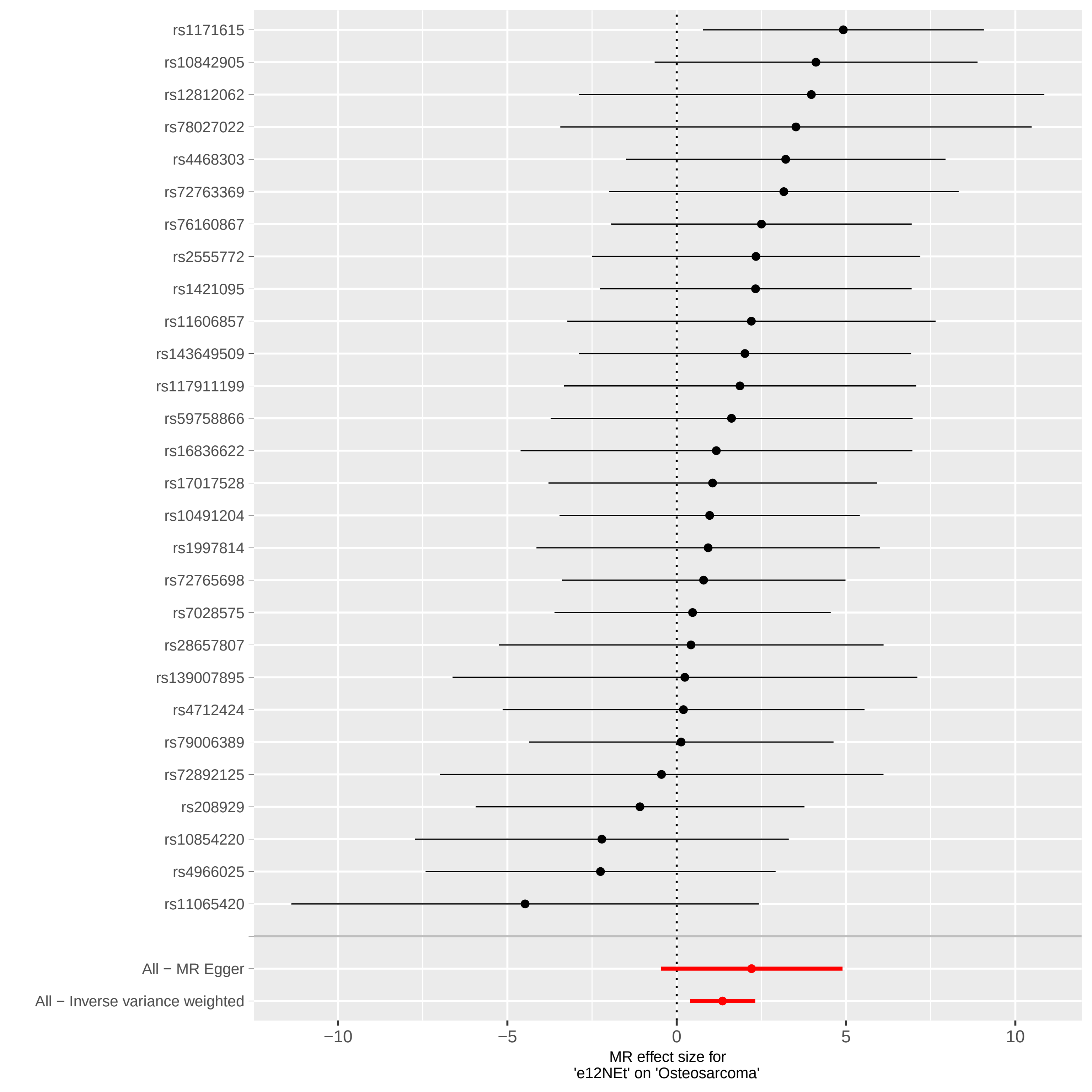
rs1171615
rs10842905
rs12812062
rs78027022
rs4468303
rs72763369
rs76160867
rs2555772
rs1421095
rs11606857
rs143649509
rs117911199
rs59758866
rs16836622
rs17017528
rs10491204
rs1997814
rs72765698
rs7028575
rs28657807
rs139007895
rs4712424
rs79006389
rs72892125
rs208929
rs10854220
rs4966025
rs11065420
All − MR Egger
All − Inverse variance weighted
−10
−5
0
5
10
MR effect size for
'e12NEt' on 'Osteosarcoma'

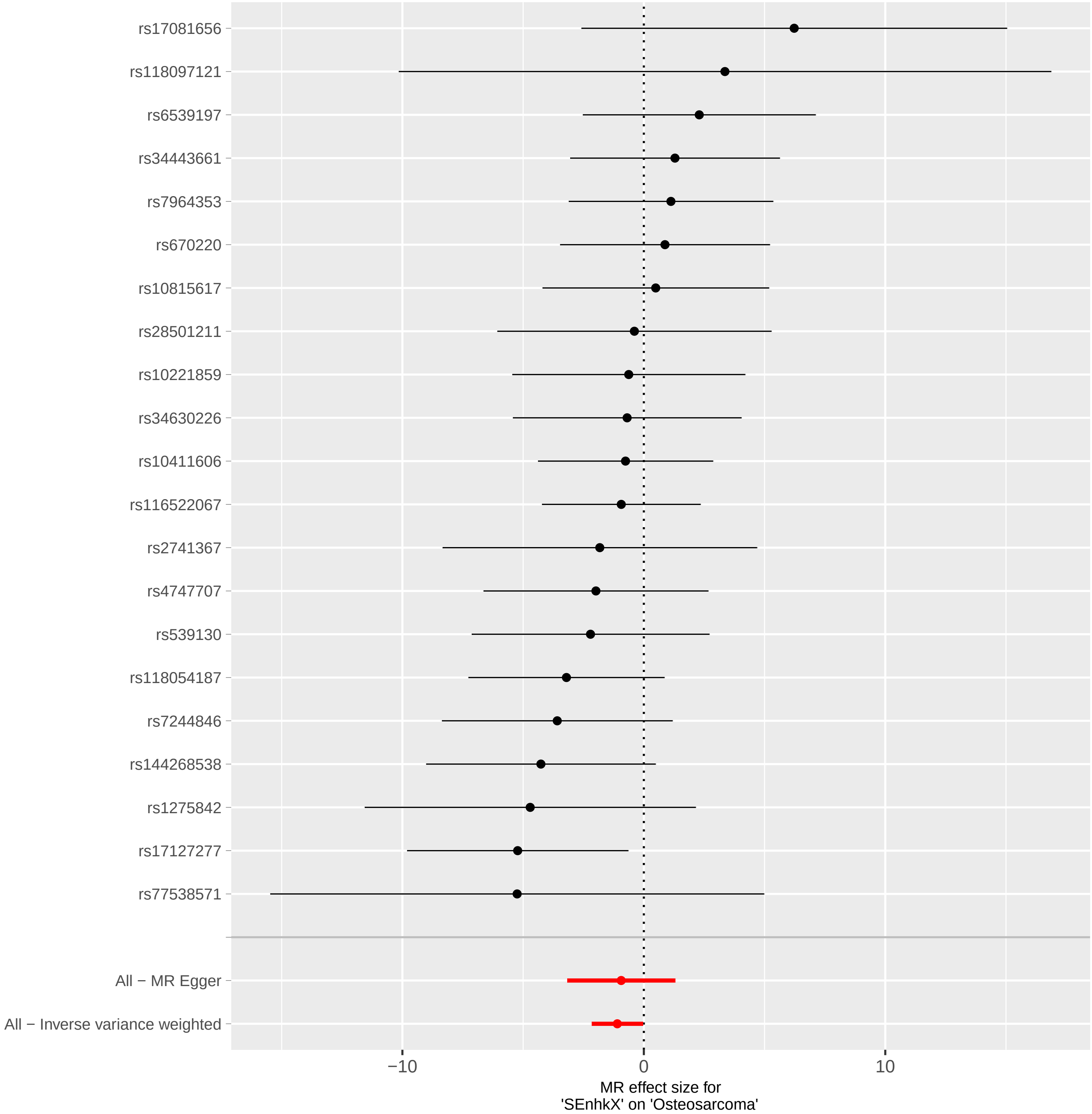

rs17081656
rs118097121
rs6539197
rs34443661
rs7964353
rs670220
rs10815617
rs28501211
rs10221859
rs34630226
rs10411606
rs116522067
rs2741367
rs4747707
rs539130
rs118054187
rs7244846
rs144268538
rs1275842
rs17127277
rs77538571
All − MR Egger
All − Inverse variance weighted
−10
0
10
MR effect size for
'SEnhkX' on 'Osteosarcoma'

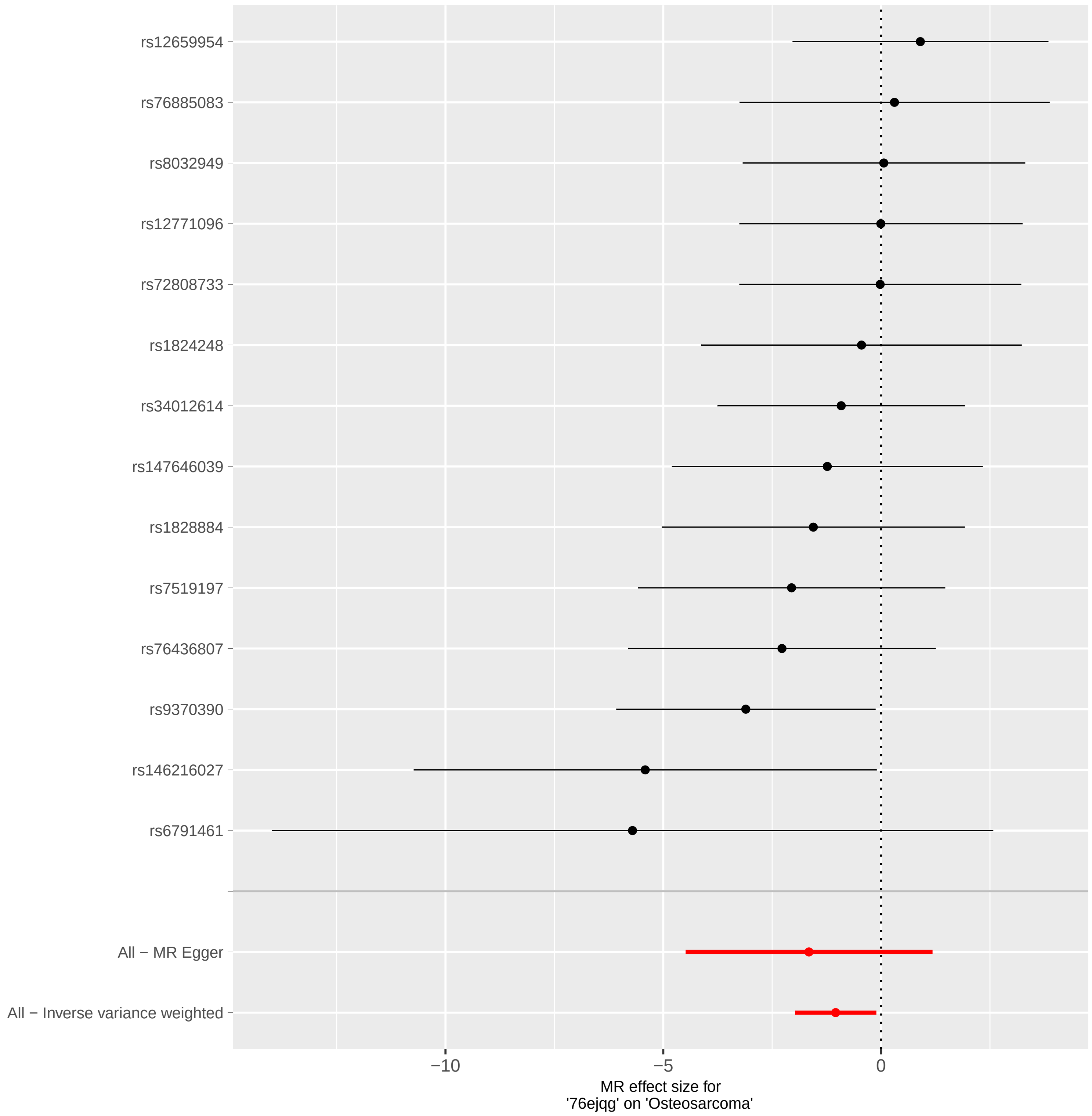

rs12659954
rs76885083
rs8032949
rs12771096
rs72808733
rs1824248
rs34012614
rs147646039
rs1828884
rs7519197
rs76436807
rs9370390
rs146216027
rs6791461
All − MR Egger
All − Inverse variance weighted
−10
−5
0
MR effect size for
'76ejqg' on 'Osteosarcoma'

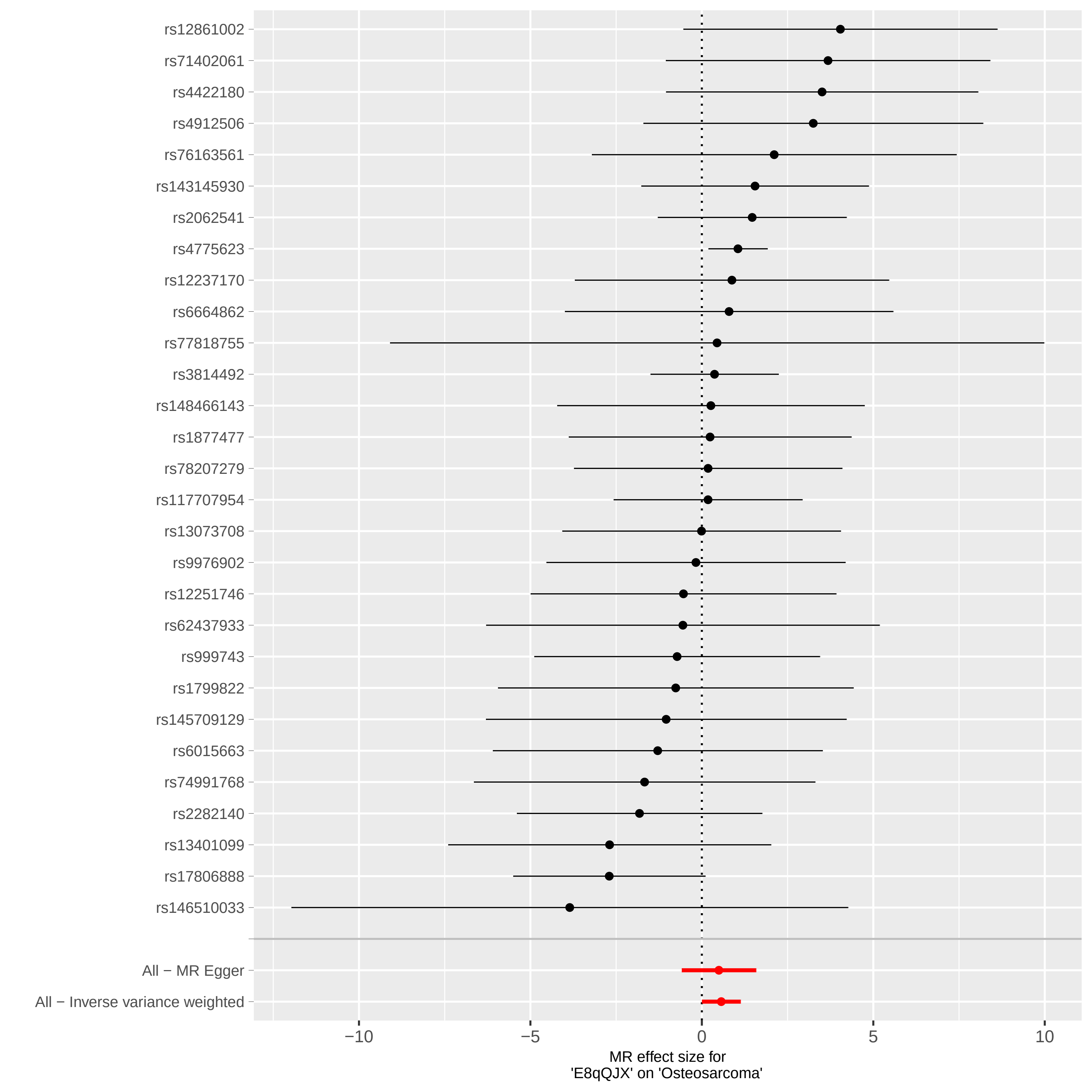
rs12861002
rs71402061
rs4422180
rs4912506
rs76163561
rs143145930
rs2062541
rs4775623
rs12237170
rs6664862
rs77818755
rs3814492
rs148466143
rs1877477
rs78207279
rs117707954
rs13073708
rs9976902
rs12251746
rs62437933
rs999743
rs1799822
rs145709129
rs6015663
rs74991768
rs2282140
rs13401099
rs17806888
rs146510033
All − MR Egger
All − Inverse variance weighted
−10
−5
0
5
10
MR effect size for
'E8qQJX' on 'Osteosarcoma'

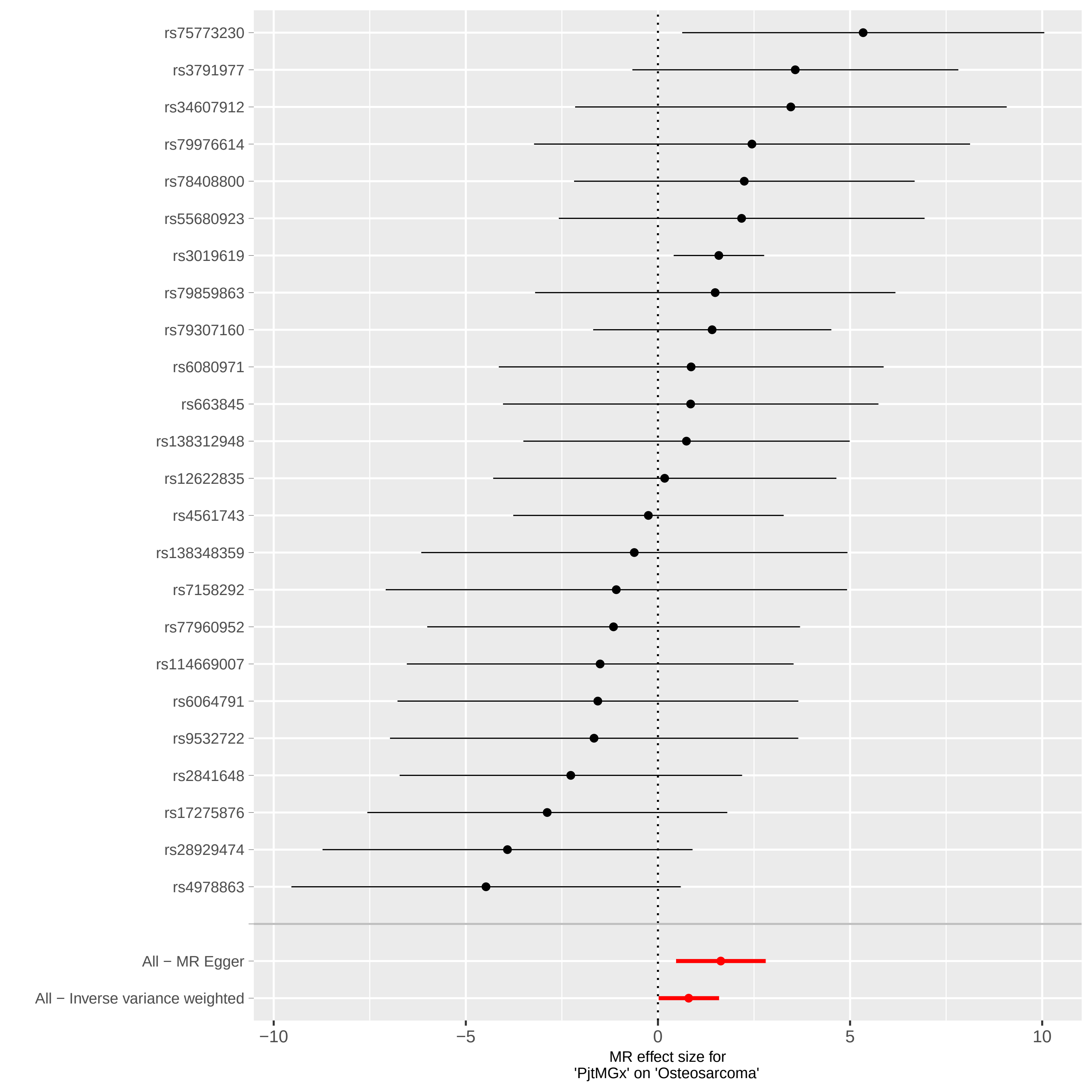

rs75773230
rs3791977
rs34607912
rs79976614
rs78408800
rs55680923
rs3019619
rs79859863
rs79307160
rs6080971
rs663845
rs138312948
rs12622835
rs4561743
rs138348359
rs7158292
rs77960952
rs114669007
rs6064791
rs9532722
rs2841648
rs17275876
rs28929474
rs4978863
All − MR Egger
All − Inverse variance weighted
−10
−5
0
5
10
MR effect size for
'PjtMGx' on 'Osteosarcoma'

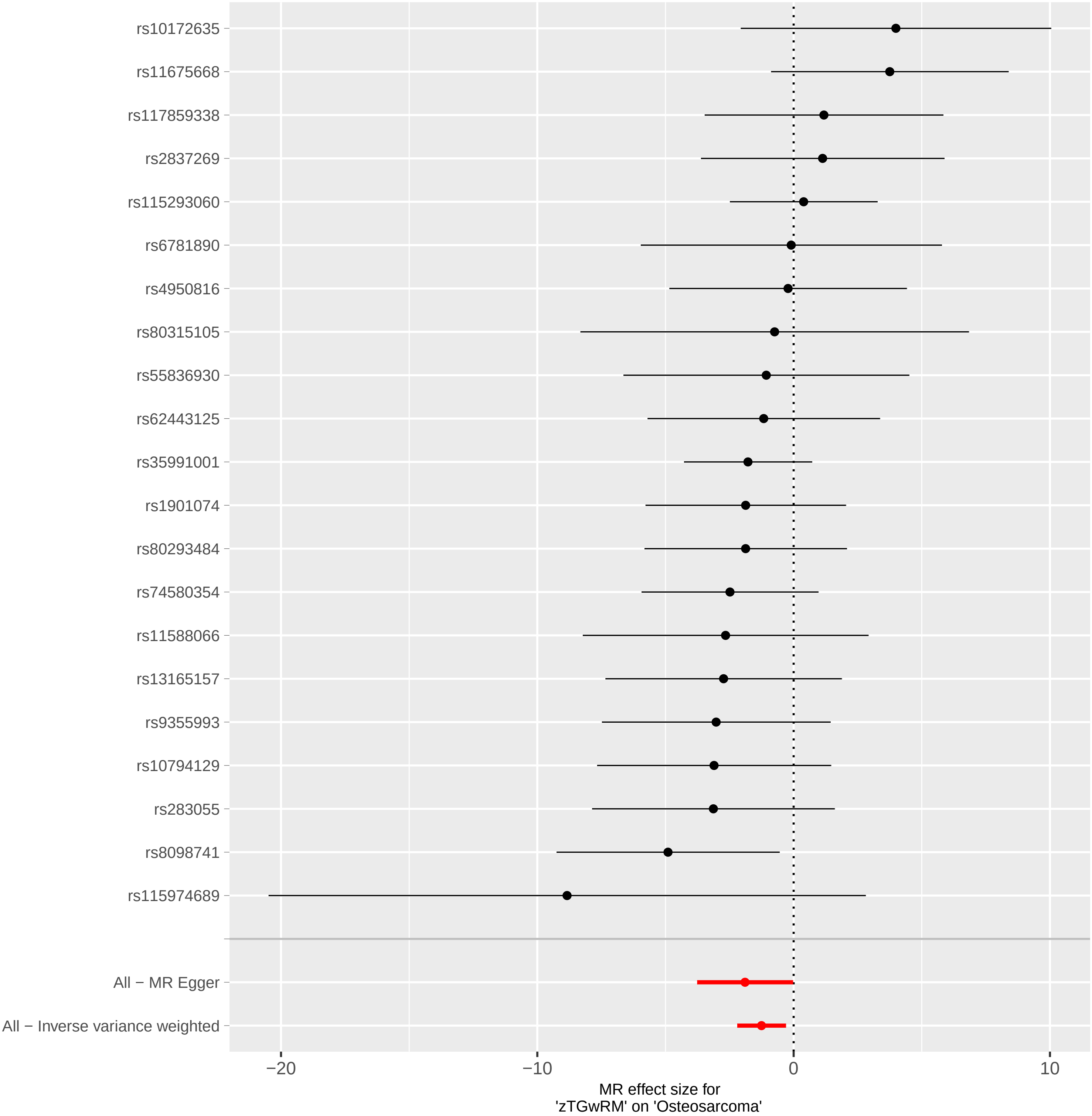
rs10172635
rs11675668
rs117859338
rs2837269
rs115293060
rs6781890
rs4950816
rs80315105
rs55836930
rs62443125
rs35991001
rs1901074
rs80293484
rs74580354
rs11588066
rs13165157
rs9355993
rs10794129
rs283055
rs8098741
rs115974689
All − MR Egger
All − Inverse variance weighted
−20
−10
0
10
MR effect size for
'zTGwRM' on 'Osteosarcoma'

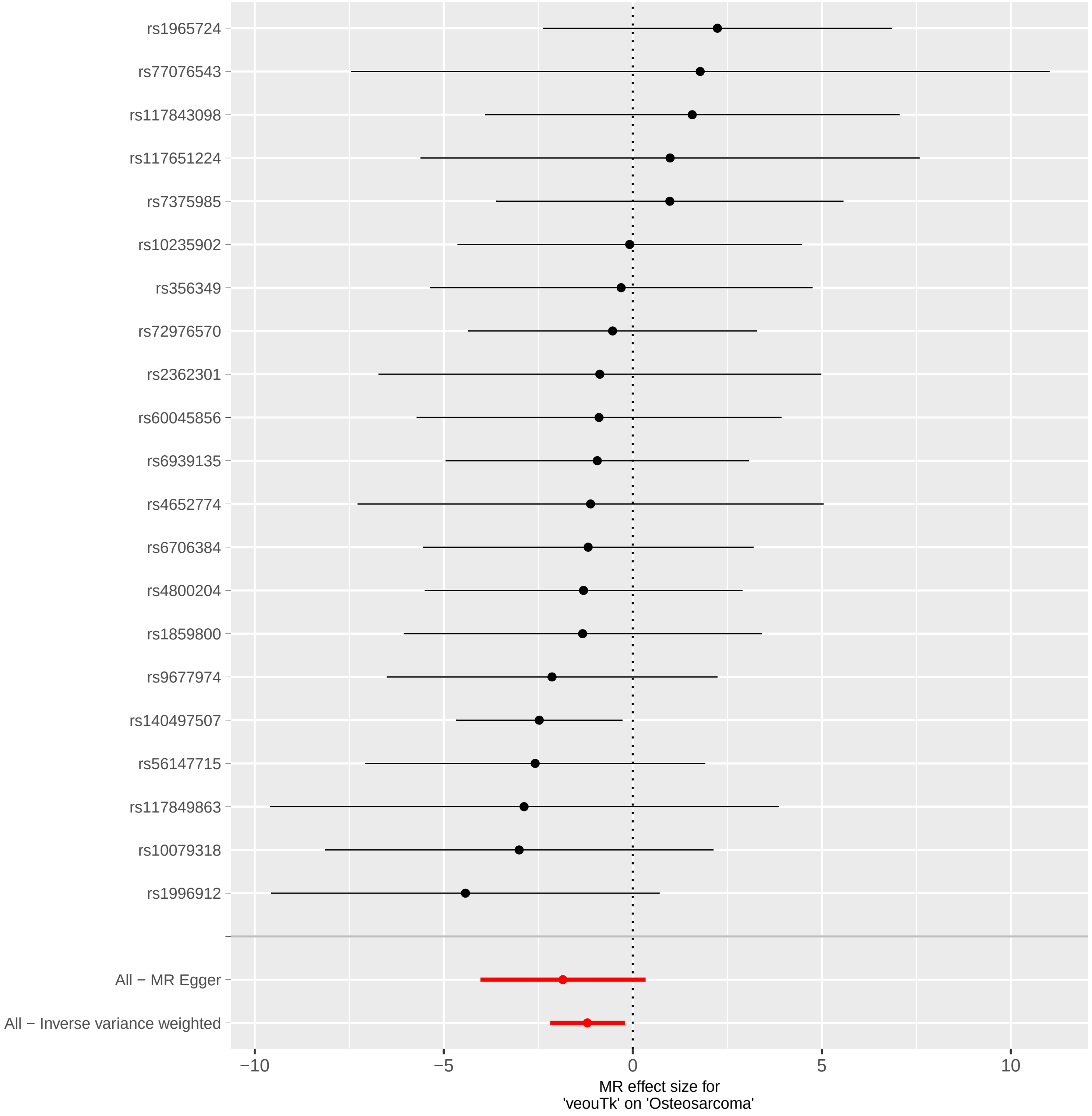
rs1965724
rs77076543
rs117843098
rs117651224
rs7375985
rs10235902
rs356349
rs72976570
rs2362301
rs60045856
rs6939135
rs4652774
rs6706384
rs4800204
rs1859800
rs9677974
rs140497507
rs56147715
rs117849863
rs10079318
rs1996912
All − MR Egger
All − Inverse variance weighted
−10
−5
0
5
10
MR effect size for
'veouTk' on 'Osteosarcoma'

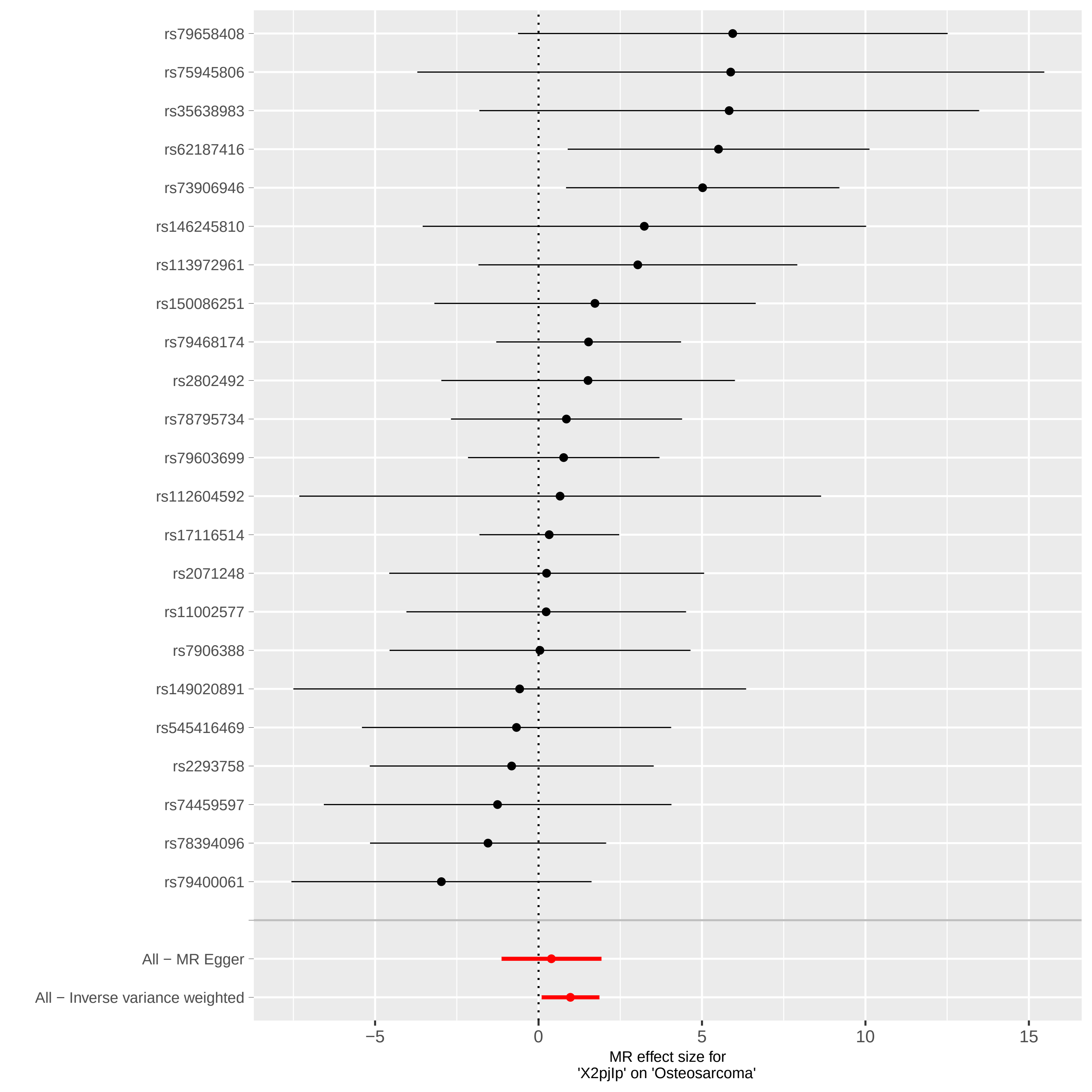
rs79658408
rs75945806
rs35638983
rs62187416
rs73906946
rs146245810
rs113972961
rs150086251
rs79468174
rs2802492
rs78795734
rs79603699
rs112604592
rs17116514
rs2071248
rs11002577
rs7906388
rs149020891
rs545416469
rs2293758
rs74459597
rs78394096
rs79400061
All − MR Egger
All − Inverse variance weighted
−5
0
5
10
15
MR effect size for
'X2pjIp' on 'Osteosarcoma'

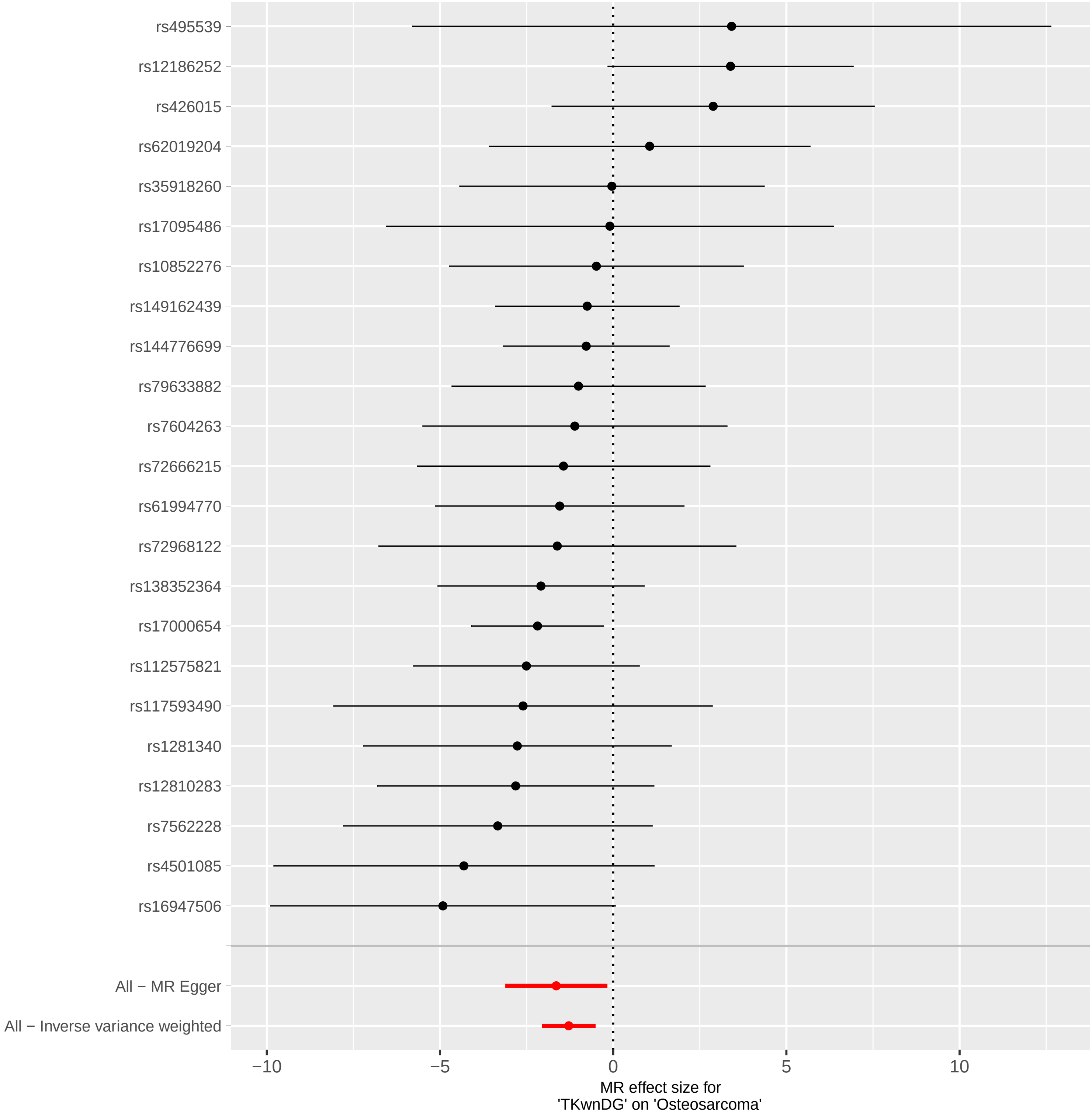
rs495539
rs12186252
rs426015
rs62019204
rs35918260
rs17095486
rs10852276
rs149162439
rs144776699
rs79633882
rs7604263
rs72666215
rs61994770
rs72968122
rs138352364
rs17000654
rs112575821
rs117593490
rs1281340
rs12810283
rs7562228
rs4501085
rs16947506
All − MR Egger
All − Inverse variance weighted
−10
−5
0
5
10
MR effect size for
'TKwnDG' on 'Osteosarcoma'

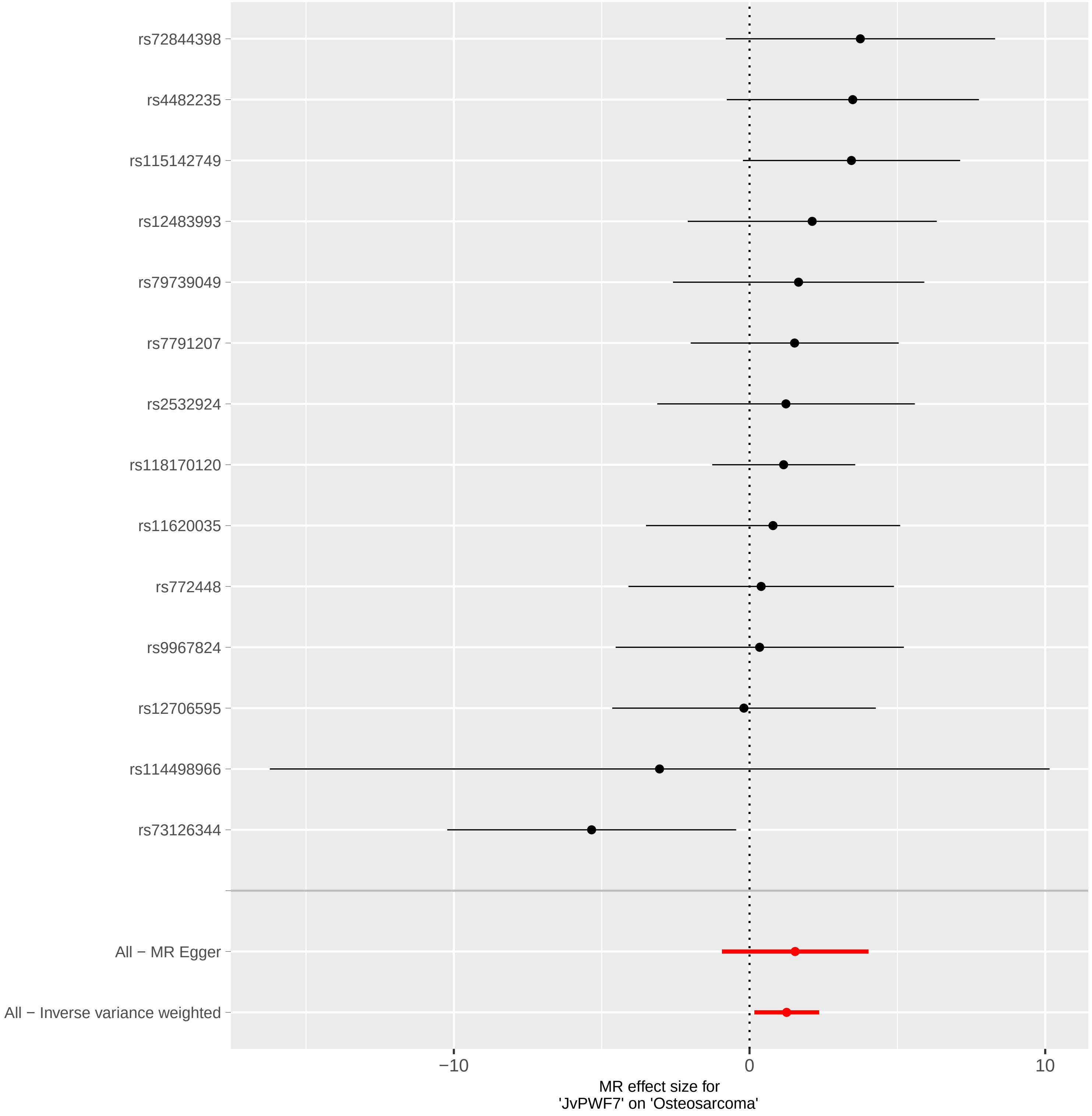
rs72844398
rs4482235
rs115142749
rs12483993
rs79739049
rs7791207
rs2532924
rs118170120
rs11620035
rs772448
rs9967824
rs12706595
rs114498966
rs73126344
All − MR Egger
All − Inverse variance weighted
−10
0
10
MR effect size for
'JvPWF7' on 'Osteosarcoma'

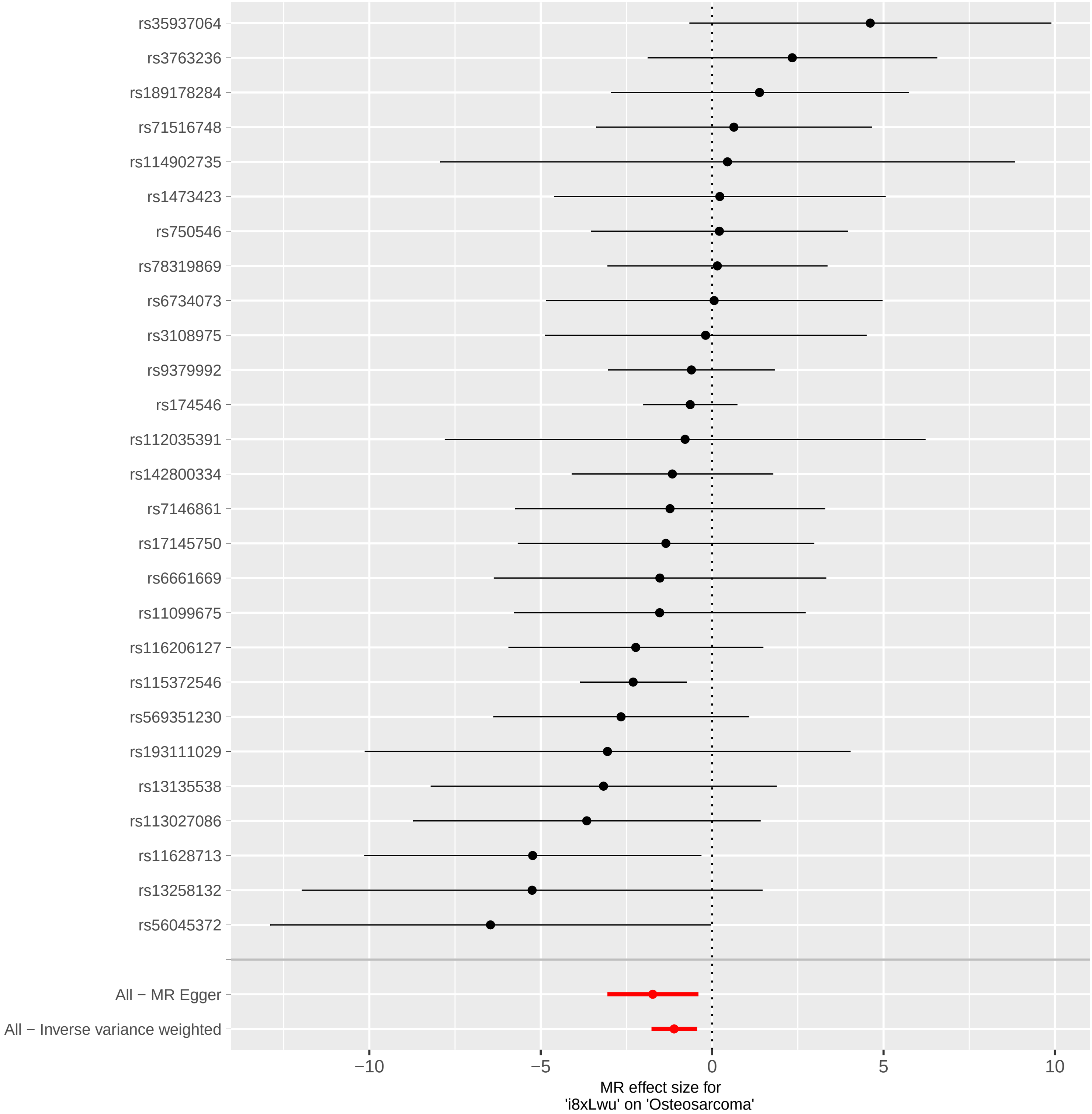
rs35937064
rs3763236
rs189178284
rs71516748
rs114902735
rs1473423
rs750546
rs78319869
rs6734073
rs3108975
rs9379992
rs174546
rs112035391
rs142800334
rs7146861
rs17145750
rs6661669
rs11099675
rs116206127
rs115372546
rs569351230
rs193111029
rs13135538
rs113027086
rs11628713
rs13258132
rs56045372
All − MR Egger
All − Inverse variance weighted
−10
−5
0
5
10
MR effect size for
'i8xLwu' on 'Osteosarcoma'

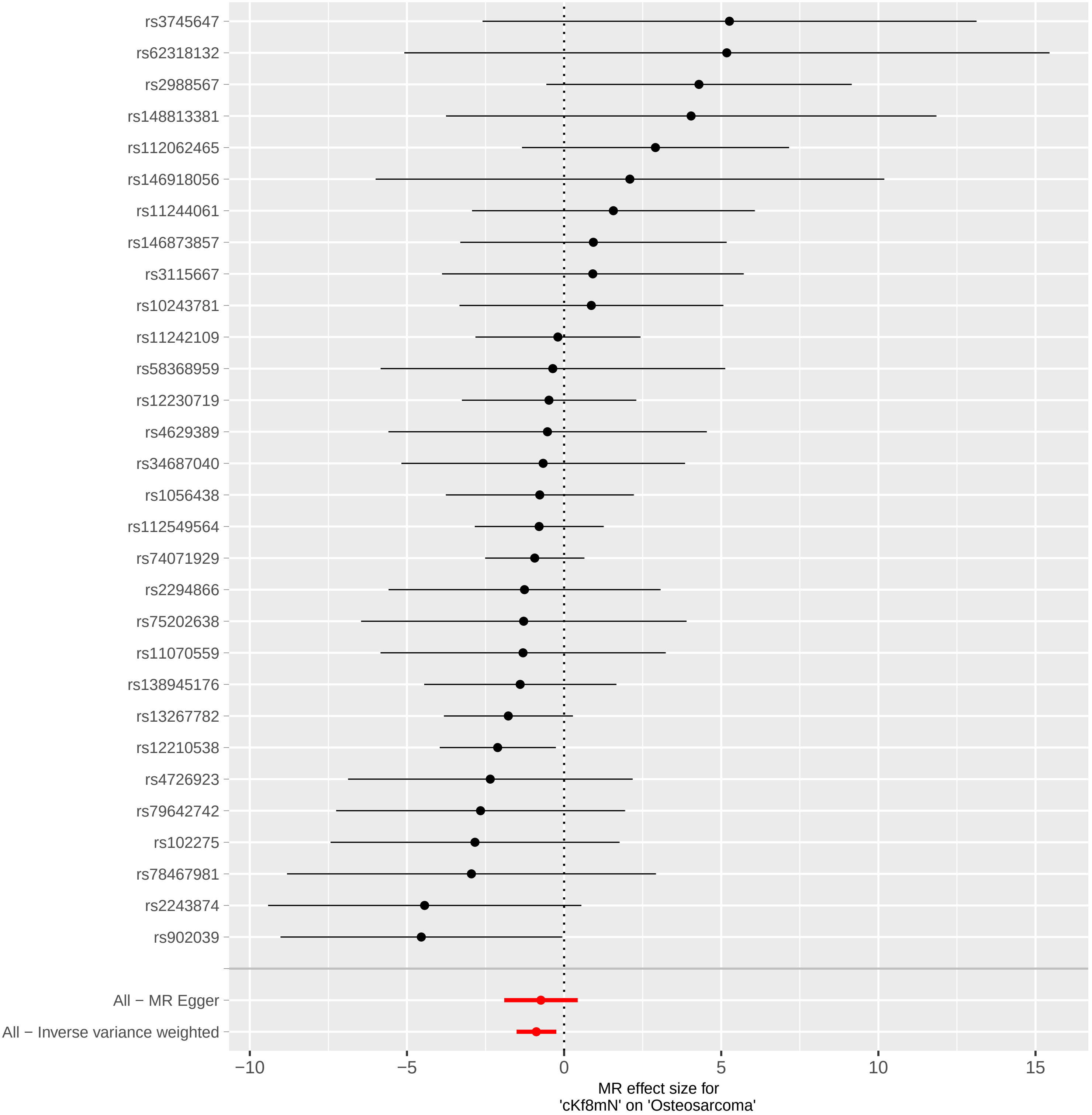
rs3745647
rs62318132
rs2988567
rs148813381
rs112062465
rs146918056
rs11244061
rs146873857
rs3115667
rs10243781
rs11242109
rs58368959
rs12230719
rs4629389
rs34687040
rs1056438
rs112549564
rs74071929
rs2294866
rs75202638
rs11070559
rs138945176
rs13267782
rs12210538
rs4726923
rs79642742
rs102275
rs78467981
rs2243874
rs902039
All − MR Egger
All − Inverse variance weighted
−10
−5
0
5
10
15
MR effect size for
'cKf8mN' on 'Osteosarcoma'

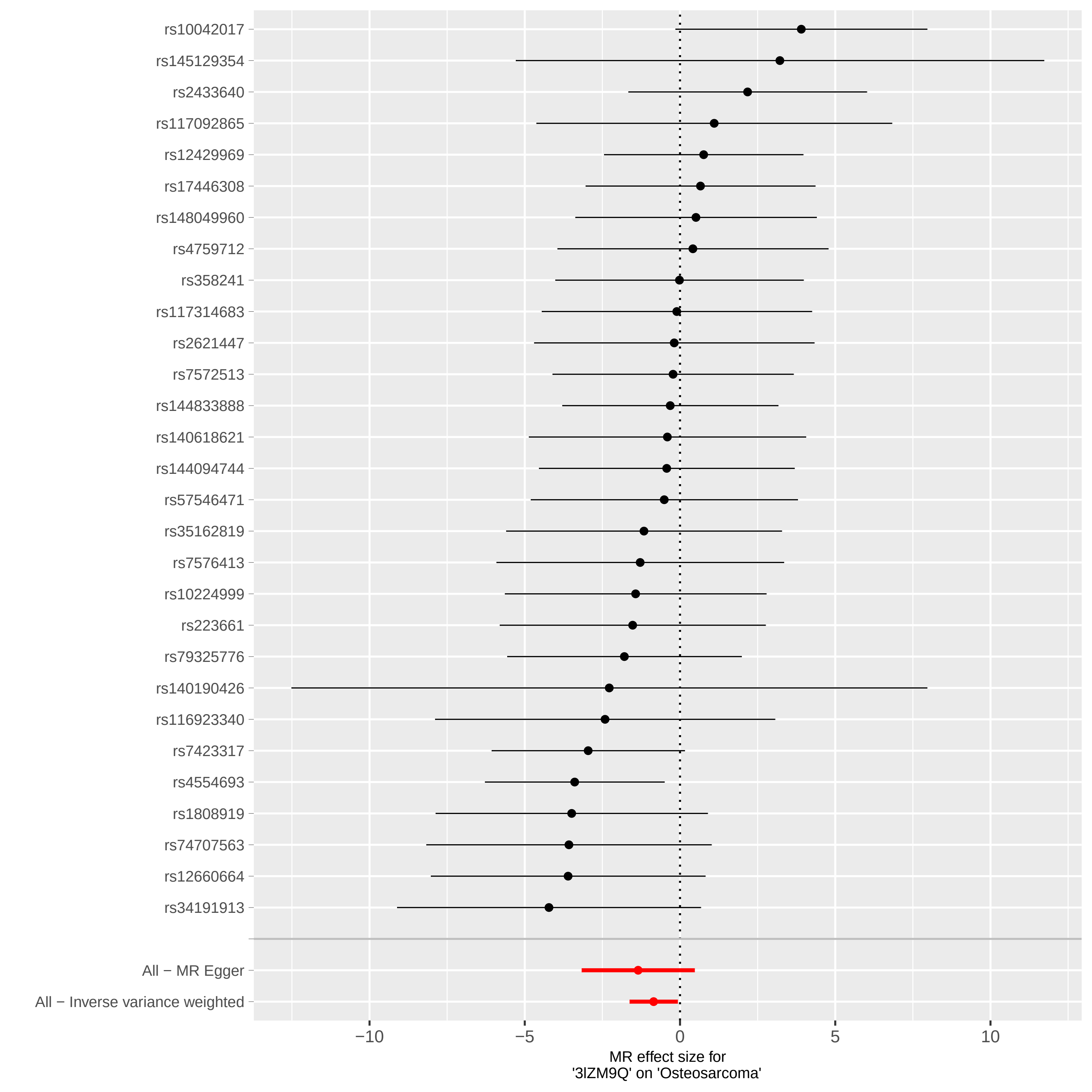
rs10042017
rs145129354
rs2433640
rs117092865
rs12429969
rs17446308
rs148049960
rs4759712
rs358241
rs117314683
rs2621447
rs7572513
rs144833888
rs140618621
rs144094744
rs57546471
rs35162819
rs7576413
rs10224999
rs223661
rs79325776
rs140190426
rs116923340
rs7423317
rs4554693
rs1808919
rs74707563
rs12660664
rs34191913
All − MR Egger
All − Inverse variance weighted
−10
−5
0
5
10
MR effect size for
'3IZM9Q' on 'Osteosarcoma'

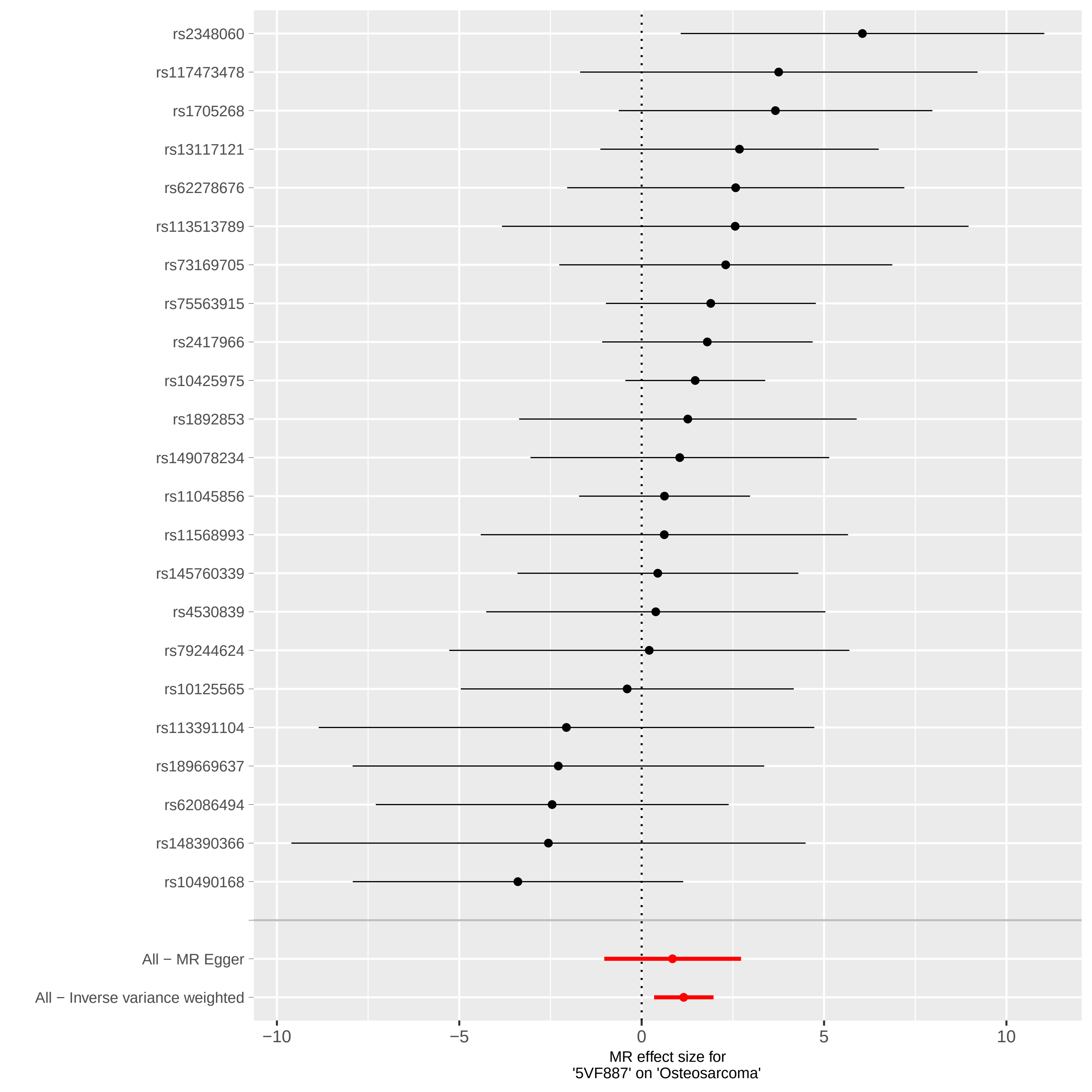

rs2348060
rs117473478
rs1705268
rs13117121
rs62278676
rs113513789
rs73169705
rs75563915
rs2417966
rs10425975
rs1892853
rs149078234
rs11045856
rs11568993
rs145760339
rs4530839
rs79244624
rs10125565
rs113391104
rs189669637
rs62086494
rs148390366
rs10490168
All − MR Egger
All − Inverse variance weighted
−10
−5
0
5
10
MR effect size for
'5VF887' on 'Osteosarcoma'

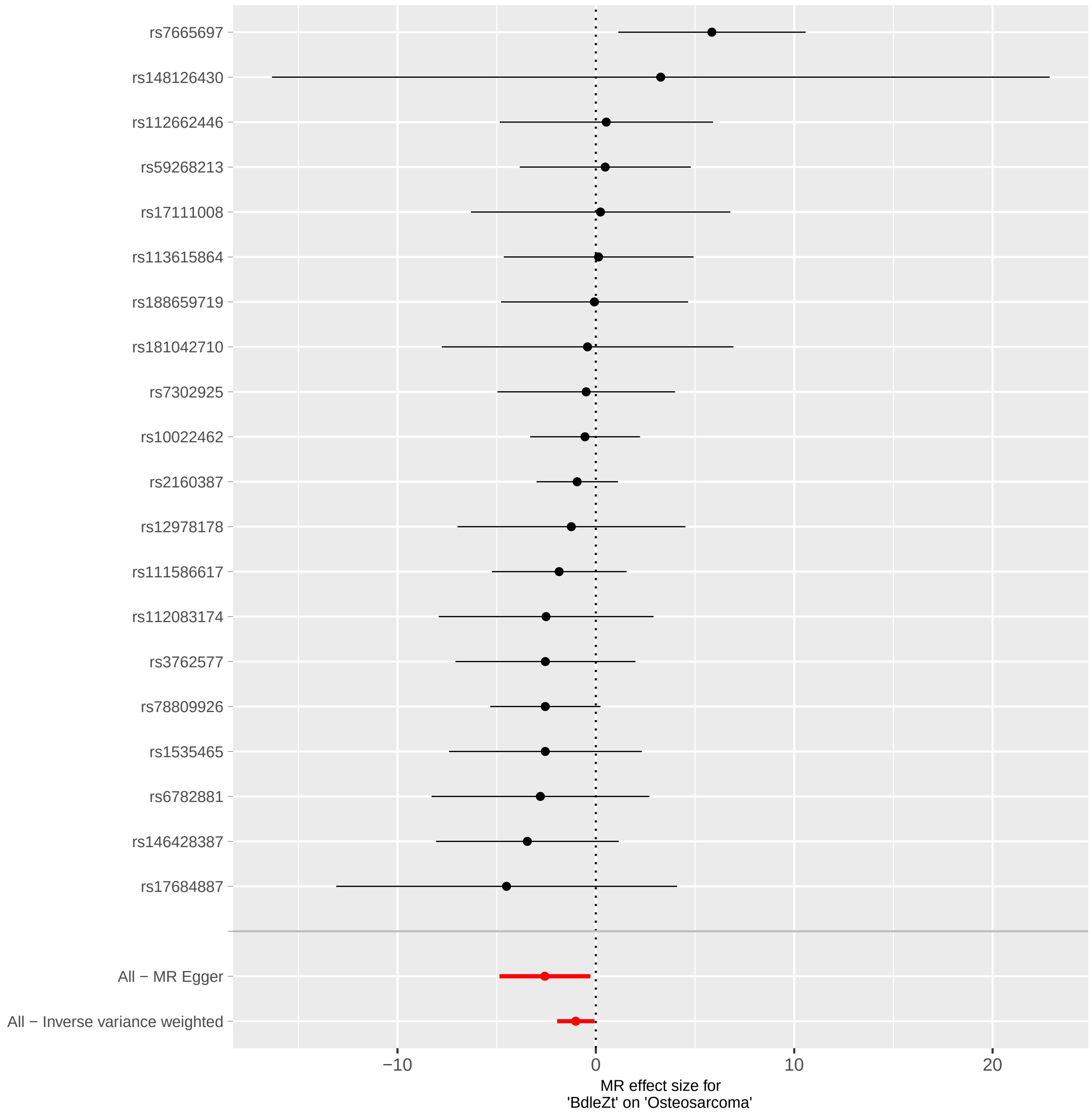

rs7665697
rs148126430
rs112662446
rs59268213
rs17111008
rs113615864
rs188659719
rs181042710
rs7302925
rs10022462
rs2160387
rs12978178
rs111586617
rs112083174
rs3762577
rs78809926
rs1535465
rs6782881
rs146428387
rs17684887
All − MR Egger
All − Inverse variance weighted
−10
0
10
20
MR effect size for
'BdleZt' on 'Osteosarcoma'

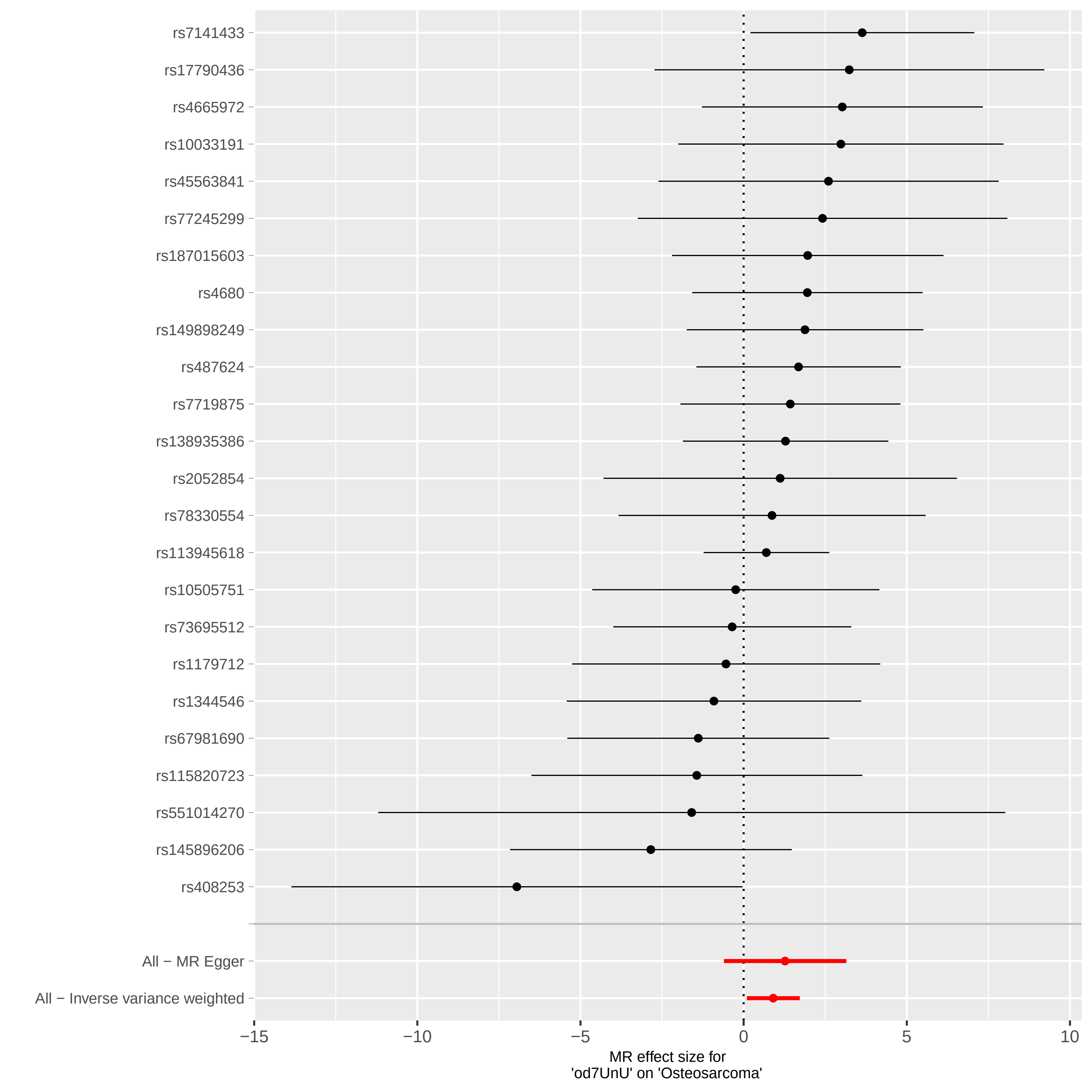
rs7141433
rs17790436
rs4665972
rs10033191
rs45563841
rs77245299
rs187015603
rs4680
rs149898249
rs487624
rs7719875
rs138935386
rs2052854
rs78330554
rs113945618
rs10505751
rs73695512
rs1179712
rs1344546
rs67981690
rs115820723
rs551014270
rs145896206
rs408253
All − MR Egger
All − Inverse variance weighted
−15
−10
−5
0
5
10
MR effect size for
'od7UnU' on 'Osteosarcoma'

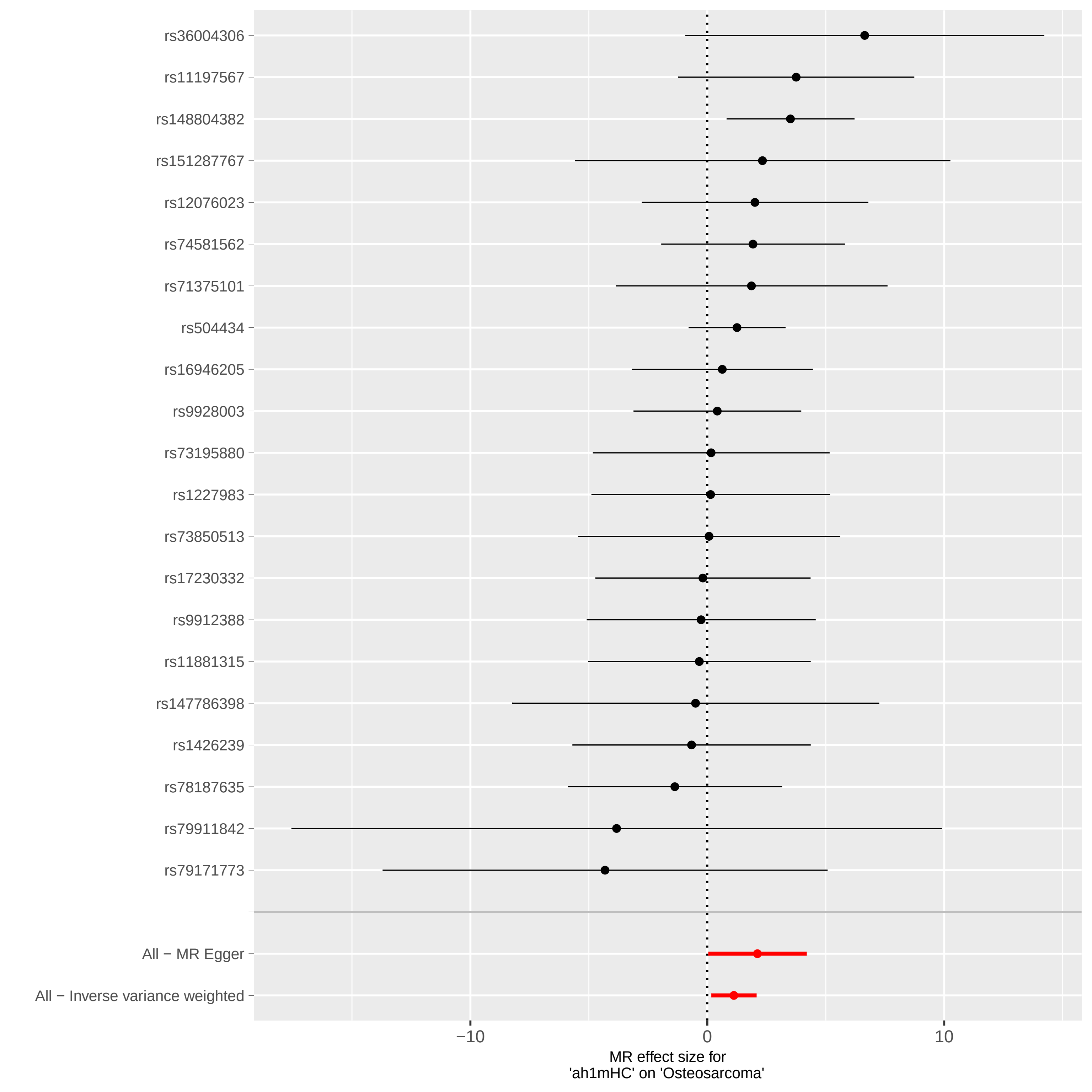

rs36004306
rs11197567
rs148804382
rs151287767
rs12076023
rs74581562
rs71375101
rs504434
rs16946205
rs9928003
rs73195880
rs1227983
rs73850513
rs17230332
rs9912388
rs11881315
rs147786398
rs1426239
rs78187635
rs79911842
rs79171773
All − MR Egger
All − Inverse variance weighted
−10
0
10
MR effect size for
'ah1mHC' on 'Osteosarcoma'

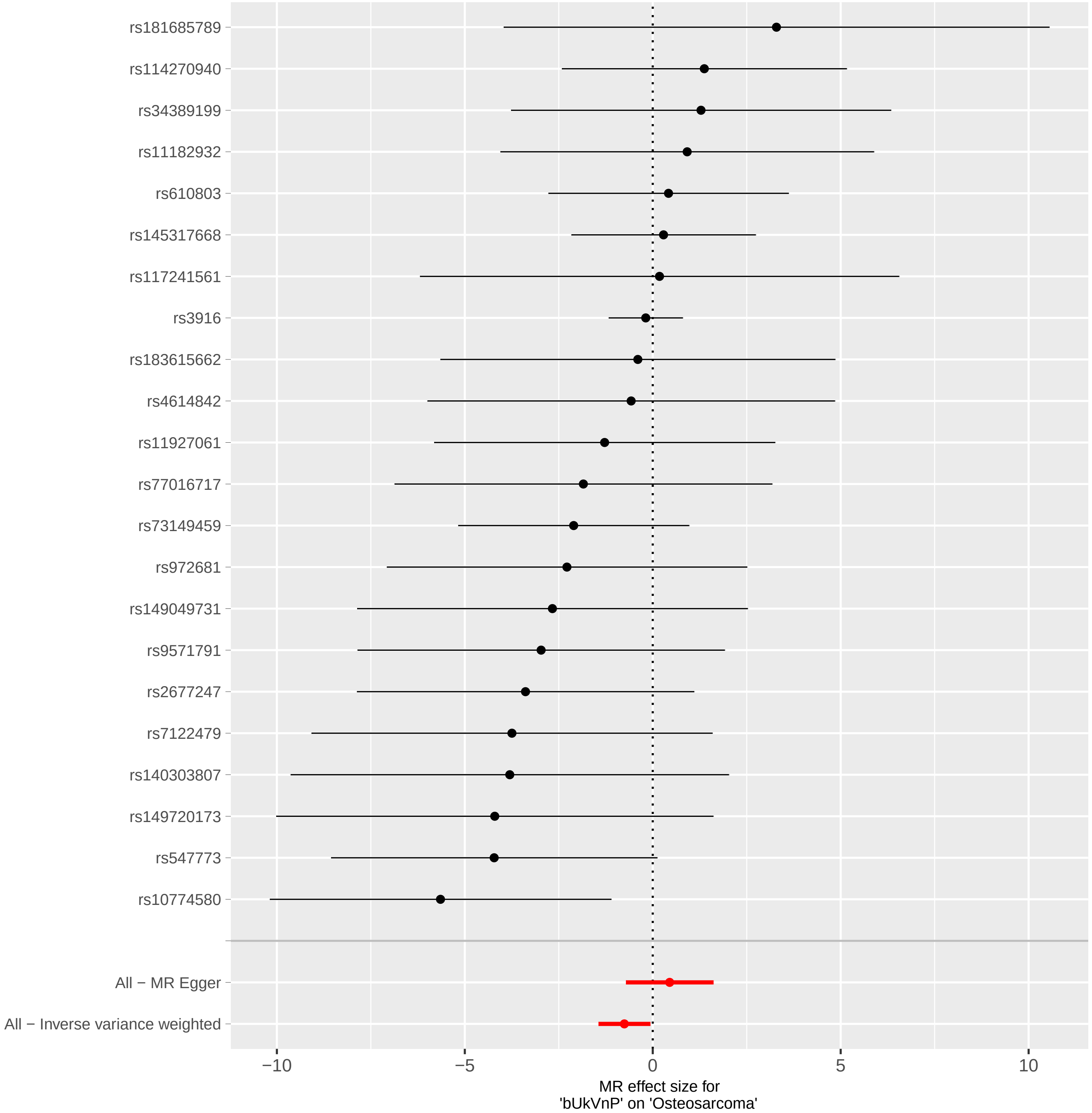

rs181685789
rs114270940
rs34389199
rs11182932
rs610803
rs145317668
rs117241561
rs3916
rs183615662
rs4614842
rs11927061
rs77016717
rs73149459
rs972681
rs149049731
rs9571791
rs2677247
rs7122479
rs140303807
rs149720173
rs547773
rs10774580
All − MR Egger
All − Inverse variance weighted
−10
−5
0
5
10
MR effect size for
'bUkVnP' on 'Osteosarcoma'

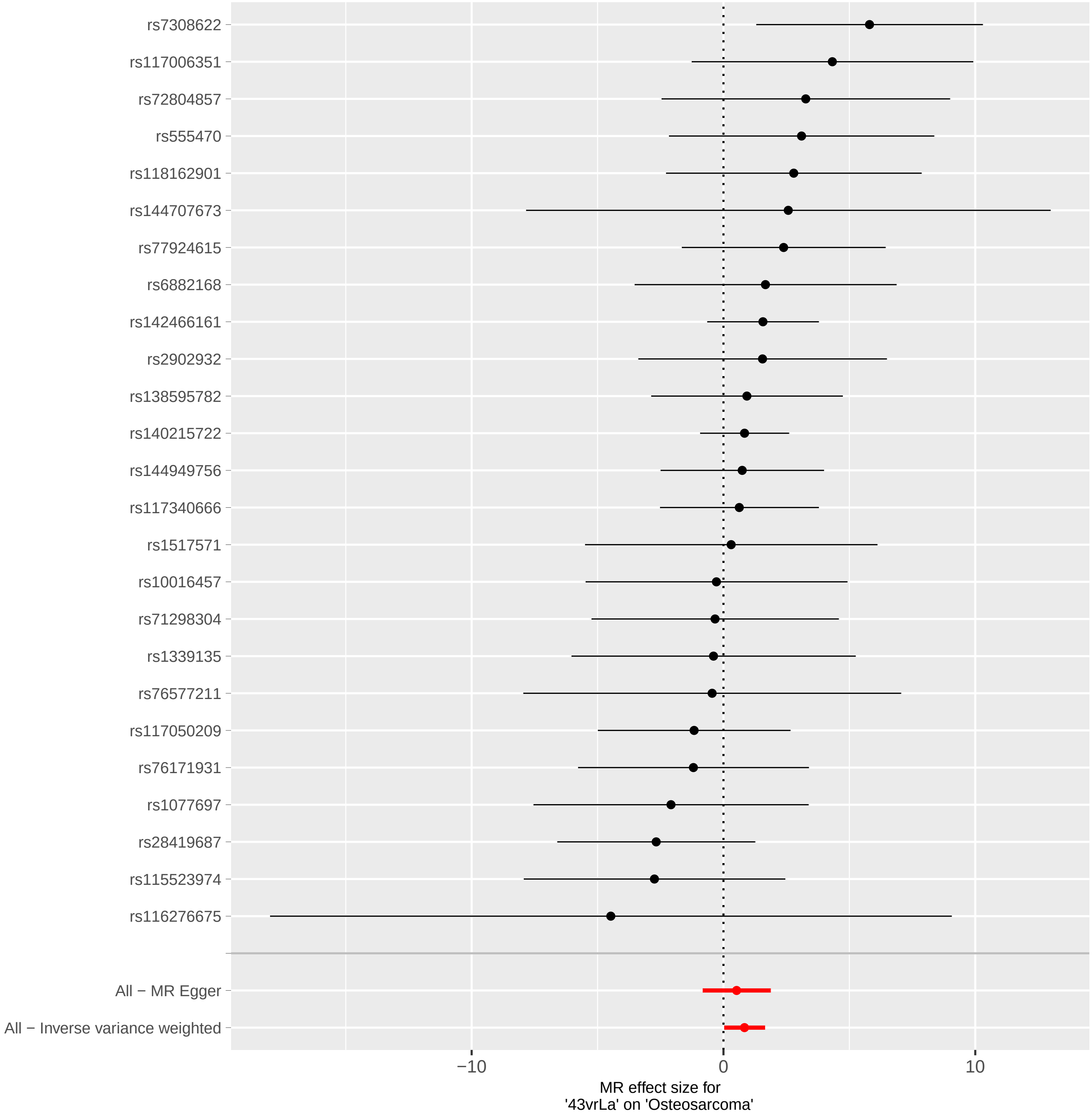

rs7308622
rs117006351
rs72804857
rs555470
rs118162901
rs144707673
rs77924615
rs6882168
rs142466161
rs2902932
rs138595782
rs140215722
rs144949756
rs117340666
rs1517571
rs10016457
rs71298304
rs1339135
rs76577211
rs117050209
rs76171931
rs1077697
rs28419687
rs115523974
rs116276675
All − MR Egger
All − Inverse variance weighted
−10
0
10
MR effect size for
'43vrLa' on 'Osteosarcoma'

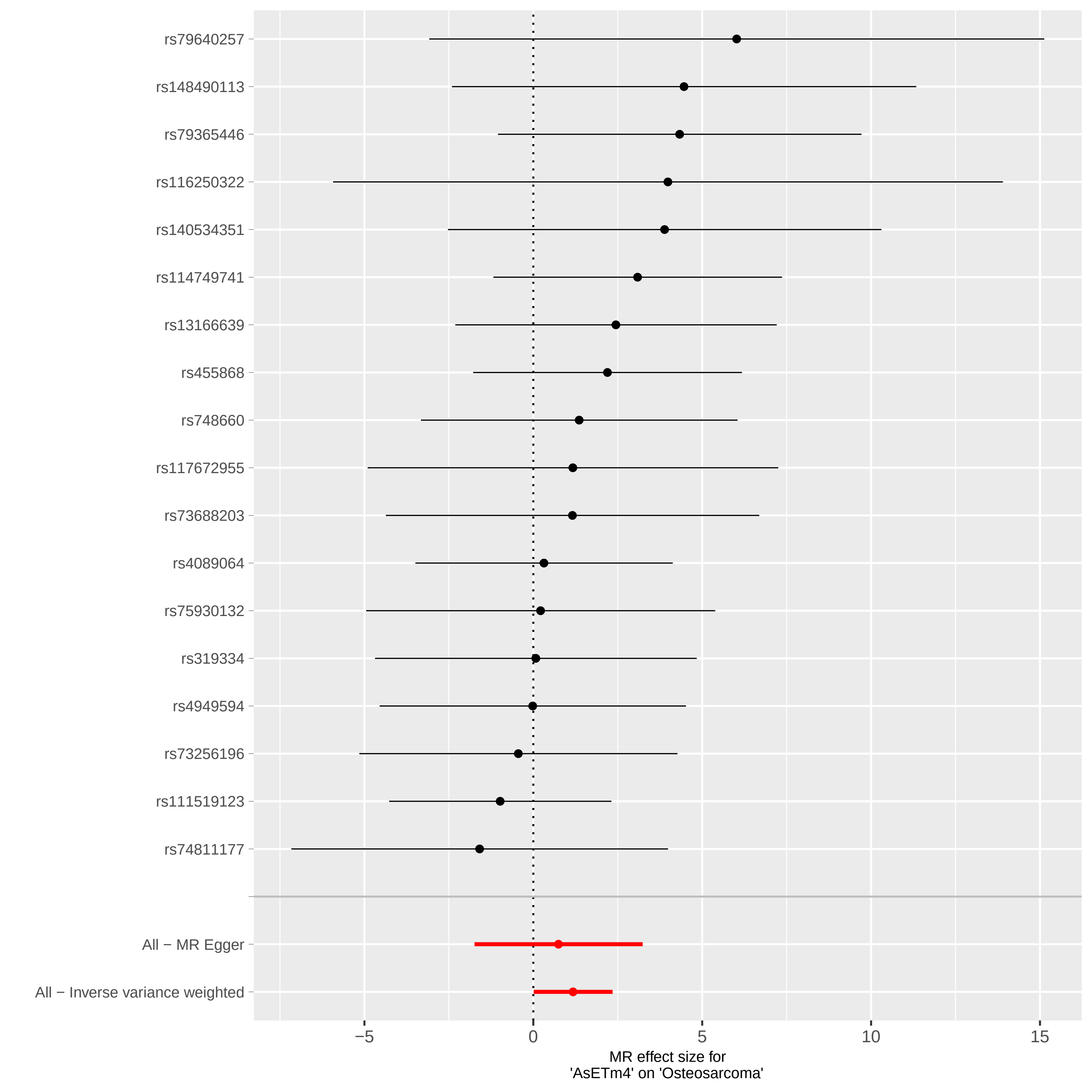
rs79640257
rs148490113
rs79365446
rs116250322
rs140534351
rs114749741
rs13166639
rs455868
rs748660
rs117672955
rs73688203
rs4089064
rs75930132
rs319334
rs4949594
rs73256196
rs111519123
rs74811177
All − MR Egger
All − Inverse variance weighted
−5
0
5
10
15
MR effect size for
'AsETm4' on 'Osteosarcoma'

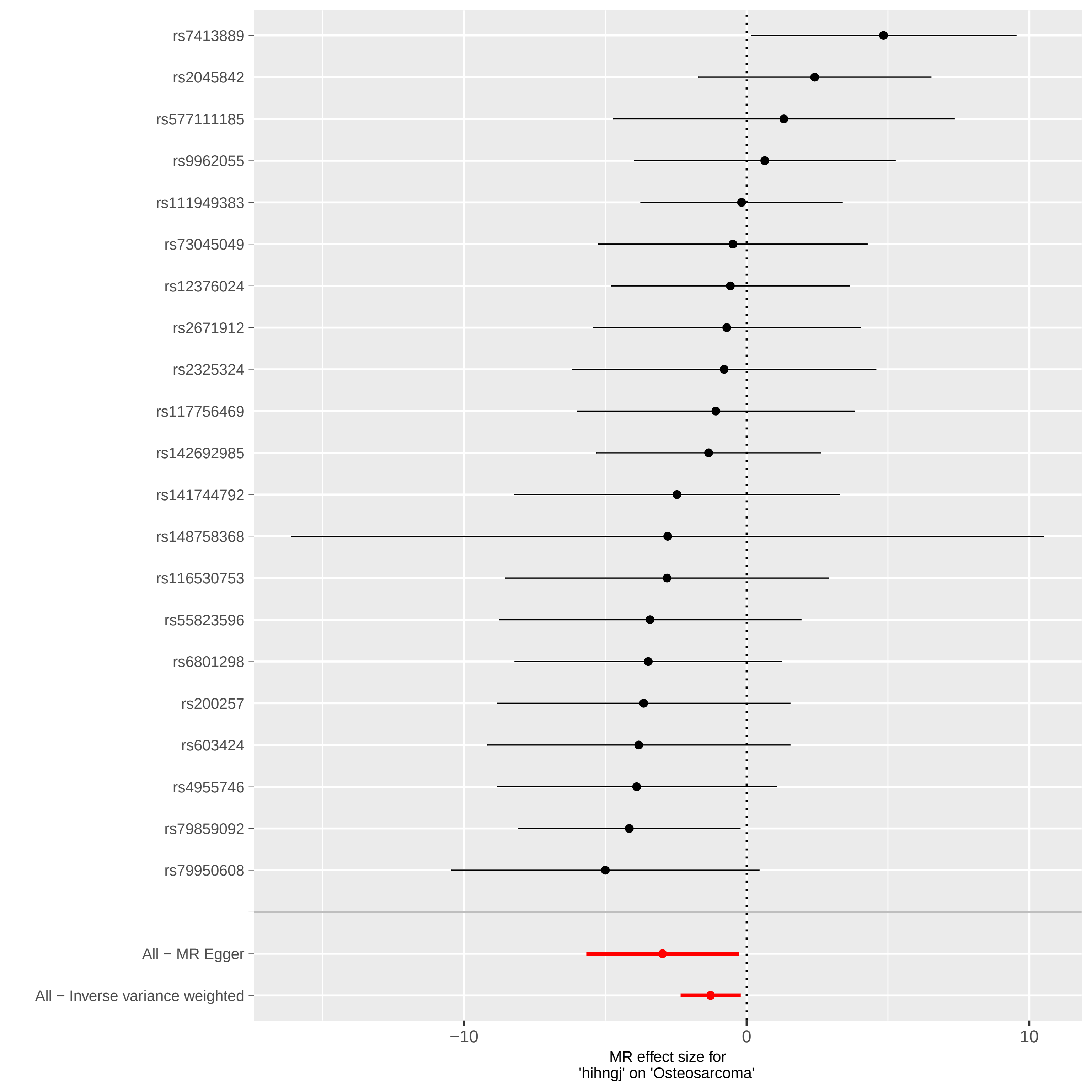
rs7413889
rs2045842
rs577111185
rs9962055
rs111949383
rs73045049
rs12376024
rs2671912
rs2325324
rs117756469
rs142692985
rs141744792
rs148758368
rs116530753
rs55823596
rs6801298
rs200257
rs603424
rs4955746
rs79859092
rs79950608
All − MR Egger
All − Inverse variance weighted
−10
0
10
MR effect size for
'hihngj' on 'Osteosarcoma'

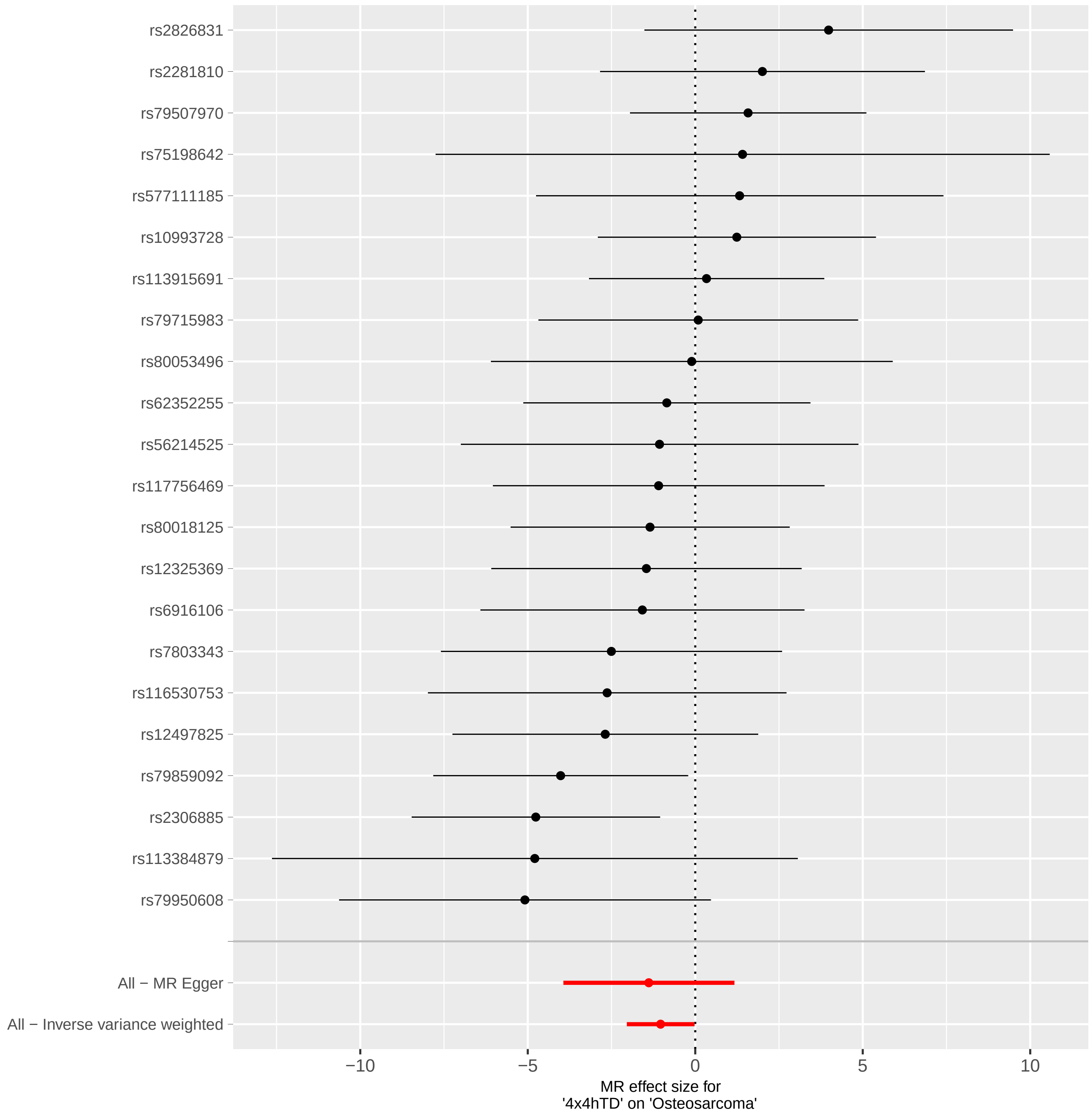

rs2826831
rs2281810
rs79507970
rs75198642
rs577111185
rs10993728
rs113915691
rs79715983
rs80053496
rs62352255
rs56214525
rs117756469
rs80018125
rs12325369
rs6916106
rs7803343
rs116530753
rs12497825
rs79859092
rs2306885
rs113384879
rs79950608
All − MR Egger
All − Inverse variance weighted
−10
−5
0
5
10
MR effect size for
'4x4hTD' on 'Osteosarcoma'

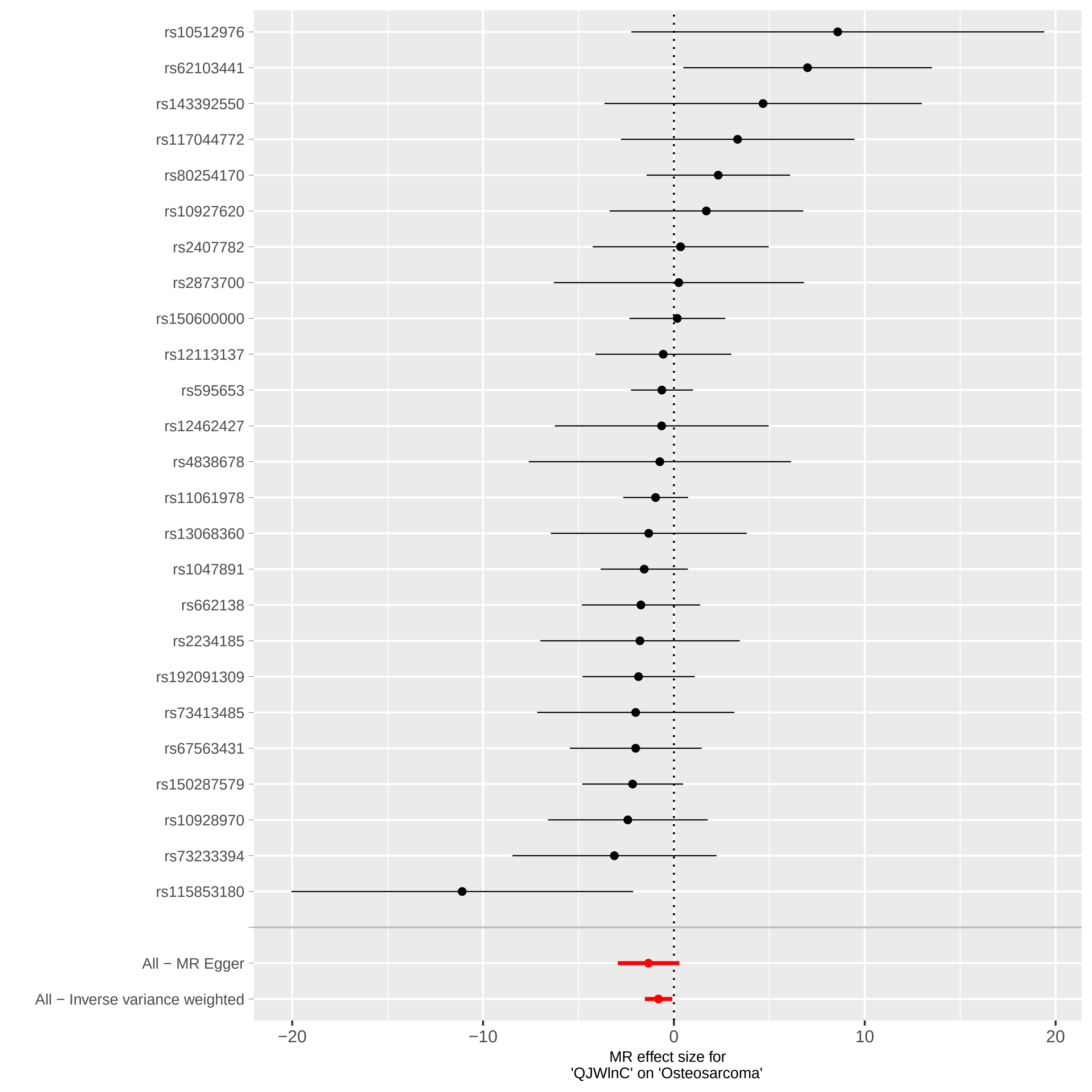
rs10512976
rs62103441
rs143392550
rs117044772
rs80254170
rs10927620
rs2407782
rs2873700
rs150600000
rs12113137
rs595653
rs12462427
rs4838678
rs11061978
rs13068360
rs1047891
rs662138
rs2234185
rs192091309
rs73413485
rs67563431
rs150287579
rs10928970
rs73233394
rs115853180
All − MR Egger
All − Inverse variance weighted
−20
−10
0
10
20
MR effect size for
'QJWlnC' on 'Osteosarcoma'

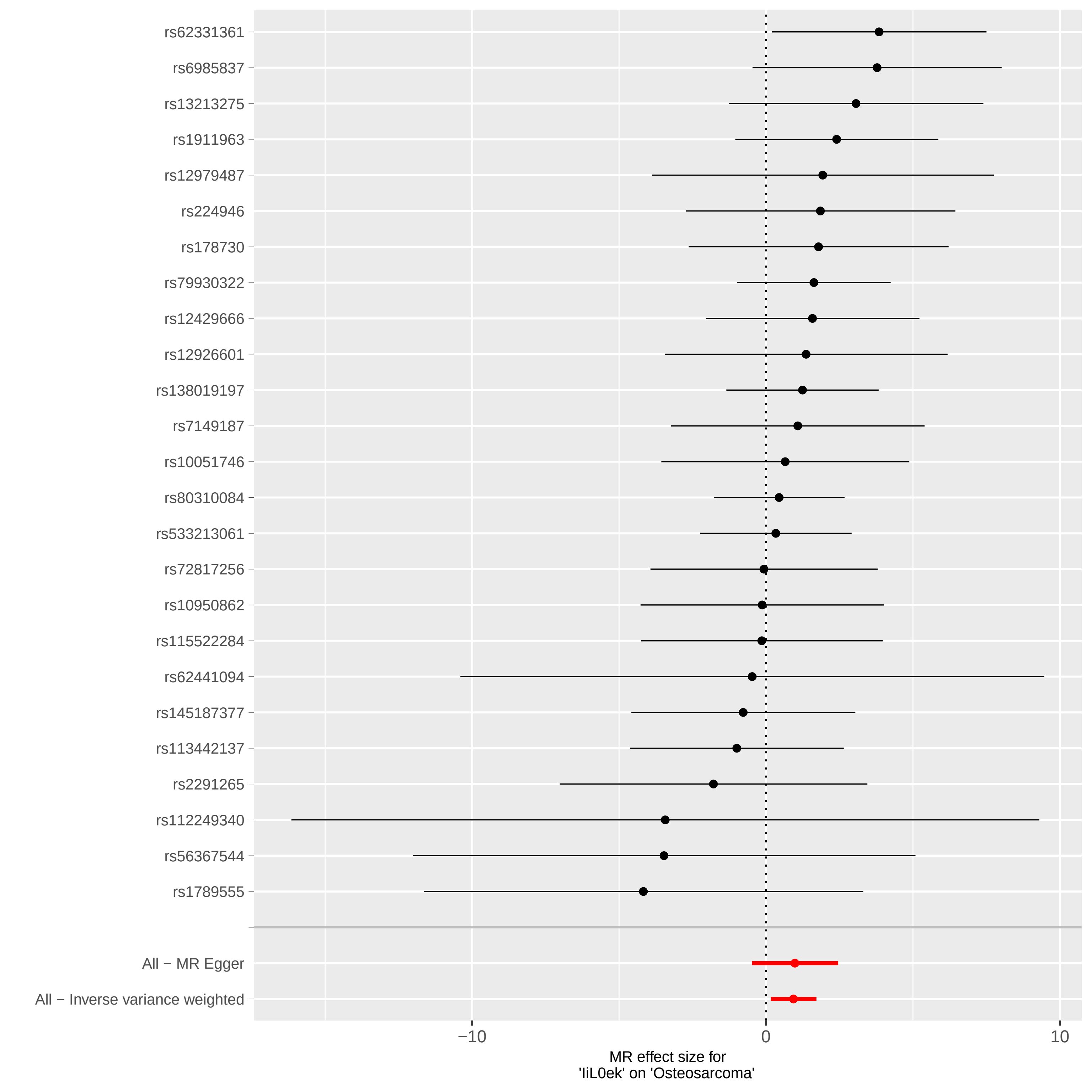
rs62331361
rs6985837
rs13213275
rs1911963
rs12979487
rs224946
rs178730
rs79930322
rs12429666
rs12926601
rs138019197
rs7149187
rs10051746
rs80310084
rs533213061
rs72817256
rs10950862
rs115522284
rs62441094
rs145187377
rs113442137
rs2291265
rs112249340
rs56367544
rs1789555
All − MR Egger
All − Inverse variance weighted
−10
0
10
MR effect size for
'IiL0ek' on 'Osteosarcoma'

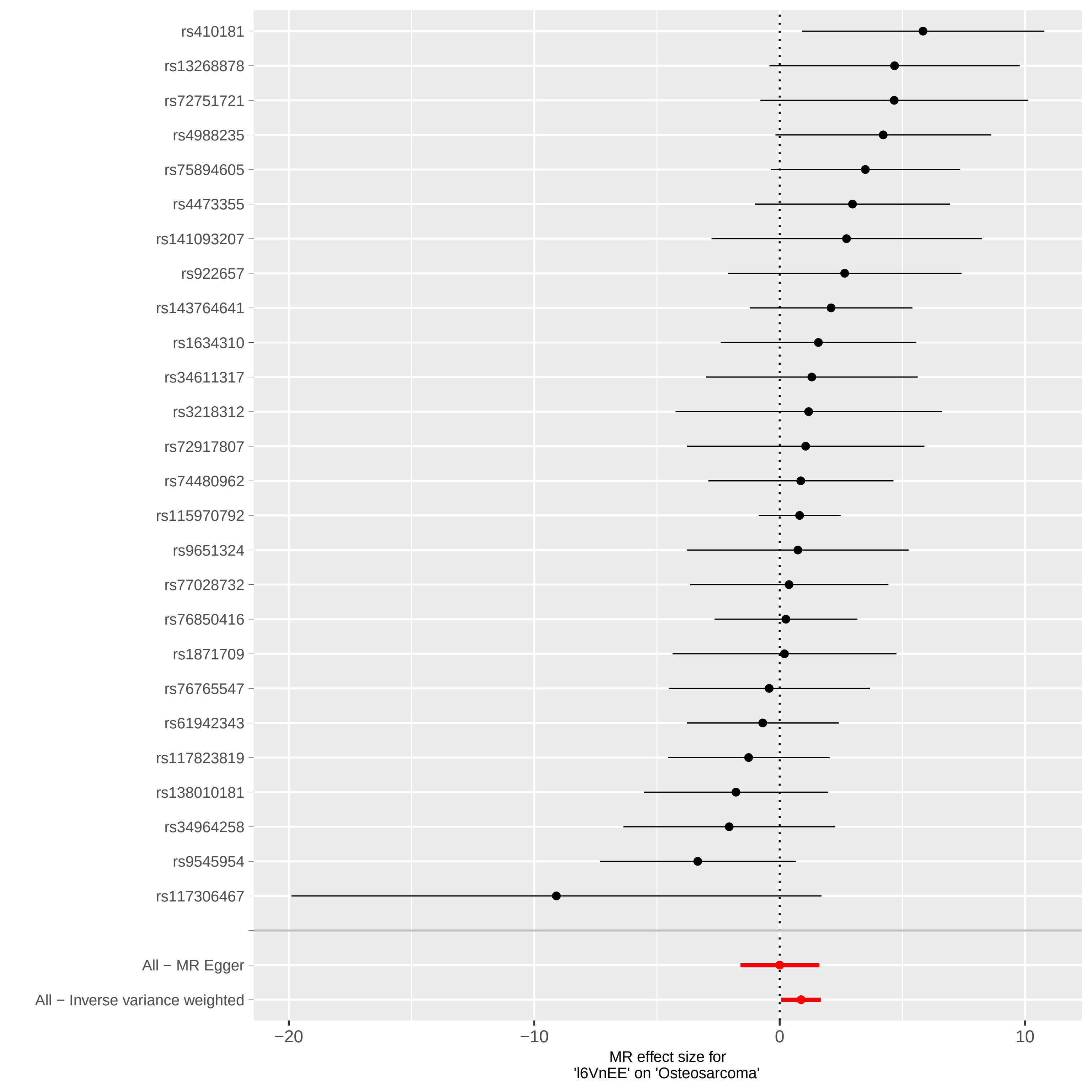
rs410181
rs13268878
rs72751721
rs4988235
rs75894605
rs4473355
rs141093207
rs922657
rs143764641
rs1634310
rs34611317
rs3218312
rs72917807
rs74480962
rs115970792
rs9651324
rs77028732
rs76850416
rs1871709
rs76765547
rs61942343
rs117823819
rs138010181
rs34964258
rs9545954
rs117306467
All − MR Egger
All − Inverse variance weighted
−20
−10
0
10
MR effect size for
'l6VnEE' on 'Osteosarcoma'

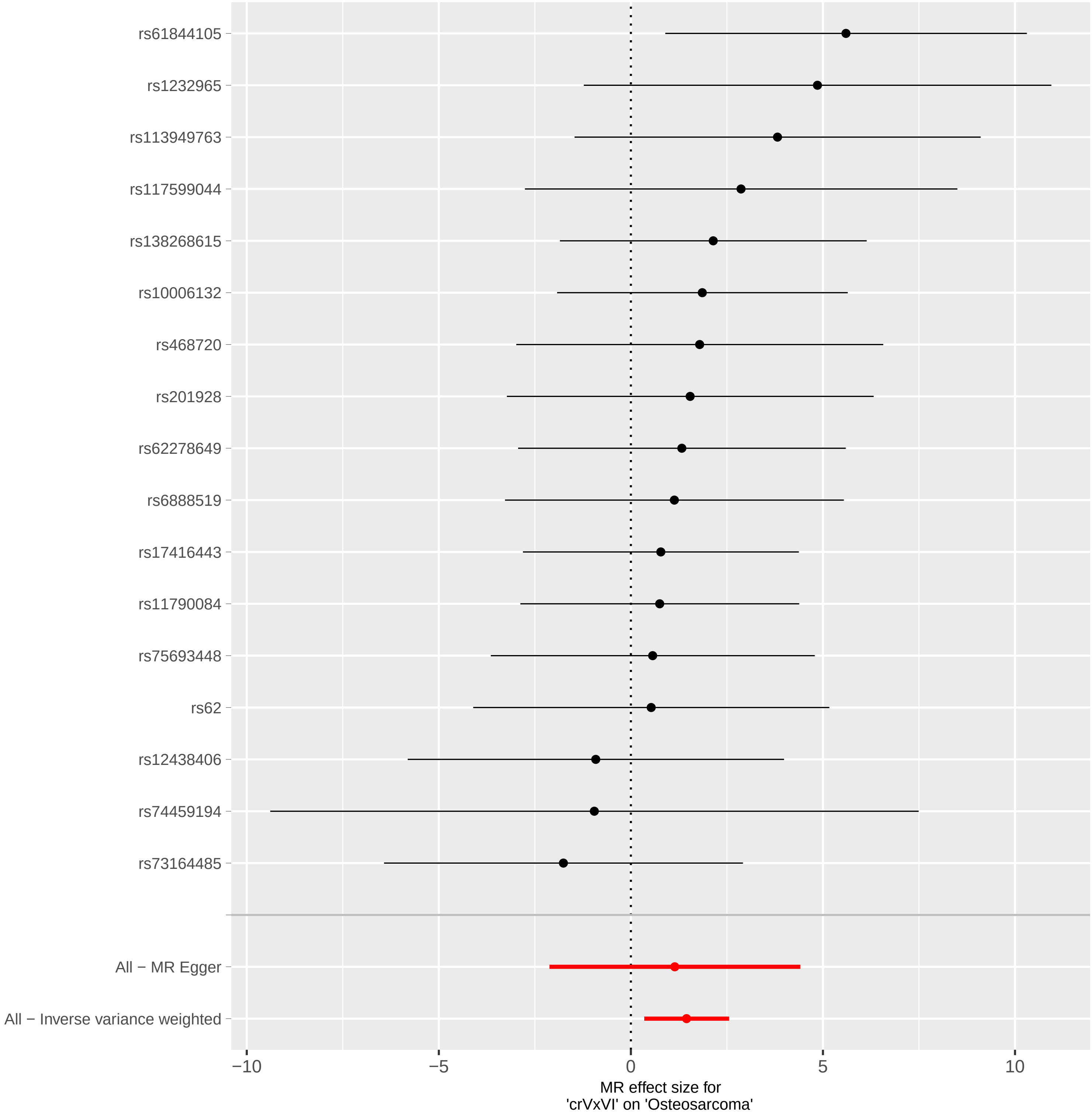
rs61844105
rs1232965
rs113949763
rs117599044
rs138268615
rs10006132
rs468720
rs201928
rs62278649
rs6888519
rs17416443
rs11790084
rs75693448
rs62
rs12438406
rs74459194
rs73164485
All − MR Egger
All − Inverse variance weighted
−10
−5
0
5
10
MR effect size for
'crVxVI' on 'Osteosarcoma'

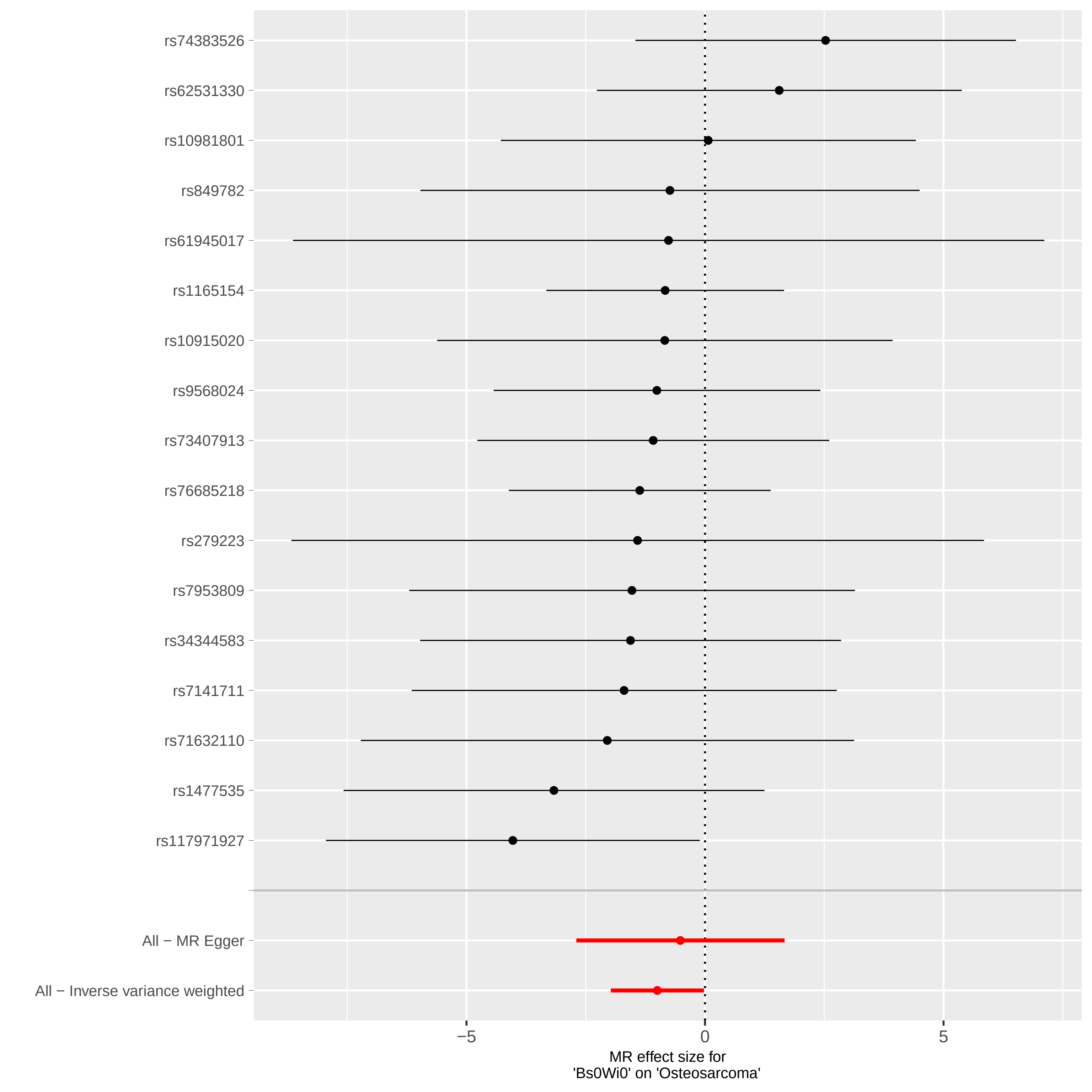
rs74383526
rs62531330
rs10981801
rs849782
rs61945017
rs1165154
rs10915020
rs9568024
rs73407913
rs76685218
rs279223
rs7953809
rs34344583
rs7141711
rs71632110
rs1477535
rs117971927
All − MR Egger
All − Inverse variance weighted
−5
0
5
MR effect size for
'Bs0Wi0' on 'Osteosarcoma'

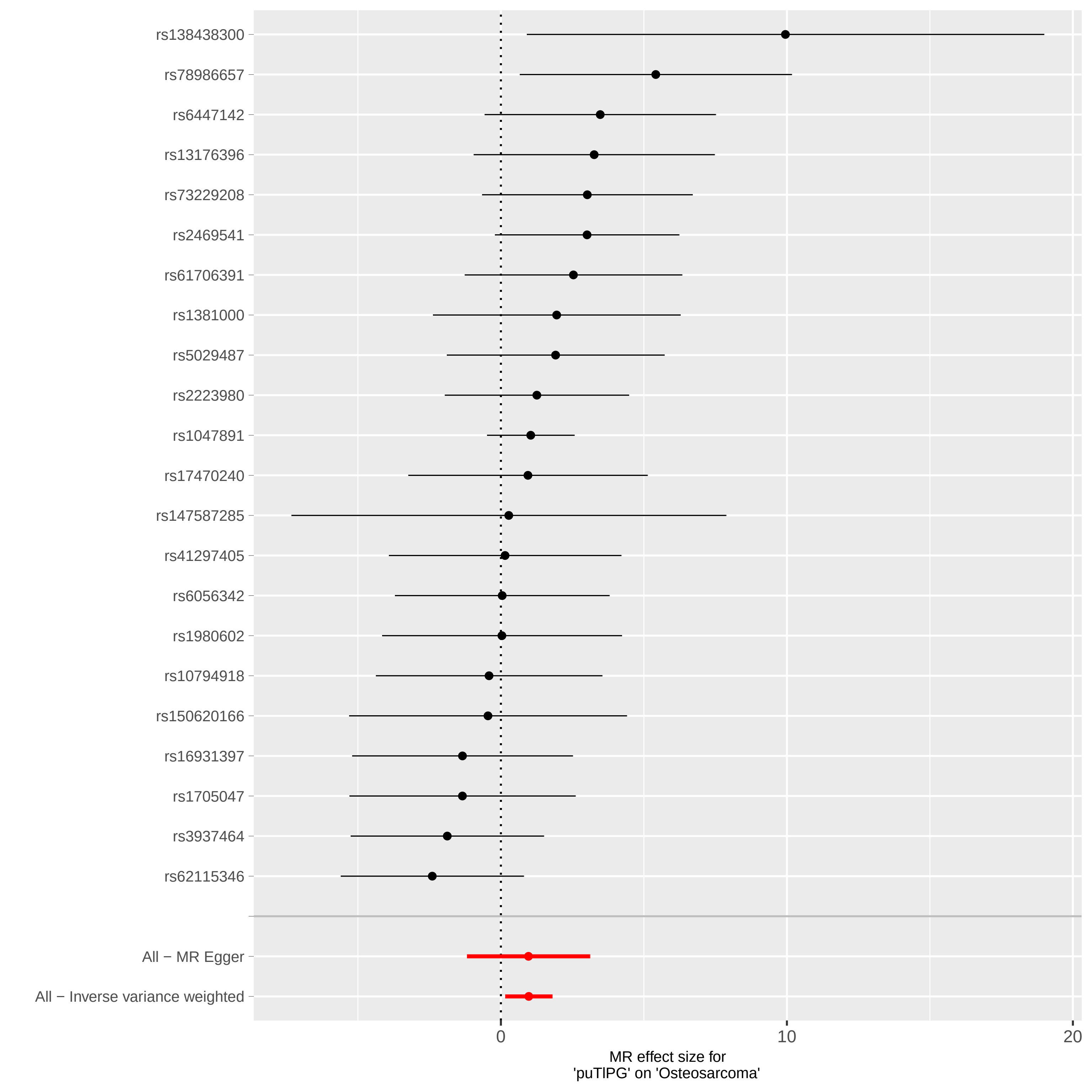

rs138438300
rs78986657
rs6447142
rs13176396
rs73229208
rs2469541
rs61706391
rs1381000
rs5029487
rs2223980
rs1047891
rs17470240
rs147587285
rs41297405
rs6056342
rs1980602
rs10794918
rs150620166
rs16931397
rs1705047
rs3937464
rs62115346
All − MR Egger
All − Inverse variance weighted
0
10
20
MR effect size for
'puTIPG' on 'Osteosarcoma'

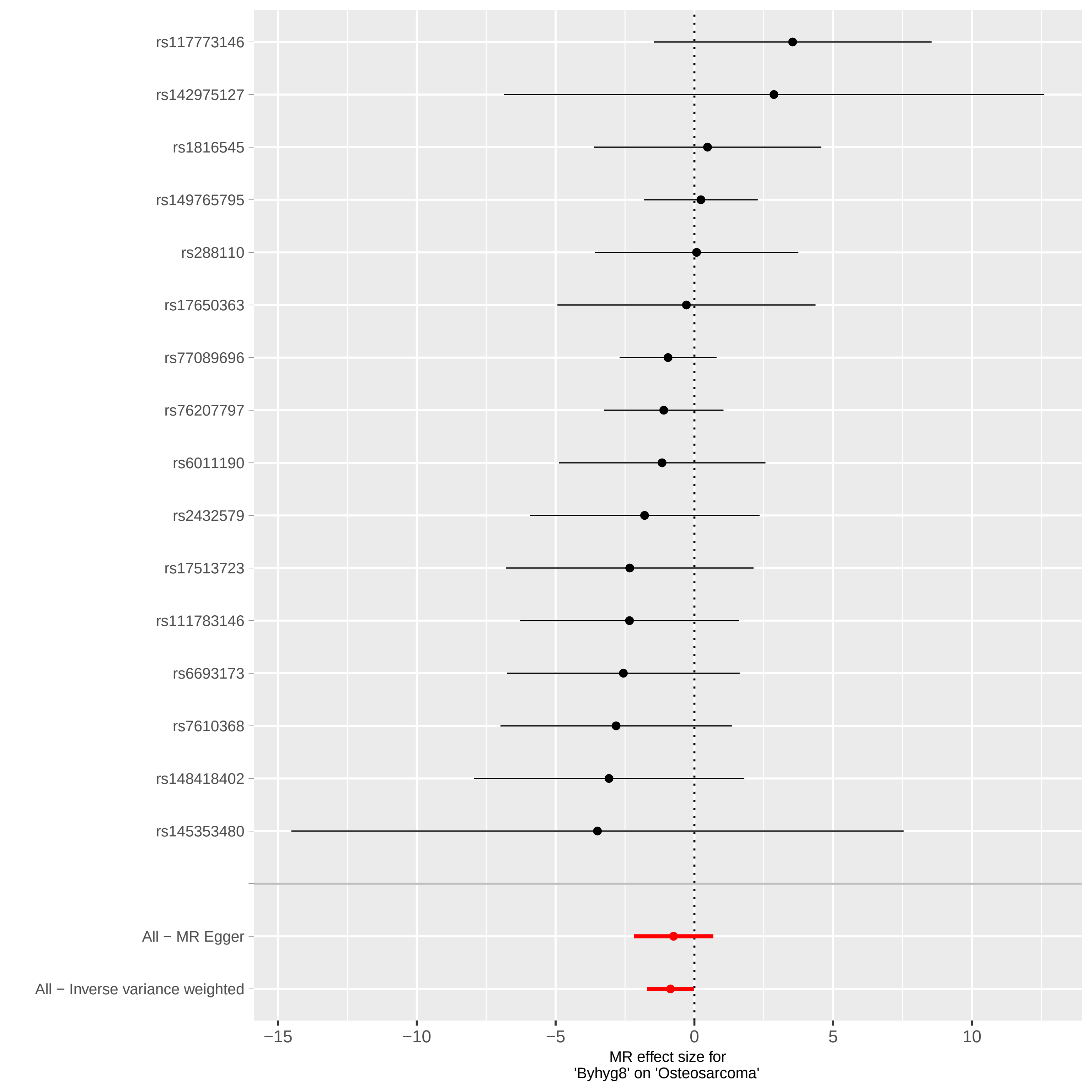
rs117773146
rs142975127
rs1816545
rs149765795
rs288110
rs17650363
rs77089696
rs76207797
rs6011190
rs2432579
rs17513723
rs111783146
rs6693173
rs7610368
rs148418402
rs145353480
All − MR Egger
All − Inverse variance weighted
−15
−10
−5
0
5
10
MR effect size for
'Byhyg8' on 'Osteosarcoma'

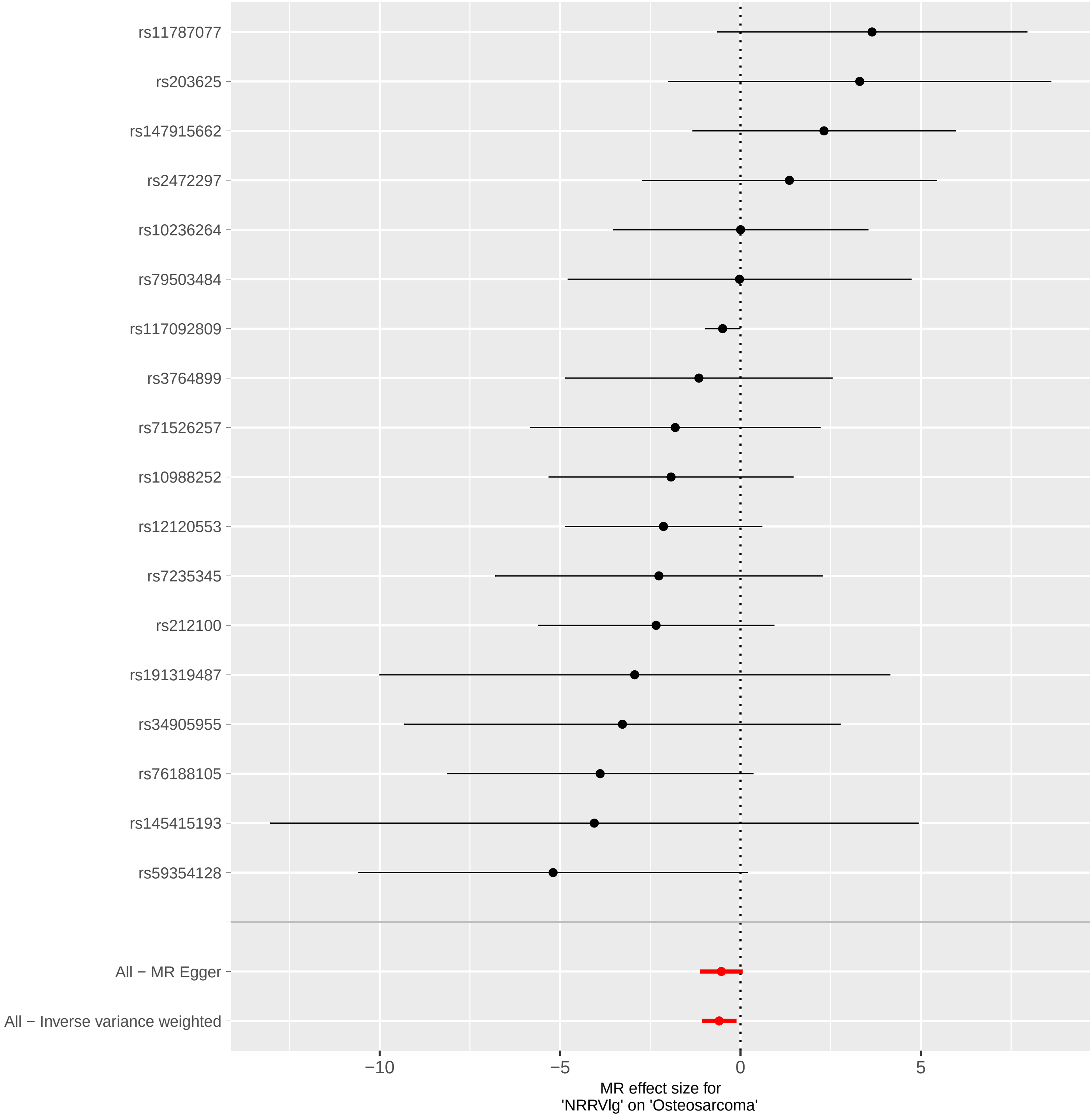
rs11787077
rs203625
rs147915662
rs2472297
rs10236264
rs79503484
rs117092809
rs3764899
rs71526257
rs10988252
rs12120553
rs7235345
rs212100
rs191319487
rs34905955
rs76188105
rs145415193
rs59354128
All − MR Egger
All − Inverse variance weighted
−10
−5
0
5
MR effect size for
'NRRVlg' on 'Osteosarcoma'

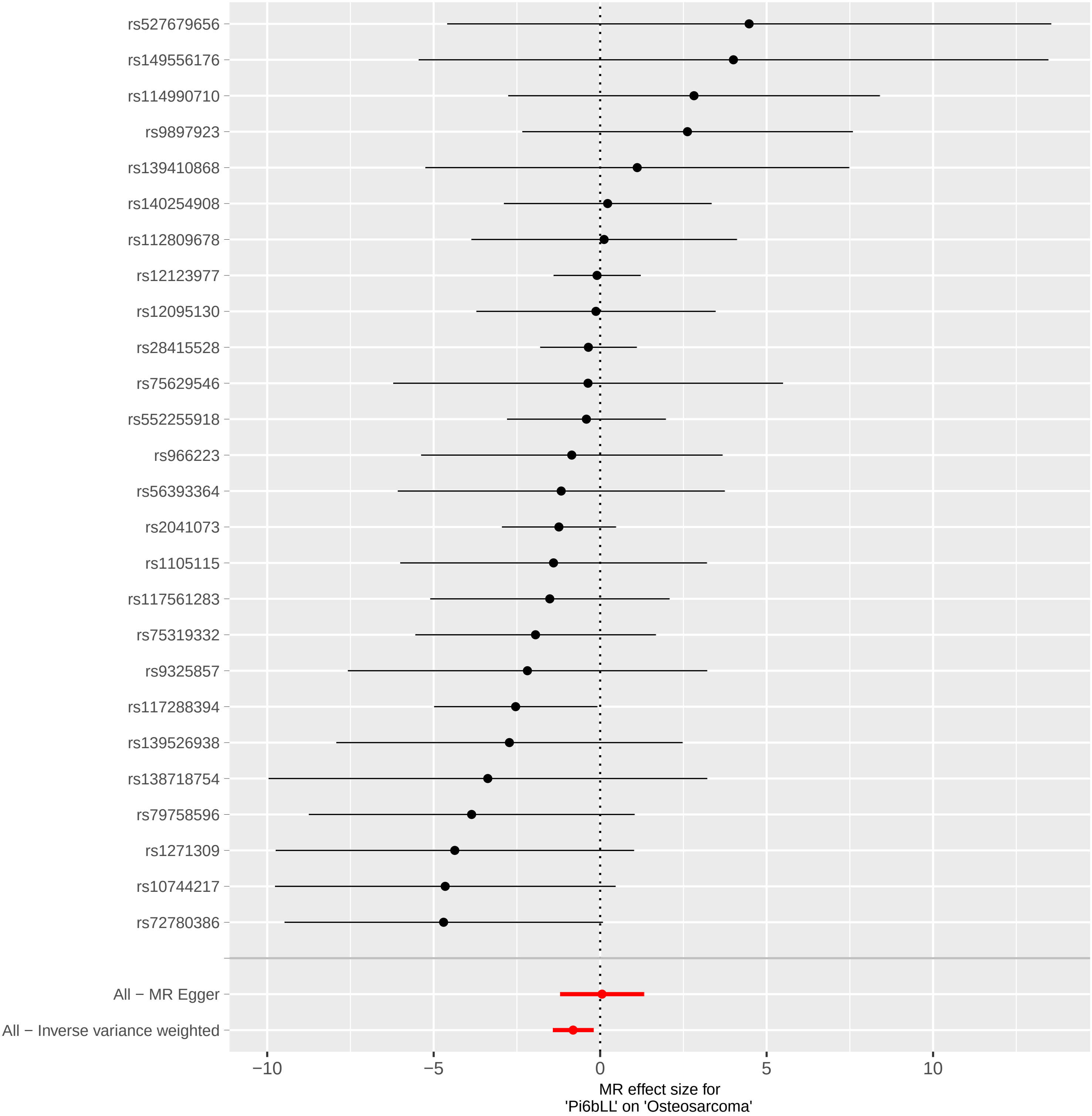
rs527679656
rs149556176
rs114990710
rs9897923
rs139410868
rs140254908
rs112809678
rs12123977
rs12095130
rs28415528
rs75629546
rs552255918
rs966223
rs56393364
rs2041073
rs1105115
rs117561283
rs75319332
rs9325857
rs117288394
rs139526938
rs138718754
rs79758596
rs1271309
rs10744217
rs72780386
All − MR Egger
All − Inverse variance weighted
−10
−5
0
5
10
MR effect size for
'Pi6bLL' on 'Osteosarcoma'

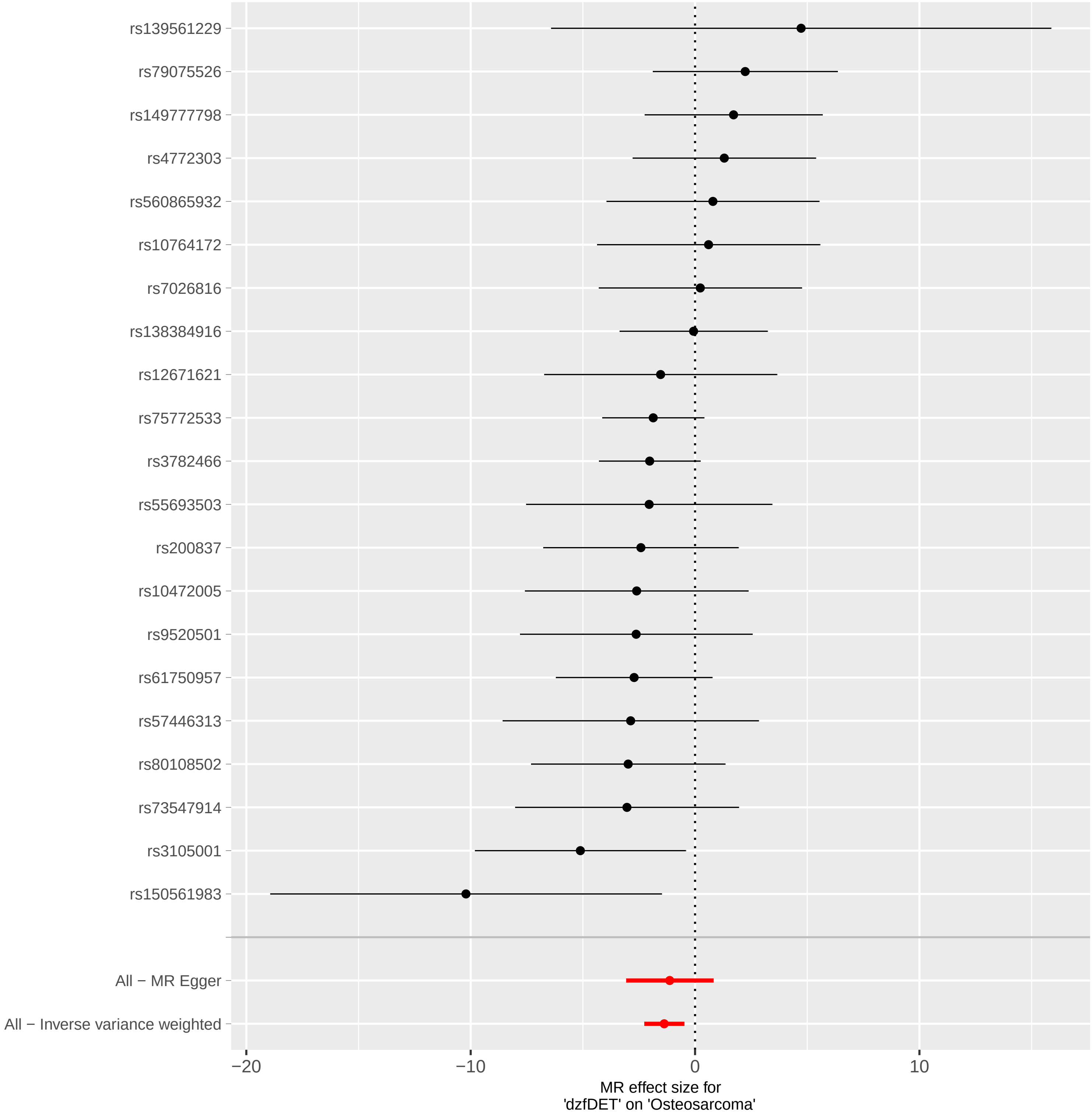

rs139561229
rs79075526
rs149777798
rs4772303
rs560865932
rs10764172
rs7026816
rs138384916
rs12671621
rs75772533
rs3782466
rs55693503
rs200837
rs10472005
rs9520501
rs61750957
rs57446313
rs80108502
rs73547914
rs3105001
rs150561983
All − MR Egger
All − Inverse variance weighted
−20
−10
0
10
MR effect size for
'dzfDET' on 'Osteosarcoma'

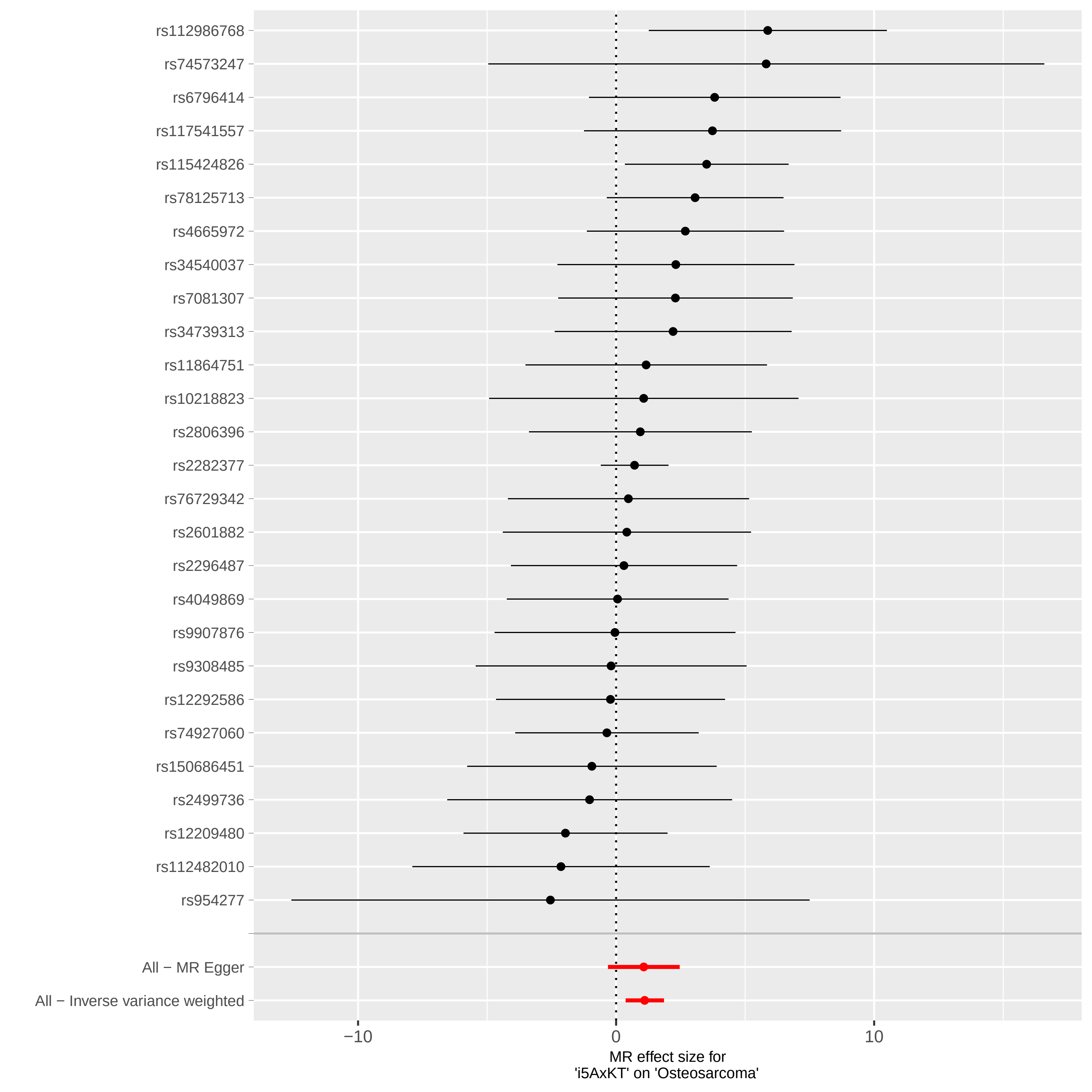

rs112986768
rs74573247
rs6796414
rs117541557
rs115424826
rs78125713
rs4665972
rs34540037
rs7081307
rs34739313
rs11864751
rs10218823
rs2806396
rs2282377
rs76729342
rs2601882
rs2296487
rs4049869
rs9907876
rs9308485
rs12292586
rs74927060
rs150686451
rs2499736
rs12209480
rs112482010
rs954277
All − MR Egger
All − Inverse variance weighted
−10
0
10
MR effect size for
'i5AxKT' on 'Osteosarcoma'

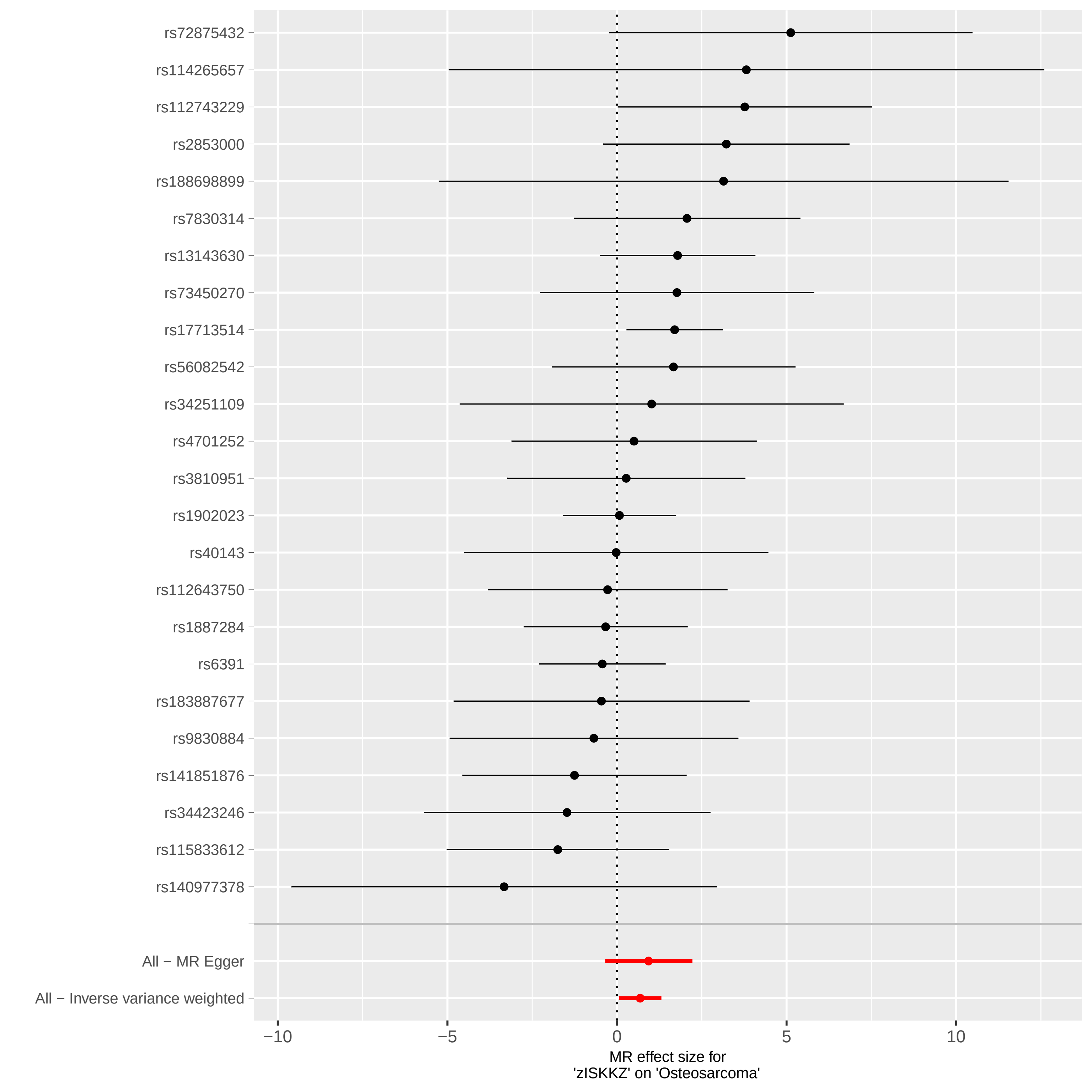

rs72875432
rs114265657
rs112743229
rs2853000
rs188698899
rs7830314
rs13143630
rs73450270
rs17713514
rs56082542
rs34251109
rs4701252
rs3810951
rs1902023
rs40143
rs112643750
rs1887284
rs6391
rs183887677
rs9830884
rs141851876
rs34423246
rs115833612
rs140977378
All − MR Egger
All − Inverse variance weighted
−10
−5
0
5
10
MR effect size for
'zISKKZ' on 'Osteosarcoma'

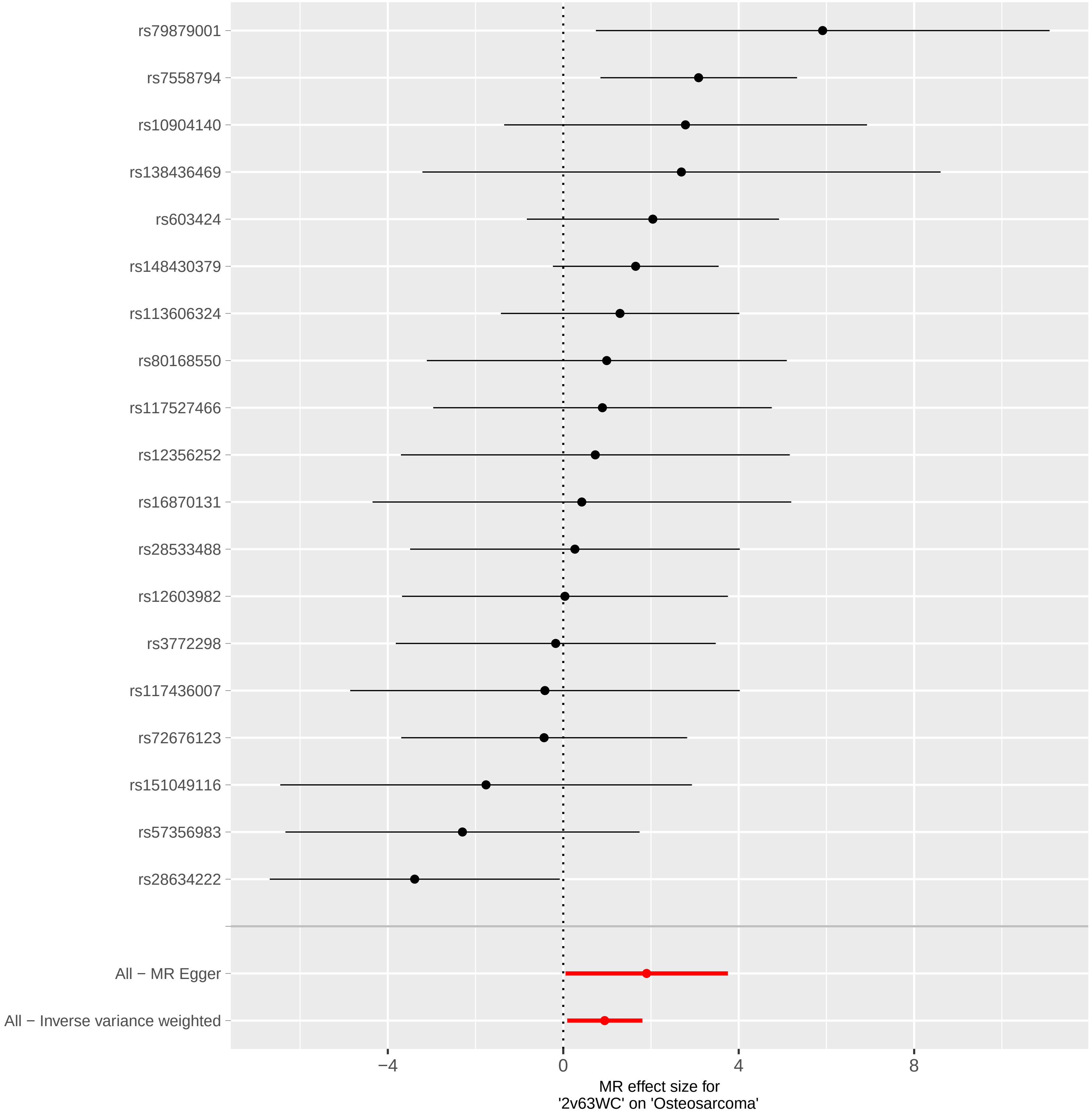
rs79879001
rs7558794
rs10904140
rs138436469
rs603424
rs148430379
rs113606324
rs80168550
rs117527466
rs12356252
rs16870131
rs28533488
rs12603982
rs3772298
rs117436007
rs72676123
rs151049116
rs57356983
rs28634222
All − MR Egger
All − Inverse variance weighted
−4
0
4
8
MR effect size for
'2v63WC' on 'Osteosarcoma'

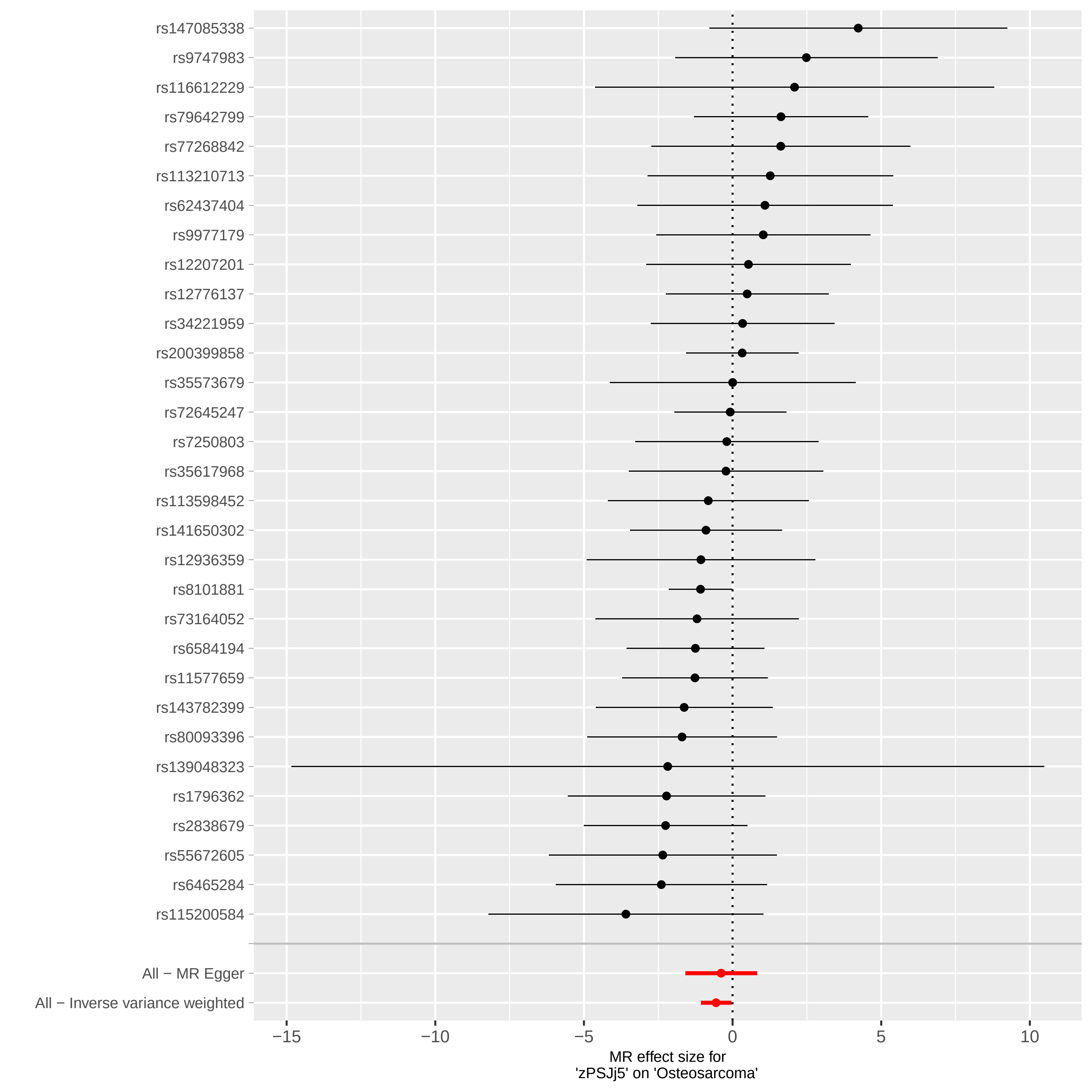

rs147085338
rs9747983
rs116612229
rs79642799
rs77268842
rs113210713
rs62437404
rs9977179
rs12207201
rs12776137
rs34221959
rs200399858
rs35573679
rs72645247
rs7250803
rs35617968
rs113598452
rs141650302
rs12936359
rs8101881
rs73164052
rs6584194
rs11577659
rs143782399
rs80093396
rs139048323
rs1796362
rs2838679
rs55672605
rs6465284
rs115200584
All − MR Egger
All − Inverse variance weighted
−15
−10
−5
0
5
10
MR effect size for
'zPSJj5' on 'Osteosarcoma'

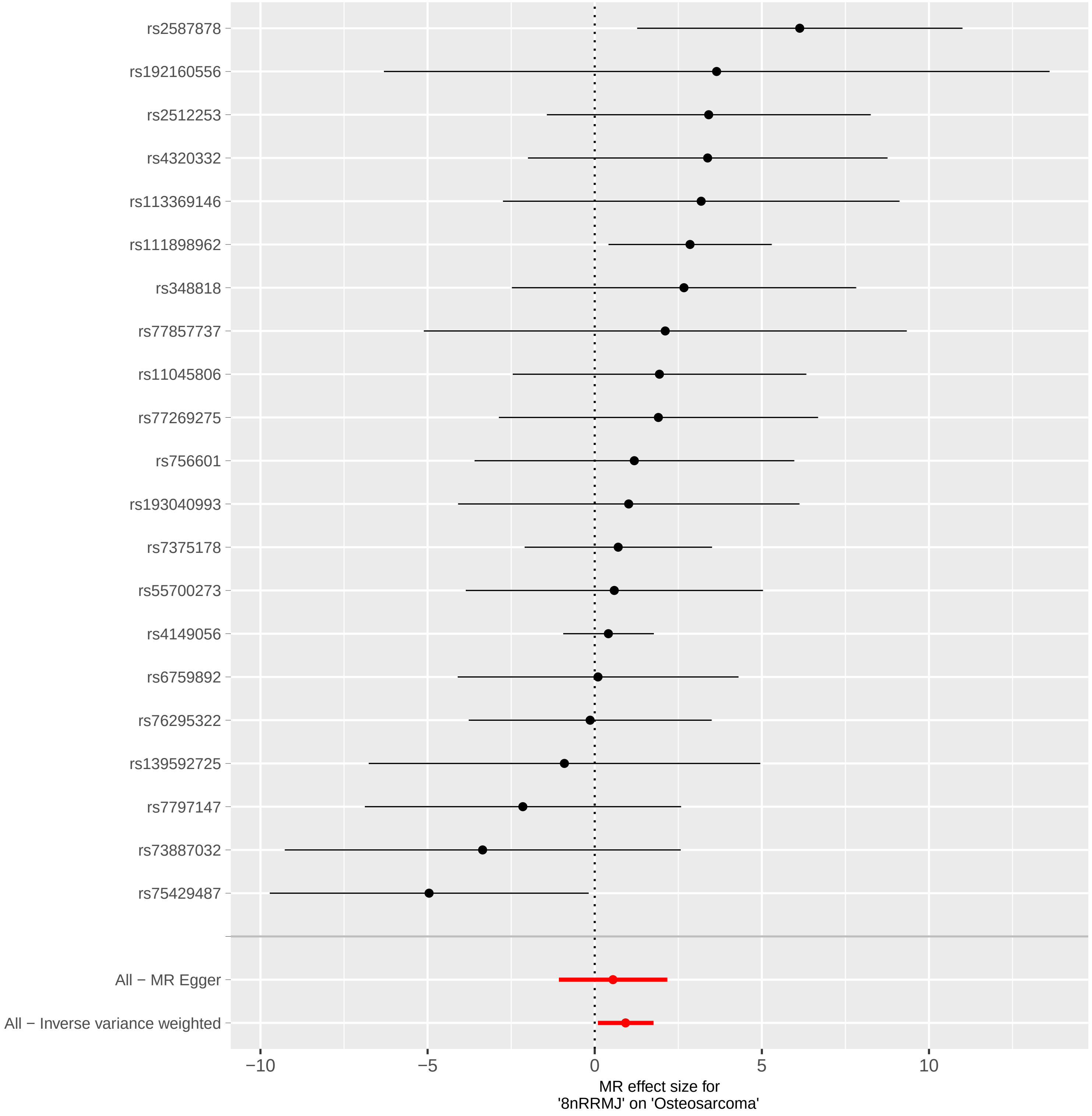

rs2587878
rs192160556
rs2512253
rs4320332
rs113369146
rs111898962
rs348818
rs77857737
rs11045806
rs77269275
rs756601
rs193040993
rs7375178
rs55700273
rs4149056
rs6759892
rs76295322
rs139592725
rs7797147
rs73887032
rs75429487
All − MR Egger
All − Inverse variance weighted
−10
−5
0
5
10
MR effect size for
'8nRRMJ' on 'Osteosarcoma'

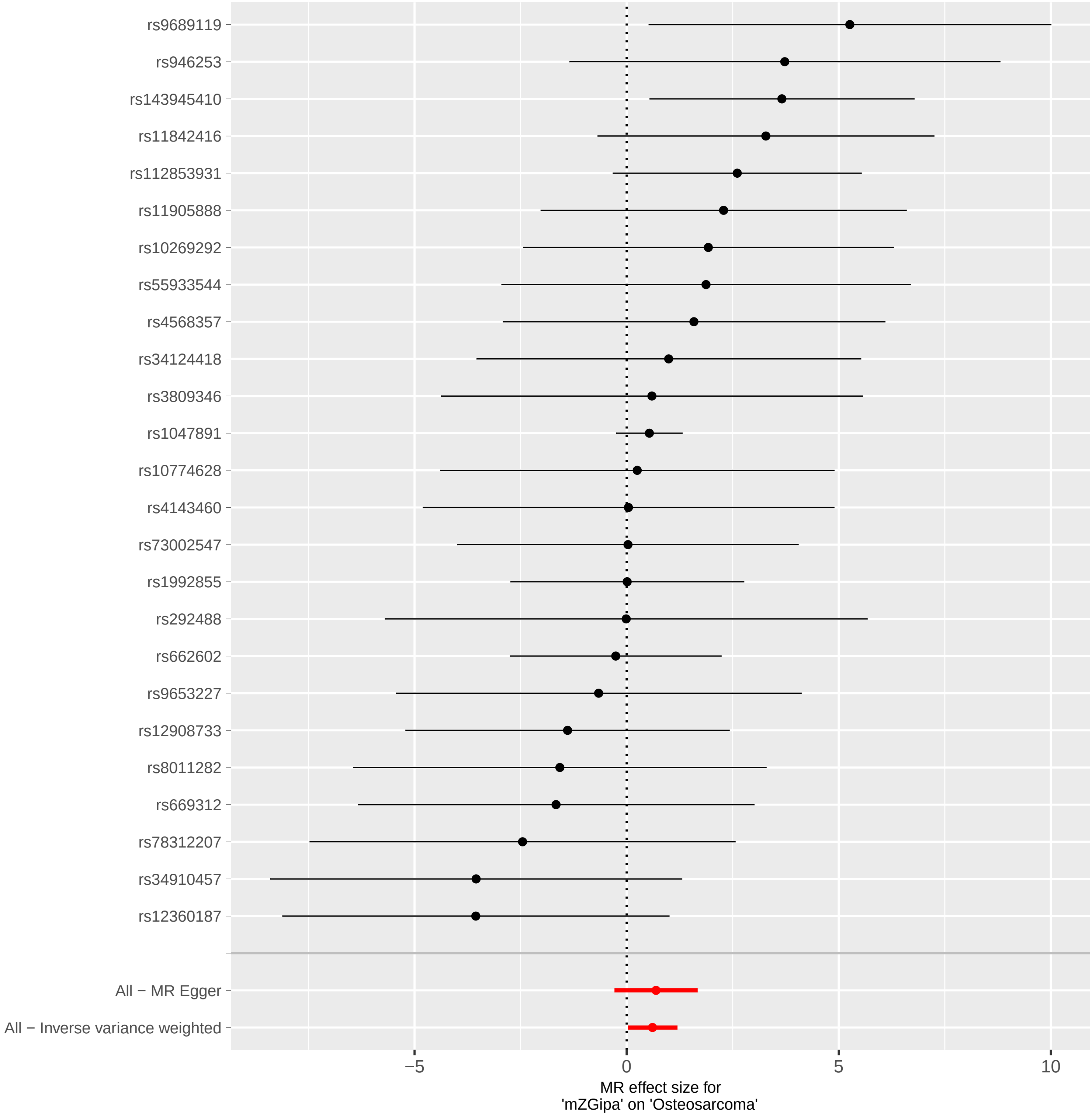
rs9689119
rs946253
rs143945410
rs11842416
rs112853931
rs11905888
rs10269292
rs55933544
rs4568357
rs34124418
rs3809346
rs1047891
rs10774628
rs4143460
rs73002547
rs1992855
rs292488
rs662602
rs9653227
rs12908733
rs8011282
rs669312
rs78312207
rs34910457
rs12360187
All − MR Egger
All − Inverse variance weighted
−5
0
5
10
MR effect size for
'mZGipa' on 'Osteosarcoma'

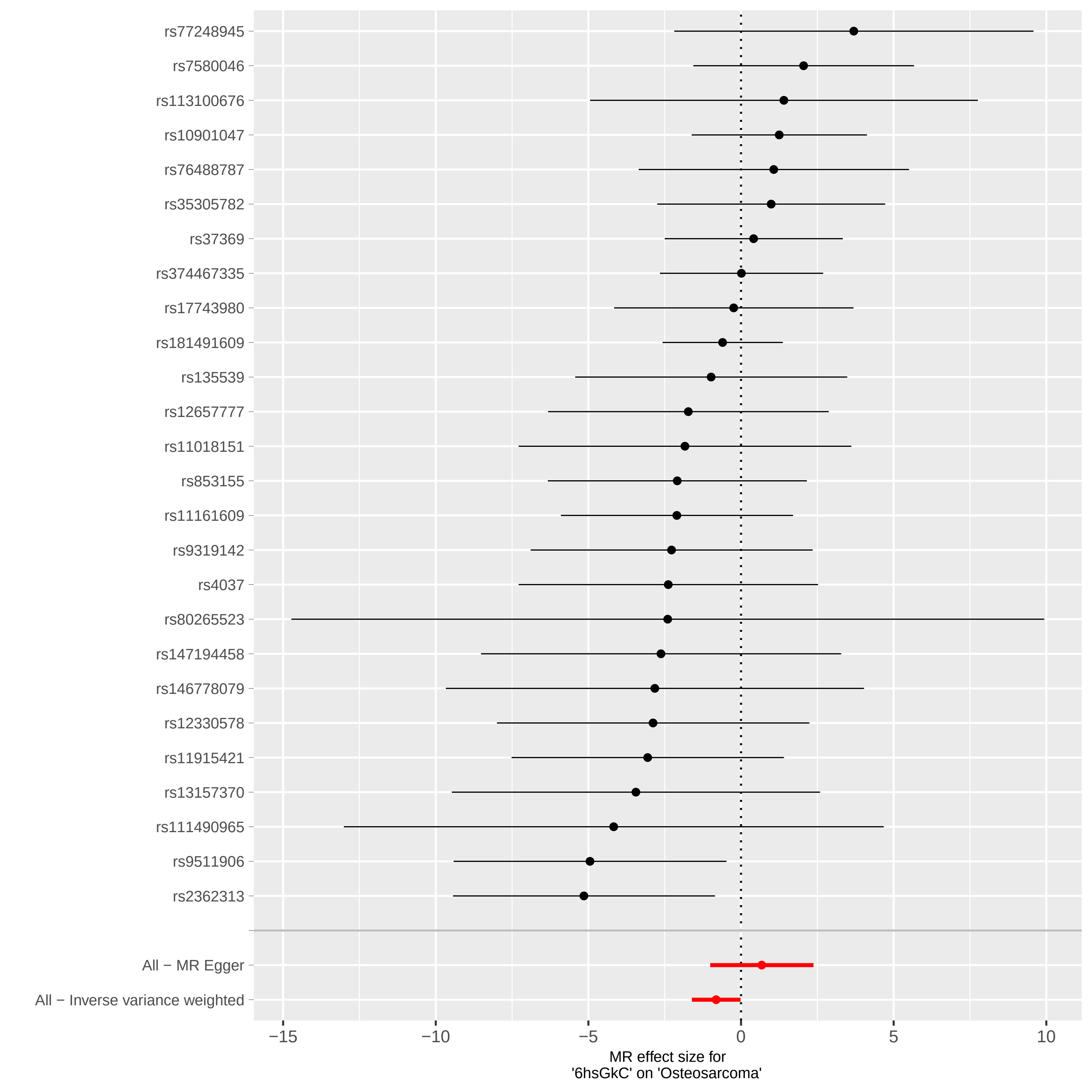

rs77248945
rs7580046
rs113100676
rs10901047
rs76488787
rs35305782
rs37369
rs374467335
rs17743980
rs181491609
rs135539
rs12657777
rs11018151
rs853155
rs11161609
rs9319142
rs4037
rs80265523
rs147194458
rs146778079
rs12330578
rs11915421
rs13157370
rs111490965
rs9511906
rs2362313
All − MR Egger
All − Inverse variance weighted
−15
−10
−5
0
5
10
MR effect size for
'6hsGkC' on 'Osteosarcoma'

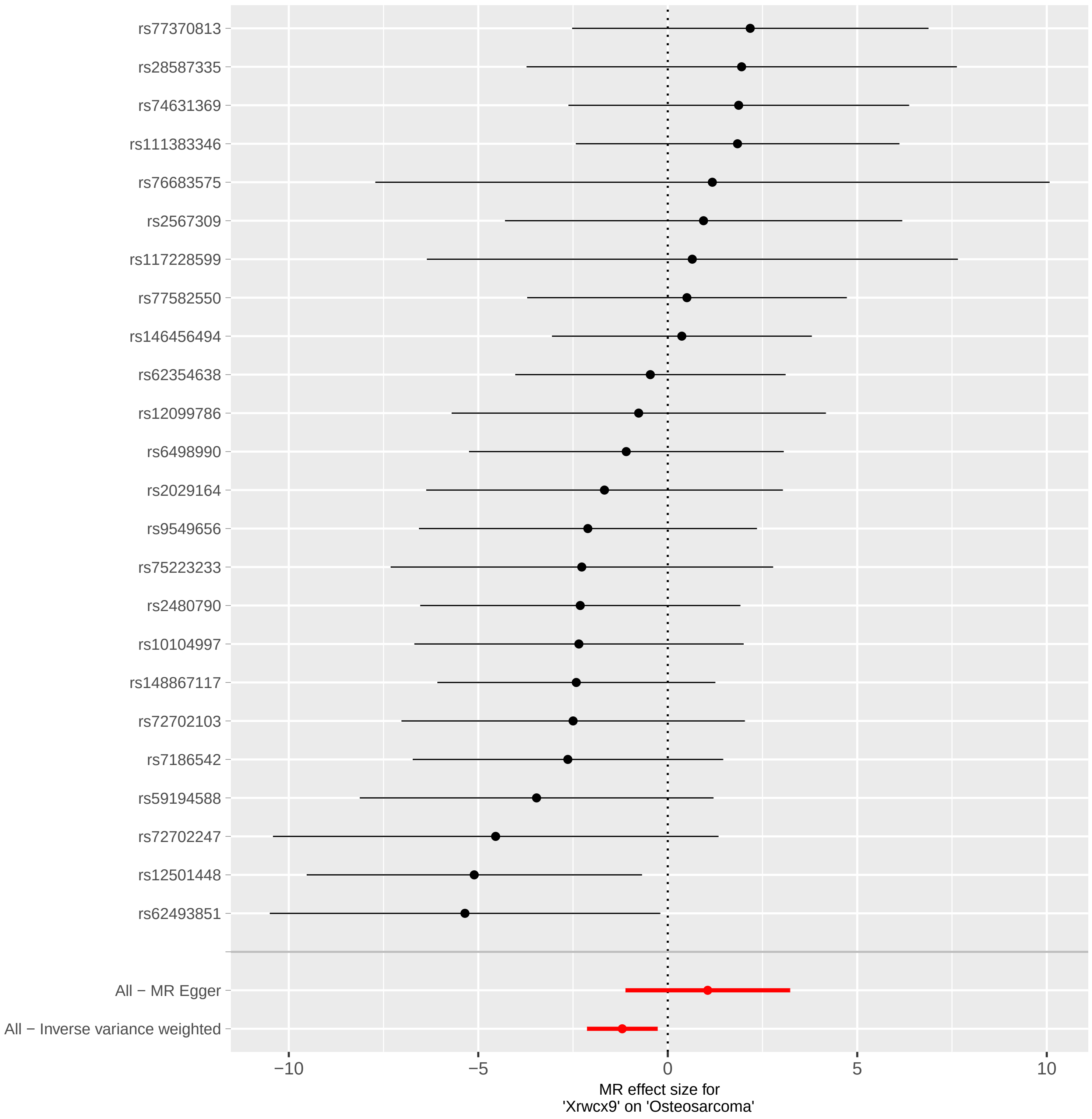
rs77370813
rs28587335
rs74631369
rs111383346
rs76683575
rs2567309
rs117228599
rs77582550
rs146456494
rs62354638
rs12099786
rs6498990
rs2029164
rs9549656
rs75223233
rs2480790
rs10104997
rs148867117
rs72702103
rs7186542
rs59194588
rs72702247
rs12501448
rs62493851
All − MR Egger
All − Inverse variance weighted
−10
−5
0
5
10
MR effect size for
'Xrwcx9' on 'Osteosarcoma'

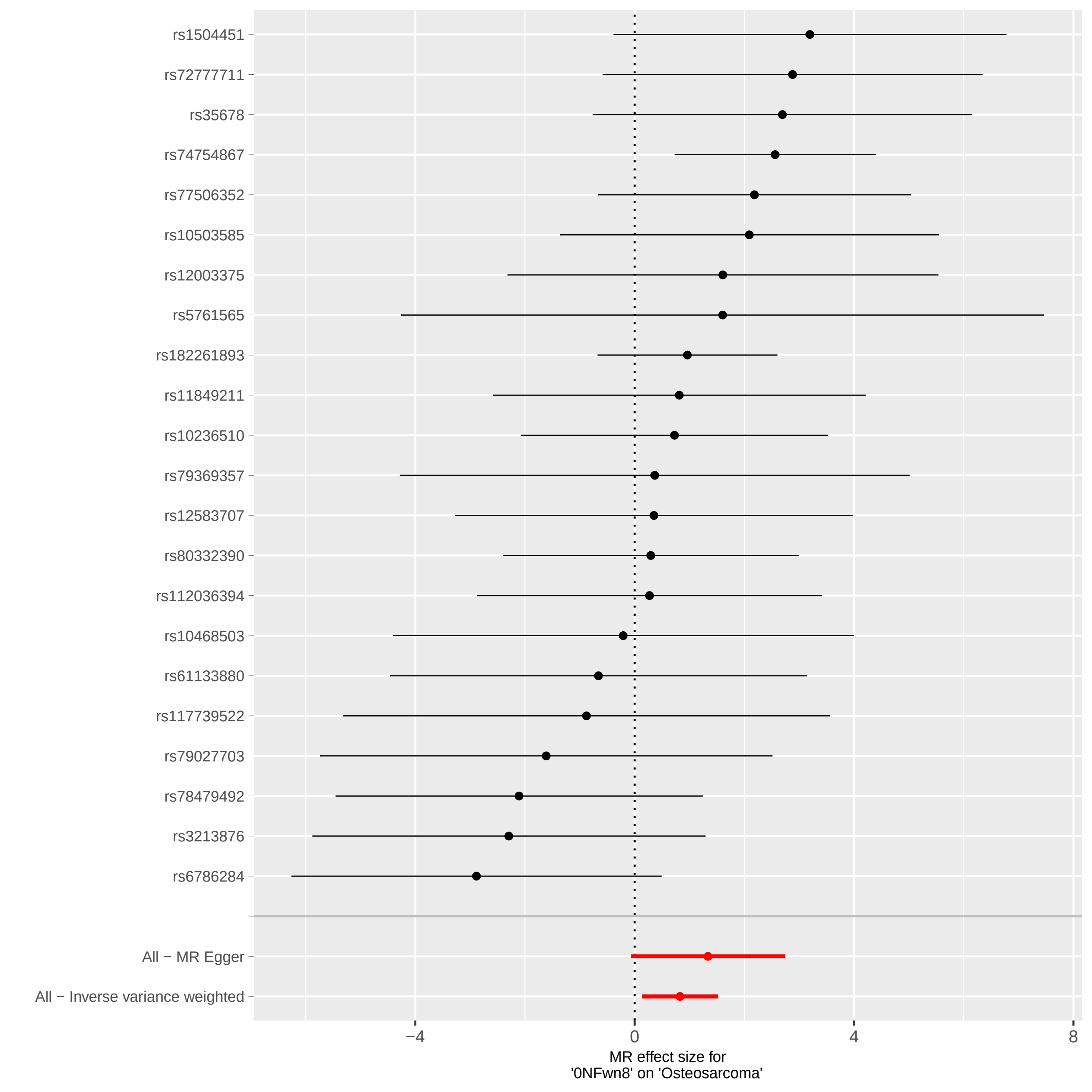

rs1504451
rs72777711
rs35678
rs74754867
rs77506352
rs10503585
rs12003375
rs5761565
rs182261893
rs11849211
rs10236510
rs79369357
rs12583707
rs80332390
rs112036394
rs10468503
rs61133880
rs117739522
rs79027703
rs78479492
rs3213876
rs6786284
All − MR Egger
All − Inverse variance weighted
−4
0
4
8
MR effect size for
'0NFwn8' on 'Osteosarcoma'

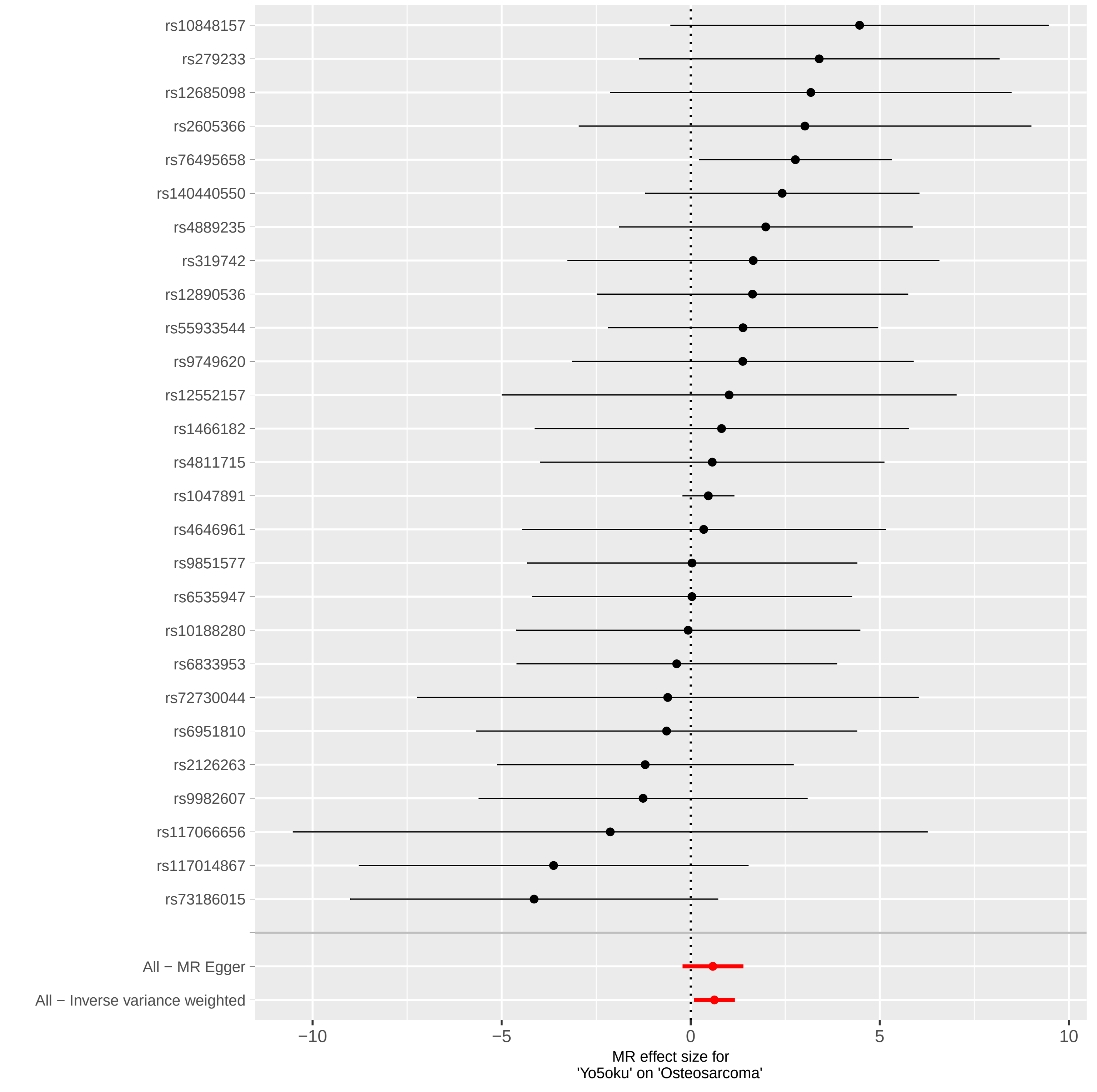
rs10848157
rs279233
rs12685098
rs2605366
rs76495658
rs140440550
rs4889235
rs319742
rs12890536
rs55933544
rs9749620
rs12552157
rs1466182
rs4811715
rs1047891
rs4646961
rs9851577
rs6535947
rs10188280
rs6833953
rs72730044
rs6951810
rs2126263
rs9982607
rs117066656
rs117014867
rs73186015
All − MR Egger
All − Inverse variance weighted
−10
−5
0
5
10
MR effect size for
'Yo5oku' on 'Osteosarcoma'

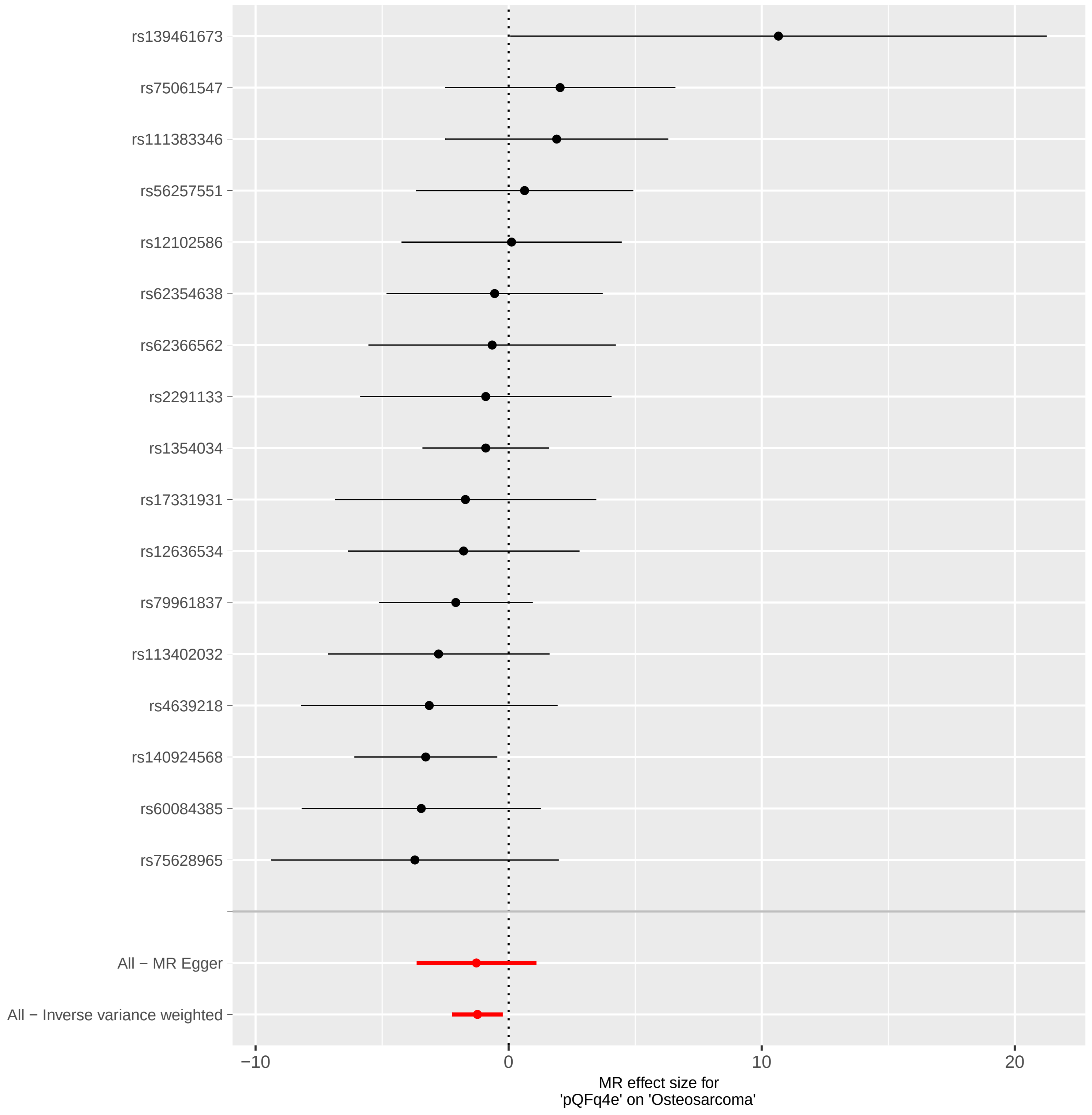
rs139461673
rs75061547
rs111383346
rs56257551
rs12102586
rs62354638
rs62366562
rs2291133
rs1354034
rs17331931
rs12636534
rs79961837
rs113402032
rs4639218
rs140924568
rs60084385
rs75628965
All − MR Egger
All − Inverse variance weighted
−10
0
10
20
MR effect size for
'pQFq4e' on 'Osteosarcoma'

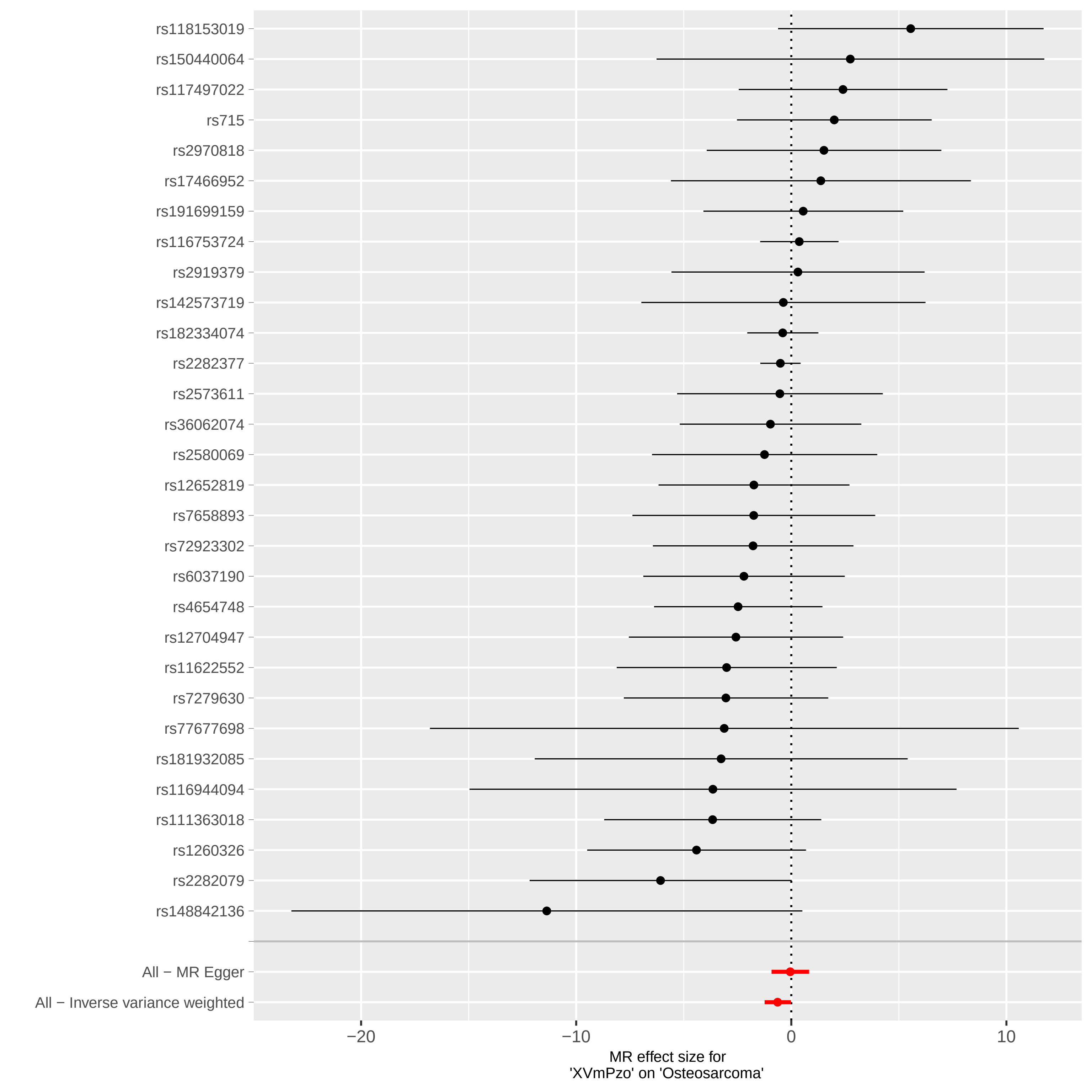

rs118153019
rs150440064
rs117497022
rs715
rs2970818
rs17466952
rs191699159
rs116753724
rs2919379
rs142573719
rs182334074
rs2282377
rs2573611
rs36062074
rs2580069
rs12652819
rs7658893
rs72923302
rs6037190
rs4654748
rs12704947
rs11622552
rs7279630
rs77677698
rs181932085
rs116944094
rs111363018
rs1260326
rs2282079
rs148842136
All − MR Egger
All − Inverse variance weighted
−20
−10
0
10
MR effect size for
'XVmPzo' on 'Osteosarcoma'

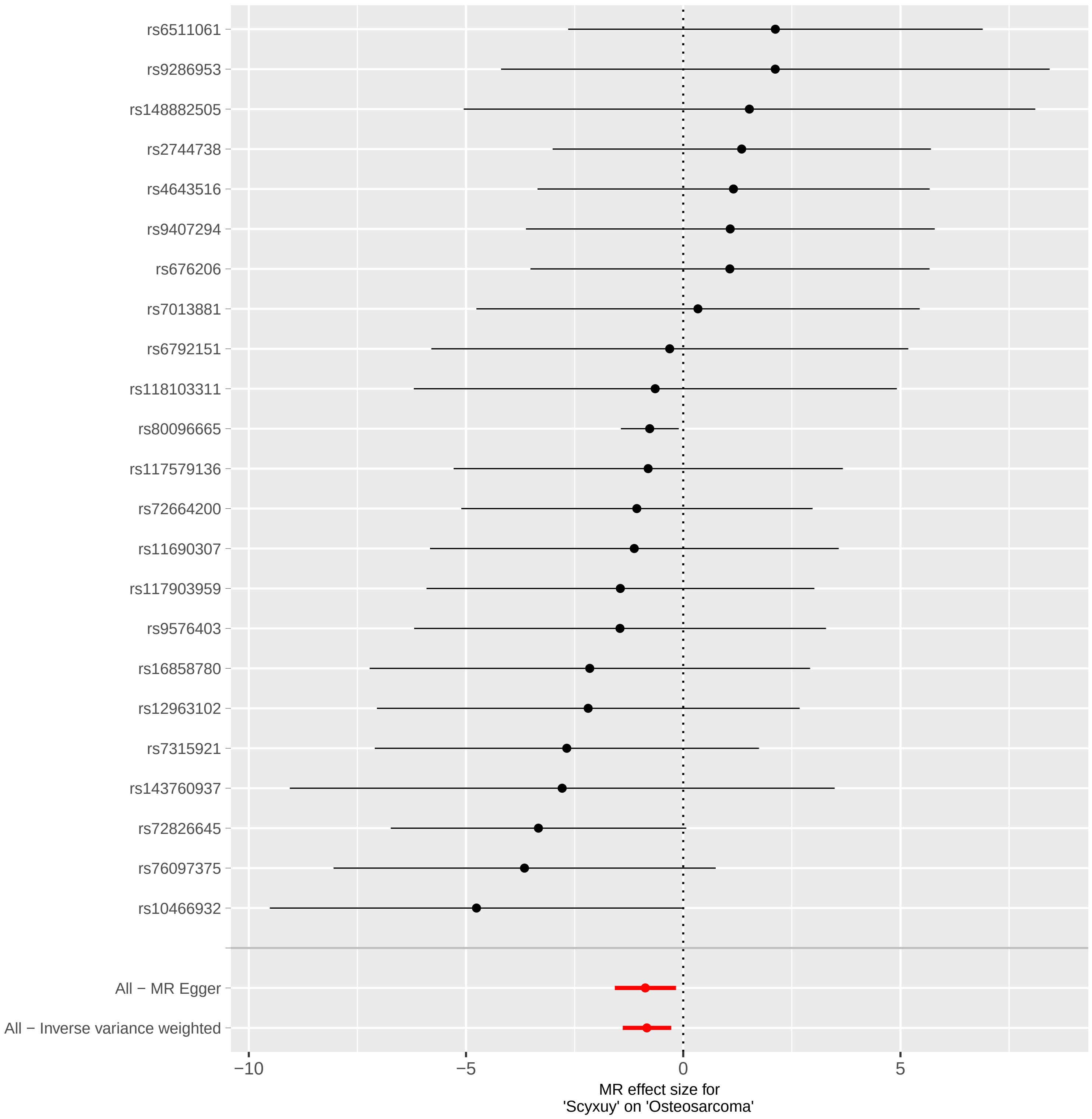

rs6511061
rs9286953
rs148882505
rs2744738
rs4643516
rs9407294
rs676206
rs7013881
rs6792151
rs118103311
rs80096665
rs117579136
rs72664200
rs11690307
rs117903959
rs9576403
rs16858780
rs12963102
rs7315921
rs143760937
rs72826645
rs76097375
rs10466932
All − MR Egger
All − Inverse variance weighted
−10
−5
0
5
MR effect size for
'Scyxuy' on 'Osteosarcoma'

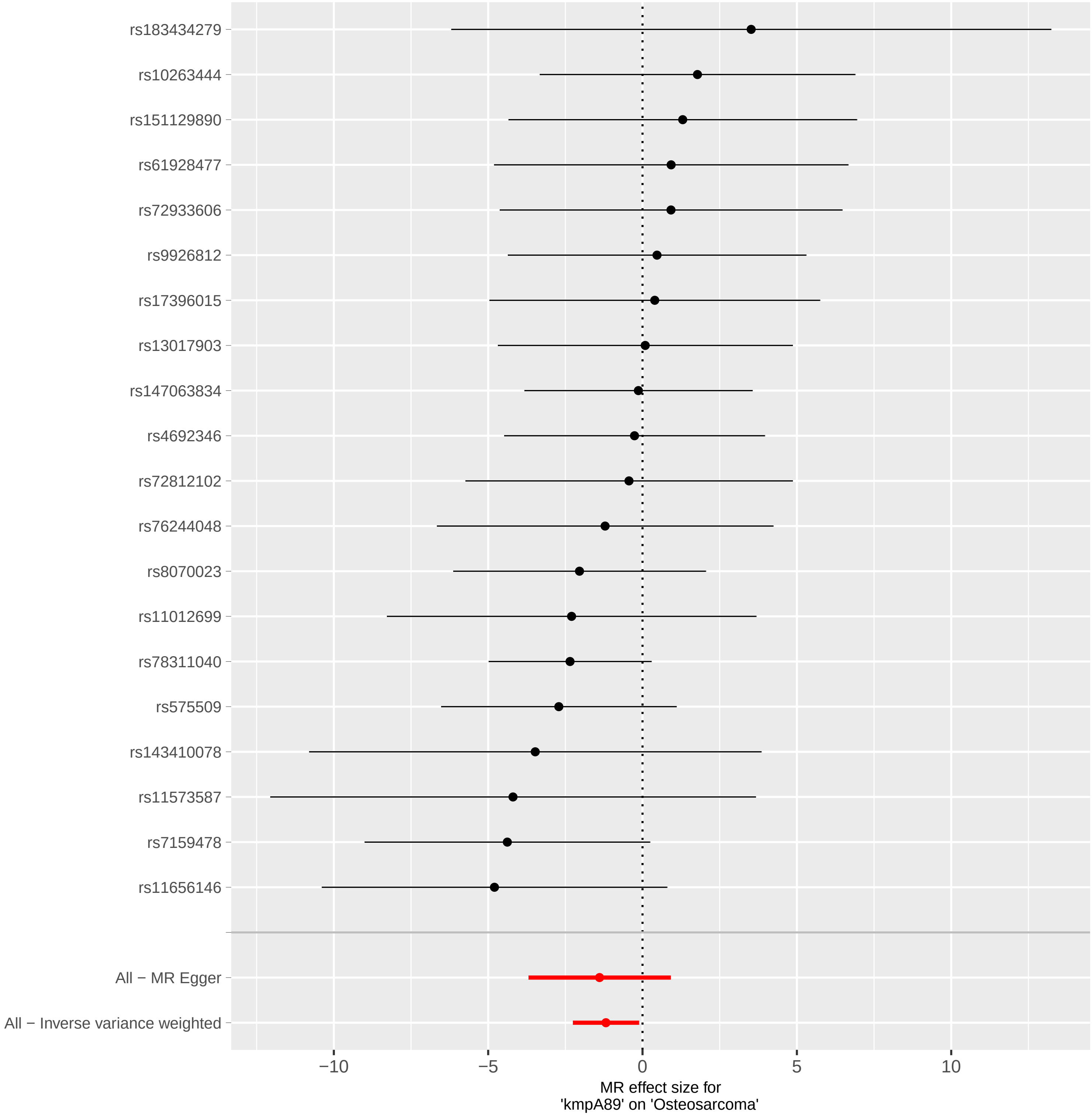

rs183434279
rs10263444
rs151129890
rs61928477
rs72933606
rs9926812
rs17396015
rs13017903
rs147063834
rs4692346
rs72812102
rs76244048
rs8070023
rs11012699
rs78311040
rs575509
rs143410078
rs11573587
rs7159478
rs11656146
All − MR Egger
All − Inverse variance weighted
−10
−5
0
5
10
MR effect size for
'kmpA89' on 'Osteosarcoma'

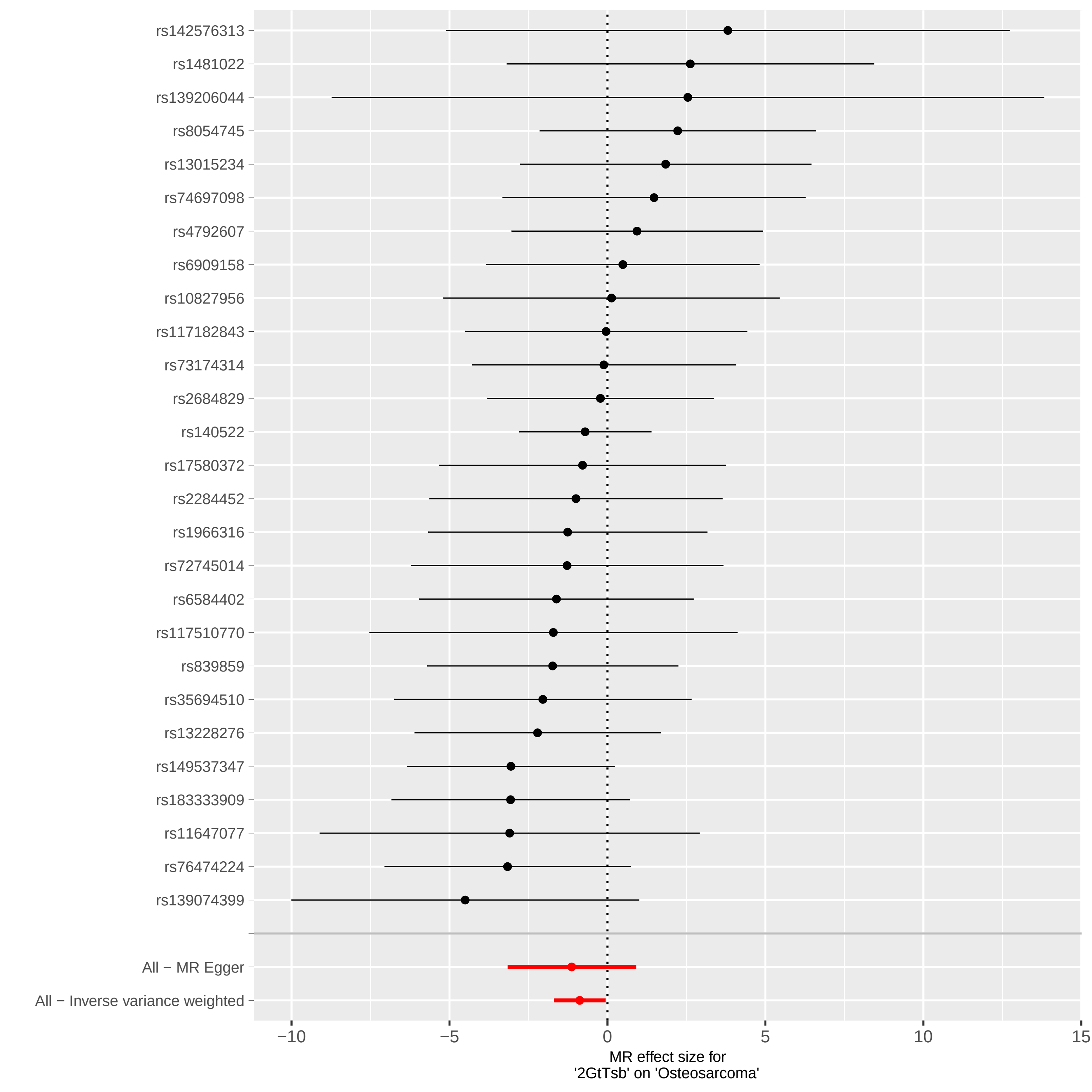
rs142576313
rs1481022
rs139206044
rs8054745
rs13015234
rs74697098
rs4792607
rs6909158
rs10827956
rs117182843
rs73174314
rs2684829
rs140522
rs17580372
rs2284452
rs1966316
rs72745014
rs6584402
rs117510770
rs839859
rs35694510
rs13228276
rs149537347
rs183333909
rs11647077
rs76474224
rs139074399
All − MR Egger
All − Inverse variance weighted
−10
−5
0
5
10
15
MR effect size for
'2GtTsb' on 'Osteosarcoma'

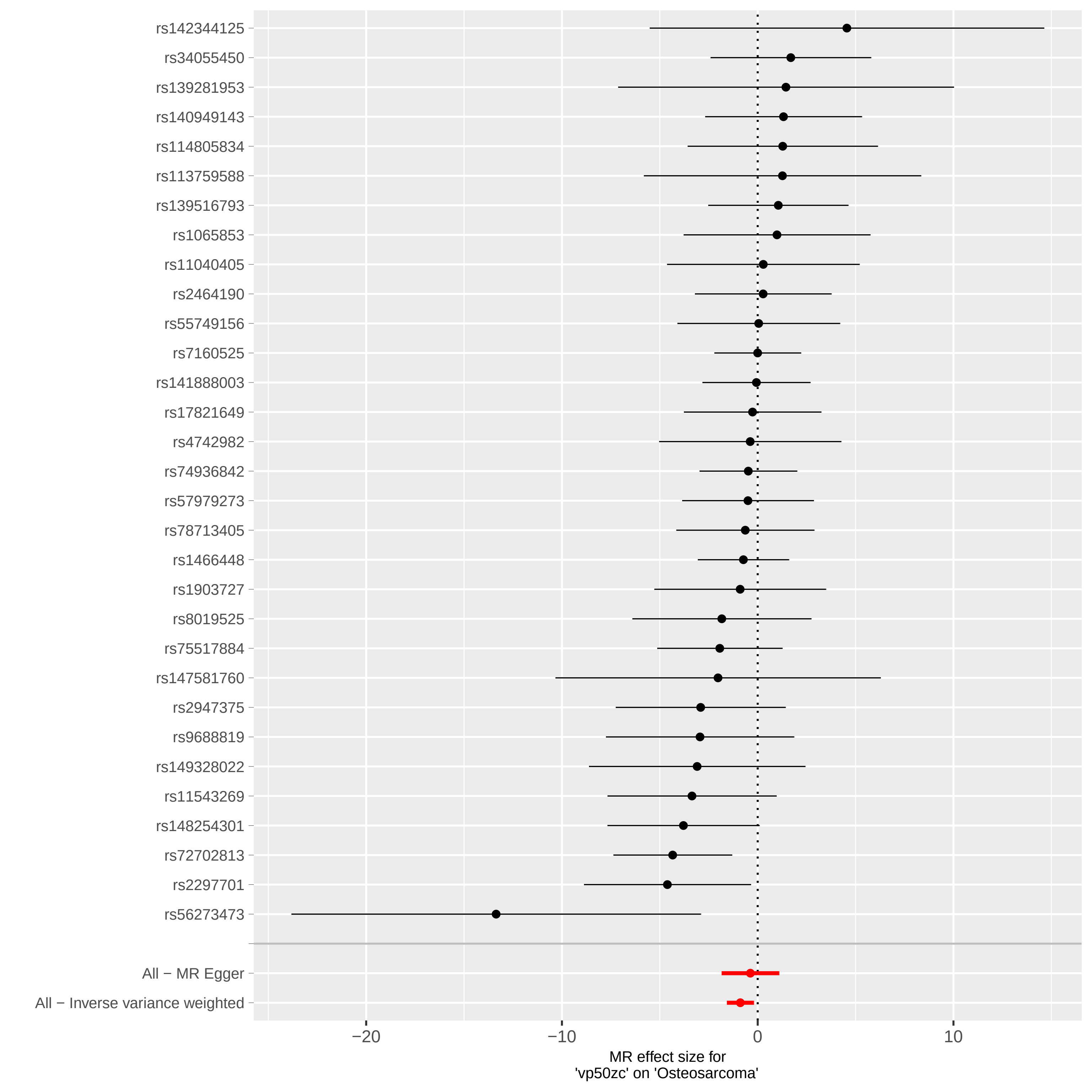
rs142344125
rs34055450
rs139281953
rs140949143
rs114805834
rs113759588
rs139516793
rs1065853
rs11040405
rs2464190
rs55749156
rs7160525
rs141888003
rs17821649
rs4742982
rs74936842
rs57979273
rs78713405
rs1466448
rs1903727
rs8019525
rs75517884
rs147581760
rs2947375
rs9688819
rs149328022
rs11543269
rs148254301
rs72702813
rs2297701
rs56273473
All − MR Egger
All − Inverse variance weighted
−20
−10
0
10
MR effect size for
'vp50zc' on 'Osteosarcoma'

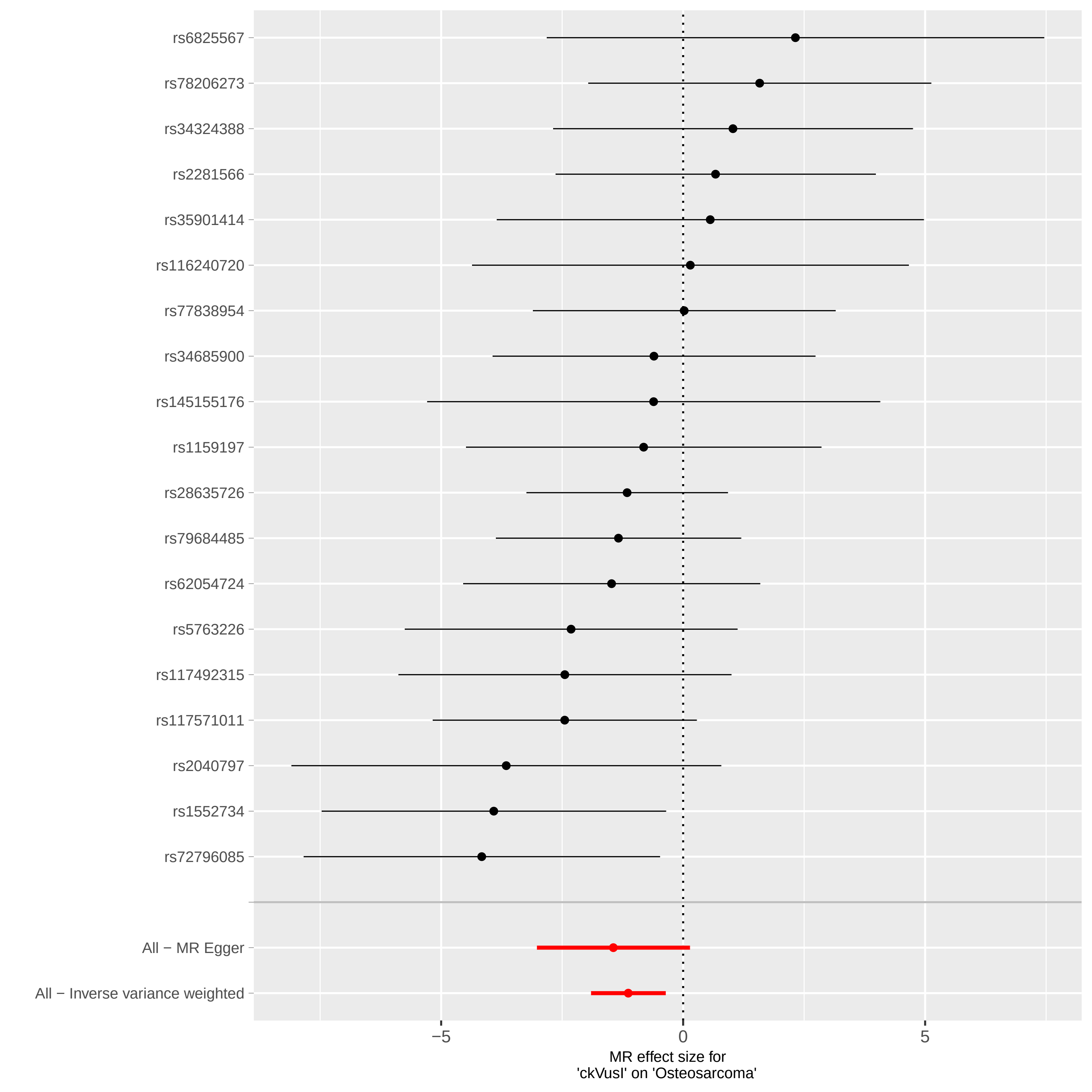
rs6825567
rs78206273
rs34324388
rs2281566
rs35901414
rs116240720
rs77838954
rs34685900
rs145155176
rs1159197
rs28635726
rs79684485
rs62054724
rs5763226
rs117492315
rs117571011
rs2040797
rs1552734
rs72796085
All − MR Egger
All − Inverse variance weighted
−5
0
5
MR effect size for
'ckVusI' on 'Osteosarcoma'

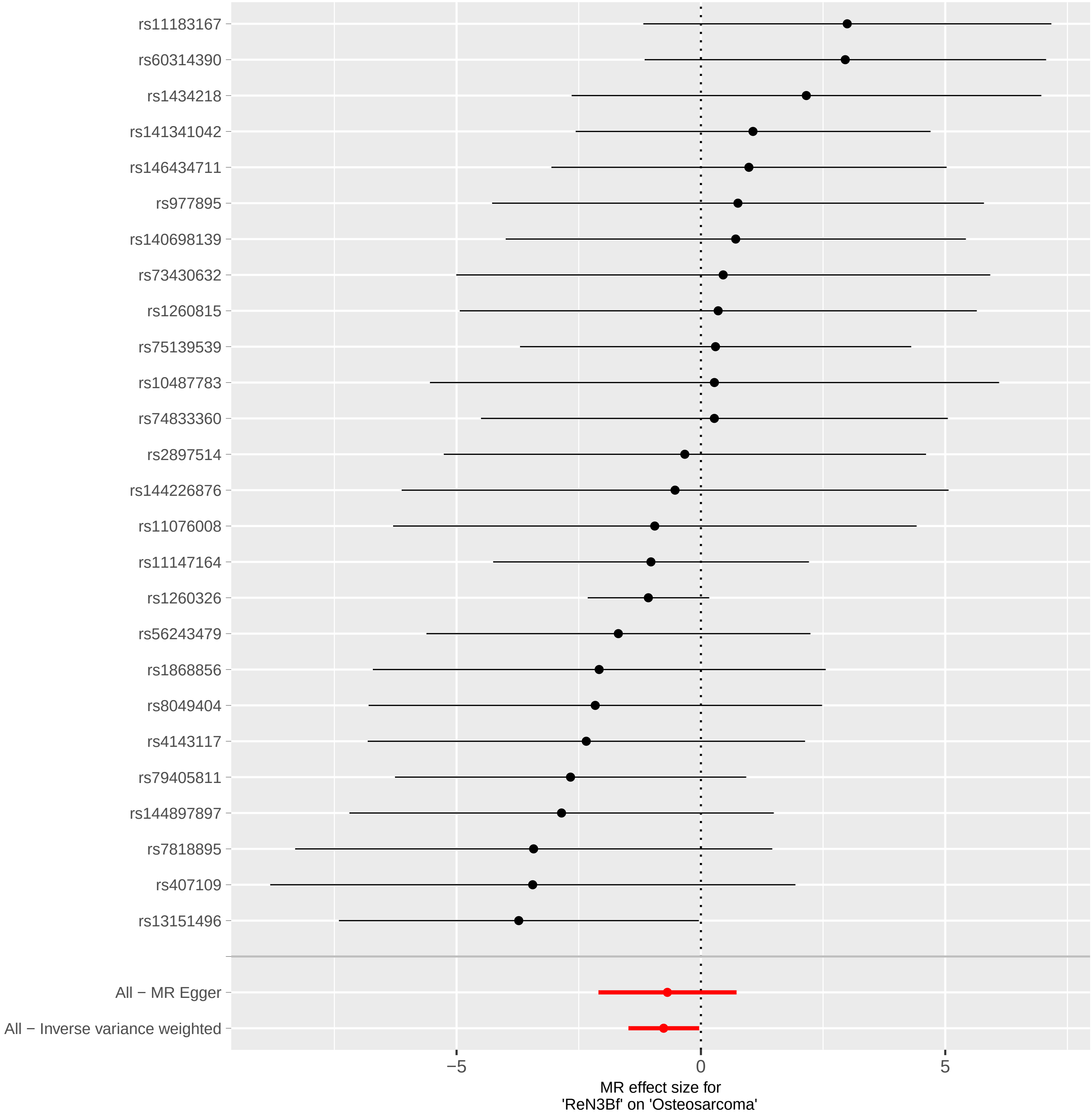
rs11183167
rs60314390
rs1434218
rs141341042
rs146434711
rs977895
rs140698139
rs73430632
rs1260815
rs75139539
rs10487783
rs74833360
rs2897514
rs144226876
rs11076008
rs11147164
rs1260326
rs56243479
rs1868856
rs8049404
rs4143117
rs79405811
rs144897897
rs7818895
rs407109
rs13151496
All − MR Egger
All − Inverse variance weighted
−5
0
5
MR effect size for
'ReN3Bf' on 'Osteosarcoma'

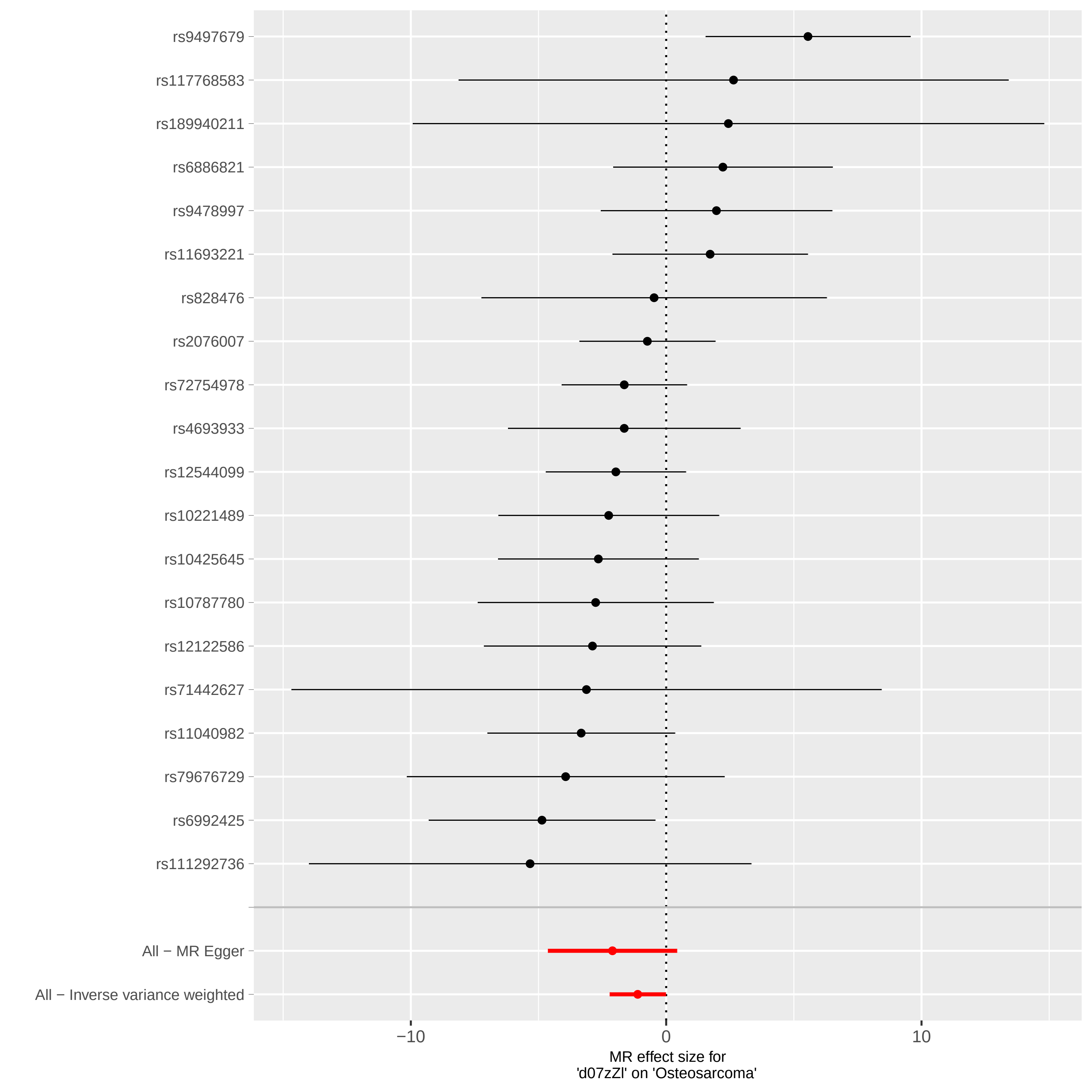
rs9497679
rs117768583
rs189940211
rs6886821
rs9478997
rs11693221
rs828476
rs2076007
rs72754978
rs4693933
rs12544099
rs10221489
rs10425645
rs10787780
rs12122586
rs71442627
rs11040982
rs79676729
rs6992425
rs111292736
All − MR Egger
All − Inverse variance weighted
−10
0
10
MR effect size for
'd07zZl' on 'Osteosarcoma'

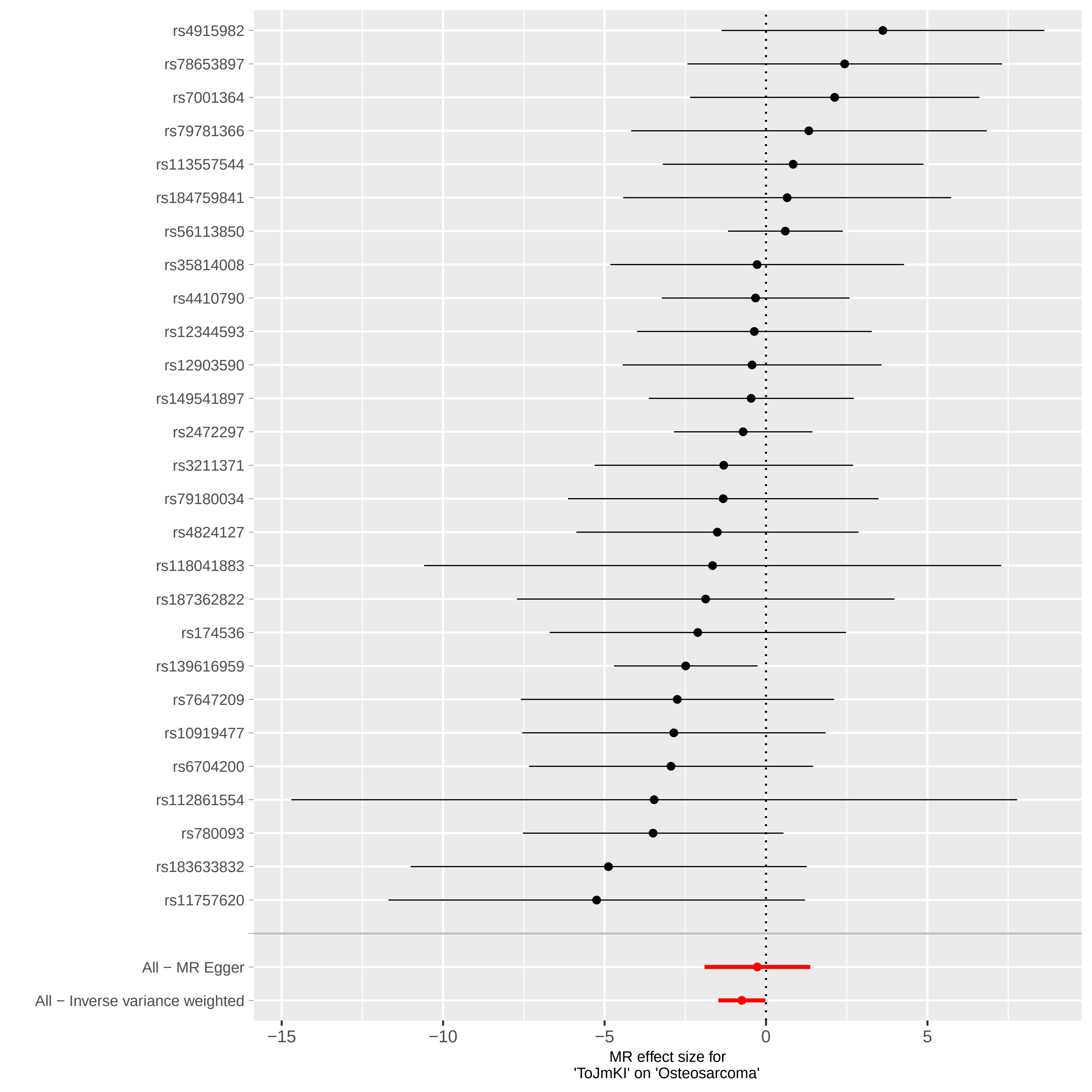

rs4915982
rs78653897
rs7001364
rs79781366
rs113557544
rs184759841
rs56113850
rs35814008
rs4410790
rs12344593
rs12903590
rs149541897
rs2472297
rs3211371
rs79180034
rs4824127
rs118041883
rs187362822
rs174536
rs139616959
rs7647209
rs10919477
rs6704200
rs112861554
rs780093
rs183633832
rs11757620
All − MR Egger
All − Inverse variance weighted
−15
−10
−5
0
5
MR effect size for
'ToJmKI' on 'Osteosarcoma'

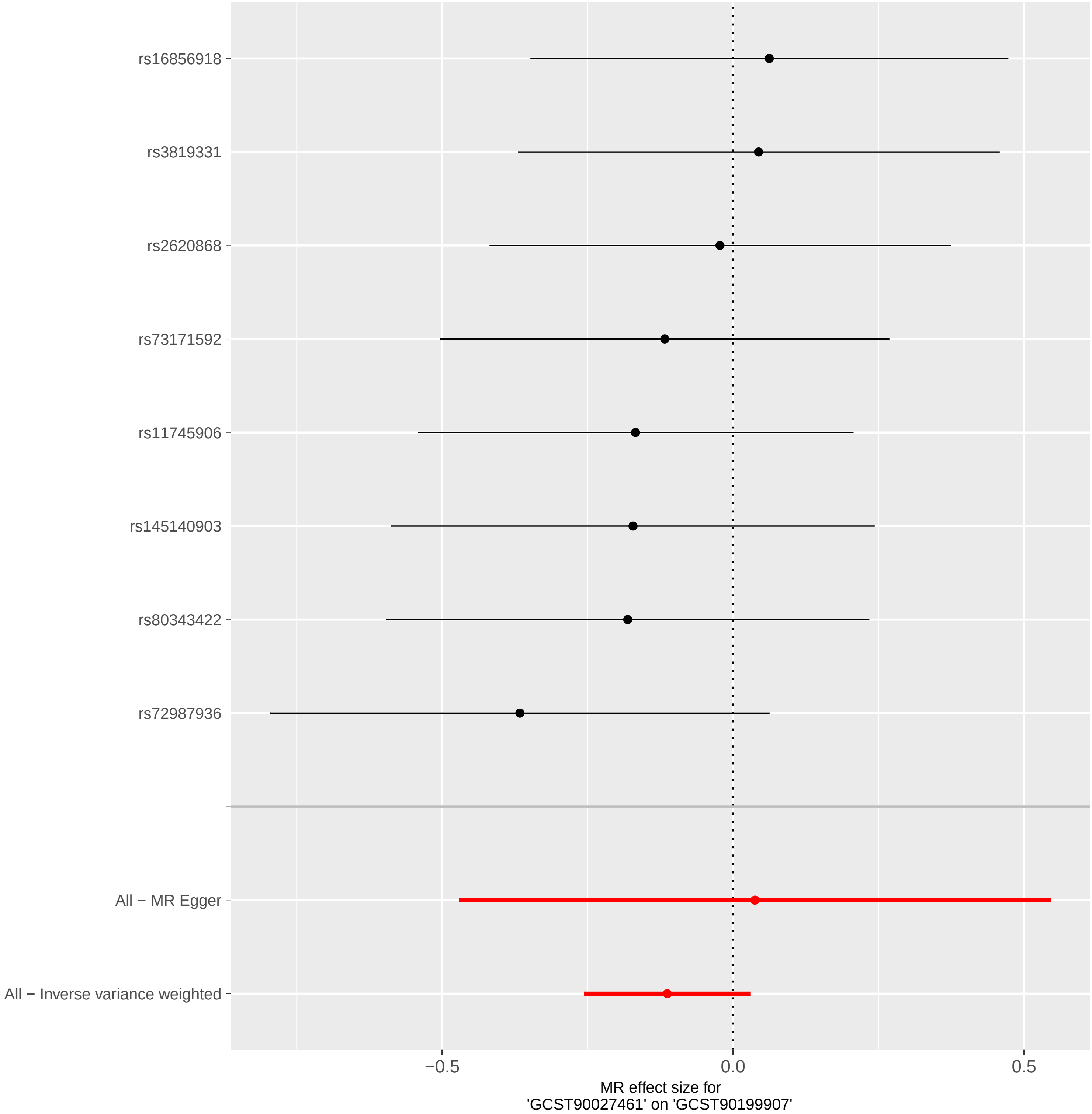

rs16856918
rs3819331
rs2620868
rs73171592
rs11745906
rs145140903
rs80343422
rs72987936
All − MR Egger
All − Inverse variance weighted
−0.5
0.0
0.5
MR effect size for
'GCST90027461' on 'GCST90199907'

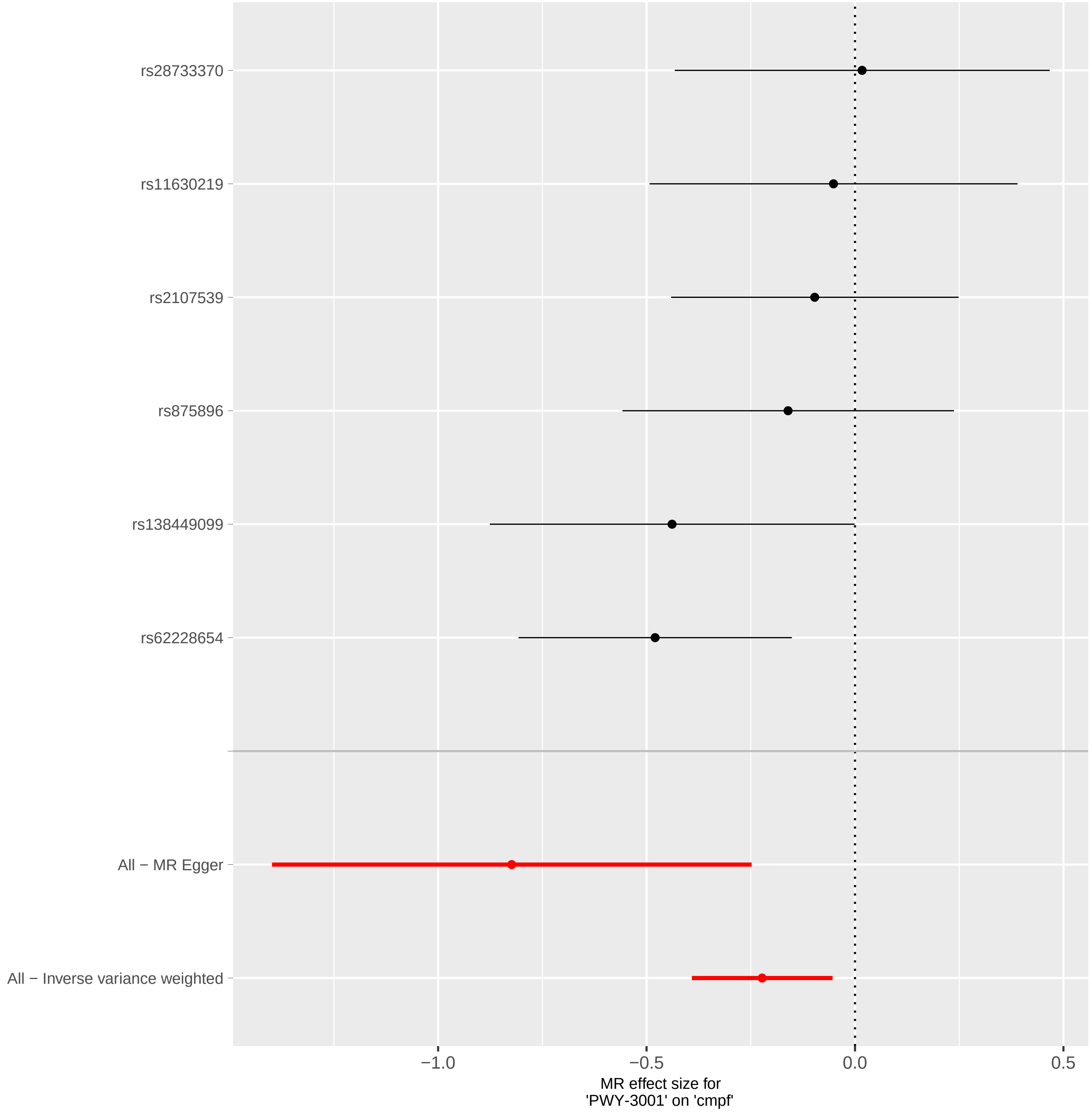
rs28733370
rs11630219
rs2107539
rs875896
rs138449099
rs62228654
All − MR Egger
All − Inverse variance weighted
−1.0
−0.5
0.0
0.5
MR effect size for
'PWY-3001' on 'cmpf'

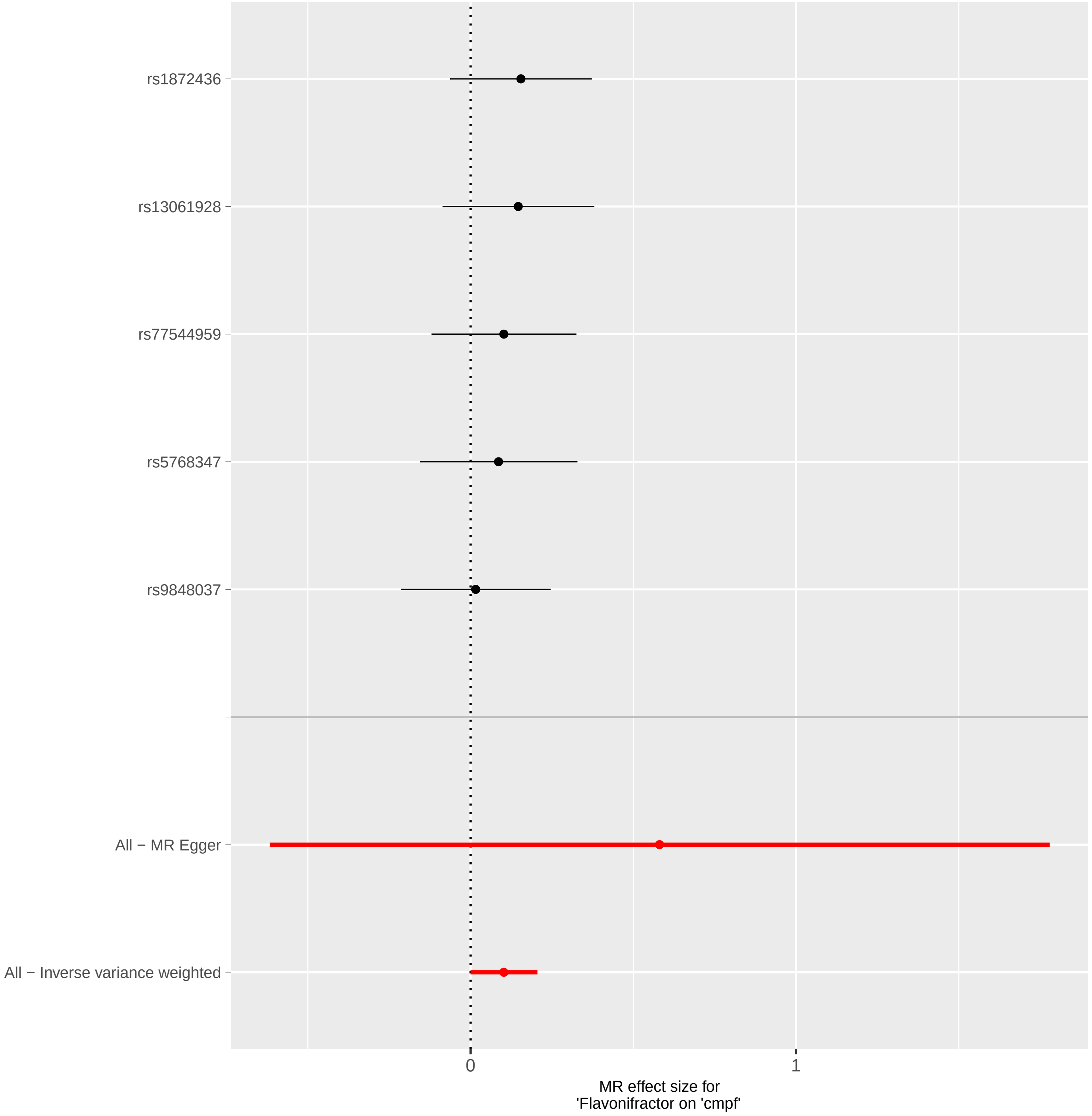
rs1872436
rs13061928
rs77544959
rs5768347
rs9848037
All − MR Egger
All − Inverse variance weighted
0
1
MR effect size for
'Flavonifractor on 'cmpf'

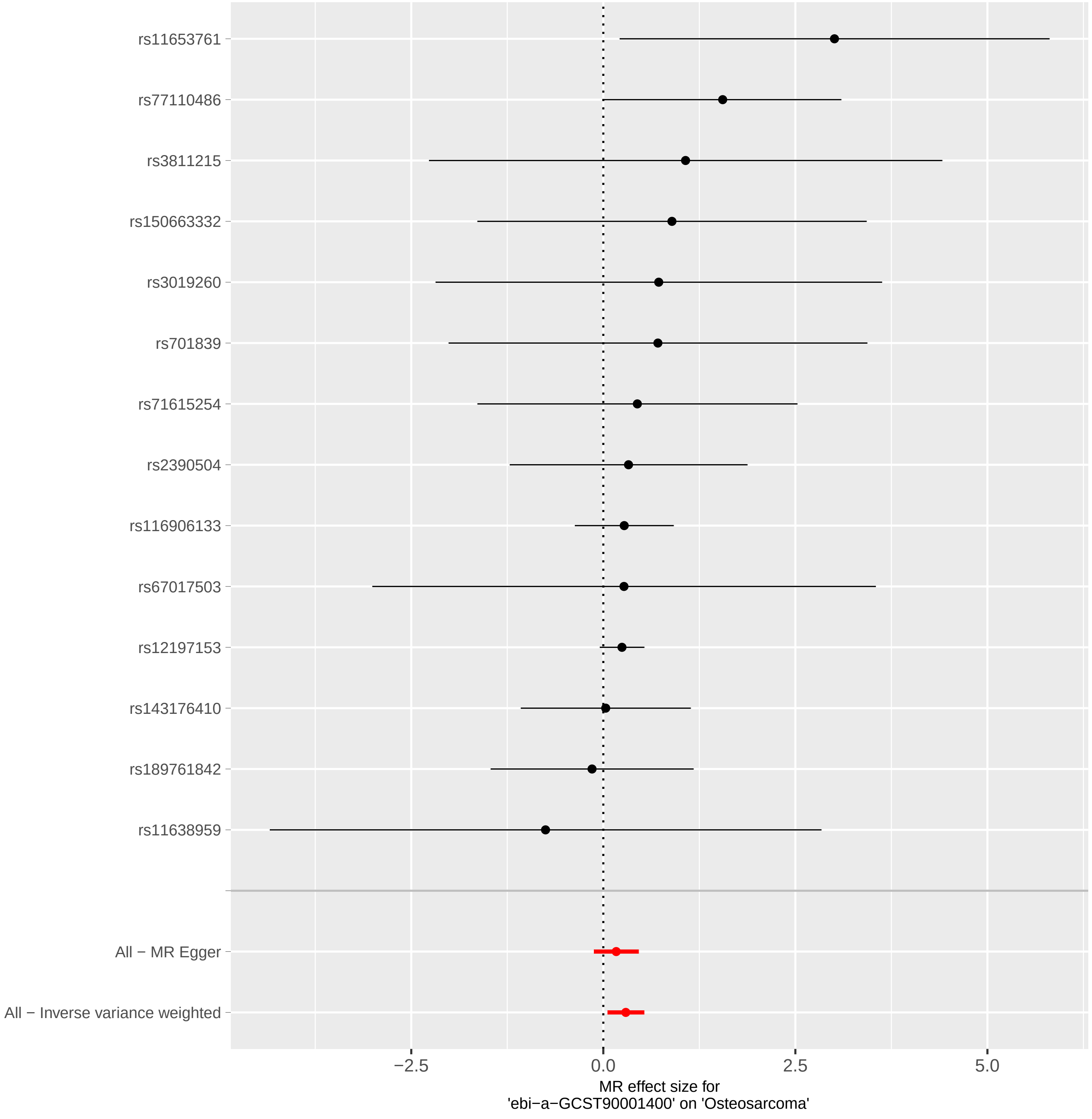
rs11653761
rs77110486
rs3811215
rs150663332
rs3019260
rs701839
rs71615254
rs2390504
rs116906133
rs67017503
rs12197153
rs143176410
rs189761842
rs11638959
All − MR Egger
All − Inverse variance weighted
−2.5
0.0
2.5
5.0
MR effect size for
'ebi−a−GCST90001400' on 'Osteosarcoma'

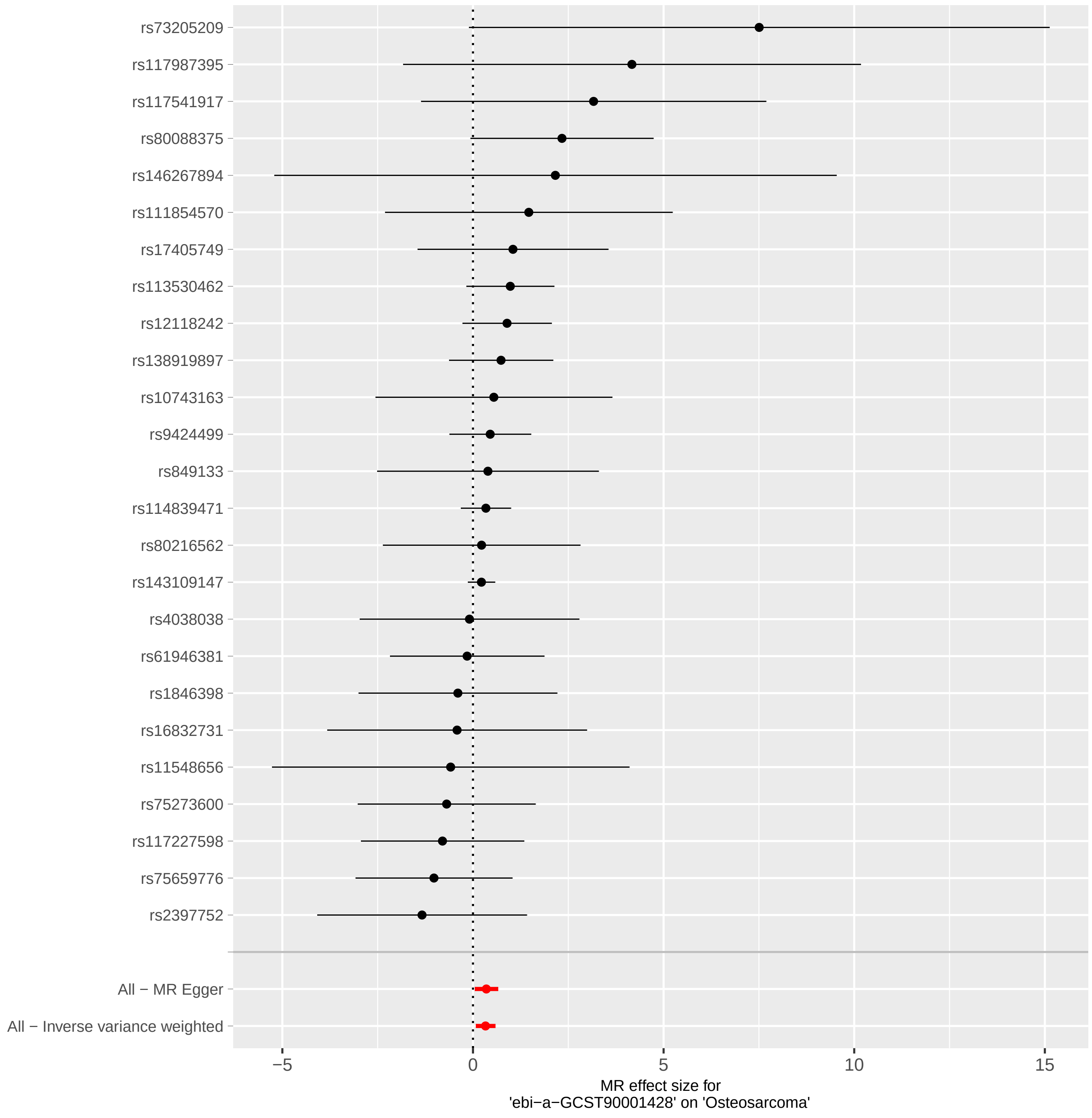

rs73205209
rs117987395
rs117541917
rs80088375
rs146267894
rs111854570
rs17405749
rs113530462
rs12118242
rs138919897
rs10743163
rs9424499
rs849133
rs114839471
rs80216562
rs143109147
rs4038038
rs61946381
rs1846398
rs16832731
rs11548656
rs75273600
rs117227598
rs75659776
rs2397752
All − MR Egger
All − Inverse variance weighted
−5
0
5
10
15
MR effect size for
'ebi−a−GCST90001428' on 'Osteosarcoma'

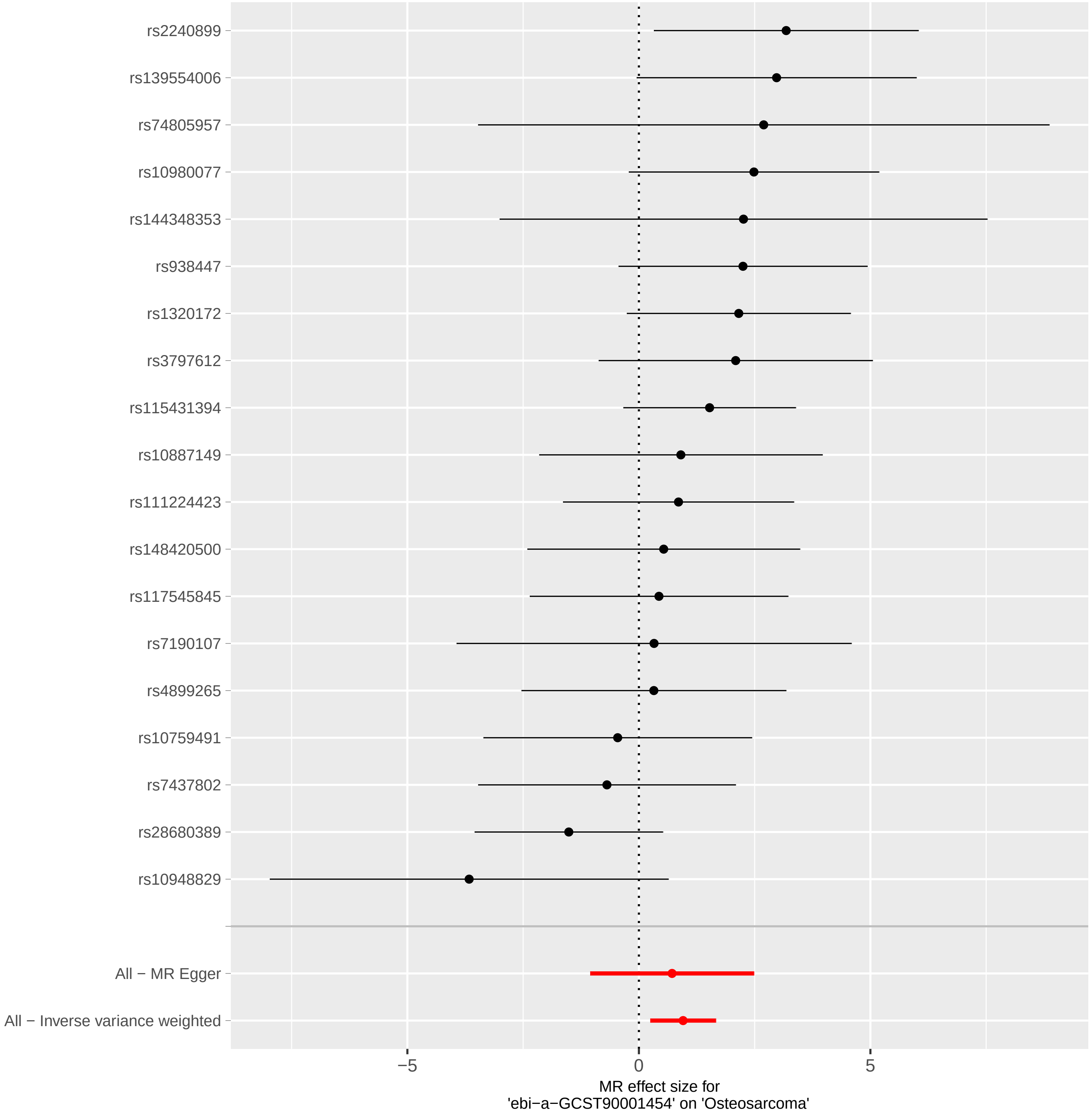

rs2240899
rs139554006
rs74805957
rs10980077
rs144348353
rs938447
rs1320172
rs3797612
rs115431394
rs10887149
rs111224423
rs148420500
rs117545845
rs7190107
rs4899265
rs10759491
rs7437802
rs28680389
rs10948829
All − MR Egger
All − Inverse variance weighted
−5
0
5
MR effect size for
'ebi−a−GCST90001454' on 'Osteosarcoma'

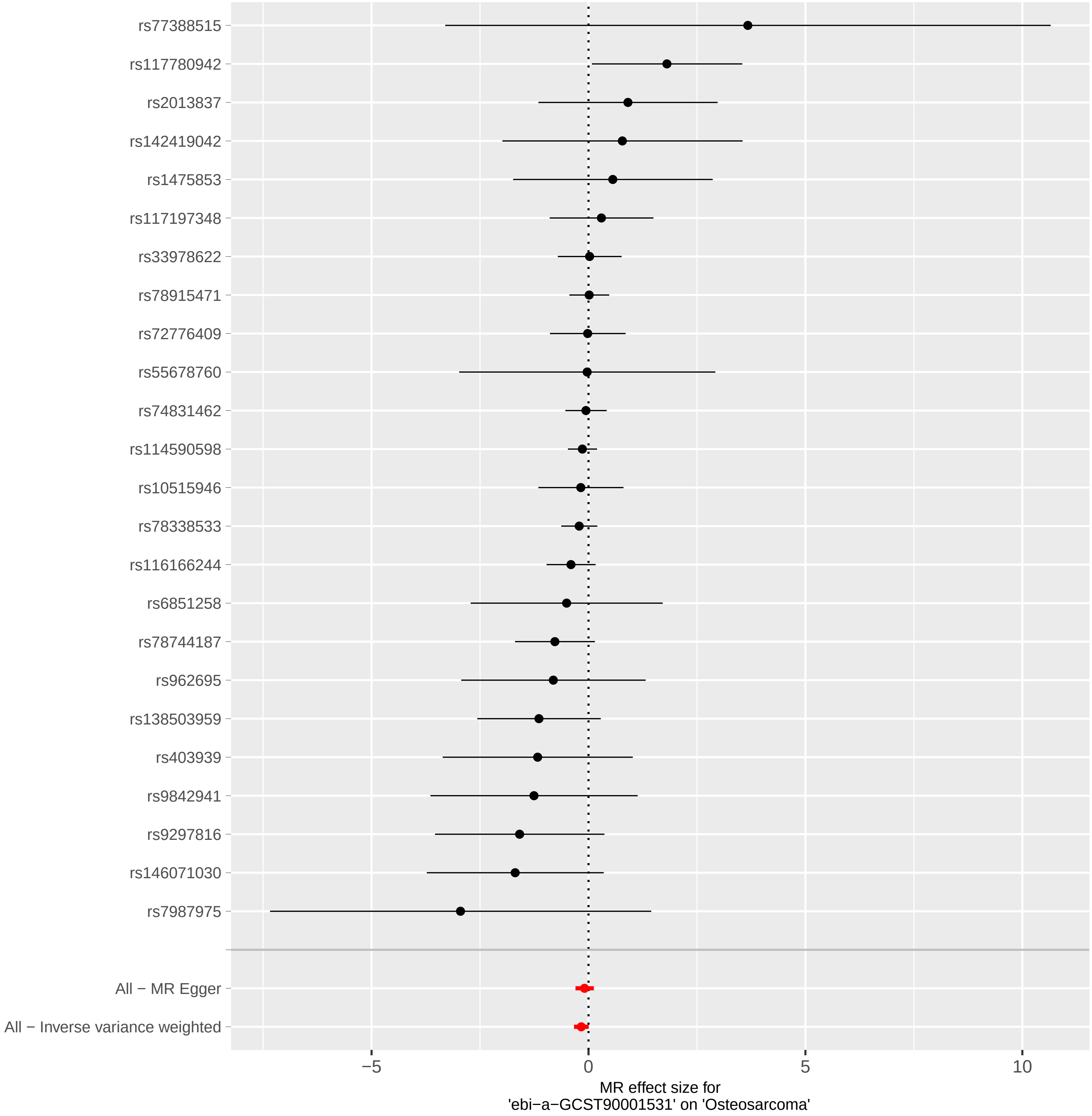

rs77388515
rs117780942
rs2013837
rs142419042
rs1475853
rs117197348
rs33978622
rs78915471
rs72776409
rs55678760
rs74831462
rs114590598
rs10515946
rs78338533
rs116166244
rs6851258
rs78744187
rs962695
rs138503959
rs403939
rs9842941
rs9297816
rs146071030
rs7987975
All − MR Egger
All − Inverse variance weighted
−5
0
5
10
MR effect size for
'ebi−a−GCST90001531' on 'Osteosarcoma'

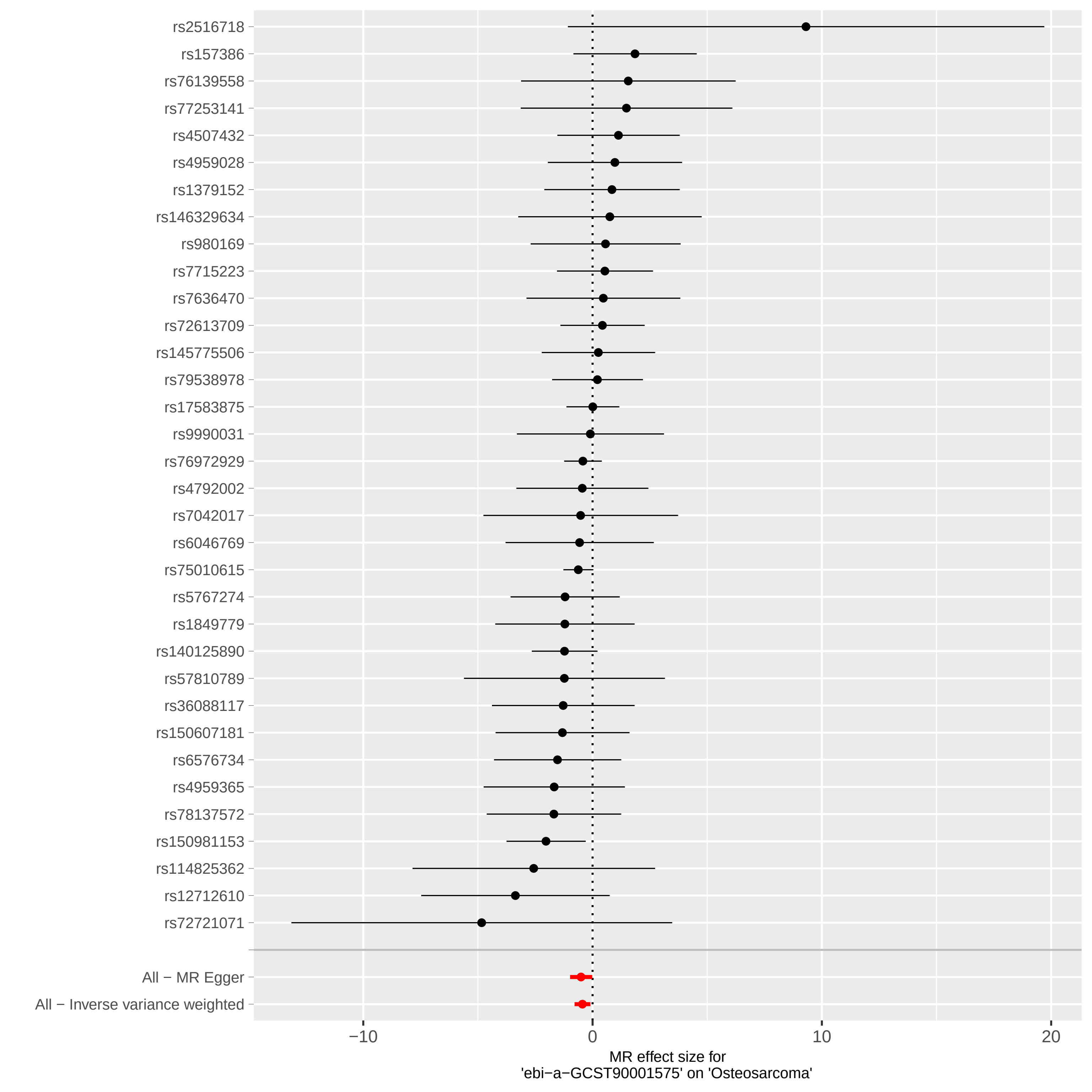
rs2516718
rs157386
rs76139558
rs77253141
rs4507432
rs4959028
rs1379152
rs146329634
rs980169
rs7715223
rs7636470
rs72613709
rs145775506
rs79538978
rs17583875
rs9990031
rs76972929
rs4792002
rs7042017
rs6046769
rs75010615
rs5767274
rs1849779
rs140125890
rs57810789
rs36088117
rs150607181
rs6576734
rs4959365
rs78137572
rs150981153
rs114825362
rs12712610
rs72721071
All − MR Egger
All − Inverse variance weighted
−10
0
10
20
MR effect size for
'ebi−a−GCST90001575' on 'Osteosarcoma'

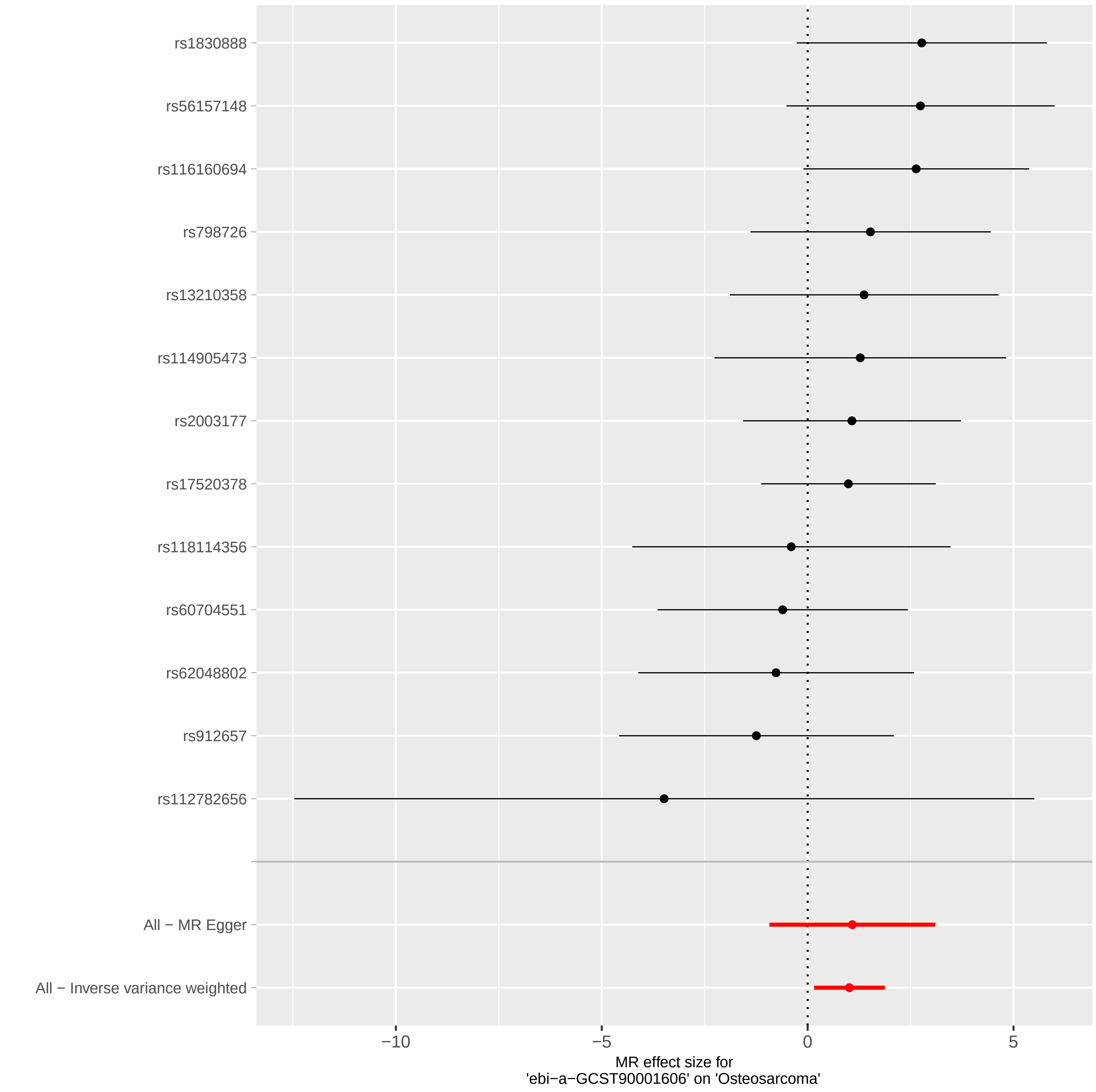

rs1830888
rs56157148
rs116160694
rs798726
rs13210358
rs114905473
rs2003177
rs17520378
rs118114356
rs60704551
rs62048802
rs912657
rs112782656
All − MR Egger
All − Inverse variance weighted
−10
−5
0
5
MR effect size for
'ebi−a−GCST90001606' on 'Osteosarcoma'

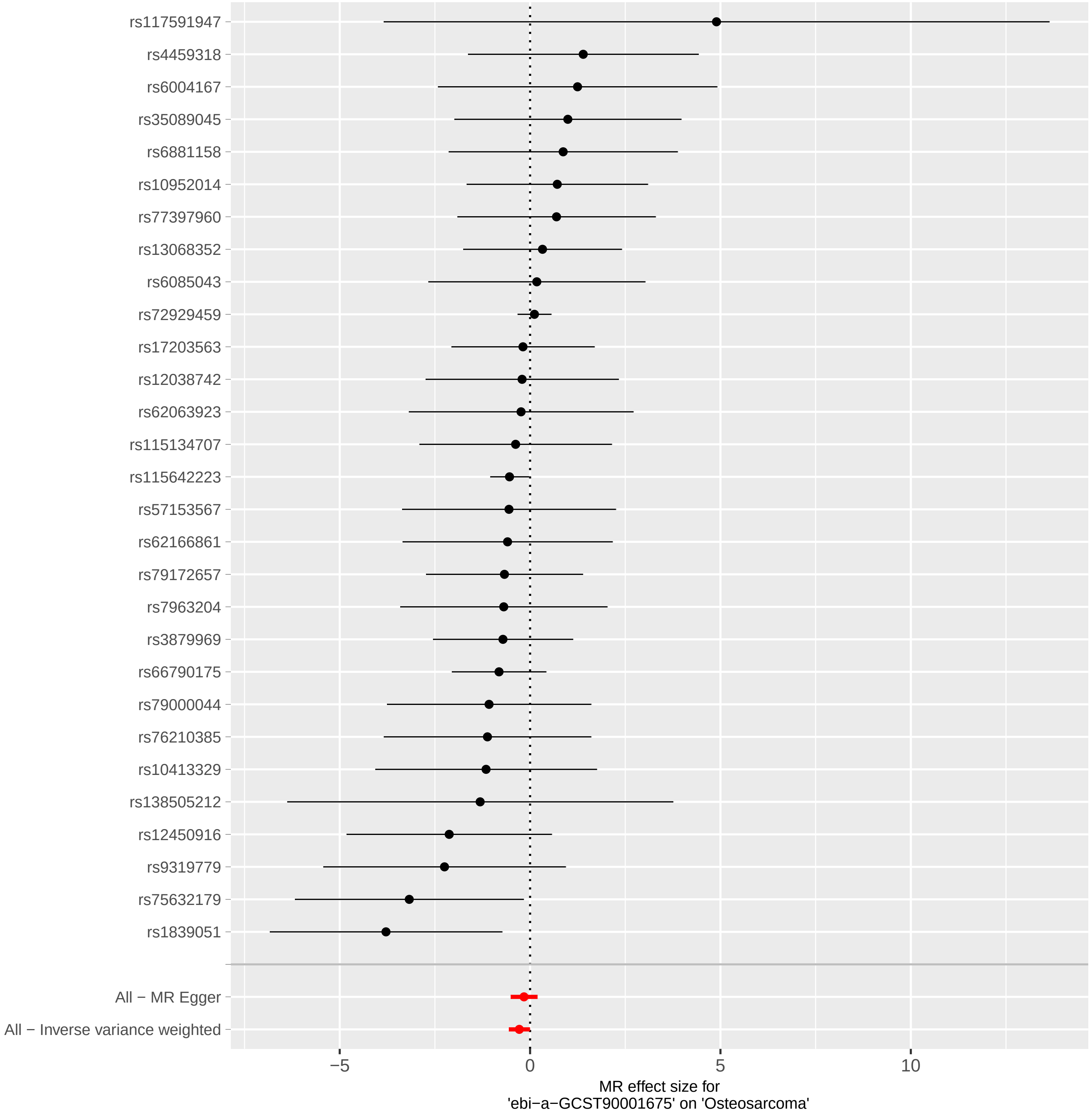
rs117591947
rs4459318
rs6004167
rs35089045
rs6881158
rs10952014
rs77397960
rs13068352
rs6085043
rs72929459
rs17203563
rs12038742
rs62063923
rs115134707
rs115642223
rs57153567
rs62166861
rs79172657
rs7963204
rs3879969
rs66790175
rs79000044
rs76210385
rs10413329
rs138505212
rs12450916
rs9319779
rs75632179
rs1839051
All − MR Egger
All − Inverse variance weighted
−5
0
5
10
MR effect size for
'ebi−a−GCST90001675' on 'Osteosarcoma'

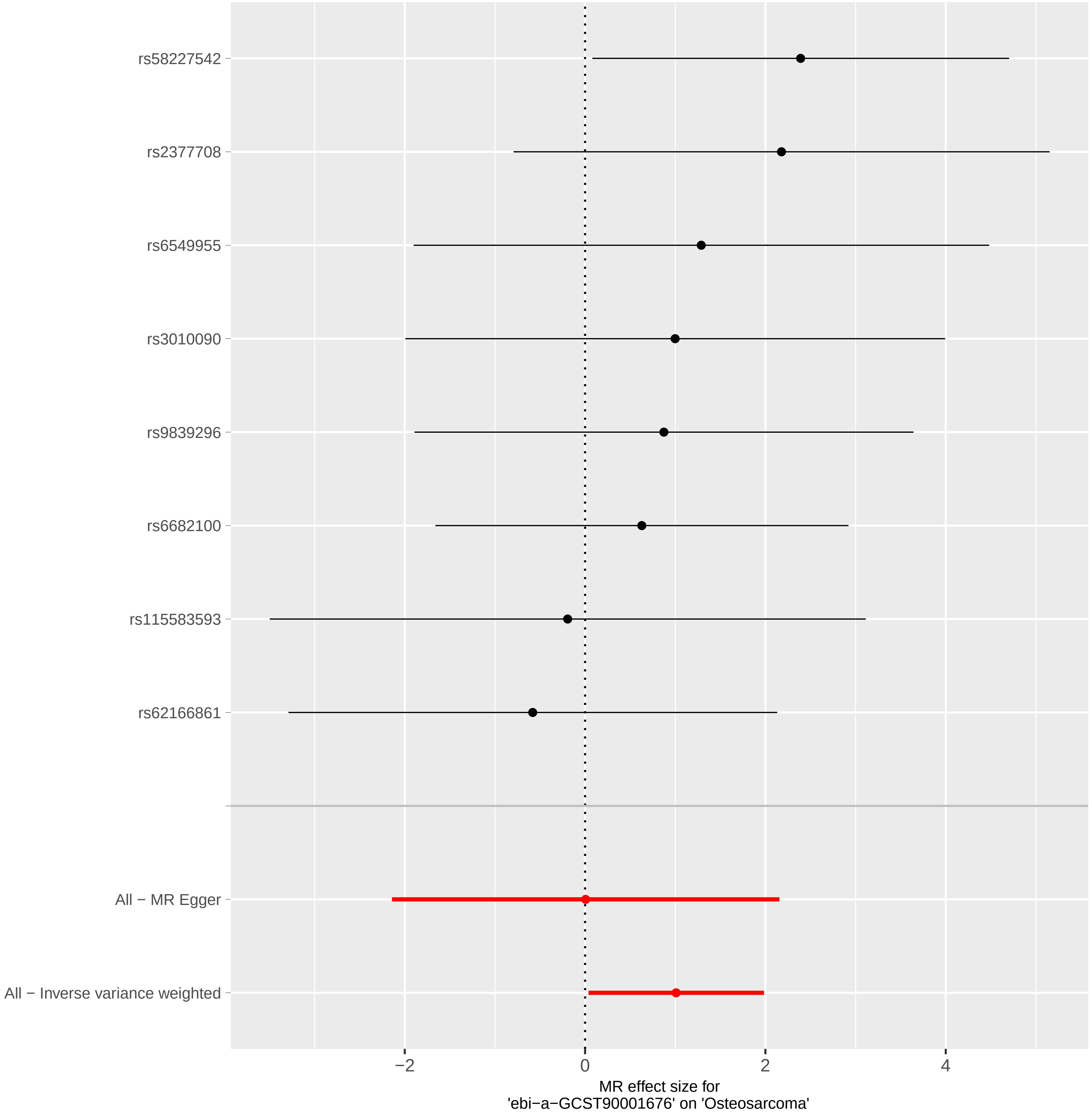
rs58227542
rs2377708
rs6549955
rs3010090
rs9839296
rs6682100
rs115583593
rs62166861
All − MR Egger
All − Inverse variance weighted
−2
0
2
4
MR effect size for
'ebi−a−GCST90001676' on 'Osteosarcoma'

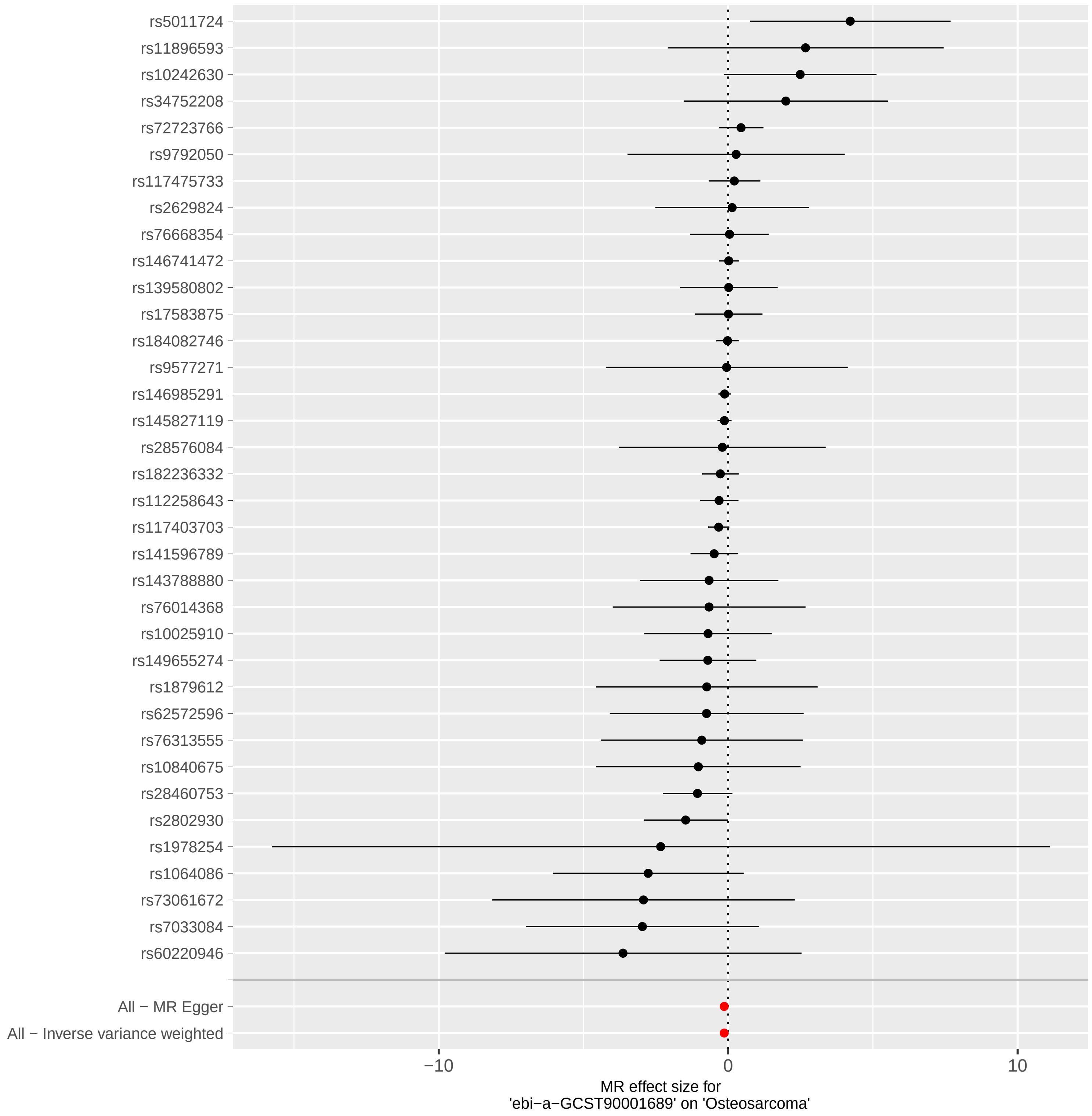
rs5011724
rs11896593
rs10242630
rs34752208
rs72723766
rs9792050
rs117475733
rs2629824
rs76668354
rs146741472
rs139580802
rs17583875
rs184082746
rs9577271
rs146985291
rs145827119
rs28576084
rs182236332
rs112258643
rs117403703
rs141596789
rs143788880
rs76014368
rs10025910
rs149655274
rs1879612
rs62572596
rs76313555
rs10840675
rs28460753
rs2802930
rs1978254
rs1064086
rs73061672
rs7033084
rs60220946
All − MR Egger
All − Inverse variance weighted
−10
0
10
MR effect size for
'ebi−a−GCST90001689' on 'Osteosarcoma'

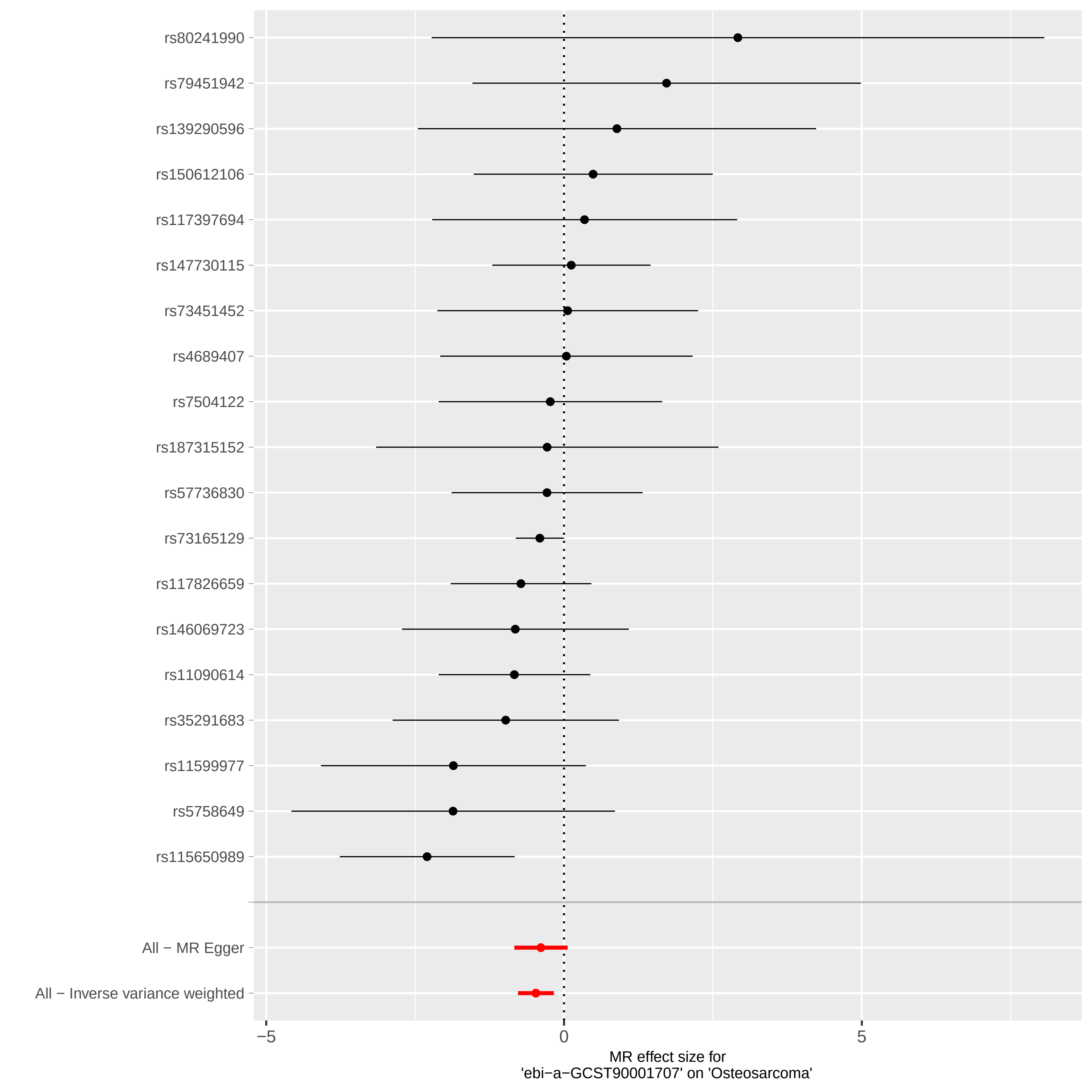
rs80241990
rs79451942
rs139290596
rs150612106
rs117397694
rs147730115
rs73451452
rs4689407
rs7504122
rs187315152
rs57736830
rs73165129
rs117826659
rs146069723
rs11090614
rs35291683
rs11599977
rs5758649
rs115650989
All − MR Egger
All − Inverse variance weighted
−5
0
5
MR effect size for
'ebi−a−GCST90001707' on 'Osteosarcoma'

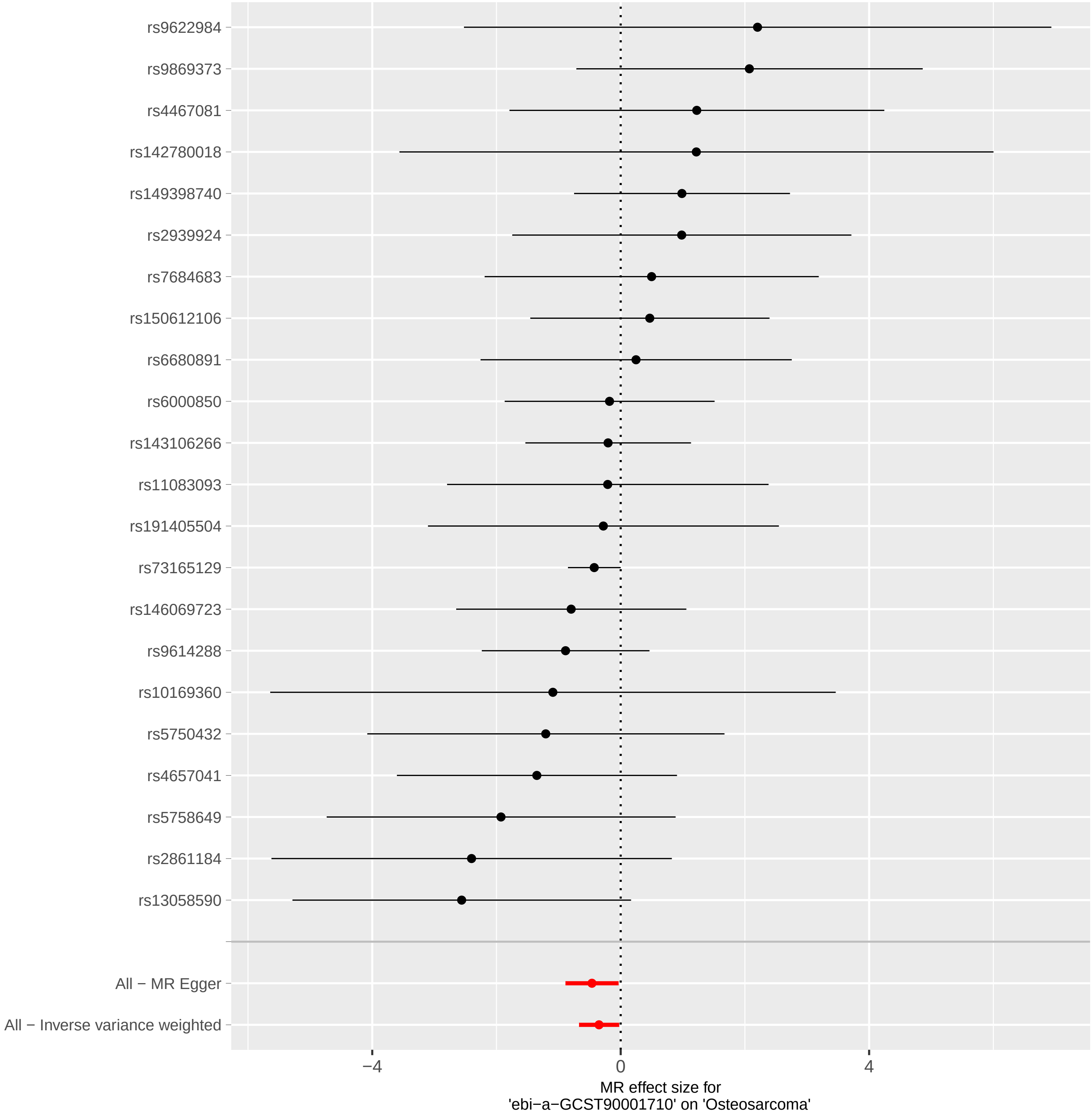
rs9622984
rs9869373
rs4467081
rs142780018
rs149398740
rs2939924
rs7684683
rs150612106
rs6680891
rs6000850
rs143106266
rs11083093
rs191405504
rs73165129
rs146069723
rs9614288
rs10169360
rs5750432
rs4657041
rs5758649
rs2861184
rs13058590
All − MR Egger
All − Inverse variance weighted
−4
0
4
MR effect size for
'ebi−a−GCST90001710' on 'Osteosarcoma'

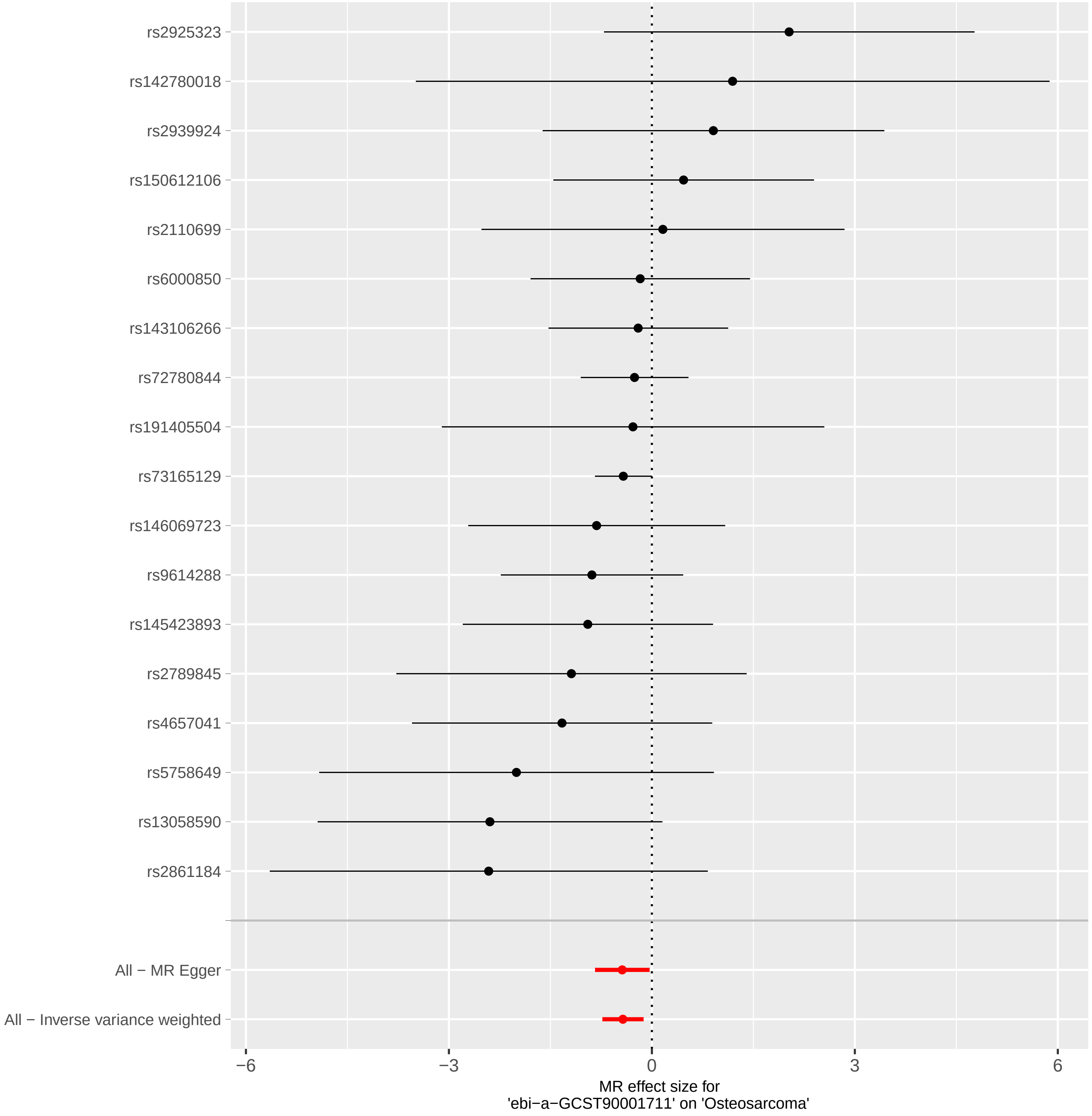
rs2925323
rs142780018
rs2939924
rs150612106
rs2110699
rs6000850
rs143106266
rs72780844
rs191405504
rs73165129
rs146069723
rs9614288
rs145423893
rs2789845
rs4657041
rs5758649
rs13058590
rs2861184
All − MR Egger
All − Inverse variance weighted
−6
−3
0
3
6
MR effect size for
'ebi−a−GCST90001711' on 'Osteosarcoma'

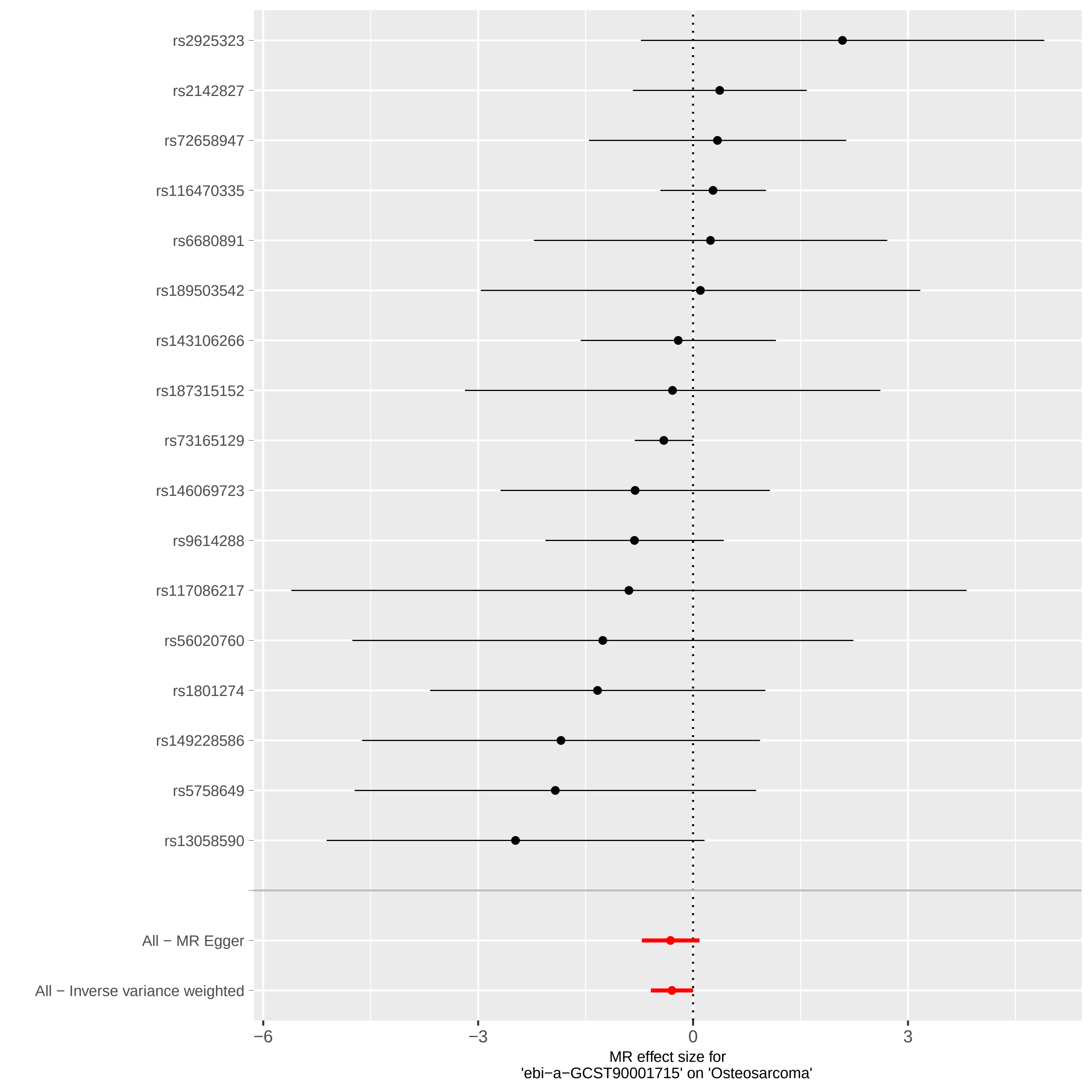

rs2925323
rs2142827
rs72658947
rs116470335
rs6680891
rs189503542
rs143106266
rs187315152
rs73165129
rs146069723
rs9614288
rs117086217
rs56020760
rs1801274
rs149228586
rs5758649
rs13058590
All − MR Egger
All − Inverse variance weighted
−6
−3
0
3
MR effect size for
'ebi−a−GCST90001715' on 'Osteosarcoma'

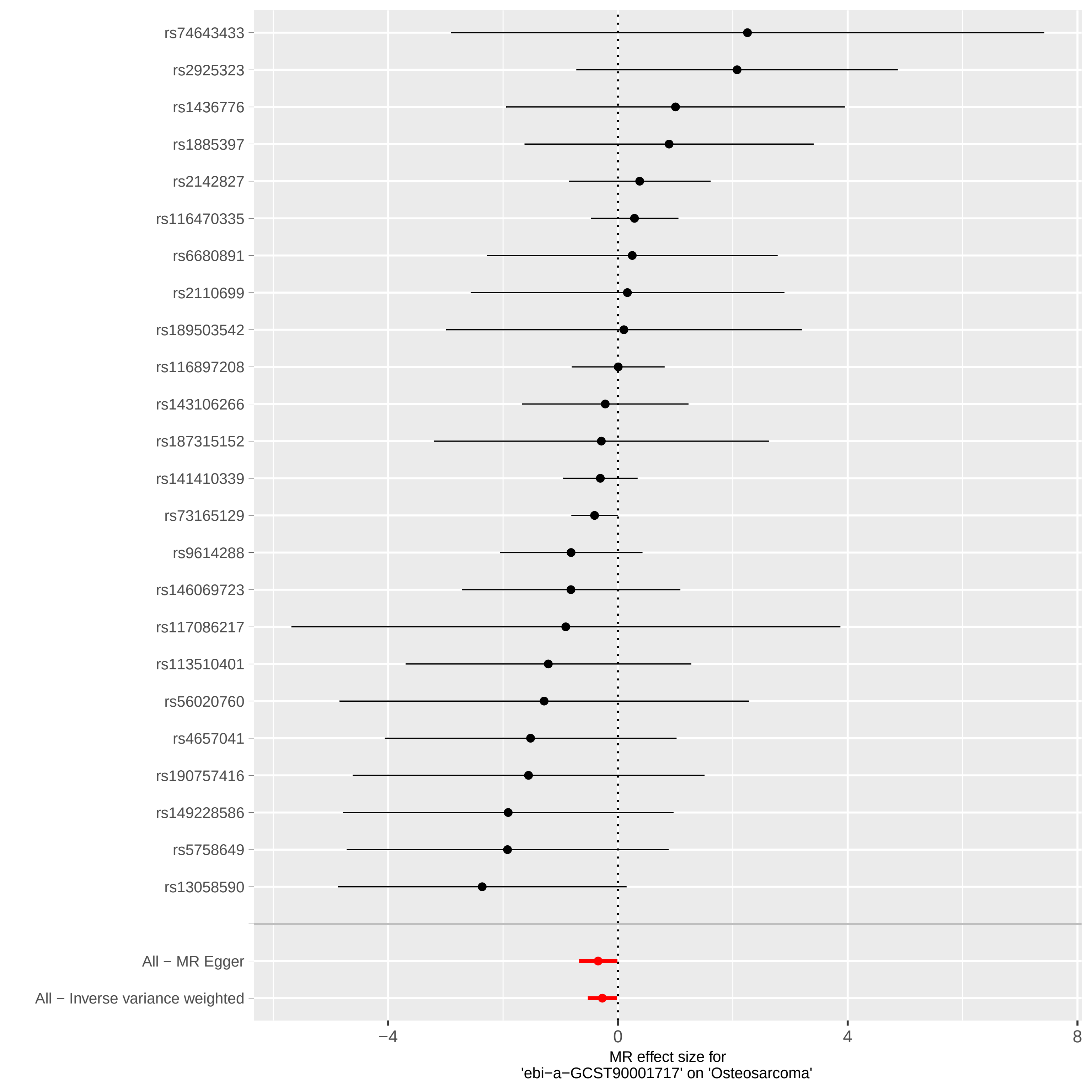
rs74643433
rs2925323
rs1436776
rs1885397
rs2142827
rs116470335
rs6680891
rs2110699
rs189503542
rs116897208
rs143106266
rs187315152
rs141410339
rs73165129
rs9614288
rs146069723
rs117086217
rs113510401
rs56020760
rs4657041
rs190757416
rs149228586
rs5758649
rs13058590
All − MR Egger
All − Inverse variance weighted
−4
0
4
8
MR effect size for
'ebi−a−GCST90001717' on 'Osteosarcoma'

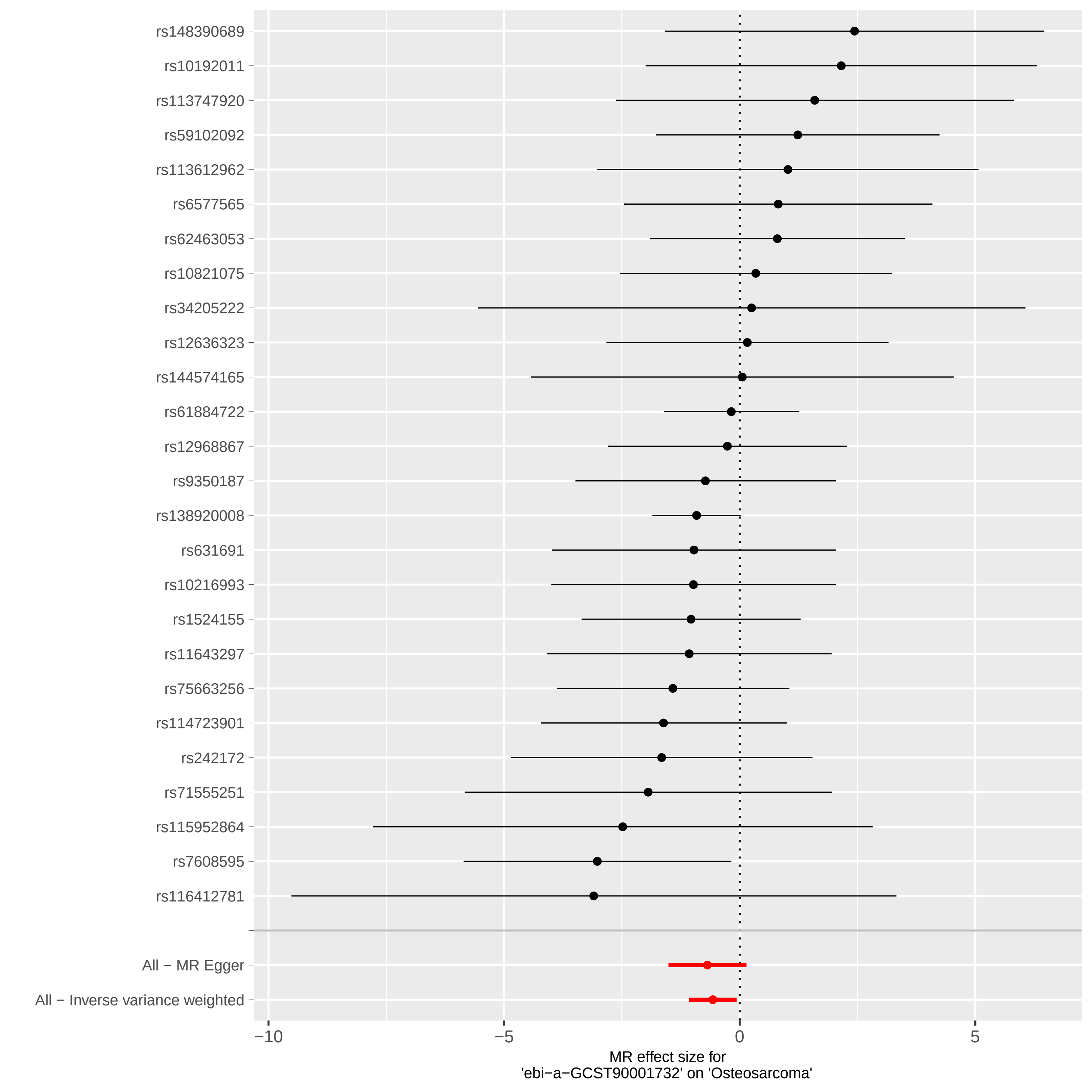

rs148390689
rs10192011
rs113747920
rs59102092
rs113612962
rs6577565
rs62463053
rs10821075
rs34205222
rs12636323
rs144574165
rs61884722
rs12968867
rs9350187
rs138920008
rs631691
rs10216993
rs1524155
rs11643297
rs75663256
rs114723901
rs242172
rs71555251
rs115952864
rs7608595
rs116412781
All − MR Egger
All − Inverse variance weighted
−10
−5
0
5
MR effect size for
'ebi−a−GCST90001732' on 'Osteosarcoma'

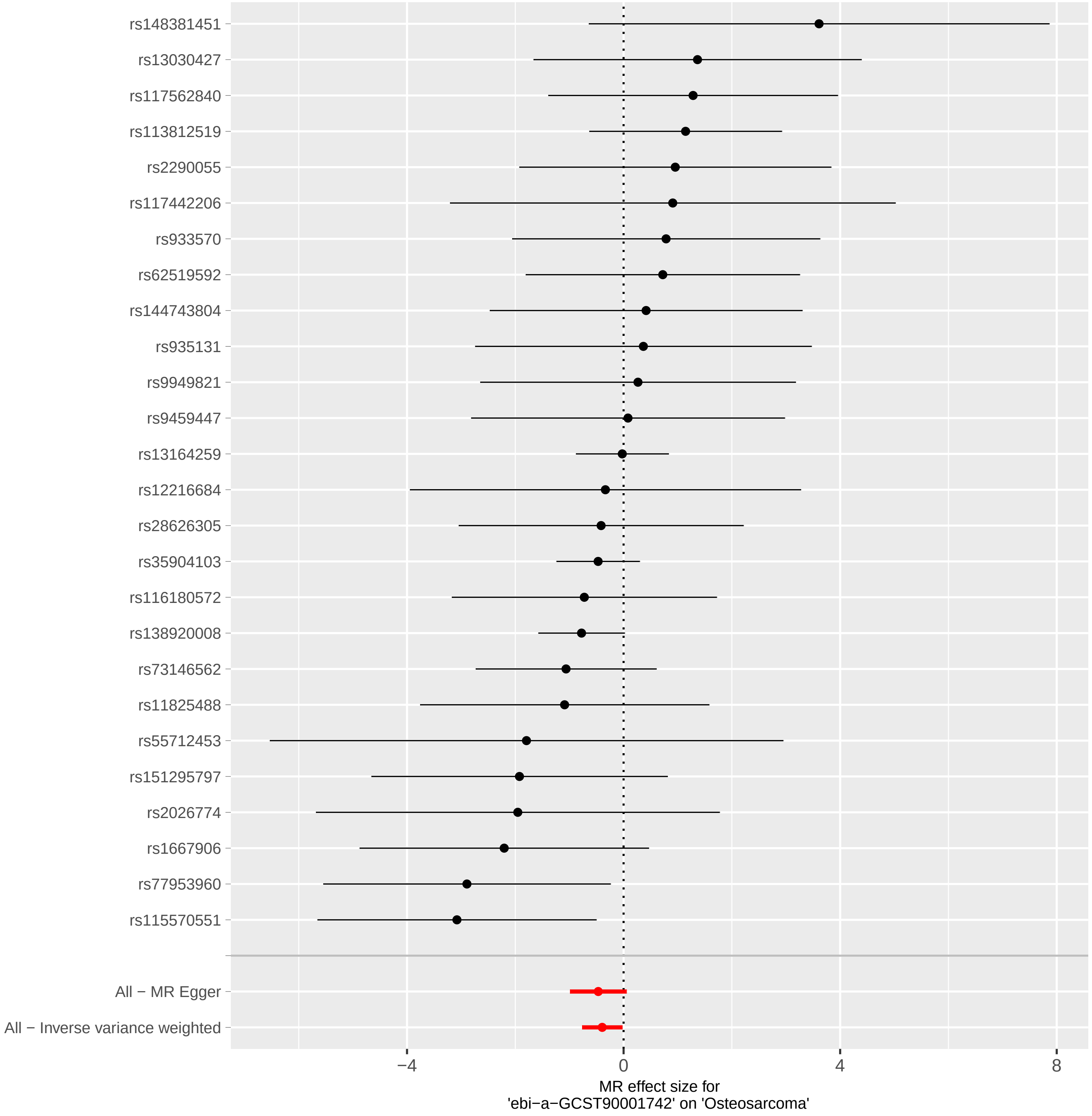
rs148381451
rs13030427
rs117562840
rs113812519
rs2290055
rs117442206
rs933570
rs62519592
rs144743804
rs935131
rs9949821
rs9459447
rs13164259
rs12216684
rs28626305
rs35904103
rs116180572
rs138920008
rs73146562
rs11825488
rs55712453
rs151295797
rs2026774
rs1667906
rs77953960
rs115570551
All − MR Egger
All − Inverse variance weighted
−4
0
4
8
MR effect size for
'ebi−a−GCST90001742' on 'Osteosarcoma'

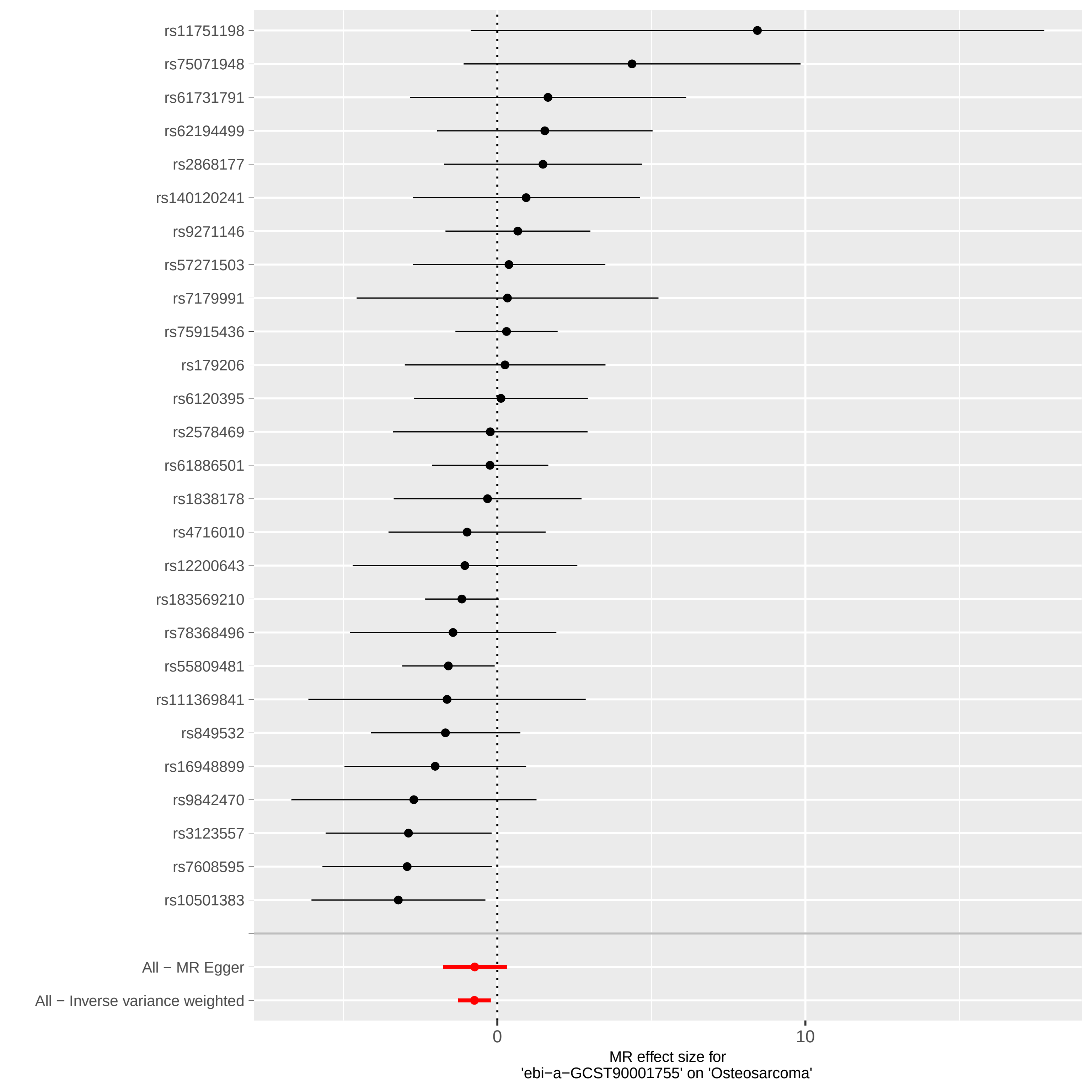
rs11751198
rs75071948
rs61731791
rs62194499
rs2868177
rs140120241
rs9271146
rs57271503
rs7179991
rs75915436
rs179206
rs6120395
rs2578469
rs61886501
rs1838178
rs4716010
rs12200643
rs183569210
rs78368496
rs55809481
rs111369841
rs849532
rs16948899
rs9842470
rs3123557
rs7608595
rs10501383
All − MR Egger
All − Inverse variance weighted
0
10
MR effect size for
'ebi−a−GCST90001755' on 'Osteosarcoma'

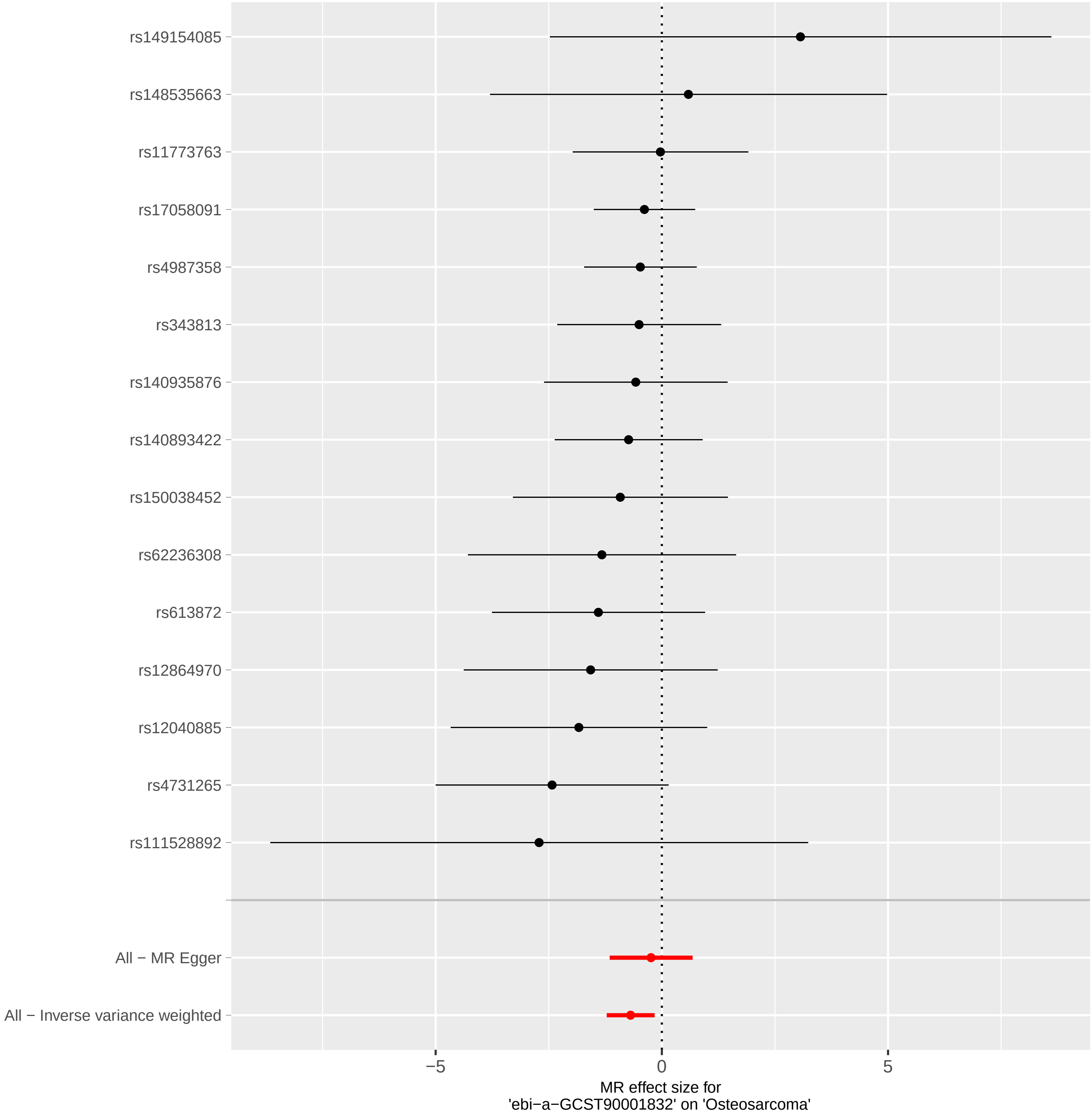
rs149154085
rs148535663
rs11773763
rs17058091
rs4987358
rs343813
rs140935876
rs140893422
rs150038452
rs62236308
rs613872
rs12864970
rs12040885
rs4731265
rs111528892
All − MR Egger
All − Inverse variance weighted
−5
0
5
MR effect size for
'ebi−a−GCST90001832' on 'Osteosarcoma'

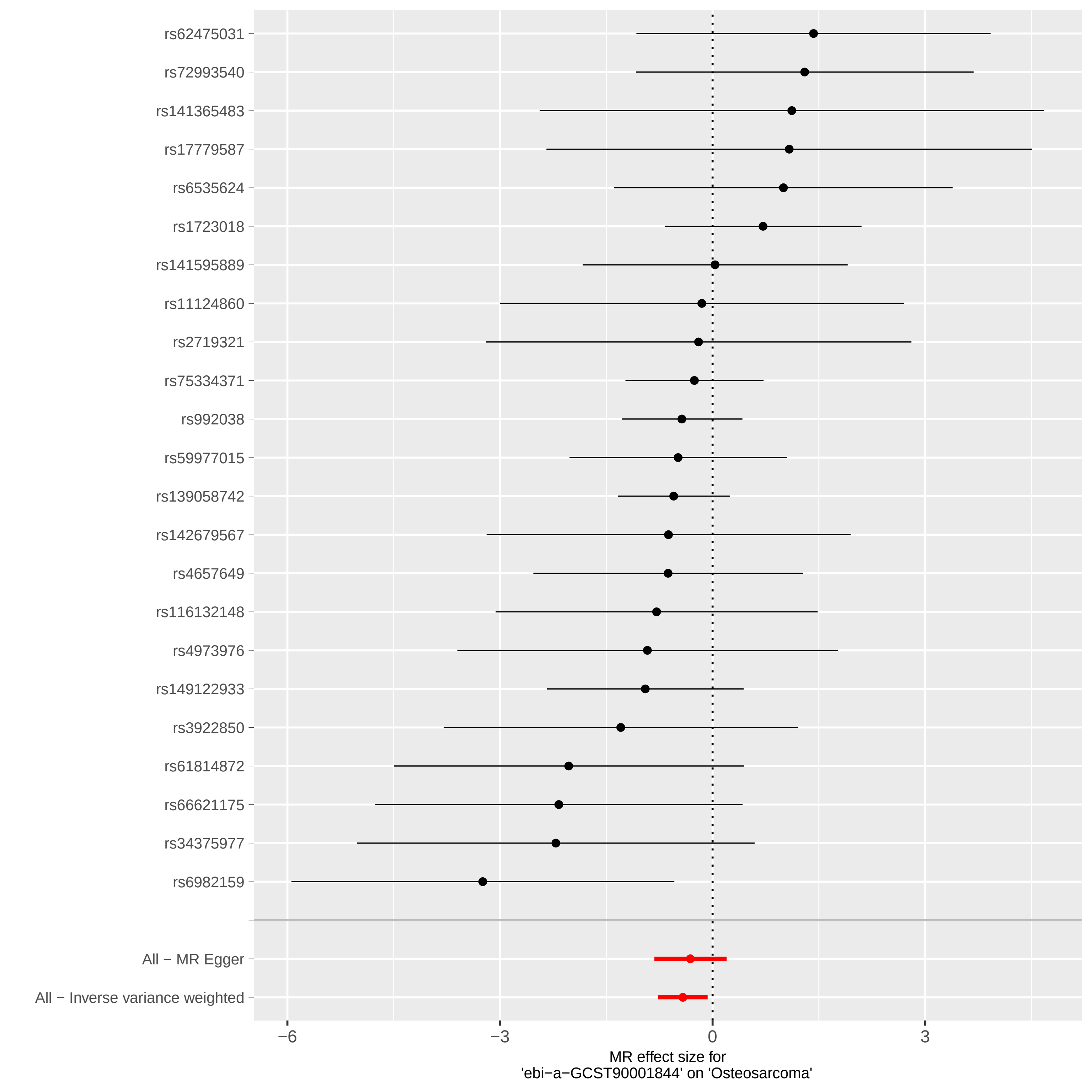
rs62475031
rs72993540
rs141365483
rs17779587
rs6535624
rs1723018
rs141595889
rs11124860
rs2719321
rs75334371
rs992038
rs59977015
rs139058742
rs142679567
rs4657649
rs116132148
rs4973976
rs149122933
rs3922850
rs61814872
rs66621175
rs34375977
rs6982159
All − MR Egger
All − Inverse variance weighted
−6
−3
0
3
MR effect size for
'ebi−a−GCST90001844' on 'Osteosarcoma'

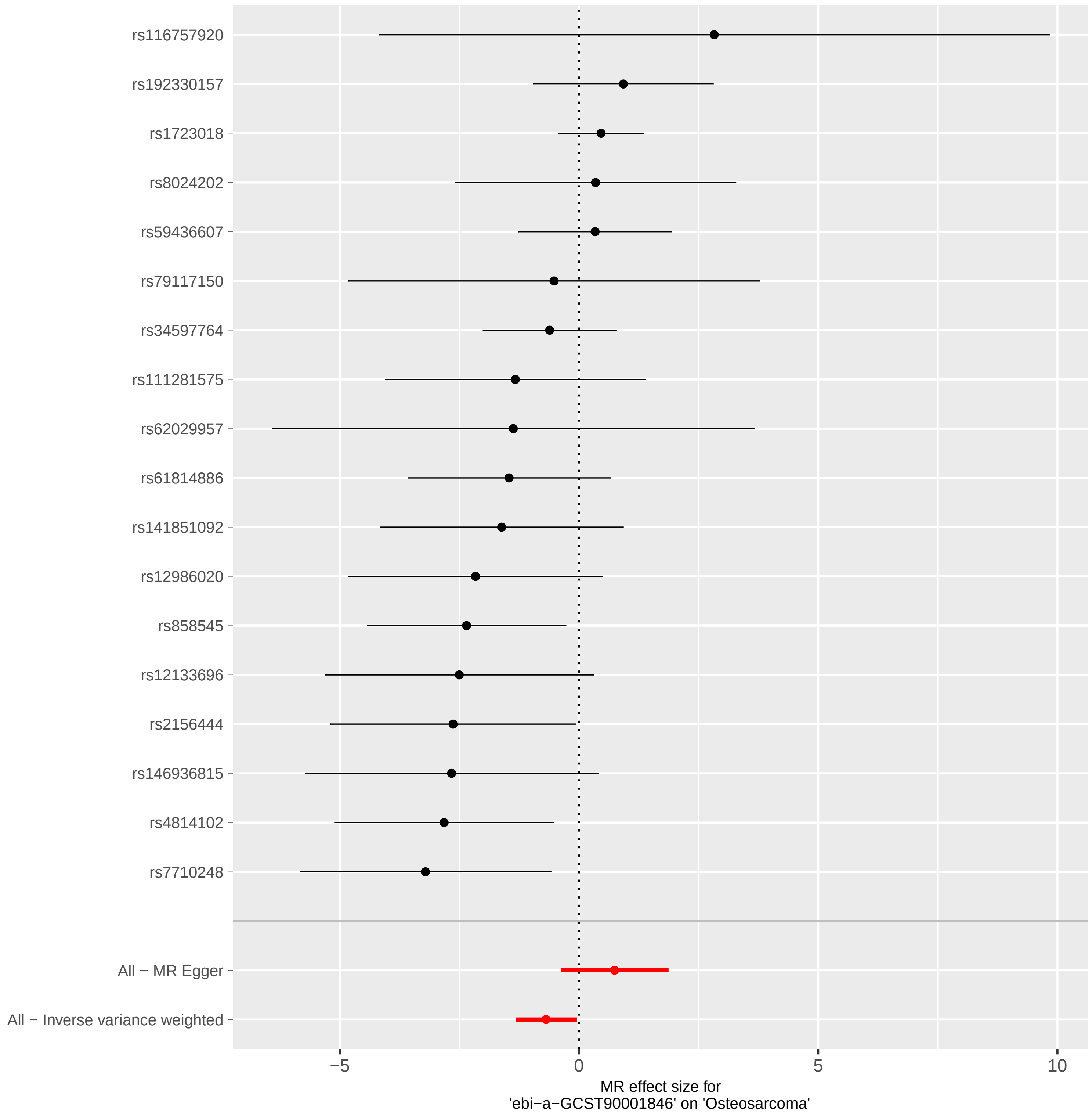
rs116757920
rs192330157
rs1723018
rs8024202
rs59436607
rs79117150
rs34597764
rs111281575
rs62029957
rs61814886
rs141851092
rs12986020
rs858545
rs12133696
rs2156444
rs146936815
rs4814102
rs7710248
All − MR Egger
All − Inverse variance weighted
−5
0
5
10
MR effect size for
'ebi−a−GCST90001846' on 'Osteosarcoma'

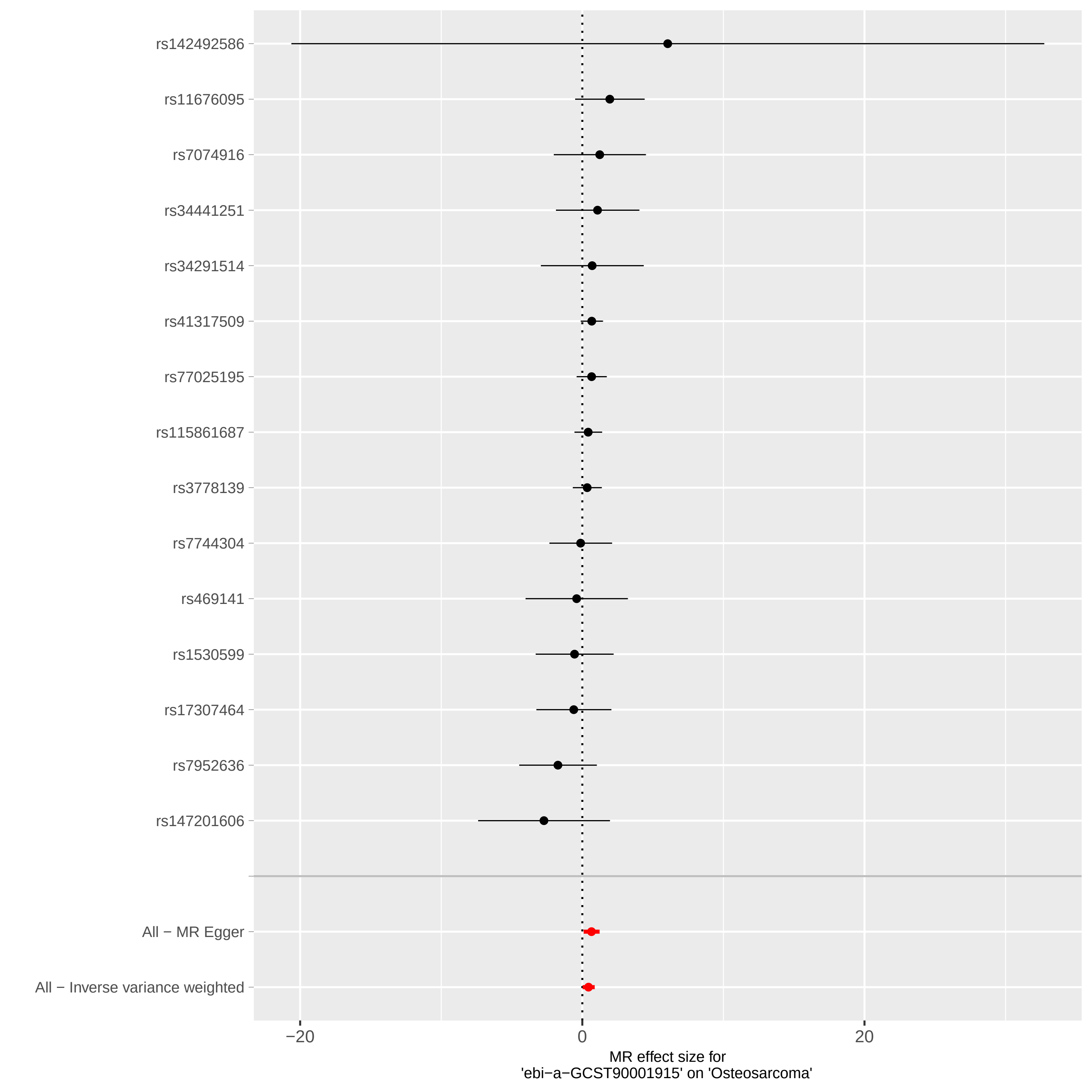
rs142492586
rs11676095
rs7074916
rs34441251
rs34291514
rs41317509
rs77025195
rs115861687
rs3778139
rs7744304
rs469141
rs1530599
rs17307464
rs7952636
rs147201606
All − MR Egger
All − Inverse variance weighted
−20
0
20
MR effect size for
'ebi−a−GCST90001915' on 'Osteosarcoma'

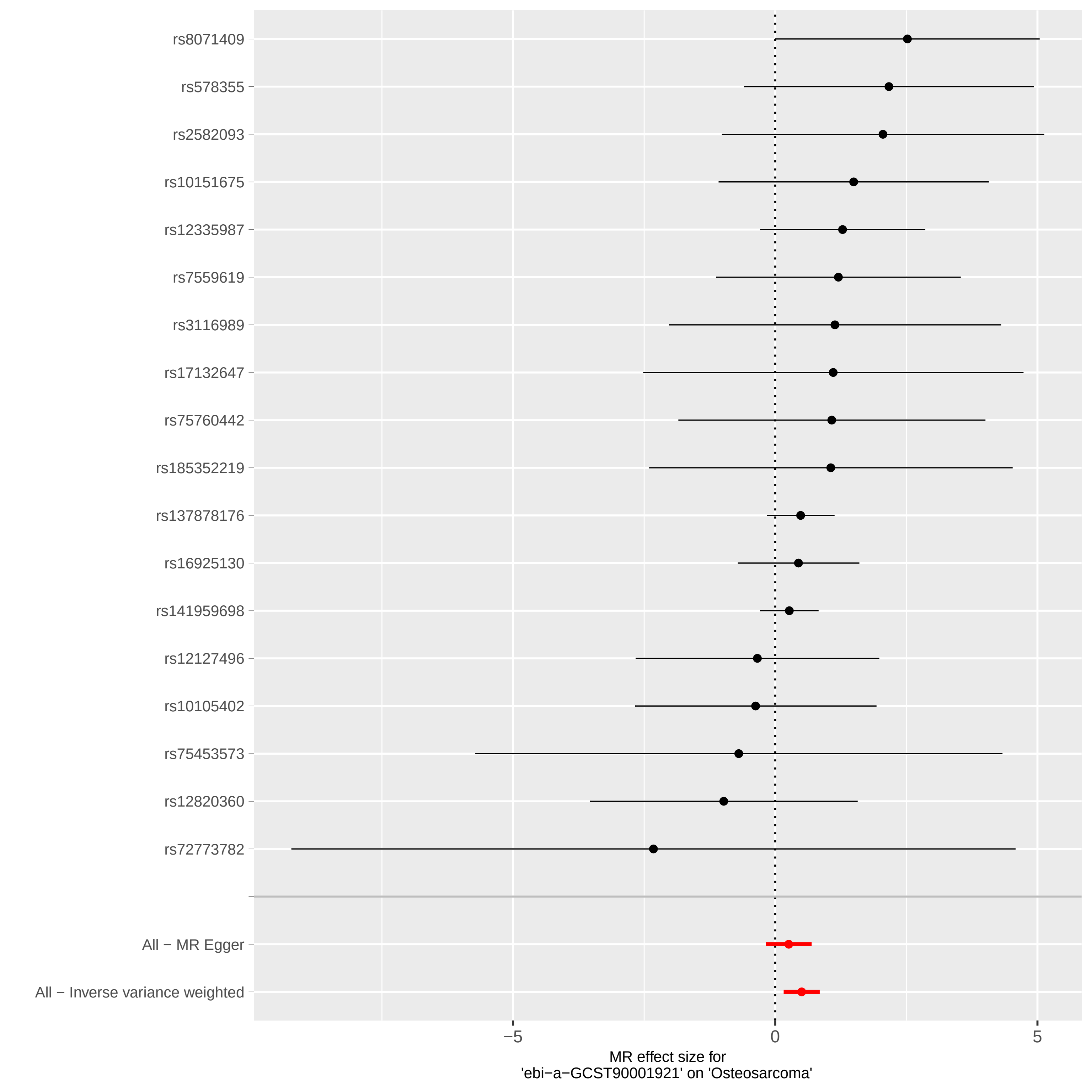
rs8071409
rs578355
rs2582093
rs10151675
rs12335987
rs7559619
rs3116989
rs17132647
rs75760442
rs185352219
rs137878176
rs16925130
rs141959698
rs12127496
rs10105402
rs75453573
rs12820360
rs72773782
All − MR Egger
All − Inverse variance weighted
−5
0
5
MR effect size for
'ebi−a−GCST90001921' on 'Osteosarcoma'

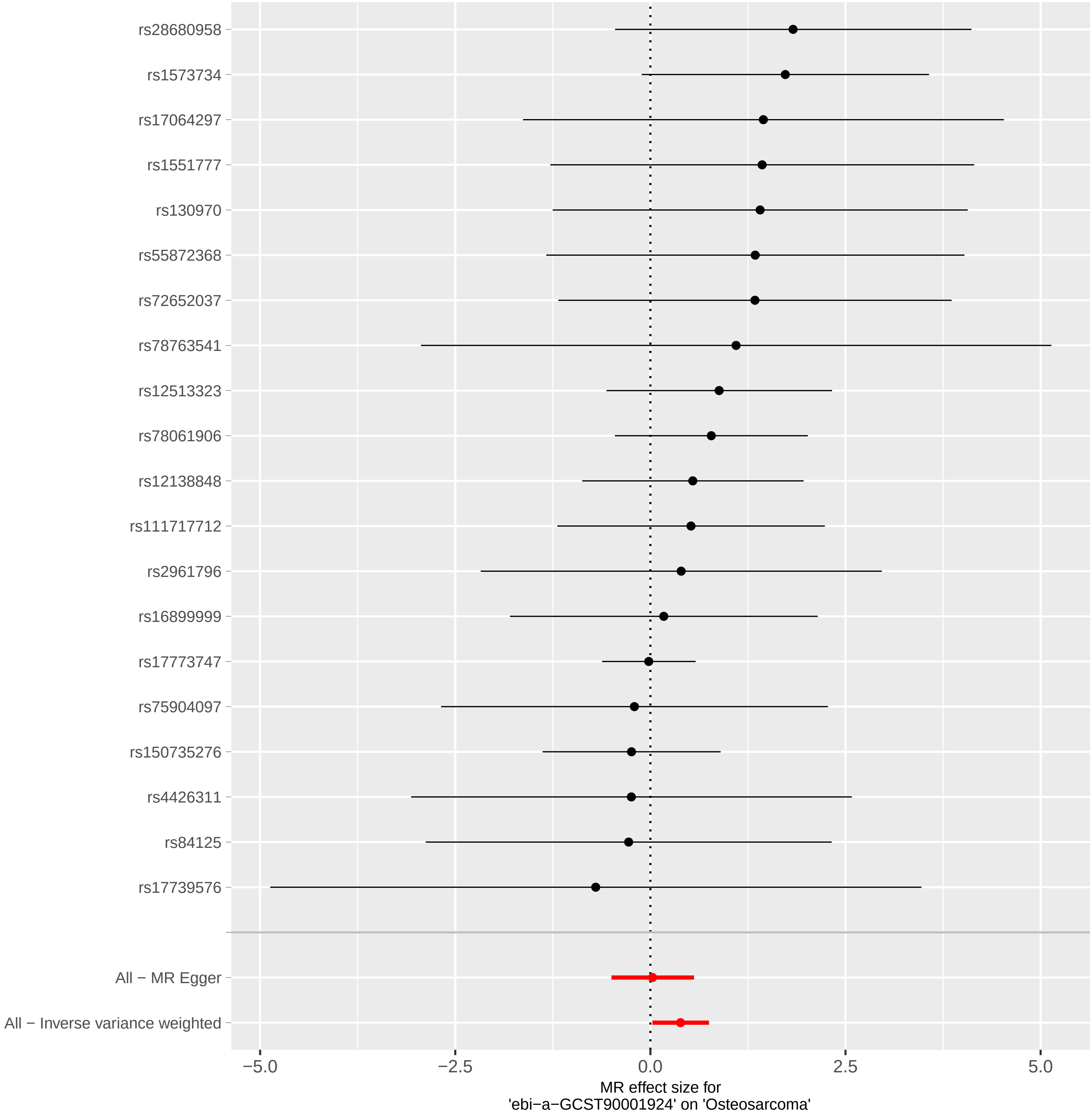
rs28680958
rs1573734
rs17064297
rs1551777
rs130970
rs55872368
rs72652037
rs78763541
rs12513323
rs78061906
rs12138848
rs111717712
rs2961796
rs16899999
rs17773747
rs75904097
rs150735276
rs4426311
rs84125
rs17739576
All − MR Egger
All − Inverse variance weighted
−5.0
−2.5
0.0
2.5
5.0
MR effect size for
'ebi−a−GCST90001924' on 'Osteosarcoma'

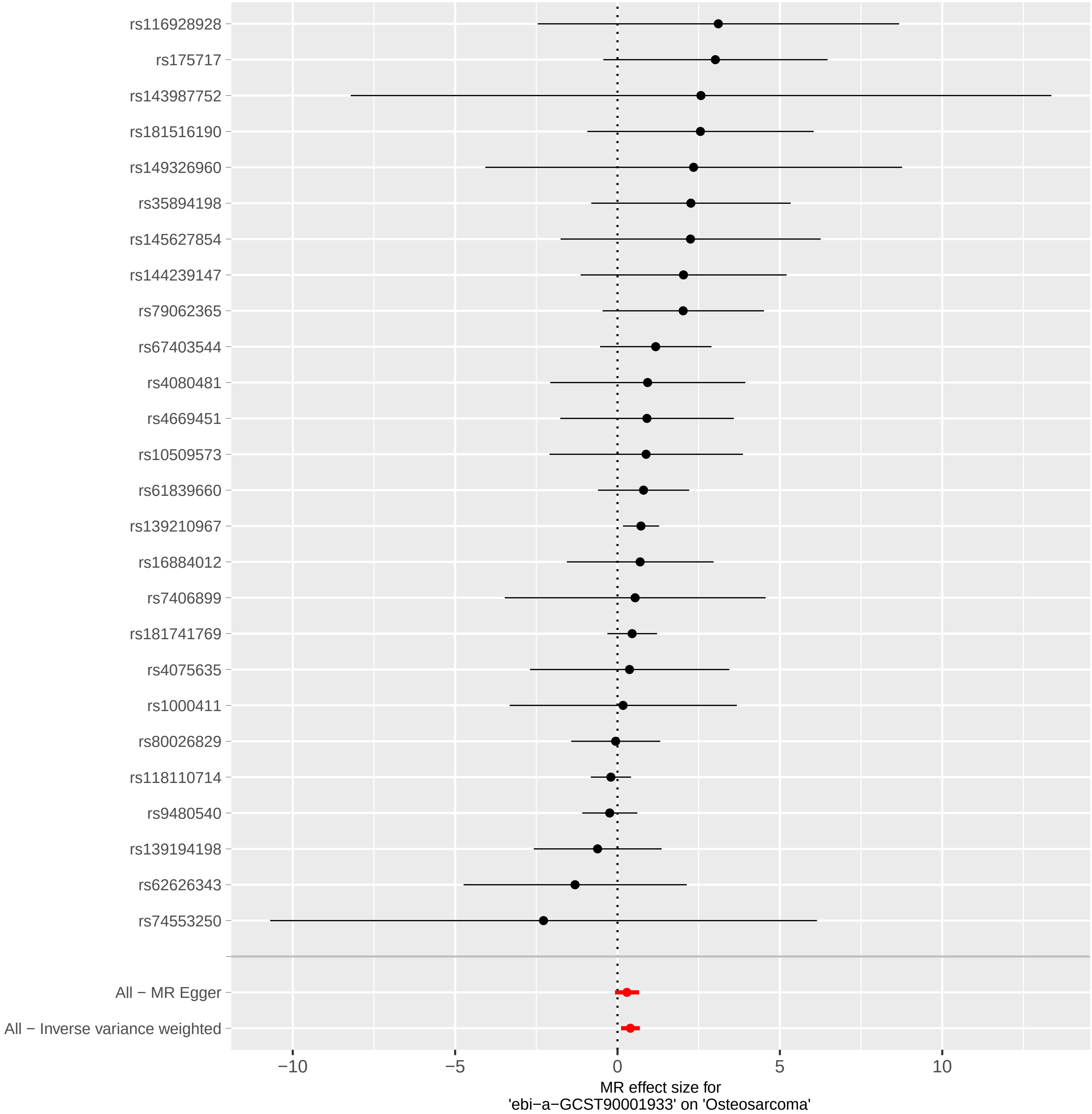
rs116928928
rs175717
rs143987752
rs181516190
rs149326960
rs35894198
rs145627854
rs144239147
rs79062365
rs67403544
rs4080481
rs4669451
rs10509573
rs61839660
rs139210967
rs16884012
rs7406899
rs181741769
rs4075635
rs1000411
rs80026829
rs118110714
rs9480540
rs139194198
rs62626343
rs74553250
All − MR Egger
All − Inverse variance weighted
−10
−5
0
5
10
MR effect size for
'ebi−a−GCST90001933' on 'Osteosarcoma'

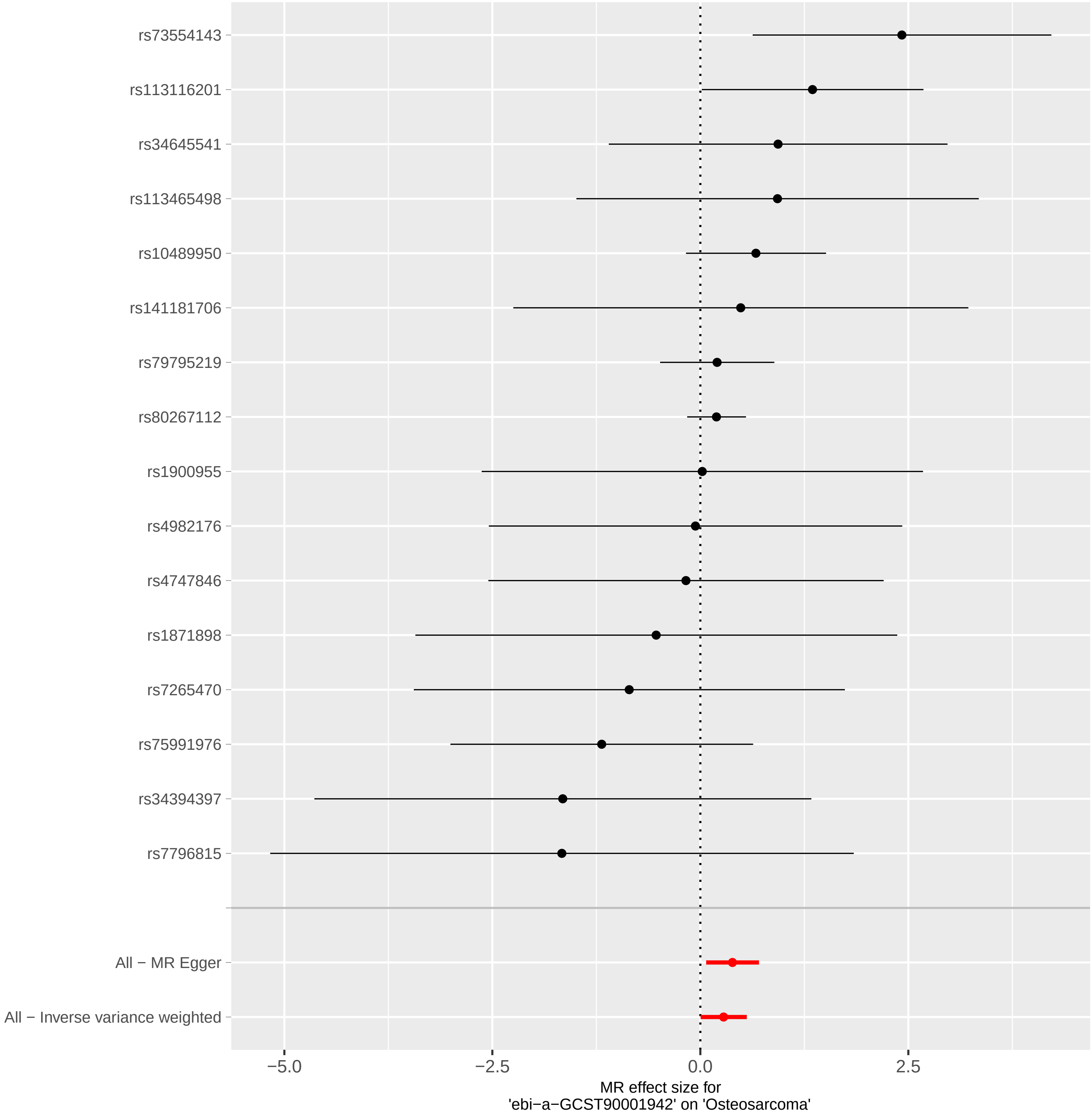
rs73554143
rs113116201
rs34645541
rs113465498
rs10489950
rs141181706
rs79795219
rs80267112
rs1900955
rs4982176
rs4747846
rs1871898
rs7265470
rs75991976
rs34394397
rs7796815
All − MR Egger
All − Inverse variance weighted
−5.0
−2.5
0.0
2.5
MR effect size for
'ebi−a−GCST90001942' on 'Osteosarcoma'

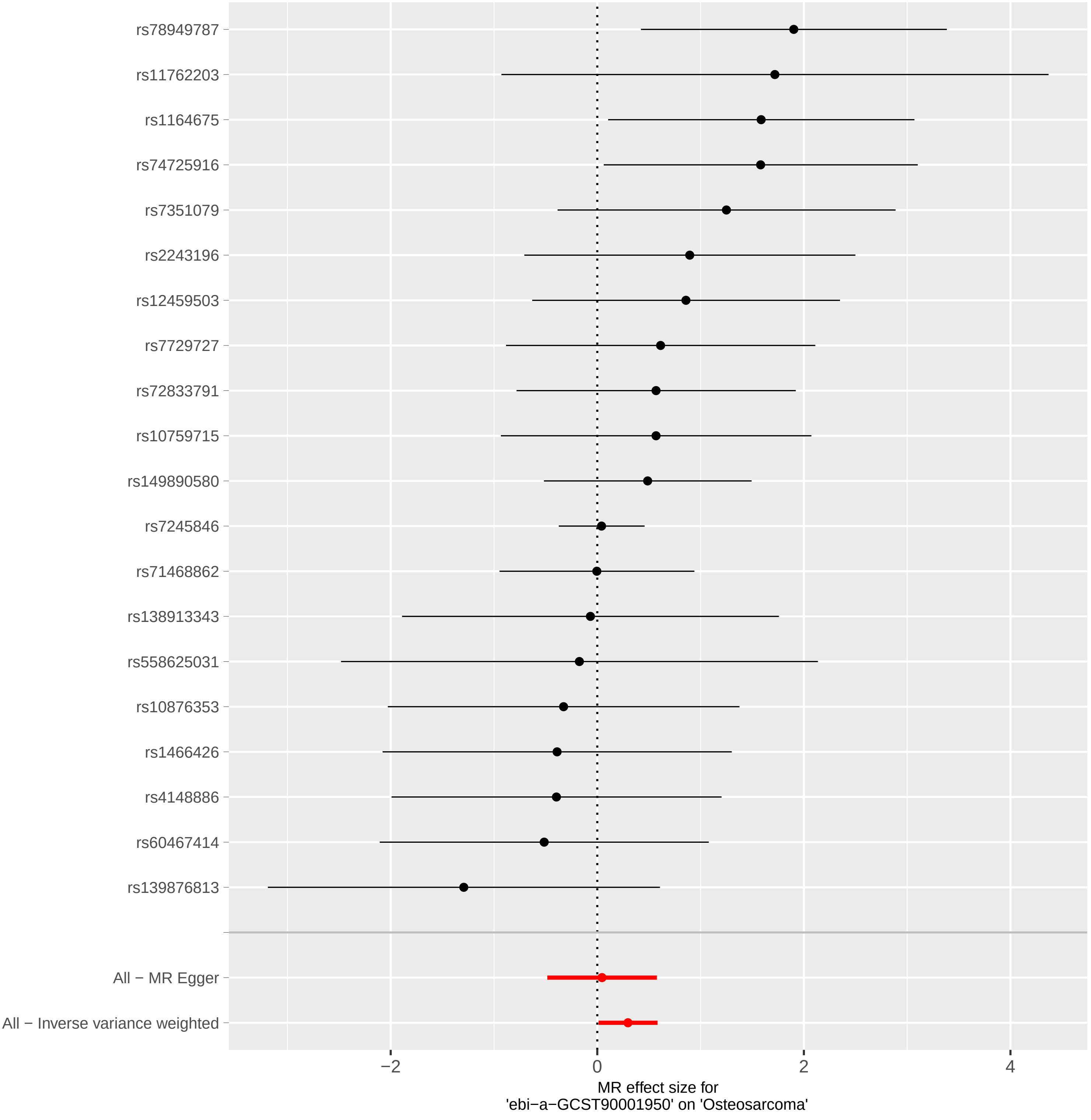
rs78949787
rs11762203
rs1164675
rs74725916
rs7351079
rs2243196
rs12459503
rs7729727
rs72833791
rs10759715
rs149890580
rs7245846
rs71468862
rs138913343
rs558625031
rs10876353
rs1466426
rs4148886
rs60467414
rs139876813
All − MR Egger
All − Inverse variance weighted
−2
0
2
4
MR effect size for
'ebi−a−GCST90001950' on 'Osteosarcoma'

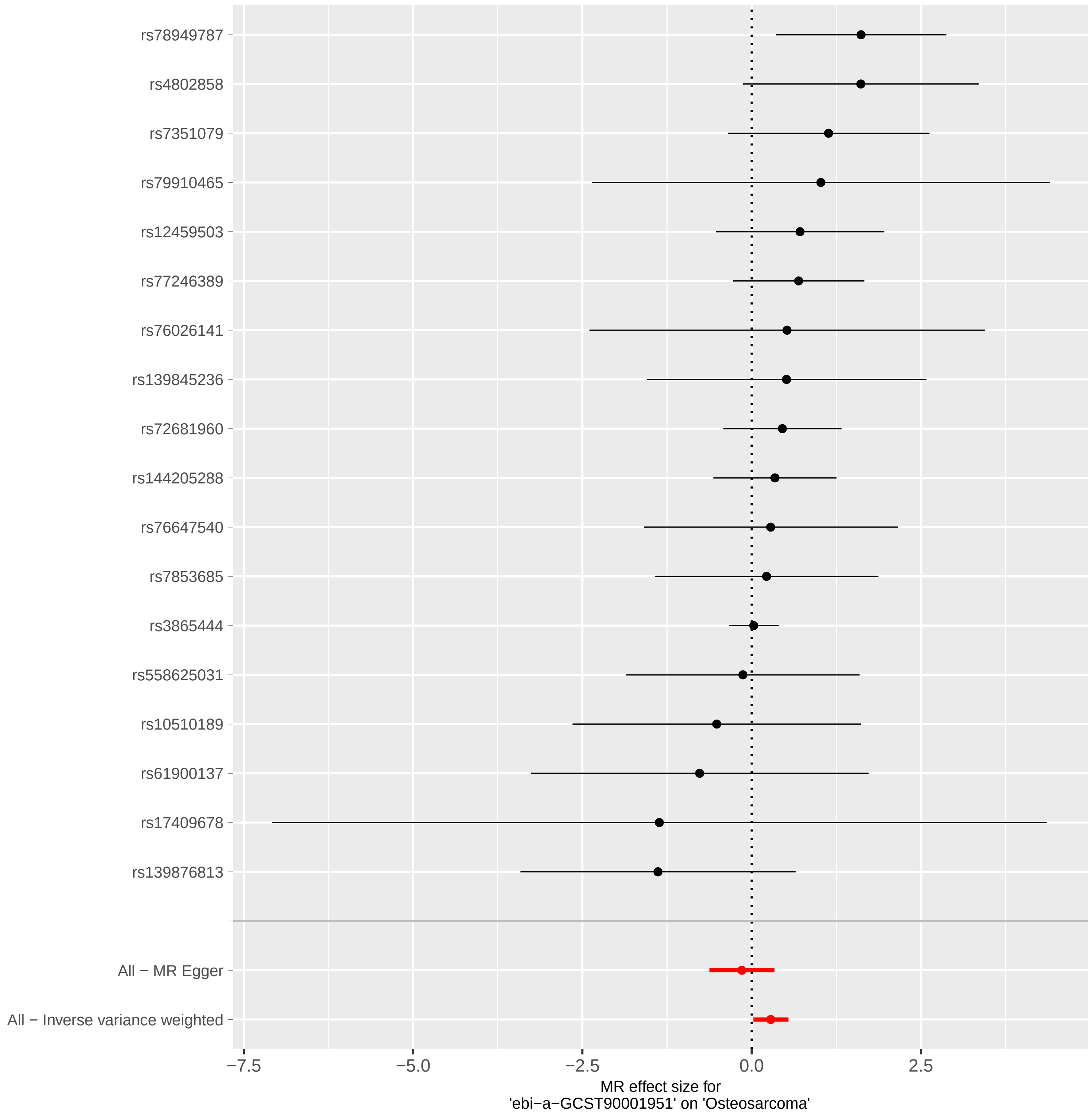
rs78949787
rs4802858
rs7351079
rs79910465
rs12459503
rs77246389
rs76026141
rs139845236
rs72681960
rs144205288
rs76647540
rs7853685
rs3865444
rs558625031
rs10510189
rs61900137
rs17409678
rs139876813
All − MR Egger
All − Inverse variance weighted
−7.5
−5.0
−2.5
0.0
2.5
MR effect size for
'ebi−a−GCST90001951' on 'Osteosarcoma'

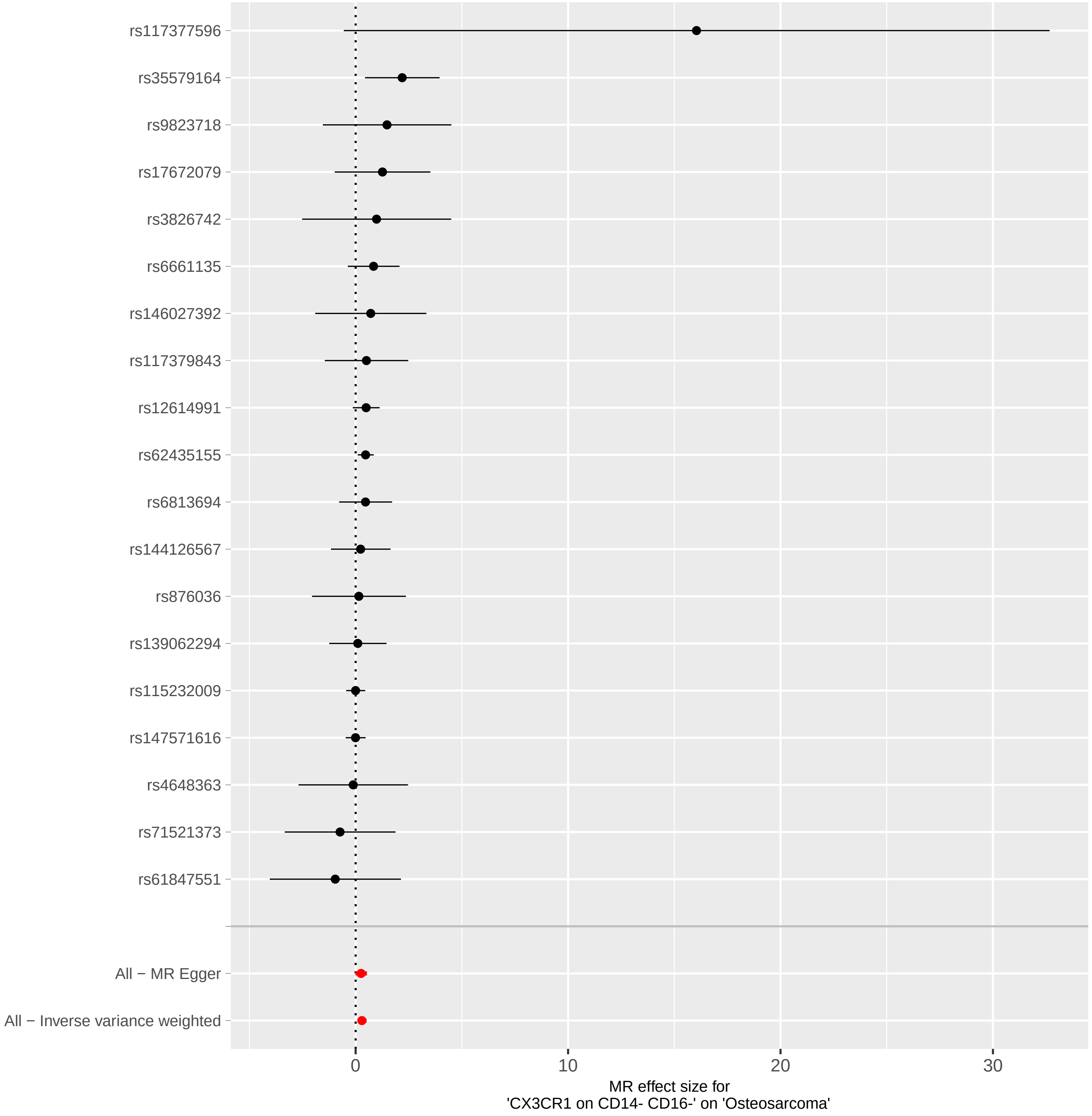

rs117377596
rs35579164
rs9823718
rs17672079
rs3826742
rs6661135
rs146027392
rs117379843
rs12614991
rs62435155
rs6813694
rs144126567
rs876036
rs139062294
rs115232009
rs147571616
rs4648363
rs71521373
rs61847551
All − MR Egger
All − Inverse variance weighted
0
10
20
30
MR effect size for
'CX3CR1 on CD14- CD16-' on 'Osteosarcoma'

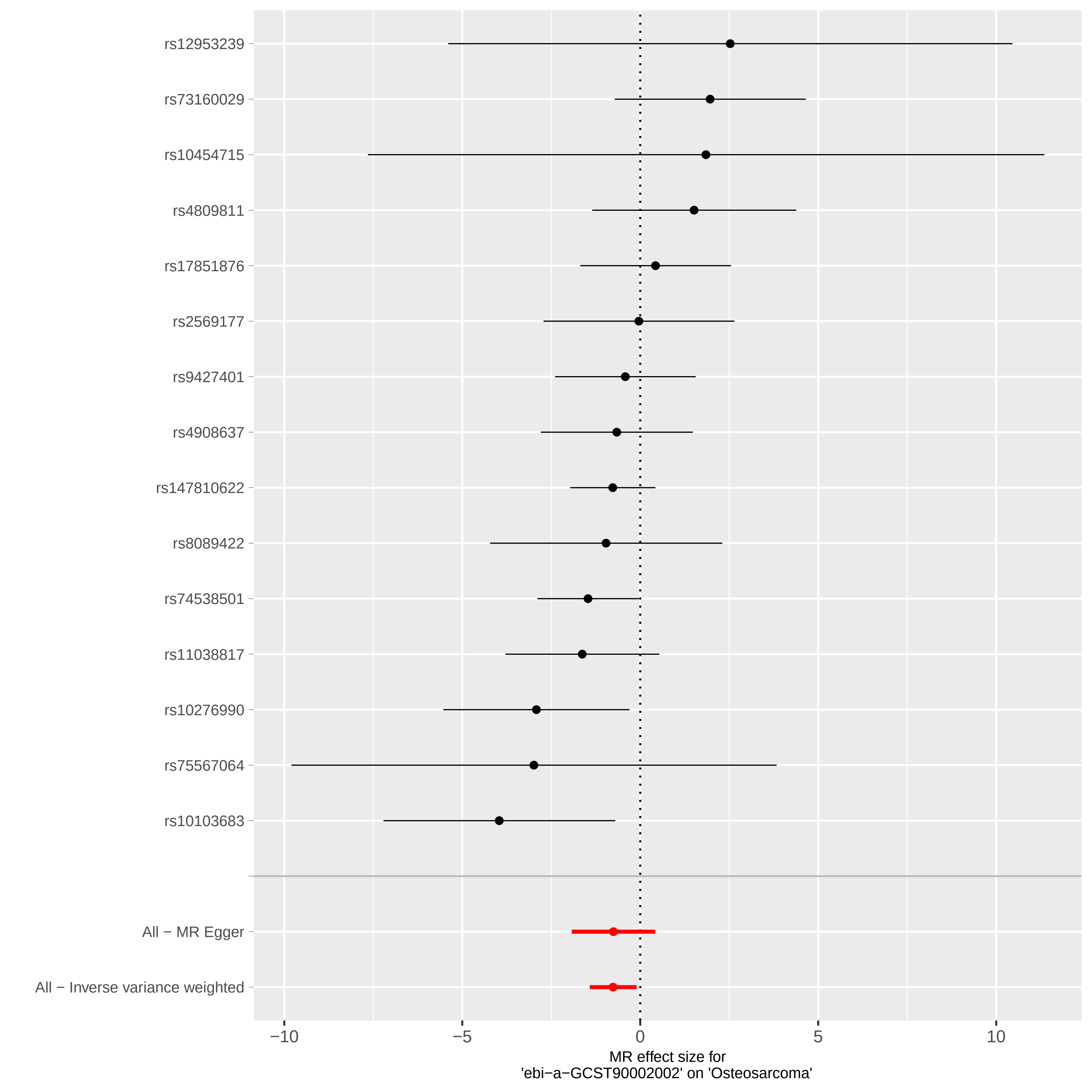

rs12953239
rs73160029
rs10454715
rs4809811
rs17851876
rs2569177
rs9427401
rs4908637
rs147810622
rs8089422
rs74538501
rs11038817
rs10276990
rs75567064
rs10103683
All − MR Egger
All − Inverse variance weighted
−10
−5
0
5
10
MR effect size for
'ebi−a−GCST90002002' on 'Osteosarcoma'

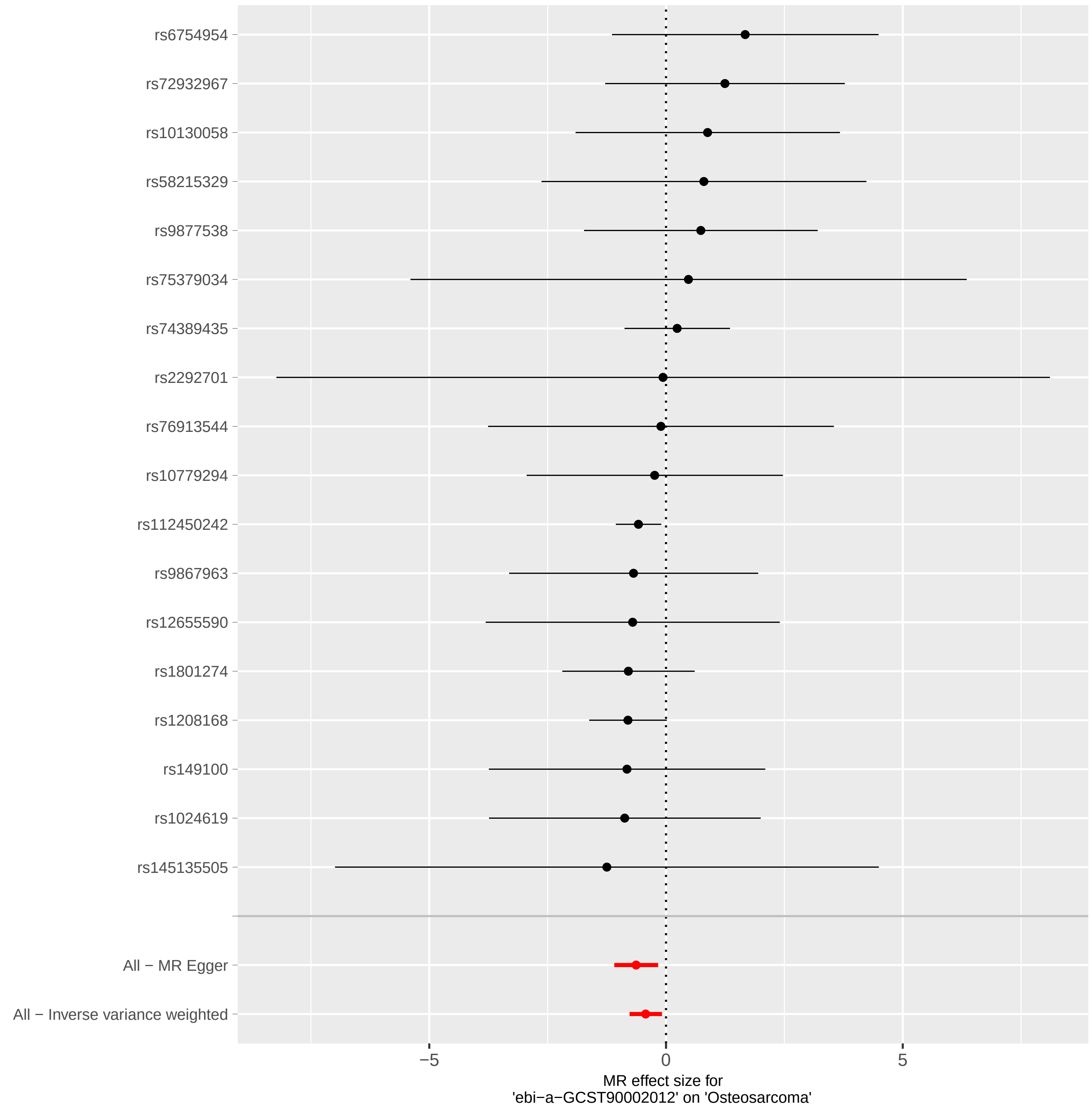
rs6754954
rs72932967
rs10130058
rs58215329
rs9877538
rs75379034
rs74389435
rs2292701
rs76913544
rs10779294
rs112450242
rs9867963
rs12655590
rs1801274
rs1208168
rs149100
rs1024619
rs145135505
All − MR Egger
All − Inverse variance weighted
−5
0
5
MR effect size for
'ebi−a−GCST90002012' on 'Osteosarcoma'

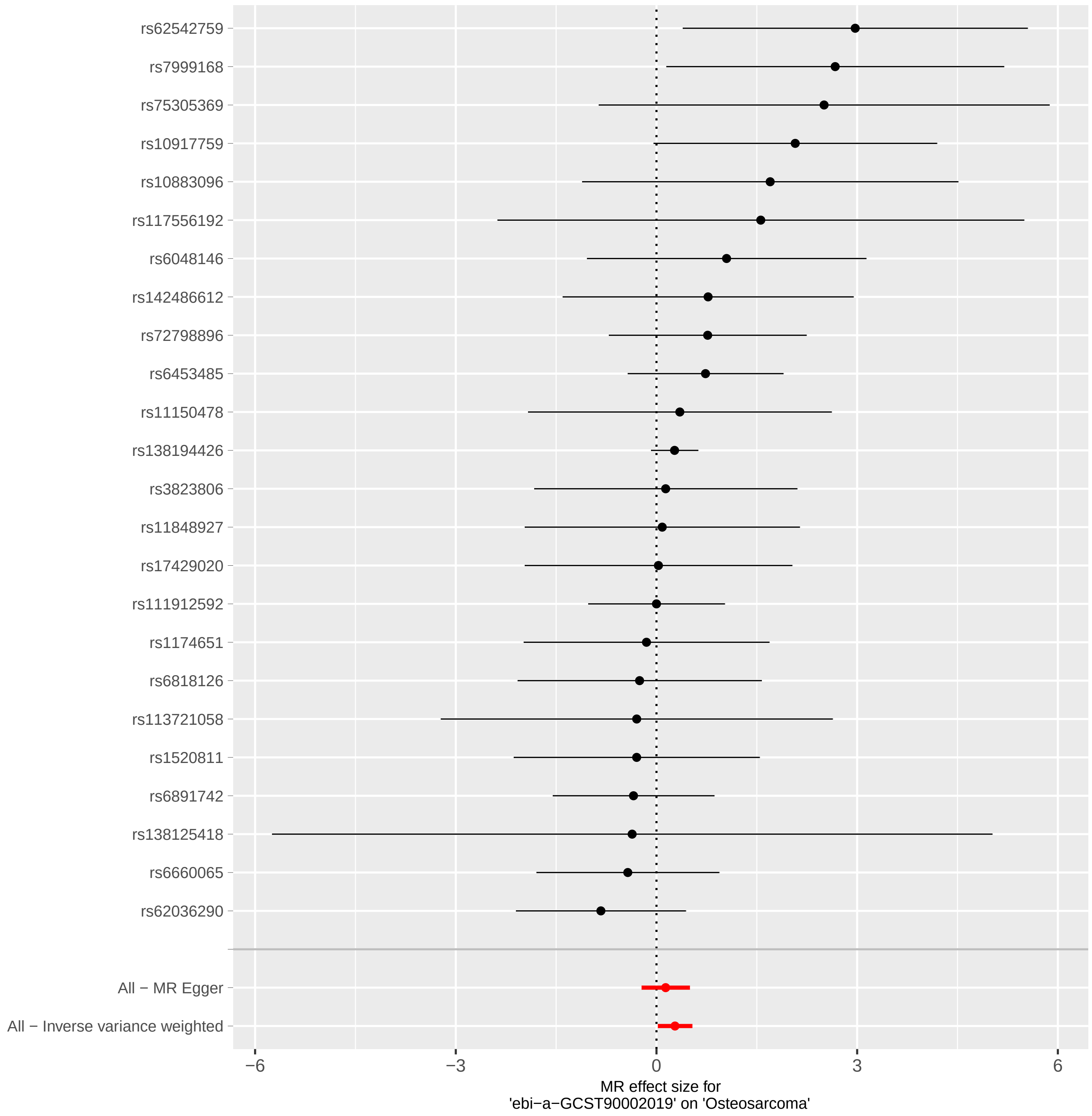
rs62542759
rs7999168
rs75305369
rs10917759
rs10883096
rs117556192
rs6048146
rs142486612
rs72798896
rs6453485
rs11150478
rs138194426
rs3823806
rs11848927
rs17429020
rs111912592
rs1174651
rs6818126
rs113721058
rs1520811
rs6891742
rs138125418
rs6660065
rs62036290
All − MR Egger
All − Inverse variance weighted
−6
−3
0
3
6
MR effect size for
'ebi−a−GCST90002019' on 'Osteosarcoma'

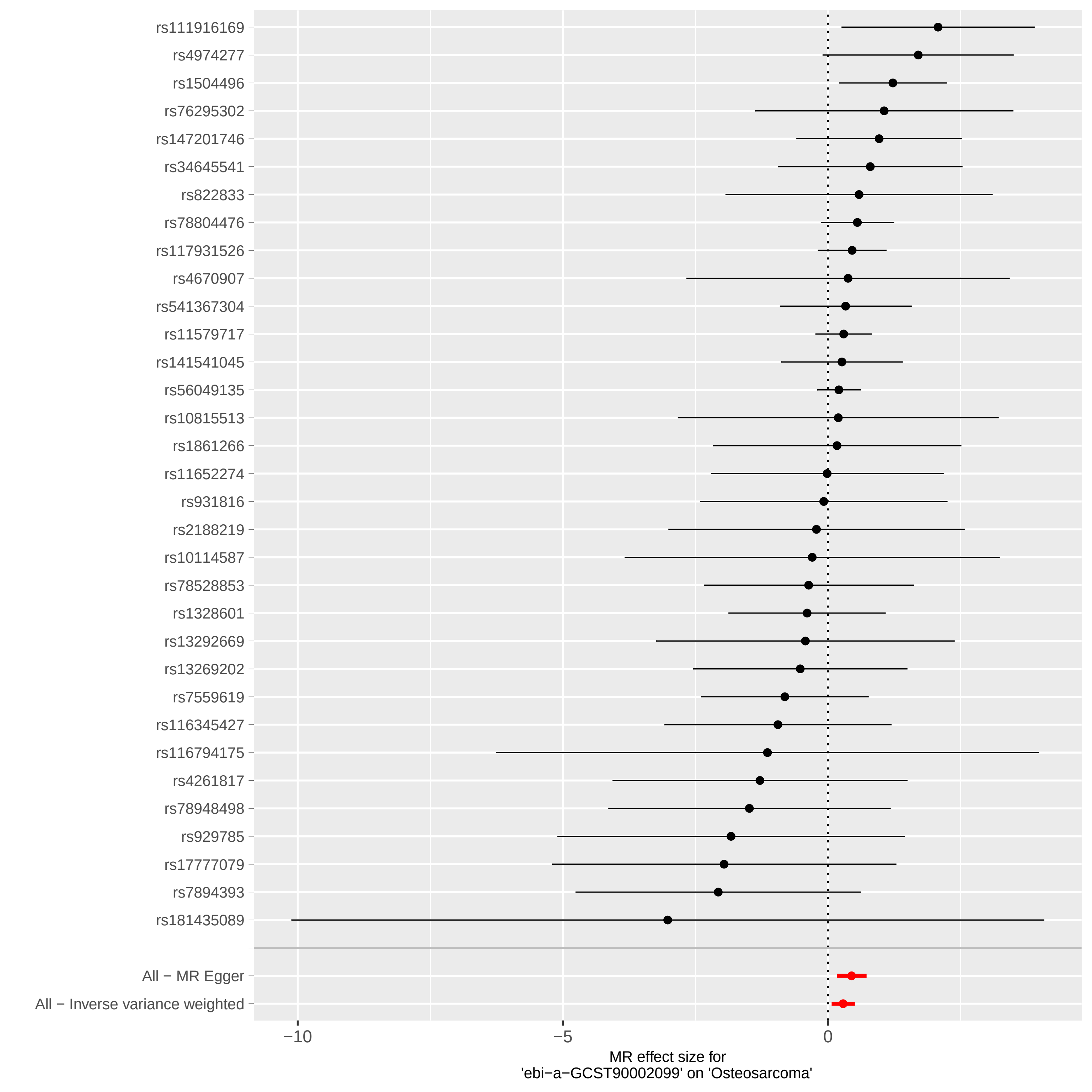

rs111916169
rs4974277
rs1504496
rs76295302
rs147201746
rs34645541
rs822833
rs78804476
rs117931526
rs4670907
rs541367304
rs11579717
rs141541045
rs56049135
rs10815513
rs1861266
rs11652274
rs931816
rs2188219
rs10114587
rs78528853
rs1328601
rs13292669
rs13269202
rs7559619
rs116345427
rs116794175
rs4261817
rs78948498
rs929785
rs17777079
rs7894393
rs181435089
All − MR Egger
All − Inverse variance weighted
−10
−5
0
MR effect size for
'ebi−a−GCST90002099' on 'Osteosarcoma'

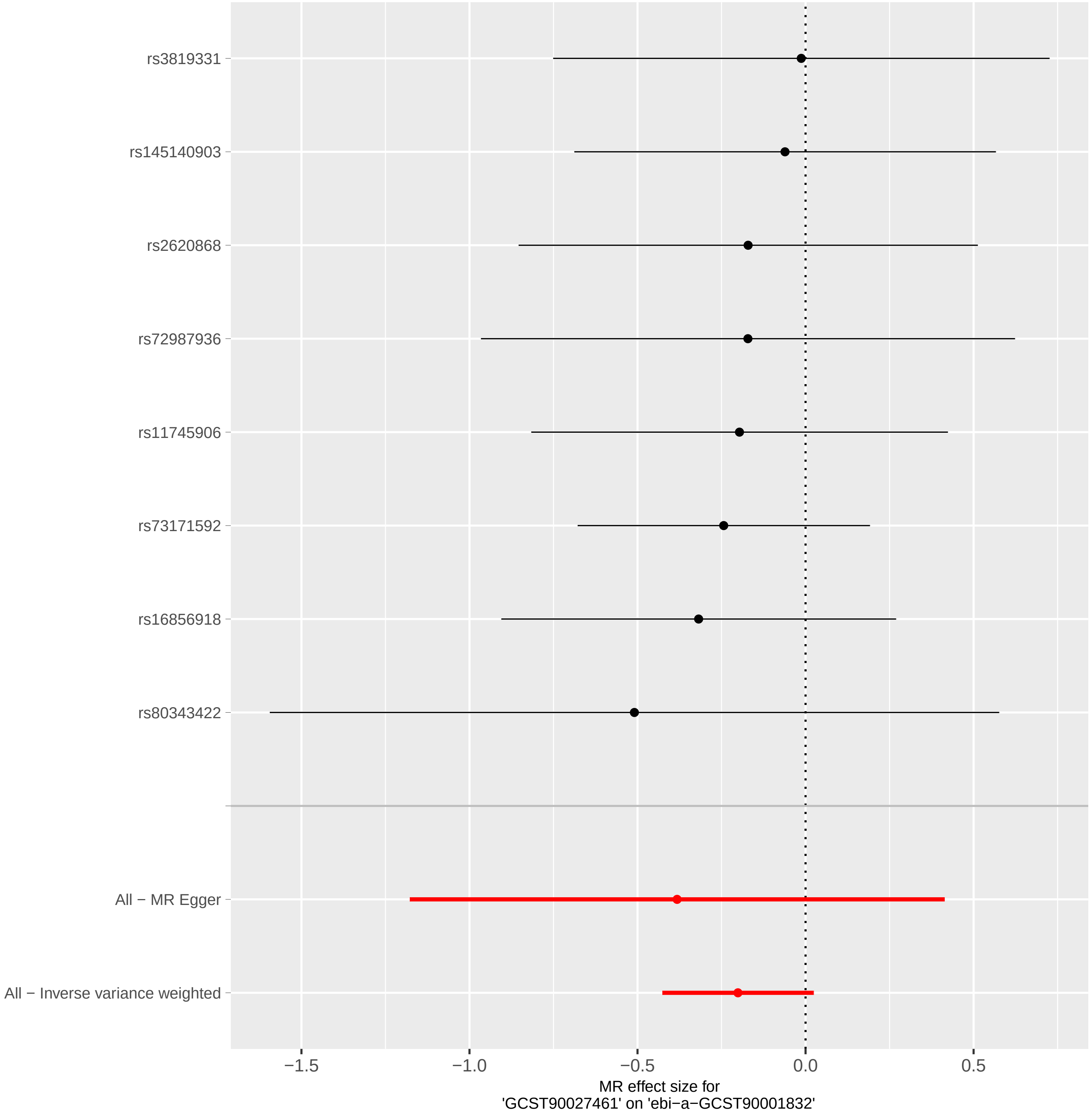
rs3819331
rs145140903
rs2620868
rs72987936
rs11745906
rs73171592
rs16856918
rs80343422
All − MR Egger
All − Inverse variance weighted
−1.5
−1.0
−0.5
0.0
0.5
MR effect size for
'GCST90027461' on 'ebi−a−GCST90001832'

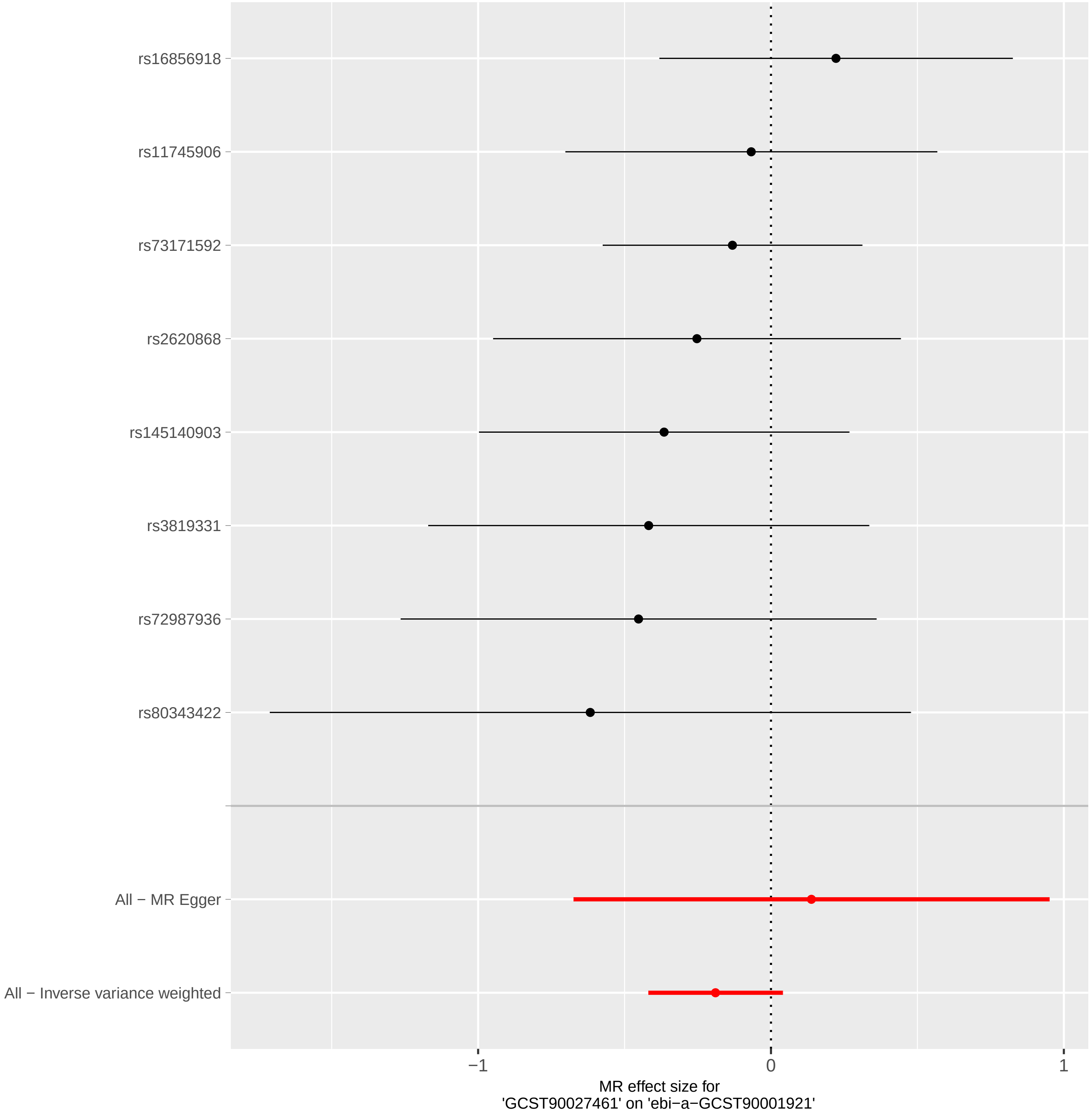
rs16856918
rs11745906
rs73171592
rs2620868
rs145140903
rs3819331
rs72987936
rs80343422
All − MR Egger
All − Inverse variance weighted
−1
0
1
MR effect size for
'GCST90027461' on 'ebi−a−GCST90001921'

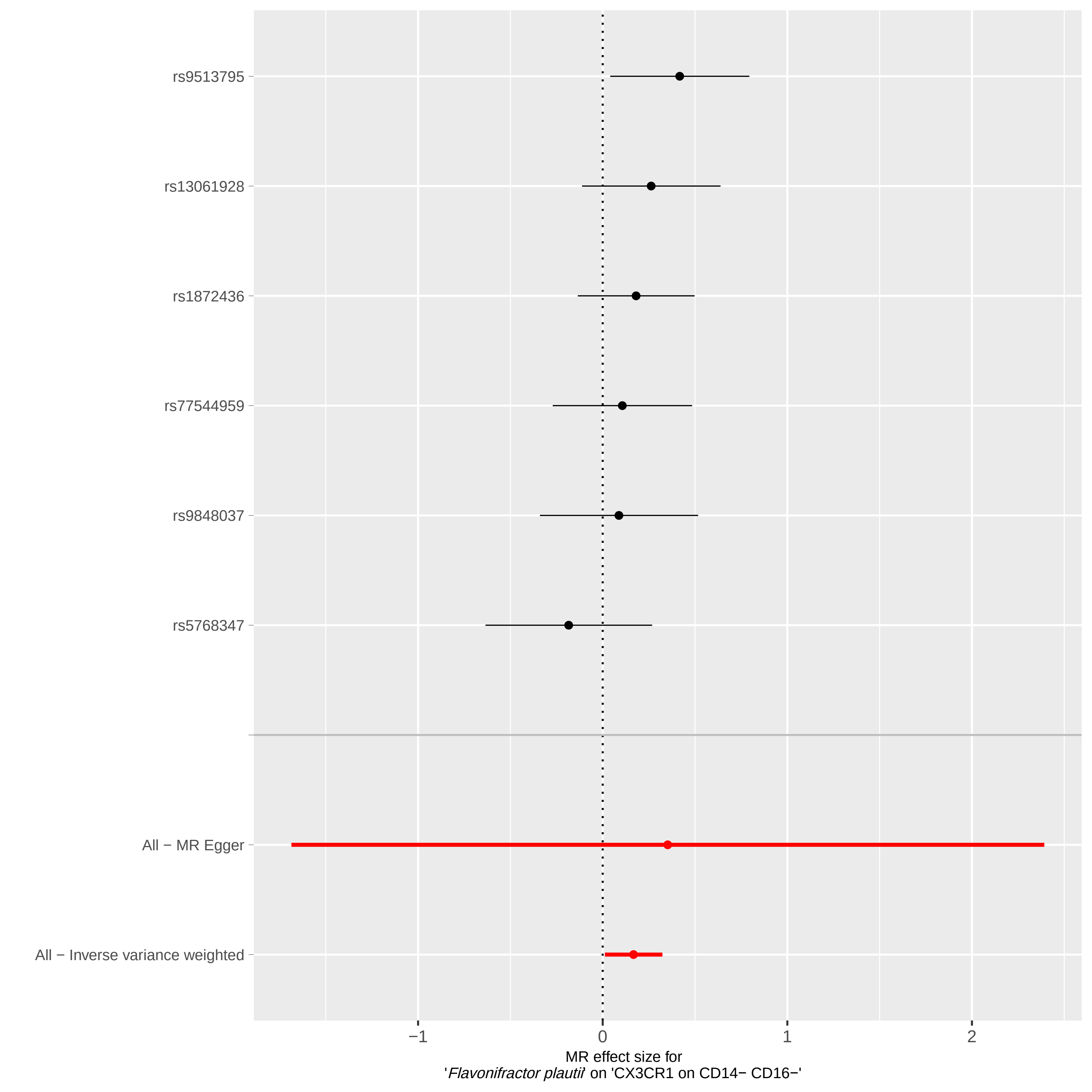

rs9513795
rs13061928
rs1872436
rs77544959
rs9848037
rs5768347
All − MR Egger
All − Inverse variance weighted
−1
0
1
2
MR effect size for
'*Flavonifractor plautii*' on 'CX3CR1 on CD14− CD16−'
