# Supplementary material for: Metabolites and Immune Cells Mediated the Causal Relationship Between the Gut Microbiota and Osteosarcoma: A Mendelian Randomization Study
Source: Health Sci Rep. 2025 Oct 30;8(11):e71430. doi: 10.1002/hsr2.71430 (PMC12575158; doi:10.1002/hsr2.71430)
Supplement: Supplementary file 2 — Supporting Figure 2: Scatter plots of SNP effect sizes for Mendelian randomization analyses visualizing genetic instrument associations between exposures and outcomes. [file HSR2-8-e71430-s003.pdf]

Figure Title

Supplementary Figure 2: Scatter Plots of SNP Effect Sizes for Mendelian Randomization Analyses Visualizing genetic instrument associations between exposures and outcomes

Figure Legend

1. Plot Description

Scatter plots displaying the relationship between SNP effect sizes on exposures (x-axis) and outcomes (y-axis) for Mendelian Randomization (MR) analyses. Each point represents an individual SNP's effect estimates.

2. Key Components

(1) Regression lines:

Colored lines show causal effect estimates from different MR methods:

Inverse variance weighted (blue): Primary fixed-effects estimator

MR Egger (blue): Corrects for directional pleiotropy

Weighted median (green): Robust to invalid instruments (<50% invalid)

Simple mode/Weighted mode (green/red): Additional pleiotropy-robust methods

(2) Axes:

X-axis: Standardized SNP effect on exposure (e.g., GCST90027452, Flavonifractor, PWY-3001)

Y-axis: Standardized SNP effect on outcome (primarily Osteosarcoma, with metabolic pathways and microbial taxa)

(3) Labels:

Top title format: "MR effect size for '[EXPOSURE]' on '[OUTCOME]'"

3. Exposure Categories

Genetic studies: GCST... (GWAS Catalog IDs)

Microbial taxa: Hierarchical names (e.g., k__Bacteria...s__Flavonifractor_plautii)

Metabolic pathways: Abbreviated names (e.g., AST.PWY..L.arginine.degradation)

Metabolites: Alphanumeric IDs (e.g., cmpf, e12NEt, 6ckFav)

4. Interpretation Guide

Slope direction:

Positive slope (/) → Exposure increases outcome risk

Negative slope (\) → Exposure decreases outcome risk

Outlier SNPs: Points deviating from regression lines may indicate pleiotropy

Method concordance: Consistent slopes across methods strengthen causal inference

5. Scale Note

Axis ranges vary across subplots (e.g., x: 0.1–0.3 vs. 0–2.5; y: -0.05–0.05 vs. -5–5) to optimally display data distributions.

Note: MR analyses test causal hypotheses using genetic variants as instrumental variables. SNP effect sizes derived from genome-wide association studies (GWAS).

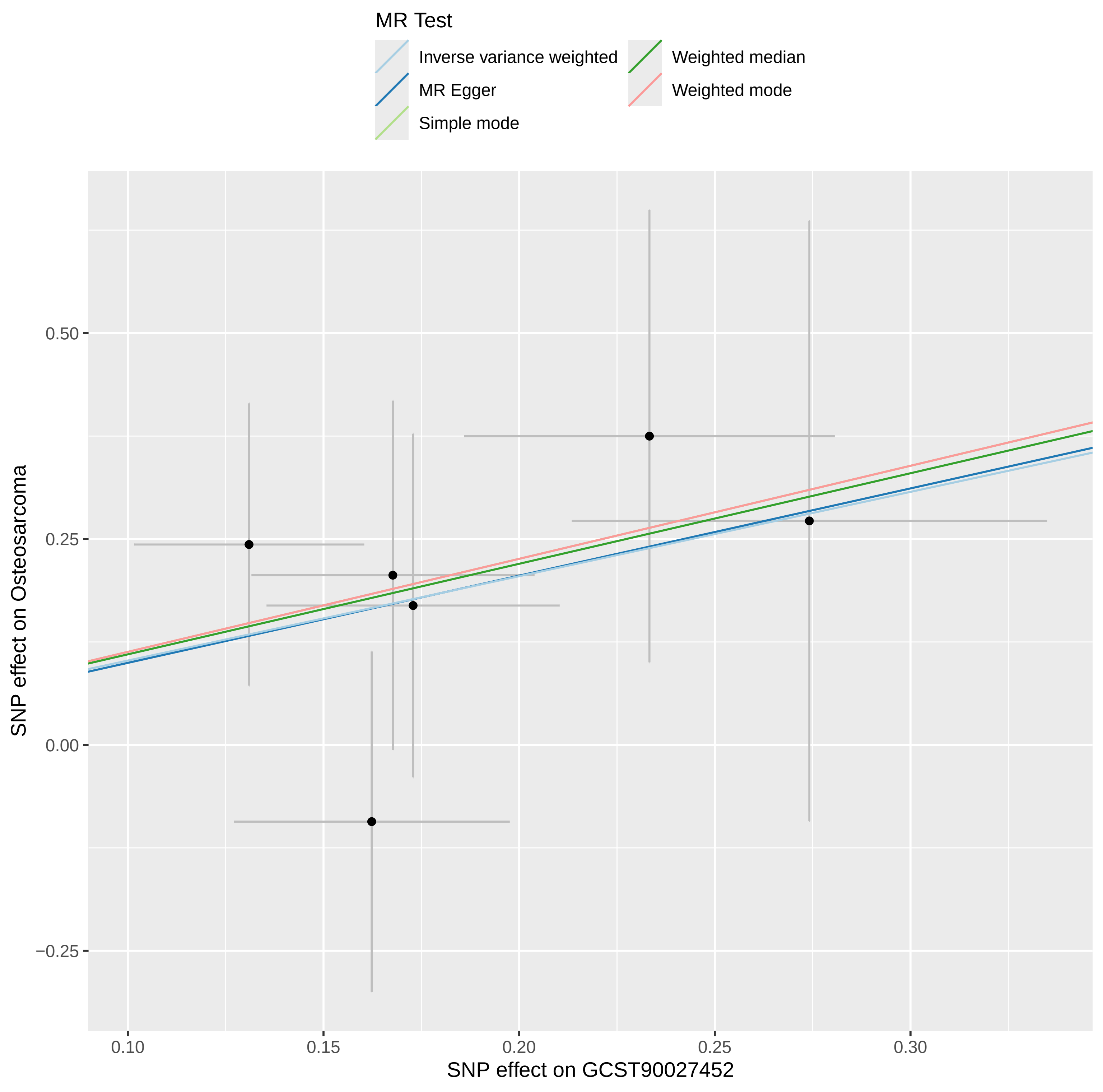
MR Test
Inverse variance weighted
MR Egger
Simple mode
Weighted median
Weighted mode
0.50
0.25
0.00
−0.25
0.10
0.15
0.20
0.25
0.30
SNP effect on Osteosarcoma
SNP effect on GCST90027452

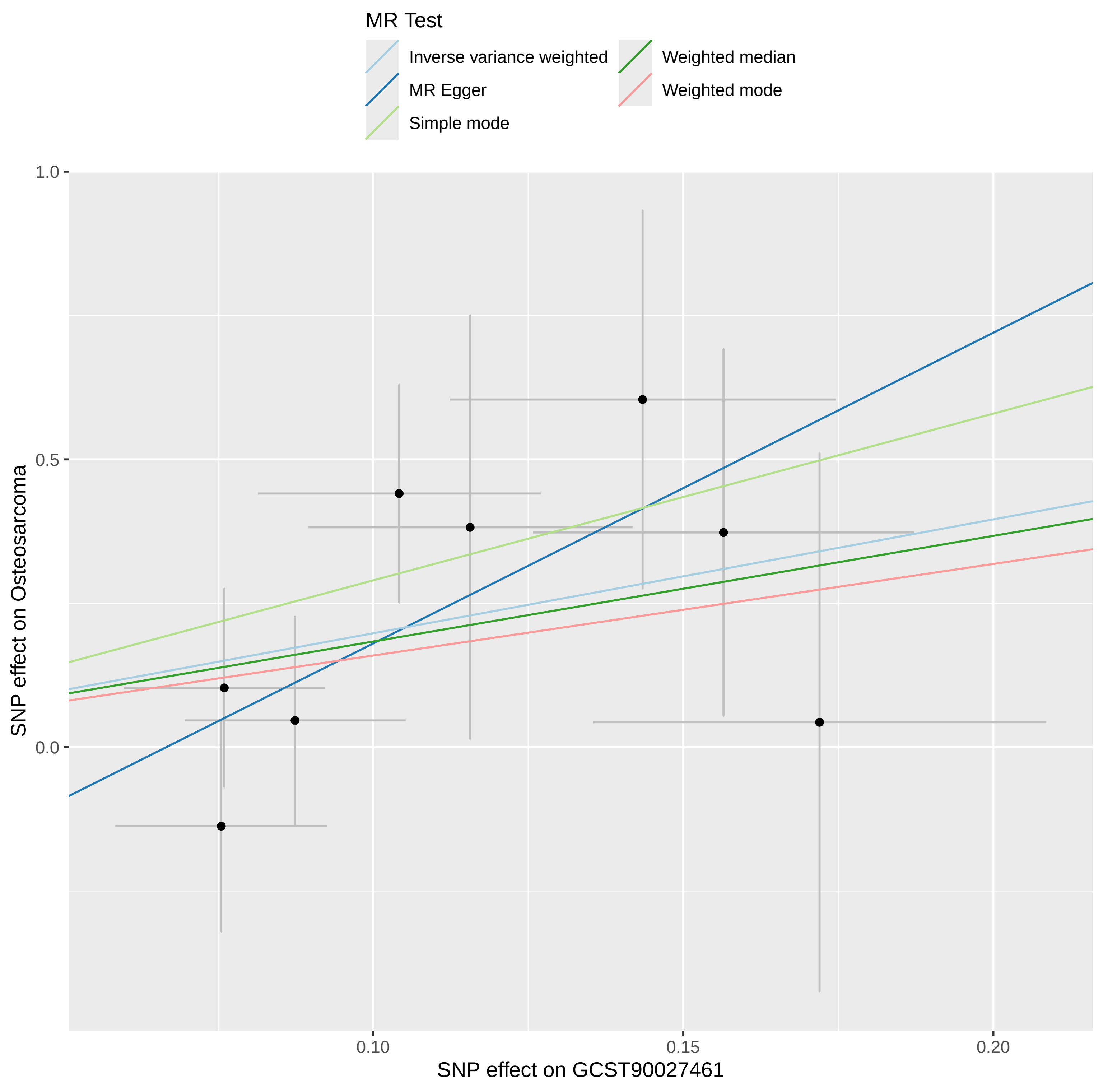
MR Test
Inverse variance weighted
MR Egger
Simple mode
Weighted median
Weighted mode
1.0
0.5
0.0
SNP effect on Osteosarcoma
0.10
0.15
0.20
SNP effect on GCST90027461

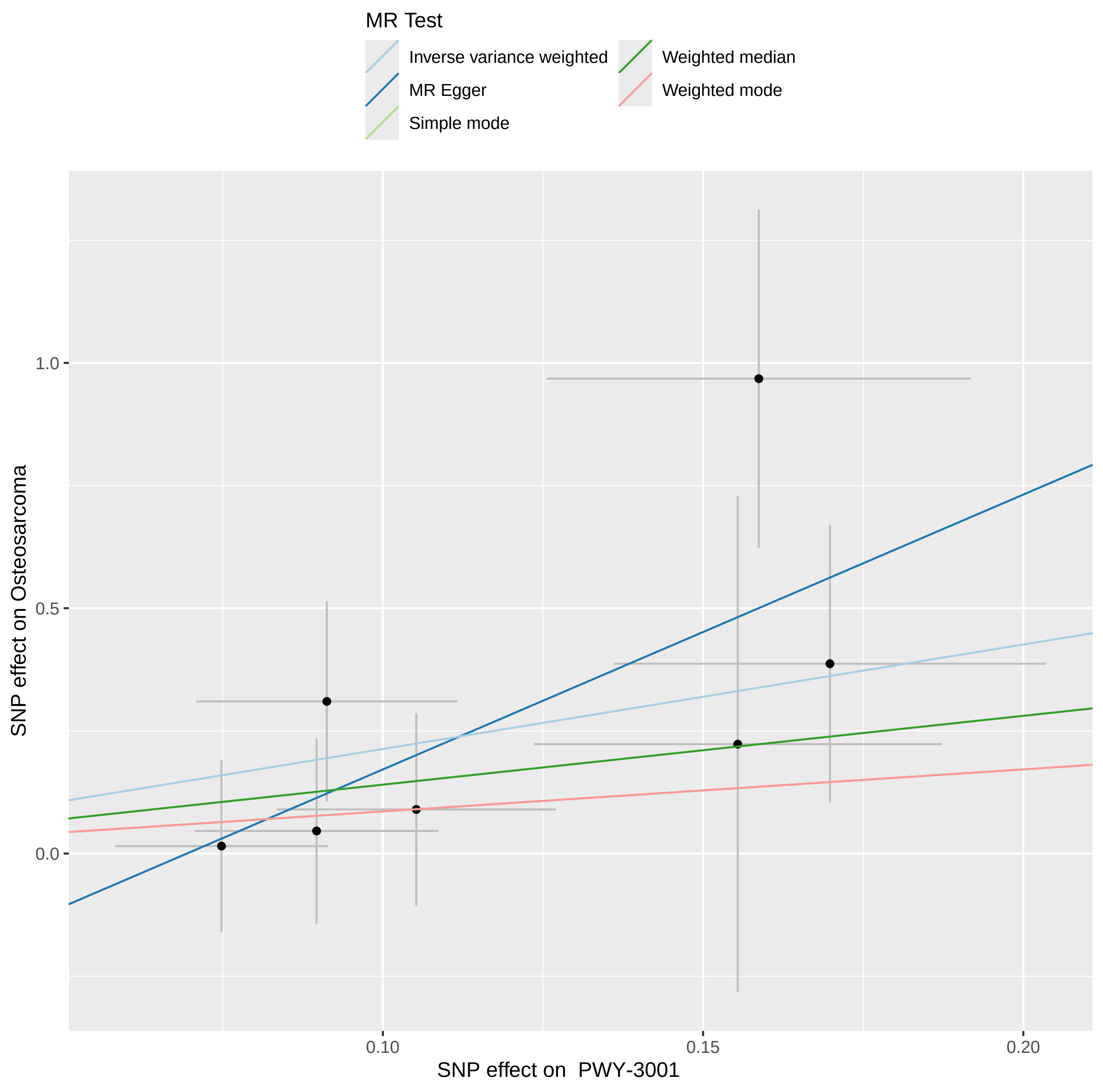

MR Test
Inverse variance weighted
MR Egger
Simple mode
Weighted median
Weighted mode
SNP effect on Osteosarcoma
1.0
0.5
0.0
0.10
0.15
0.20
SNP effect on PWY-3001

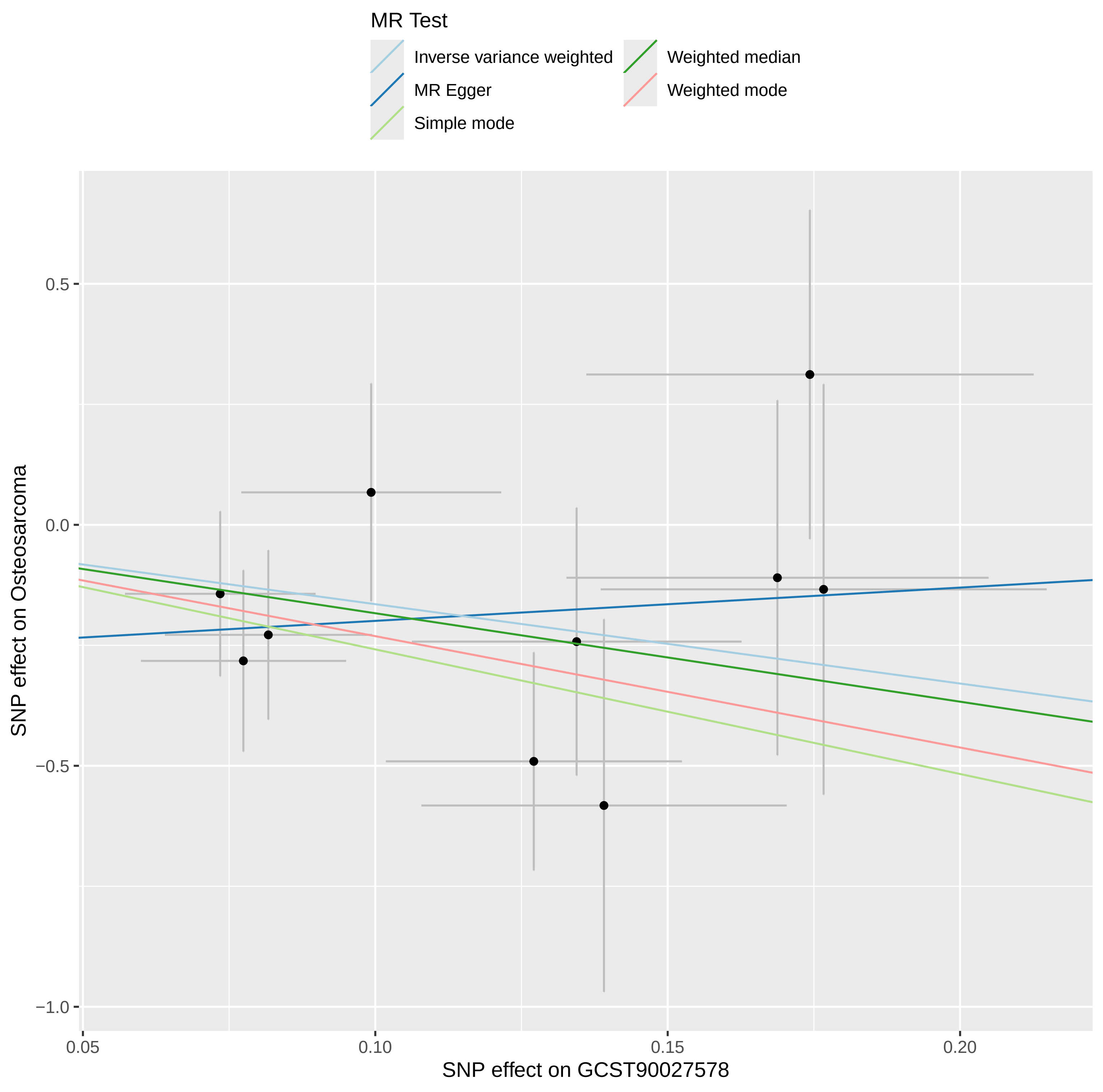
MR Test
Inverse variance weighted
MR Egger
Simple mode
Weighted median
Weighted mode
SNP effect on Osteosarcoma
0.5
0.0
−0.5
−1.0
0.05
0.10
0.15
0.20
SNP effect on GCST90027578

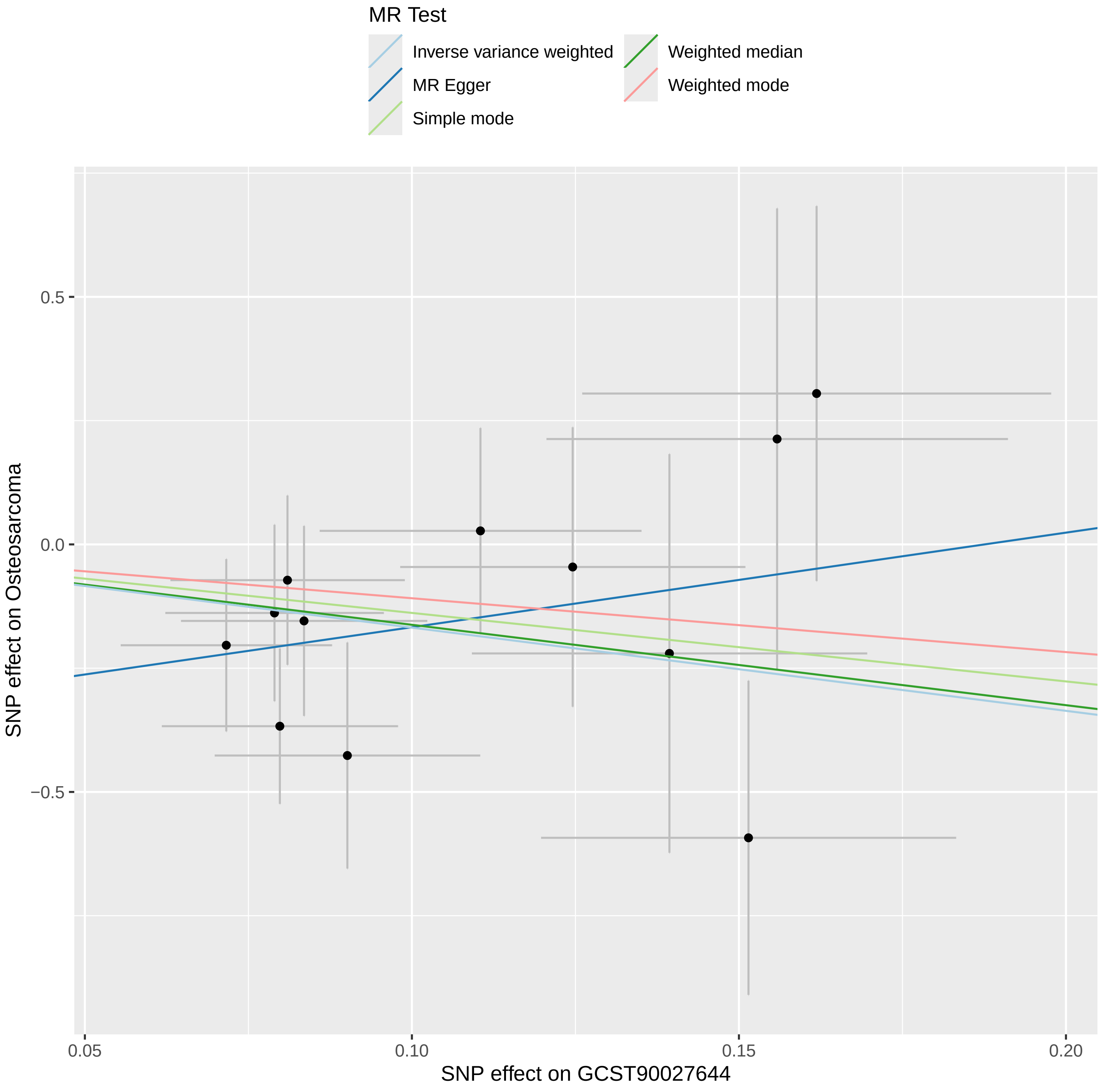
MR Test
Inverse variance weighted
MR Egger
Simple mode
Weighted median
Weighted mode
0.5
0.0
−0.5
SNP effect on Osteosarcoma
0.05
0.10
0.15
0.20
SNP effect on GCST90027644

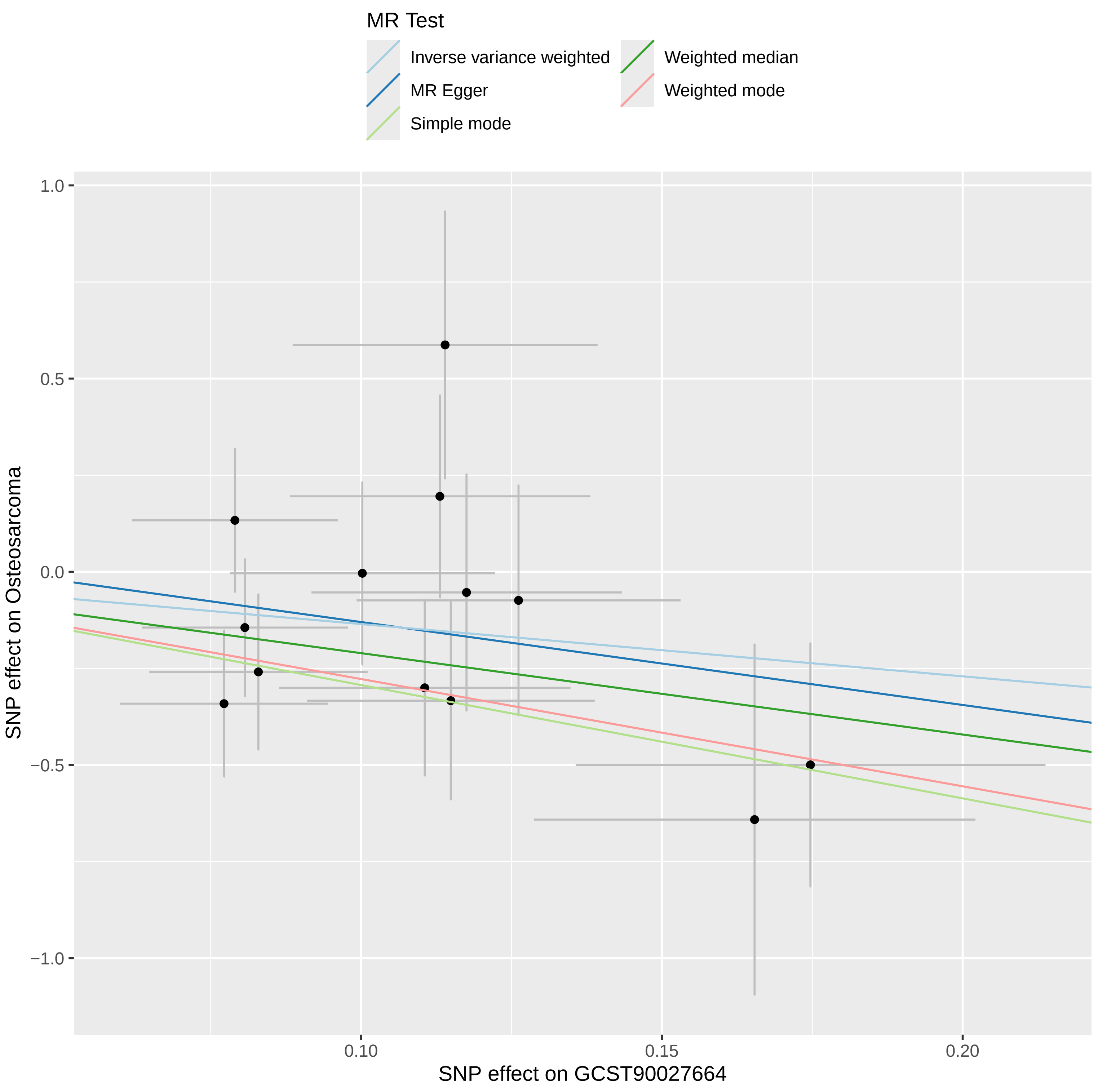
MR Test
Inverse variance weighted
MR Egger
Simple mode
Weighted median
Weighted mode
1.0
0.5
0.0
−0.5
−1.0
SNP effect on Osteosarcoma
0.10
0.15
0.20
SNP effect on GCST90027664

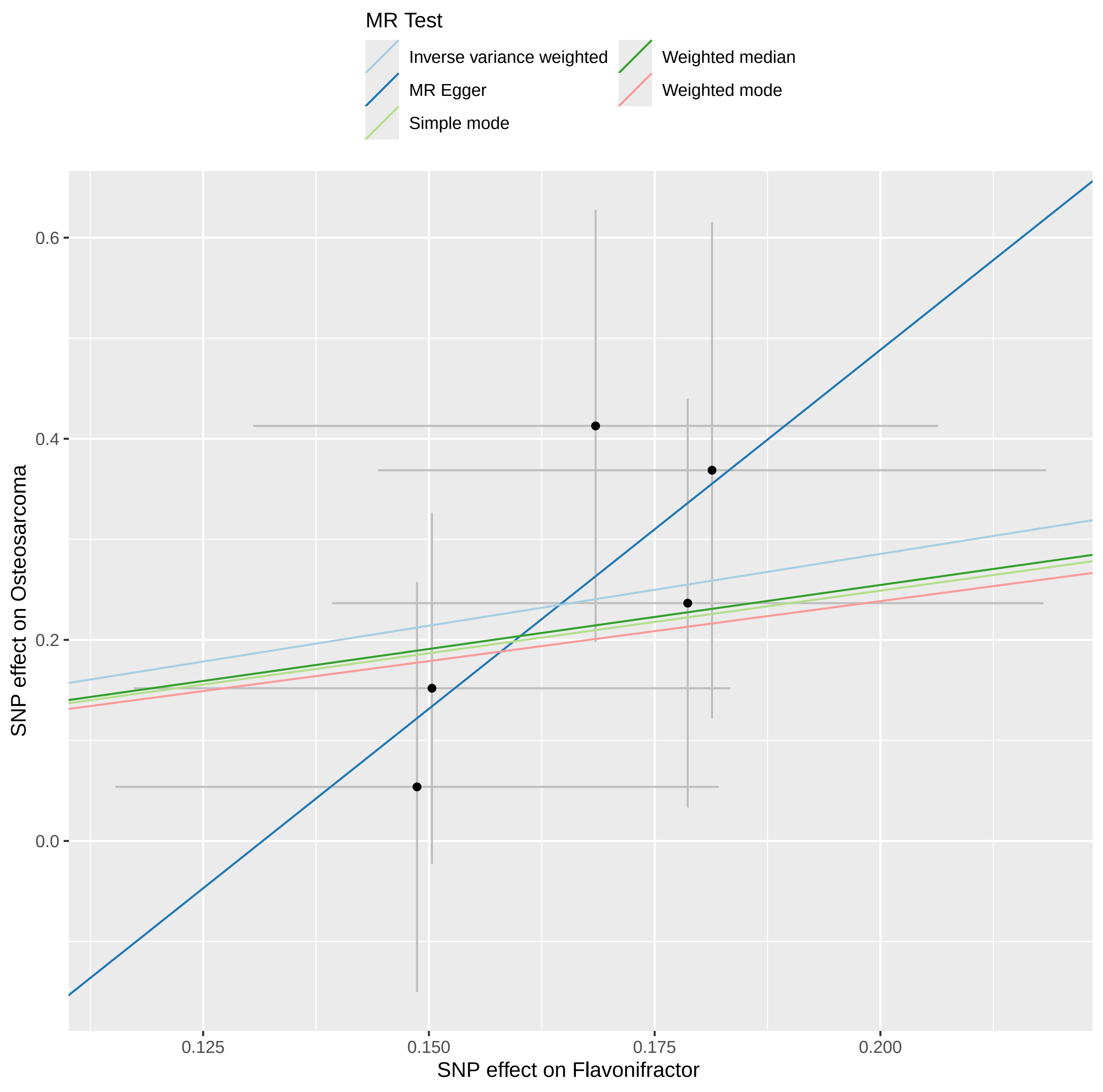

MR Test
Inverse variance weighted
MR Egger
Simple mode
Weighted median
Weighted mode
0.6
0.4
0.2
0.0
0.125
0.150
0.175
0.200
SNP effect on Osteosarcoma
SNP effect on Flavonifractor

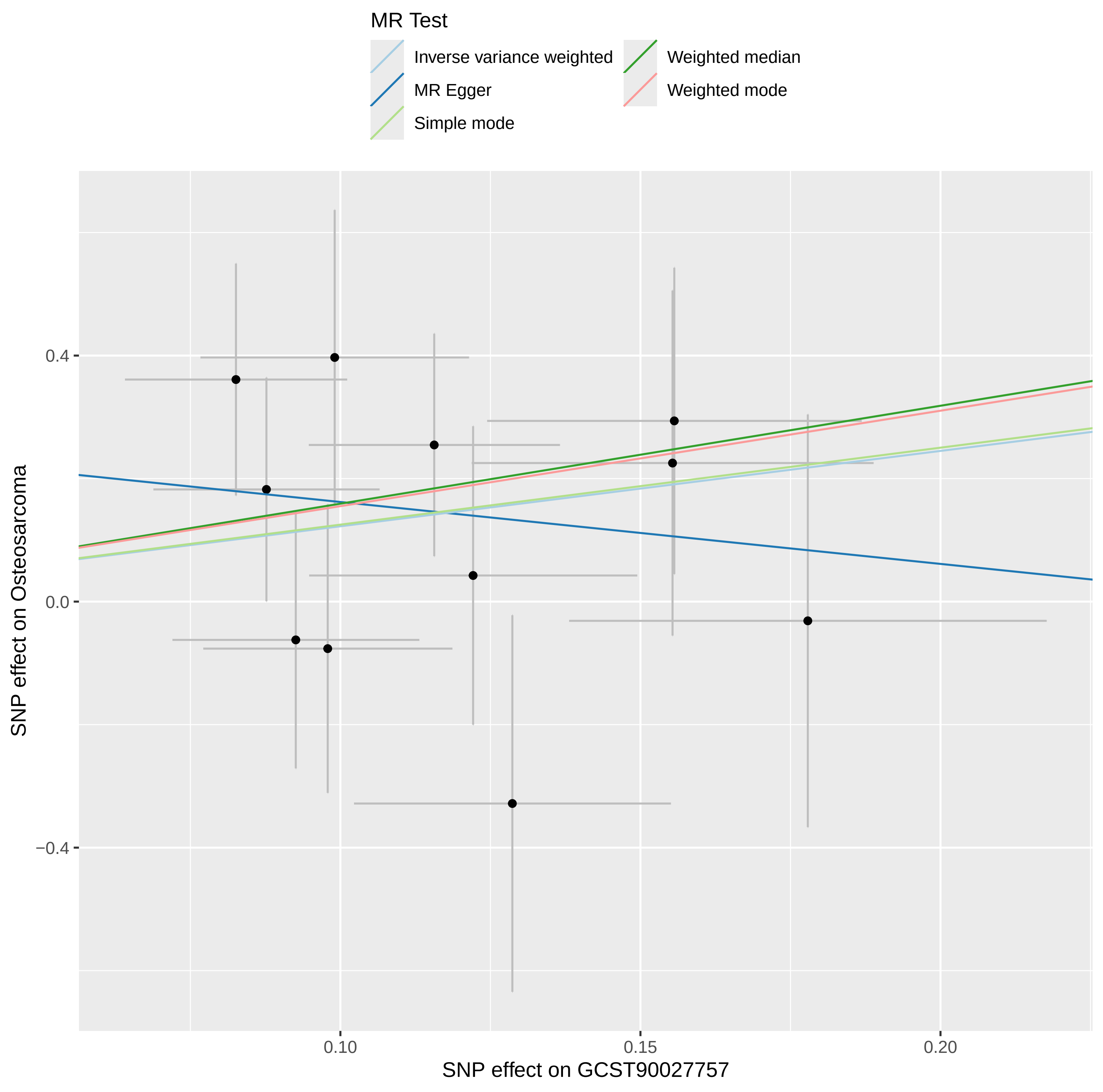

MR Test
Inverse variance weighted
MR Egger
Simple mode
Weighted median
Weighted mode
0.4
0.0
−0.4
0.10
0.15
0.20
SNP effect on Osteosarcoma
SNP effect on GCST90027757

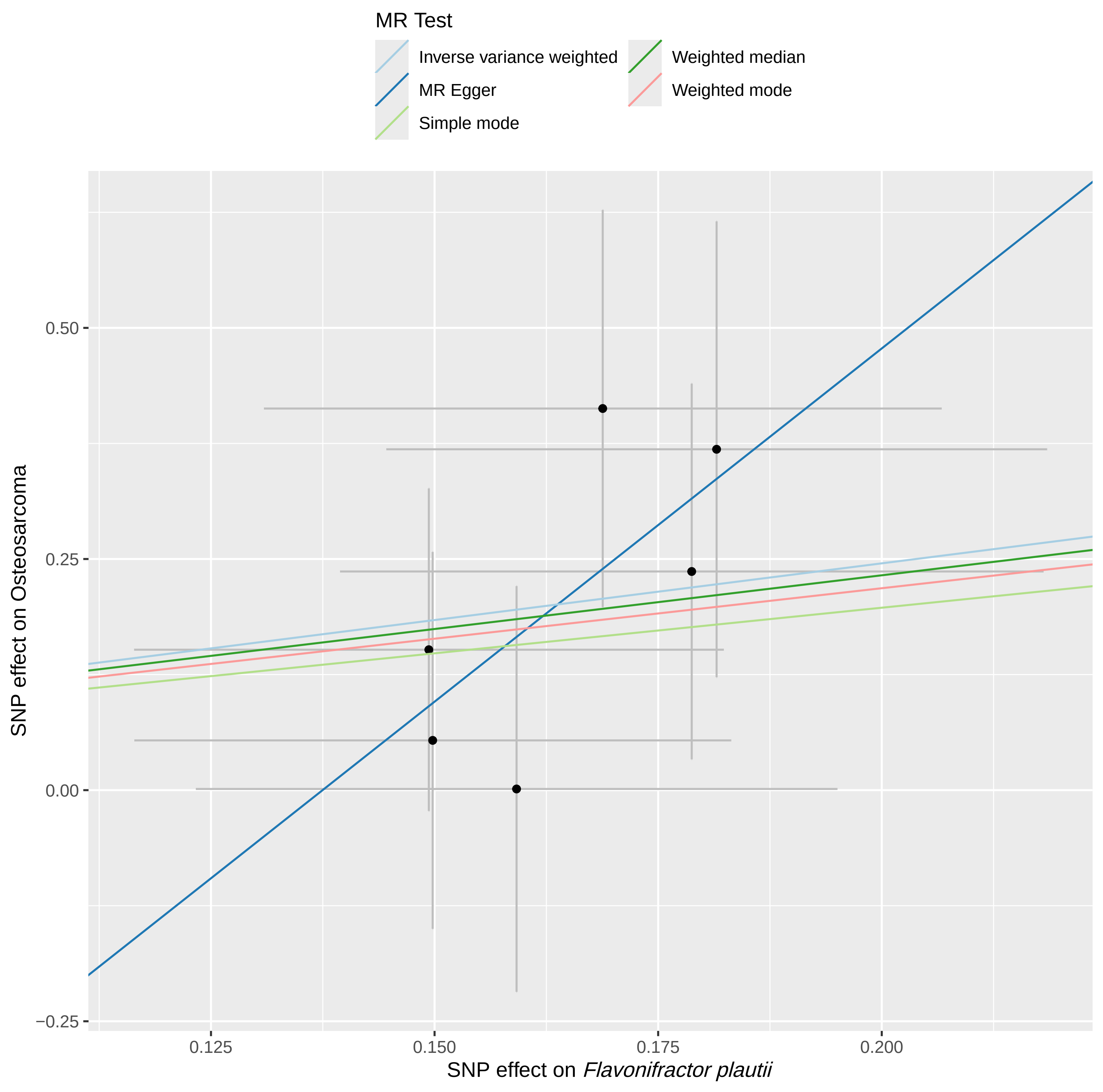

MR Test
Inverse variance weighted
MR Egger
Simple mode
Weighted median
Weighted mode
0.50
0.25
0.00
−0.25
0.125
0.150
0.175
0.200
SNP effect on Osteosarcoma
SNP effect on Flavonifractor plautii

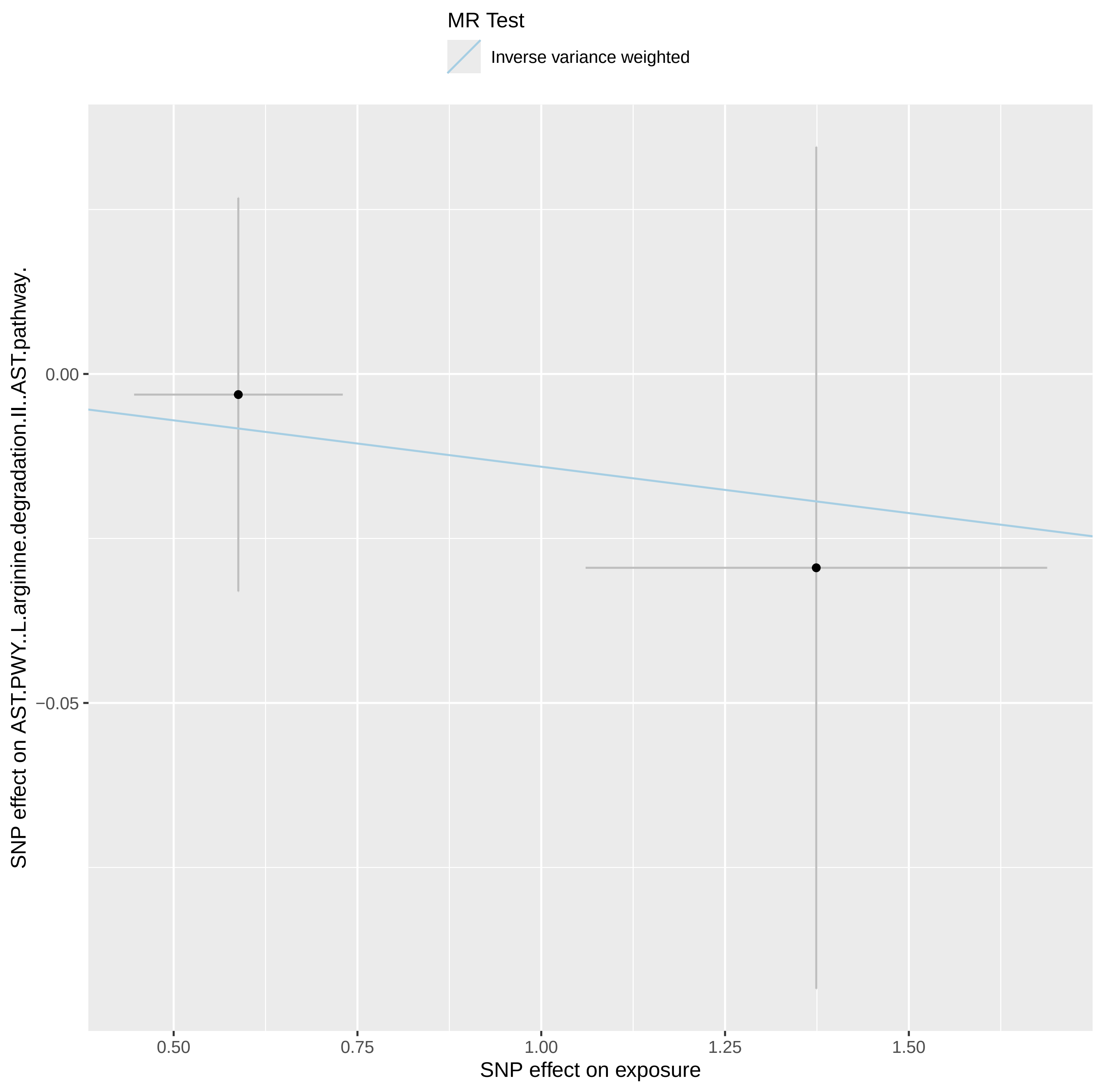

MR Test
Inverse variance weighted
SNP effect on AST.PWY..L.arginine.degradation.II..AST.pathway.
0.00
−0.05
0.50
0.75
1.00
1.25
1.50
SNP effect on exposure

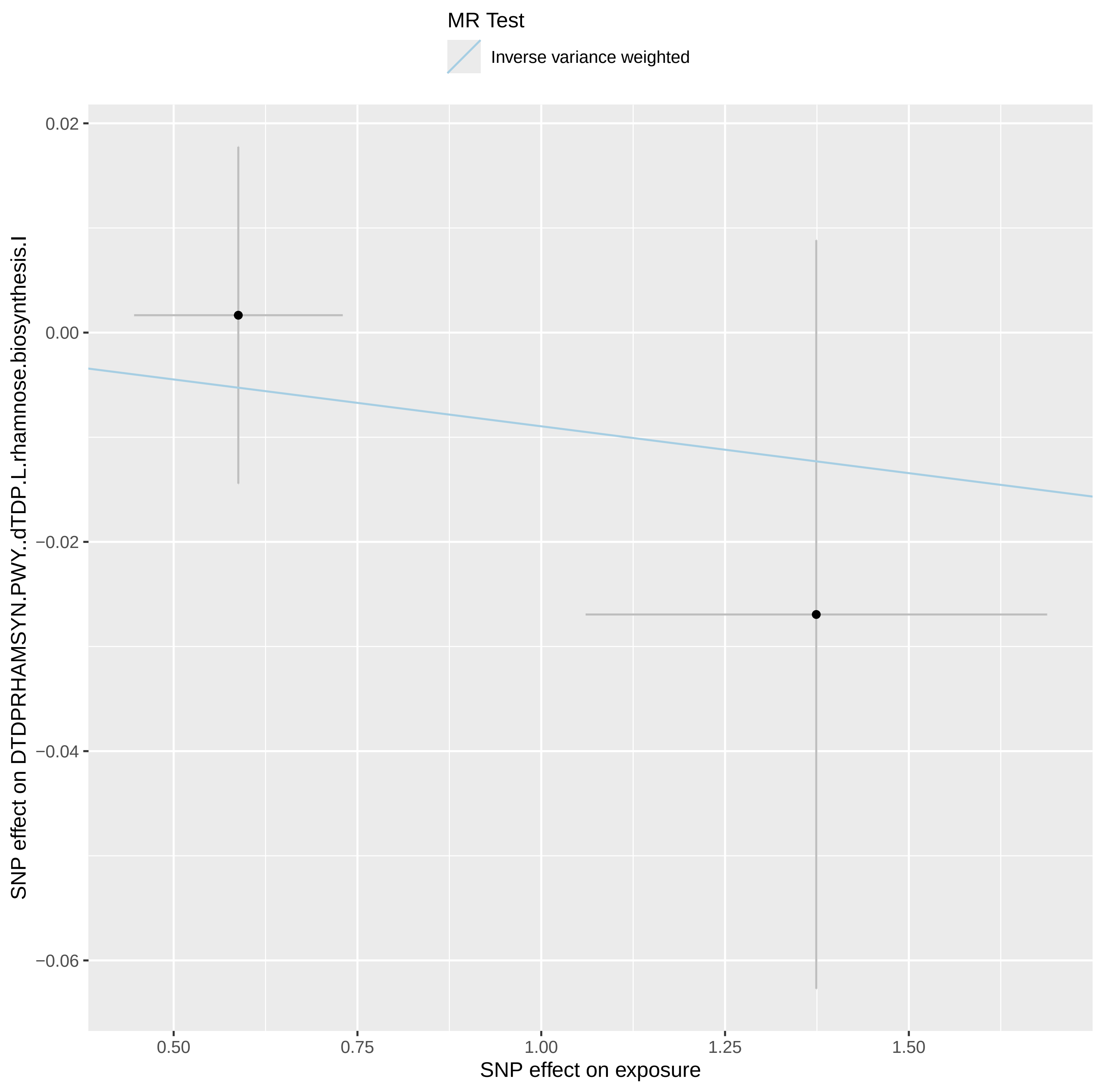

MR Test
Inverse variance weighted
SNP effect on DTDPRHAMSYN.PWY..dTDP.L.rhamnose.biosynthesis.I
0.02
0.00
−0.02
−0.04
−0.06
0.50
0.75
1.00
1.25
1.50
SNP effect on exposure

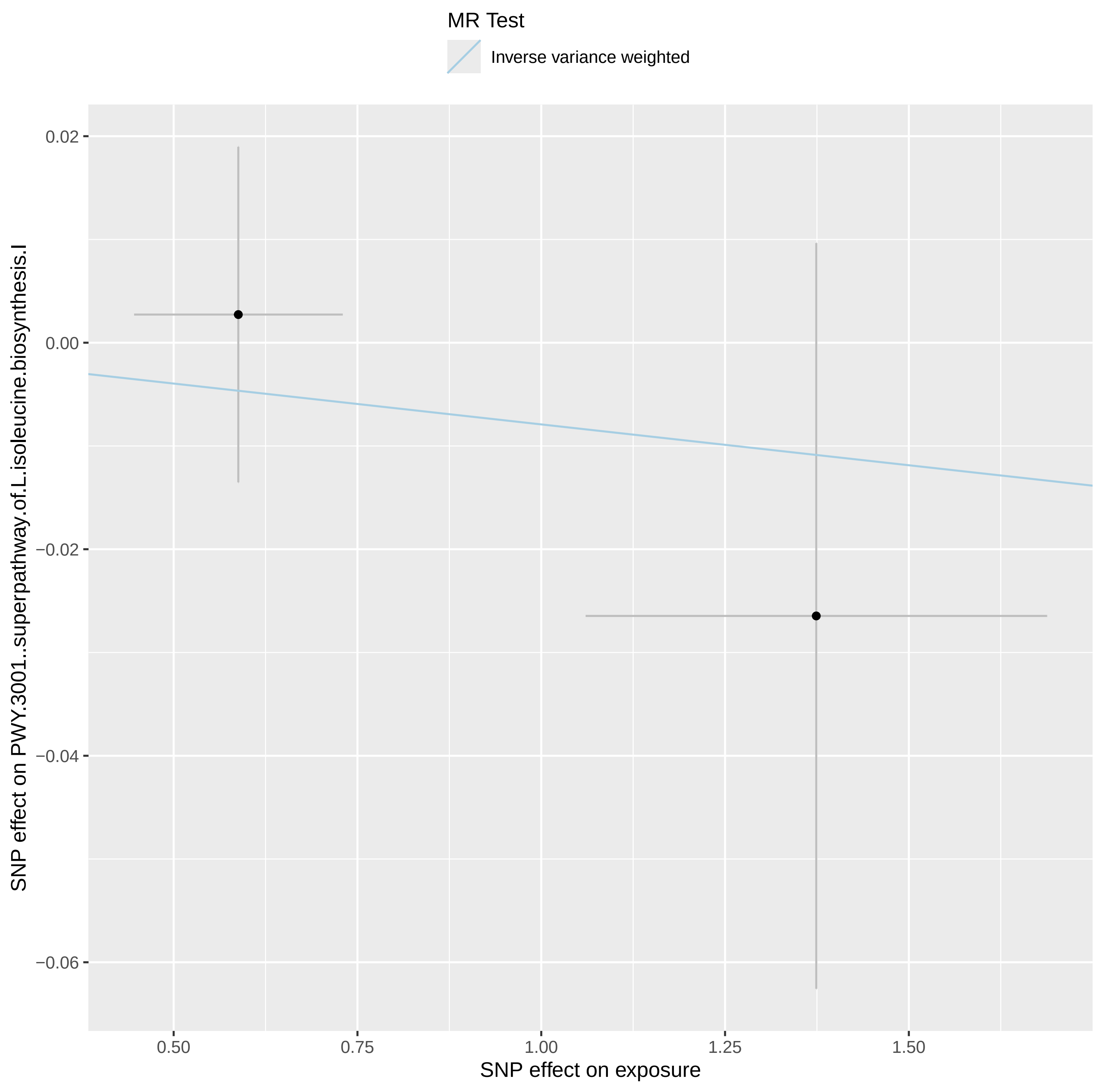
MR Test
Inverse variance weighted
0.02
0.00
−0.02
−0.04
−0.06
0.50
0.75
1.00
1.25
1.50
SNP effect on exposure
SNP effect on PWY.3001..superpathway.of.L.isoleucine.biosynthesis.I

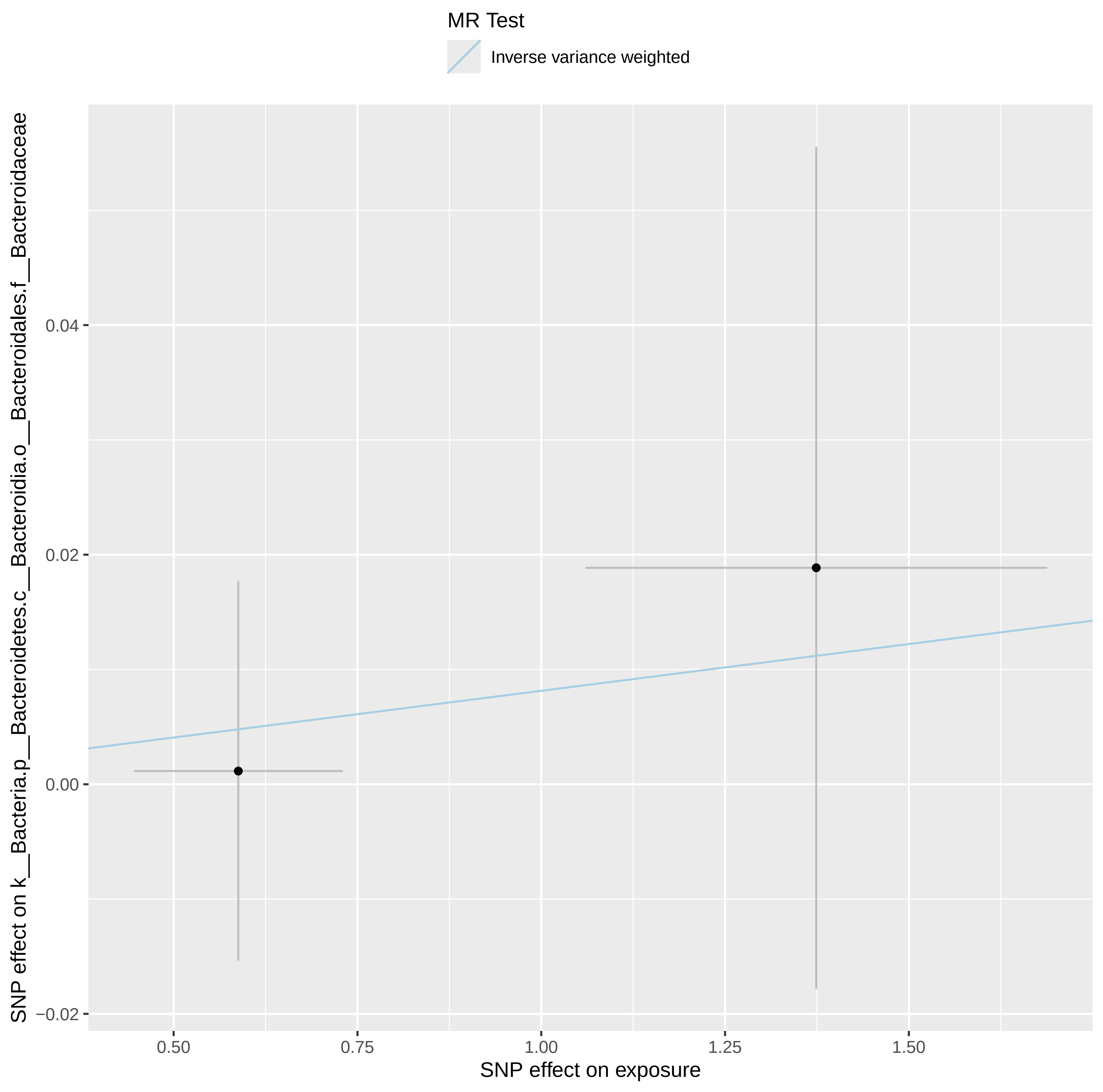
MR Test
Inverse variance weighted
SNP effect on k__Bacteria.p__Bacteroidetes.c__Bacteroidia.o__Bacteroidales.f__Bacteroidaceae
0.04
0.02
0.00
−0.02
0.50
0.75
1.00
1.25
1.50
SNP effect on exposure

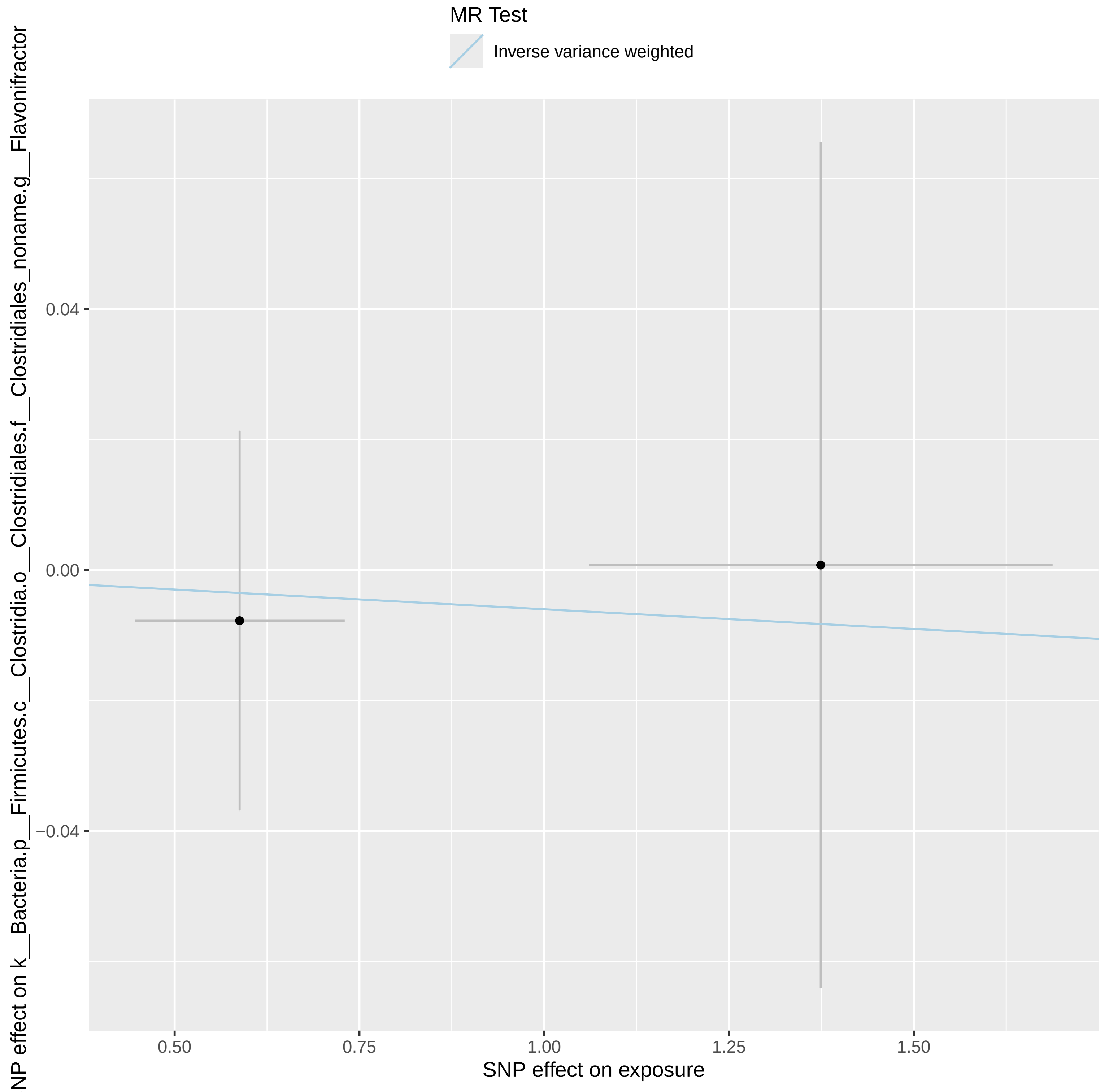
MR Test
Inverse variance weighted
SNP effect on k__Bacteria.p__Firmicutes.c__Clostridia.o__Clostridiales.f__Clostridiales_noname.g__Flavonifractor
0.04
0.00
−0.04
0.50
0.75
1.00
1.25
1.50
SNP effect on exposure

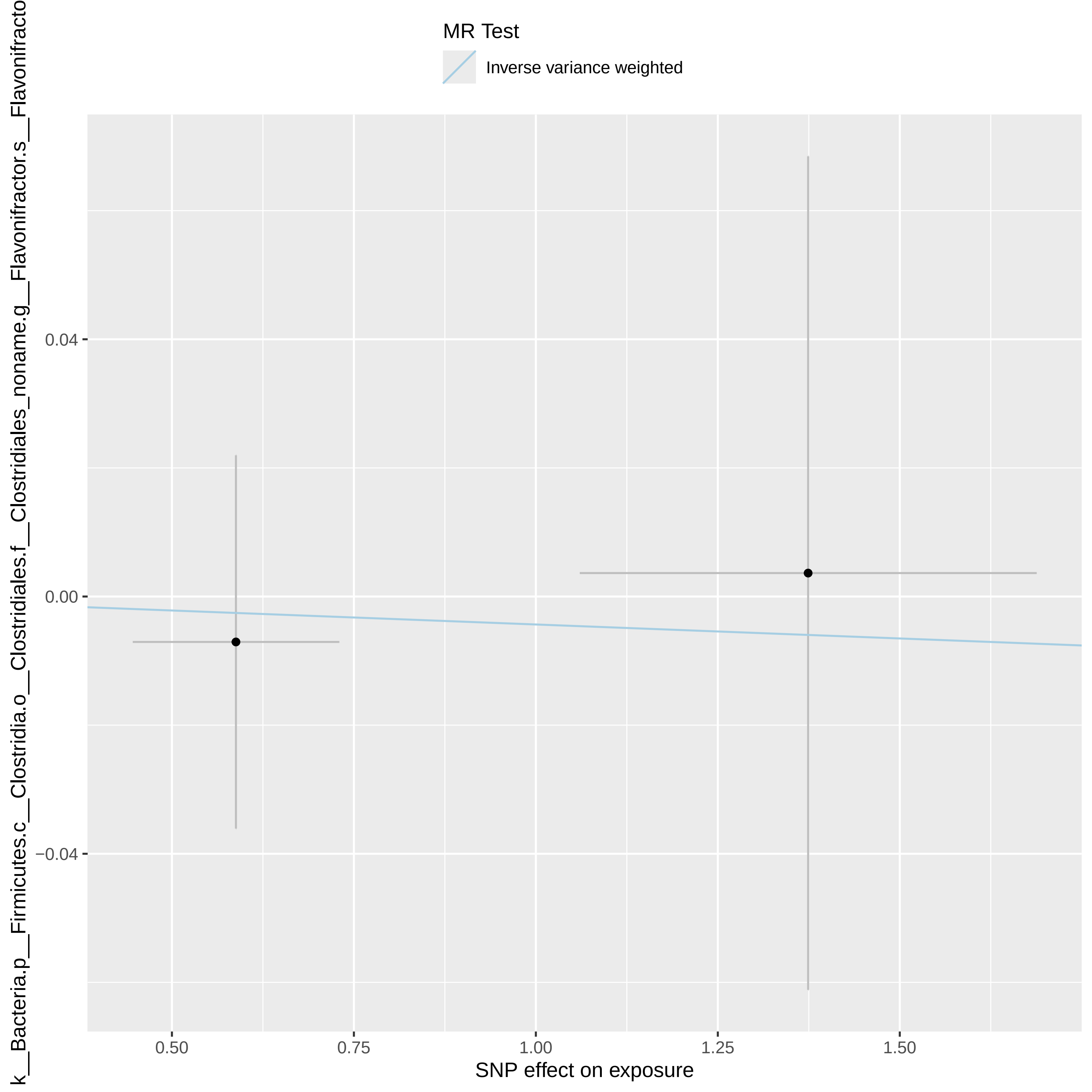
MR Test
Inverse variance weighted
0.04
0.00
−0.04
0.50
0.75
1.00
1.25
1.50
SNP effect on exposure
k__Bacteria.p__Firmicutes.c__Clostridia.o__Clostridiales.f__Clostridiales_noname.g__Flavonifractor.s__Flavonifracto

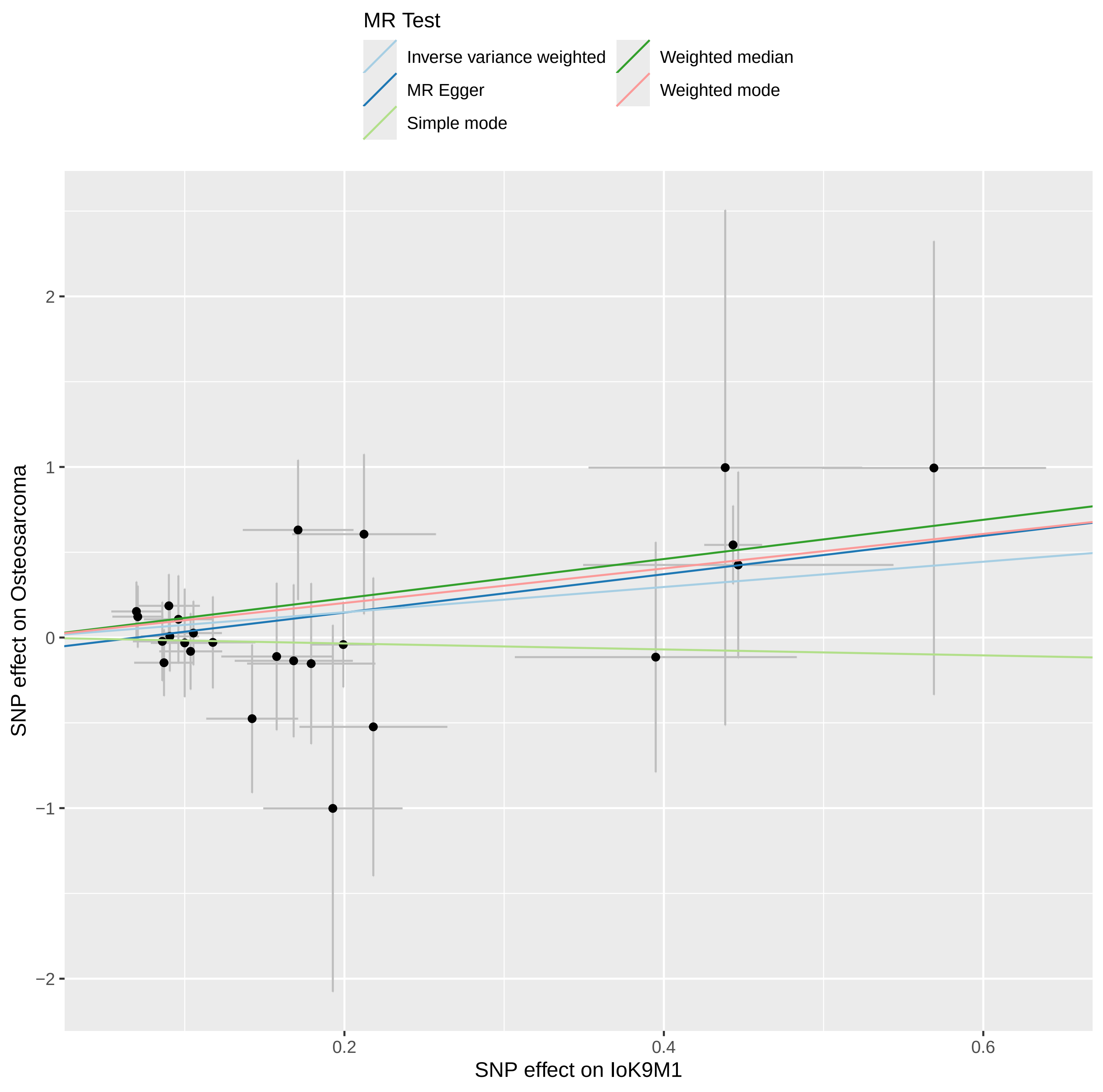

MR Test
Inverse variance weighted
MR Egger
Simple mode
Weighted median
Weighted mode
2
1
0
−1
−2
SNP effect on Osteosarcoma
0.2
0.4
0.6
SNP effect on IoK9M1

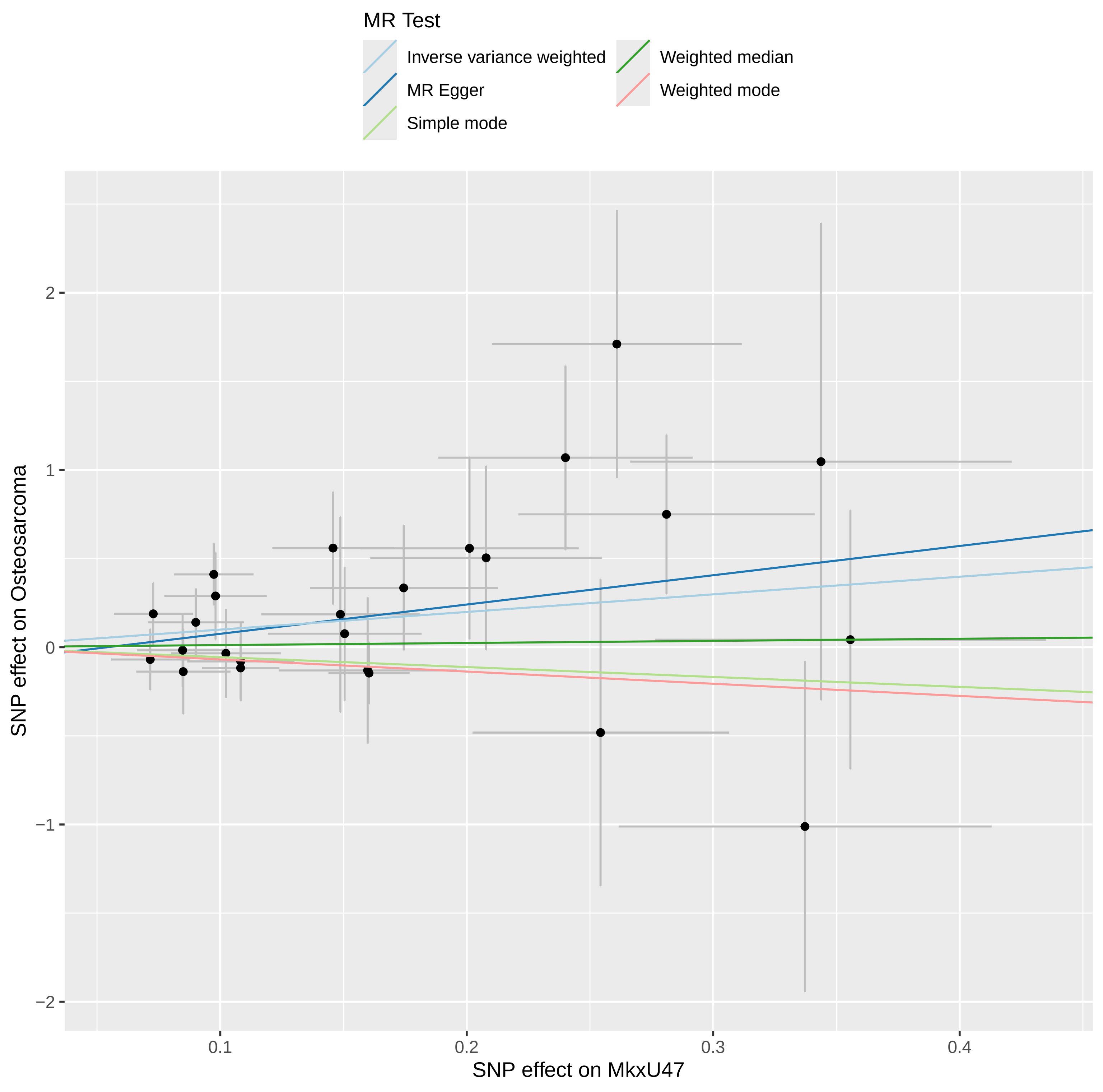

MR Test
Inverse variance weighted
MR Egger
Simple mode
Weighted median
Weighted mode
SNP effect on Osteosarcoma
2
1
0
−1
−2
0.1
0.2
0.3
0.4
SNP effect on MkxU47

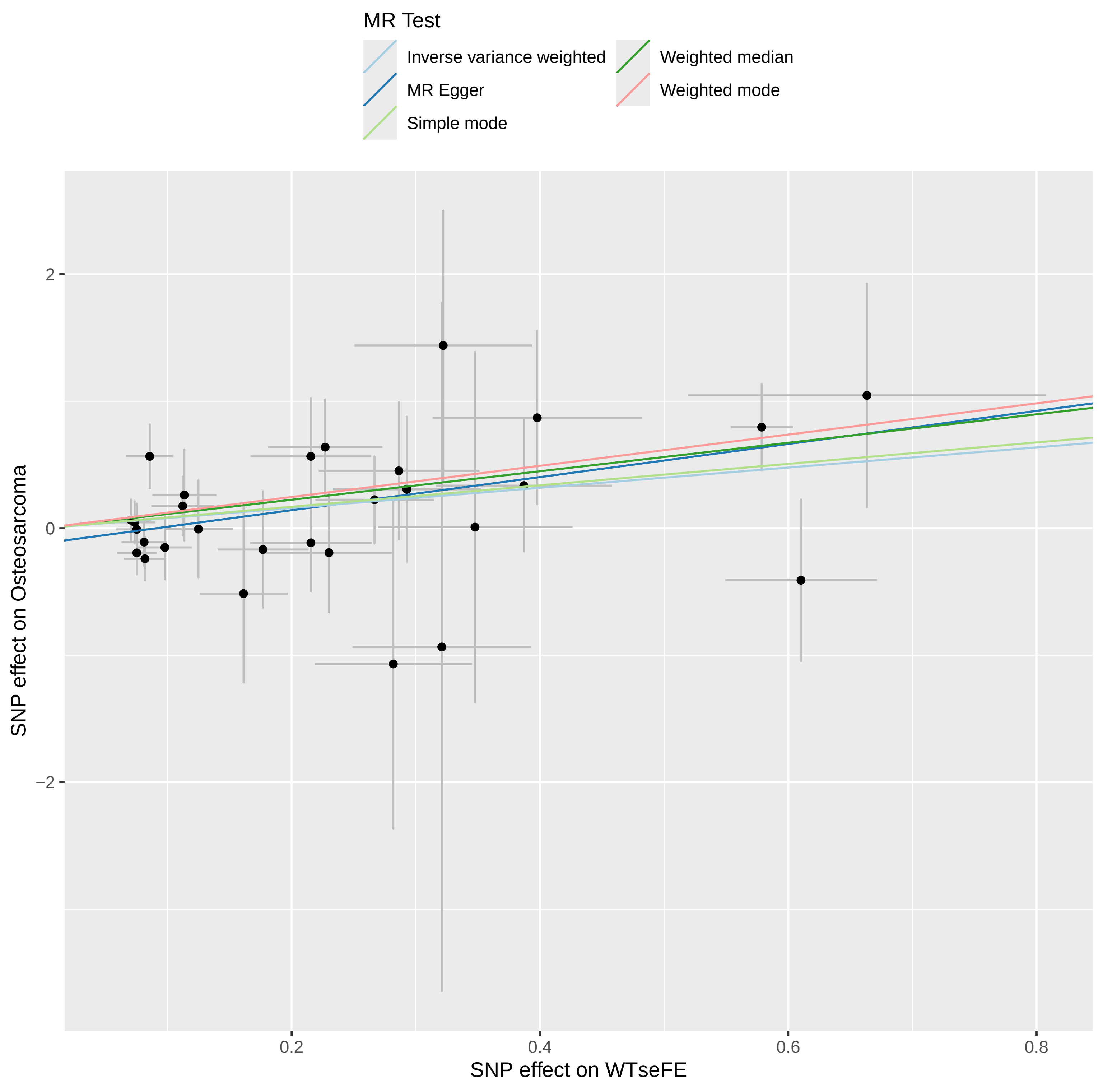
MR Test
Inverse variance weighted
MR Egger
Simple mode
Weighted median
Weighted mode
2
0
−2
0.2
0.4
0.6
0.8
SNP effect on Osteosarcoma
SNP effect on WTseFE

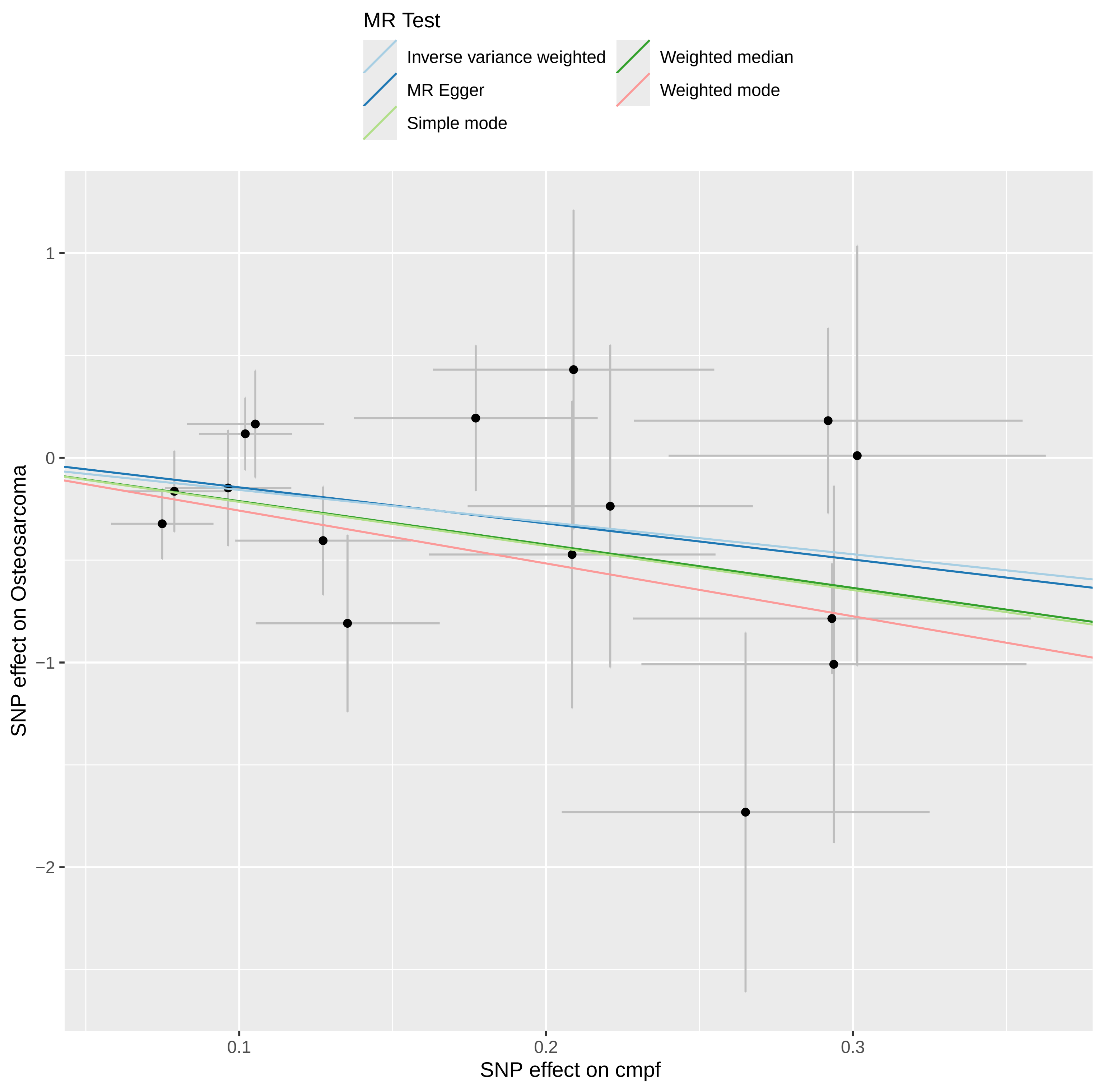
MR Test
Inverse variance weighted
MR Egger
Simple mode
Weighted median
Weighted mode
1
0
−1
−2
SNP effect on Osteosarcoma
0.1
0.2
0.3
SNP effect on cmpf

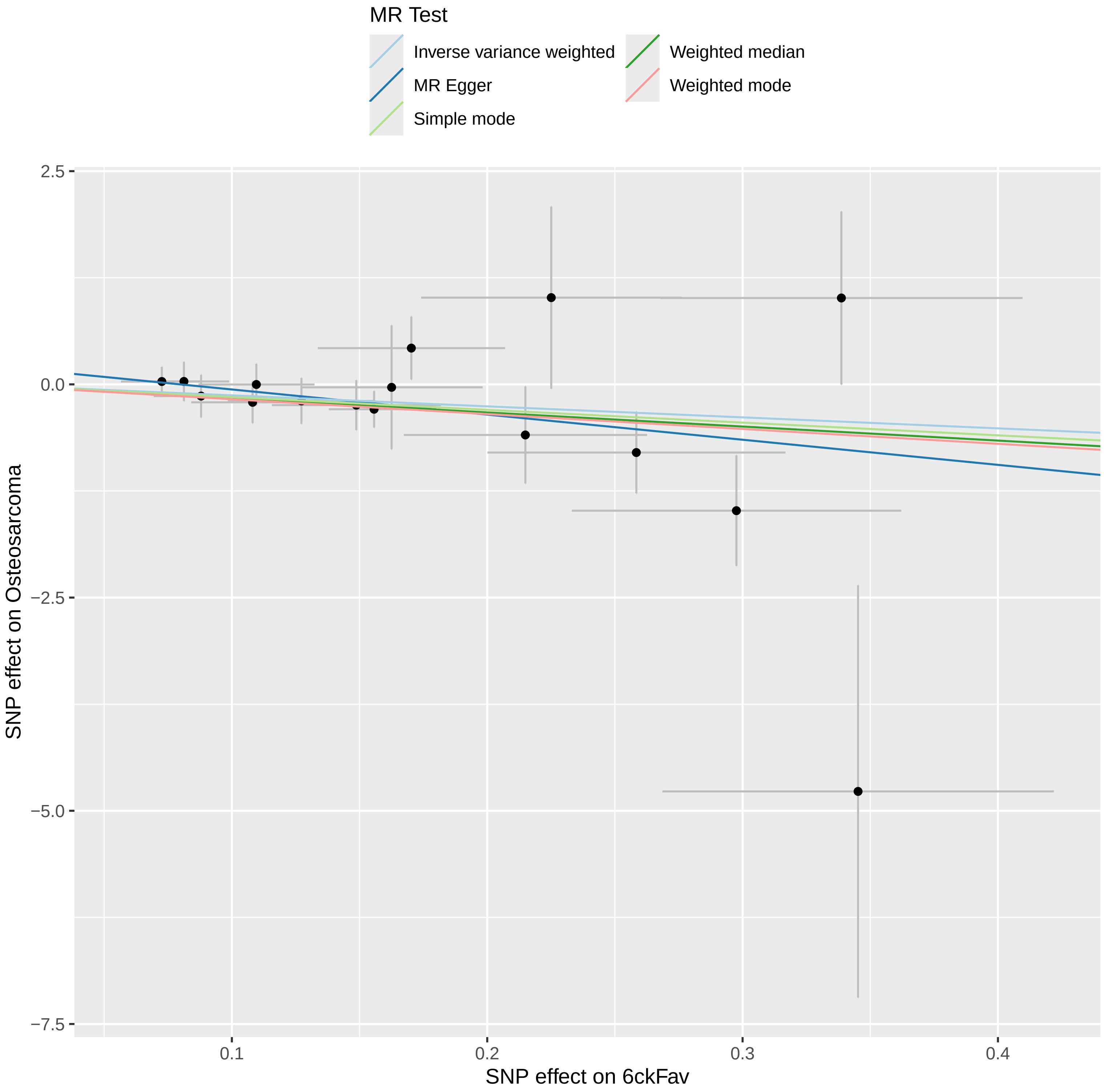
MR Test
Inverse variance weighted
MR Egger
Simple mode
Weighted median
Weighted mode
2.5
0.0
−2.5
−5.0
−7.5
0.1
0.2
0.3
0.4
SNP effect on Osteosarcoma
SNP effect on 6ckFav

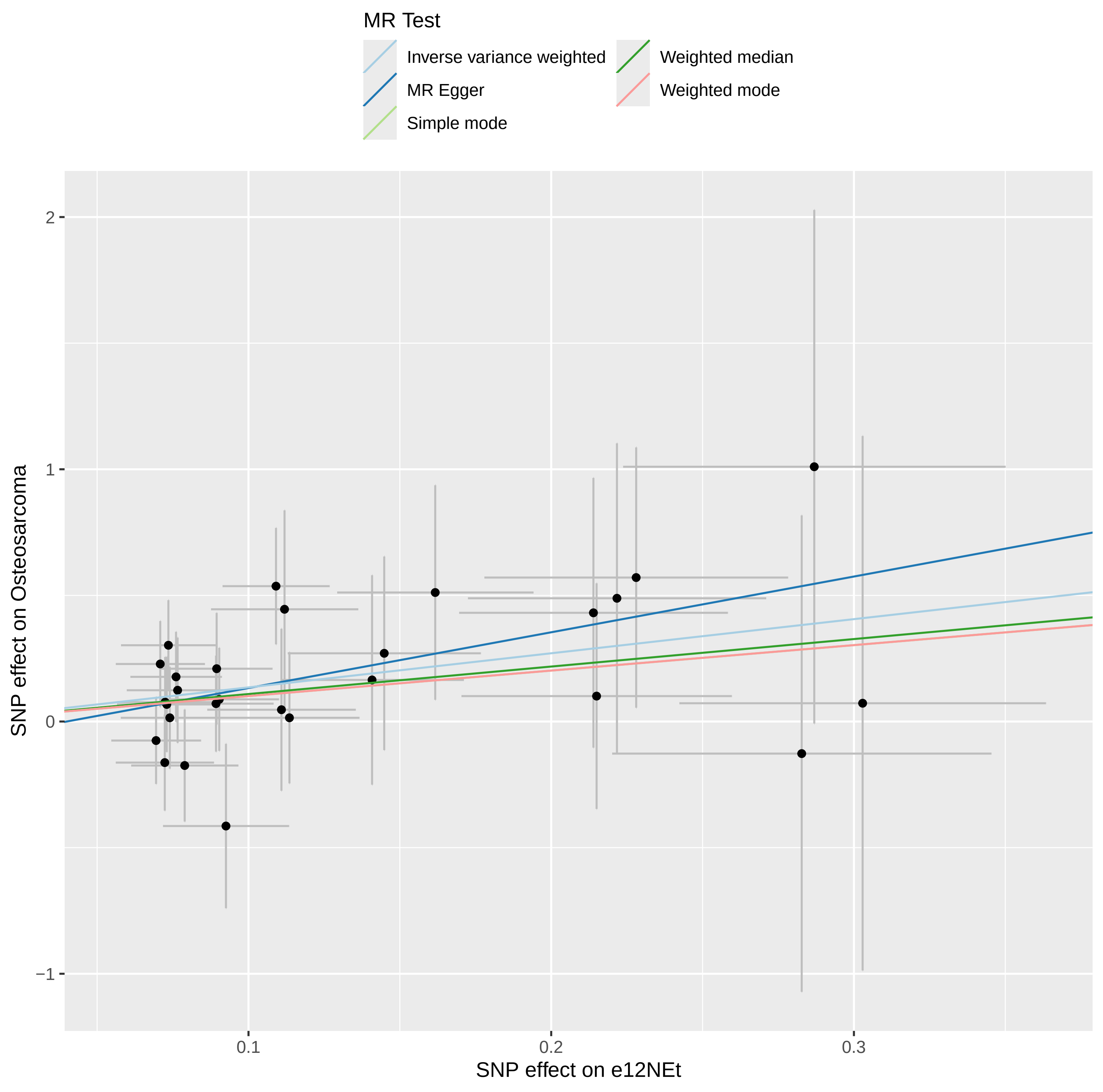

MR Test
Inverse variance weighted
Weighted median
MR Egger
Weighted mode
Simple mode
SNP effect on Osteosarcoma
SNP effect on e12NEt
2
1
0
−1
0.1
0.2
0.3

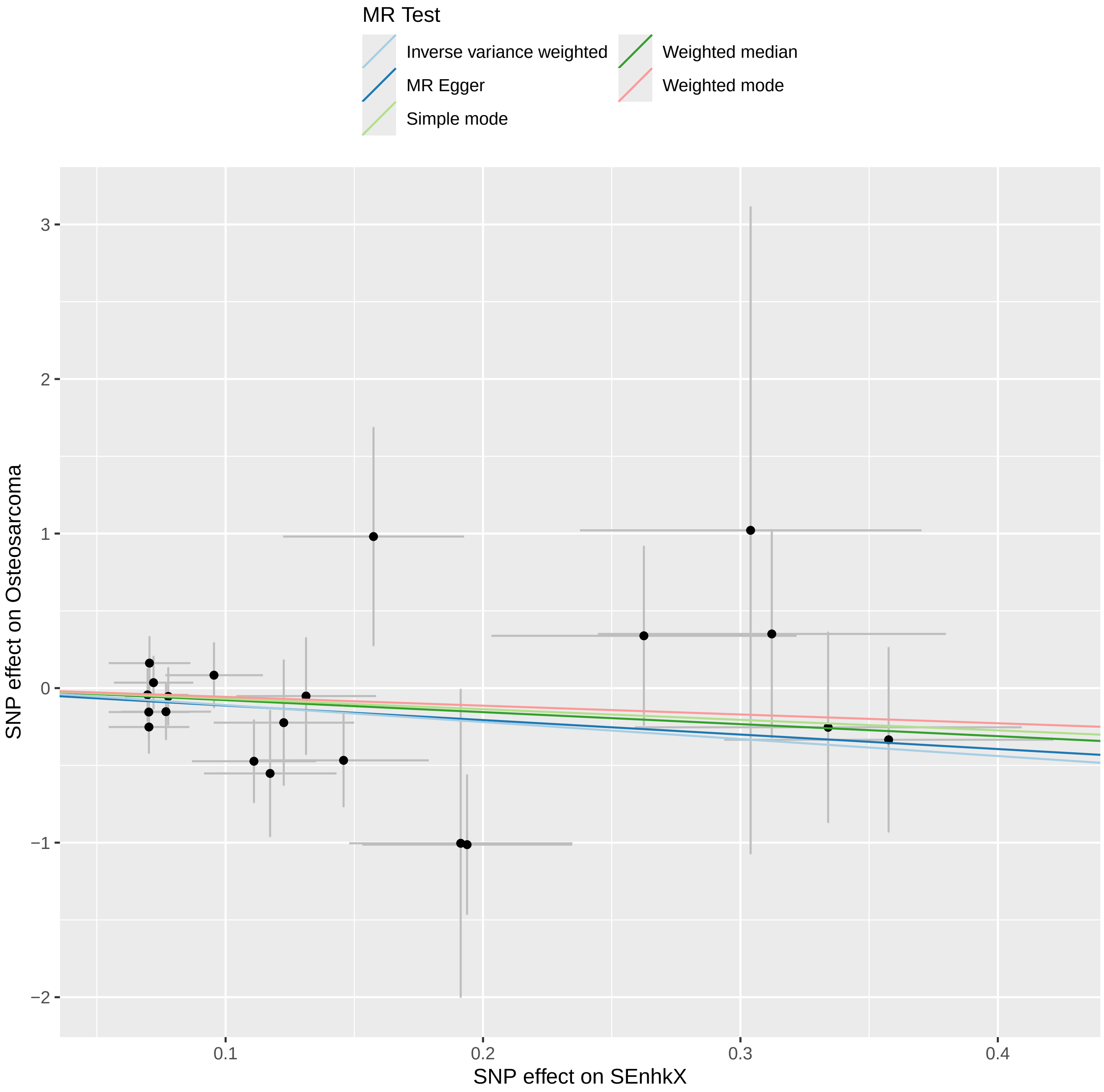

MR Test
Inverse variance weighted
MR Egger
Simple mode
Weighted median
Weighted mode
SNP effect on Osteosarcoma
3
2
1
0
−1
−2
0.1
0.2
0.3
0.4
SNP effect on SEnhkX

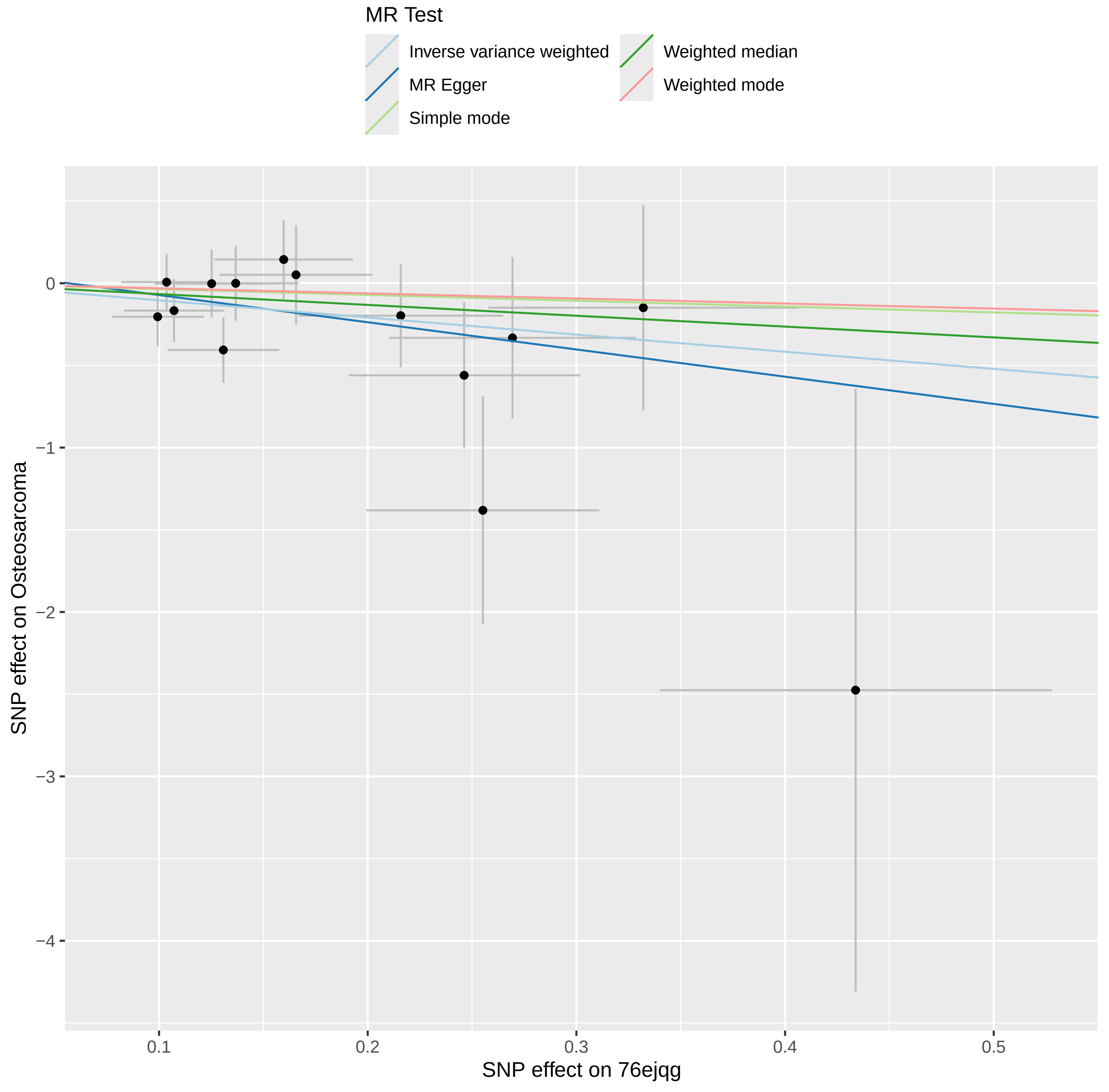
MR Test
Inverse variance weighted
MR Egger
Simple mode
Weighted median
Weighted mode
SNP effect on Osteosarcoma
0
−1
−2
−3
−4
0.1
0.2
0.3
0.4
0.5
SNP effect on 76ejqg

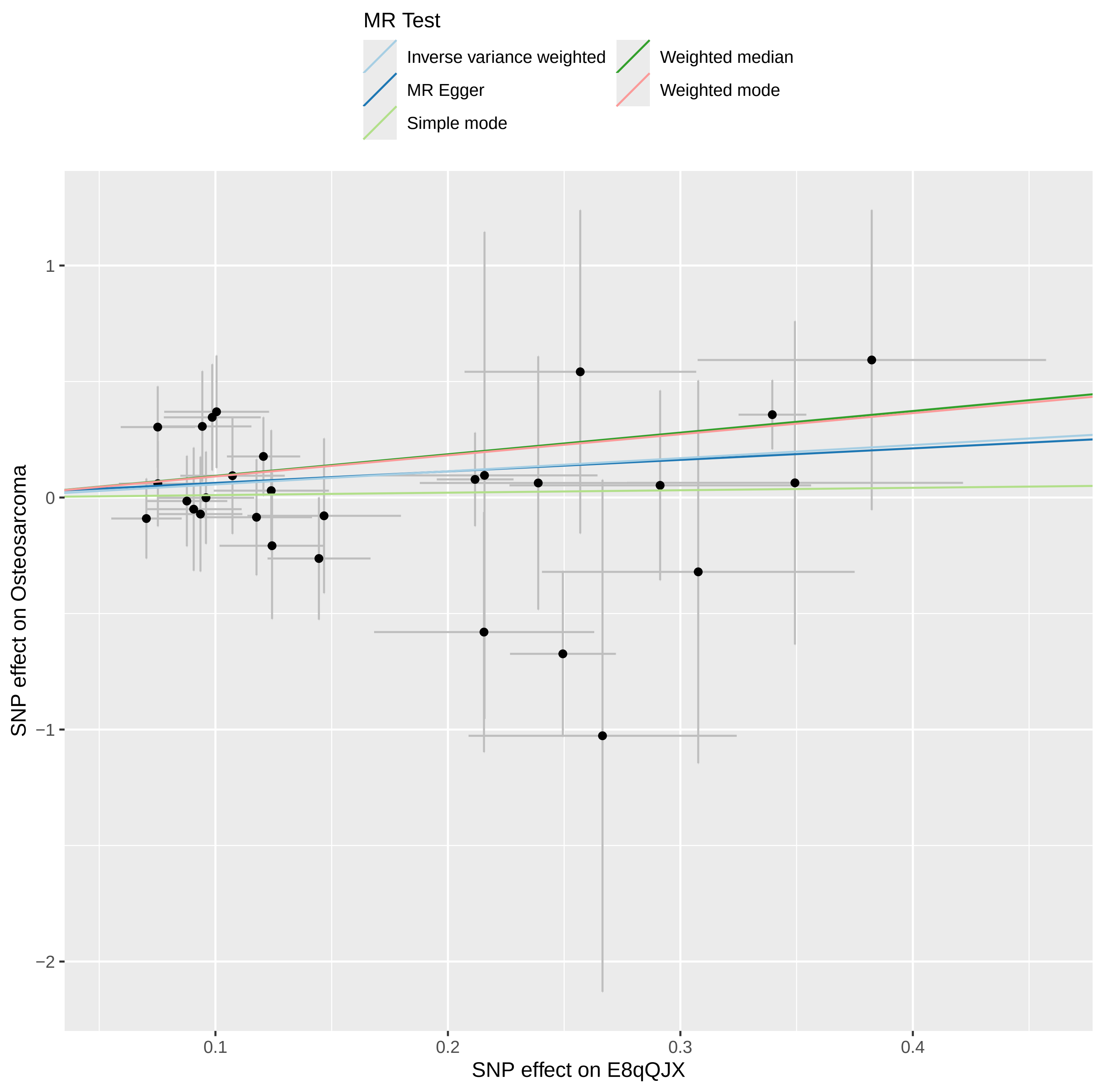

MR Test
Inverse variance weighted
MR Egger
Simple mode
Weighted median
Weighted mode
1
0
−1
−2
0.1
0.2
0.3
0.4
SNP effect on Osteosarcoma
SNP effect on E8qQJX

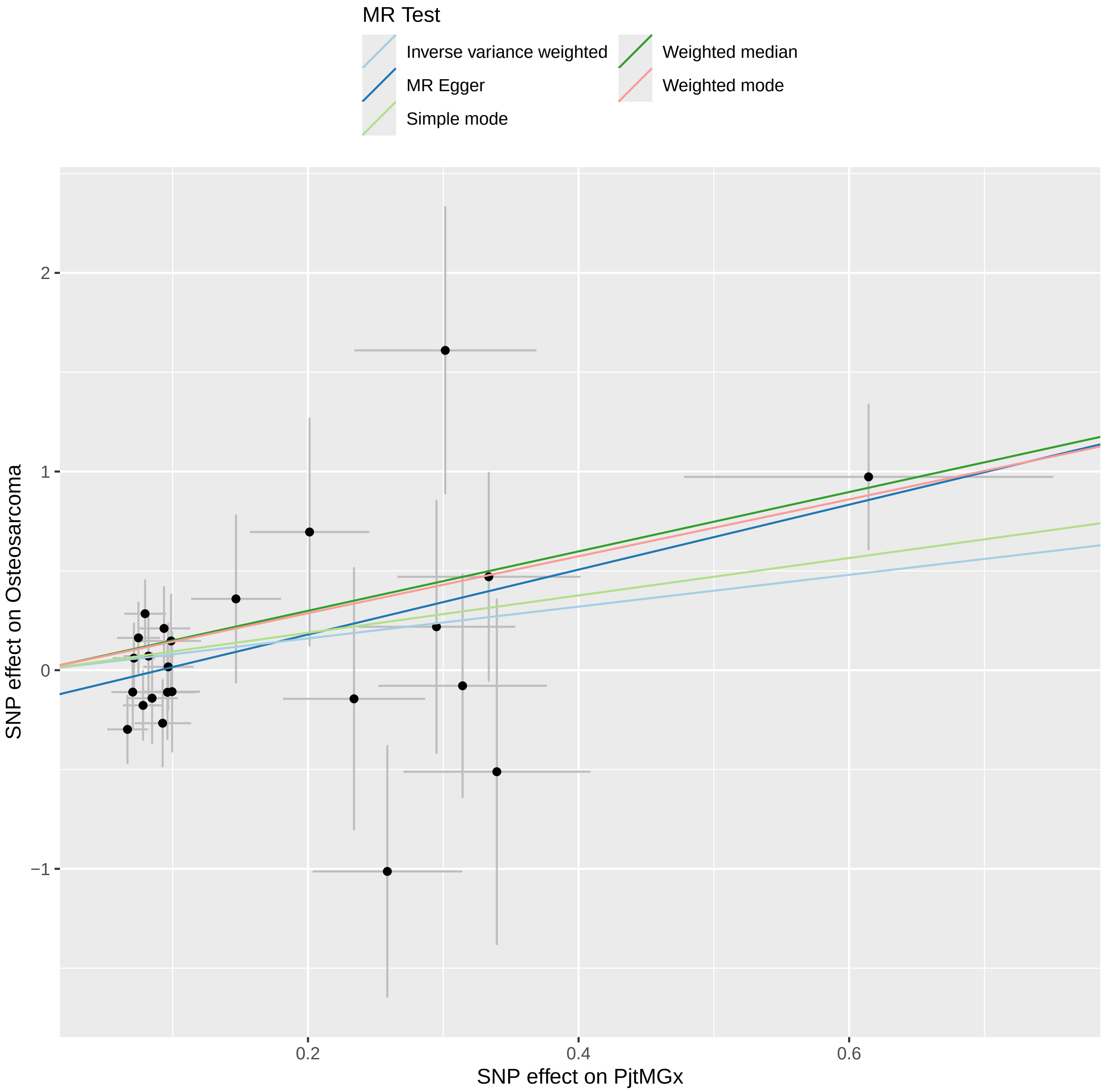

MR Test
Inverse variance weighted
MR Egger
Simple mode
Weighted median
Weighted mode
SNP effect on Osteosarcoma
2
1
0
−1
0.2
0.4
0.6
SNP effect on PjtMGx

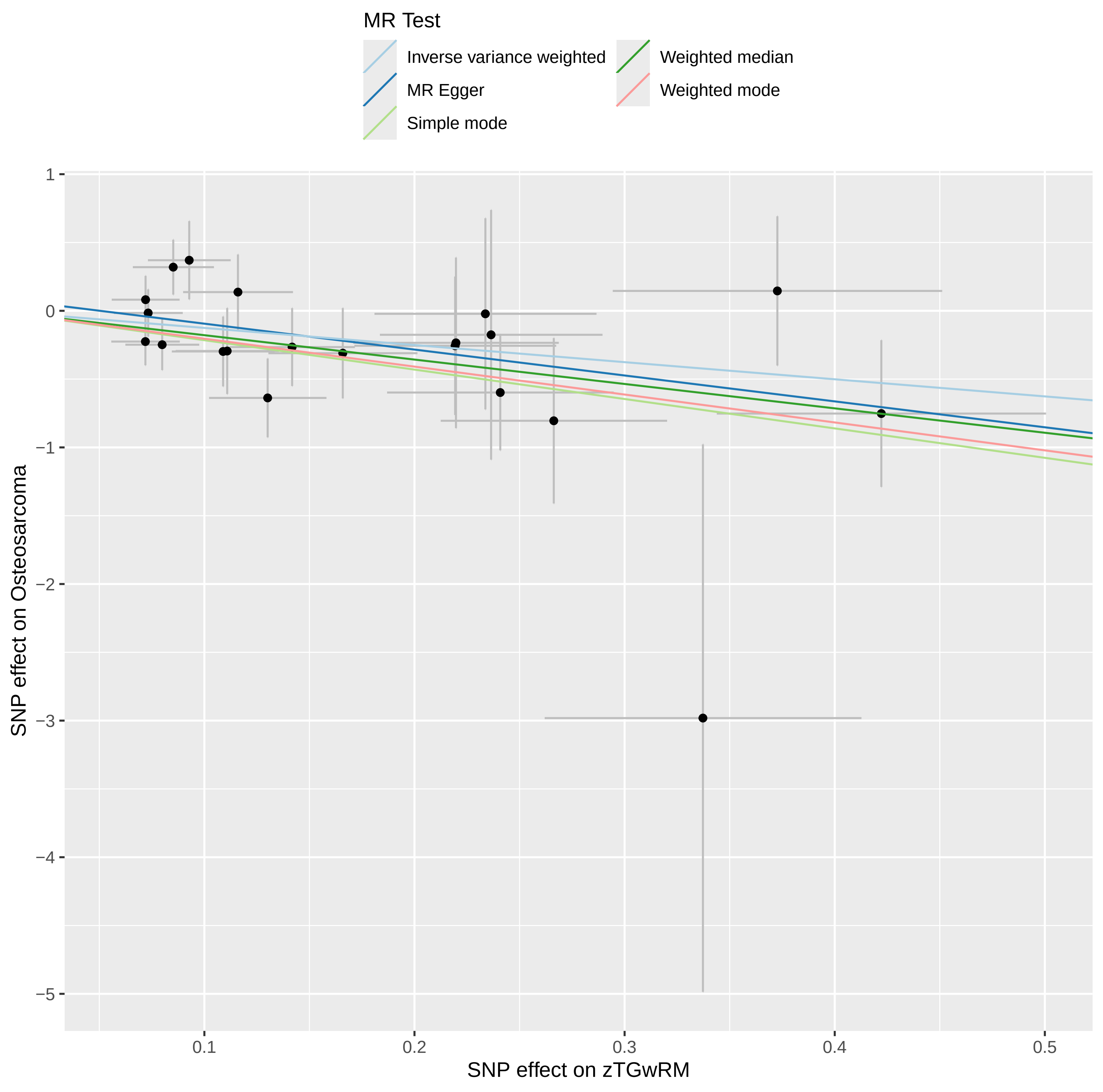

MR Test
Inverse variance weighted
MR Egger
Simple mode
Weighted median
Weighted mode
SNP effect on Osteosarcoma
SNP effect on zTGwRM
1
0
−1
−2
−3
−4
−5
0.1
0.2
0.3
0.4
0.5

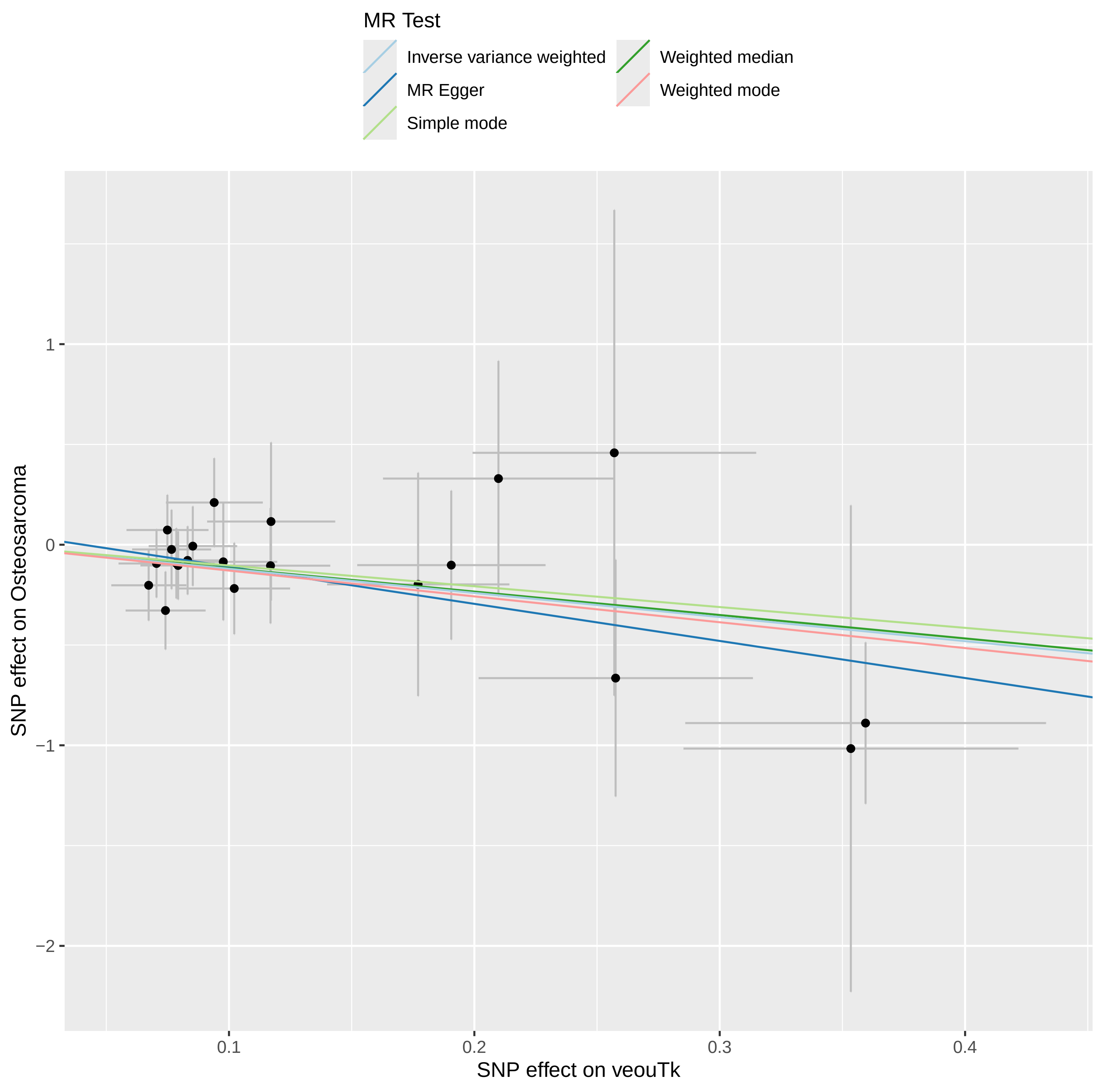

MR Test
Inverse variance weighted
MR Egger
Simple mode
Weighted median
Weighted mode
1
0
−1
−2
SNP effect on Osteosarcoma
0.1
0.2
0.3
0.4
SNP effect on veouTk

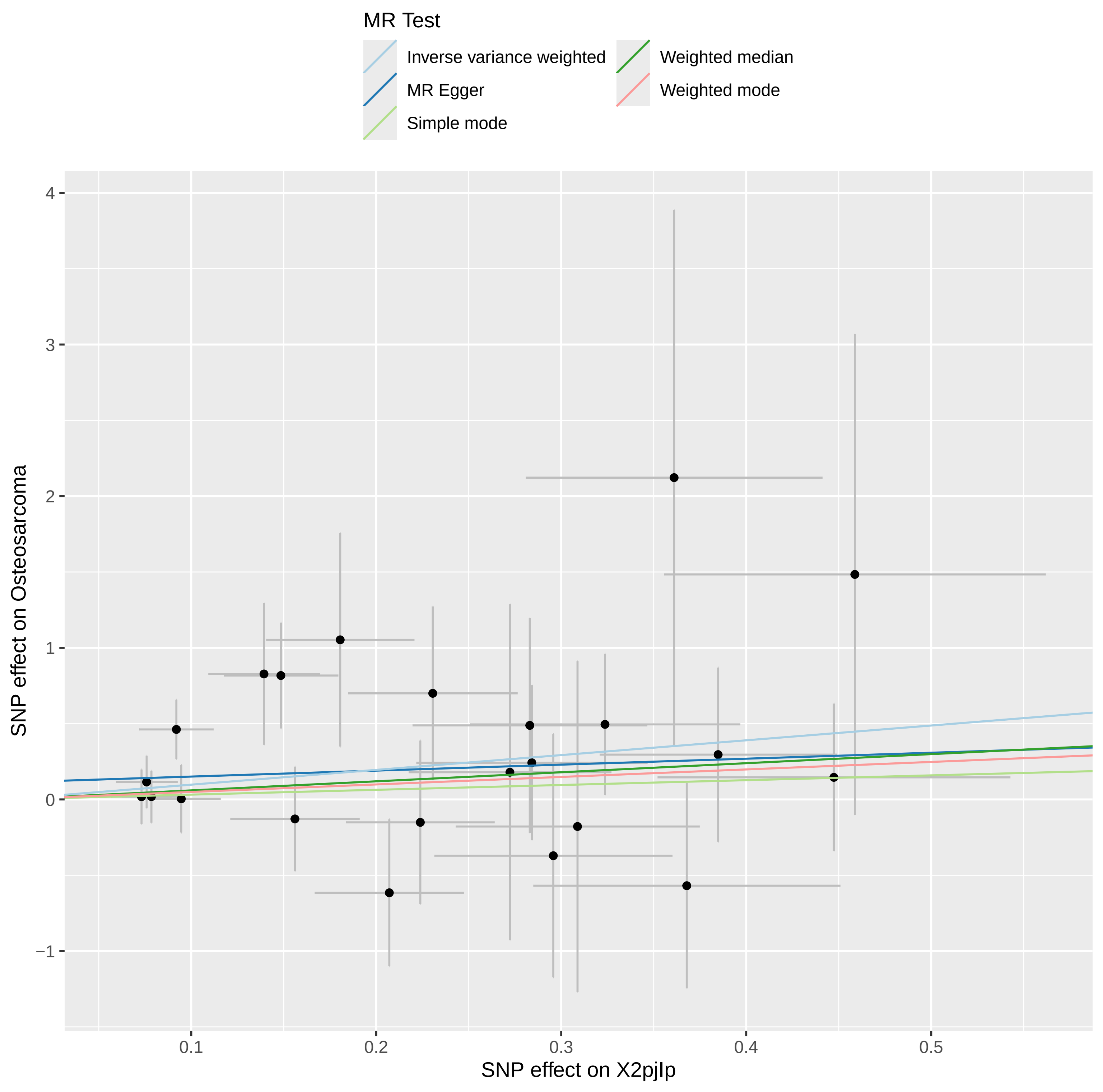

MR Test
Inverse variance weighted
MR Egger
Simple mode
Weighted median
Weighted mode
SNP effect on Osteosarcoma
4
3
2
1
0
−1
0.1
0.2
0.3
0.4
0.5
SNP effect on X2pjIp

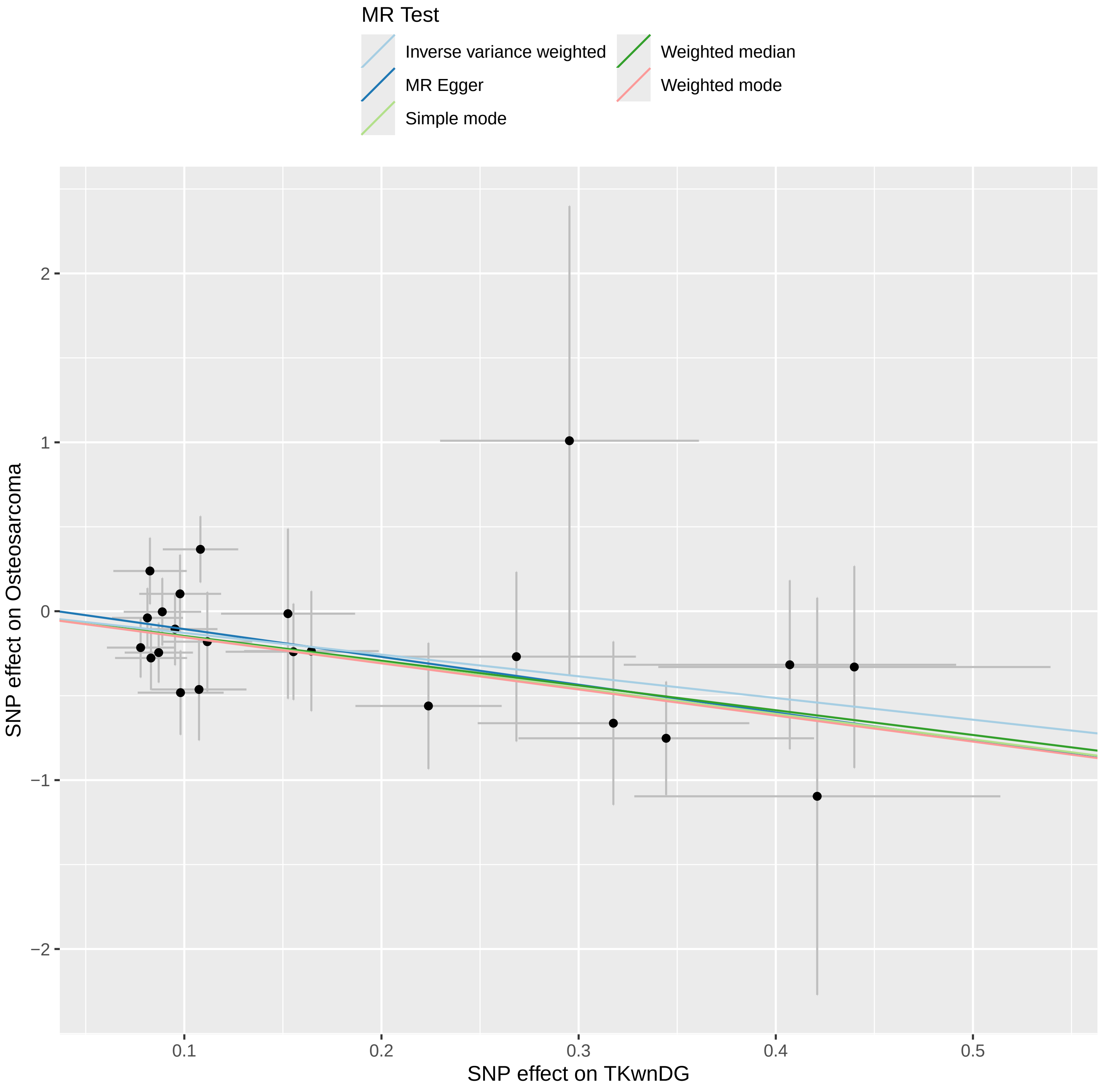

MR Test
Inverse variance weighted
MR Egger
Simple mode
Weighted median
Weighted mode
2
1
0
−1
−2
SNP effect on Osteosarcoma
0.1
0.2
0.3
0.4
0.5
SNP effect on TKwnDG

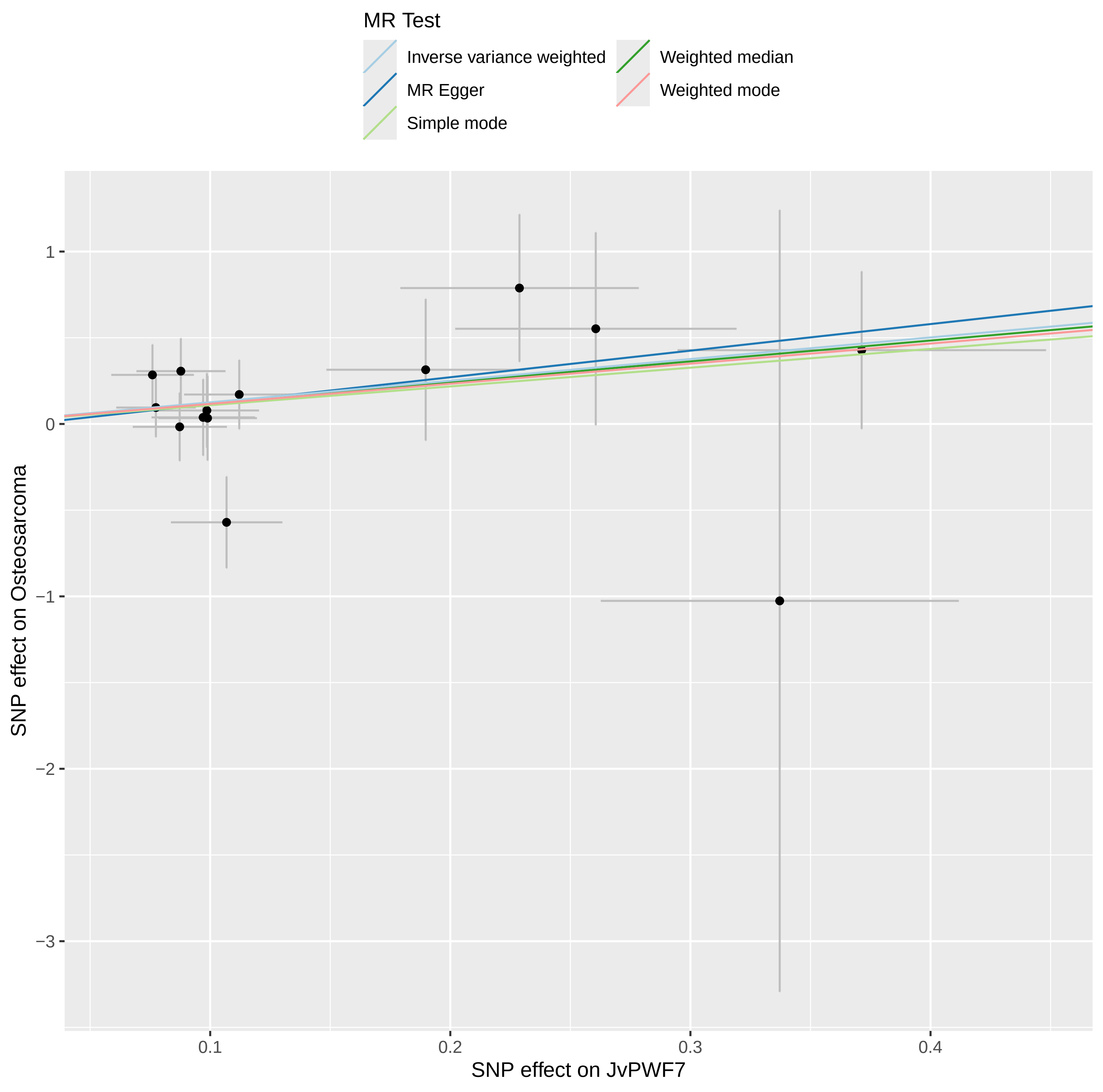

MR Test
Inverse variance weighted
MR Egger
Simple mode
Weighted median
Weighted mode
1
0
−1
−2
−3
SNP effect on Osteosarcoma
0.1
0.2
0.3
0.4
SNP effect on JvPWF7

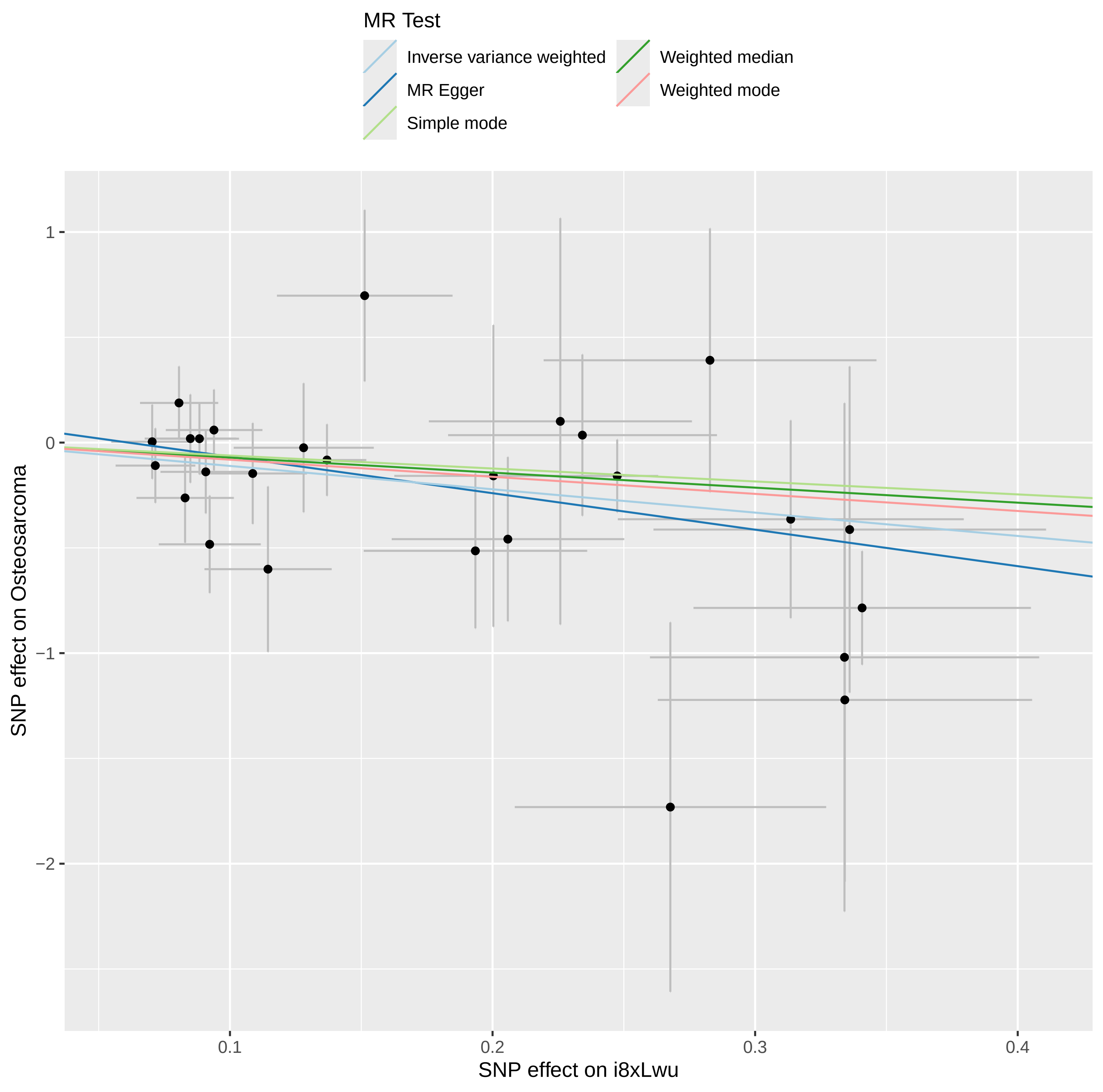
MR Test
Inverse variance weighted
MR Egger
Simple mode
Weighted median
Weighted mode
1
0
−1
−2
0.1
0.2
0.3
0.4
SNP effect on Osteosarcoma
SNP effect on i8xLwu

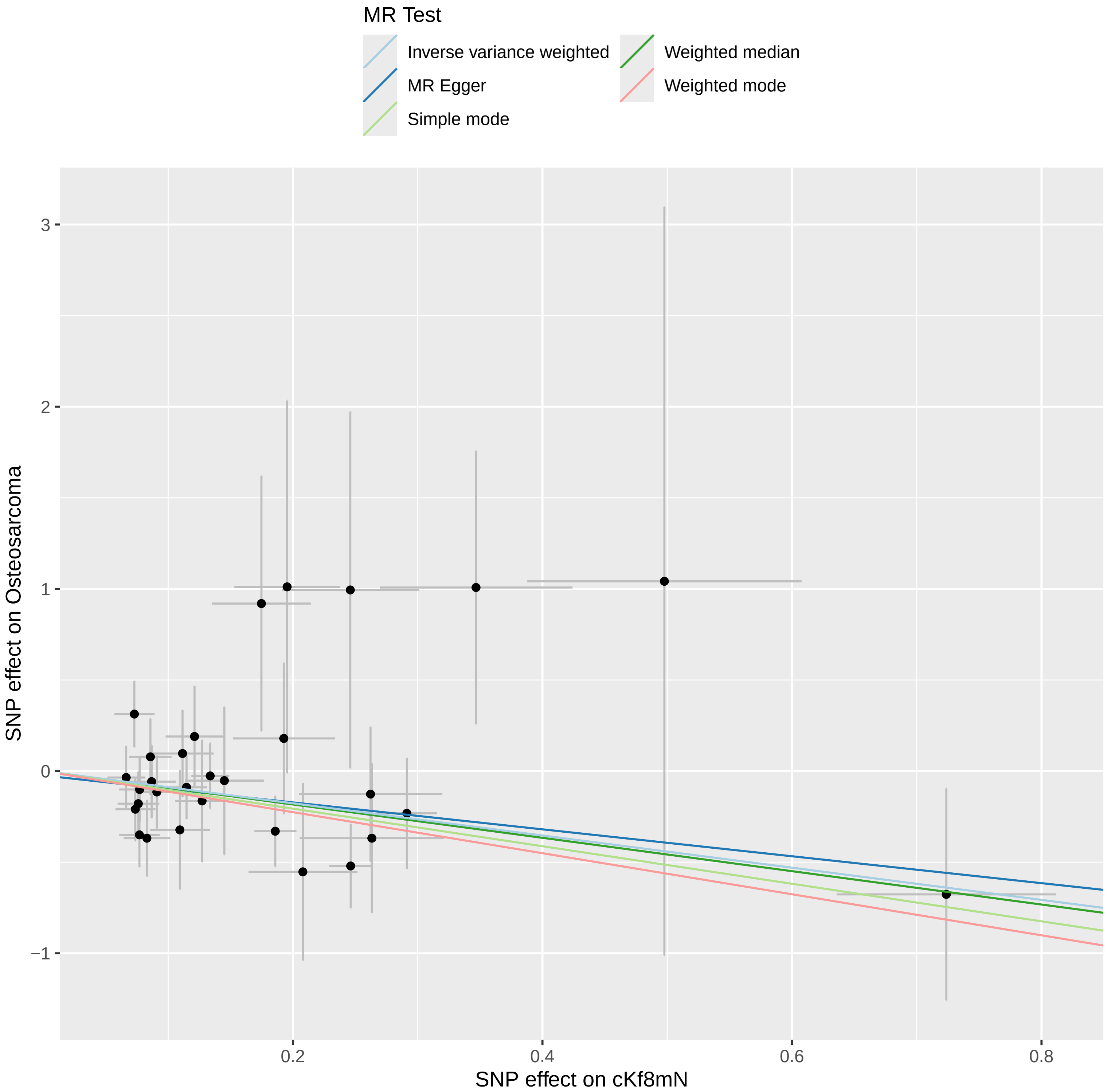

MR Test
Inverse variance weighted
MR Egger
Simple mode
Weighted median
Weighted mode
SNP effect on Osteosarcoma
3
2
1
0
−1
0.2
0.4
0.6
0.8
SNP effect on cKf8mN

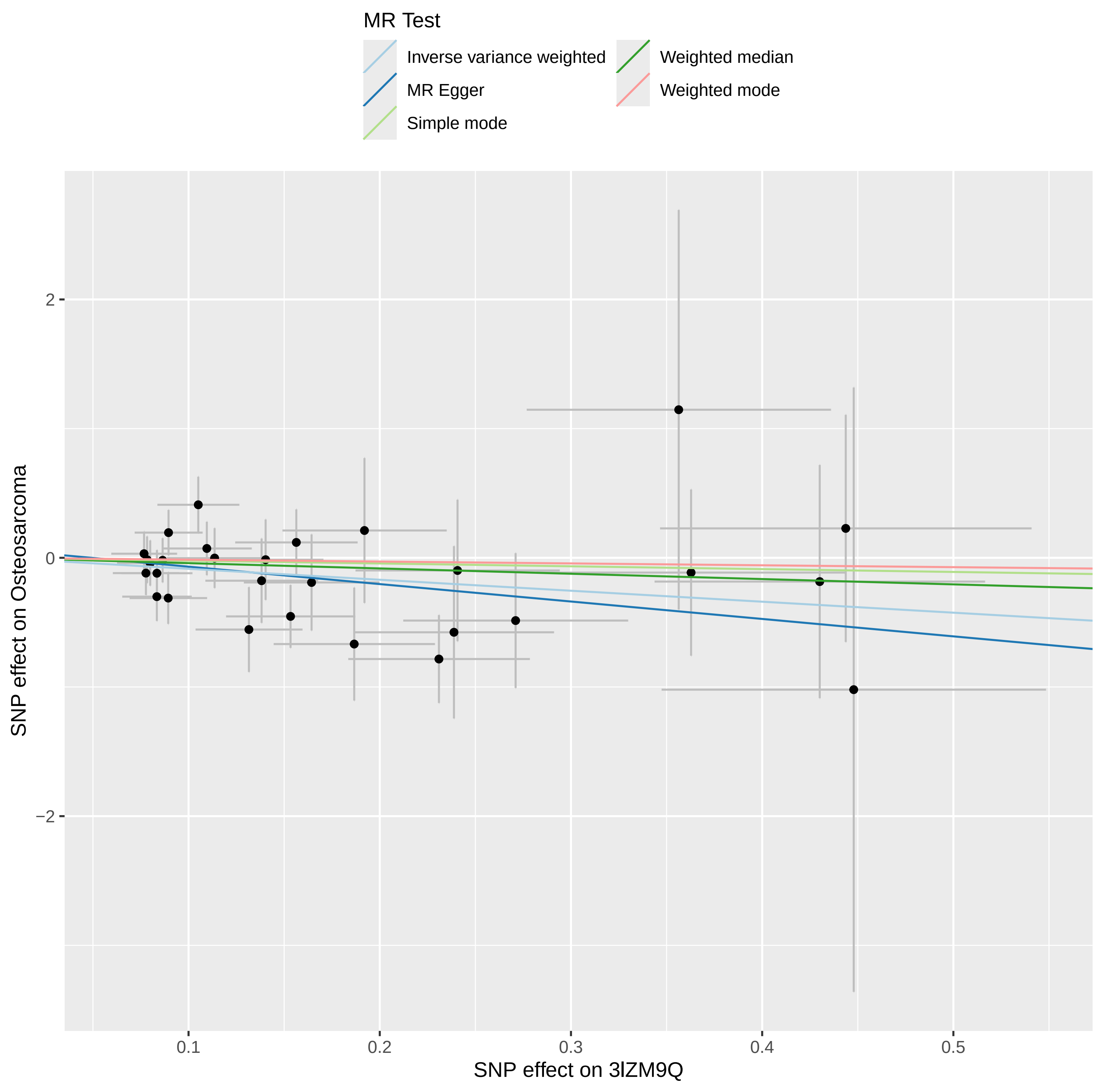

MR Test
Inverse variance weighted
MR Egger
Simple mode
Weighted median
Weighted mode
SNP effect on Osteosarcoma
2
0
−2
0.1
0.2
0.3
0.4
0.5
SNP effect on 3lZM9Q

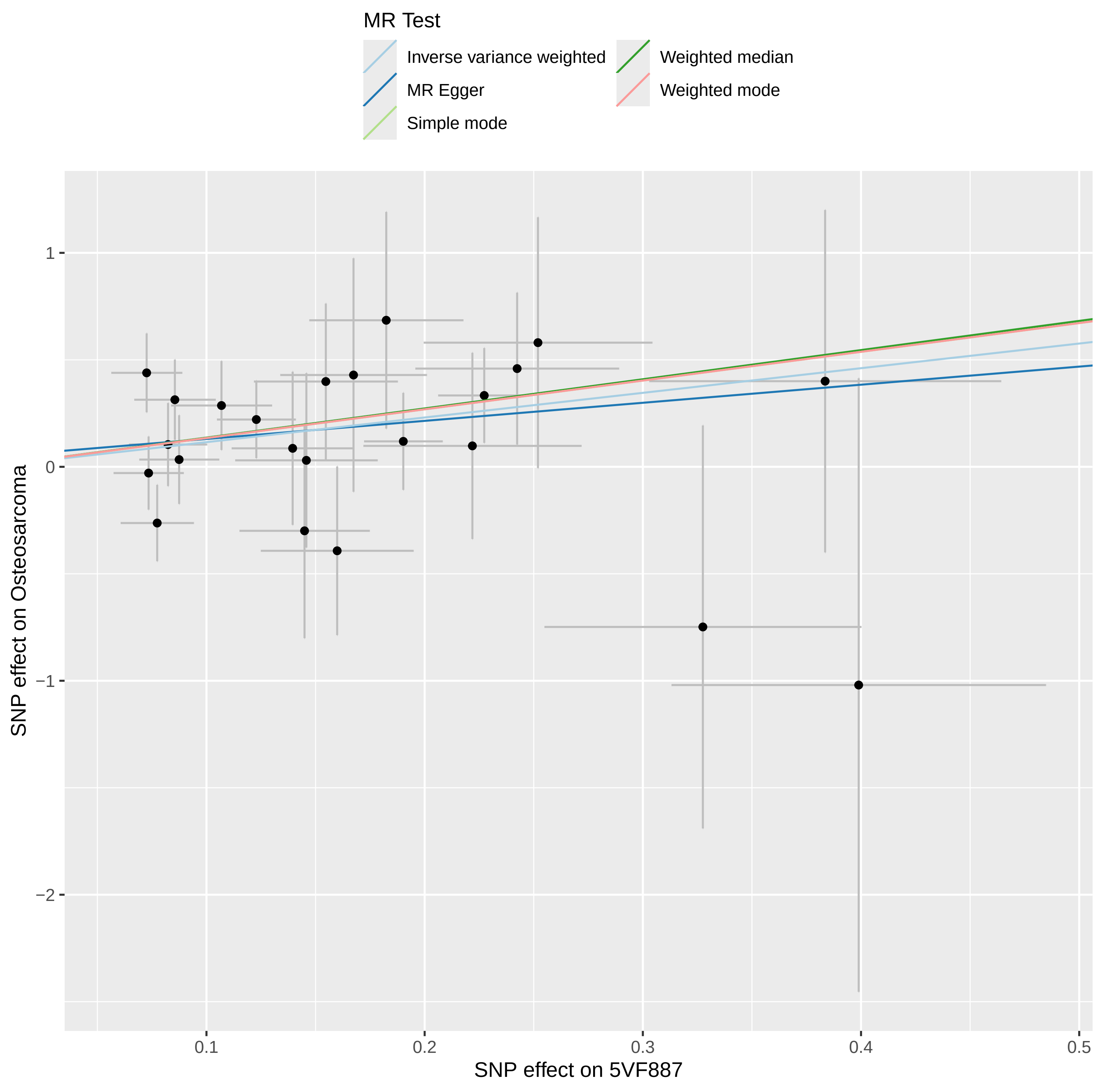

MR Test
Inverse variance weighted
MR Egger
Simple mode
Weighted median
Weighted mode
1
0
−1
−2
0.1
0.2
0.3
0.4
0.5
SNP effect on Osteosarcoma
SNP effect on 5VF887

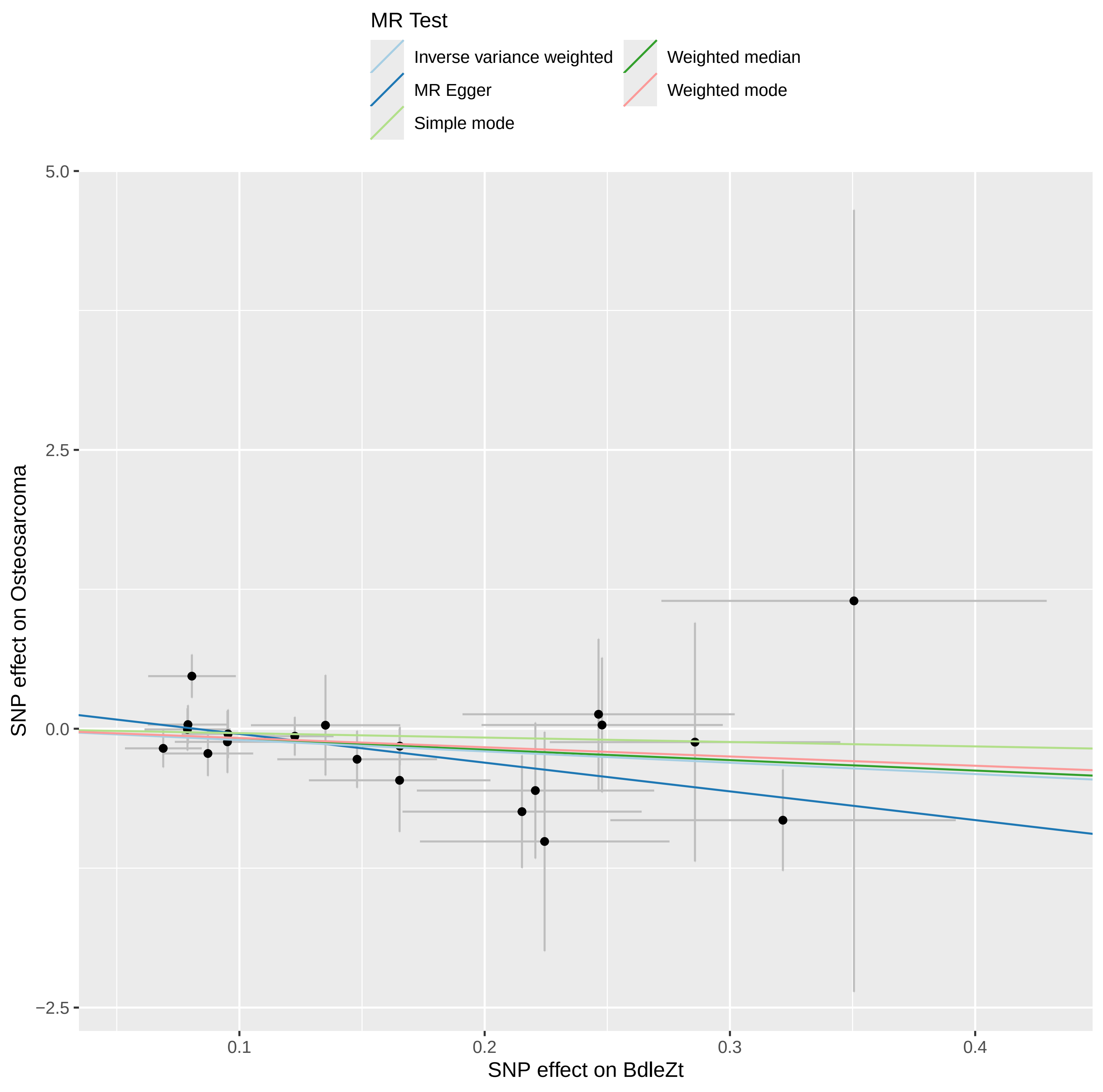
MR Test
Inverse variance weighted
MR Egger
Simple mode
Weighted median
Weighted mode
5.0
2.5
0.0
−2.5
0.1
0.2
0.3
0.4
SNP effect on Osteosarcoma
SNP effect on BdleZt

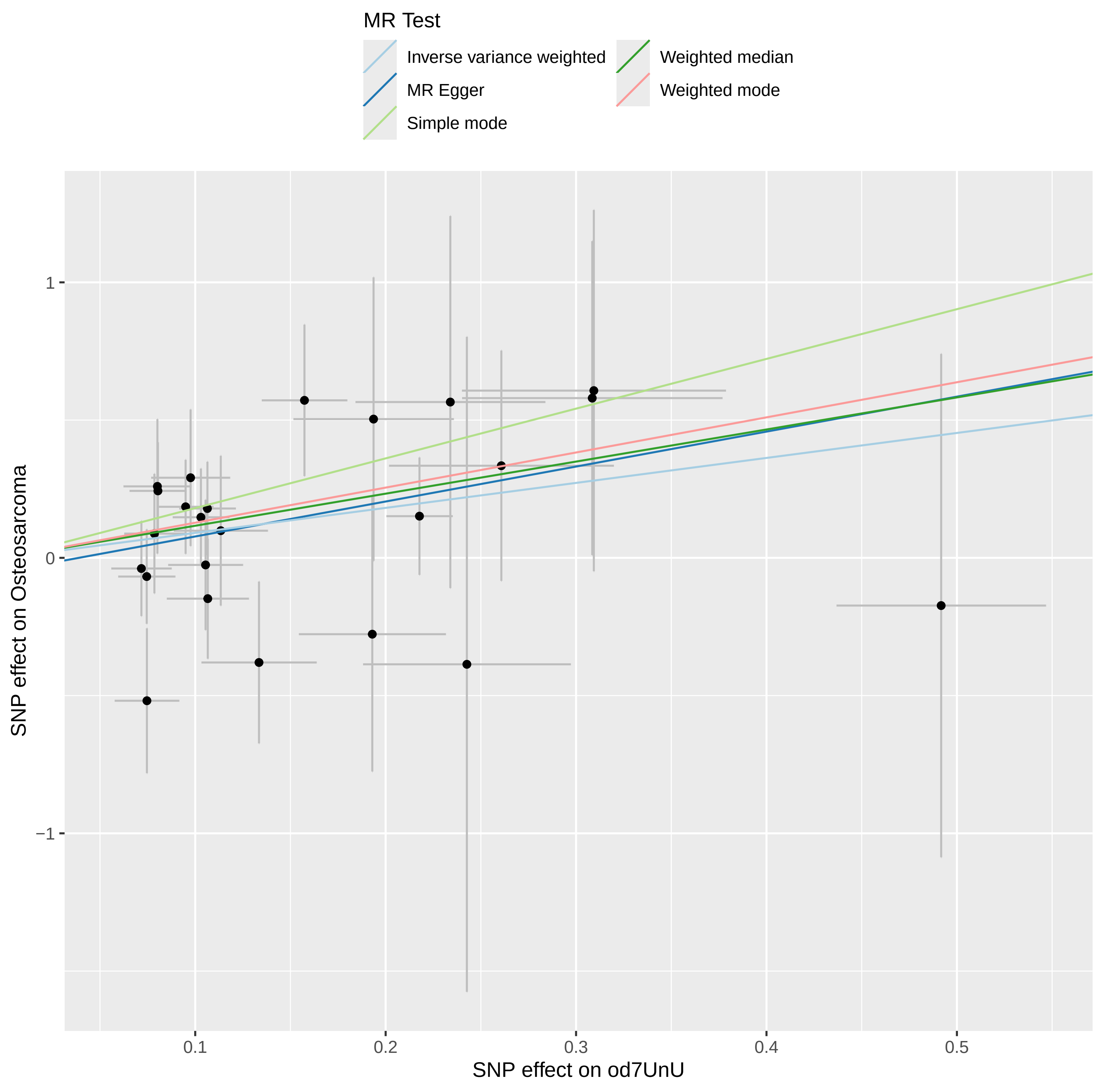

MR Test
Inverse variance weighted
MR Egger
Simple mode
Weighted median
Weighted mode
SNP effect on Osteosarcoma
1
0
−1
0.1
0.2
0.3
0.4
0.5
SNP effect on od7UnU

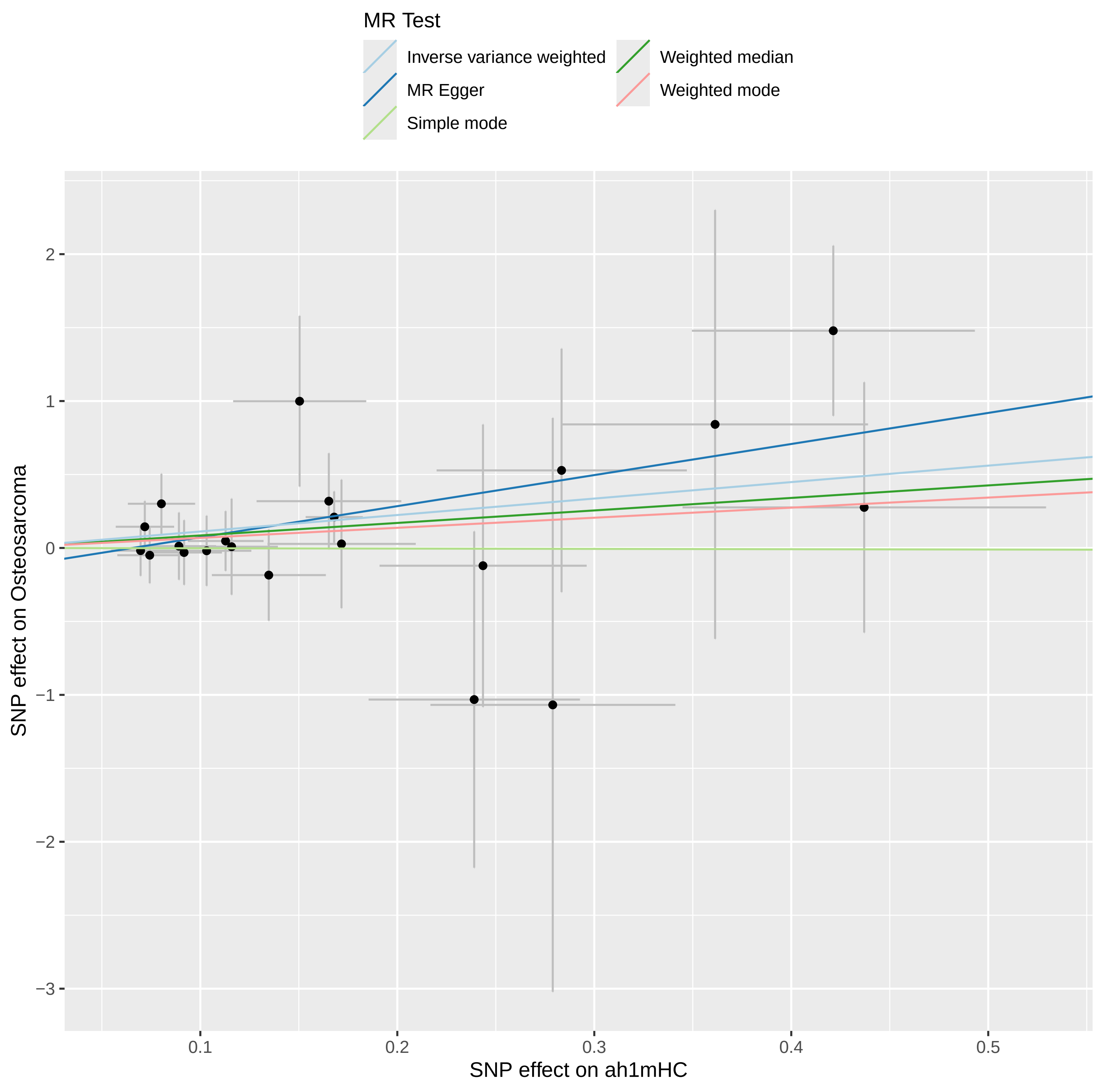

MR Test
Inverse variance weighted
MR Egger
Simple mode
Weighted median
Weighted mode
SNP effect on Osteosarcoma
2
1
0
−1
−2
−3
0.1
0.2
0.3
0.4
0.5
SNP effect on ah1mHC

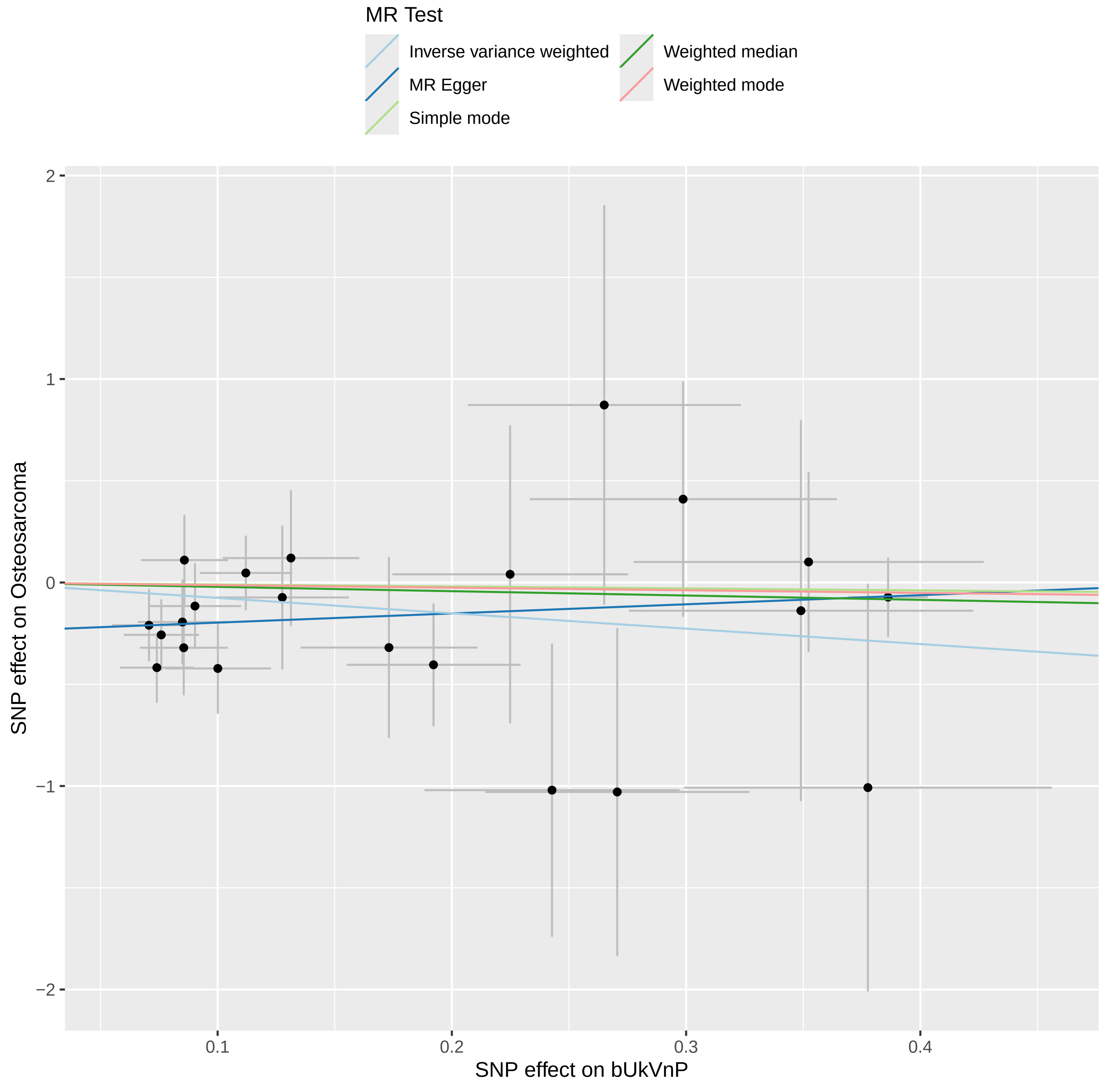

MR Test
Inverse variance weighted
MR Egger
Simple mode
Weighted median
Weighted mode
2
1
0
−1
−2
0.1
0.2
0.3
0.4
SNP effect on Osteosarcoma
SNP effect on bUkVnP

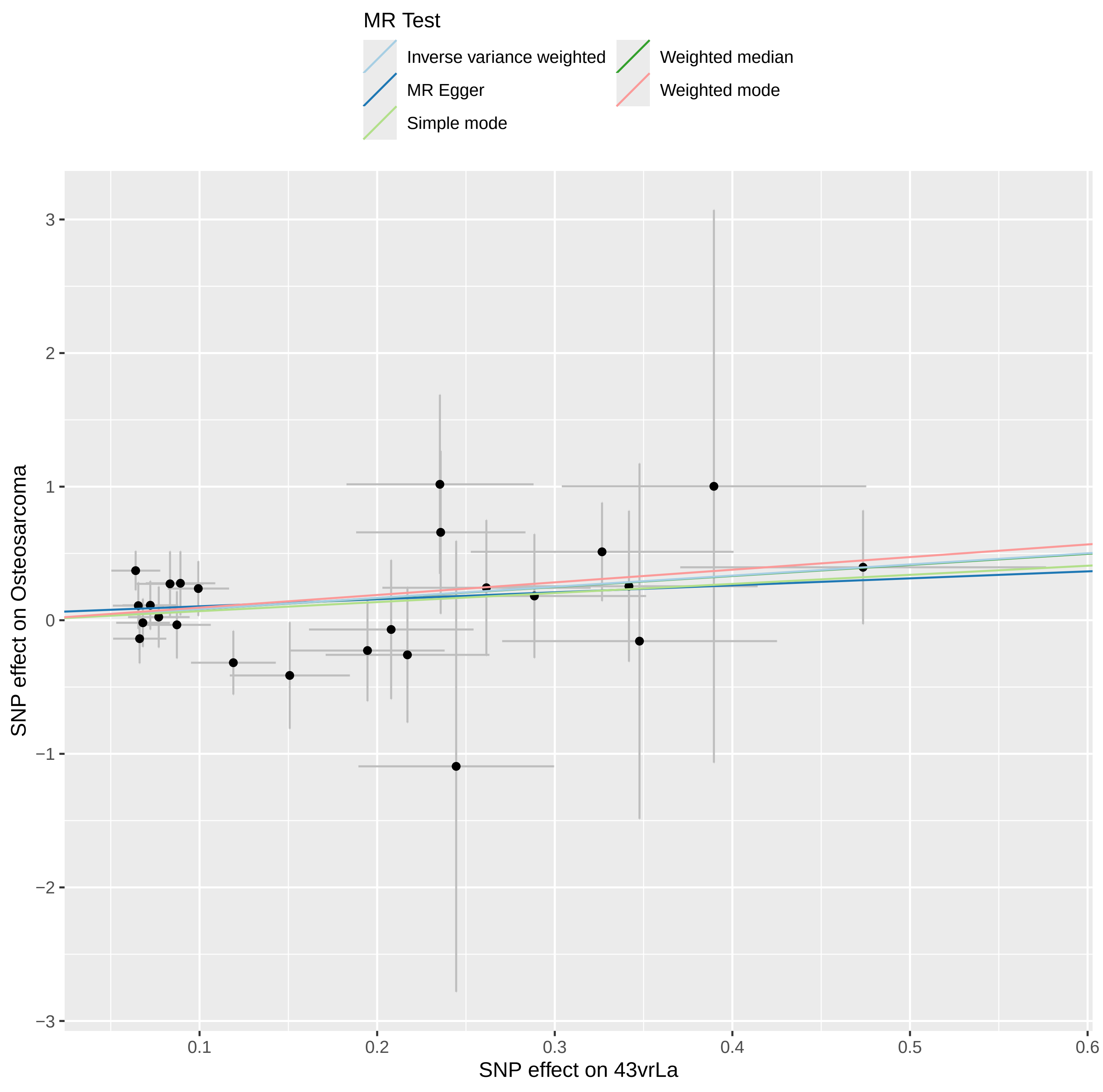
MR Test
Inverse variance weighted
MR Egger
Simple mode
Weighted median
Weighted mode
SNP effect on Osteosarcoma
SNP effect on 43vrLa
3
2
1
0
−1
−2
−3
0.1
0.2
0.3
0.4
0.5
0.6

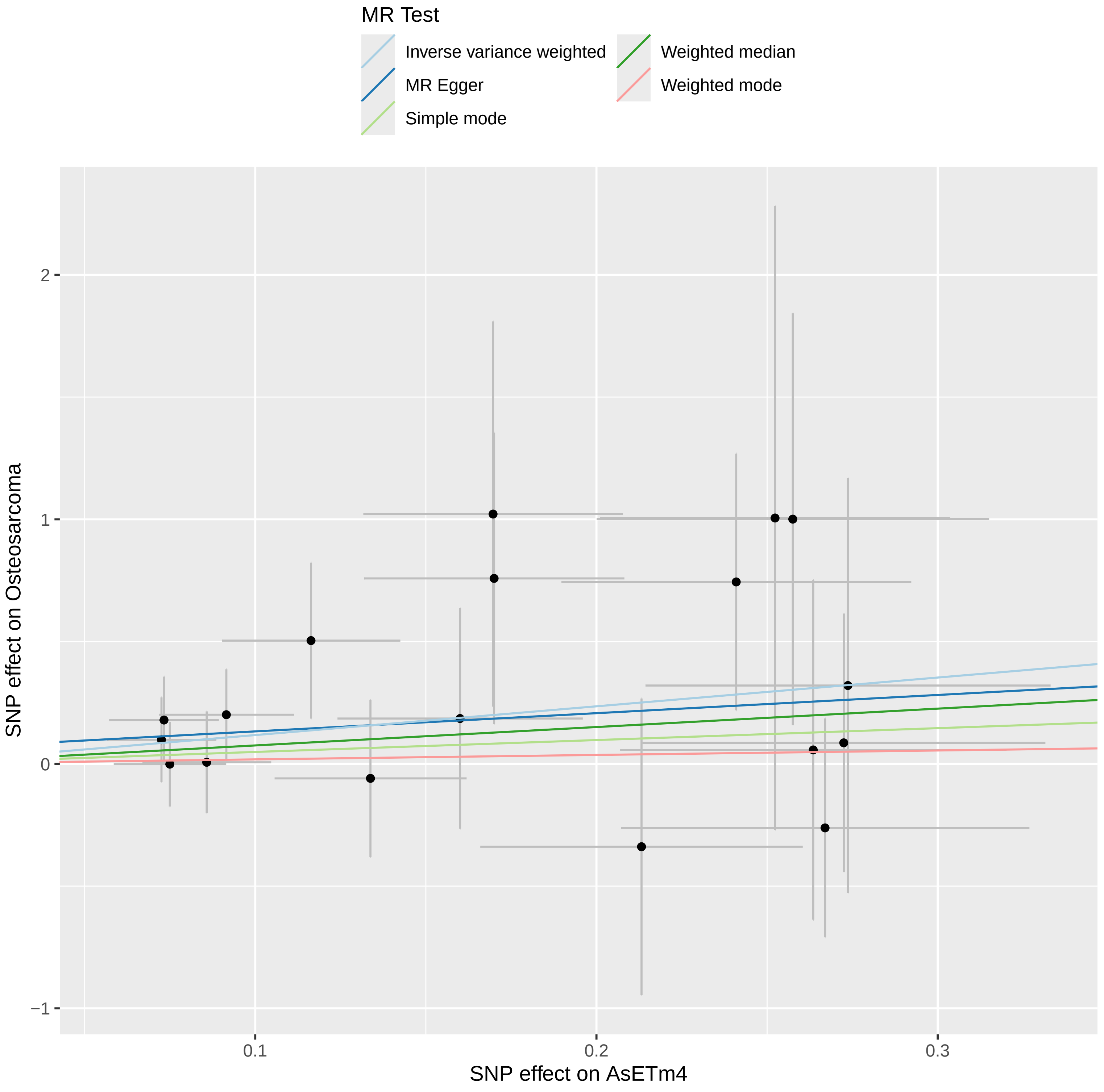

MR Test
Inverse variance weighted
MR Egger
Simple mode
Weighted median
Weighted mode
SNP effect on Osteosarcoma
2
1
0
−1
0.1
0.2
0.3
SNP effect on AsETm4

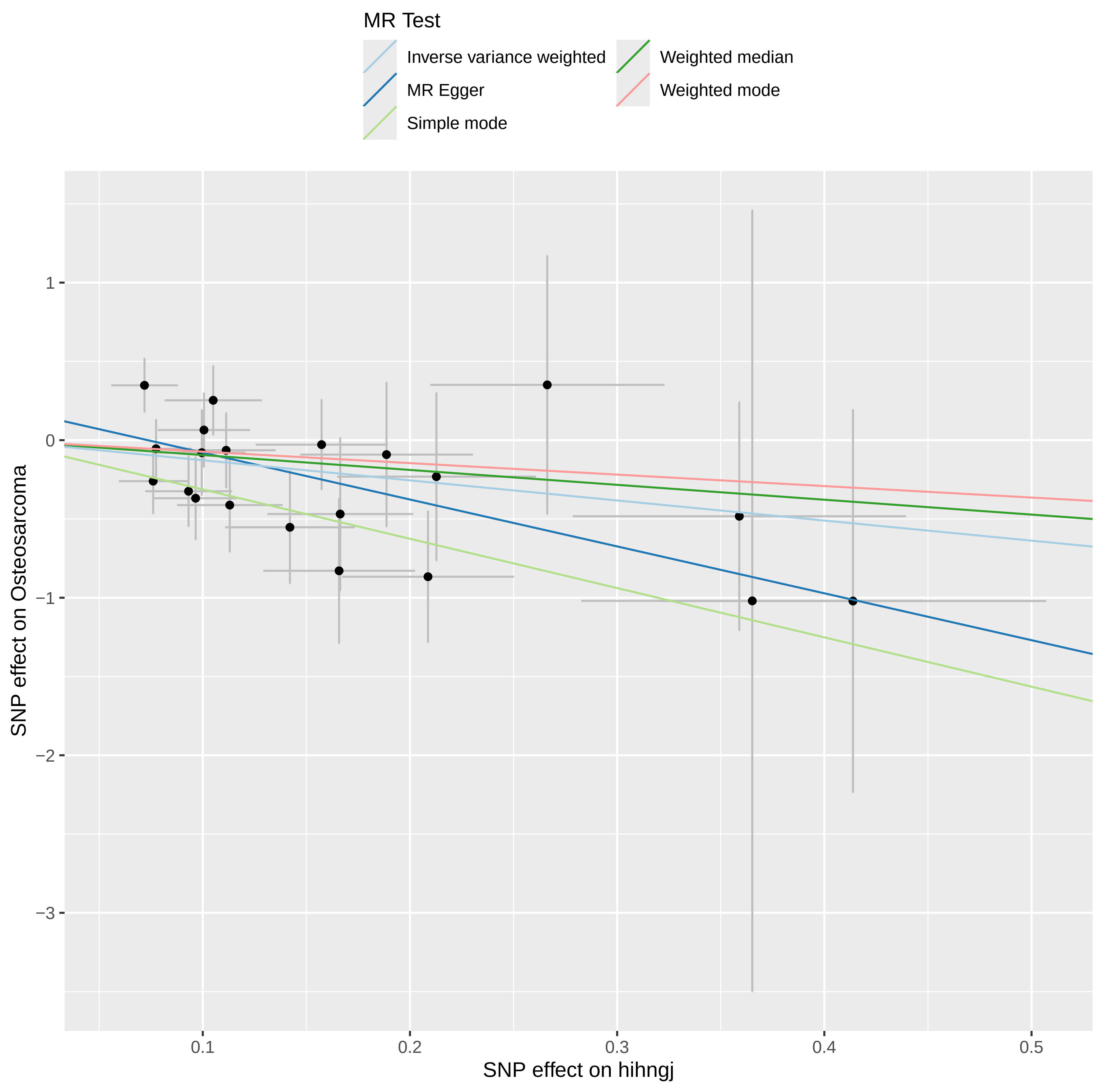

MR Test
Inverse variance weighted
MR Egger
Simple mode
Weighted median
Weighted mode
SNP effect on Osteosarcoma
1
0
−1
−2
−3
0.1
0.2
0.3
0.4
0.5
SNP effect on hihngj

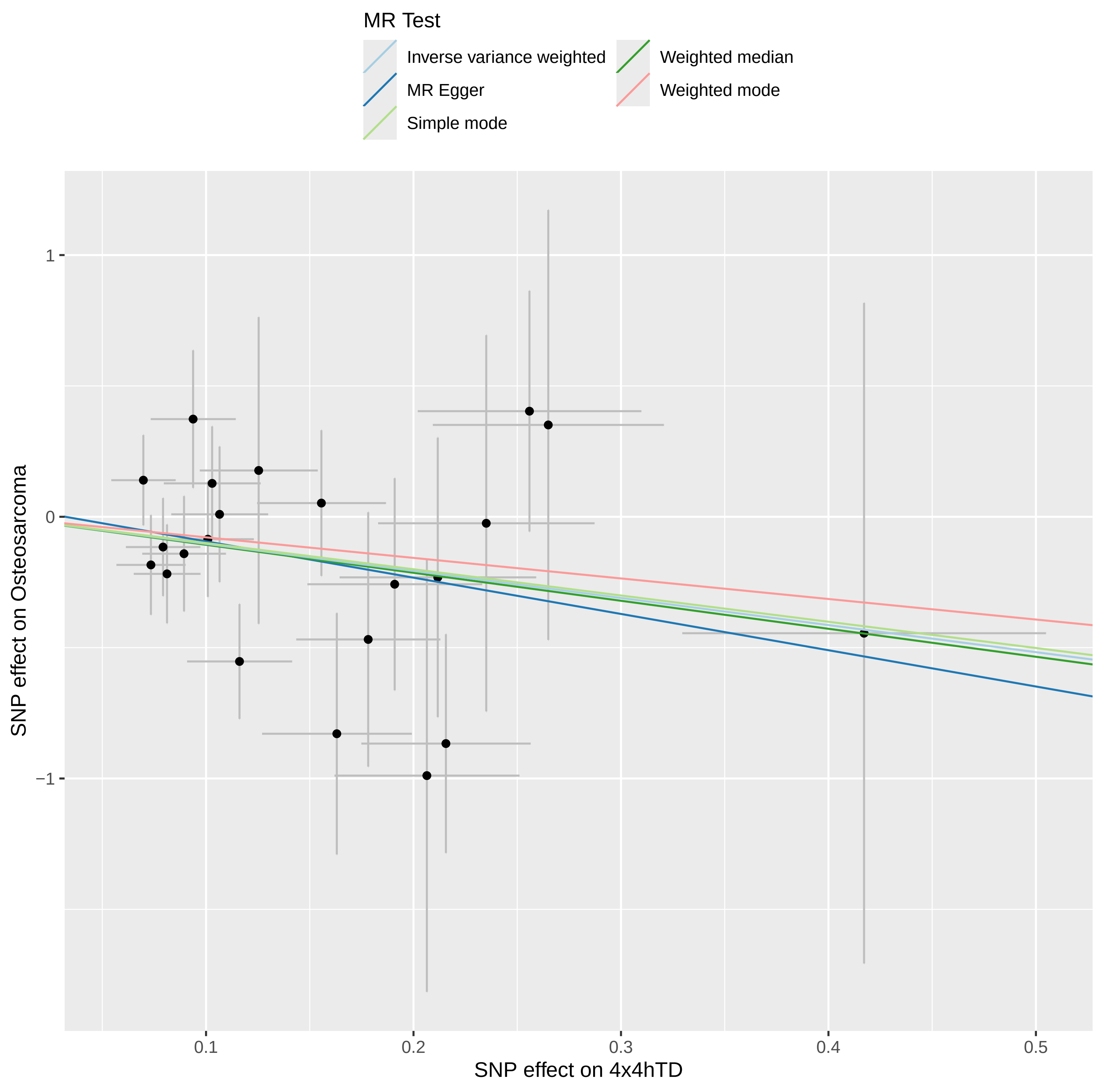
MR Test
Inverse variance weighted
MR Egger
Simple mode
Weighted median
Weighted mode
1
0
−1
SNP effect on Osteosarcoma
0.1
0.2
0.3
0.4
0.5
SNP effect on 4x4hTD

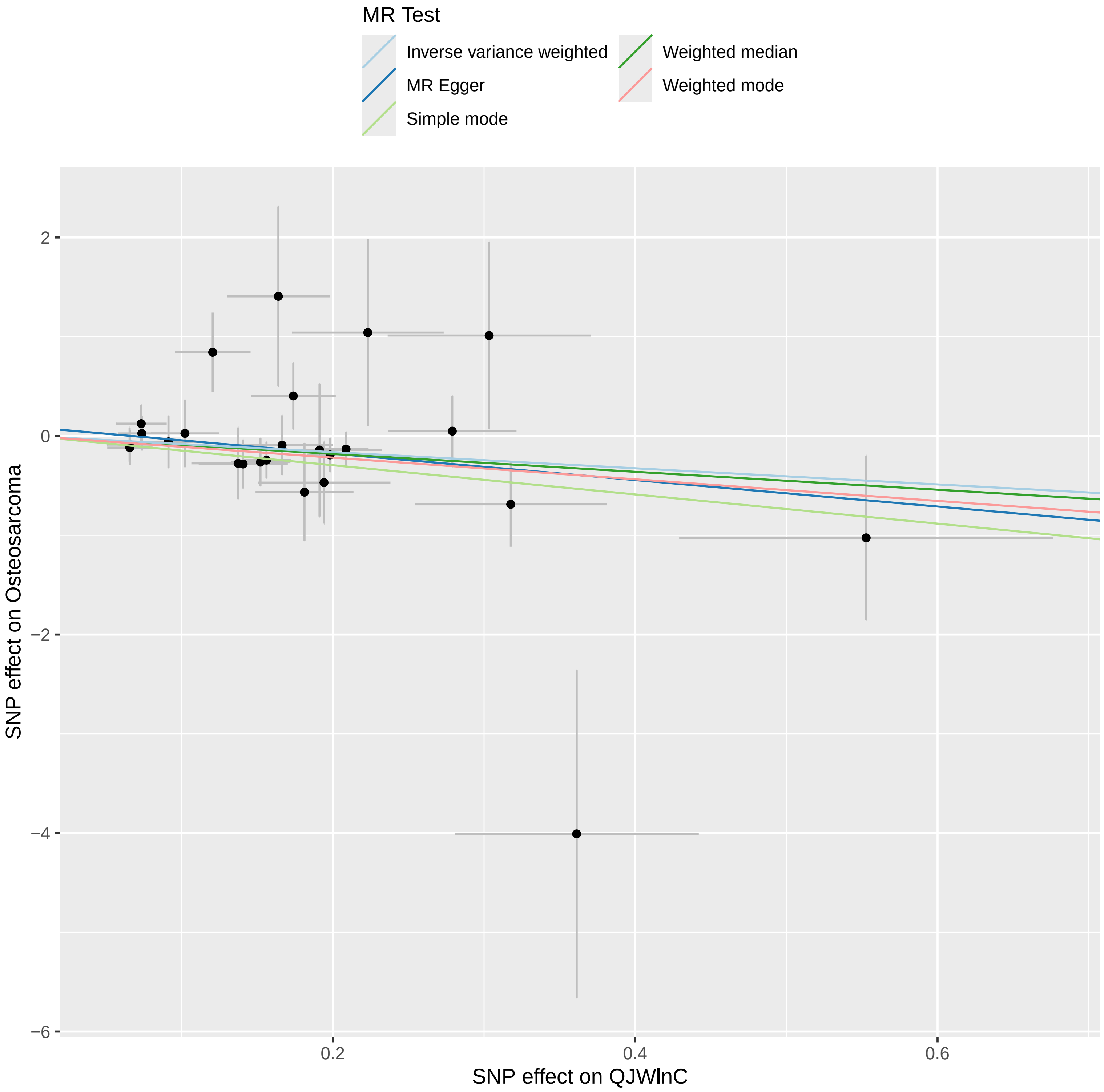
MR Test
Inverse variance weighted
MR Egger
Simple mode
Weighted median
Weighted mode
SNP effect on Osteosarcoma
SNP effect on QJWlnC
2
0
−2
−4
−6
0.2
0.4
0.6

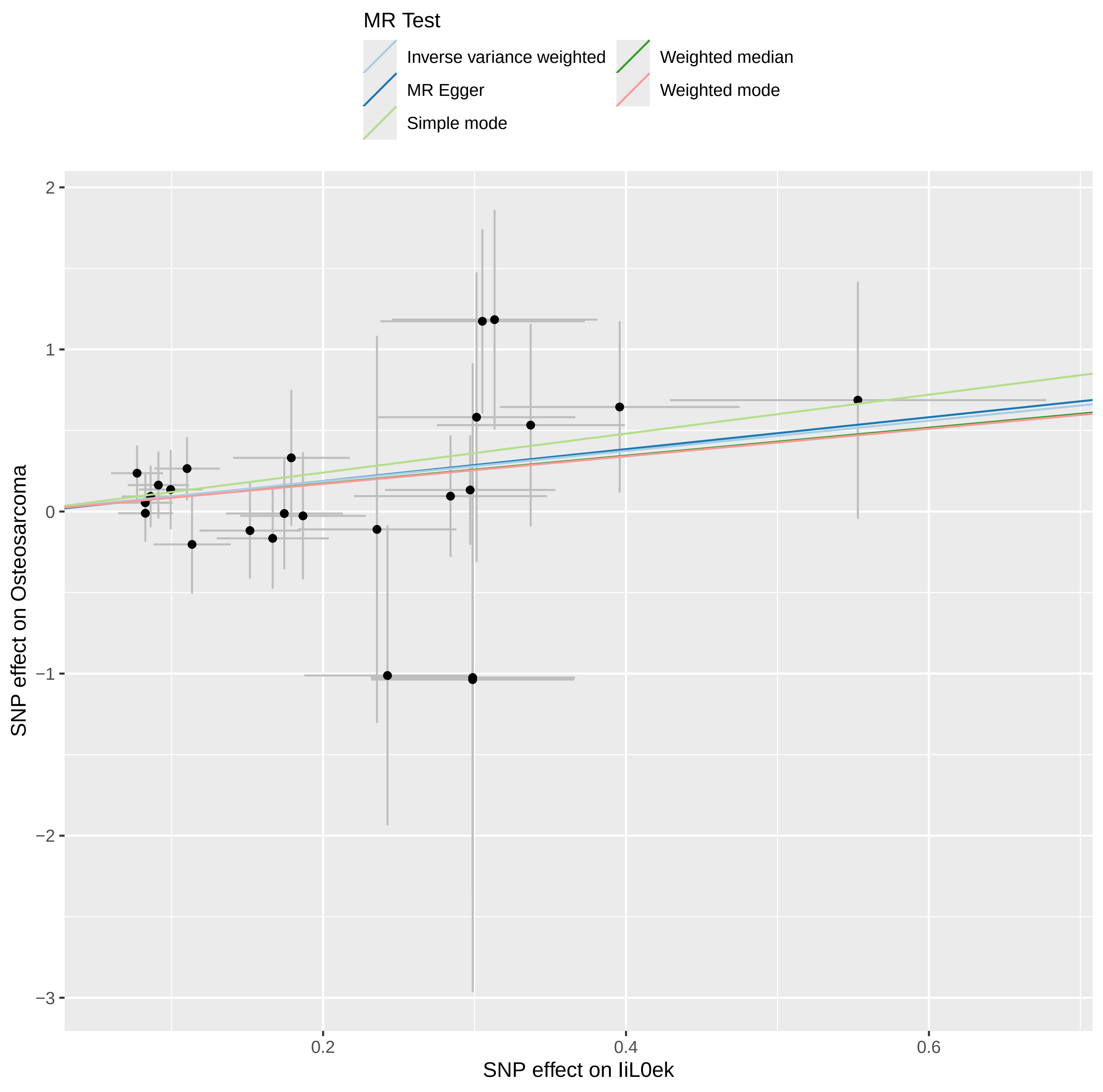

MR Test
Inverse variance weighted
MR Egger
Simple mode
Weighted median
Weighted mode
SNP effect on Osteosarcoma
SNP effect on IiL0ek
2
1
0
−1
−2
−3
0.2
0.4
0.6

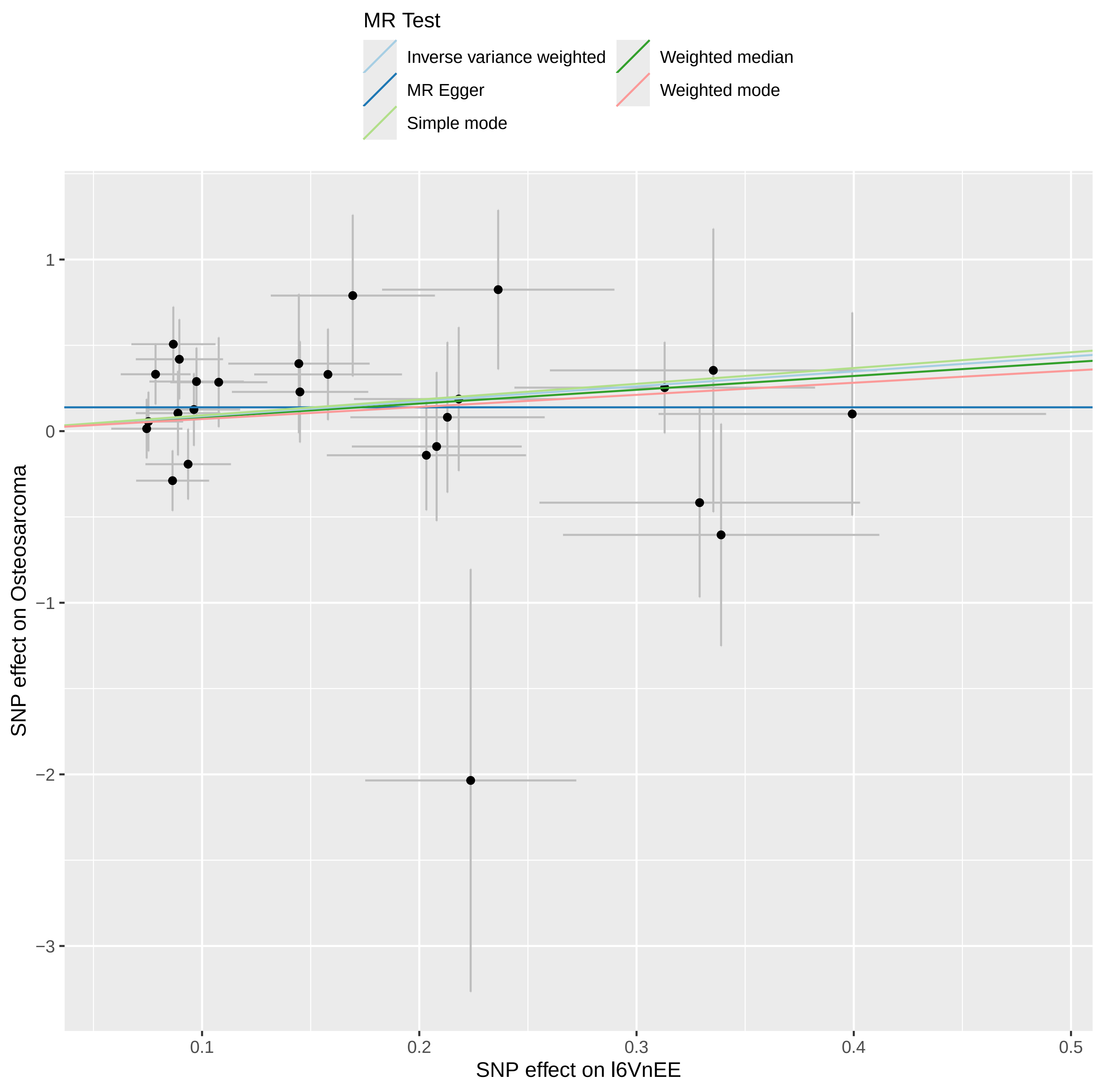
MR Test
Inverse variance weighted
MR Egger
Simple mode
Weighted median
Weighted mode
1
0
−1
−2
−3
SNP effect on Osteosarcoma
0.1
0.2
0.3
0.4
0.5
SNP effect on I6VnEE

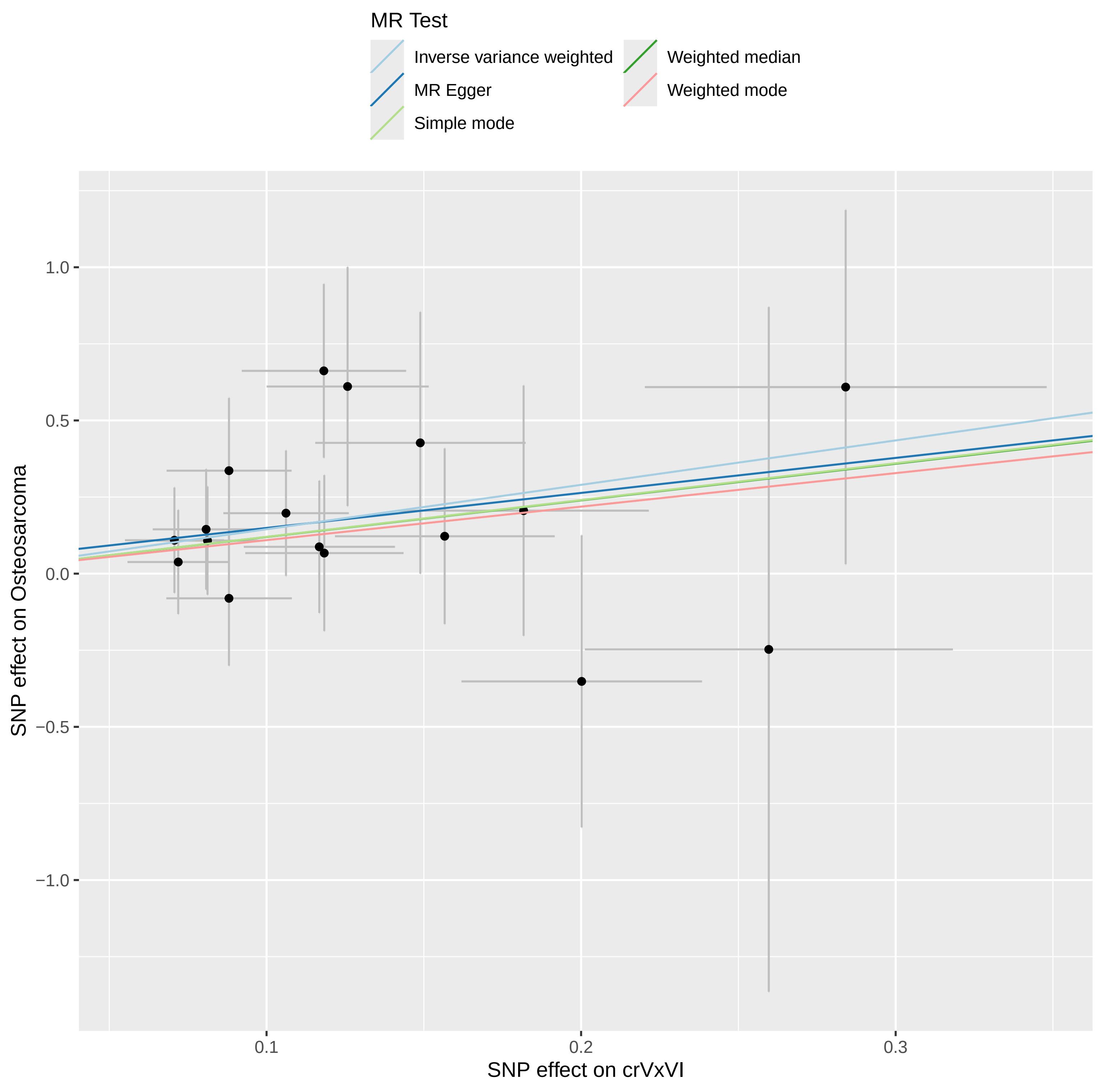

MR Test
Inverse variance weighted
MR Egger
Simple mode
Weighted median
Weighted mode
1.0
0.5
0.0
−0.5
−1.0
0.1
0.2
0.3
SNP effect on Osteosarcoma
SNP effect on crVxVI

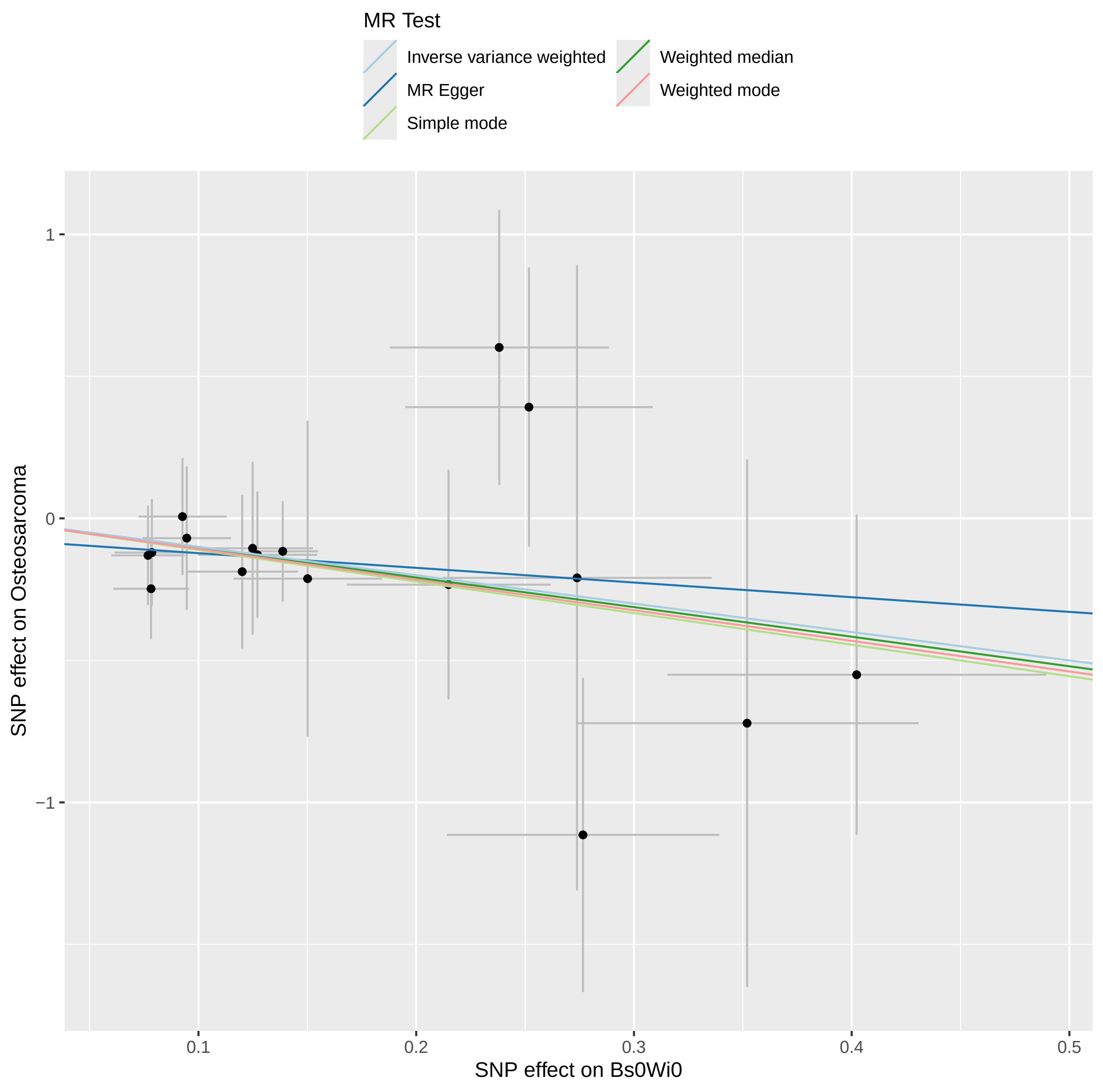

MR Test
Inverse variance weighted
MR Egger
Simple mode
Weighted median
Weighted mode
1
0
−1
0.1
0.2
0.3
0.4
0.5
SNP effect on Osteosarcoma
SNP effect on Bs0Wi0

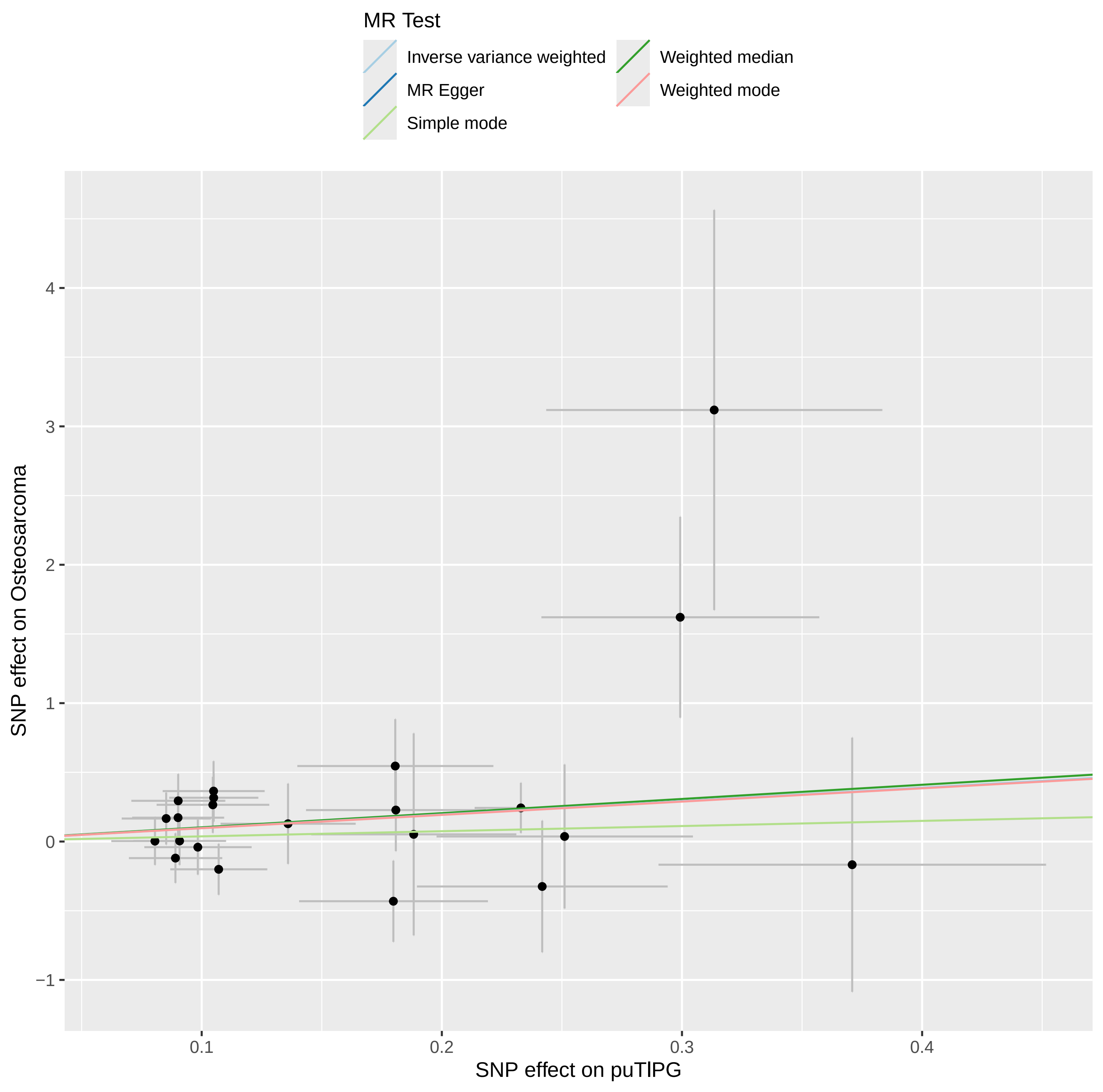

MR Test
Inverse variance weighted
MR Egger
Simple mode
Weighted median
Weighted mode
SNP effect on Osteosarcoma
4
3
2
1
0
−1
0.1
0.2
0.3
0.4
SNP effect on puTIPG

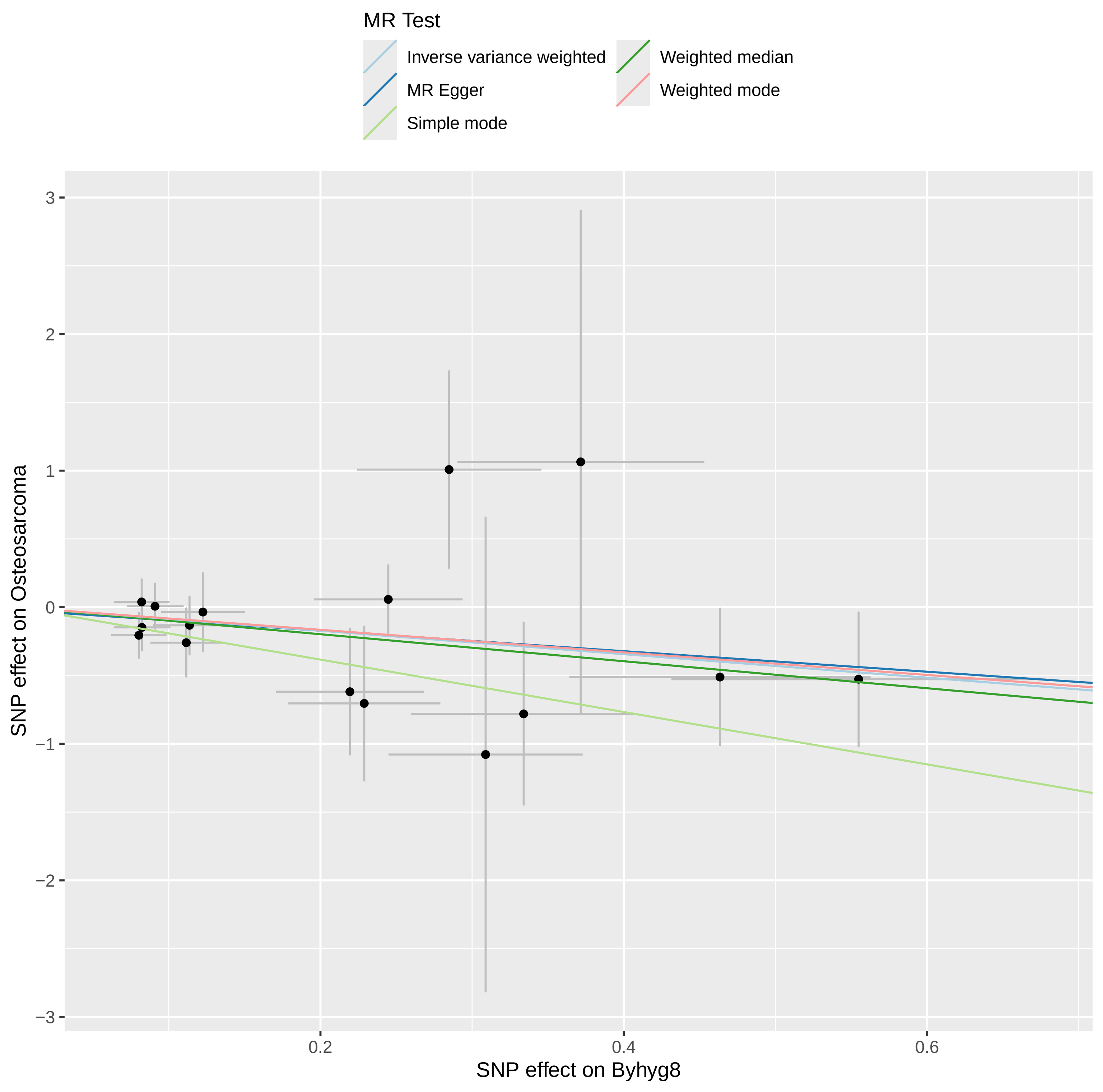

MR Test
Inverse variance weighted
MR Egger
Simple mode
Weighted median
Weighted mode
SNP effect on Osteosarcoma
3
2
1
0
−1
−2
−3
0.2
0.4
0.6
SNP effect on Byhyg8

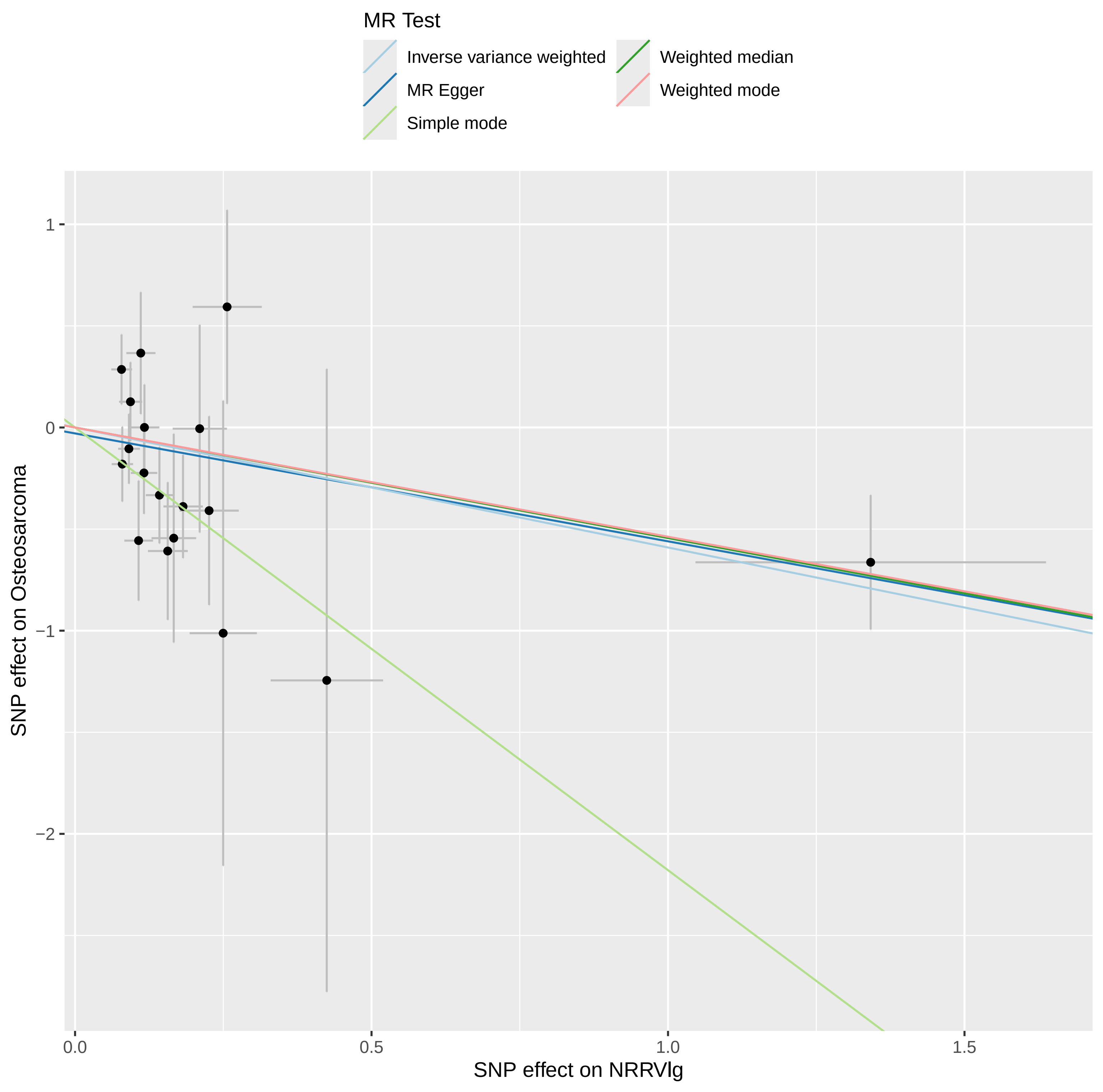

MR Test
Inverse variance weighted
MR Egger
Simple mode
Weighted median
Weighted mode
1
0
−1
−2
SNP effect on Osteosarcoma
0.0
0.5
1.0
1.5
SNP effect on NRRVlg

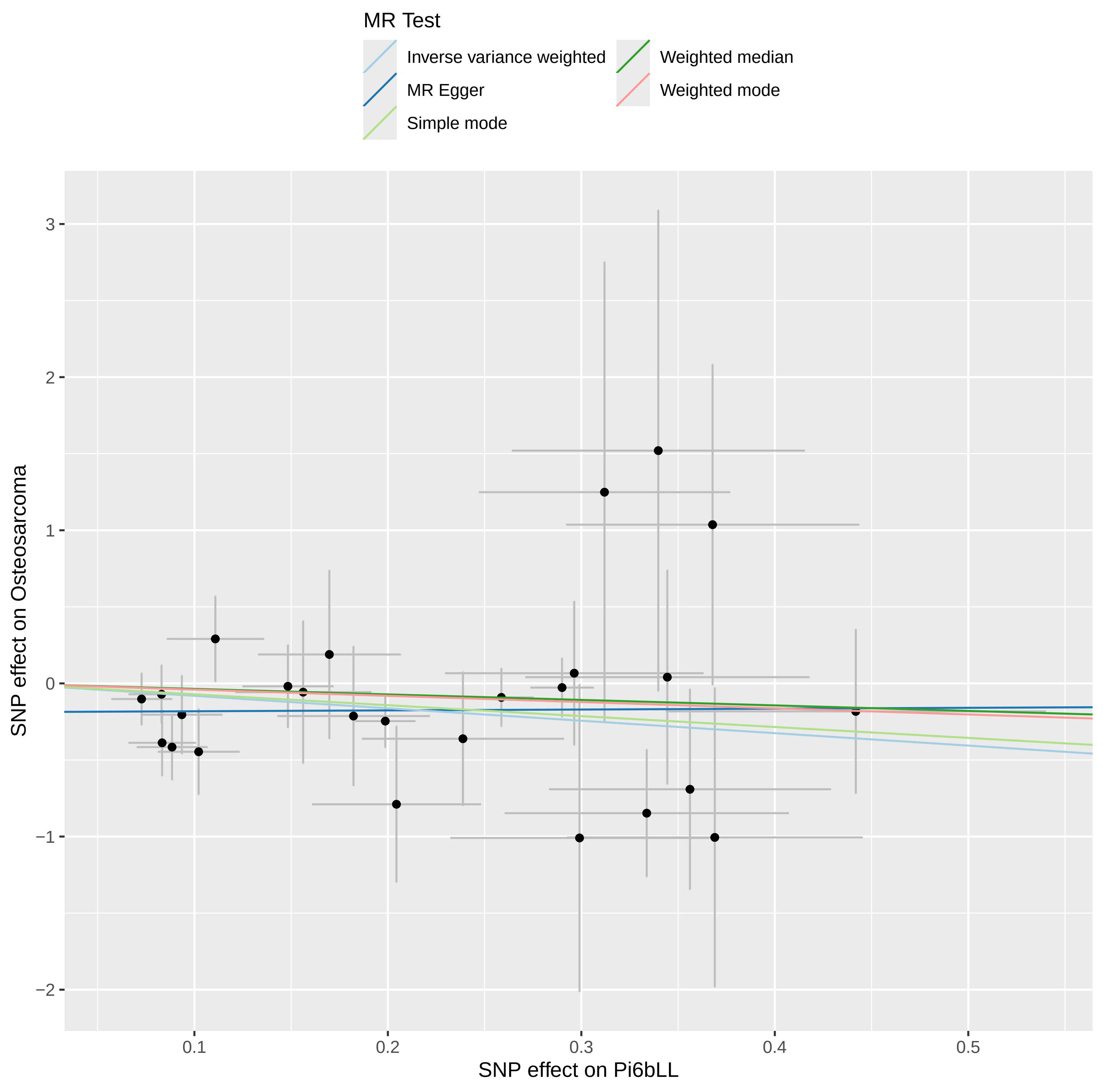

MR Test
Inverse variance weighted
MR Egger
Simple mode
Weighted median
Weighted mode
3
2
1
0
−1
−2
0.1
0.2
0.3
0.4
0.5
SNP effect on Osteosarcoma
SNP effect on Pi6bLL

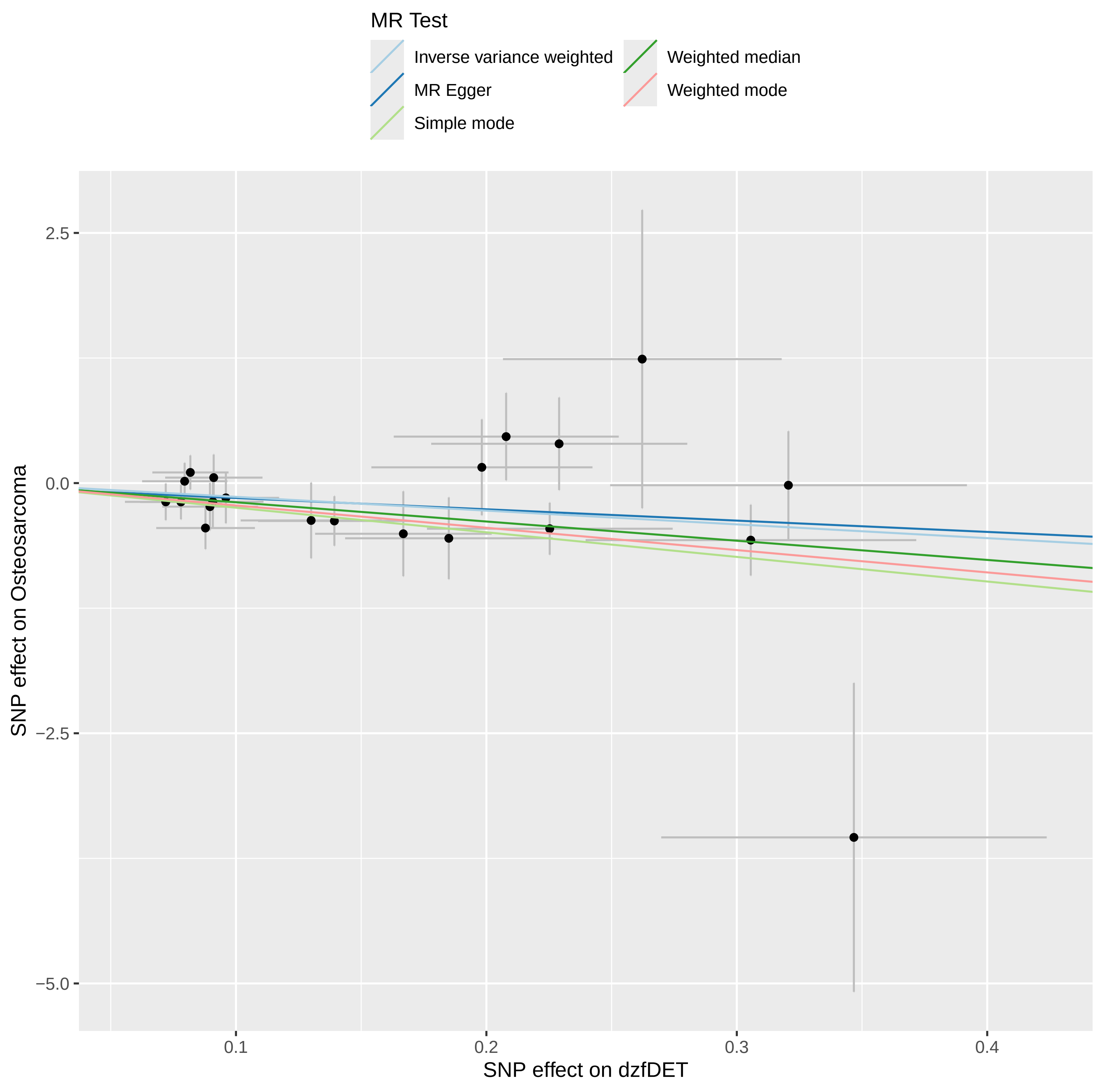
MR Test
Inverse variance weighted
MR Egger
Simple mode
Weighted median
Weighted mode
2.5
0.0
−2.5
−5.0
SNP effect on Osteosarcoma
0.1
0.2
0.3
0.4
SNP effect on dzfDET

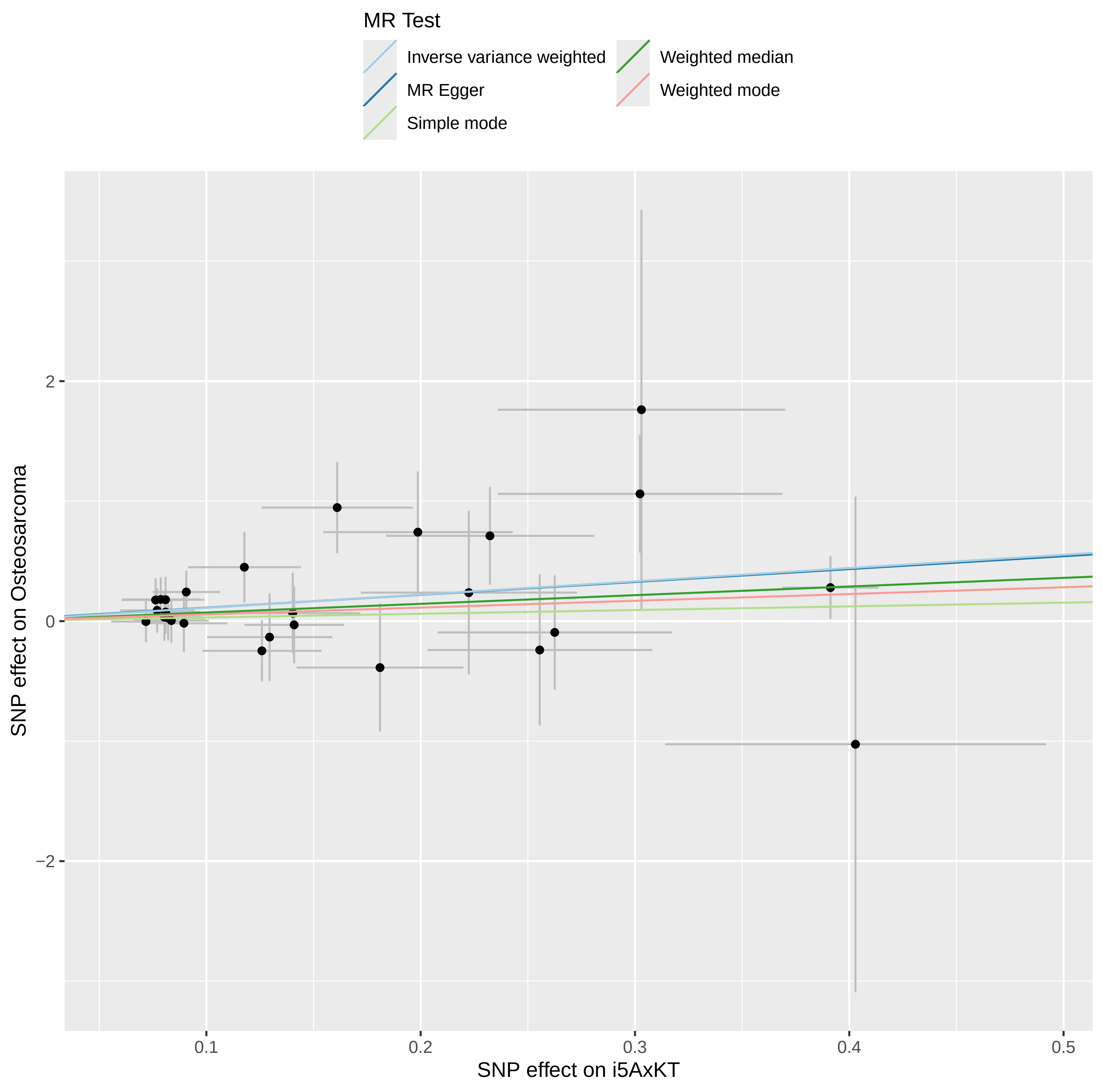

MR Test
Inverse variance weighted
MR Egger
Simple mode
Weighted median
Weighted mode
2
0
−2
0.1
0.2
0.3
0.4
0.5
SNP effect on Osteosarcoma
SNP effect on i5AxKT

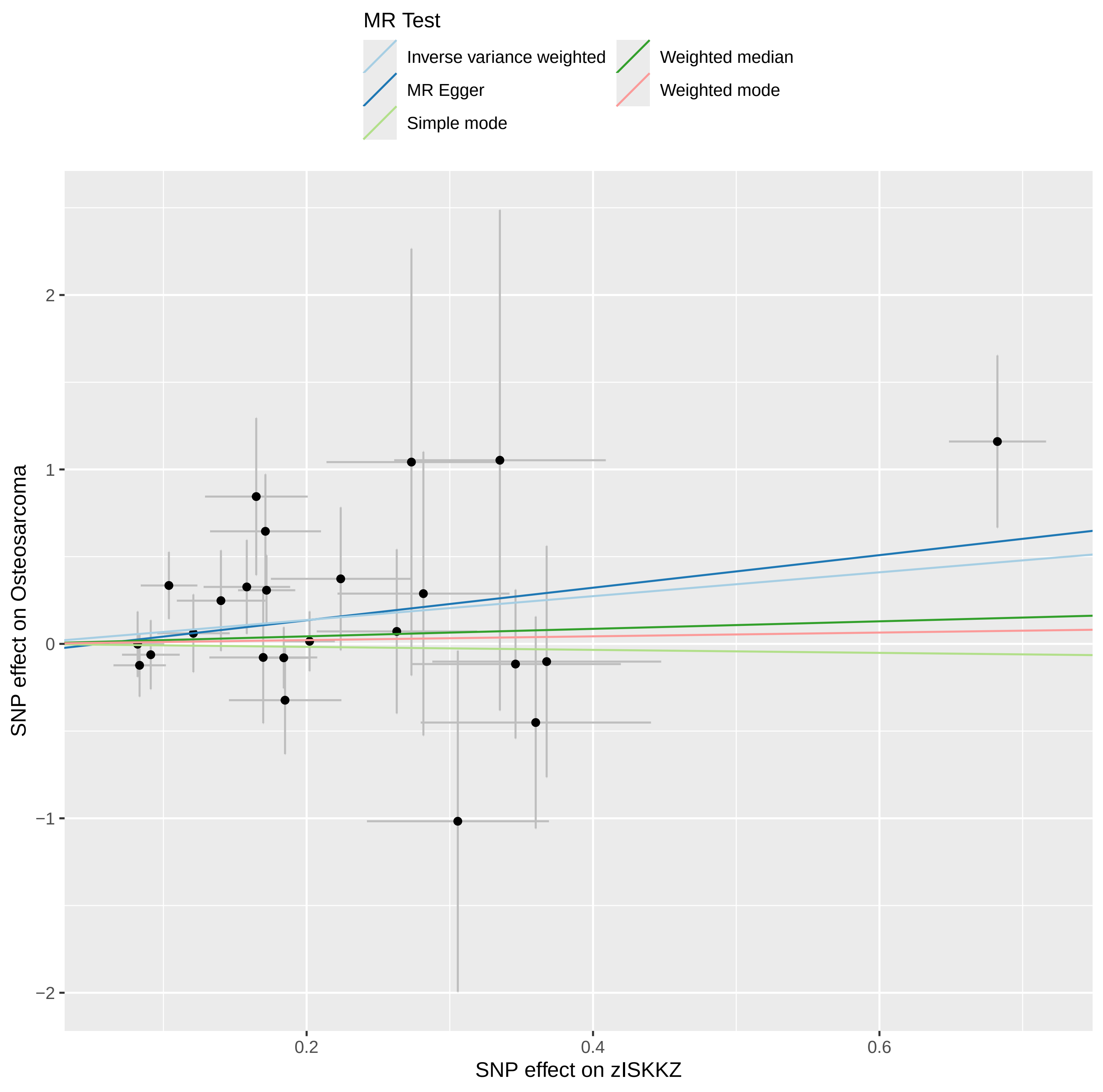

MR Test
Inverse variance weighted
MR Egger
Simple mode
Weighted median
Weighted mode
SNP effect on Osteosarcoma
SNP effect on zISKKZ
2
1
0
−1
−2
0.2
0.4
0.6

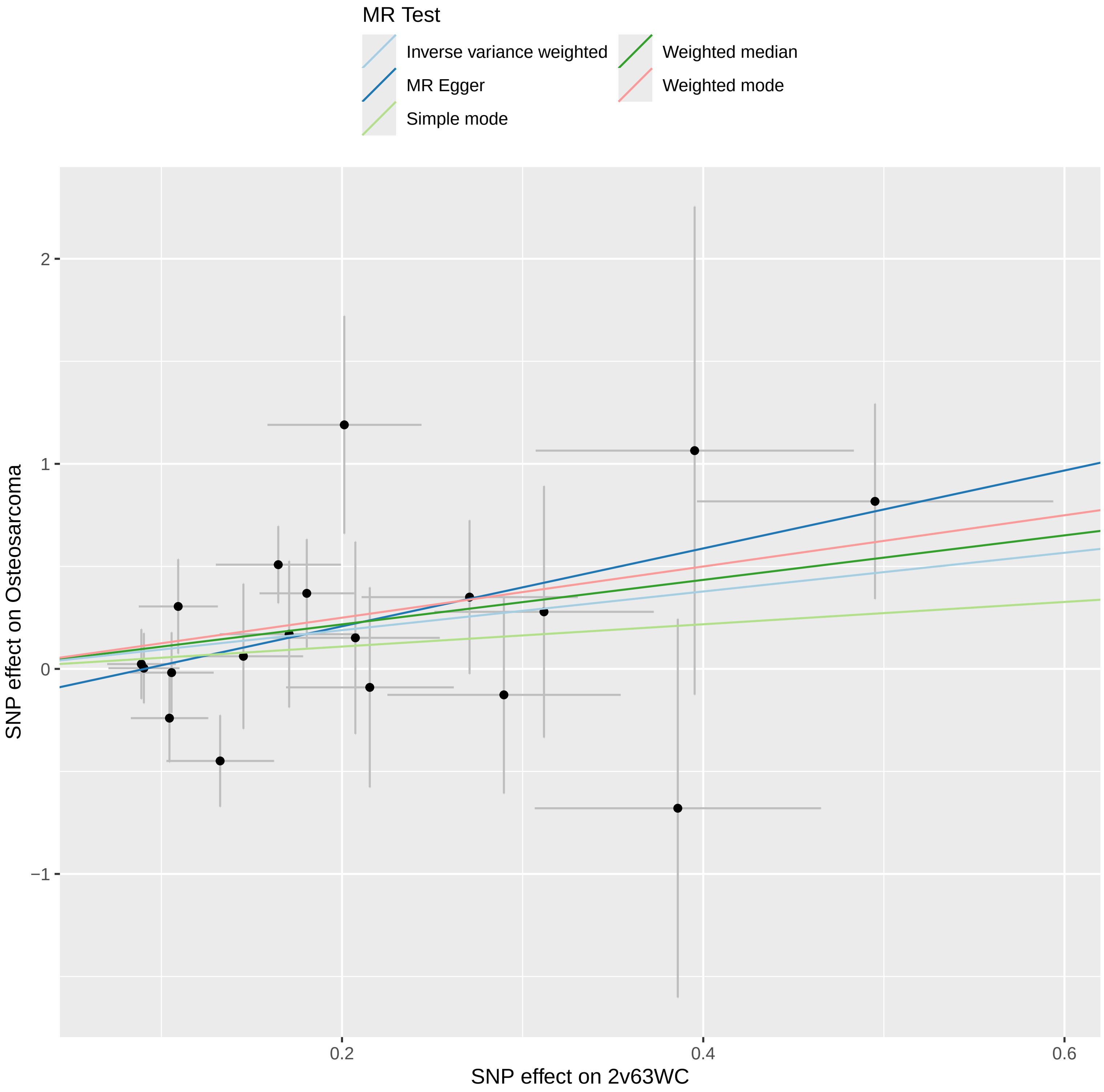

MR Test
Inverse variance weighted
MR Egger
Simple mode
Weighted median
Weighted mode
SNP effect on Osteosarcoma
2
1
0
−1
0.2
0.4
0.6
SNP effect on 2v63WC

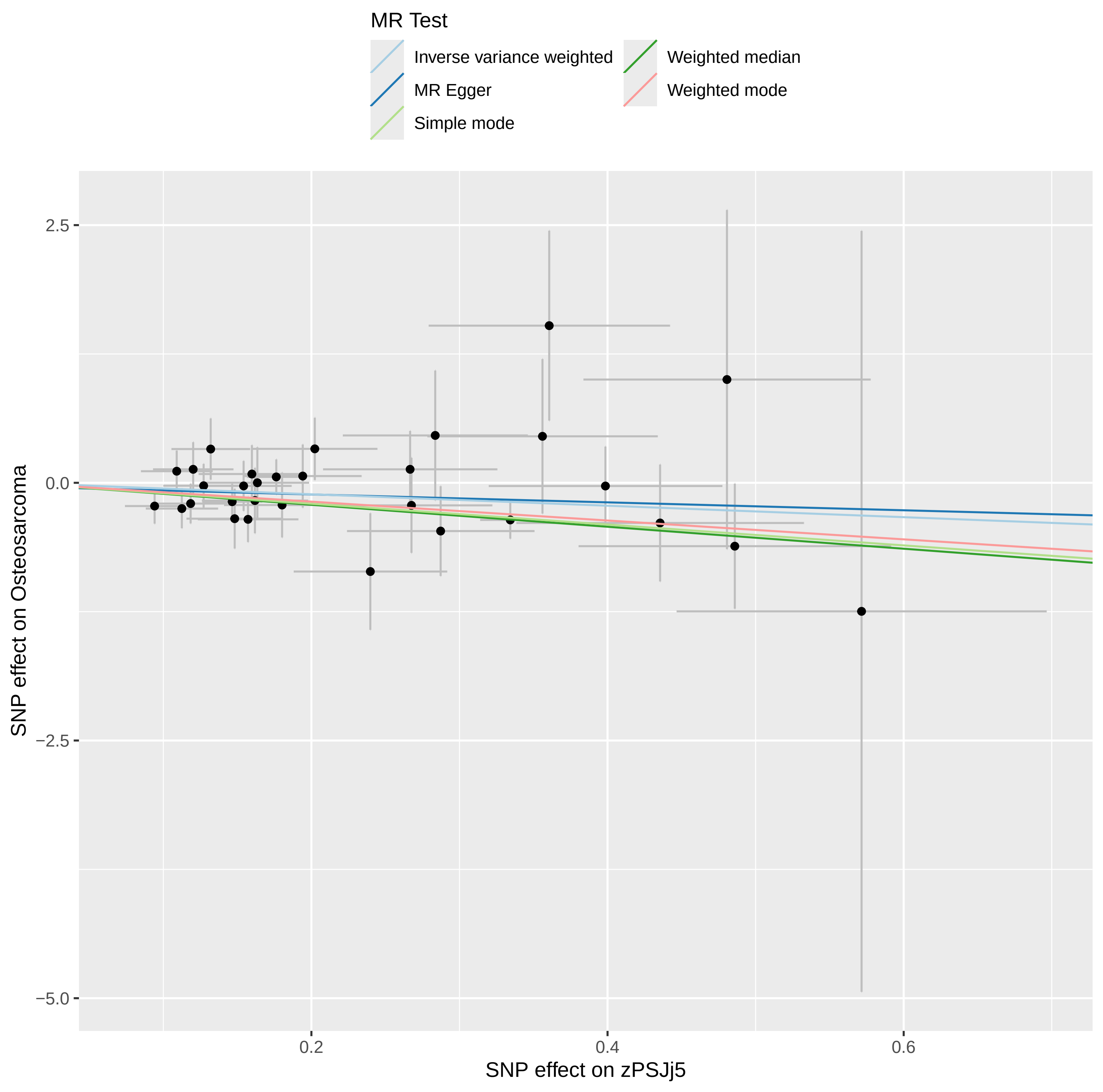

MR Test
Inverse variance weighted
MR Egger
Simple mode
Weighted median
Weighted mode
2.5
0.0
−2.5
−5.0
0.2
0.4
0.6
SNP effect on zPSJj5
SNP effect on Osteosarcoma

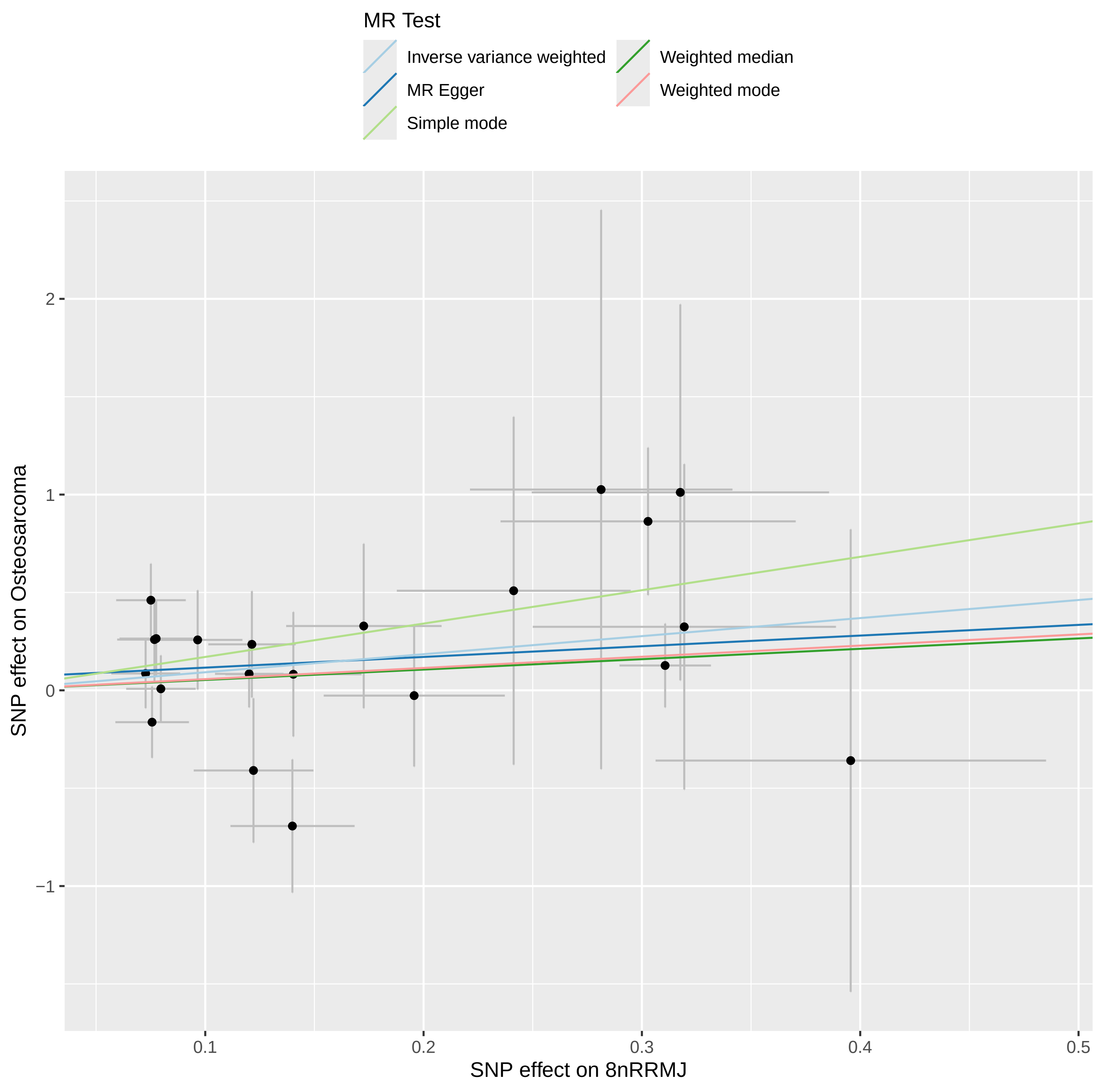
MR Test
Inverse variance weighted
MR Egger
Simple mode
Weighted median
Weighted mode
SNP effect on Osteosarcoma
2
1
0
−1
0.1
0.2
0.3
0.4
0.5
SNP effect on 8nRRMJ

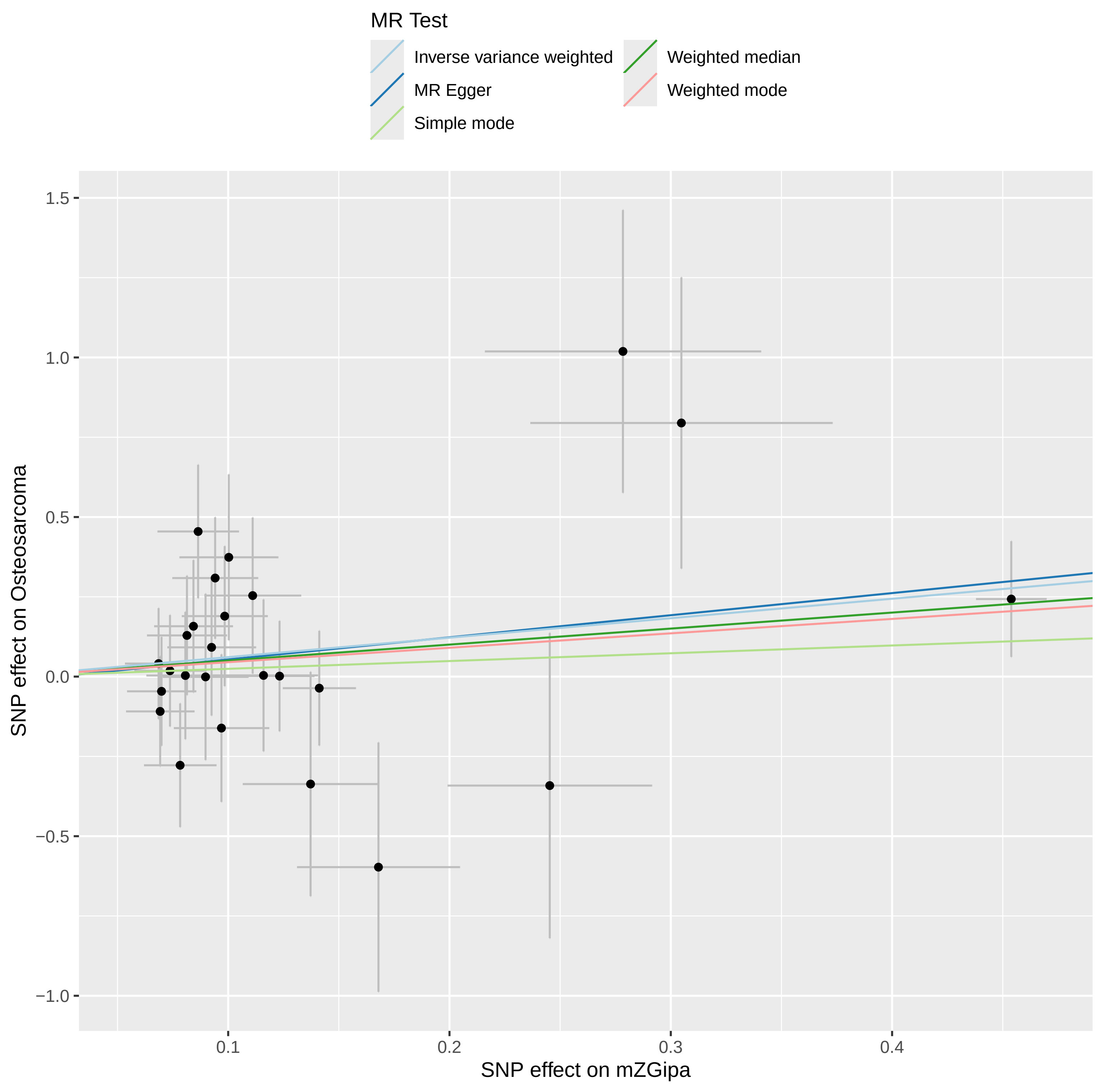

MR Test
Inverse variance weighted
MR Egger
Simple mode
Weighted median
Weighted mode
1.5
1.0
0.5
0.0
−0.5
−1.0
SNP effect on Osteosarcoma
0.1
0.2
0.3
0.4
SNP effect on mZGipa

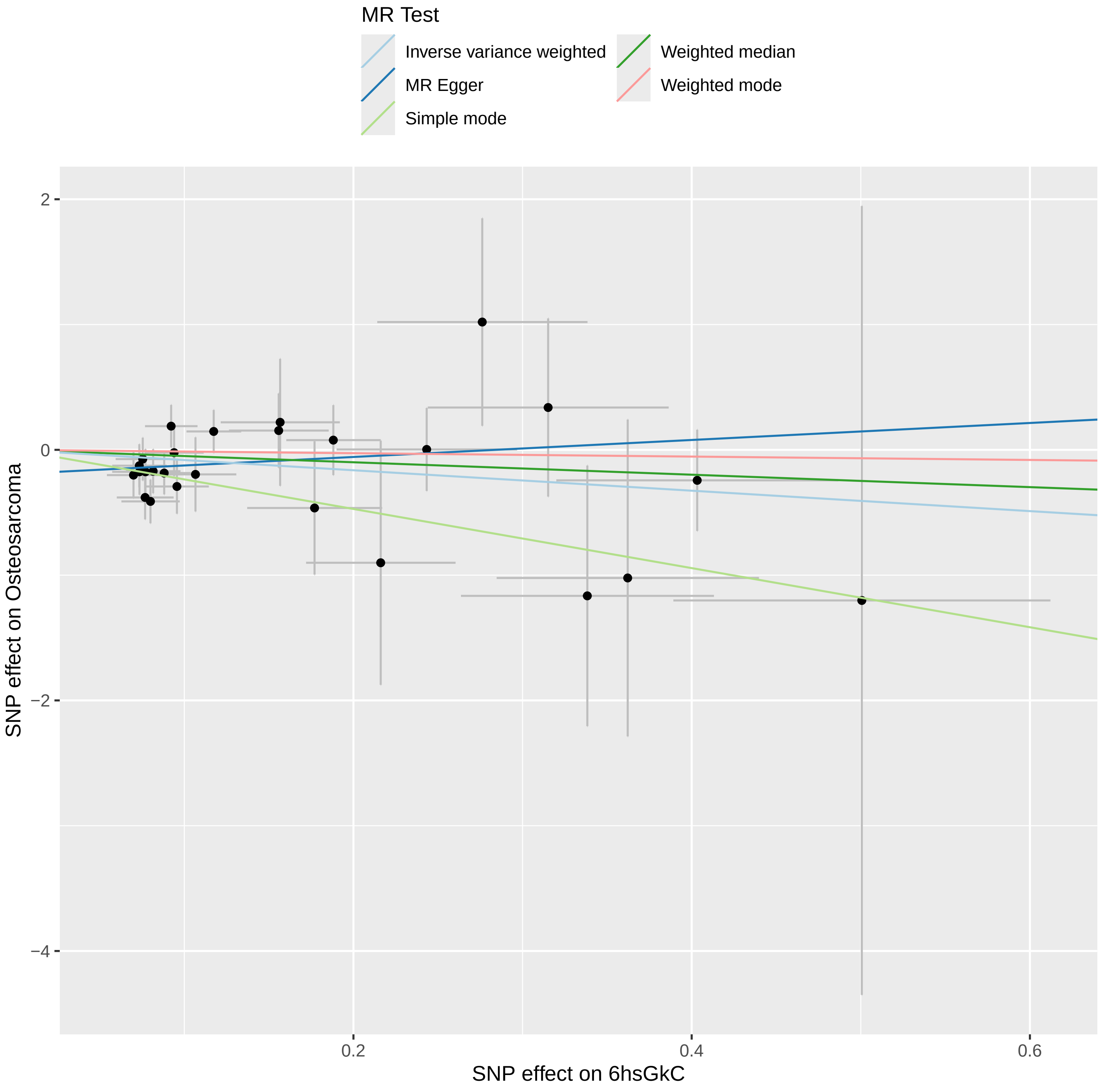

MR Test
Inverse variance weighted
MR Egger
Simple mode
Weighted median
Weighted mode
SNP effect on Osteosarcoma
2
0
−2
−4
0.2
0.4
0.6
SNP effect on 6hsGkC

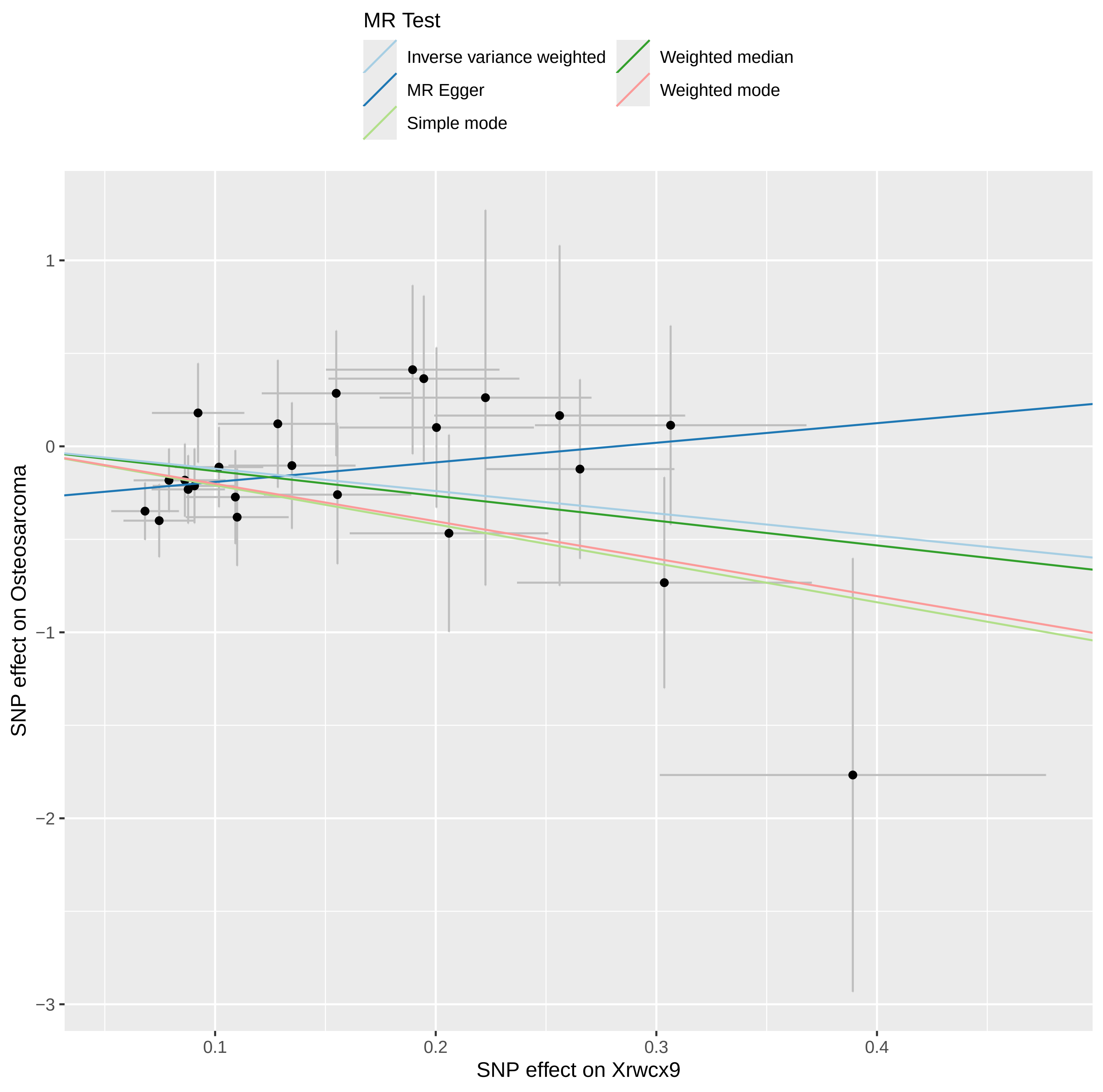

MR Test
Inverse variance weighted
MR Egger
Simple mode
Weighted median
Weighted mode
SNP effect on Osteosarcoma
1
0
−1
−2
−3
0.1
0.2
0.3
0.4
SNP effect on Xrwcx9

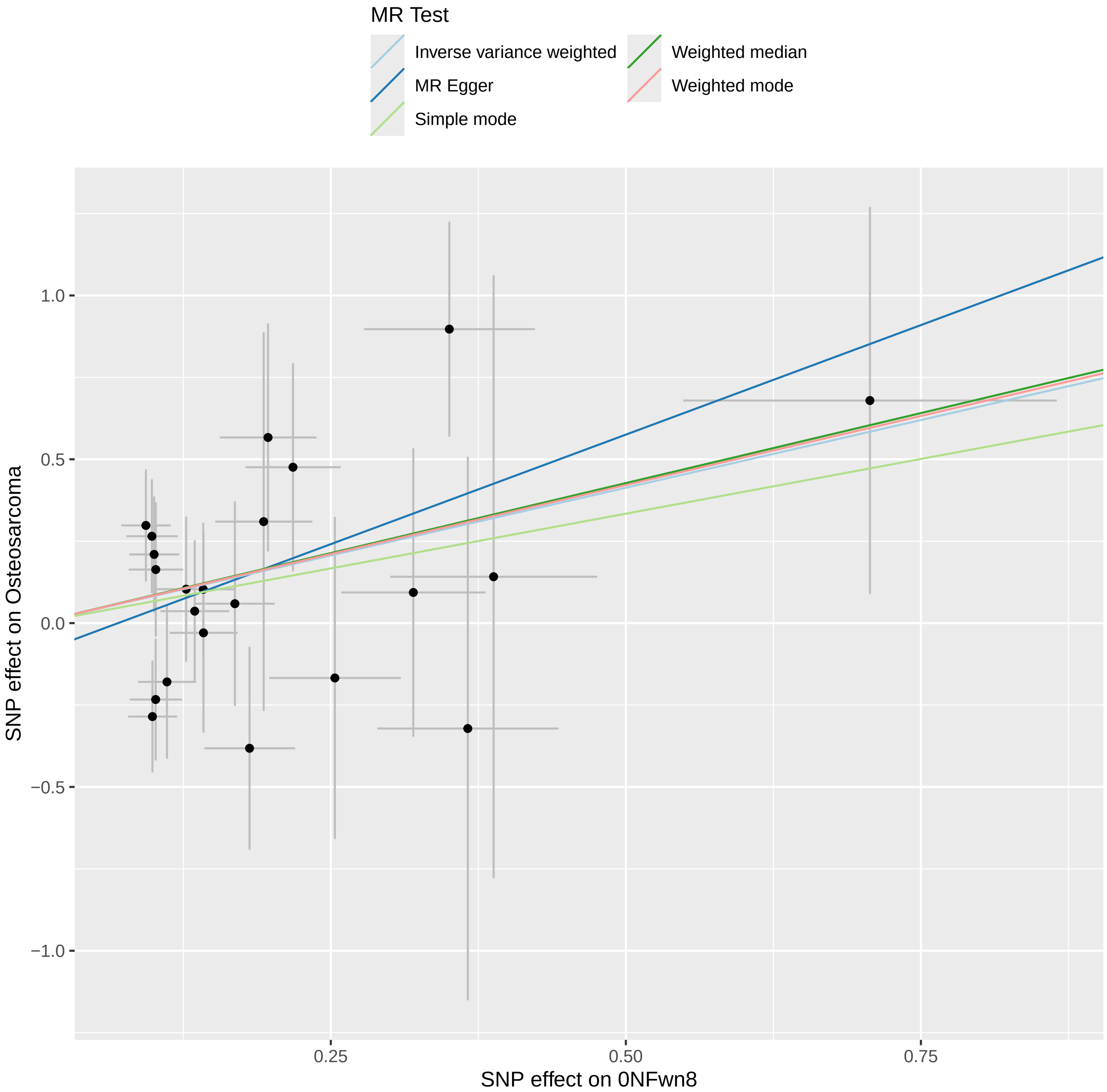

MR Test
Inverse variance weighted
MR Egger
Simple mode
Weighted median
Weighted mode
1.0
0.5
0.0
−0.5
−1.0
SNP effect on Osteosarcoma
0.25
0.50
0.75
SNP effect on 0NFwn8

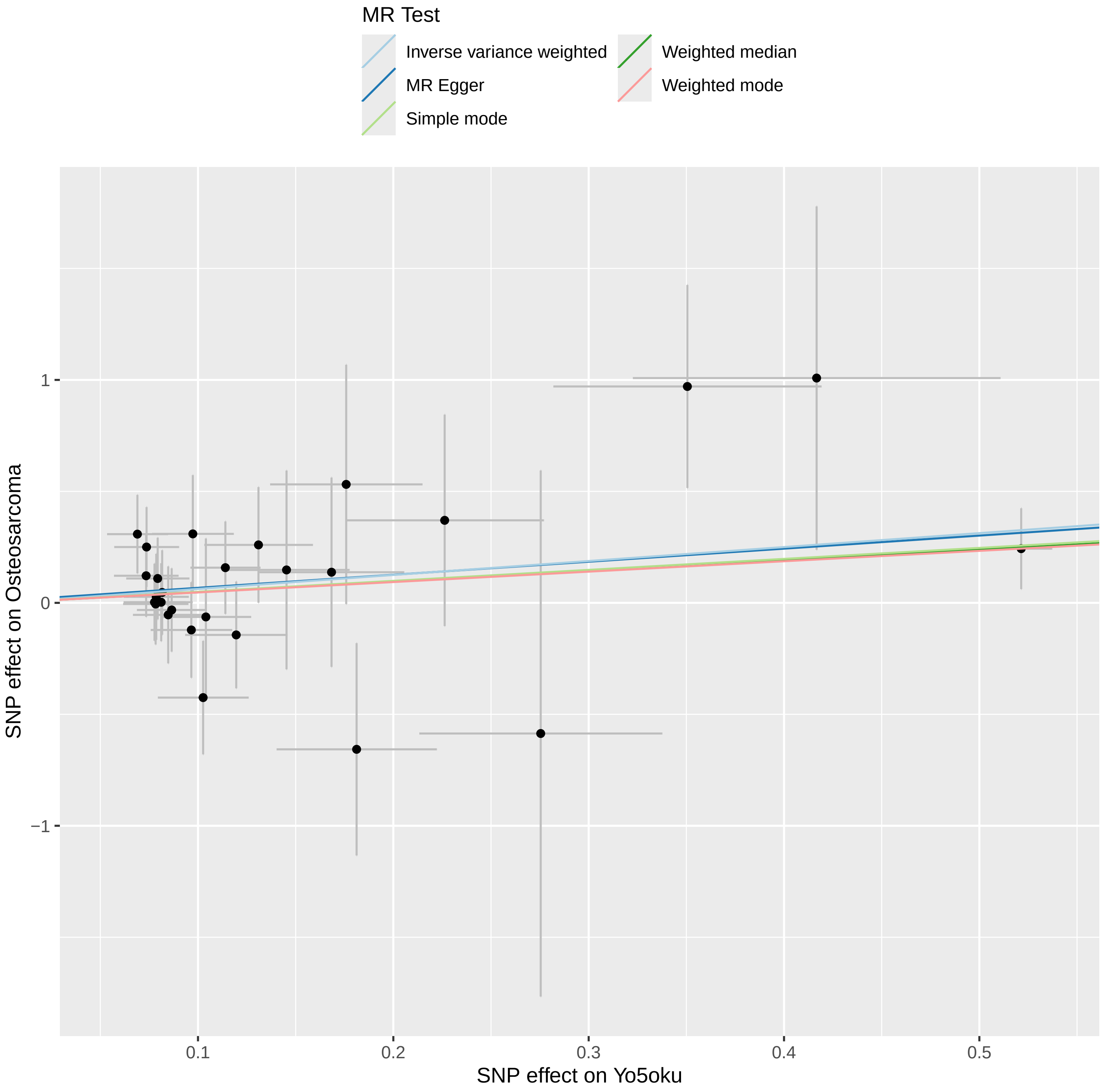

MR Test
Inverse variance weighted
MR Egger
Simple mode
Weighted median
Weighted mode
1
0
−1
0.1
0.2
0.3
0.4
0.5
SNP effect on Osteosarcoma
SNP effect on Yo5oku

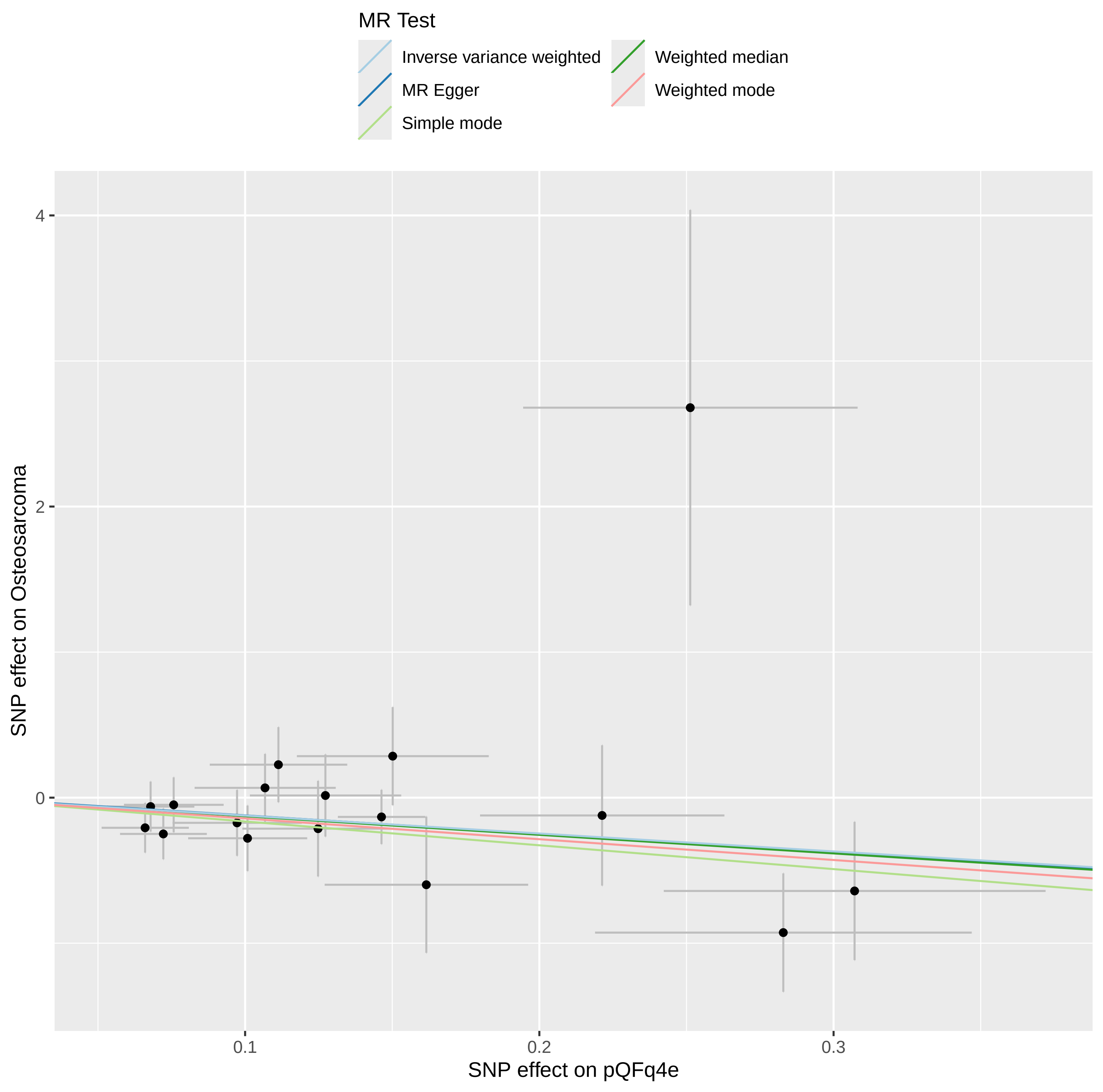

MR Test
Inverse variance weighted
MR Egger
Simple mode
Weighted median
Weighted mode
SNP effect on Osteosarcoma
4
2
0
0.1
0.2
0.3
SNP effect on pQFq4e

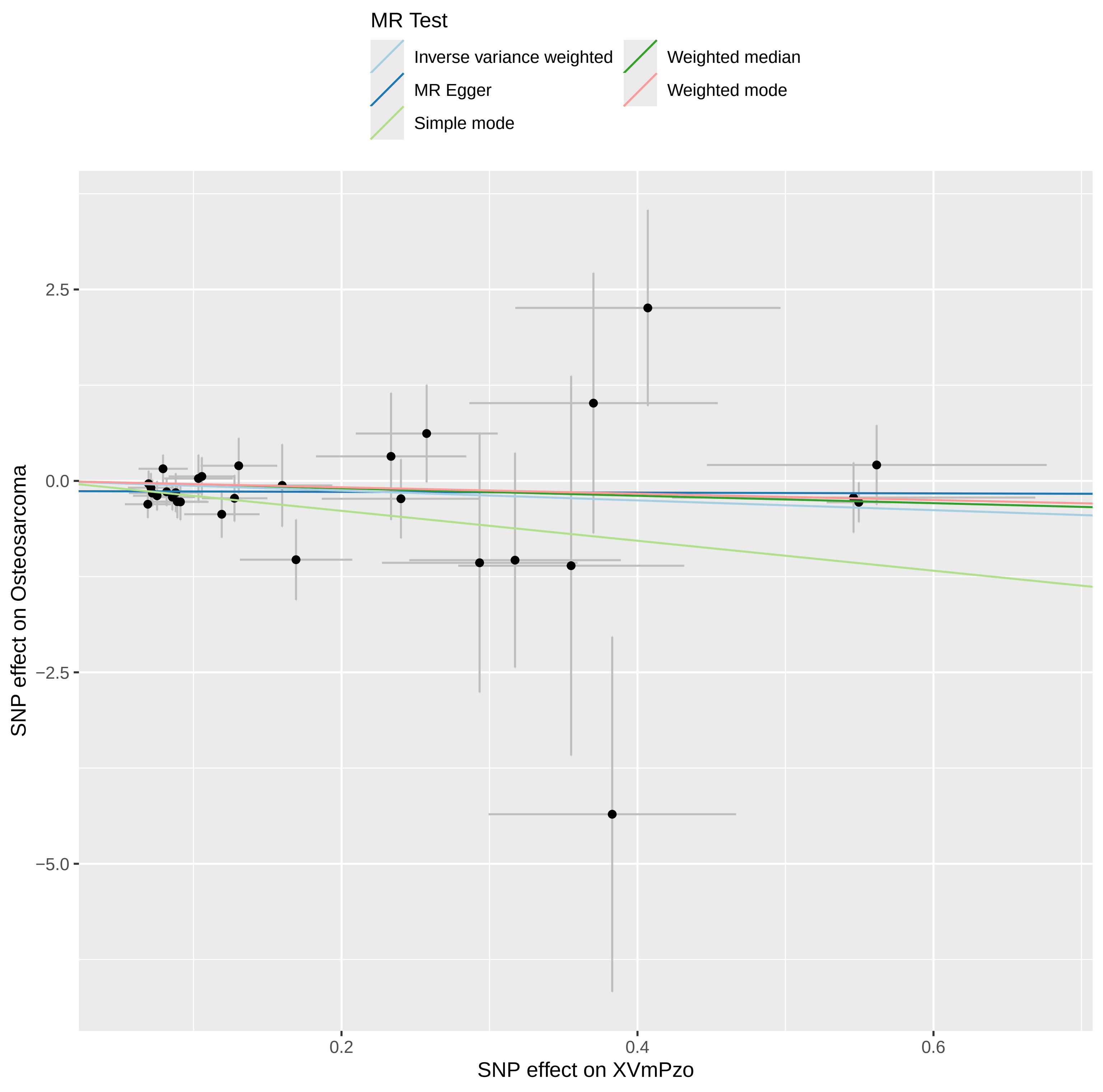
MR Test
Inverse variance weighted
MR Egger
Simple mode
Weighted median
Weighted mode
2.5
0.0
−2.5
−5.0
SNP effect on Osteosarcoma
0.2
0.4
0.6
SNP effect on XVmPzo

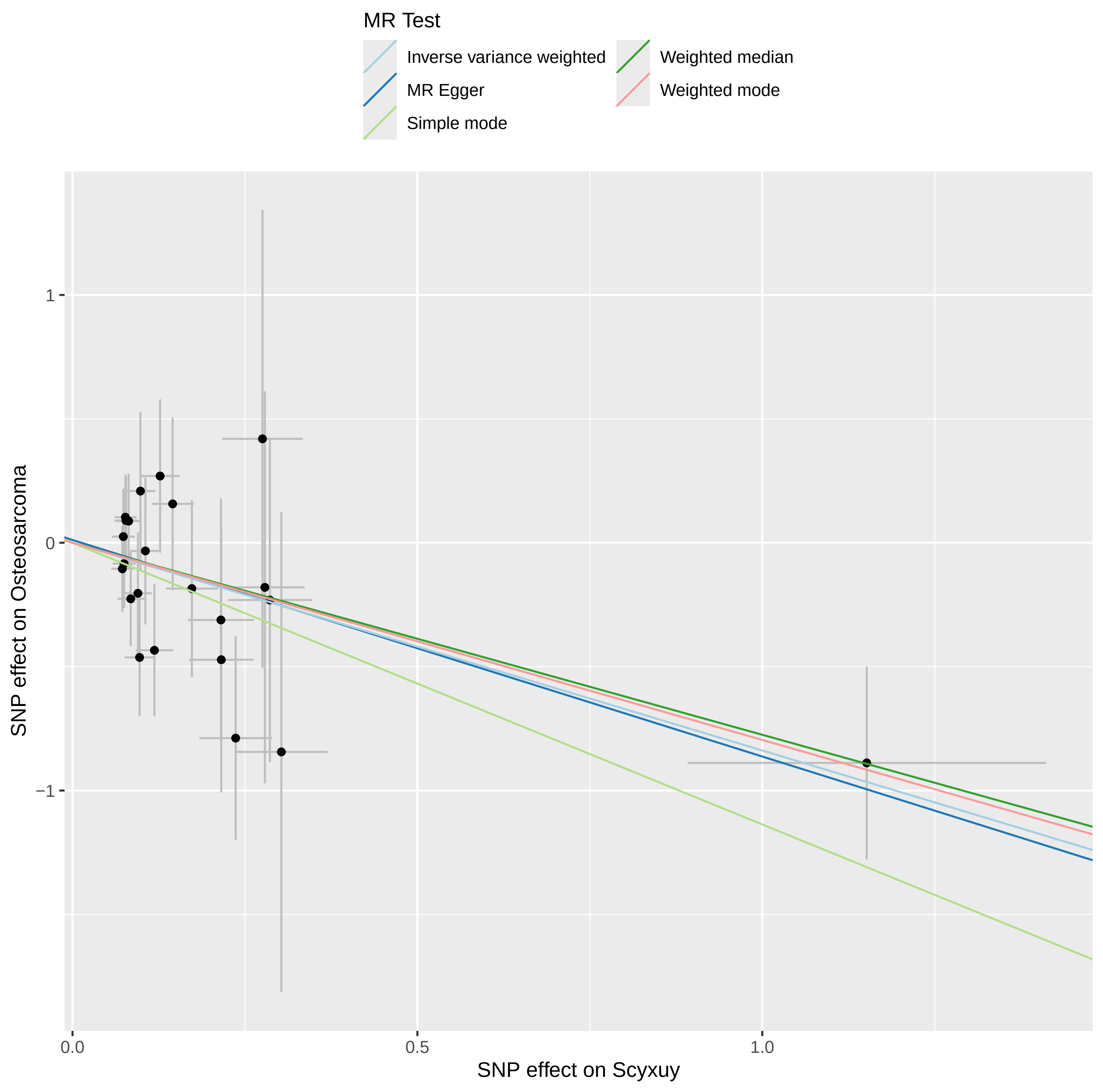
MR Test
Inverse variance weighted
MR Egger
Simple mode
Weighted median
Weighted mode
SNP effect on Osteosarcoma
1
0
−1
0.0
0.5
1.0
SNP effect on Scyxuy

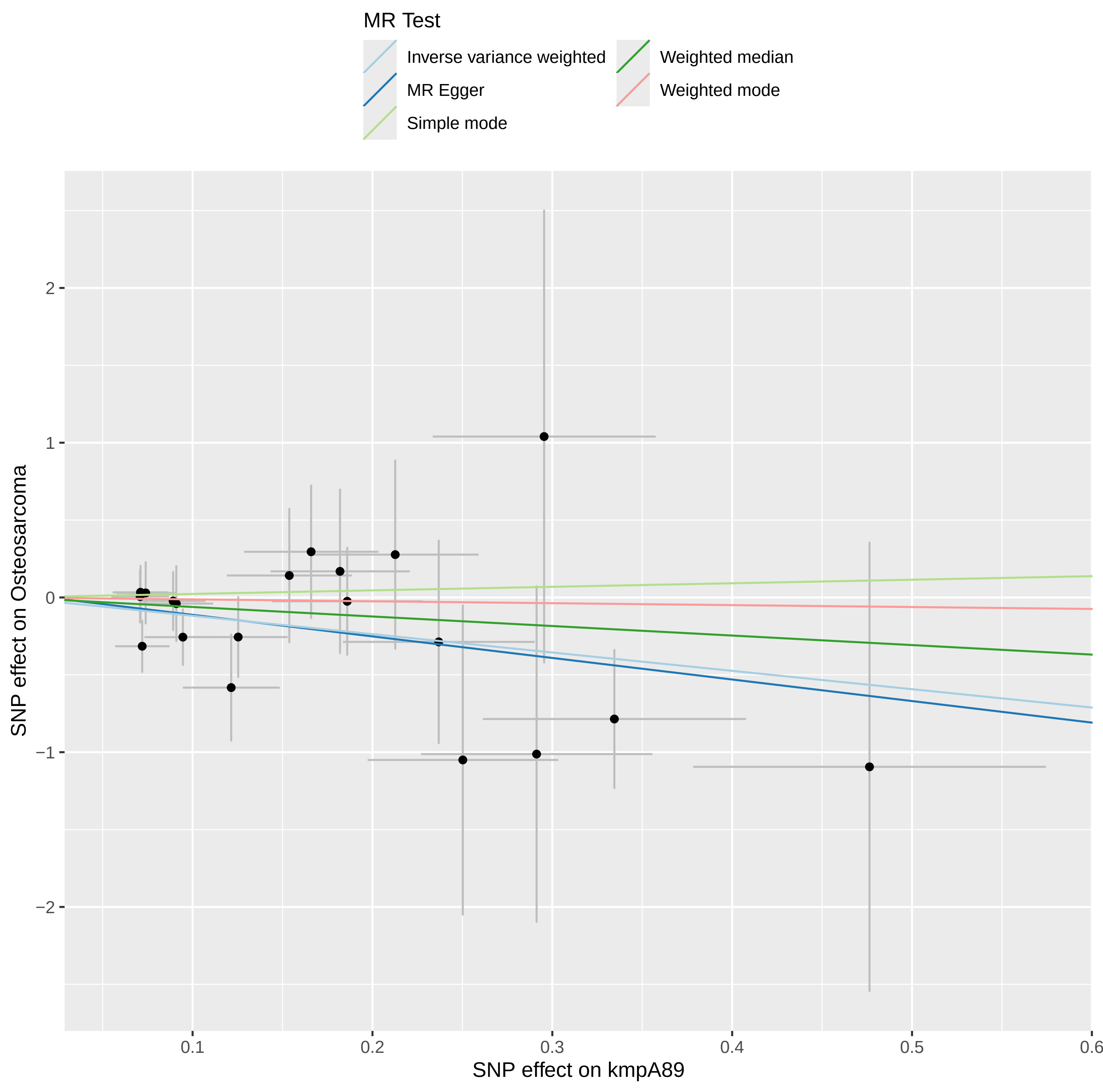

MR Test
Inverse variance weighted
MR Egger
Simple mode
Weighted median
Weighted mode
2
1
0
−1
−2
SNP effect on Osteosarcoma
0.1
0.2
0.3
0.4
0.5
0.6
SNP effect on kmpA89

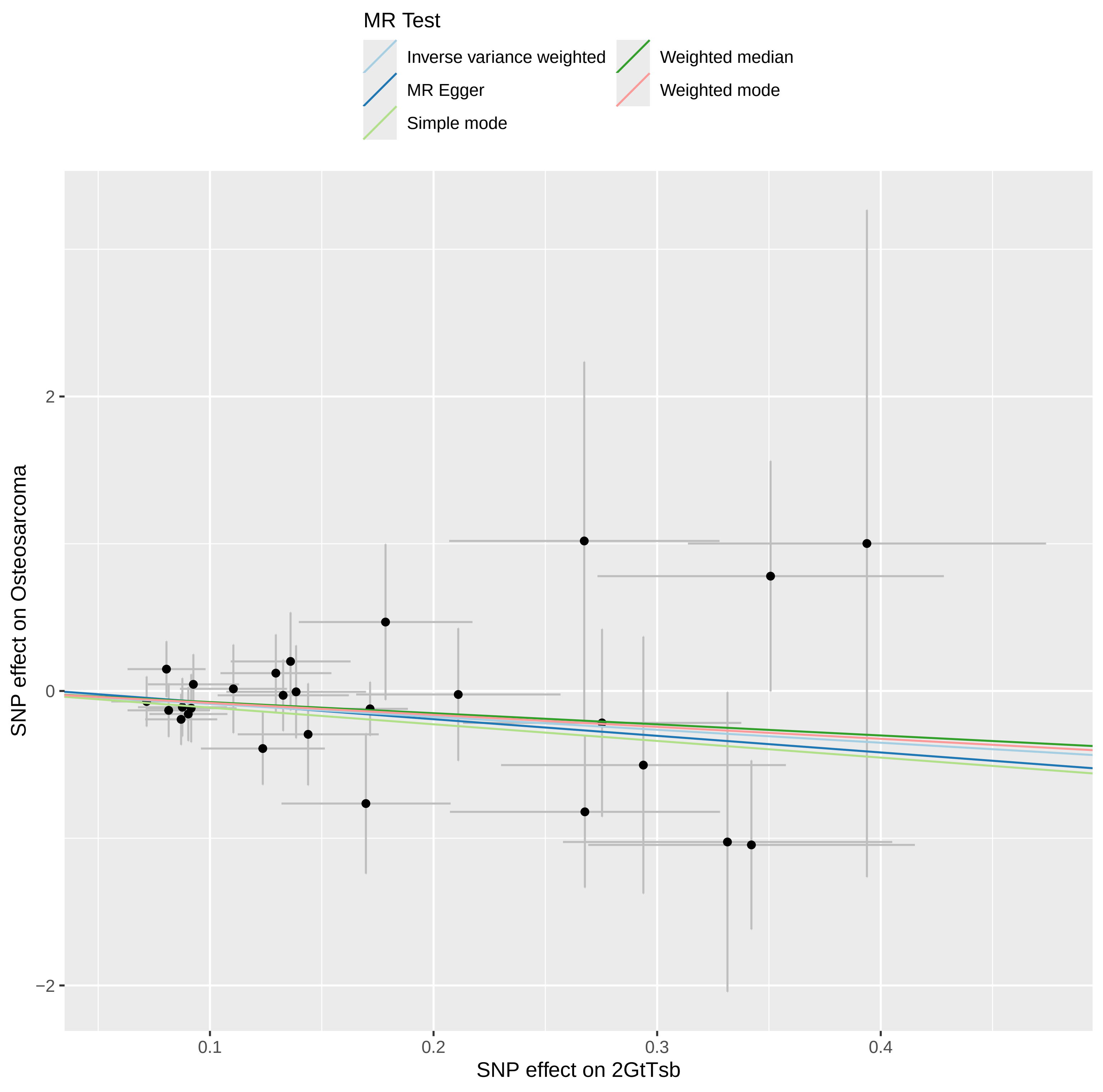

MR Test
Inverse variance weighted
MR Egger
Simple mode
Weighted median
Weighted mode
2
0
−2
0.1
0.2
0.3
0.4
SNP effect on 2GtTsb
SNP effect on Osteosarcoma

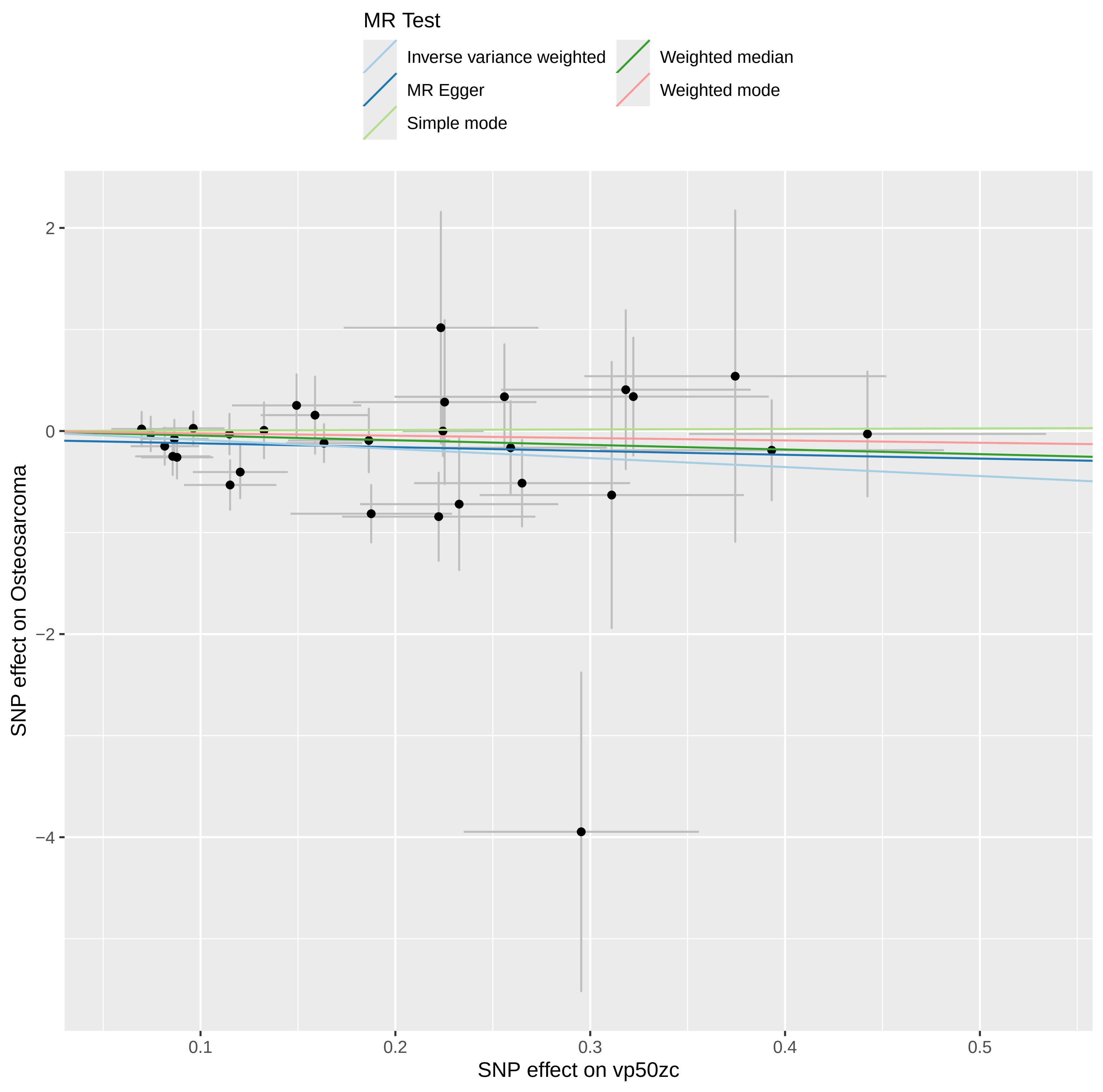

MR Test
Inverse variance weighted
MR Egger
Simple mode
Weighted median
Weighted mode
2
0
−2
−4
0.1
0.2
0.3
0.4
0.5
SNP effect on Osteosarcoma
SNP effect on vp50zc

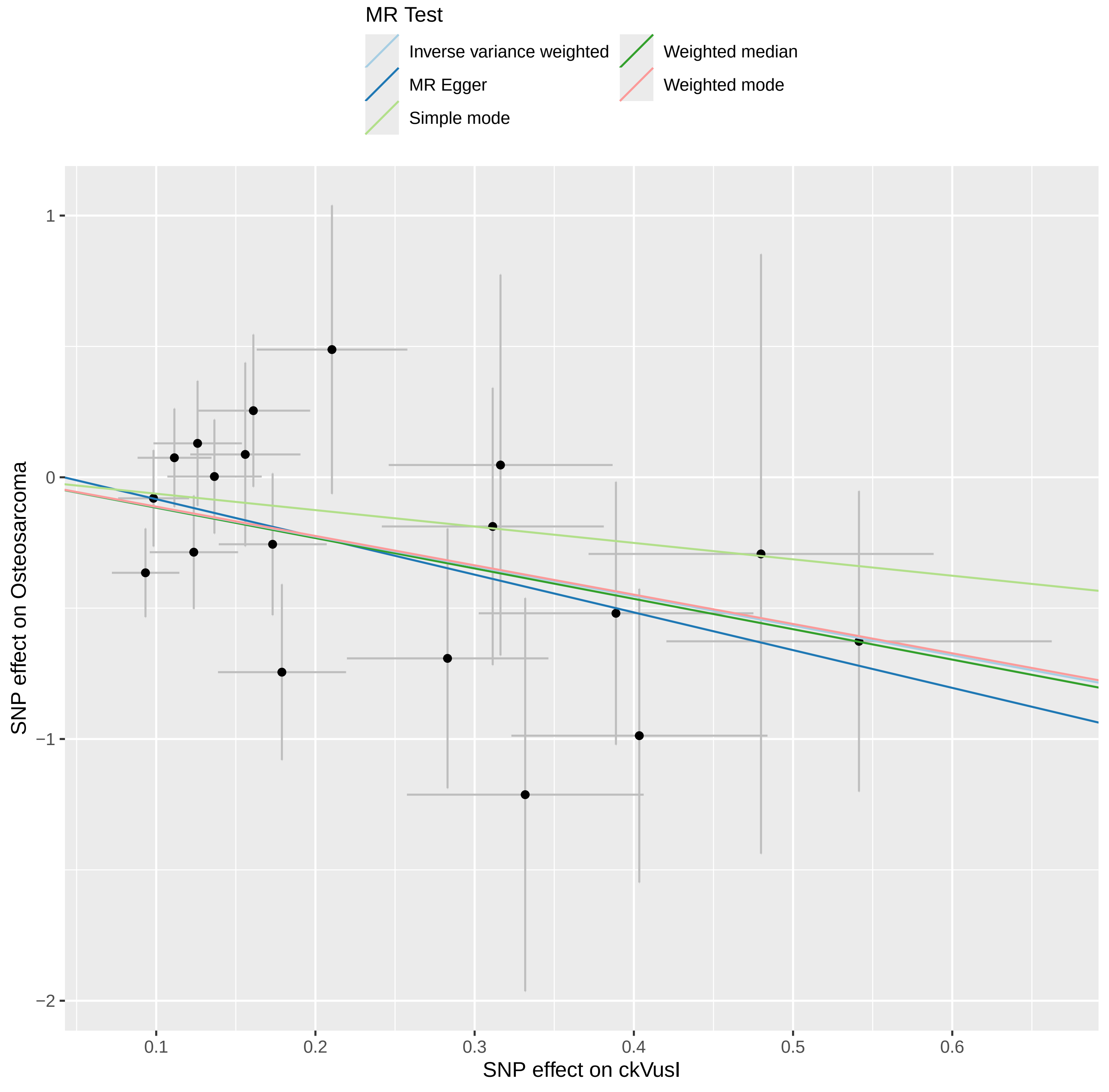

MR Test
Inverse variance weighted
MR Egger
Simple mode
Weighted median
Weighted mode
1
0
−1
−2
0.1
0.2
0.3
0.4
0.5
0.6
SNP effect on Osteosarcoma
SNP effect on ckVusI

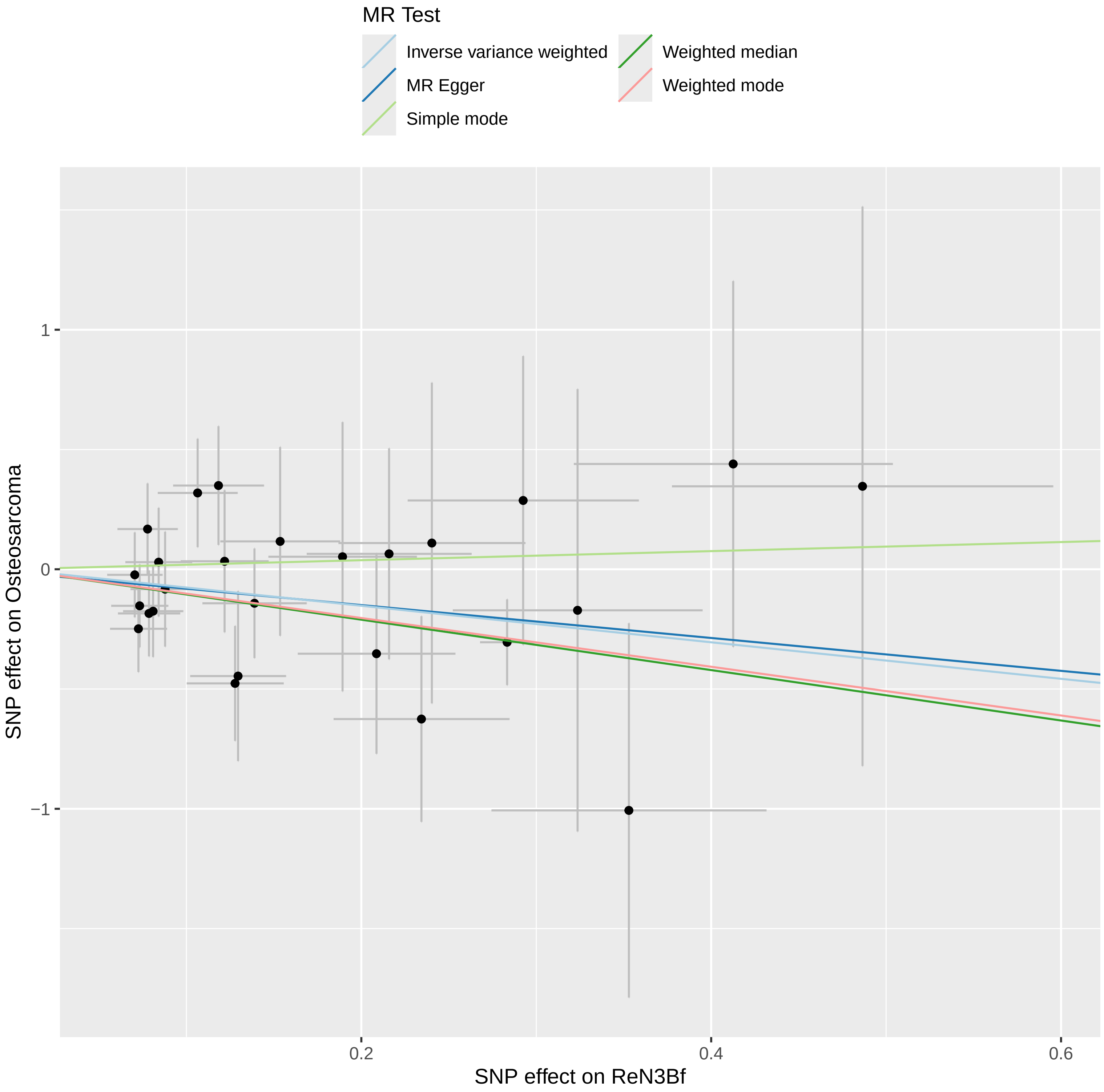

MR Test
Inverse variance weighted
MR Egger
Simple mode
Weighted median
Weighted mode
1
0
−1
0.2
0.4
0.6
SNP effect on Osteosarcoma
SNP effect on ReN3Bf

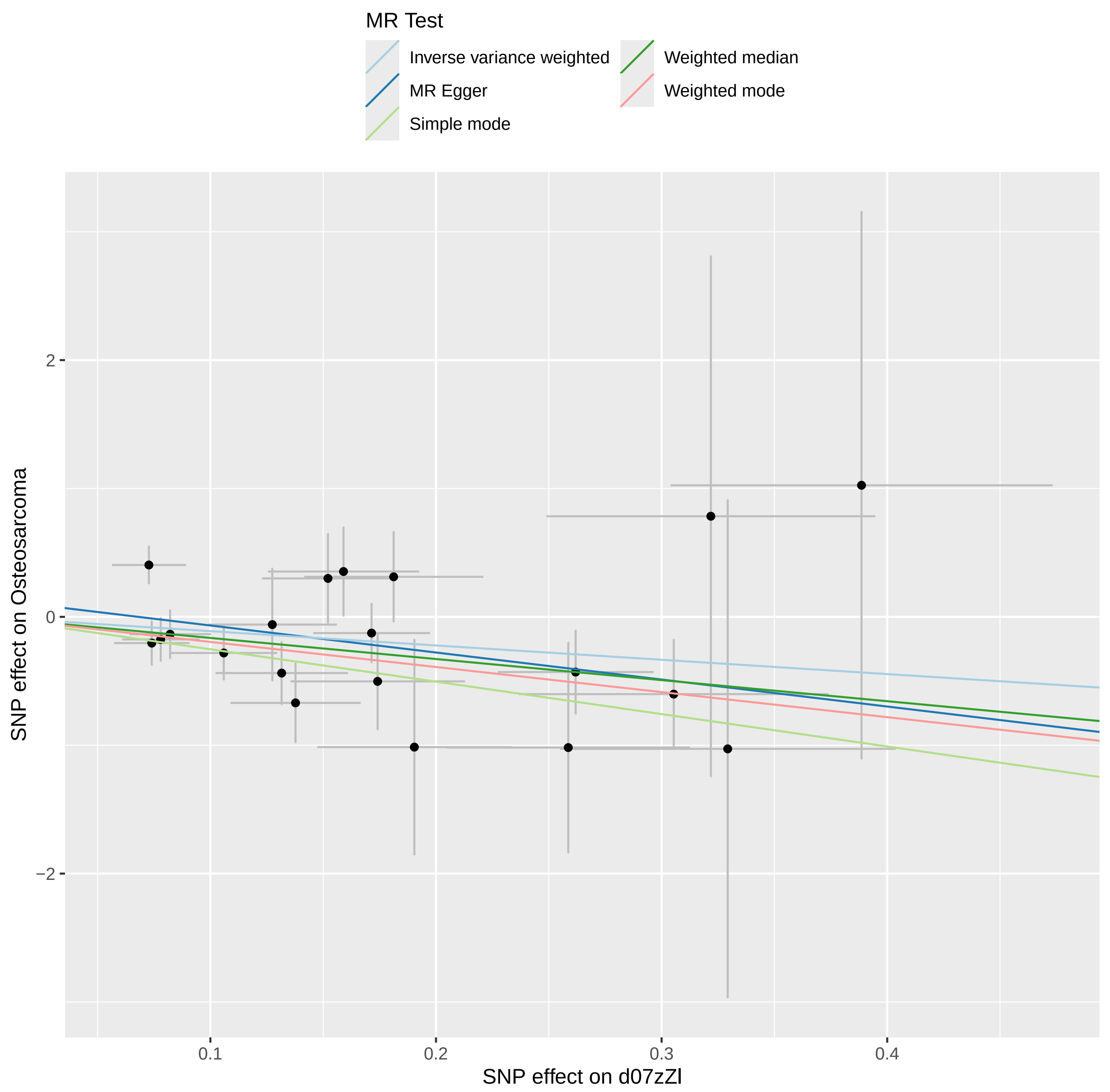

MR Test
Inverse variance weighted
MR Egger
Simple mode
Weighted median
Weighted mode
2
0
−2
0.1
0.2
0.3
0.4
SNP effect on d07zZI
SNP effect on Osteosarcoma

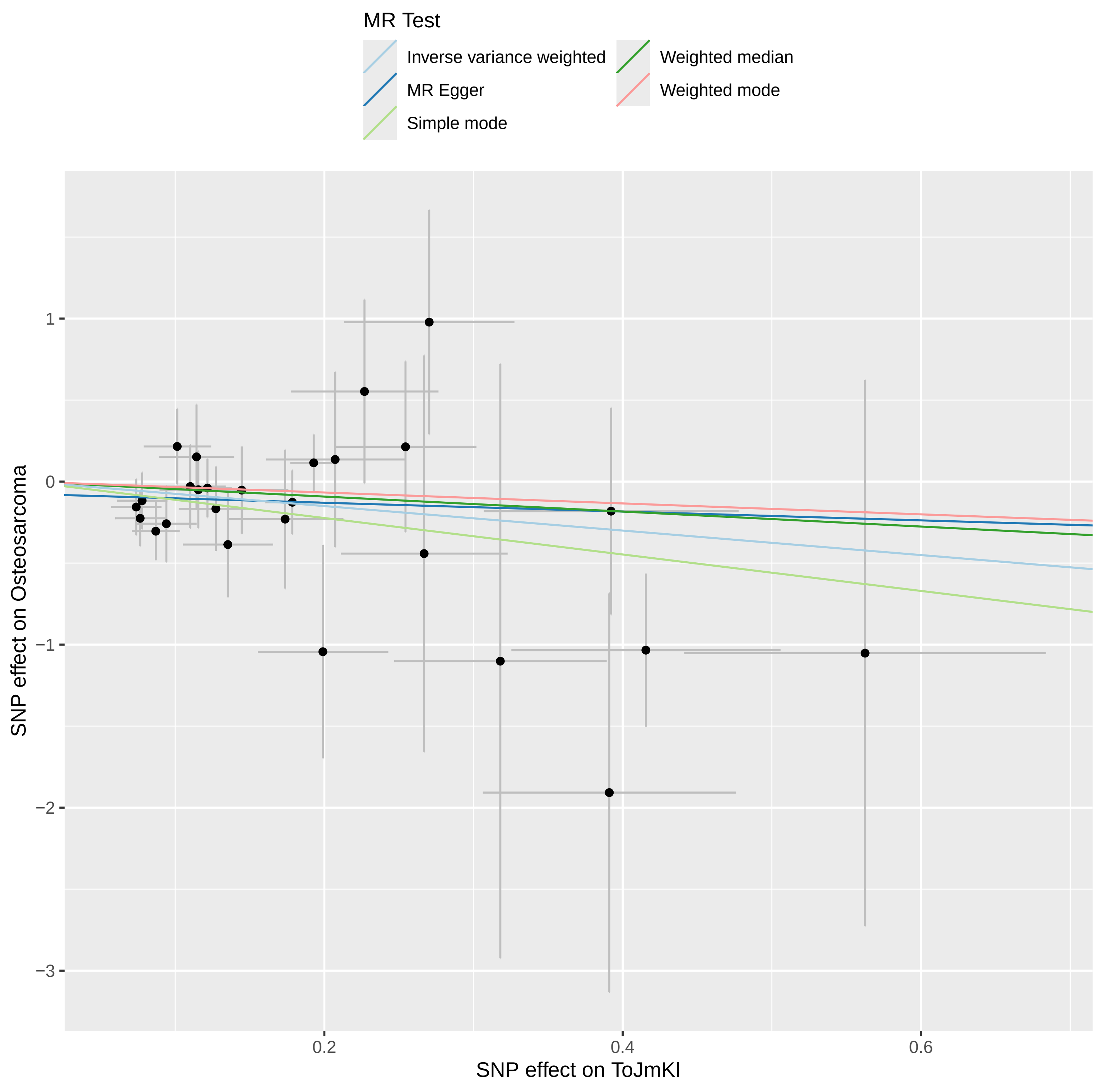
MR Test
Inverse variance weighted
MR Egger
Simple mode
Weighted median
Weighted mode
1
0
−1
−2
−3
0.2
0.4
0.6
SNP effect on Osteosarcoma
SNP effect on ToJmKI

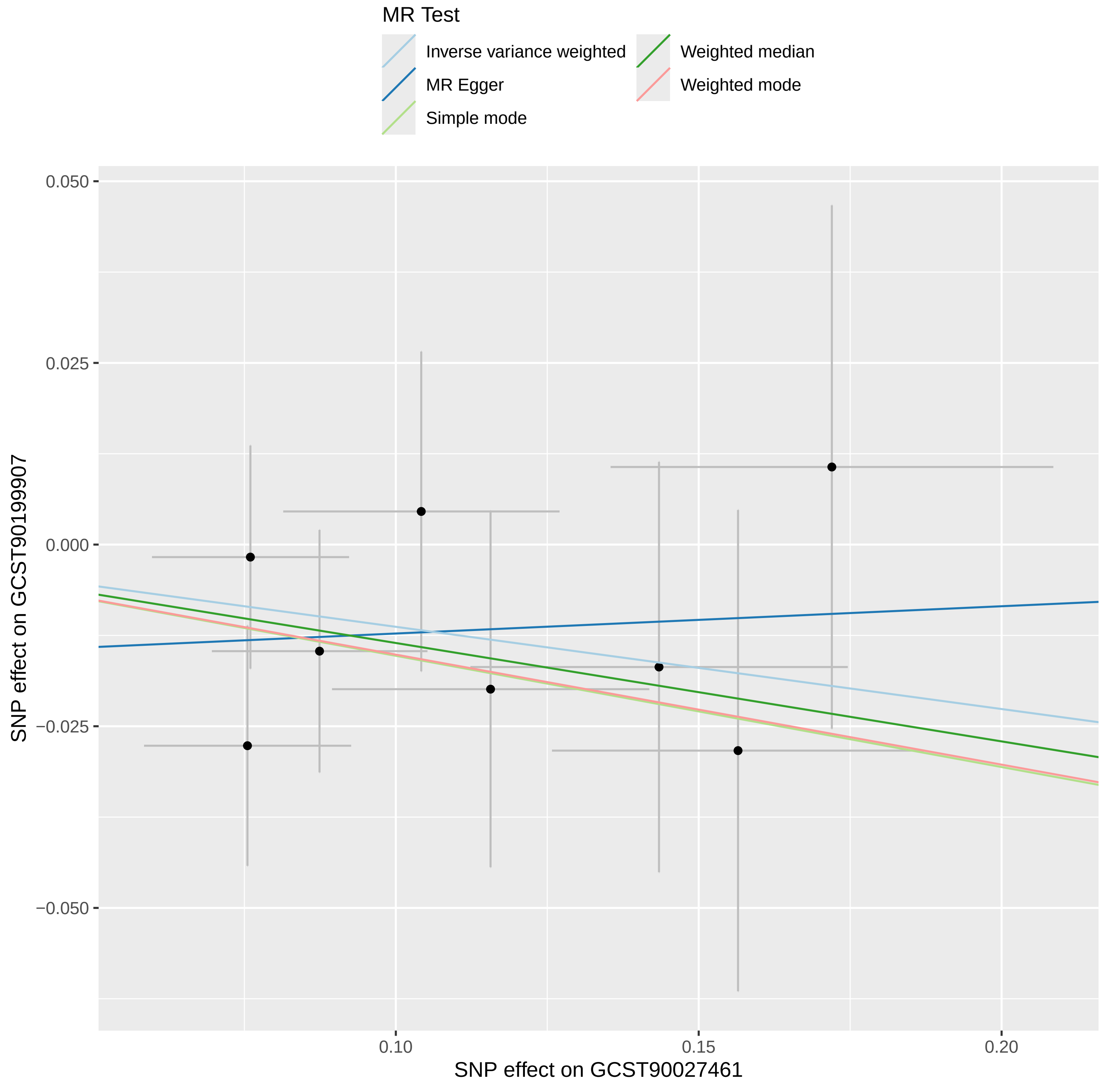

MR Test
Inverse variance weighted
MR Egger
Simple mode
Weighted median
Weighted mode
0.050
0.025
0.000
−0.025
−0.050
SNP effect on GCST90199907
0.10
0.15
0.20
SNP effect on GCST90027461

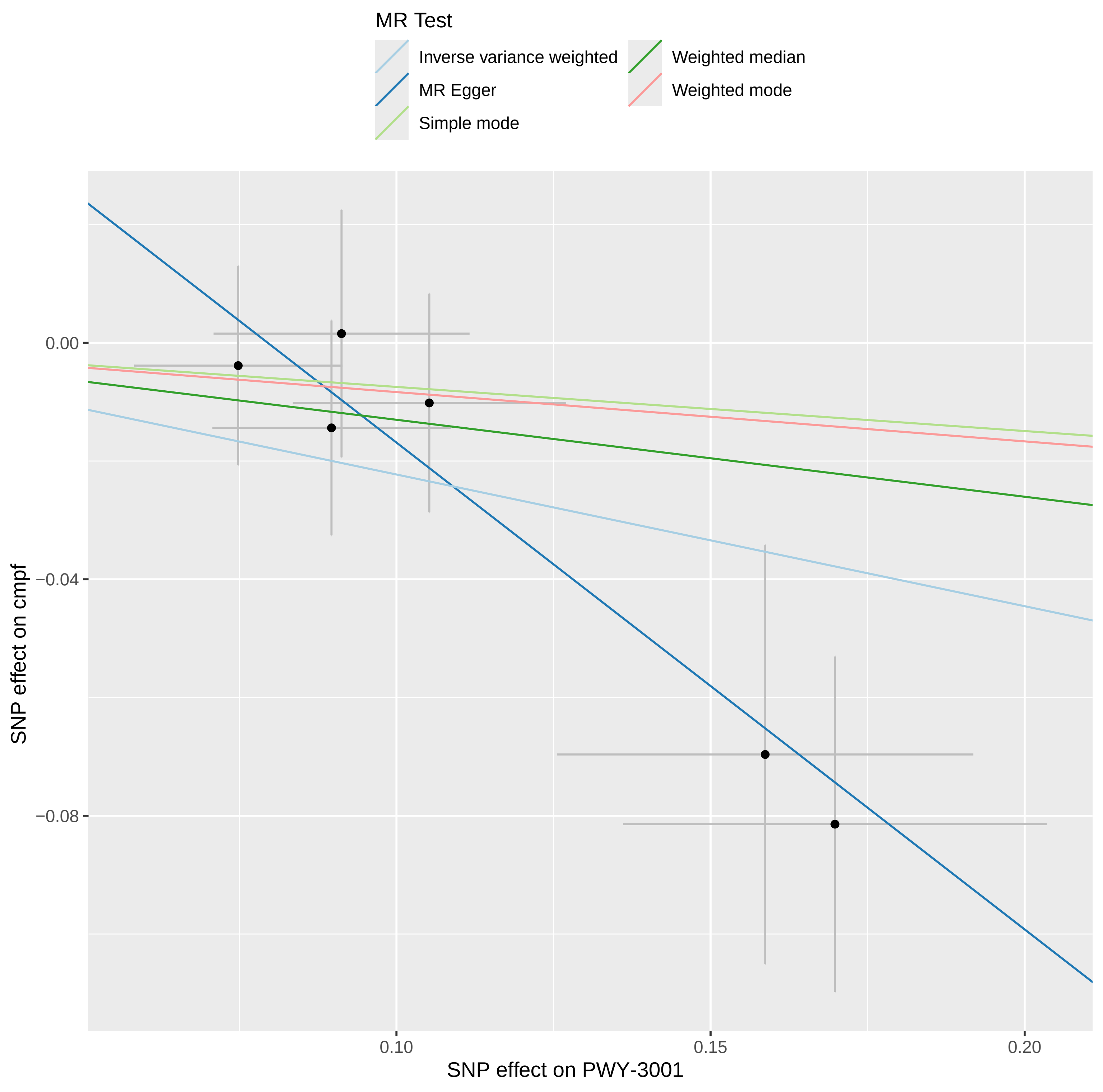

MR Test
Inverse variance weighted
MR Egger
Simple mode
Weighted median
Weighted mode
0.00
−0.04
−0.08
0.10
0.15
0.20
SNP effect on cmpf
SNP effect on PWY-3001

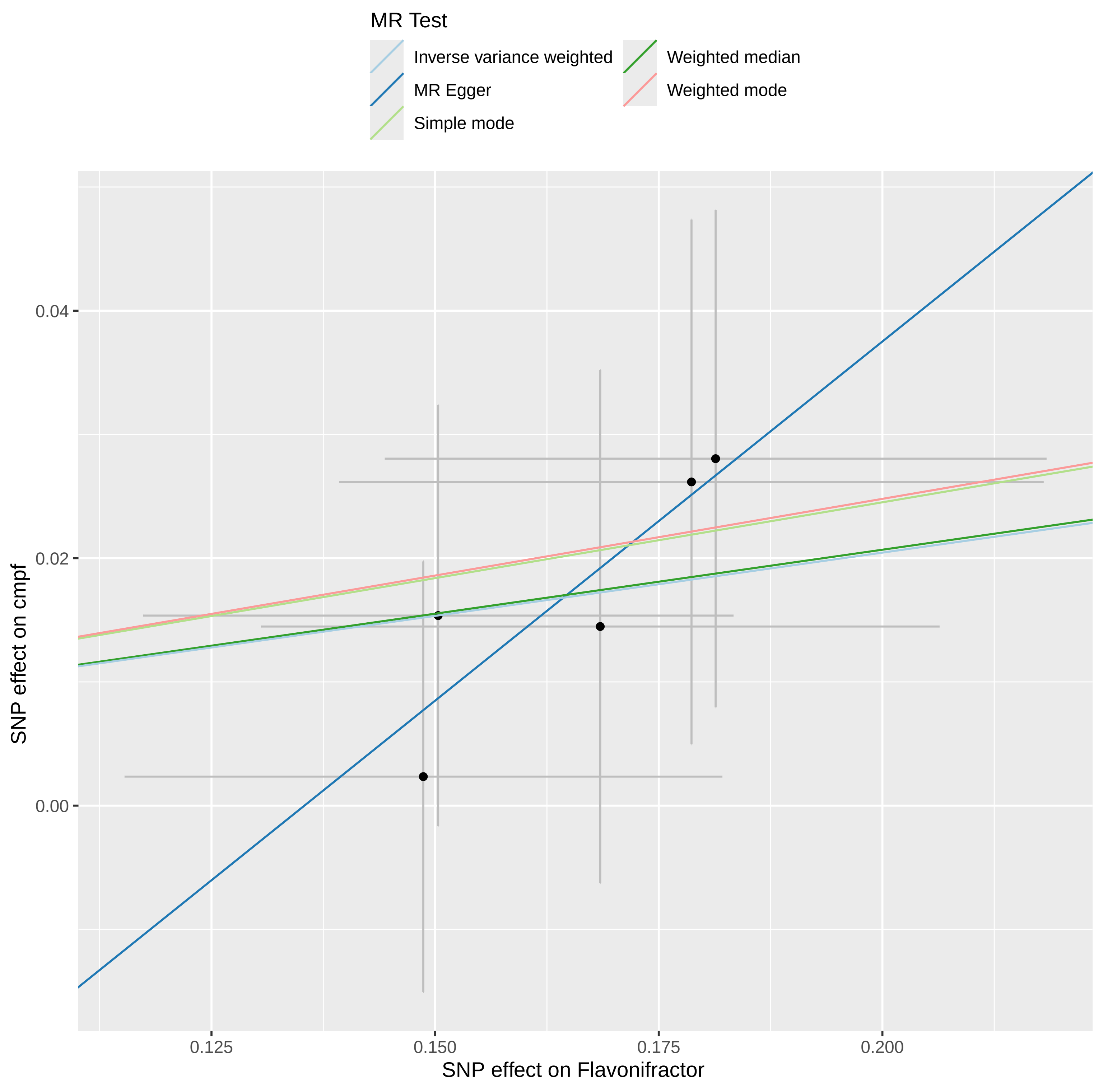
MR Test
Inverse variance weighted
MR Egger
Simple mode
Weighted median
Weighted mode
0.04
0.02
0.00
SNP effect on cmpf
0.125
0.150
0.175
0.200
SNP effect on Flavonifractor

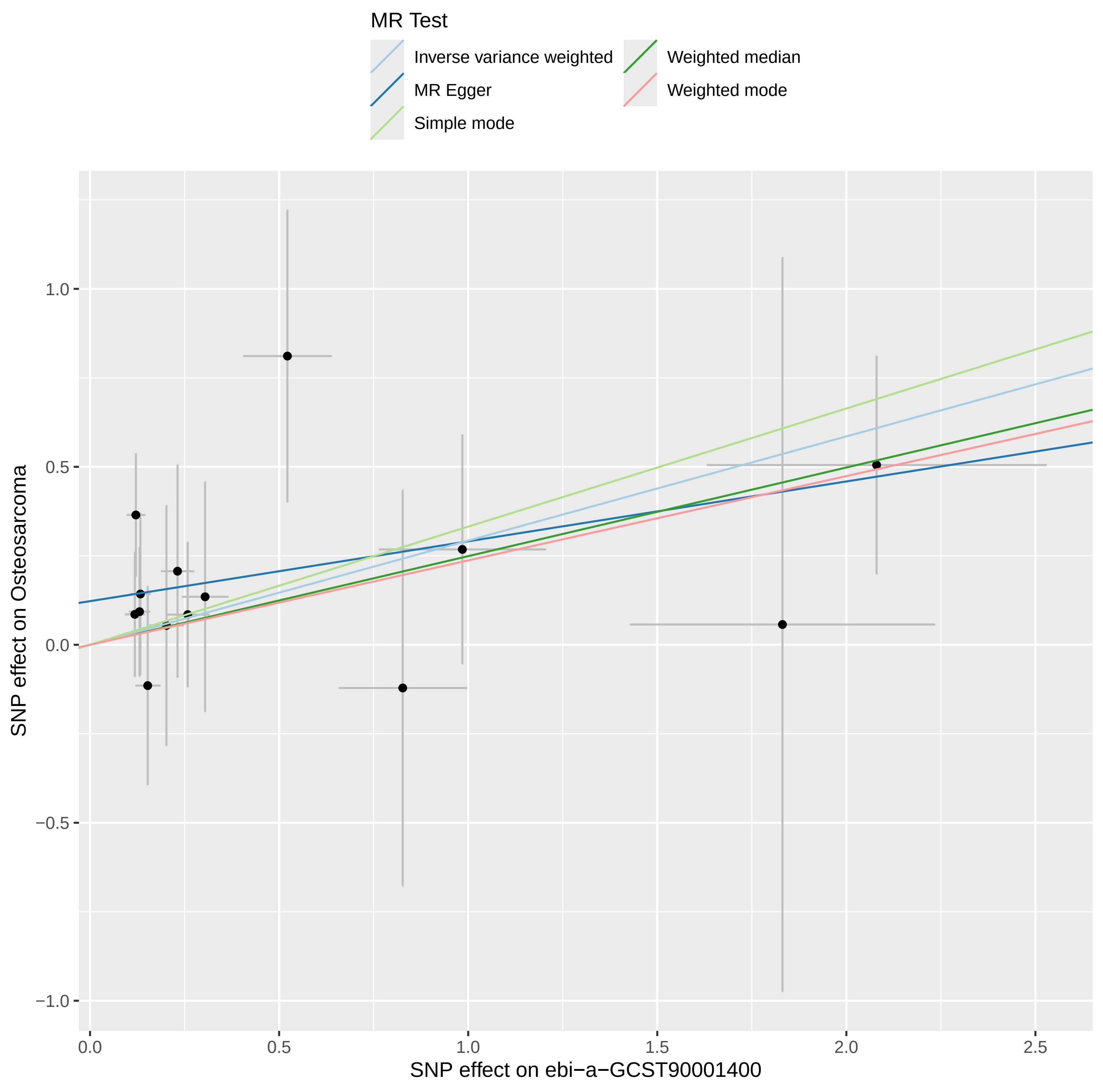

MR Test
Inverse variance weighted
MR Egger
Simple mode
Weighted median
Weighted mode
SNP effect on Osteosarcoma
SNP effect on ebi−a−GCST90001400
1.0
0.5
0.0
−0.5
−1.0
0.0
0.5
1.0
1.5
2.0
2.5

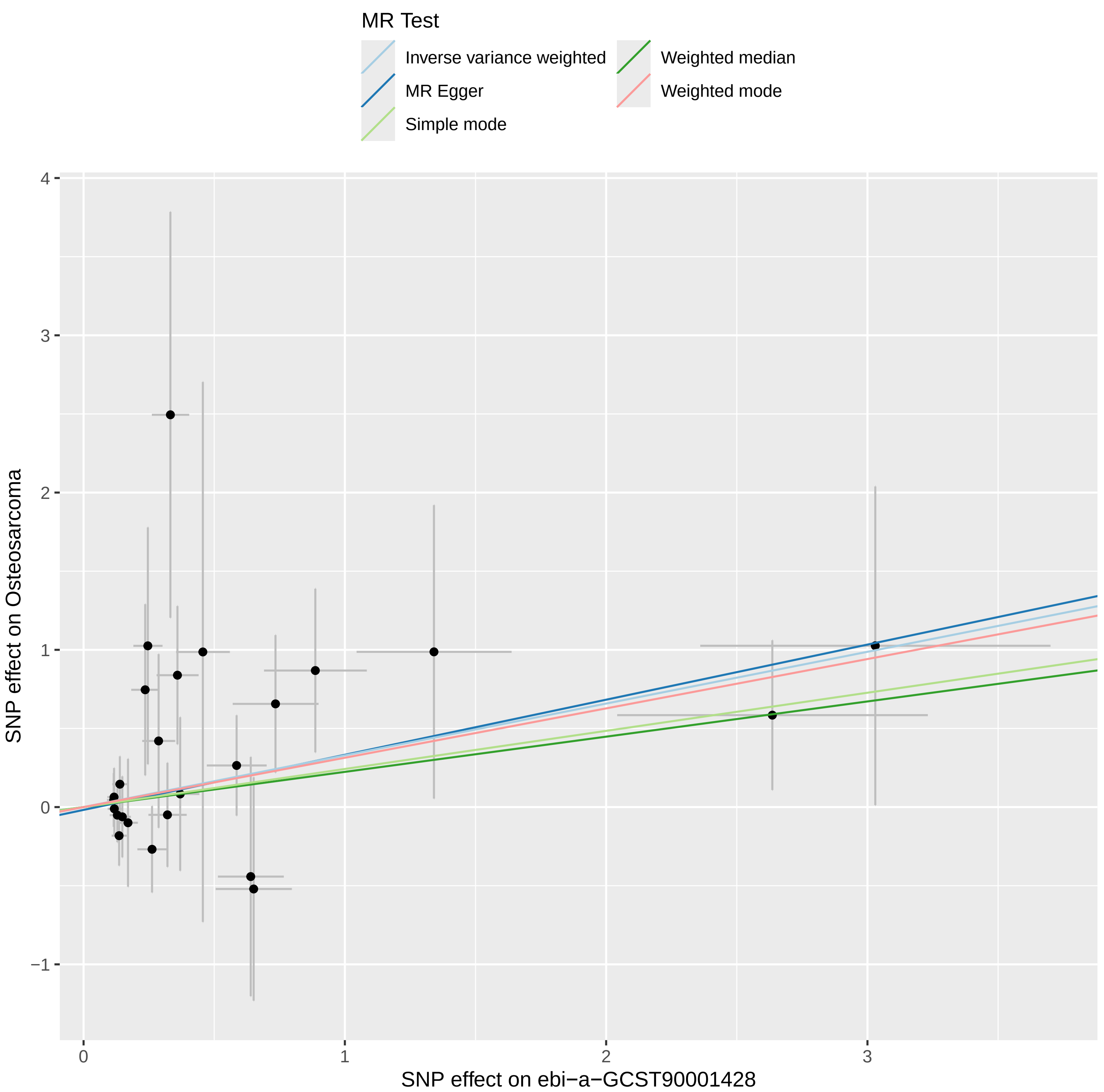

MR Test
Inverse variance weighted
MR Egger
Simple mode
Weighted median
Weighted mode
SNP effect on Osteosarcoma
4
3
2
1
0
−1
0
1
2
3
SNP effect on ebi−a−GCST90001428

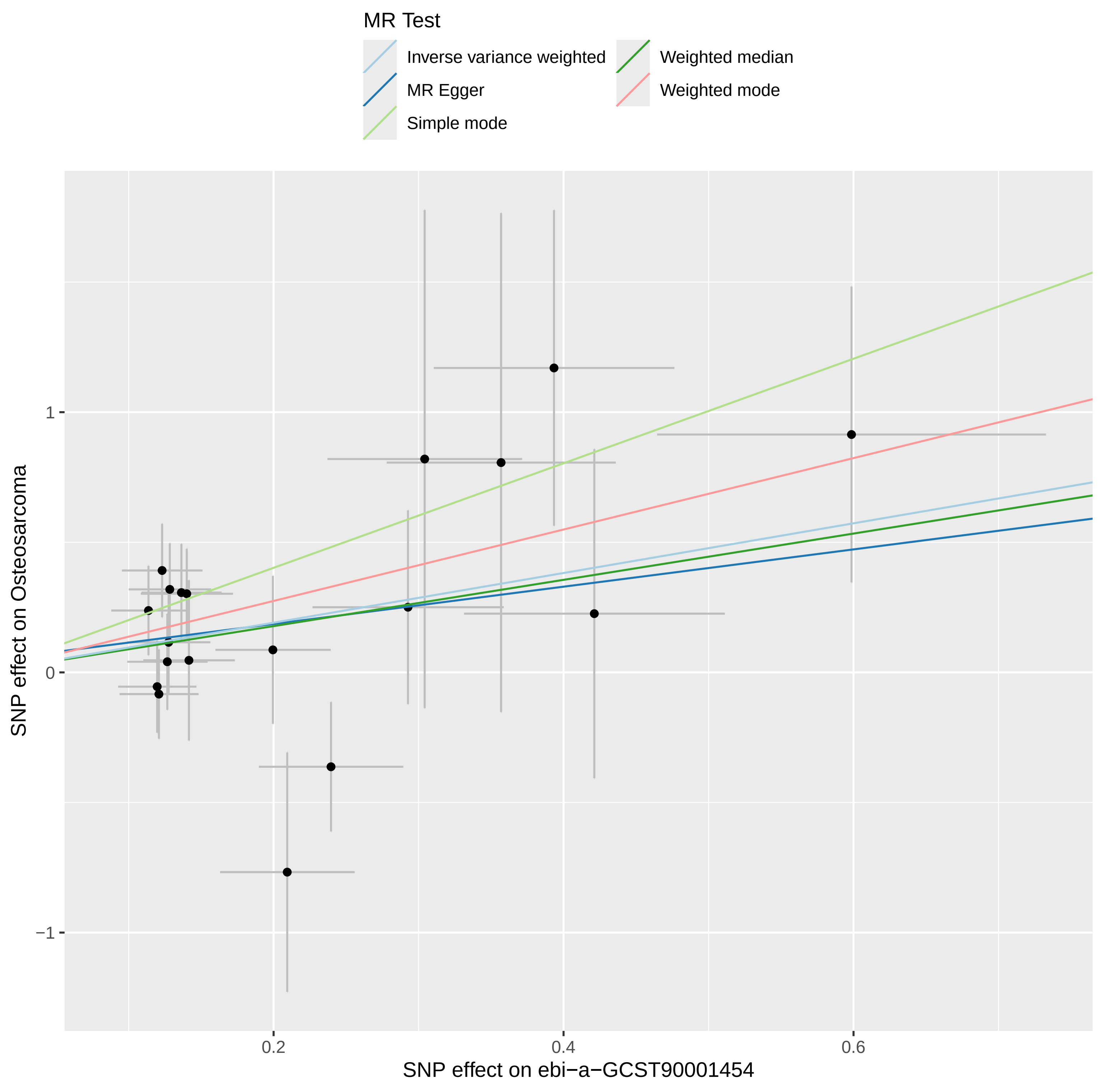

MR Test
Inverse variance weighted
MR Egger
Simple mode
Weighted median
Weighted mode
1
0
−1
0.2
0.4
0.6
SNP effect on Osteosarcoma
SNP effect on ebi−a−GCST90001454

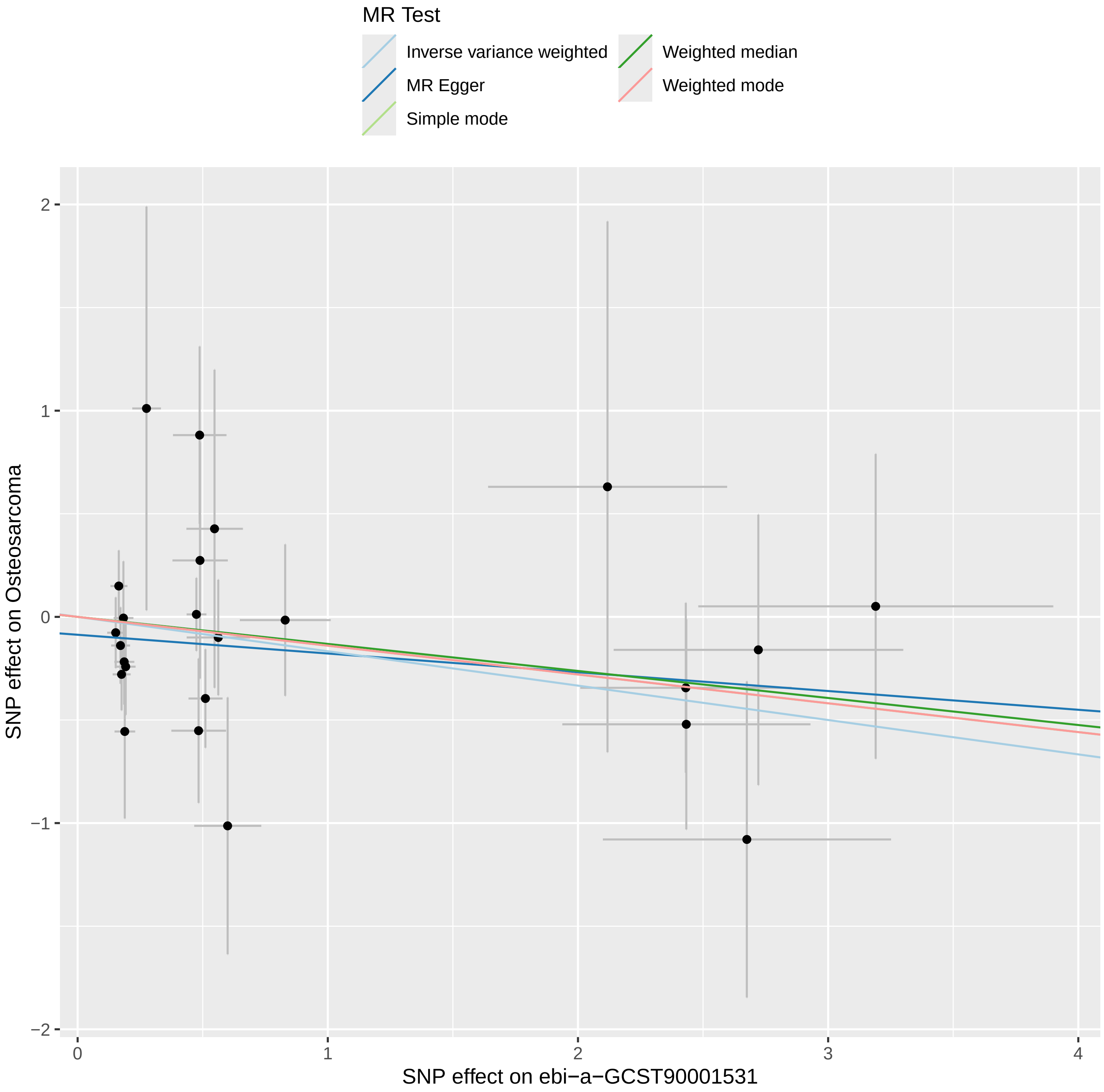

MR Test
Inverse variance weighted
MR Egger
Simple mode
Weighted median
Weighted mode
SNP effect on Osteosarcoma
SNP effect on ebi−a−GCST90001531
2
1
0
−1
−2
0
1
2
3
4

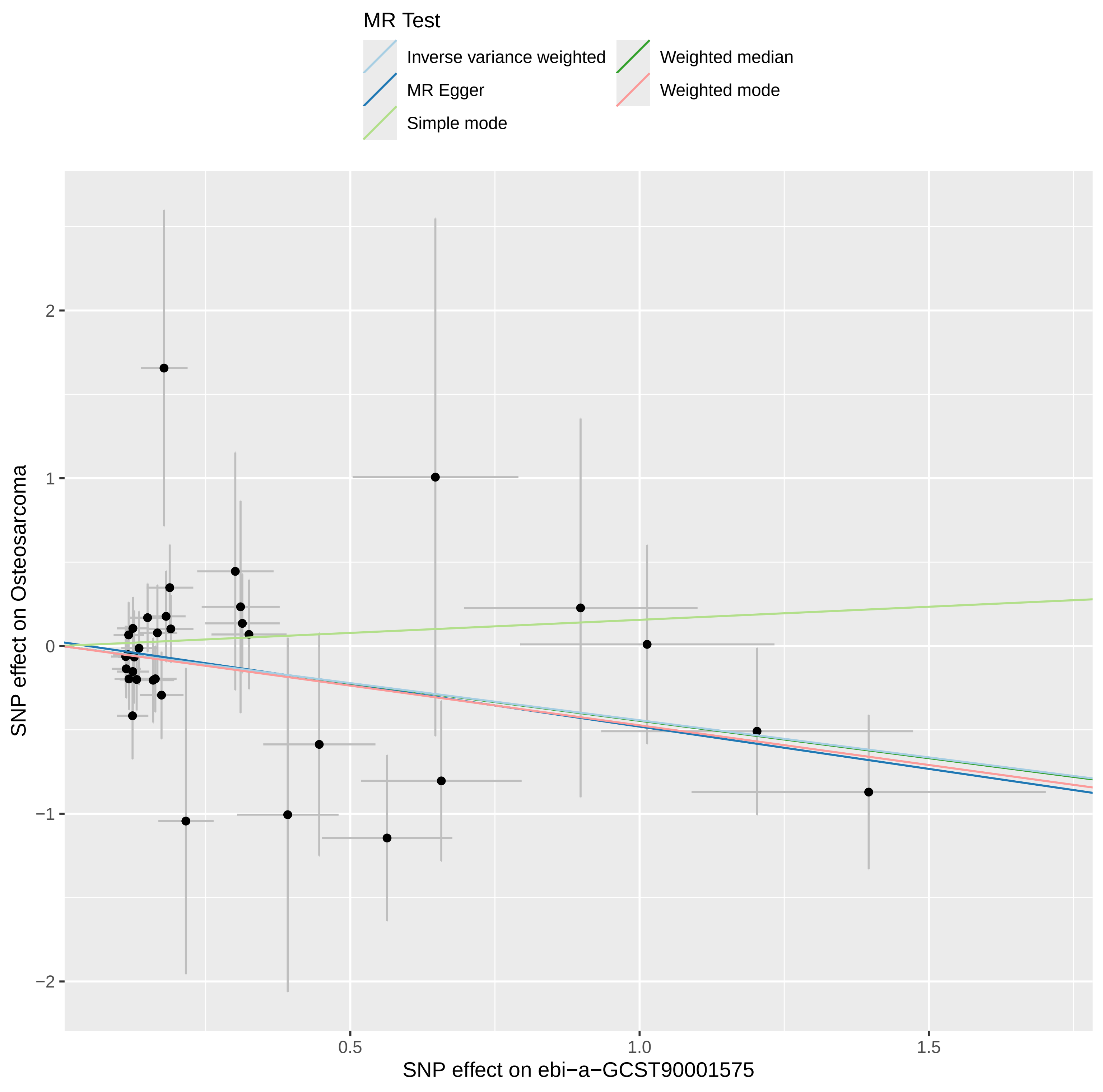

MR Test
Inverse variance weighted
MR Egger
Simple mode
Weighted median
Weighted mode
2
1
0
−1
−2
0.5
1.0
1.5
SNP effect on Osteosarcoma
SNP effect on ebi−a−GCST90001575

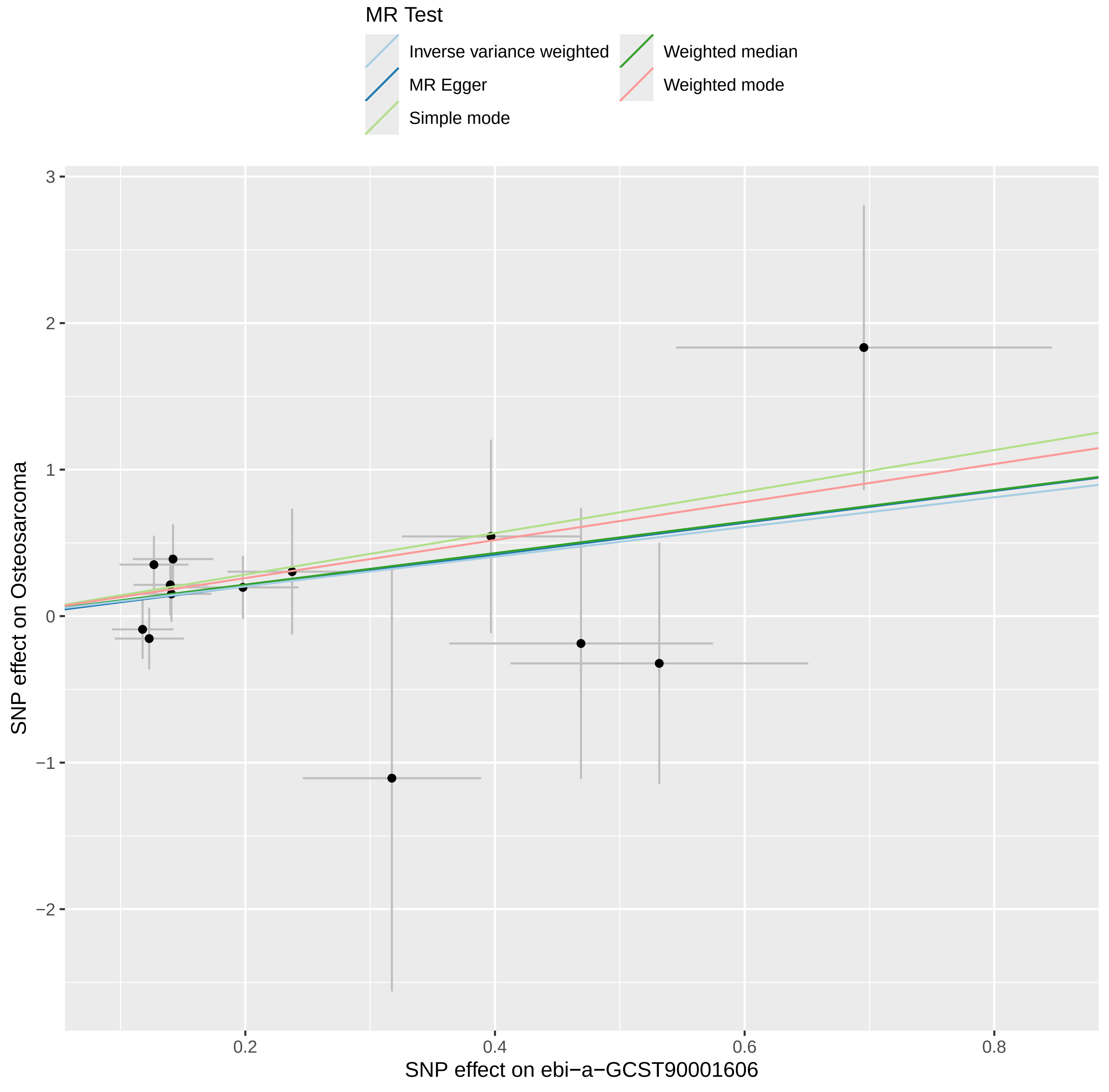
MR Test
Inverse variance weighted
MR Egger
Simple mode
Weighted median
Weighted mode
SNP effect on Osteosarcoma
SNP effect on ebi−a−GCST90001606
3
2
1
0
−1
−2
0.2
0.4
0.6
0.8

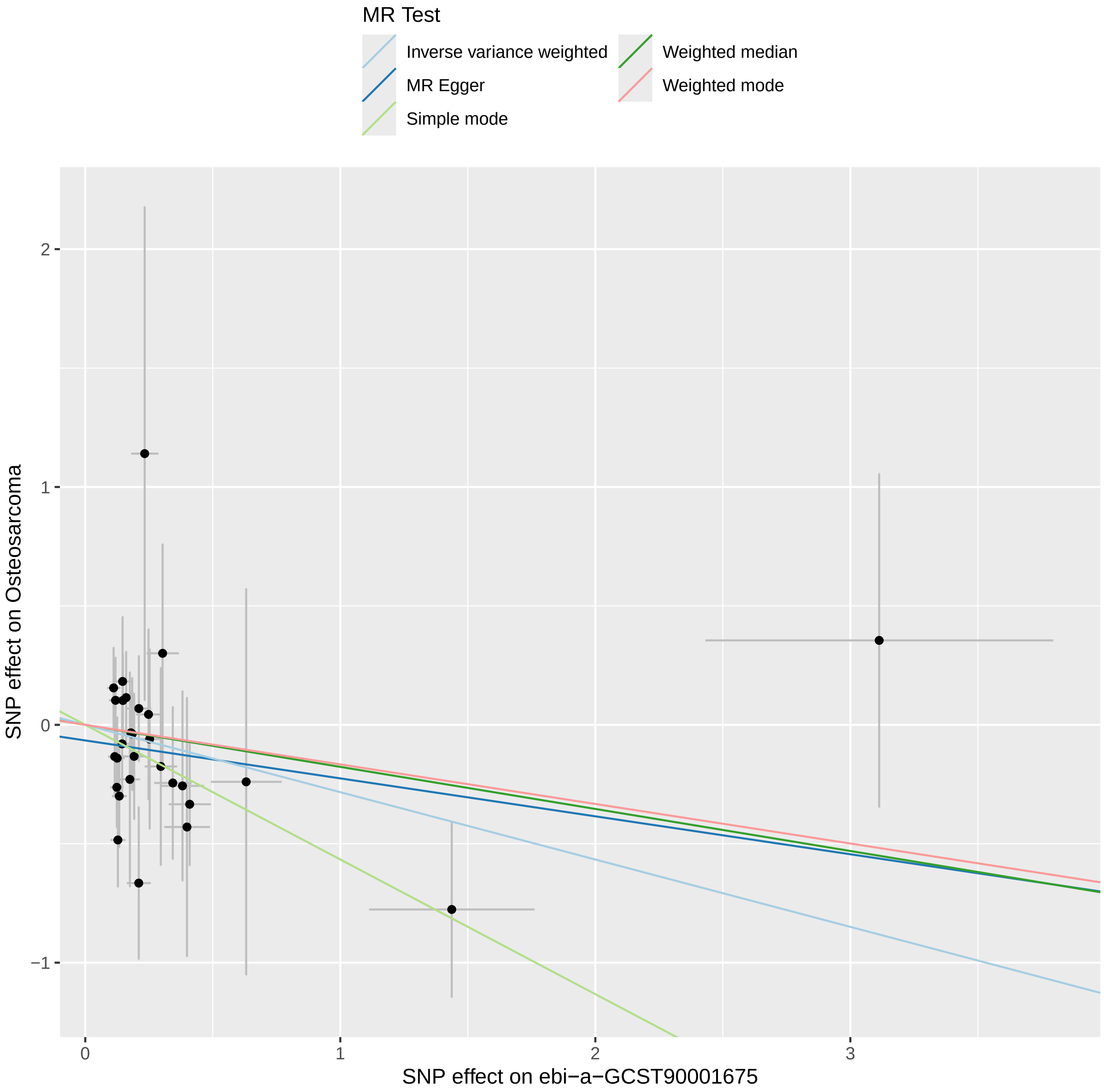

MR Test
Inverse variance weighted
MR Egger
Simple mode
Weighted median
Weighted mode
SNP effect on Osteosarcoma
2
1
0
−1
0
1
2
3
SNP effect on ebi−a−GCST90001675

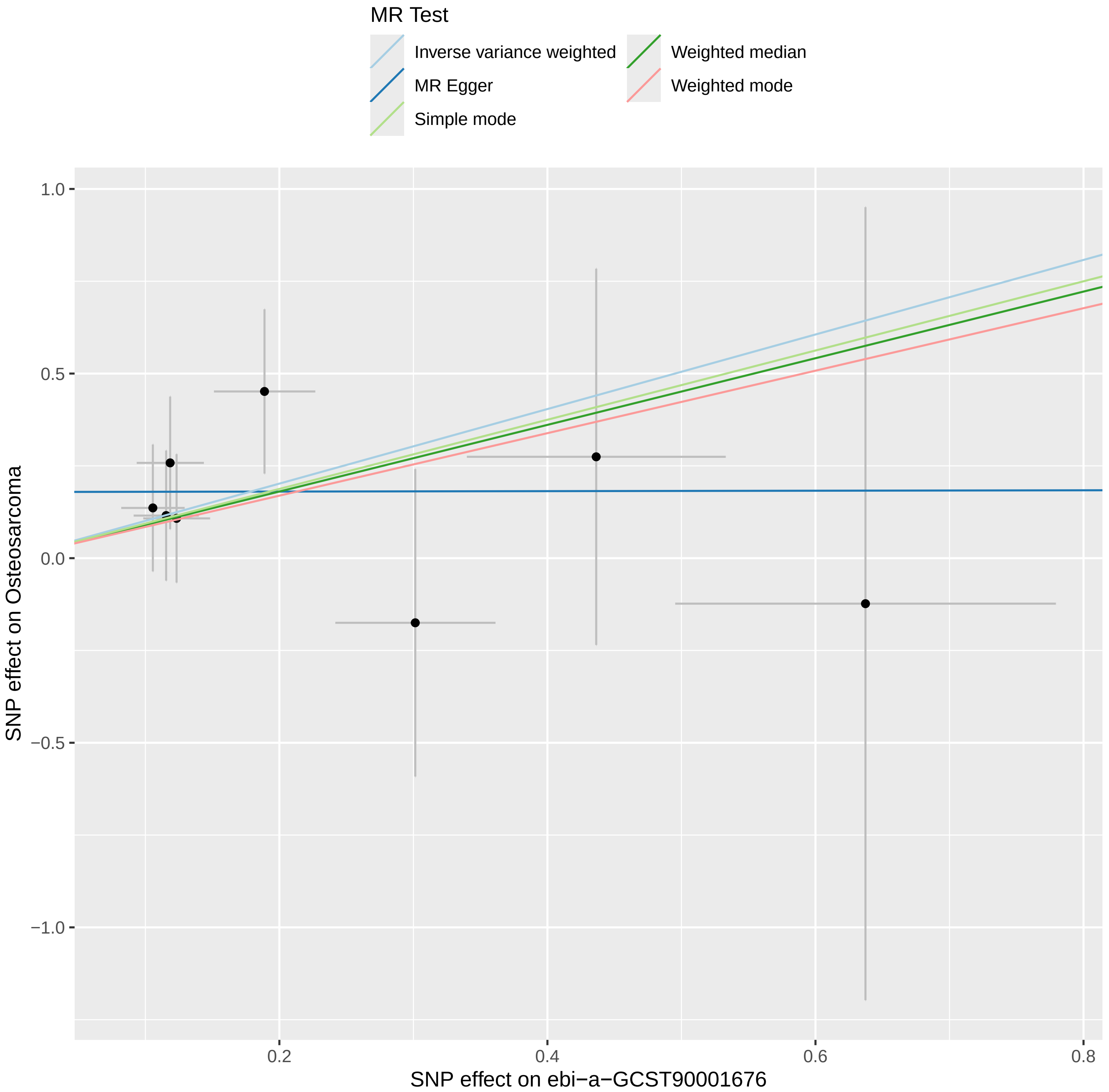

MR Test
Inverse variance weighted
MR Egger
Simple mode
Weighted median
Weighted mode
1.0
0.5
0.0
−0.5
−1.0
0.2
0.4
0.6
0.8
SNP effect on Osteosarcoma
SNP effect on ebi−a−GCST90001676

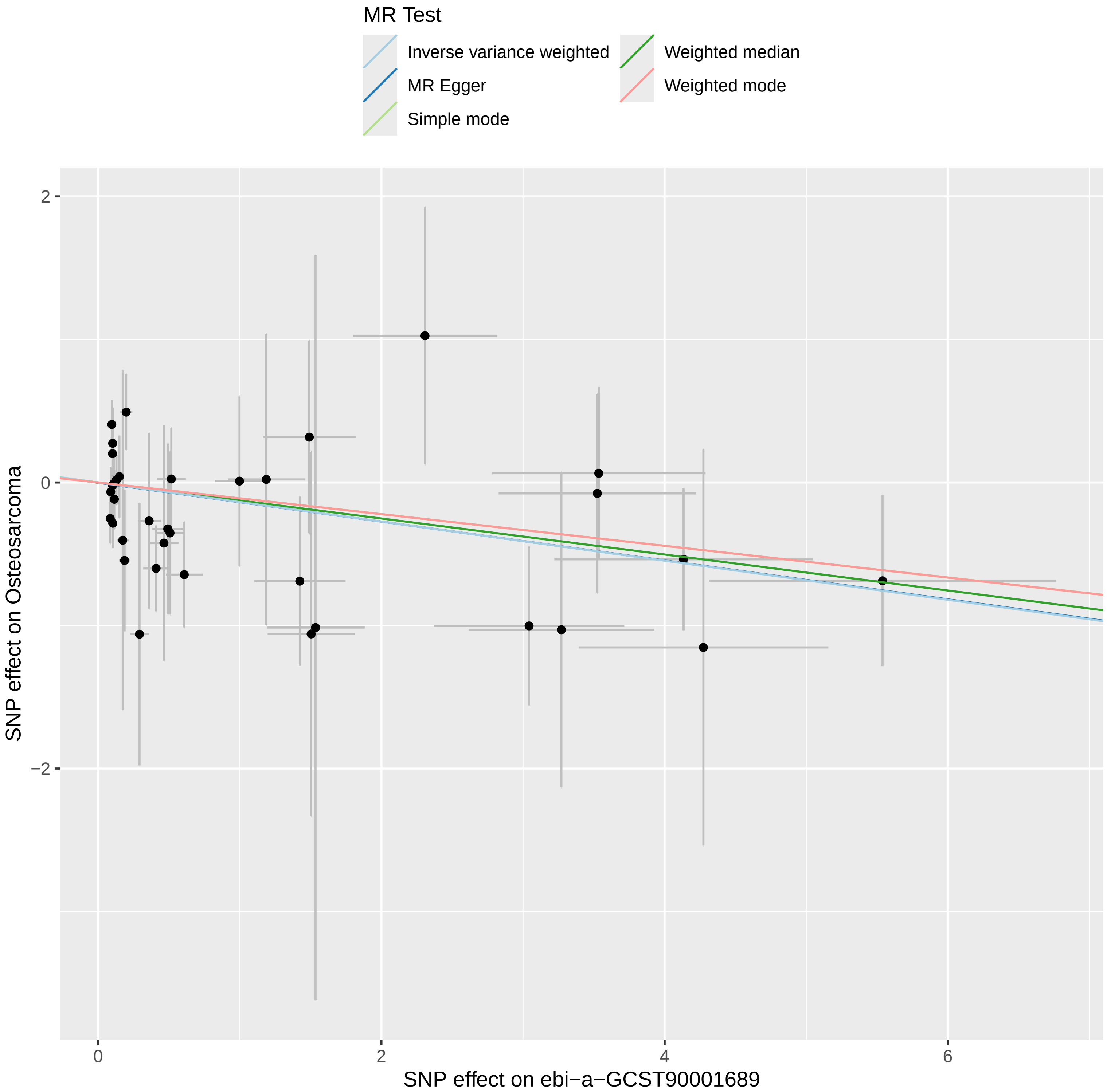
MR Test
Inverse variance weighted
MR Egger
Simple mode
Weighted median
Weighted mode
SNP effect on Osteosarcoma
SNP effect on ebi−a−GCST90001689
2
0
−2
0
2
4
6

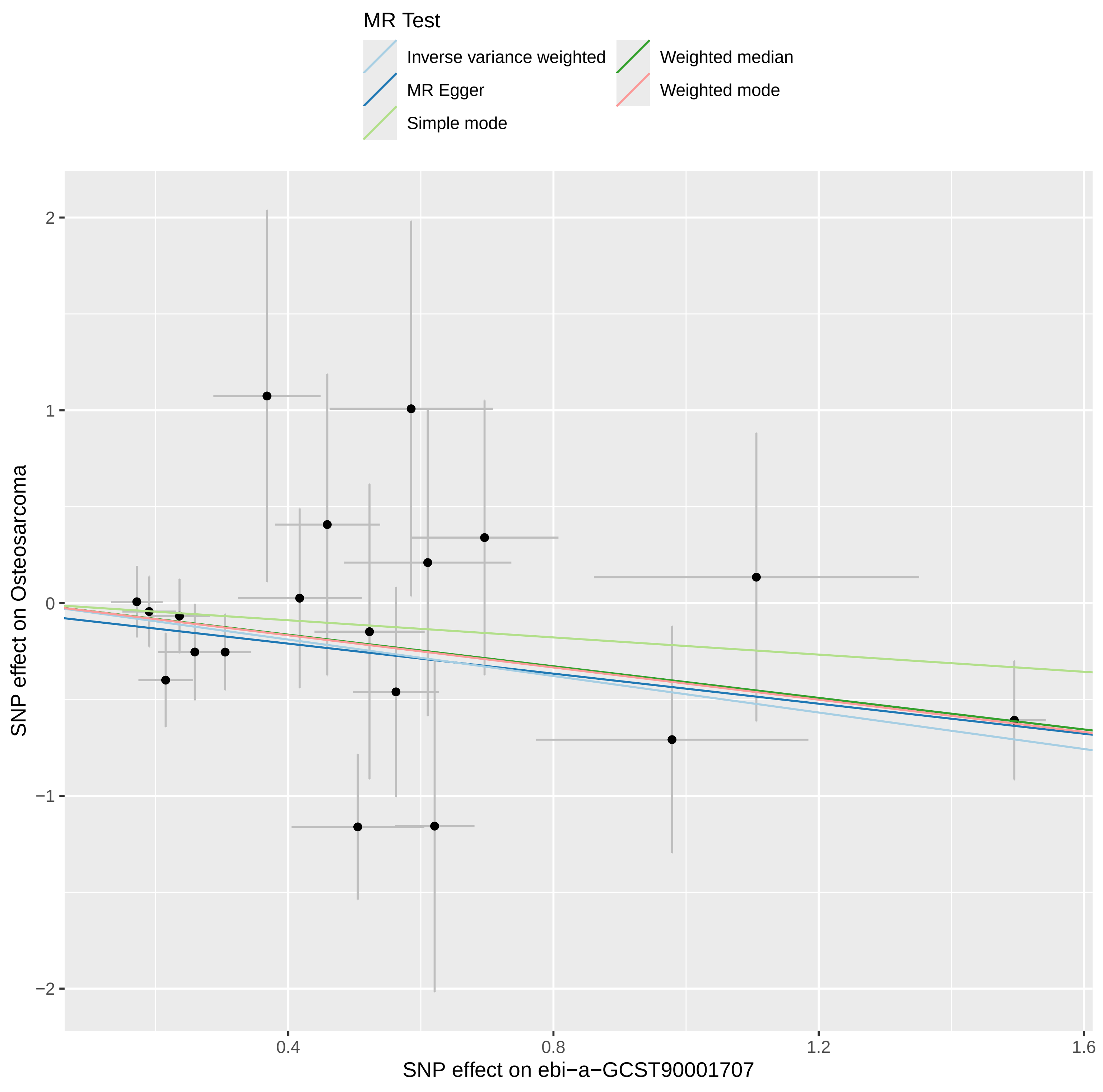
MR Test
Inverse variance weighted
MR Egger
Simple mode
Weighted median
Weighted mode
SNP effect on Osteosarcoma
2
1
0
−1
−2
0.4
0.8
1.2
1.6
SNP effect on ebi−a−GCST90001707

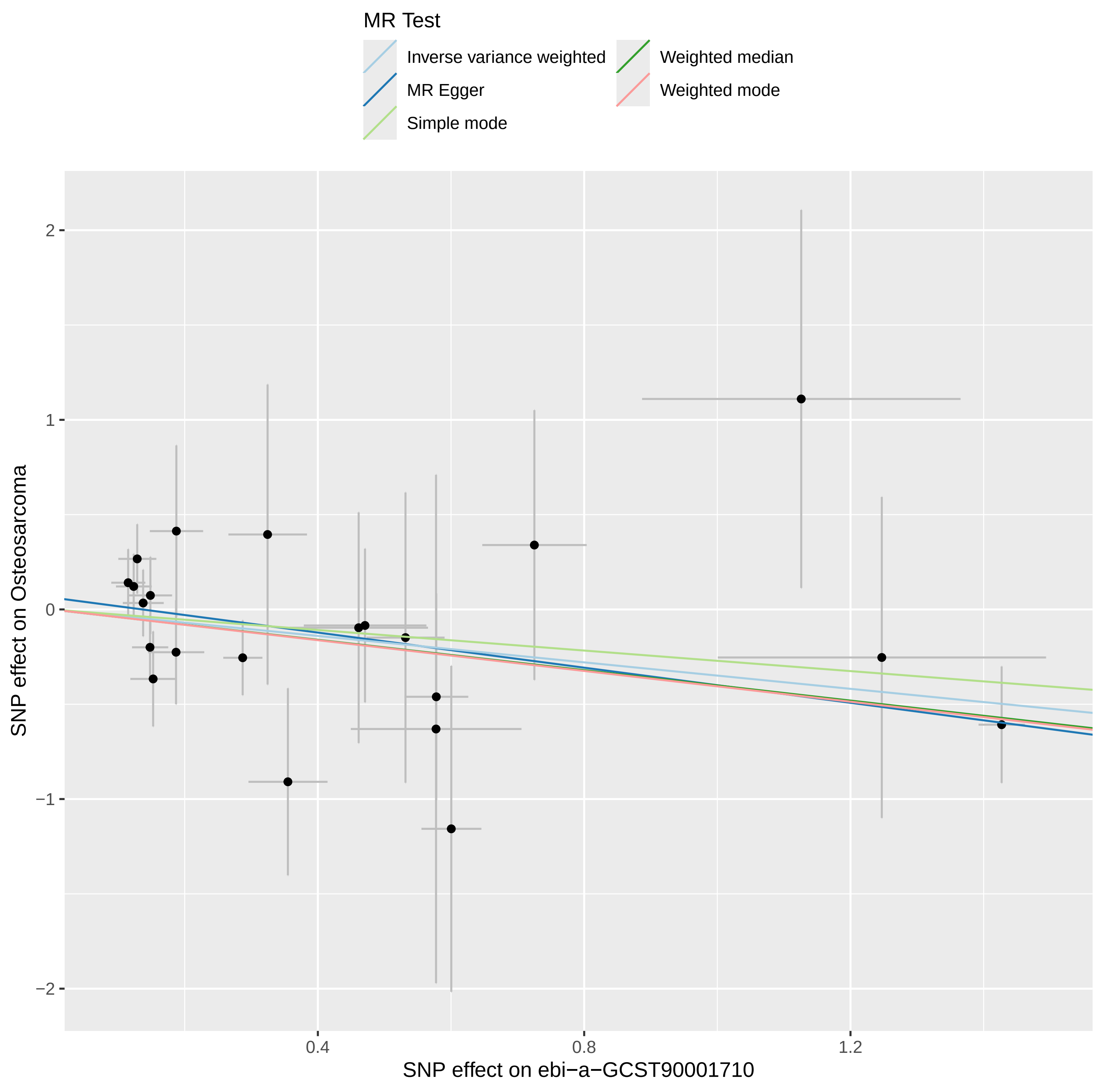

MR Test
Inverse variance weighted
MR Egger
Simple mode
Weighted median
Weighted mode
SNP effect on Osteosarcoma
2
1
0
−1
−2
0.4
0.8
1.2
SNP effect on ebi−a−GCST90001710

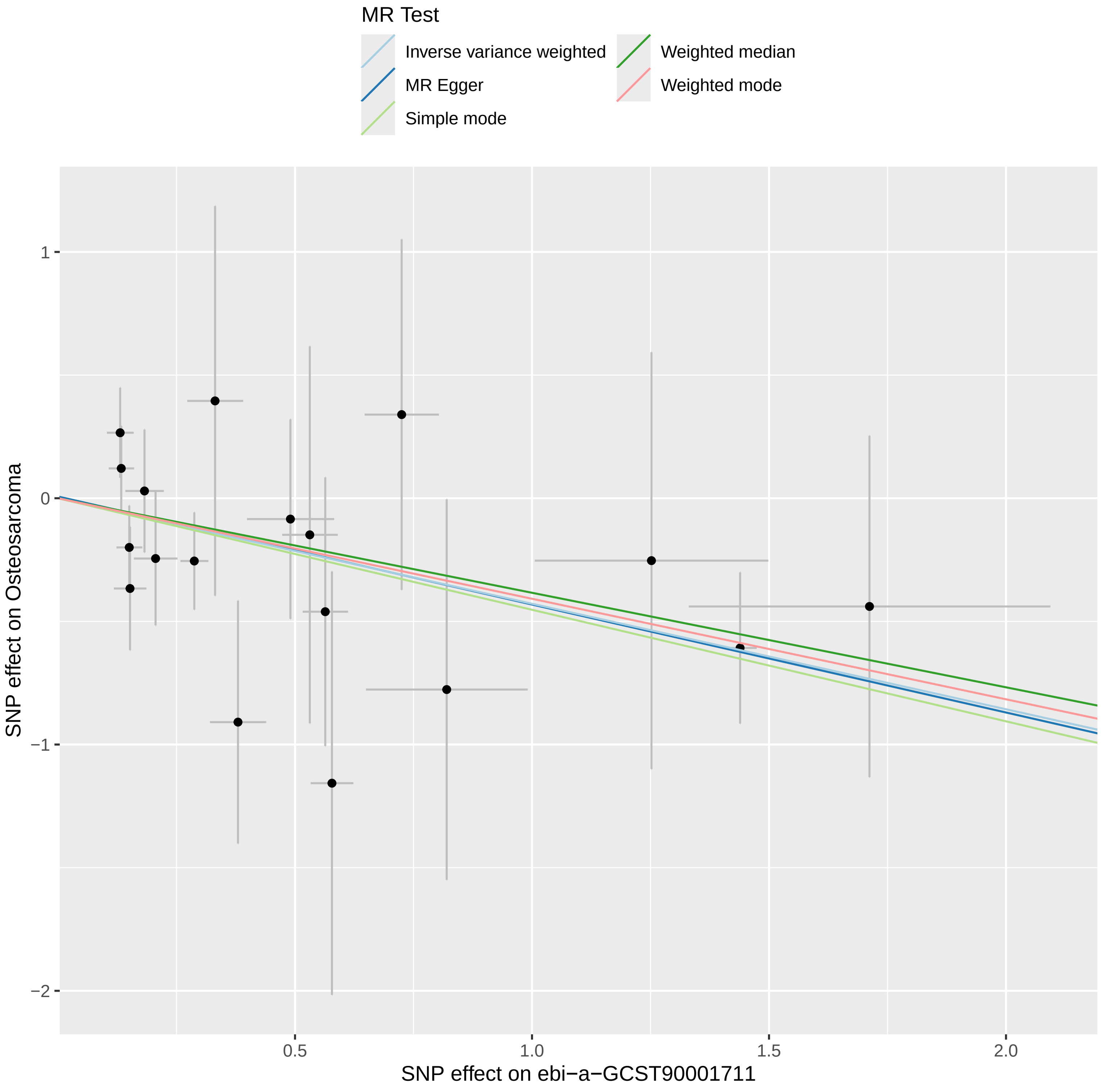

MR Test
Inverse variance weighted
MR Egger
Simple mode
Weighted median
Weighted mode
1
0
−1
−2
SNP effect on Osteosarcoma
0.5
1.0
1.5
2.0
SNP effect on ebi−a−GCST90001711

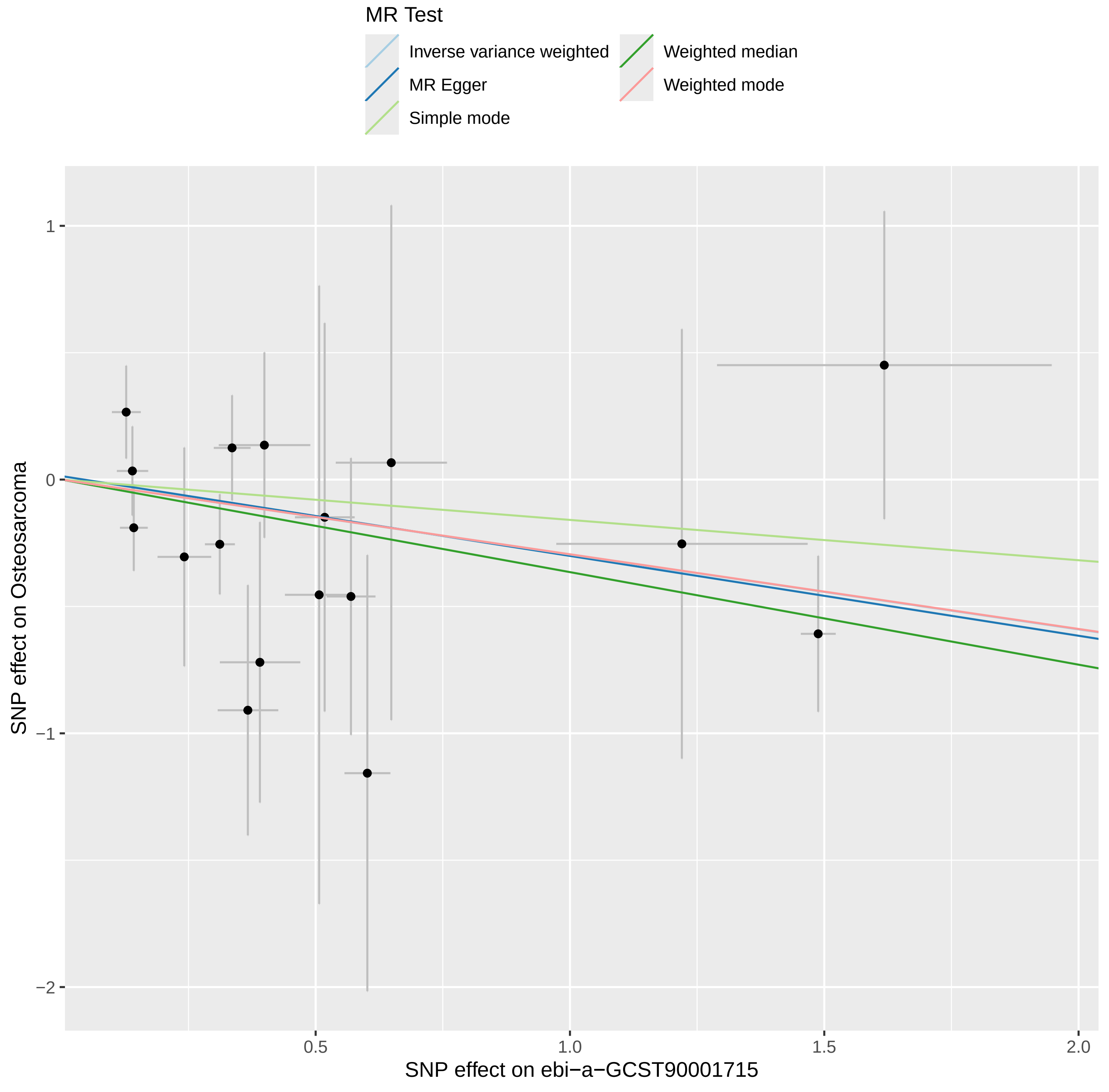

MR Test
Inverse variance weighted
MR Egger
Simple mode
Weighted median
Weighted mode
1
0
−1
−2
0.5
1.0
1.5
2.0
SNP effect on Osteosarcoma
SNP effect on ebi−a−GCST90001715

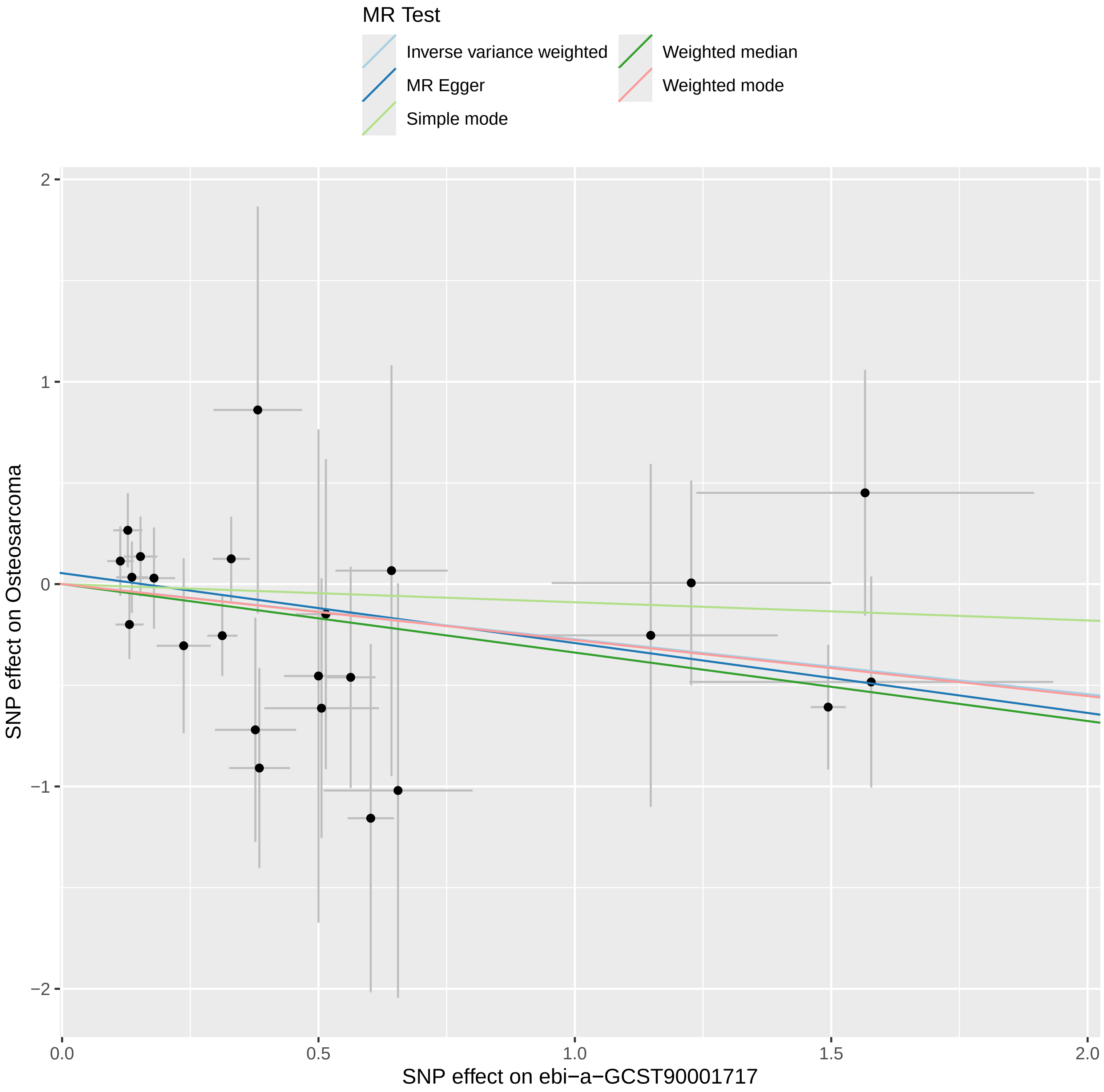

MR Test
Inverse variance weighted
MR Egger
Simple mode
Weighted median
Weighted mode
SNP effect on Osteosarcoma
SNP effect on ebi−a−GCST90001717
2
1
0
−1
−2
0.0
0.5
1.0
1.5
2.0

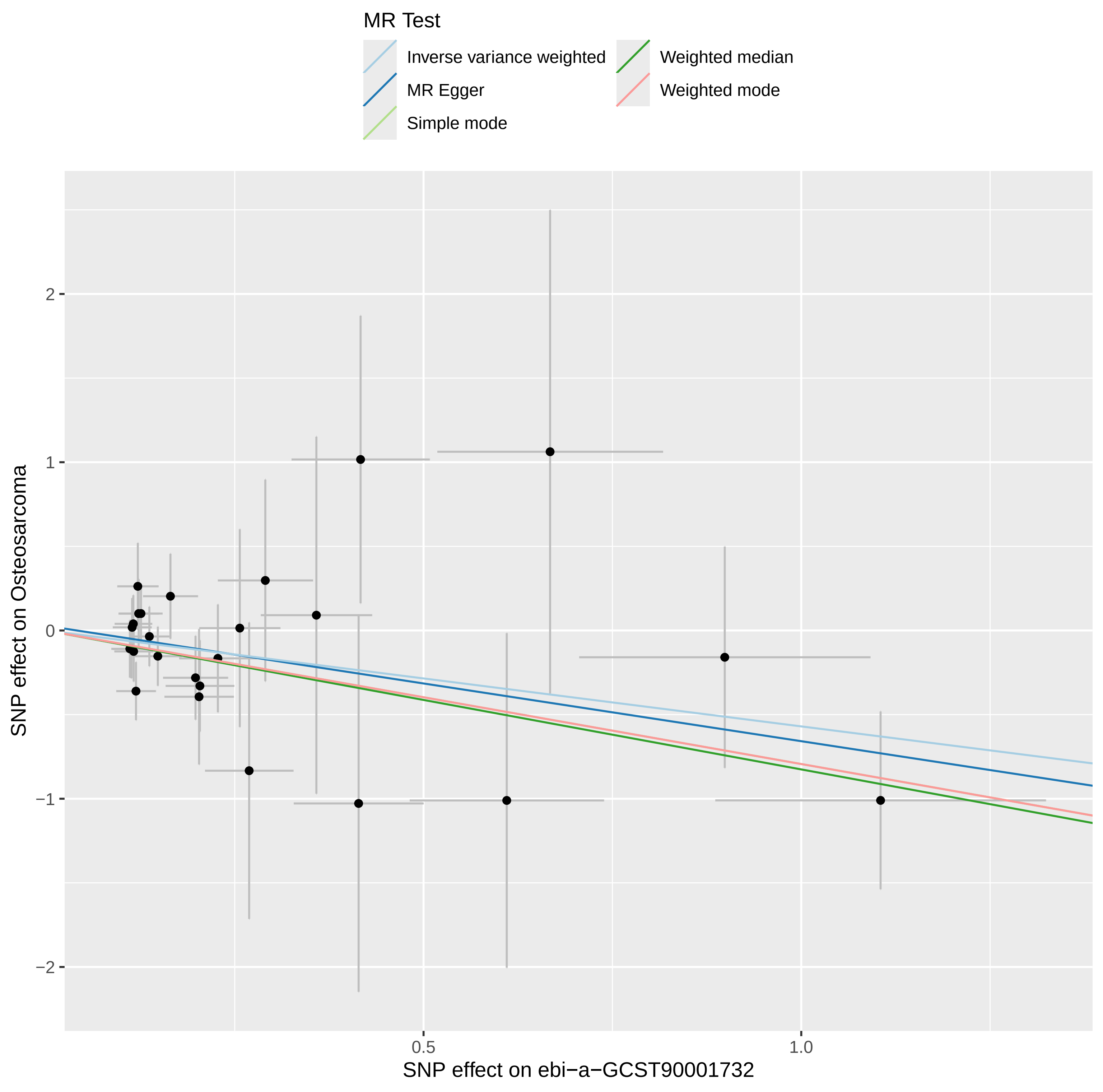

MR Test
Inverse variance weighted
MR Egger
Simple mode
Weighted median
Weighted mode
2
1
0
−1
−2
0.5
1.0
SNP effect on Osteosarcoma
SNP effect on ebi−a−GCST90001732

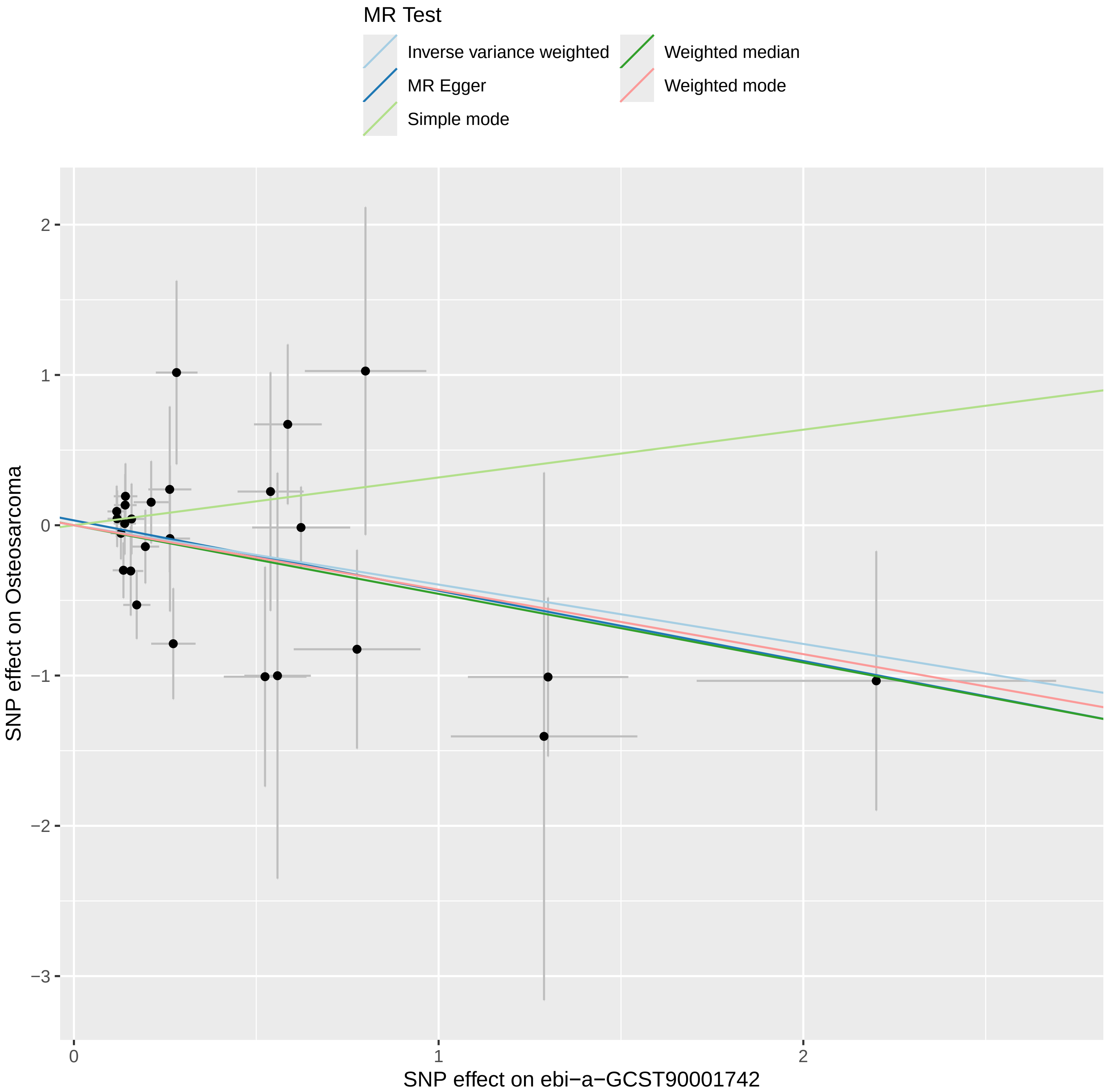

MR Test
Inverse variance weighted
MR Egger
Simple mode
Weighted median
Weighted mode
2
1
0
−1
−2
−3
SNP effect on Osteosarcoma
0
1
2
SNP effect on ebi−a−GCST90001742

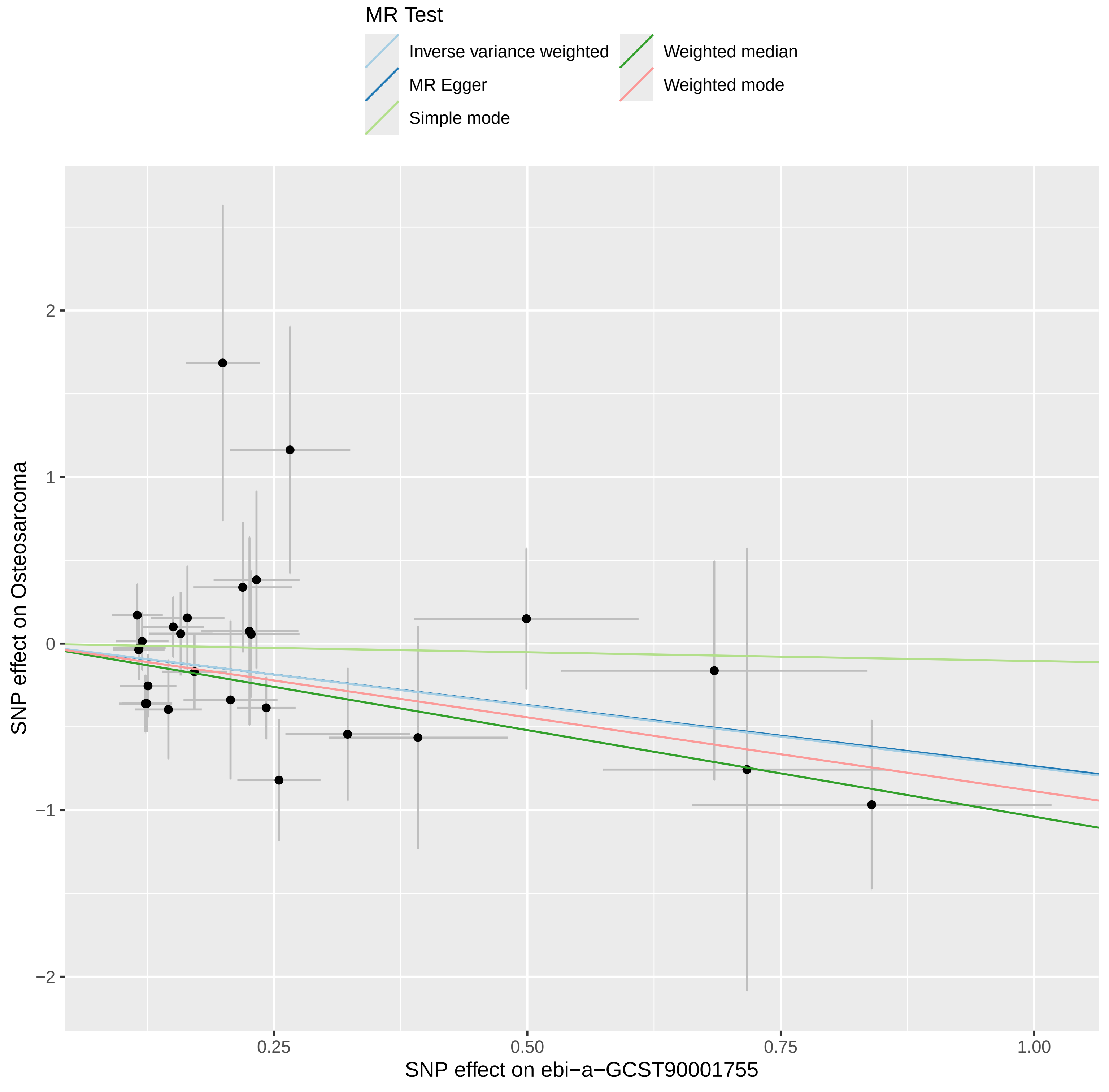

MR Test
Inverse variance weighted
MR Egger
Simple mode
Weighted median
Weighted mode
SNP effect on Osteosarcoma
2
1
0
−1
−2
0.25
0.50
0.75
1.00
SNP effect on ebi−a−GCST90001755

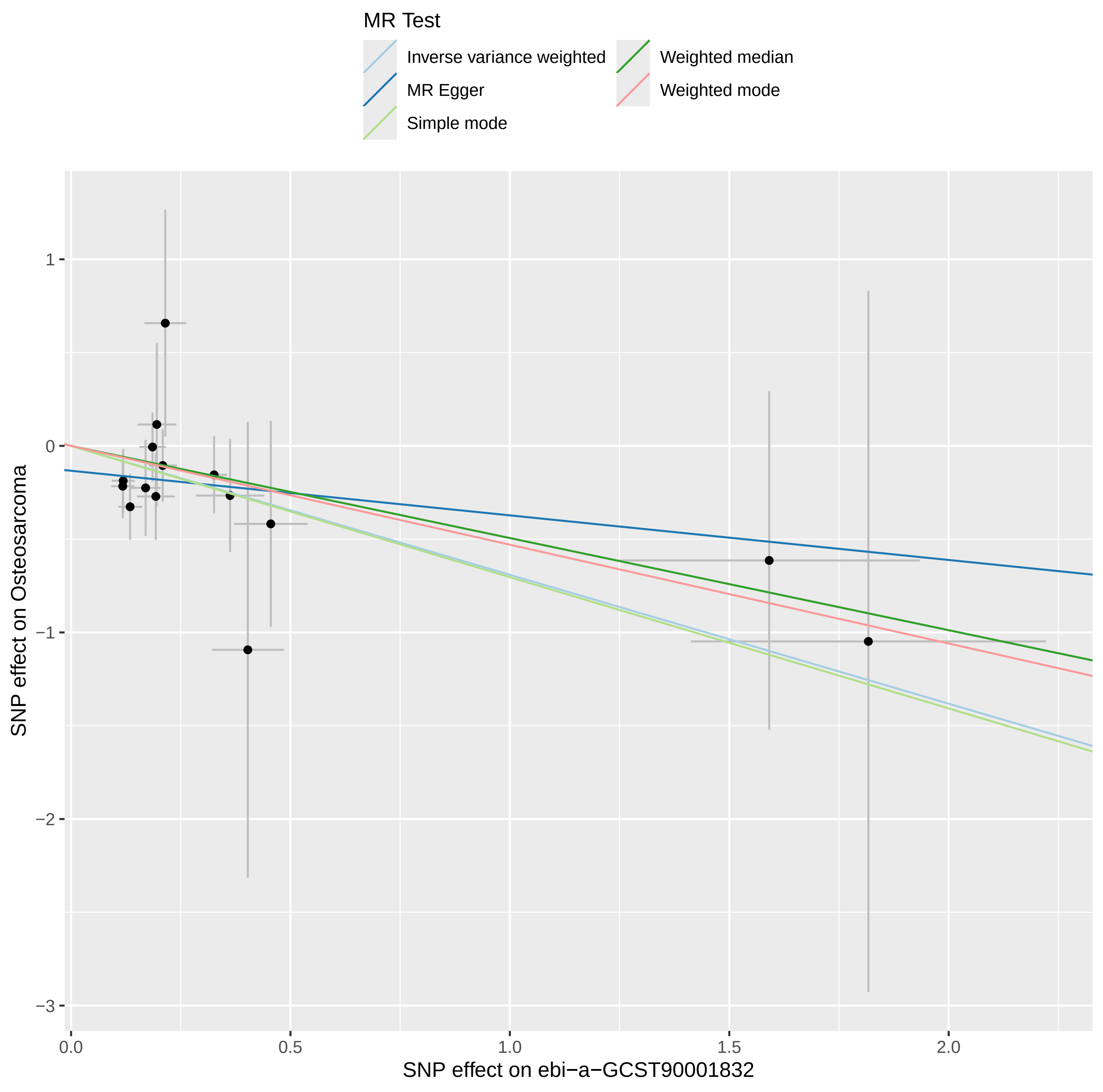

MR Test
Inverse variance weighted
MR Egger
Simple mode
Weighted median
Weighted mode
1
0
−1
−2
−3
0.0
0.5
1.0
1.5
2.0
SNP effect on Osteosarcoma
SNP effect on ebi−a−GCST90001832

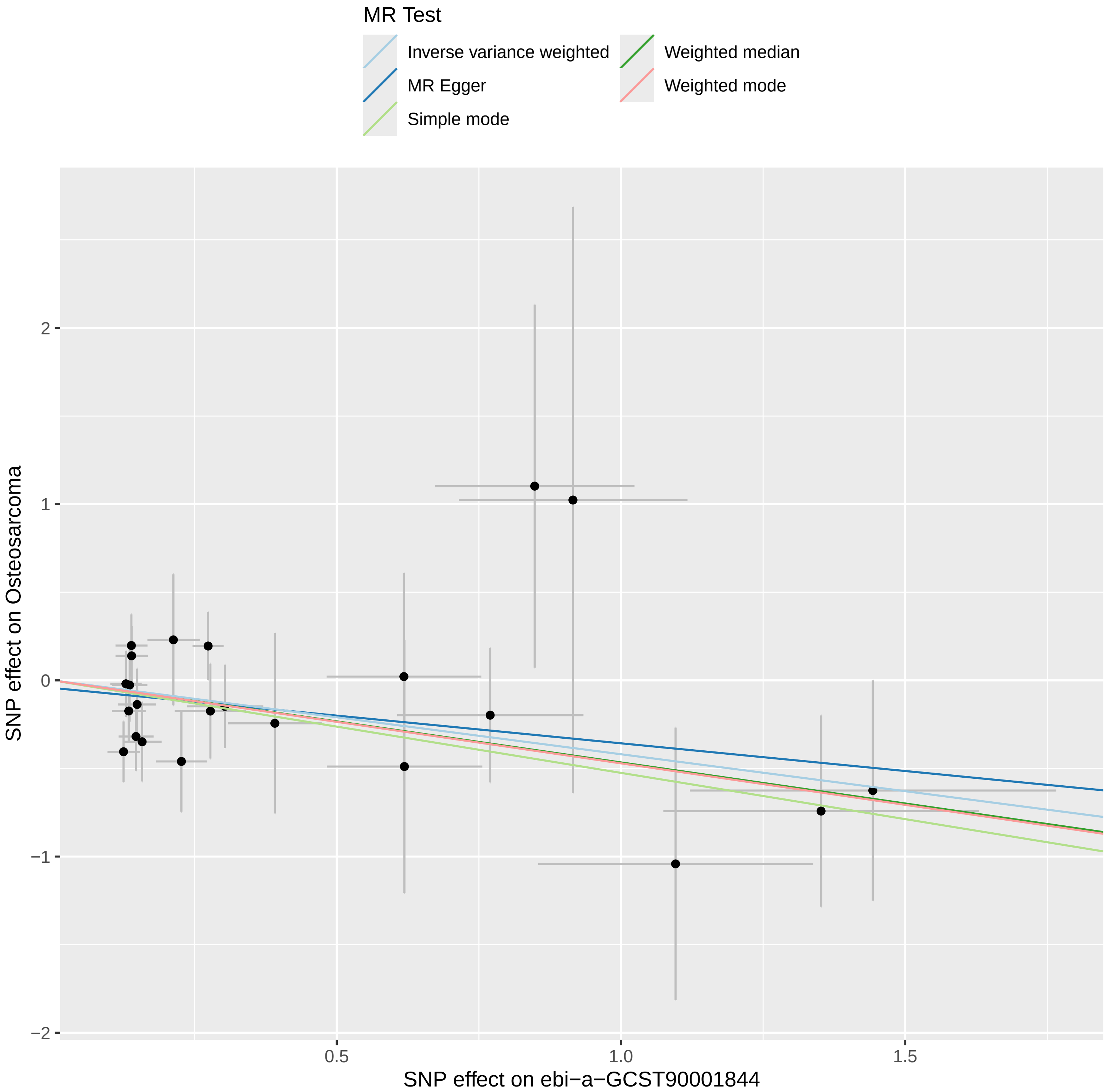

MR Test
Inverse variance weighted
MR Egger
Simple mode
Weighted median
Weighted mode
2
1
0
−1
−2
0.5
1.0
1.5
SNP effect on Osteosarcoma
SNP effect on ebi−a−GCST90001844

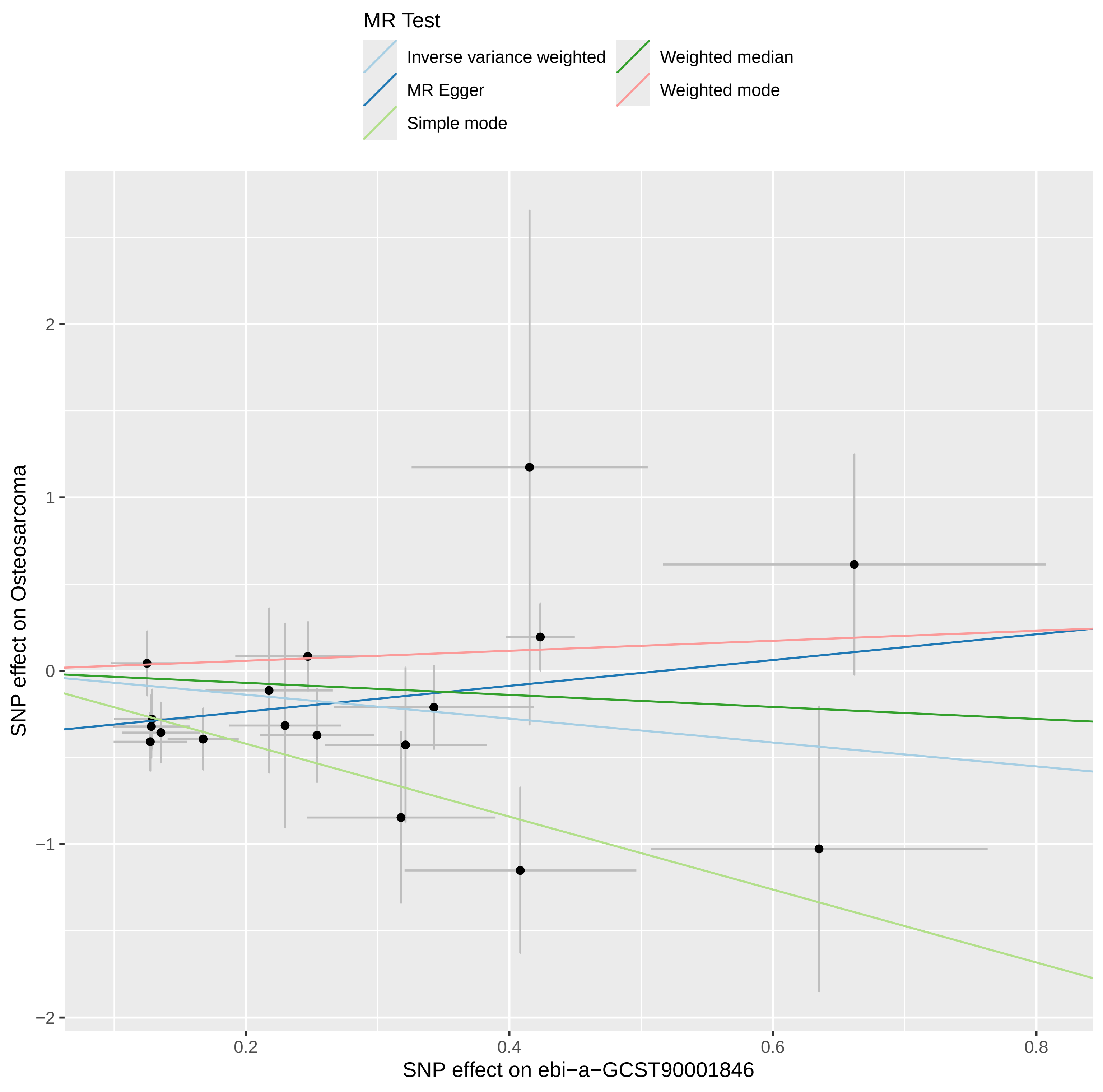

MR Test
Inverse variance weighted
MR Egger
Simple mode
Weighted median
Weighted mode
SNP effect on Osteosarcoma
2
1
0
−1
−2
0.2
0.4
0.6
0.8
SNP effect on ebi−a−GCST90001846

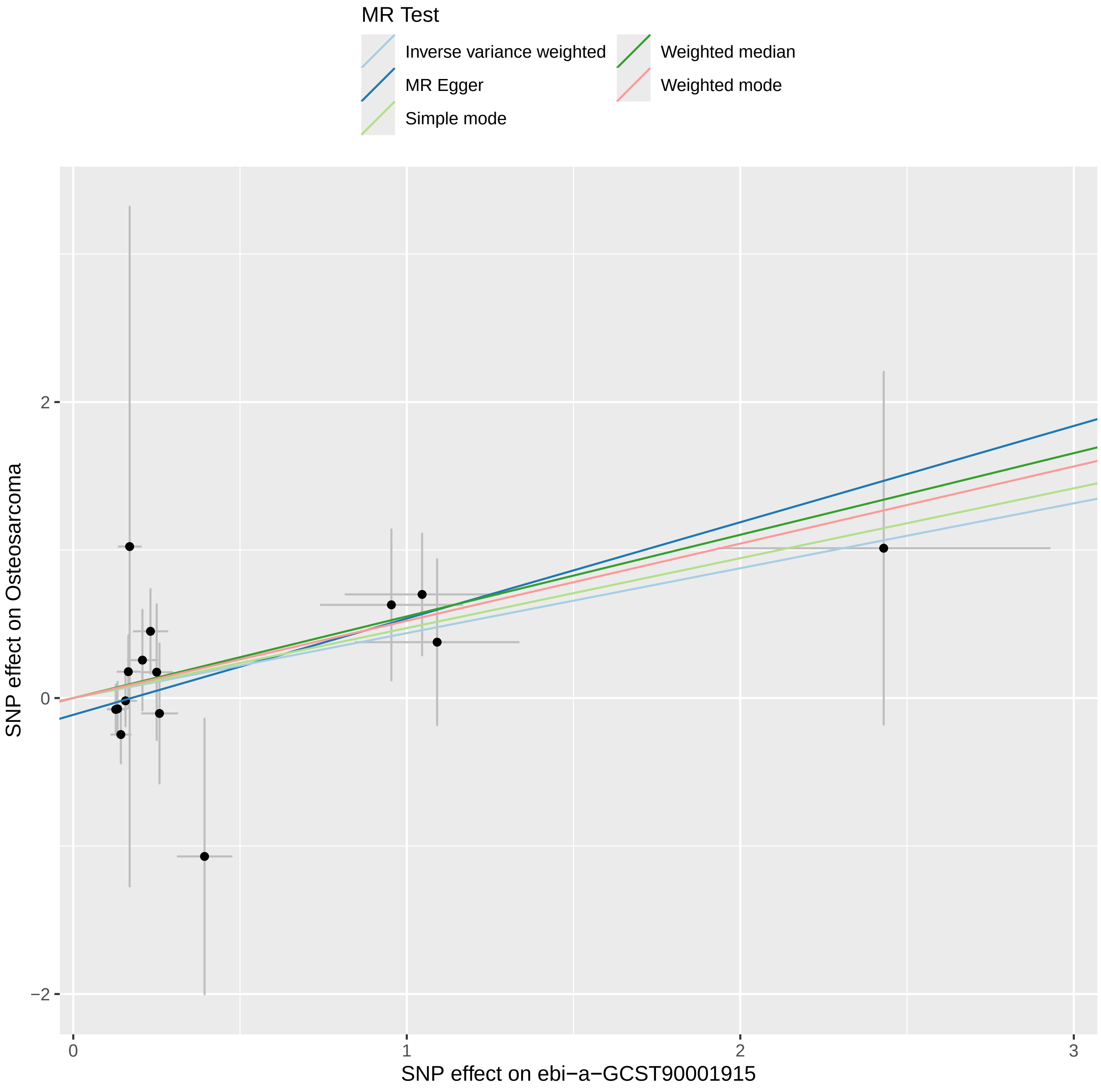
MR Test
Inverse variance weighted
MR Egger
Simple mode
Weighted median
Weighted mode
2
0
−2
0
1
2
3
SNP effect on Osteosarcoma
SNP effect on ebi−a−GCST90001915

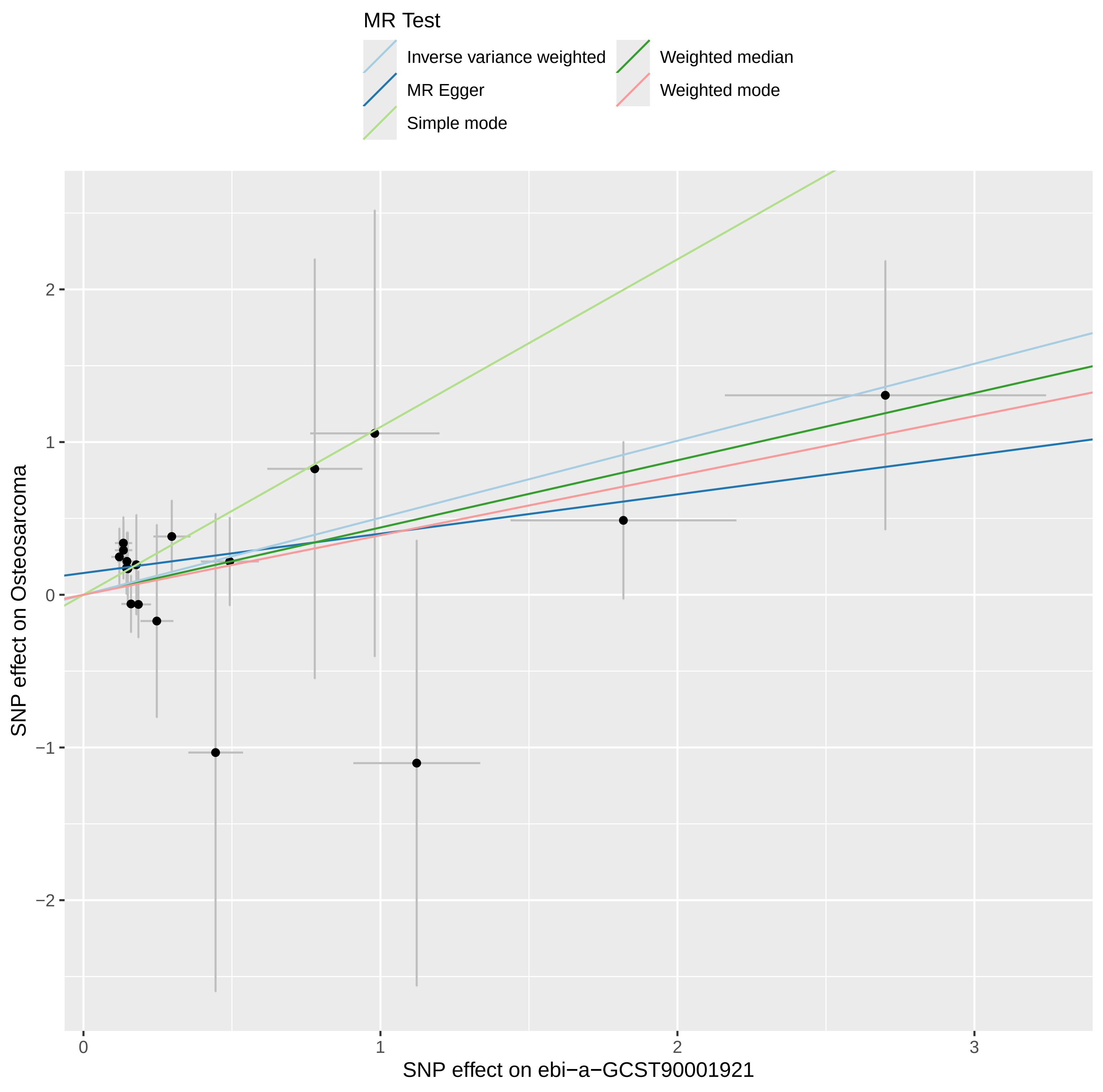

MR Test
Inverse variance weighted
MR Egger
Simple mode
Weighted median
Weighted mode
SNP effect on Osteosarcoma
SNP effect on ebi−a−GCST90001921
2
1
0
−1
−2
0
1
2
3

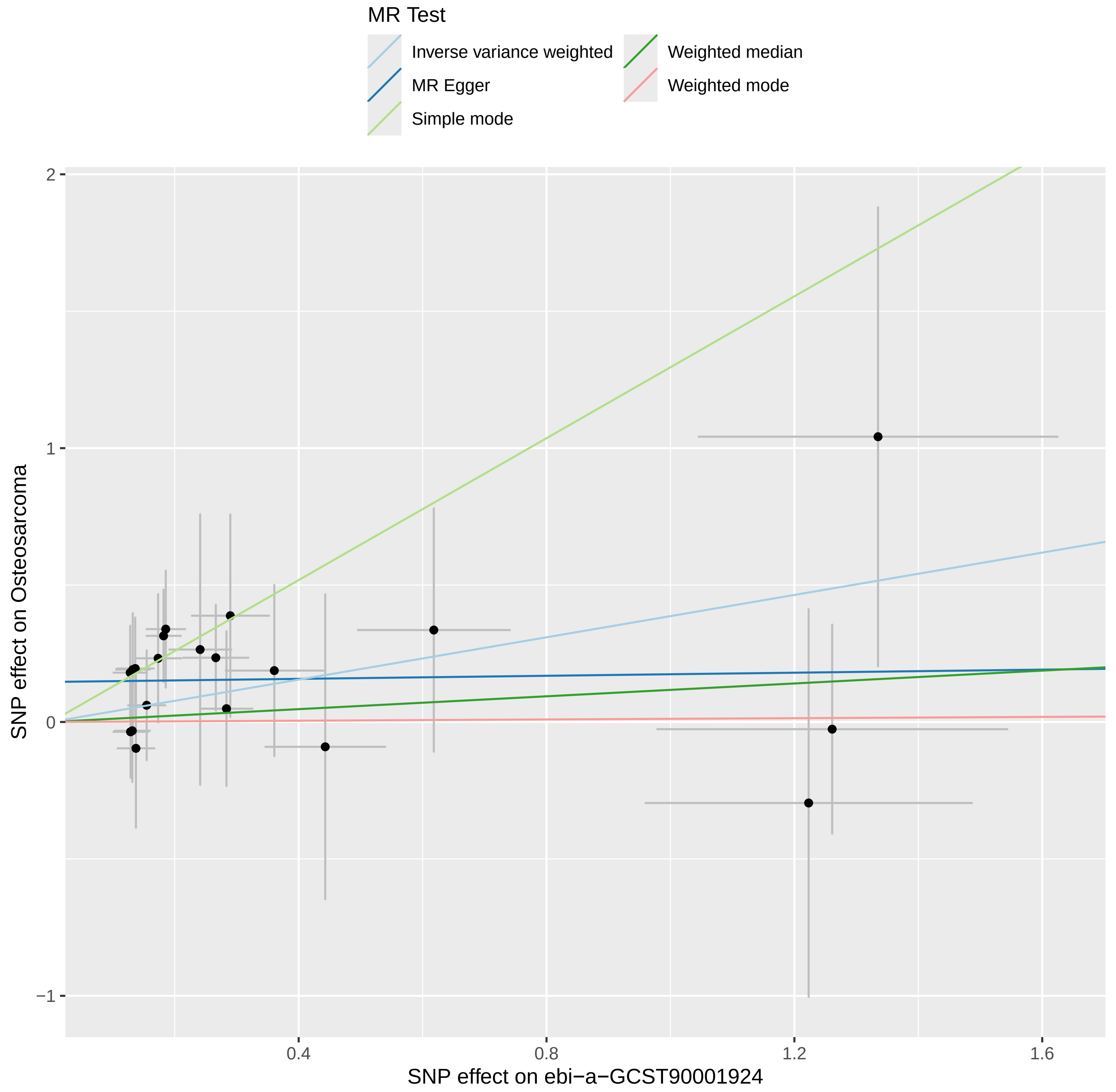

MR Test
Inverse variance weighted
MR Egger
Simple mode
Weighted median
Weighted mode
2
1
0
−1
SNP effect on Osteosarcoma
0.4
0.8
1.2
1.6
SNP effect on ebi−a−GCST90001924

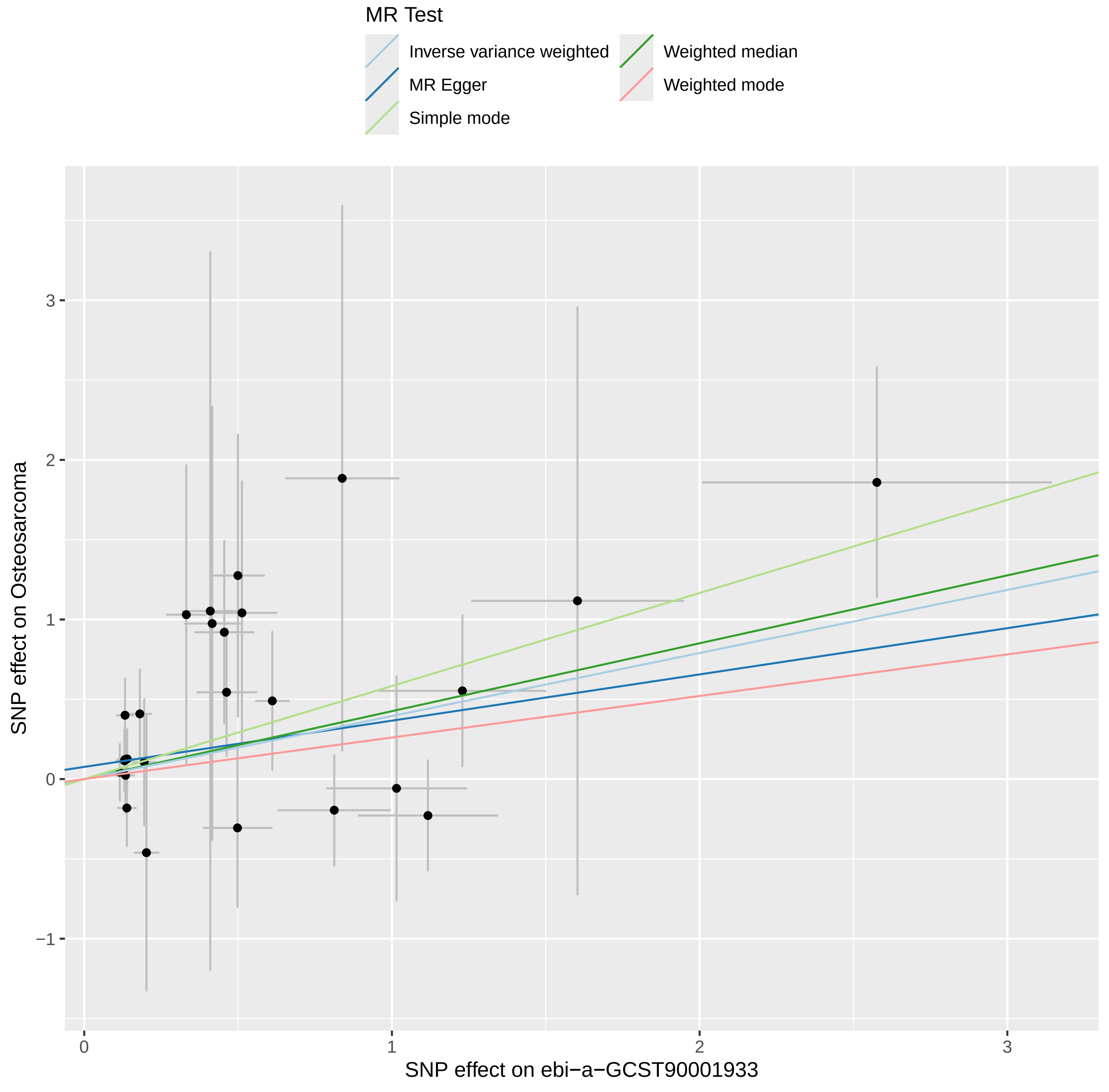
MR Test
Inverse variance weighted
MR Egger
Simple mode
Weighted median
Weighted mode
SNP effect on Osteosarcoma
SNP effect on ebi−a−GCST90001933
3
2
1
0
−1
0
1
2
3

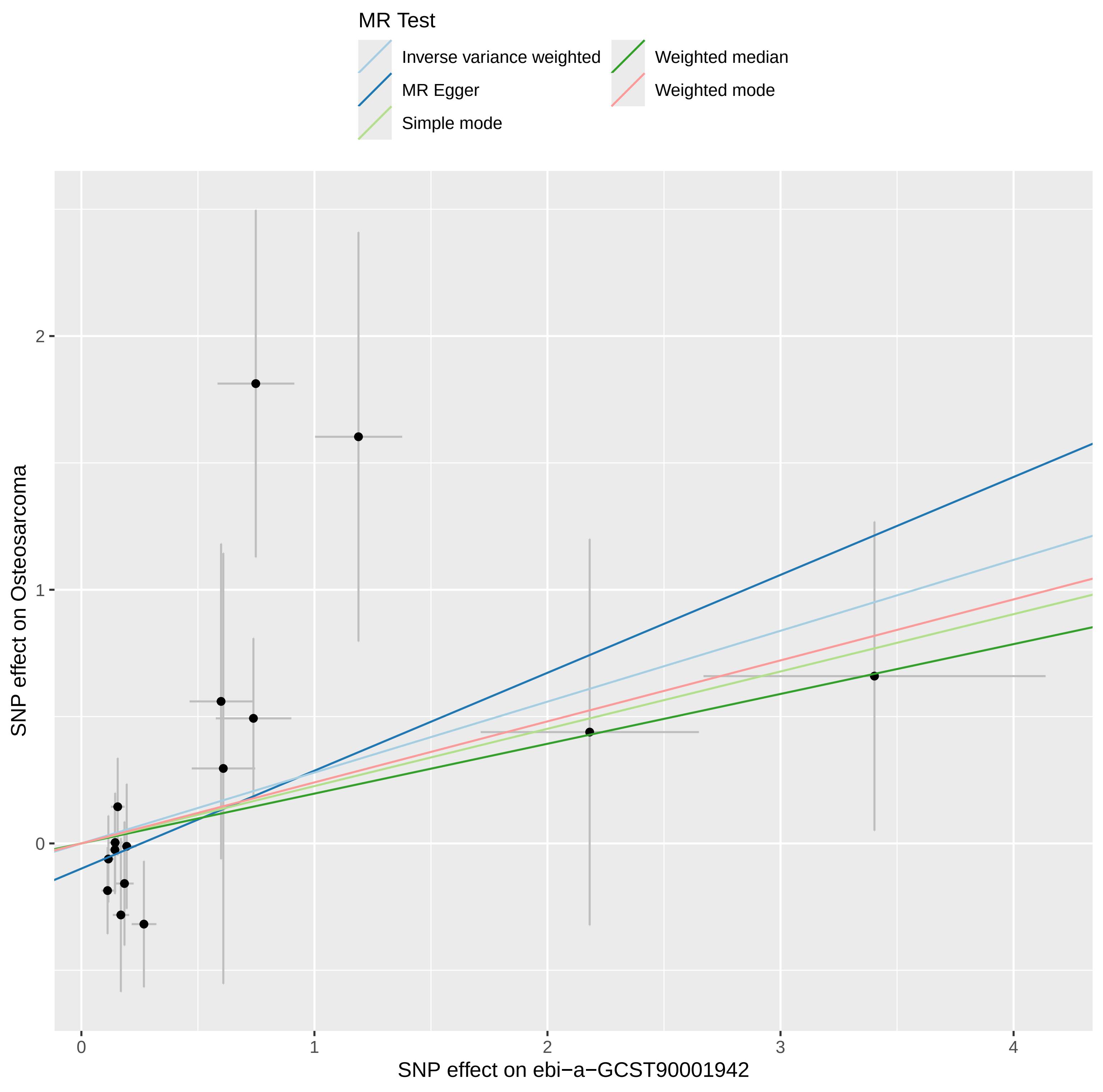

MR Test
Inverse variance weighted
MR Egger
Simple mode
Weighted median
Weighted mode
SNP effect on Osteosarcoma
SNP effect on ebi−a−GCST90001942
0
1
2
3
4

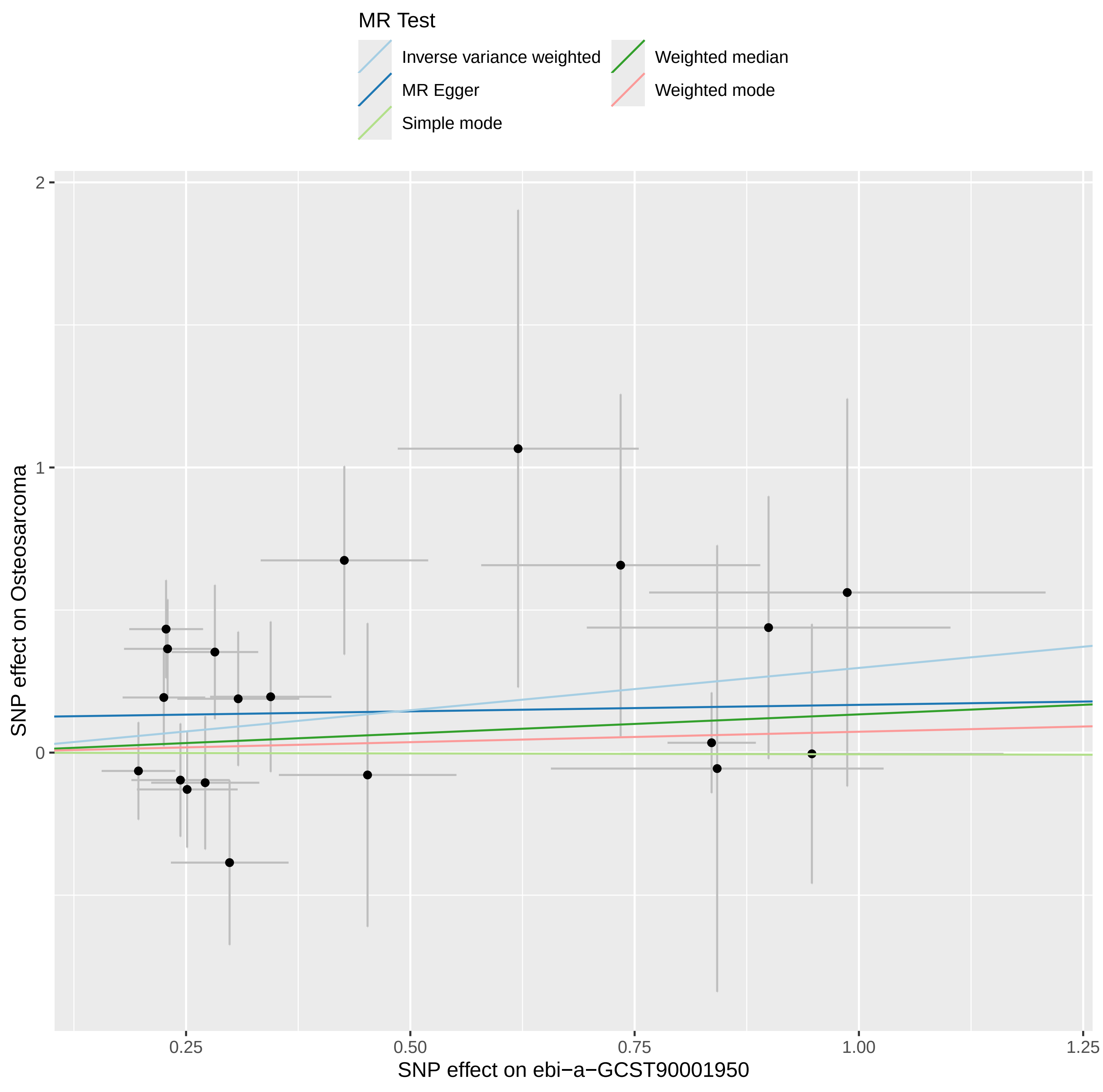

MR Test
Inverse variance weighted
MR Egger
Simple mode
Weighted median
Weighted mode
2
1
0
0.25
0.50
0.75
1.00
1.25
SNP effect on Osteosarcoma
SNP effect on ebi−a−GCST90001950

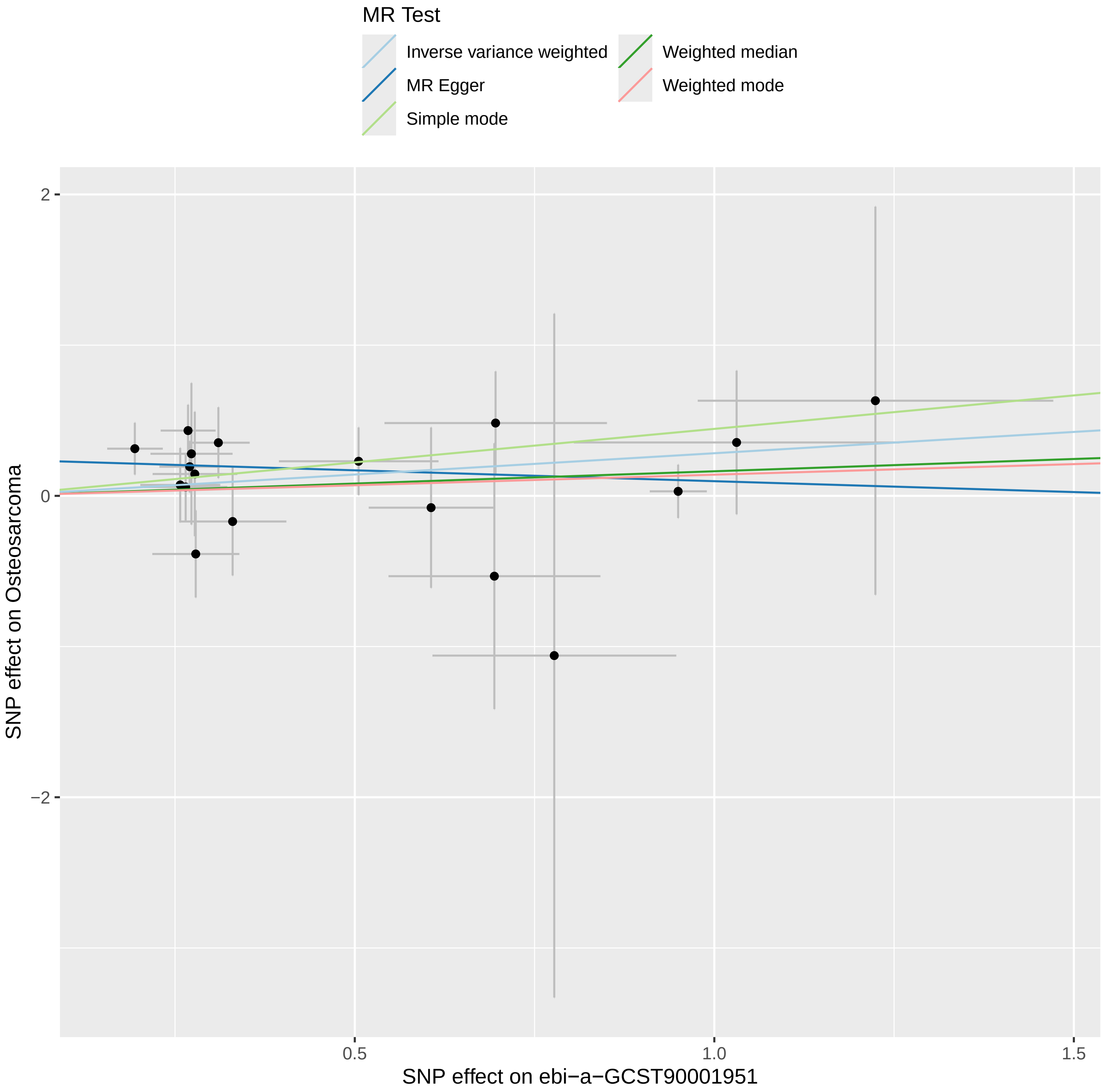
MR Test
Inverse variance weighted
MR Egger
Simple mode
Weighted median
Weighted mode
SNP effect on Osteosarcoma
2
0
−2
0.5
1.0
1.5
SNP effect on ebi−a−GCST90001951

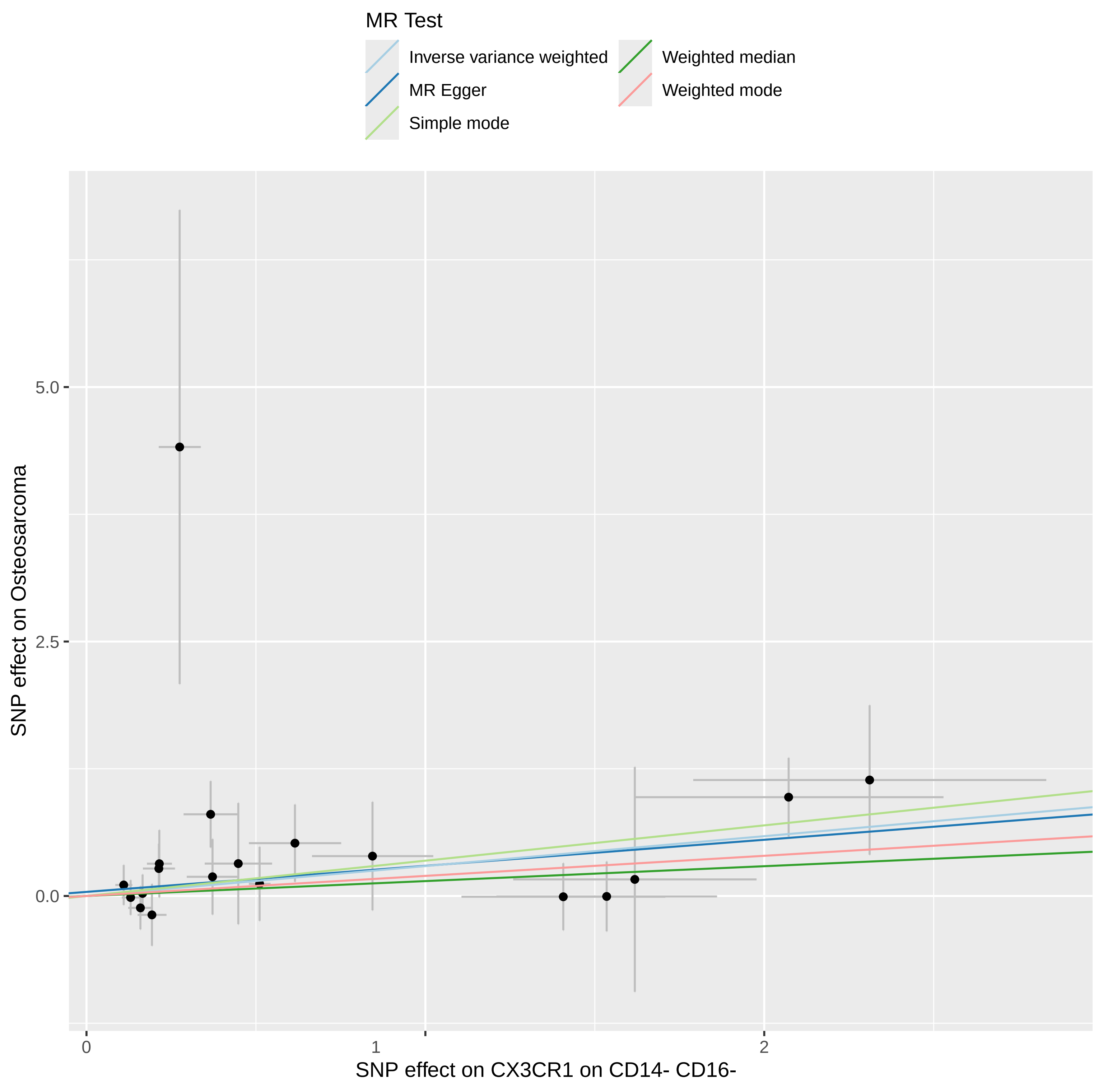

MR Test
Inverse variance weighted
MR Egger
Simple mode
Weighted median
Weighted mode
5.0
2.5
0.0
0
1
2
SNP effect on Osteosarcoma
SNP effect on CX3CR1 on CD14- CD16-

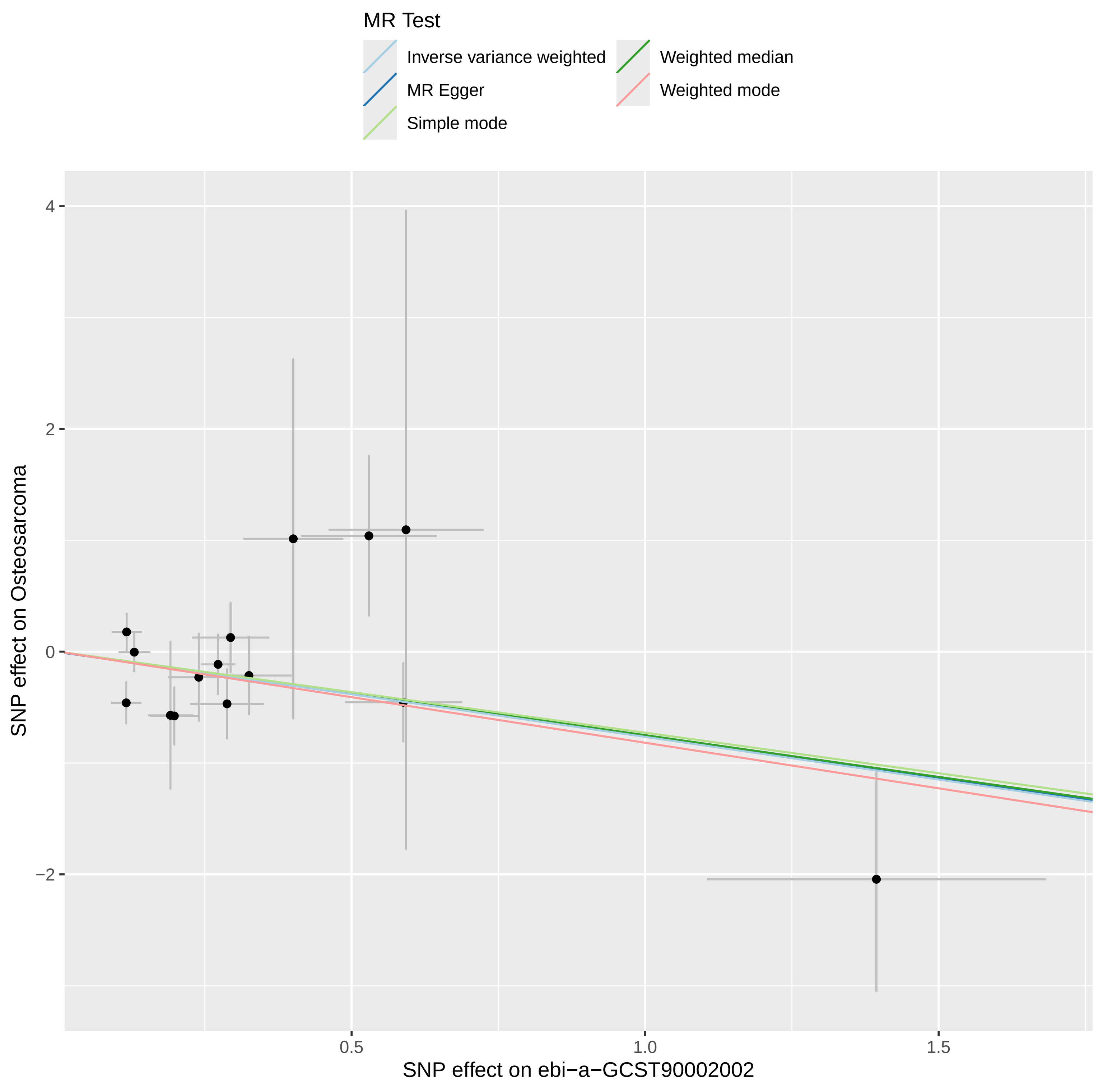

MR Test
Inverse variance weighted
MR Egger
Simple mode
Weighted median
Weighted mode
SNP effect on Osteosarcoma
4
2
0
−2
0.5
1.0
1.5
SNP effect on ebi−a−GCST90002002

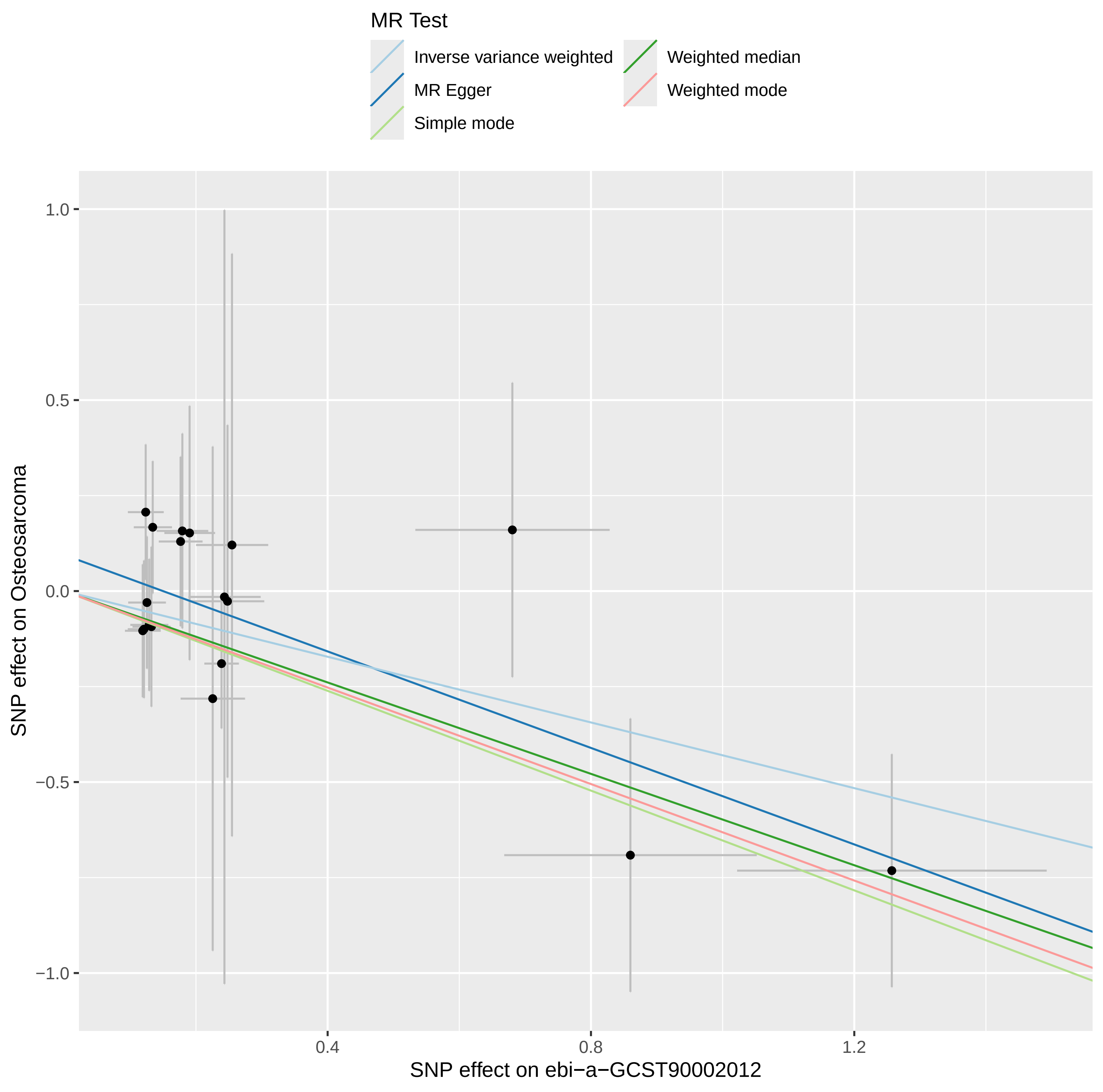

MR Test
Inverse variance weighted
MR Egger
Simple mode
Weighted median
Weighted mode
1.0
0.5
0.0
−0.5
−1.0
0.4
0.8
1.2
SNP effect on Osteosarcoma
SNP effect on ebi−a−GCST90002012

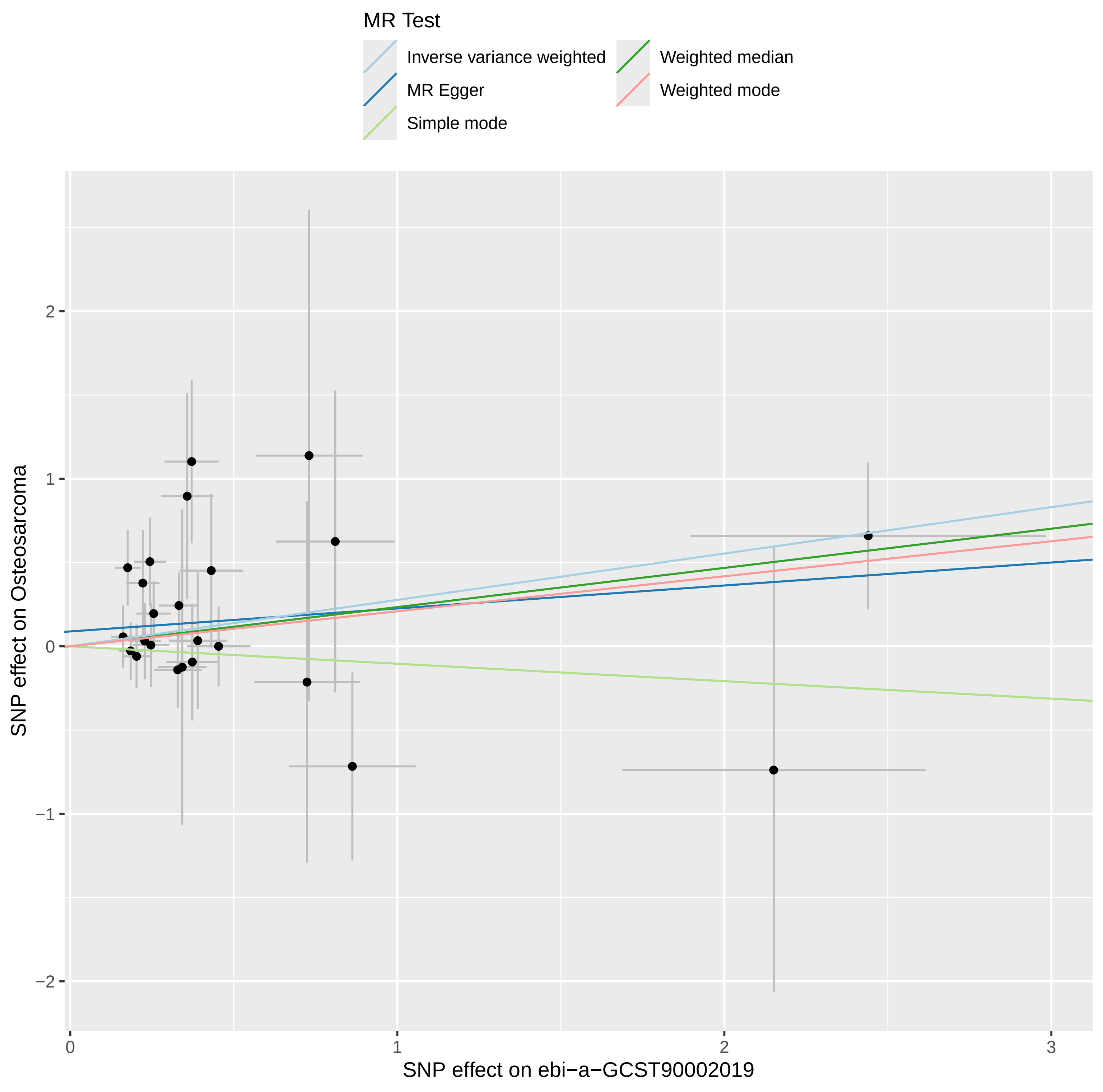

MR Test
Inverse variance weighted
MR Egger
Simple mode
Weighted median
Weighted mode
SNP effect on Osteosarcoma
SNP effect on ebi−a−GCST90002019
2
1
0
−1
−2
0
1
2
3

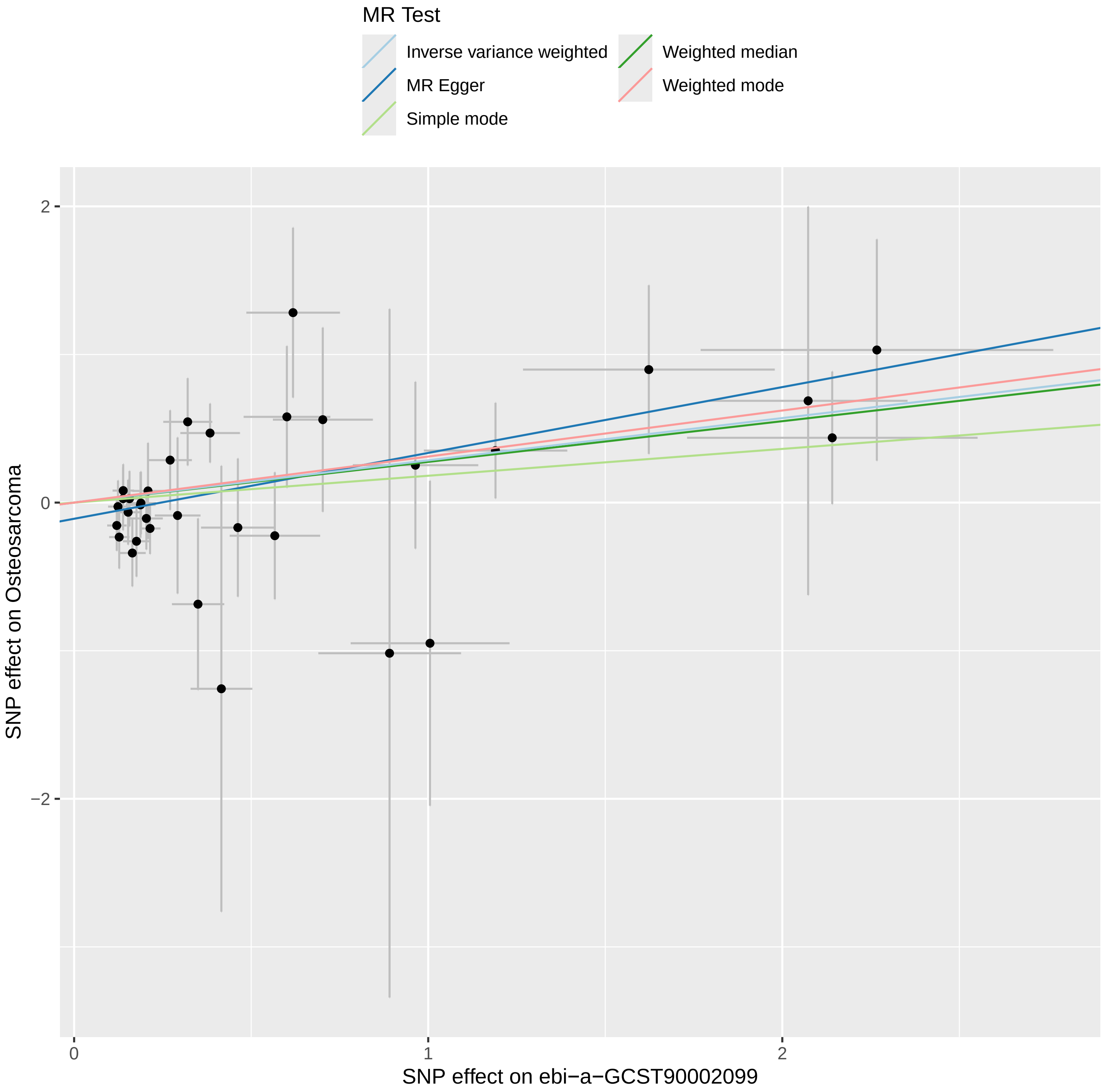

MR Test
Inverse variance weighted
MR Egger
Simple mode
Weighted median
Weighted mode
2
0
−2
0
1
2
SNP effect on Osteosarcoma
SNP effect on ebi−a−GCST90002099

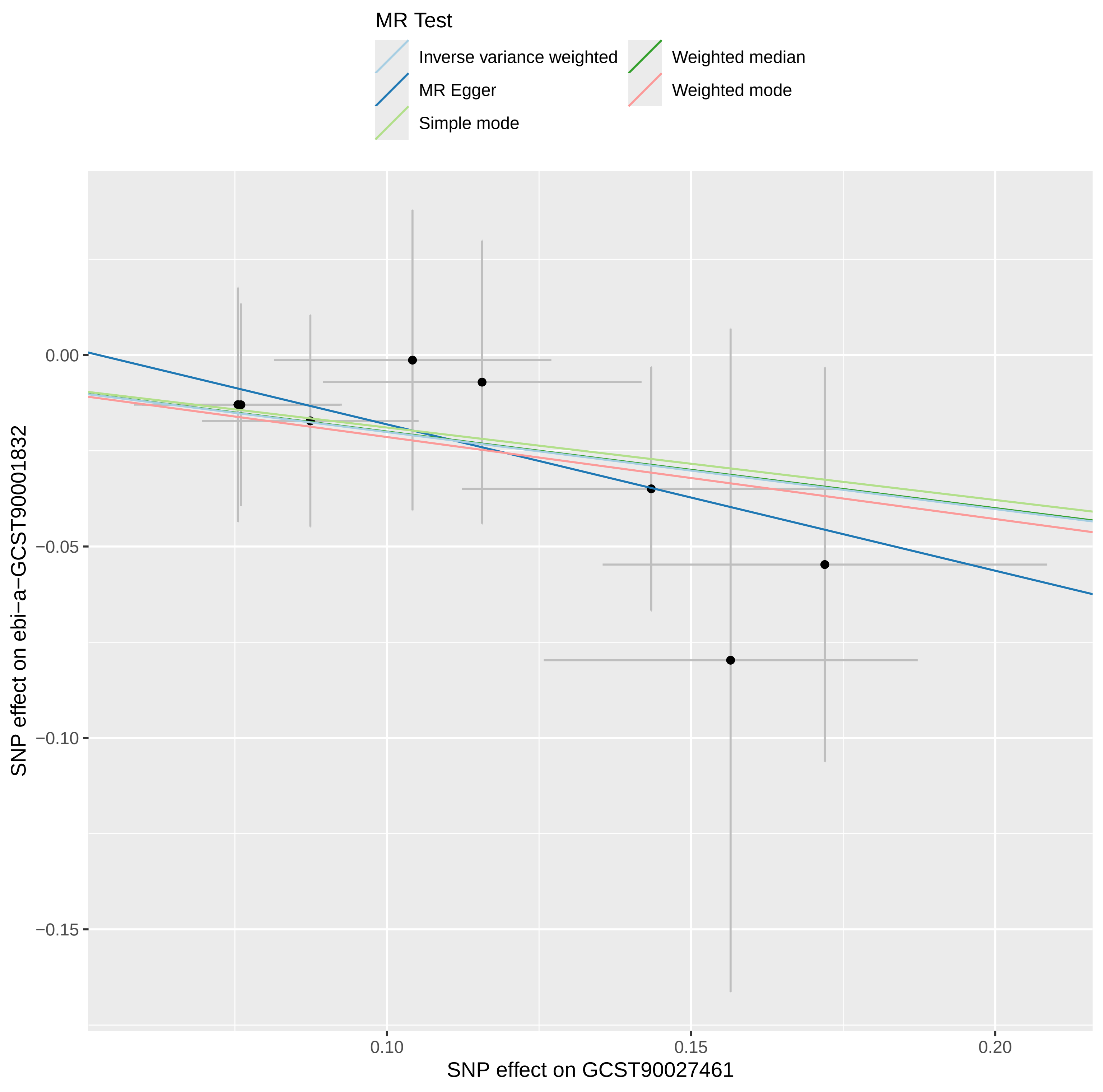

MR Test
Inverse variance weighted
MR Egger
Simple mode
Weighted median
Weighted mode
SNP effect on ebi−a−GCST90001832
0.00
−0.05
−0.10
−0.15
0.10
0.15
0.20
SNP effect on GCST90027461

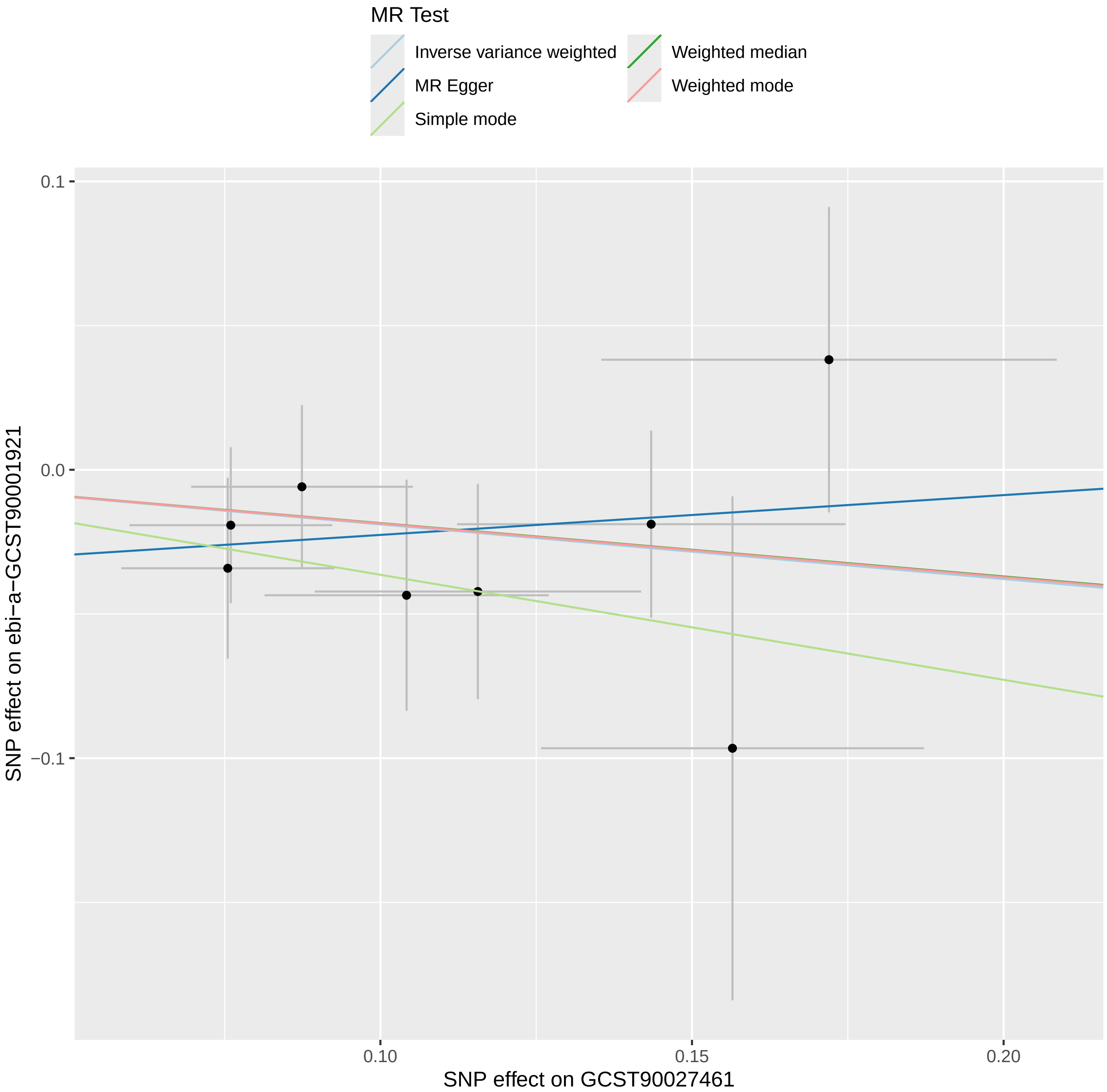

MR Test
Inverse variance weighted
MR Egger
Simple mode
Weighted median
Weighted mode
0.1
0.0
−0.1
0.10
0.15
0.20
SNP effect on GCST90027461
SNP effect on ebi−a−GCST90001921

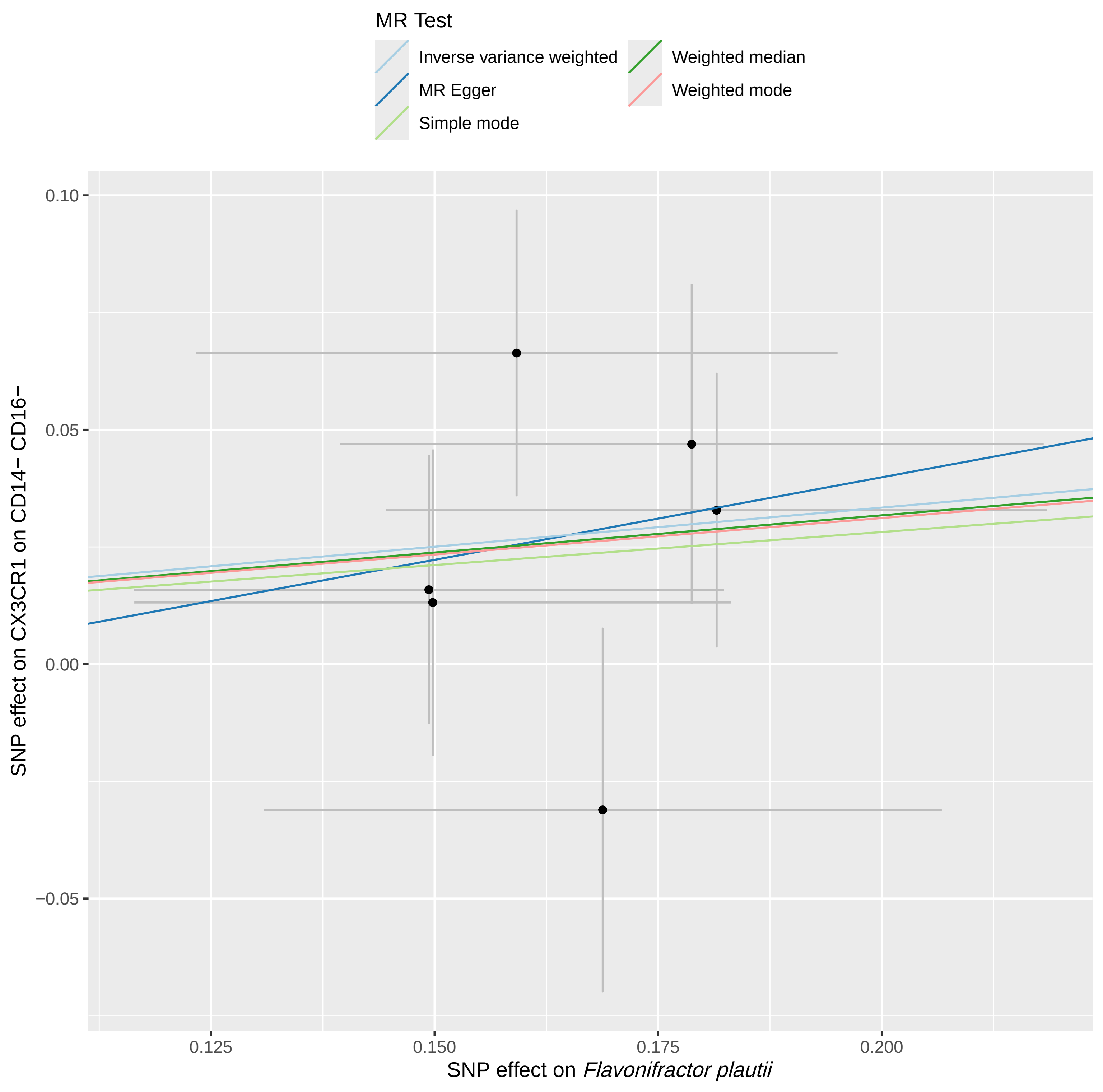

MR Test
Inverse variance weighted
MR Egger
Simple mode
Weighted median
Weighted mode
0.10
0.05
0.00
−0.05
0.125
0.150
0.175
0.200
SNP effect on CX3CR1 on CD14− CD16−
SNP effect on *Flavonifractor plautii*
